# Photoredox-controlled chemo-divergent, regio-, and stereoselective difunctionalization of olefins via switchable SO_2_ reintegration

**DOI:** 10.1126/sciadv.aeb0206

**Published:** 2025-12-19

**Authors:** Jiuli Xia, Lihan Zhu, Zhiguang Lv, Yunliang Guo, Lefeng Lin, Jiaqiong Sun, Guangfan Zheng, Qian Zhang

**Affiliations:** ^1^ Department of Chemistry, Northeast Normal University, Changchun 130024, China.; ^2^ School of Environment, Northeast Normal University, Changchun 130117, China.; ^3^ State Key Laboratory of Organometallic Chemistry, Shanghai Institute of Organic Chemistry, Chinese Academy of Sciences, 345 Lingling Lu, Shanghai 200032, China.

## Abstract

While radical difunctionalization offers substantial potential for molecular diversification, existing methodologies predominantly focus on styrenes and activated alkenes, leaving fundamental light olefins (e.g., ethylene and propylene) largely unexplored. Moreover, achieving stereoselective transformations of light olefins remains a formidable challenge. Here, we present a visible-light–mediated photoredox-catalyzed chemo-divergent and regioselective radical difluoromethylation/polyfluoroarylsulfonylation and difluoromethylation/polyfluoroarylation of structurally diverse alkenes through switchable sulfur dioxide reintegration. The method demonstrates broad applicability across styrenes, unactivated alkenes, and gaseous ethylene/propylene substrates. The photocatalyst functions as a switch, and Sodium difluoromethanesulfinate (CF_2_HSO_2_Na) acts as a bifunctional reagent, enabling controllable divergence radical conversion via radical-polar crossover pathways. Notably, chiral alcohol–derived polyfluoroarenes efficiently induce stereoselectivity control in the coupling of alkyl radicals with sulfur dioxide via dynamic kinetic resolution. Density functional theory calculations indicate noncovalent interaction between alkyl/aryl groups and polyfluorocarbons plays critical roles for the ultralong-distance (>9 atoms) stereochemical induction. The key to achieving chemo-divergent and stereocontrolled transformations lies in the precise sorting of a dynamic intermediate pool containing alkyl radicals, sulfonyl radicals, and anions.

## Teaser

Unprecedented stereoselective coupling of alkyl radicals with SO_2_ using the Curtin-Hammett principle is discussed.

## INTRODUCTION

**Fig. 1.**
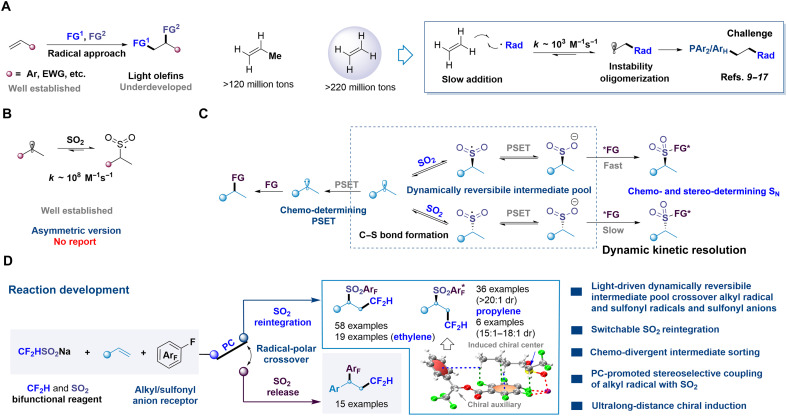
Motivation for stereoselective radical difluoromethylation/switchable SO_2_ reintegration/polyfluoroarylation cascade of alkenes. (**A**) Radical difunctionalization of olefins. (**B**) Trapping of alkyl radicals by SO_2_. (**C**) Our strategy: decoupling of radical-trapping process with stereodetermining step via dynamically reversible intermediate pool. (**D**) This work: PC-controlled stereoselective switchable SO_2_ reintegration cascade. PSET, photoinduced single electron transfer; FG, Functional group.

Olefins, particularly unactivated variants, serve as critical raw materials in chemical synthesis due to their large-scale availability from both petrochemical and renewable sources. Light olefins such as ethylene (*1*) and propylene (*2*) exemplify this significance, with annual global production exceeding 220 million and 120 million metric tons in 2022, respectively, establishing them as cornerstone feedstocks in modern industrial chemistry. The difunctionalization of olefins—the simultaneous incorporation of two functional groups across a double bond—represents a powerful strategy for constructing molecular complexity and diversity. Radical-mediated approaches (*3*–*7*) have emerged as particularly promising, enabling bond formation under mild conditions with notable reactivity and selectivity. However, current methodologies predominantly focus on styrenes or electronically activated olefins (Fig. 1A). For light olefins, the inherent nonpolar nature and instability of aliphatic radicals compared with benzylic ones lead to sluggish radical addition (*8*) processes. Ethylene presents additional challenges: The transient primary alkyl radicals generated during its functionalization lack stabilizing substituents, leading to undesired polymerization or oligomerization (*9*, *10*). Mita and colleagues (*11*) addressed this challenge through a radical chain mechanism for ethylene difunctionalization, albeit requiring elevated pressures to enhance reactivity. For nonchain systems, rapid trapping of unstable primary radicals (*12*–*17*) becomes essential to prevent side reactions. In this domain, Zhu and Wu’s research groups developed a docking-migration protocol (*12*, *13*) for installing alkyl and heteroaryl groups on ethylene frameworks. Wu and coworkers (*14*, *15*) pioneered transition metal–catalyzed four-component radical carbonylation of ethylene. Most recently, the Wu group (*16*) achieved a breakthrough in radical arylative difunctionalization through polarity-matched Minisci-type coupling using electron-deficient arenes as effective radical acceptors. Despite these milestones, the development of broadly applicable catalytic platforms for chemo-divergent and stereo-controlled difunctionalization of alkenes, particularly gaseous C_2_–C_3_ olefins, remains an unmet challenge.

Sulfur dioxide (SO_2_) emerges as a compelling candidate for radical trapping due to its stabilized singly occupied molecular orbital, enabling rapid (*18*) and reversible (*19*) radical addition to generate sulfonyl radical intermediates. While notable progress has been made in this field (*20*–*28*), the asymmetric construction of C(sp^3^)─S bonds through radical-SO_2_ coupling remains unexplored (Fig. 1B). The inherently low coupling barrier between alkyl radicals and SO_2_ facilitates uncontrolled background recombination in the absence of stereochemical guidance. Photocatalytic systems (*29*, *30*) exhibit the capacity to interconvert sulfonyl species through redox cycling, where sulfonyl radicals undergo photoreduction to sulfonyl anions (*25*–*27*) while remaining susceptible to photooxidation back to radical states (*28*). We propose that rationally designed photoredox cycles could establish a dynamic intermediate reservoir, thereby enabling dynamic equilibrium among alkyl radicals, sulfonyl radicals, and anionic species within a unified catalytic framework (Fig. 1C). Selective transformations of sulfonyl anions with electrophile may overcome key challenges in the radical difunctionalization of light olefins through a controlled radical-polar crossover strategy. The reduction of alkyl radicals to alkyl anions may yield SO_2_-released products, a process potentially modulated by the photocatalyst. Moreover, the incorporation of chiral auxiliary groups may offer opportunities for precise stereochemical control in alkyl radical–SO_2_ coupling by facilitating stereocontrollable transformations of sulfonyl anions.

As part of our continued interest in visible-light catalysis (*31*–*34*) and C─F bond activation (*35*), we now report a photoredox-controlled switchable introduction of two distinct drug-relevant fluorinated groups (*36*, *37*) difluoromethyl (CF_2_H) (*38*) and polyfluoroarenes (*39*, *40*) into olefin π-systems (styrenes, nonactivated alkenes, and gaseous ethylene/propylene substrates) using CF_2_HSO_2_Na (*41*–*47*) as a bifunctional CF_2_H radical and SO_2_ source (*48*–*53*) and polyfluoroarenes (*39*, *40*) as terminating reagents via selective C─F bond cleavage (*39*, *40*) (Fig. 1D). In situ–generated catalytic amounts of SO_2_ (*54*–*60*) reversibly trap alkyl radicals, enabling direct access to either β-CF_2_H–substituted polyfluoroaryl sulfones (*61*, *62*) or β-CF_2_H–substituted polyfluoroarenes through switchable SO_2_ reintegration (*57*) in a dynamically reversible equilibrium. Glorius and co-workers (*57*) reported an Ent-induced chemo-divergent difunctionalization (*63*, *64*) of alkenes involving a switchable reintegration of SO_2_ controlled by bases through their SO_2_-absorbing capability; our findings complementary with their system. The incorporation of chiral auxiliaries into polyfluoroarenes effectively modulates asymmetric difluoromethylation/sulfonylation via ultralong-distance stereochemical induction. Crucially, the precise sorting (*65*–*70*) of enantiomeric intermediates from the dynamic equilibrium pool via radical-polar crossover (*71*, *72*) could effectively bypass the intrinsic stereochemical limitations of radical-SO_2_ recombination. A key advantage of this strategy is the decoupling of the bond-forming step from the stereodetermining step (*73*, *74*), thereby providing an attractive solution for asymmetric transformations involving highly reactive radical species, which are often synthetically challenging to control.

## RESULTS

### Reaction optimization

**Fig. 2.**
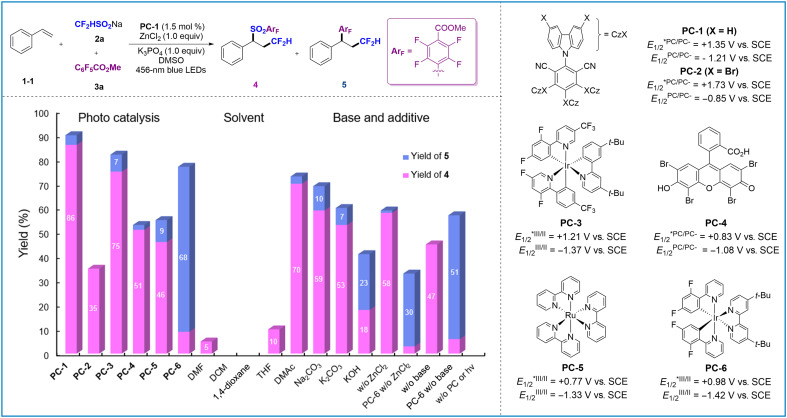
Optimization of reaction conditions. Unless otherwise noted, all the reactions were carried out with **1** (0.2 mmol, 1.0 equiv.), **2** (0.4 mmol, 2.0 equiv.), **3** (0.4 mmol, 2.0 equiv.), base (0.2 mmol, 1.0 equiv), PC (1.5 mol %), and ZnCl_2_ (0.2 mmol, 1.0 equiv) in dry solvent (2 ml), irradiated with blue LEDs at room temperature (rt) for 48 hours. w/o, without. Ar_F_, polyfluoroarylhv, light irradiation.

We selected styrene (**1-1**) as an olefin starting material, CF_2_HSO_2_Na (**2a**) as bifunctional reagent, and methyl pentafluorobenzoate (**3a**) as an arylation source. After screening reaction conditions (Fig. 2 and table S1), the desired sulfone **4** was isolated in 86% yield when 4CzIPN (1.5 mol %), ZnCl_2_ (1.0 equiv), and K_3_PO_4_ (1.0 equiv) were used under irradiation with blue light-emitting diodes (LEDs) in dimethyl sulfoxide (DMSO) for 48 hours (conditions A), with trace amount of difluoromethylation/polyfluoroarylation product **5**. The photocatalyst screening results indicate that **PC-3** achieved a comparable reaction efficiency. However, a significant decrease in yield was observed for **PC-2**, **PC-4**, and **PC-5**. The use of **PC-6** as a photocatalyst resulted in reversed chemoselectivity, yielding 68% of product **5** and 9% of product **4** (conditions B). The solvent screening shows that only *N*, *N*-dimethylacetamide (DMAc) produces **4** with a good yield, while other solvents such as tetrahydrofuran (THF), dichloromethane (DCM), and *N*,*N*′-dimethylformamide (DMF) yield almost no product. Without K_3_PO_4_, the reaction becomes sluggish, and only 58% of product **4** was observed. Other bases such as Na_2_CO_3_ and K_2_CO_3_ were less efficient than K_3_PO_4_; adding KOH could improve the ratio of compound **5**, but it made the reaction sluggish. Both SO_2_ reintegration and release reactions can be carried out without K_3_PO_4_ and ZnCl_2_; however, these additives effectively increase the reaction rate. Last, the control experiment indicated that the photocatalyst and light irradiation were indispensable for the cascade transformation.

### Substrate scope for chemo-divergent difluoromethylation/switchable SO_2_ reintegration/polyfluoroarylation of alkenes

**Fig. 3.**
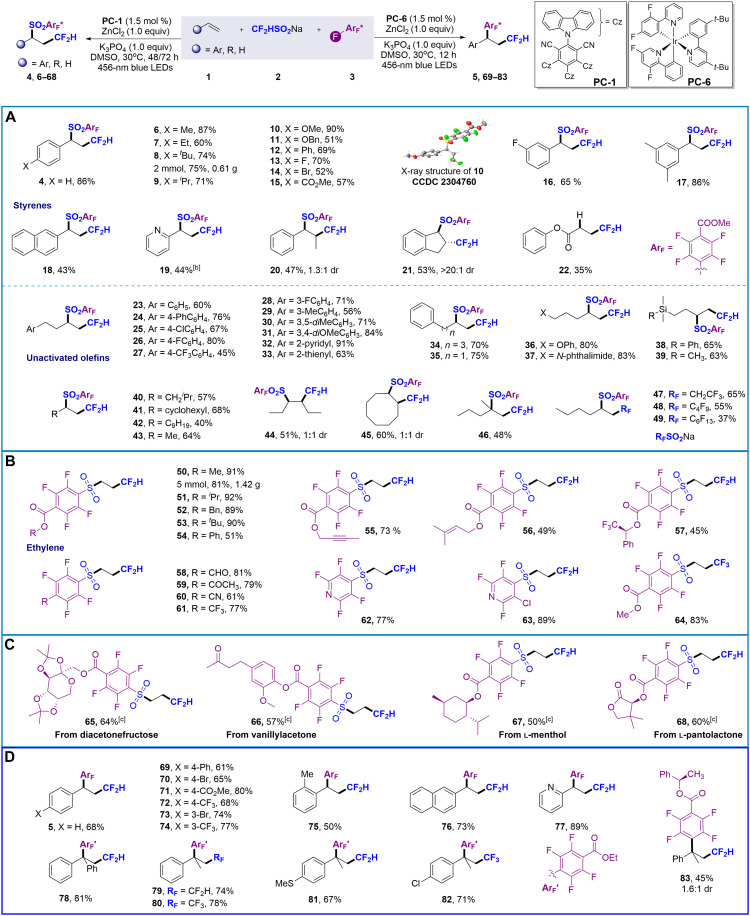
Substrate scope for radical chemo-divergent difluoromethylation/switchable SO_2_ reintegration/polyfluoroarylation of alkenes.^a^ (**A**) Substrate scope for styrenes and unactivated olefins. (**B**) Substrate scope for difluoromethylative sulfonylation of ethylene^[c]^. (**C**) Late-stage functionalization. (**D**) Substrate scope for stereoselective SO_2_ release difunctionalization of alkenes^[d]^. ^a^Condition A: Unless otherwise noted, all the reactions were carried out with **1** (0.2 mmol), **2** (0.4 mmol, 2.0 equiv), **3** (0.4 mmol, 2.0 equiv), K_3_PO_4_ (0.2 mmol, 1.0 equiv), **PC-1** (1.5 mol %), and ZnCl_2_ (0.2 mmol, 1.0 equiv) in dry DMSO (2 ml) at rt for 48 hours. Isolated yield. ^b^Reaction at 30°C. ^c^Condition C: Reactions were carried out with **2** (0.4 mmol), **3** (0.2 mmol), **PC-1** (1.5 mol %), and ethylene (1 atm; balloon) in dry DMSO (2 ml) at 30°C for 72 hours. ^d^Condition B: Unless otherwise noted, all the reactions were carried out with **1** (0.2 mmol), **2** (0.4 mmol, 2.0 equiv), **3** (0.4 mmol, 2.0 equiv), K_3_PO_4_ (0.2 mmol, 1.0 equiv), **PC-6** (1.5 mol %), and ZnCl_2_ (0.2 mmol, 1.0 equiv) in dry DMSO (2 ml) at 30°C for 12 hours. Isolated yield. h, hours.

After screening for reaction parameters, the substrate scope of the difluoromethylation/SO_2_ reintegration/polyfluoroarylation of olefins was explored, and the results were summarized in Fig. 3. First, the substrate scope for olefins was evaluated, and the applicative range of olefins is extensive (Fig. 3A). Styrene derivatives containing different patterns of substitutions can be converted into target products **4** to **17** in 51 to 90% yields under standard conditions. Heteroaromatic and fused-aromatic (naphthalene and pyridine) olefins are also considered suitable starting materials, delivering **18** and **19** in 43 and 44% yield, respectively. Further extension to internal alkenes was also explored. For instance, when β-methylstyrene was used as the substrate, the desired product **20** was obtained in 47% yield with 1.3:1 diastereomeric ratio (dr). Encouragingly, the employment of a cyclic aryl-substituted internal alkene, indene, afforded product **21** in 53% yield with >20:1 dr. Other internal alkenes, including trisubstituted (**1** to **63** to **1** to **65**) and tetrasubstituted (**1** to **66** and **1** to **67**) variants, were also examined but proved unreactive, which we attribute to steric hindrance. Reaction of electron-deficient alkenes afforded the corresponding hydrodifluoromethylation product **22** in 35% yield. We further attempted more challenging unactivated olefins. A wide range of electron-rich and electron-deficient substituents on the aryl ring in 4-aryl-1-butenes could undergo the difluoromethylation/sulfonylation process, furnishing an array of polyfluoroaryl-substituted sulfones **23** to **31** in moderate to good yields. In addition, heterocycles such as pyridine (**32**; 91%) and thiophene (**33**; 63%) can be compatible with the reaction. Chain length seems not to affect reactivity, as demonstrated by the formation of **34** and **35**. Allylarenes, prone to isomerization, can efficiently undergo 1,2-difunctionalization reactions, resulting in **35** with yield of 75%. Alkenes containing distal phenoxy (**36**; 80%), phthalimide (**37**; 83%), and silyl groups [**38** (65%) and **39** (63%)] can also be well compatible. This chemistry can also be applied to simple alkyl olefins, delivering multifunctionalized sulfones **40** to **45** in moderate yields. Gaseous propylene also has good reaction activity under atmospheric pressure. For internal olefins, **44** and **45** were produced with 51 and 60% yield, respectively, showing nearly 1:1 diastereoselectivity. The transformation was also effective for 1,1-dialkyl–substituted alkenes, affording product **46** in 48% yield. Nonetheless, substrates featuring 1,1-diaryl (**1** to **56**) or 1-aryl–1-alkyl (**1** to **57**) substitution proved unsuitable. Furthermore, evaluation of various sodium fluoroalkylsulfinates revealed that the 2,2,2-trifluoroethyl, C_4_F_9_, and C_6_F_13_ radicals were competent reaction partners (**47** to **49**; 37 to 65% yields), in contrast to the monofluoromethyl analog (from CH_2_FSO_2_Na), which remained inactive.

Next, we shift our focus to the highly challenging difunctionalization of ethylene (Fig. 3B). To our delight, desired product **50** could be isolated in 91% yield by using only ethylene balloons and eliminating the need for bubbling or pressurization (conditions C). Multifluoroarenes bearing electron-withdrawing groups such as ester carbonyl (**50** to **57**), aldehyde (**58**), carbonyl (**59**), cyano (**60**), and trifluoromethyl (**61**) groups were well tolerated and delivered para-substituted products in moderate to high yields. Polyfluoro-substituted pyridines were found to be valuable substrates, resulting in the formation of **62** and **63** with yields of 77 and 89%, respectively. When CF_3_SO_2_Na was used as bifunctional reagent, the product **64** was obtained with 83% yield. Functional groups sensitive to radical reactions, such as benzyl, olefin, alkynes, and aldehyde groups, are retained, indicating excellent chemoselectivity of the transformation. Gram-scale synthesis of **8** and **50** was carried out, indicating scalability of the mentioned methodology. The broad functional group compatibility and mild reaction conditions prompted exploring this reaction in applying late-stage functionalization of natural products. Polyfluoroarenes derived from diacetylrenin, vanillylacetone, menthol, or pantolactone can undergo difluoromethylation/sulfonylation cascade with to form **65** to **68** in 50 to 64% yield.

We further explored the substrate scope of the difluoromethylation/polyfluoroarylation cascade for olefins. As illustrated in Fig. 3C, a broad range of styrene derivatives bearing diverse substituents—including electron-donating, electron-withdrawing, and halogen groups at the para, meta, or ortho positions of the arene—underwent the cascade reaction smoothly, yielding products **5** and **69** to **75** in 50 to 80% yields. Notably, 2-naphthyl (**76**; 73%)– and 2-pyridine (**77**; 89%)– substituted alkenes demonstrated excellent compatibility with this transformation. Furthermore, 1,1-disubstituted styrenes were also viable substrates, generating **78** to **82** with comparable efficiencies (67 to 81%). Chiral polyfluorobenzates derived from (*R*)-1-phenylethan-1-ol exhibited lower stereoselectivity in the polyfluoroarylation step, as evidenced by the formation of **83** (45% yield, 1.6:1 dr). Despite unsatisfactory selectivity, this result provides an opportunity for modulating stereoselectivity using chiral auxiliary groups. However, alkyl alkenes were not suitable for this transformation.

### Substrate scope for stereoselective difluoromethylation/SO_2_ reintegration/polyfluoroarylation of alkenes

**Fig. 4.**
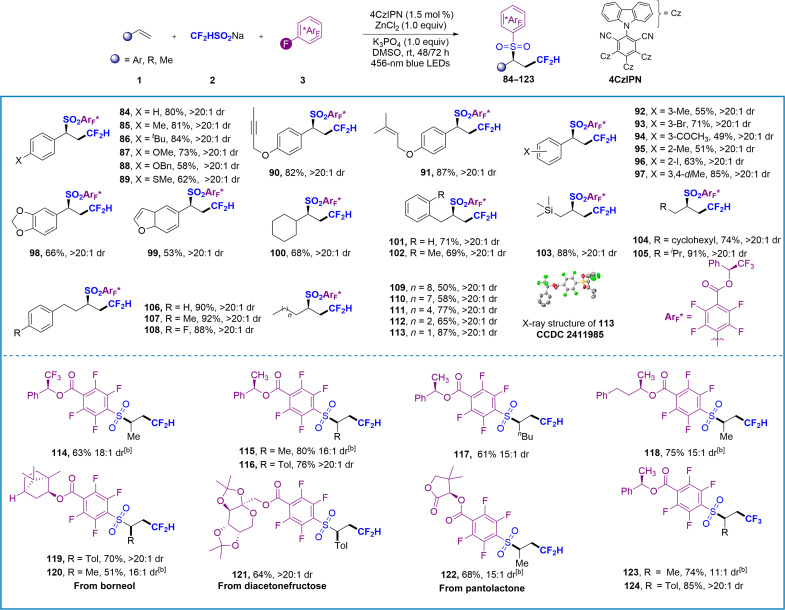
Substrate scope for stereoselective radical difluoromethylation/SO_2_ reintegration/polyfluoroarylation cascade of alkenes.^ac^ ^a^Condition A: Unless otherwise noted, all the reactions were carried out with **1** (0.2 mmol), **2** (0.4 mmol, 2.0 equiv), **3** (0.4 mmol, 2.0 equiv), K_3_PO_4_ (0.2 mmol, 1.0 equiv), 4CzIPN (1.5 mol %), and ZnCl_2_ (0.2 mmol, 1.0 equiv) in dry DMSO (2 ml) at rt for 48 hours. Isolated yield. ^b^Condition C: Reactions were carried out with 2 (0.4 mmol), 3 (0.2 mmol), 4CzIPN (1.5 mol %), and propylene (1 atm; balloon) in dry DMSO (2 ml) at 30°C for 72 hours. ^c^The dr values were determined by ^1^H nuclear magnetic resonance (NMR).

We further demonstrate the successful implementation of chiral alcohol–derived polyfluoroarenes as versatile stereocontrol reagents using noncovalent interactions (NCIs) in cascade transformations. Using (*S*)-2,2,2-trifluoro-1-phenylethanol–derived polyfluoroarene **3h** as substrate, the target compound **84** was obtained in 80% yield with exceptional diastereoselectivity (>20:1 dr), showcasing a stereoinduction effect. The reaction exhibits broad substrate generality across diverse olefins (Fig. 4). Styrene derivatives bearing electron-donating (alkyl, methoxy, benzyloxy, propargyloxy, allyloxy, and methylthio), halogen, or electron-withdrawing (carbonyl) substituents at various positions underwent efficient conversion to products **84** to **97** with 49 to 87% yields and excellent stereoselectivity. Notably, functional groups typically incompatible with transition metal catalysts (**89**; 62% yield) or prone to radical side reactions [internal alkynes in **90** (82%) and conjugated alkenes in **91** (87%)] demonstrated excellent compatibility. Heterocyclic systems including piperonyl ring and benzofuran-substituted olefins proved effective. The stereochemical discrimination capability of system enabled precise stereoselective transformations of challenging nonactivated alkenes. Cyclohexyl (**100**), benzyl (**101** and **102**), and -CH_2_-TMS (**103**) olefins were proven to be valuable substrate too. Alkenes containing functional groups such as secondary alkyl (**104** and **105**), and aryl (**106** to **108**) groups at the distal site can also obtain target products with excellent stereoselectivity. Achieving asymmetric transformation of linear alkenes without branched or other functional groups is undoubtedly the most challenging task due to the absence of effective NCI to induce stereoselectivity. To our delight, our reaction system can be used for the difluoromethylation/sulfonylation of chain alkanes including light alkene, delivering **109** to **113** in 50 to 87% yields and excellent stereoselectivity (>20:1 dr). The stereochemistry of the transformation was confirmed by x-ray analysis of the product **113** (CCDC 2411985). Gaseous chemical feedstock propylene can be transformed into the desired product, although there is a reduction in diastereomeric selectivity (18:1 dr). We further investigated the applicability of chiral polyfluorobenzates.

Chiral polyfluorobenzates derived from (*R*)-1-phenylethan-1-ol (**115** to **117**), (*R*)-4-phenylbutan-2-ol (**118**), and natural products and bioactive molecules, such as borneol (**119** and **120**), diacetonefructose (**121**), and pantolactone (**122**), demonstrated the ability to deliver the desired chiral polyfluoroaryl sulfone with yields ranging from 51 to 80% and excellent stereoselectivities (with a dr of 15:1 to 18:1 for propylene and greater than 20:1 for styrene). In addition, the trifluoromethylative sulfonation of alkenes was achieved with high stereoselectivity, as evidenced by the production of compounds **123** (74% yield, 11:1 dr) and **124** (85% yield, >20:1 dr).

### Mechanistic investigations

**Fig. 5.**
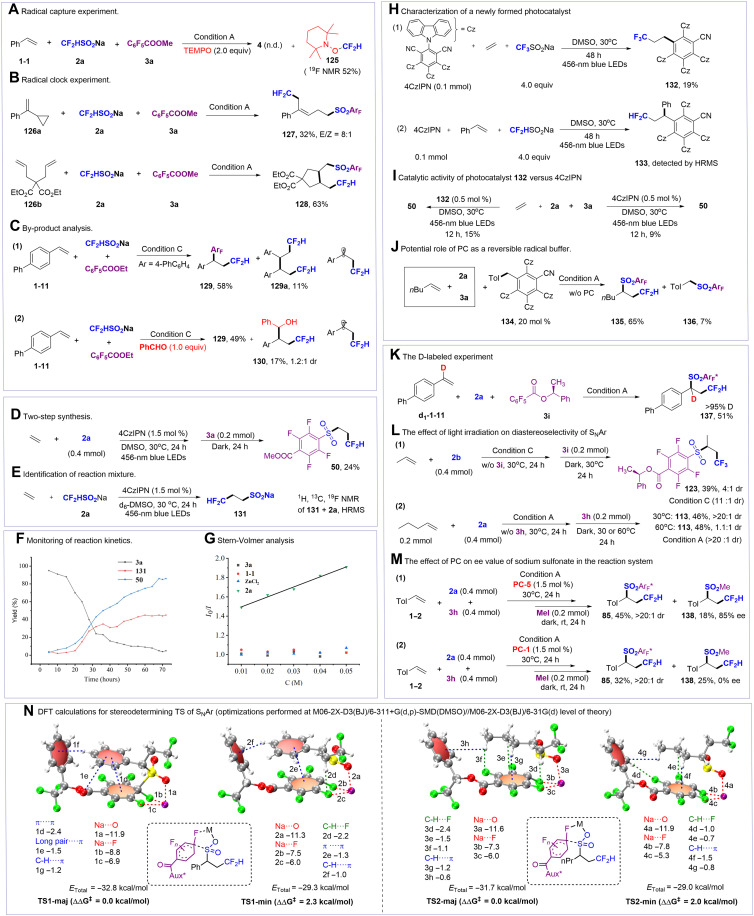
Mechanistic investigations. (**A**) Radical capture experiment. n.d., not determined. (**B**) Radical clock experiment. (**C**) By-product analysis. (**D**) Two-step synthesis. (**E**) Identification of reaction mixture. (**F**) Monitoring of reaction kinetics. (**G**) Stern-Volmer analysis. (**H**) Characterization of a newly formed photocatalyst. (**I**) Catalytic activity of photocatalyst **123** versus 4CzIPN. (**J**) Potential role of PC as a reversible radical buffer. (**K**) The D-labeled experiment. (**L**) The effect of light irradiation on diastereoselectivity of S_N_Ar. (**M**) The effect of PC on ee value of sodium sulfonate in the reaction system. (**N**) DFT calculations for stereodetermining TS of S_N_Ar (optimizations performed at M06-2X-D3(BJ)/6-311+ G(d,p)-SMD(DMSO)//M06-2X-D3(BJ)/6-31G(d) level of theory).

We conducted a series of investigations to elucidate the reaction mechanism, and the results were summarized in Fig. 5. A radical trapping experiment was conducted, and the addition of 2,2,6,6-Tetramethylpiperidine-1-oxyl (TEMPO) completely inhibited the transformation, resulting in the formation of **125** in a 52% nuclear magnetic resonance (NMR) yield (Fig. 5A). The radical clock experiment further supported the involvement of radical species, evidenced by the formation of ring-opening product **127** and ring-closing product **128** (Fig. 5B). In the difluoromethylation/polyfluoroarylation of **1** to **11**, we separated by-products **129a** (11%) along with **129** (Fig. 5C1). The formation of dimer **129a** provides direct evidence for the presence of a benzyl radical intermediate in this transformation. Notably, the introduction of benzaldehyde under these conditions affords hydroxyalkylation product **130** (17% yield, 1.2:1 dr), demonstrating the potential participation of alkyl anion species (Fig. 5C2). We further evaluated the reactivity of styrene derivatives bearing diverse functional groups (fig. S13) under condition A (with **PC-1**) and condition B (with **PC-6**). The results reveal that the **PC-1** system preferentially affords SO_2_-reintegration products, while the **PC-6** system favors the formation of SO_2_-release products. Notably, across both photocatalytic systems, electron-deficient styrene derivatives consistently displayed a stronger tendency to undergo SO_2_ release compared to their electron-rich counterparts. This divergence can be rationalized by the higher oxidative potential of the benzyl radicals derived from electron-deficient styrenes, which promotes their reduction by the photoreduced sensitizer, thereby leading to the formation of a key carbanion intermediate and thus steering the reaction pathway toward SO_2_-release products. Two-step experiments were conducted with ethylene, and **50** was observed with 24% yield (Fig. 5D). Identification of the reaction of CF_2_HSO_2_Na and ethylene in d_6_-DMSO provides more detailed information of intermediate **131** (Fig. 5E), which is considered as alkyl sulfinate [NMR and high-resolution mass spectrum (HRMS) analysis of mixture]. This conclusion corresponds with the findings from monitoring the reaction system (Fig. 5F), suggesting the potential for a radical-polar crossover (*71*, *72*) in the delivery of polyfluoroaryl sulfone. Stern-Volmer quenching experiments (Fig. 5G and fig. S7) revealed that the excited state PC-1* and PC-6* could be quenched by CF_2_HSO_2_Na (**2a**) instead of olefin (**1-1**) or pentafluorobenzoate (**3a**). These findings, in combination with the reduction potential of PC-1* [*E*_1/2_ = 1.35 V versus saturated calomel electrode (SCE)] (*75*), PC-6* (*E*_1/2_ = 0.98 V versus SCE) (*75*), and the oxidation potential of CF_2_HSO_2_Na (*E*_Ox_ = 0.59 V versus SCE) (*76*), as well as Fig. 3E, suggest the reductive quenching pathway. We found significant rate acceleration after 20 hours looks almost similar to a sigmoidal kinetics. Stoichiometric experiments successfully isolated alkylated PC **132**, demonstrating radical trapping capability of 4CzIPN (Fig. 5H1) (*77*, *78*). HRMS characterization further revealed alkylated photosensitizer **133** in the styrene-mediated reaction system (Fig. 5H2). Control experiments established the superior catalytic efficacy of compound **132** versus conventional 4CzIPN under standardized conditions (Fig. 5I), which consistent with observation in reaction kinetics. Through strategic introduction of benzyl-modified 4CzIPN **134** [pioneering work from König and colleagues (*77*, *78*)] into reaction system, we identified polyfluoroaryl sulfone **136** as competitive product (Fig. 5J). This critical observation indicates the reversibility of alkyl radical release/recapture dynamics. Combined with Fig. 3H, 4CzIPN served as alkyl radical buffers and stabilizers, extending its lifetime for SO_2_ trapping. The role of ZnCl_2_ was investigated, and control experiments (fig. S14) reveal that it enhances reaction efficiency by forming the more reducible zinc difluoromethanesulfinate in situ via metal exchange, which subsequently facilitates the generation of difluoromethyl radicals. We further investigate the factors affecting the stereoselectivity of the stereoselectivity of the SO_2_ reintegration system. When **d**_**1**_**-1-11** was used as a starting material, the deuterium atom in **137** is completely retained. This result indicates that the C─H bond at the α-position of the sulfonyl group may not cleave throughout the reaction (Fig. 5K). A one-pot, two-step method was carried out, and a significant decrease in dr value was observed for compound **123** compared to the condition C (Fig. 5L1). For compound **113**, the dr value remained constant at 30°C; only when the temperature was raised to 60°C did the dr value decrease to approximately 1.1:1 (Fig. 5 L2). This finding was further supported by intermediate capture experiments, where the reaction was quenched after 24 hours using MeI as an electrophile (Fig. 5M). The PC-switching experiment provides key evidence for the accumulation of the sulfinate intermediate and its PC-induced racemization: **PC-5** afforded product **138** with high enantiocontrol [85% enantiomeric excess (ee), 18% yield; Fig 5M1], whereas **PC-1** led to complete racemization (racemic **138**; 25% yield, 0% ee; Fig 5M2), consistent with rapid sulfinate racemization induced by **PC-1** that enables dynamic kinetic resolution during the subsequent nucleophilic aromatic substitution (see Supplementary Materials and fig. S12).

### Density functional theory calculations

To gain insights into the origin of the remote stereoselective induction in the cascade reaction, we performed density functional theory (DFT) calculations using styrene derivatives and chained alkenes as benchmark substrates at M06-2X-D3(BJ)/6-311+ G(d,p)-SMD(DMSO)//M06-2X-D3(BJ)/6-31G(d) level of theory. In **TS1-Major**, π···π, C − H···π, and lone pair···π interactions were identified between the phenyl rings of olefins and polyfluoroaromatic hydrocarbons moiety, thereby promoting the distant stereoselective induction. Simultaneously, Na^+^ activates both reactants through Na···O and Na···F interactions. The calculated relative free energies demonstrate that the major transition states via **TS1-Major** is 2.3 kcal mol^−1^ more favorable than **TS1-Minor**, giving an ee of 96% in agreement with experimental observations. Similarly, for chained alkenes (**92**), the transition states are stabilized by C − H···F and C − H···π interactions between the -CH_2_/CH_3_ group and the polyfluoroaromatic hydrocarbon moiety, providing a remote stereoselective induction environment. The enantioselectivity transition state **TS2-Major** is energetically favored over **TS2-Minor** by 2.0 kcal/mol (corresponding to 93% ee). Quantitative analysis of NCIs through bond critical point electron densities reveals stronger interactions in **TS1-Major**/**TS2-Major** compared to **TS1-Minor**/**TS2-Minor**. The observed stereoselectivity originates from differential NCI strengths (including π···π, lone pair···π, C − H···F, C − H···π, and Na···O/F interactions) between the enantiodetermining transition states, with energy differences of 2.3 kcal/mol for styrene substrates and 2.0 kcal/mol for chain olefin substrates.

### Possible reaction pathway

**Fig. 6.**
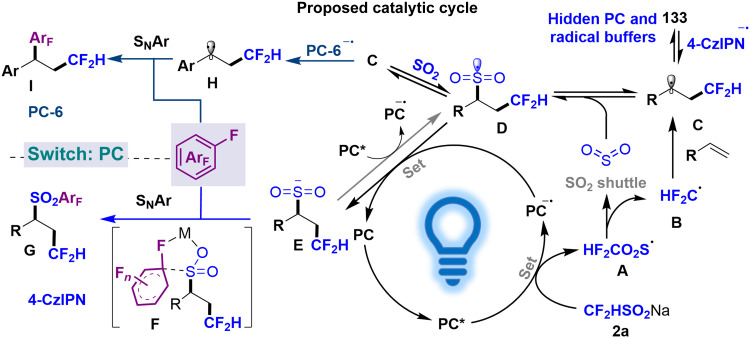
Proposed mechanism.

On the basis of the observations mentioned above and previous reports, a possible reaction mechanism for the organo-catalyzed difluoromethylation/switchable SO_2_ reintegration/polyfluoroarylation of olefin can be illustrated in Fig. 6. Photoexcitation of the catalyst (PC*) under LED irradiation initiates reductive quenching with CF_2_HSO_2_Na, generating difluoromethanesulfonyl radical **A**. Subsequent thermally driven SO_2_ extrusion from **A** produces HCF_2_ radical **B**, which undergoes regioselective addition to the alkene to form alkyl radical **C**. The liberated SO_2_ participates in trapping **C**, yielding alkylsulfonyl radical **D**. This intermediate undergoes sequential single-electron reduction by the reduced photocatalyst (**PC**^**•−**^) to form sulfinic anion **E**, while photooxidative regeneration of **D** from **E** by PC* establishes a dynamic equilibrium among intermediates **B**, **C**, and **D**.

The reaction pathway bifurcation arises from photocatalyst selection. For **PC-1**, the limited reducing capacity of **PC**^**•−**^ (*E*_1/2_ = − 1.21 V versus SCE) and reversible alkyl radical addition to 4CzIPN extend the radical lifetime to enable SO_2_ trapping, thereby preventing reduction to anions and suppressing competitive coupling with polyfluoroarenes. Nucleophilic aromatic substitution (S_N_Ar) between intermediate **E** and polyfluoroarenes proceeds through a five-membered cyclic transition state, which drives the equilibrium toward **E** via Le Chatelier’s principle to form SO_2_-reintegration product **G**. Conversely, **PC-6**^•−^ (*E*_1/2_ = −1.42 V versus SCE) promotes single-electron reduction of benzyl radicals **C** (for PhCH_2_•, *E*_1/2_ = −1.43 V versus SCE) to generate benzyl anions **H** (*79*), enabling S_N_Ar pathways with polyfluoroarenes to produce SO_2_-evolution product **I**. This conclusion is consistent with the experimental observation (fig. S13): Electron-deficient (strongly oxidizing) benzyl radicals exhibit a pronounced tendency to form the SO_2_-evolution product, in contrast to electron-rich radicals. Dynamic kinetic resolution occurs when chiral polyfluoroarenes induce differential reaction rates between enantiomeric sulfinate anions, with rapid interconversion through the alkyl radical–sulfonyl radical–sulfonyl anion equilibrium, enabling Curtin-Hammett dynamic stereochemical control. The operational key involves establishing a pendulum-swung dynamic intermediate reservoir while achieving precise chemo- and stereochemical partitioning during the product-determining step.

## DISCUSSION

In summary, we developed photoredox-controlled chemo-, regio-, and stereoselective radical difluoromethylation/switchable SO_2_ reintegration/polyfluoroarylation of alkenes based on precise sorting of intermediates within an equilibrium system containing nearly a dozen of highly active radical/anion intermediates. This approach provides a robust and practical methodology for installing two distinct fluorine-containing groups into double bonds with chemodivergence and stereoselectivity. The photocatalyst can function as a switch, while polyfluoroarenes serve as electrophile reagents and CF_2_HSO_2_Na serves as bifunctional reagents, which allows controllable radical conversion under mild conditions via radical-polar crossover. A wide range of olefins, including styrenes, nonactivated alkenes, and light alkenes such as ethylene and propylene, have been successfully used in SO_2_ reintegration systems. Notably, chiral alcohol–derived polyfluoroarenes efficiently induce stereoselectivity for coupling of alkyl radical–SO_2_, operating over nine atoms. Styrene derivatives and nonactivated alkenes, including propylene, which has formidable challenge in stereoselective transformations, exhibit satisfied stereoselectivity. DFT calculations were carried out to provide insights into the ultralong-distance stereochemical induction mechanisms. For aliphatic alkanes, the NCI between alkyl groups and polyfluorocarbons modulates the stereoselectivity of the transformation, which could provide insights into designing asymmetric transformations involving nonactivated alkenes. Furthermore, our methodology establishes the first enantioselective C─S bond formation between alkyl radicals and SO_2_ through precise sorting of the enantiomeric intermediates from the dynamic equilibrium pool, which may also be applicable to other conceptually innovative asymmetry transformations.

## MATERIALS AND METHODS

### General experimental procedures

All reactions were carried out under nitrogen atmosphere. Reagents were purchased from commercial sources and used without further purification, unless otherwise noted. All of the solvents were anhydrous according to distillation. The reactions were monitored with the aid of thin-layer chromatography (TLC) on 0.25-mm precoated silica gel plates. Melting points were measured on Büchi B-540 apparatus. ^1^H NMR spectra were recorded at 25°C on a Bruker 600 or 500, Varian 500 MHz, and ^13^C NMR spectra were recorded at 25°C on a Bruker 151, Varian 126 MHz, respectively, in CDCl_3_ by using TMS as internal standard. ^19^F NMR spectra were recorded at 25°C on a Bruker 565 MHz. ^1^H and ^13^C NMR spectra are reported in parts per million (ppm) downfield from an internal standard, tetramethylsilane (0 ppm for ^1^H NMR), and CHCl_3_ (77.0 ppm for ^13^C NMR), respectively. Letters m, s, d, t, and q stand for multiplet, singlet, doublet, triplet, and quartet, respectively. HRMS were recorded on Bruker micrOTOF. We use ACPR-50-2 type 7 + 1 position optical reaction system produced by Henan Provincial Coal Science and Technology Research Institute’s New Materials Technology Co. Ltd. located in Zhengzhou, China. The optical reactor we use is equipped with adjustable LED lights ranging from 0 to 50 W. The peak wavelength of these LED lights is 456 nm. The containers for irradiation are borosilicate glass test tubes, and the LED lights directly illuminate the tubes with a light path of 1.5 cm. There are no filters between the LED lights and the test tubes.

### General procedure

Condition A: Taking **4** as an example. In a nitrogen-filled glovebox, a flame-dried screw-cap reaction tube equipped with a Teflon-coated magnetic stir bar was charged with 4CzIPN (2.4 mg, 1.5 mol %), ZnCl_2_ (27.2 mg, 1.0 equiv), K_3_PO_4_ (42.5 mg, 1.0 equiv), and dry DMSO (2.0 ml). Then styrene **1-1** (21.3 mg, 0.2 mmol), NaSO_2_CF_2_H **2a** (56.0 mg, 2.0 equiv), and methyl pentafluorobenzoate **3a** (90.4 mg, 2.0 equiv) were added. The reaction mixture was irradiated with 456-nm blue LEDs at room temperature for 48 hours, until the reaction was complete as indicated by TLC. After the reaction, ethyl acetate and water were poured into the mixture. The organic layer was washed with brine, dried over Na_2_SO_4_, and filtered. In addition, the reaction mixture was concentrated in vacuo. The resulting crude product was purified by flash column chromatography on silica gel (petroleum ether:ether acetate = 8:1) to obtain product **4**.

Condition B: Taking **5** as an example. In a nitrogen-filled glovebox, a flame-dried screw-cap reaction tube equipped with a Teflon-coated magnetic stir bar was charged with **PC-6** (3.0 mg, 1.5 mol %), ZnCl_2_ (27.2 mg, 1.0 equiv), K_3_PO_4_ (42.5 mg, 1.0 equiv), and dry DMSO (2.0 ml). Then, styrene **1-1** (21.3 mg, 0.2 mmol), NaSO_2_CF_2_H **2a** (56.0 mg, 2.0 equiv), and methyl pentafluorobenzoate **3a** (90.4 mg, 2.0 equiv) were added. The reaction mixture was irradiated with 456-nm blue LEDs at 30°C for 12 hours, until the reaction was complete as indicated by TLC. After the reaction, ethyl acetate and water were poured into the mixture. The organic layer was washed with brine, dried over Na_2_SO_4_, and filtered. In addition, the reaction mixture was concentrated in vacuo. The resulting crude product was purified by flash column chromatography on silica gel (petroleum ether:ether acetate = 18:1) to obtain product **5**.

Condition C: Taking **50** as an example. In a schlenk tube, the tube was filled with 4CzIPN (2.4 mg, 1.5 mol %) and dry DMSO (2.0 ml). Then, NaSO_2_CF_2_H **2a** (56.0 mg, 2.0 equiv) and methyl pentafluorobenzoate **3a** (45.2 mg, 0.2 mmol) were added. Then, the nitrogen in the reaction device is pumped away and filled with ethylene gas. The reaction mixture was irradiated with 456-nm blue LEDs at 30°C for 72 hours until the TLC showed that the reaction was complete. The organic layer was washed with brine, dried over Na_2_SO_4_, and filtered. In addition, the reaction mixture was concentrated in vacuo. The resulting crude product was purified by flash column chromatography on silica gel (petroleum ether:ether acetate = 10:1) to obtain product **50**.

## Supplementary Materials

### This PDF file includes:

Supplementary Text Tables S1 to S7 Figs. S1 to S17 NMR Spectra References
