# Supplementary material for: Photoredox-controlled chemo-divergent, regio-, and stereoselective difunctionalization of olefins via switchable SO2 reintegration
Source: Sci Adv. 2025 Dec 19;11(51):eaeb0206. doi: 10.1126/sciadv.aeb0206 (PMC12716424; doi:10.1126/sciadv.aeb0206)
Supplement: Supplementary file 1 — Supplementary Text Tables S1 to S7 Figs. S1 to S17 NMR Spectra References [file sciadv.aeb0206_sm.pdf]

Science Advances
AAAS

Supplementary Materials for

Photoredox-controlled chemo-divergent, regio-, and stereoselective difunctionalization of olefins via switchable SO_2 reintegration

Jiuli Xia *et al.*

Corresponding author: Guangfan Zheng, zhenggf265@nenu.edu.cn; Qian Zhang, zhangq651@nenu.edu.cn

Sci. Adv. **11**, eaeb0206 (2025)
DOI: 10.1126/sciadv.aeb0206

This PDF file includes:

Supplementary Text
Tables S1 to S7
Figs. S1 to S17
NMR Spectra
References

General information

All reactions were carried out under nitrogen atmosphere. Reagents were purchased from commercial sources and used without further purification, unless otherwise noted. All of the solvents were anhydrous according distillation. The reactions were monitored with the aid of thin-layer chromatography (TLC) on 0.25 mm precoated silica gel plates. Melting points were measured on Büchi B-540 apparatus. ^{1}H NMR spectra were recorded at 25 °C on a Bruker 600 or 500, Varian 500 MHz, ^{13}C NMR spectra were recorded at 25 °C on a Bruker 151, Varian 126 MHz, respectively in $CDCl_3$ by using TMS as internal standard. ^{19}F NMR spectra were recorded at 25 °C on a Bruker 565 MHz. ^{1}H and ^{13}C NMR spectra are reported in parts per million (ppm) downfield from an internal standard, tetramethylsilane (0 ppm for ^{1}H NMR) and $CHCl_3$ (77.0 ppm for ^{13}C NMR), respectively. Letters m, s, d, t, and q stand for multiplet, singlet, doublet, triplet, and quartet, respectively. High-resolution mass spectra (HRMS) were recorded on Bruck microtof. We use ACPR-50-2 type 7+1 position optical reaction system produced by Henan Provincial Coal Science and Technology Research Institute's New Materials Technology Co., Ltd. located in Zhengzhou, China. The optical reactor we use is equipped with adjustable LED lights ranging from 0 to 50 W. The peak wavelength of these LED lights is 456 nm. The containers for irradiation are borosilicate glass test tubes, and the LED lights directly illuminate the tubes with a light path of 1.5 cm. There are no filters between the LED lights and the test tubes. The device diagram is as follows:

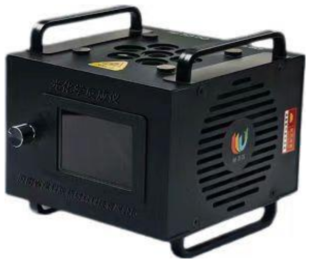

The optimization of the reaction[a]

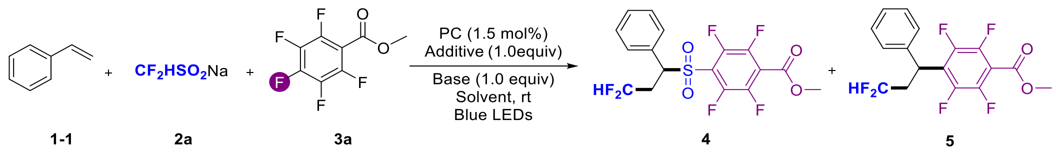

Entry	PC	Additive	Solvent	Base	Yields/4	Yields/5
**1**	**PC-1**	**$ZnCl_2$**	**DMSO**	**K_3PO_4**	**86%**	trace
2[b]	PC-1	$ZnCl_2$	DMSO	K_3PO_4	48%	0%
3	PC-2	$ZnCl_2$	DMSO	K_3PO_4	35%	0%
4	PC-3	$ZnCl_2$	DMSO	K_3PO_4	75%	7%
5	PC-4	$ZnCl_2$	DMSO	K_3PO_4	51%	4%
6	PC-5	$ZnCl_2$	DMSO	K_3PO_4	46%	9%
7	PC-6	$ZnCl_2$	DMSO	K_3PO_4	9%	68%
8	PC-1	------	DMSO	K_3PO_4	58%	trace
9	PC-1	$ZnCl_2$	THF	K_3PO_4	10%	0%
10	PC-1	$ZnCl_2$	DCM	K_3PO_4	0%	0%
11	PC-1	$ZnCl_2$	MeCN	K_3PO_4	0%	trace
12	PC-1	$ZnCl_2$	DMF	K_3PO_4	Trace	0%
13	PC-1	$ZnCl_2$	DMAc	K_3PO_4	70%	trace
14	PC-1	$ZnCl_2$	DMSO	------	45%	0%
15	PC-1	$ZnCl_2$	DMSO	Na_2CO_3	59%	10%
16	PC-1	$ZnCl_2$	DMSO	K_2CO_3	53%	7%
17[c]	PC-1	$ZnCl_2$	DMSO	K_3PO_4	20%	0
18	------	$ZnCl_2$	DMSO	K_3PO_4	0%	0
19[d]	PC-1	$ZnCl_2$	DMSO	K_3PO_4	0%	0

PC-1: 4CzIPN	PC-4: Eosin Y
PC-2: 4BrCzIPN	PC-5: $[Ru(bpy)_3]PF_6$
PC-3: $[Ir(dF(CF_3)ppy)_2(dtbbpy)]PF_6$	PC-6: $[Ir(dFppy)_2(dtbbpy)]PF_6$

Table **S1**. Optimization of the reaction conditions [a]

[a] Unless otherwise noted, all the reactions were carried out with **1-1** (0.2 mmol, 1.0 equiv), **2a** (0.4 mmol, 2.0 equiv), **3a** (0.4 mmol, 2.0 equiv), base (0.2 mmol, 1.0 equiv), PC (1.5 mol%), $ZnCl_2$ (0.2 mmol, 1.0 equiv) in dry solvent (2 mL), irradiation with blue LEDs at room temperature for 48 h. [b] Reaction for 24 h. [c] Reaction was carried out under air. [d] Reaction was carried out in dark.

General procedure

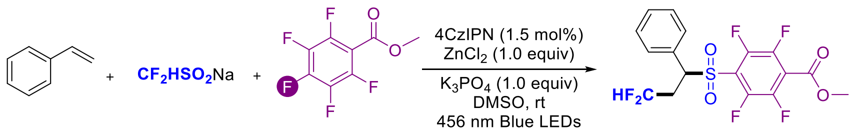

1-1 2a 3a 4

Conditions A: Taking **4** as an example. In a nitrogen-filled glovebox, a flame-dried screw-cap reaction tube equipped with a Teflon-coated magnetic stir bar were charged with 4CzIPN (2.4 mg, 1.5 mol%), $ZnCl_2$ (27.2 mg, 1.0 equiv), K_3PO_4 (42.5 mg, 1.0 equiv), and dry DMSO (2.0 mL). Then styrene **1-1** (21.3 mg, 0.2 mmol), $NaSO_2CF_2H$ **2a** (56.0 mg, 2.0 equiv) and methyl pentafluorobenzoate **3a** (90.4 mg, 2.0 equiv) were added. The reaction mixture was irradiated with 456 nm Blue LEDs at room tempurature for 48 hours, until the reaction was complete as indicated by TLC. After the reaction, ethyl acetate and water were poured into the mixture. The organic layer was washed with brine, dried over Na_2SO_4 and filtered. And the reaction mixture was concentrated in vacuo. The resulting crude product was purified by flash column chromatography on silica gel (petroleum ether: ether acetate = 8:1) to obtain product **4**. The details and characterization datas of the products were stated below.

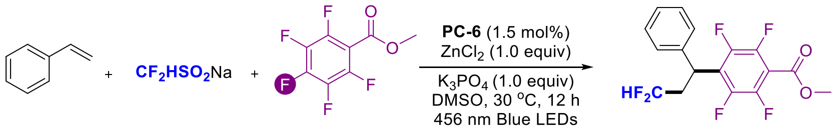

1-1 2a 3a 5

Conditions B: Taking **5** as an example. In a nitrogen-filled glovebox, a flame-dried screw-cap reaction tube equipped with a Teflon-coated magnetic stir bar were charged with **PC-6** (3.0 mg, 1.5 mol%), $ZnCl_2$ (27.2 mg, 1.0 equiv), K_3PO_4 (42.5 mg, 1.0 equiv), and dry DMSO (2.0 mL). Then styrene **1-1** (21.3 mg, 0.2 mmol), $NaSO_2CF_2H$ **2a** (56.0 mg, 2.0 equiv) and methyl pentafluorobenzoate **3a** (90.4 mg, 2.0 equiv) were added. The reaction mixture was irradiated with 456 nm Blue LEDs at 30 °C for 12 hours, until the reaction was complete as indicated by TLC. After the reaction, ethyl acetate and water were poured into the mixture. The organic layer was washed with brine, dried over Na_2SO_4 and filtered. And the reaction mixture was concentrated in vacuo. The resulting crude product was purified by flash column chromatography on silica gel (petroleum ether: ether acetate = 18:1) to obtain product **5**. The details and characterization datas of the products were stated below.

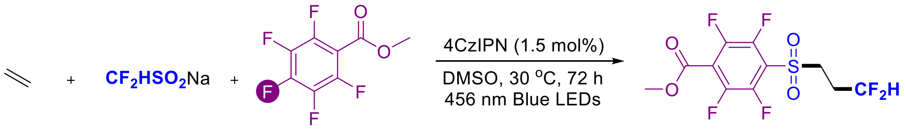

2a 3a 50

Conditions C: Taking **50** as an example. In a schlenk tube, the tube was filled with 4CzIPN (2.4 mg, 1.5 mol%), dry DMSO (2.0 mL). Then $NaSO_2CF_2H$ **2a** (56.0 mg, 2.0 equiv) and methyl pentafluorobenzoate **3a** (45.2 mg, 0.2 mmol) were added. Then the nitrogen in the reaction device is pumped away and filled with ethylene gas. The reaction mixture was irradiated with 456 nm Blue LEDs at 30 °C for 72 hours until the TLC showed that the reaction was complete. The organic layer was washed with brine, dried over Na_2SO_4 and filtered. And the reaction mixture was concentrated in vacuo.

The resulting crude product was purified by flash column chromatography on silica gel (petroleum ether: ether acetate = 10:1) to obtain product **50**. The details and characterization datas of the products were stated below.

Preparation of the starting materials

Preparation of olefin derivatives 1:

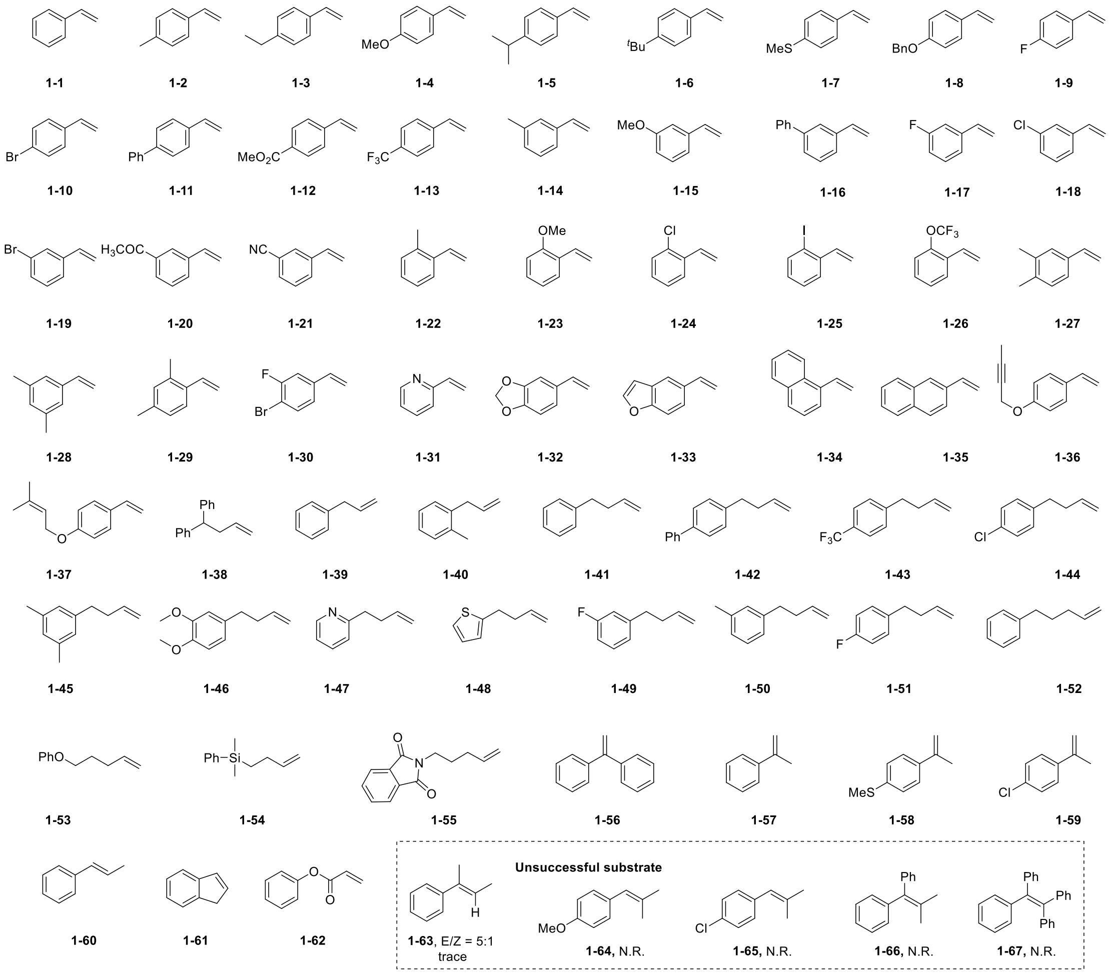

Table **S2** Substrate of olefin derivatives

Method A:

General Procedure for Preparation of Styrene **1-1 - 1-35**, **1-56-1-59**.

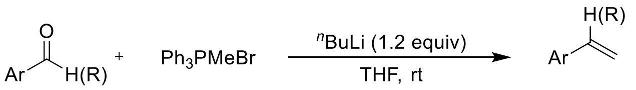

A 100.0 mL roundbottomed flask equipped with a magnetic stir bar was charged with Ph_3PMeBr (6 mmol, 1.2 equiv, 2.142 g) and 20.0 mL THF. The solution was cooled to 0 ºC, and ^{n}BuLi (2.5 M in THF, 2.4 mL, 6 mmol, 1.2 equiv) was added. The resulting solution was stirred for 20 minutes at room temperature. Aromatic aldehyde (5.0 mmol, 1.0 equiv) was added dropwise. The reaction mixture was monitored by TLC for completion. On completion, the reaction was quenched with saturated aqueous NH_4Cl (30.0 mL). The aqueous layer was extracted with ether, and the combined organic layers were washed with brine (30.0 mL), dried over Na_2SO_4, filtered, and the combined

organic solutions concentrated under vacuum. Then the mixture was purified by flash column chromatography to yield the styrene derivatives.[80]

Method B:

Procedure for Preparation of Styrene **1-36**.

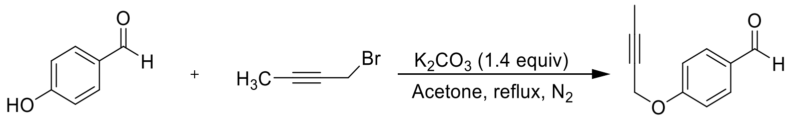

A 100.0 mL round-bottomed flask equipped with a magnetic stir bar was charged with aldehyde (10 mmol), K_2CO_3 (14 mmol, 1.4 equiv) and 30.0 mL of acetone. The resulting solution was stirred for 30 minutes at 60 °C under nitrogen atmosphere. 1-Bromobut-2-yne (20.0 mmol, 2.0 equiv) was added dropwise at room temperature. Then the resulting solution was stirred for 2 hours at 60 °C. On completion, the reaction was extracted with EtOAc, and the combined organic layers were washed with NaOH solution (30.0 mL), dried over Na_2SO_4, filtered, and the combined organic solutions concentrated under vacuum. Then the mixture was purified by flash column chromatography to yield the aldehyde derivatives[81]. The product was next used for **Method A**.

Method C:

Procedure for Preparation of Styrene **1-37**.

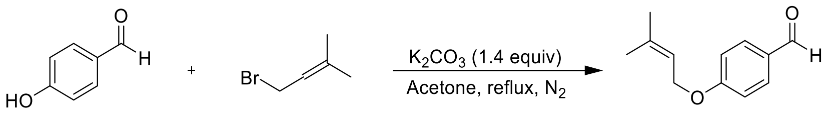

A 100.0 mL round-bottomed flask equipped with a magnetic stir bar was charged with aldehyde (10 mmol), K_2CO_3 (14 mmol, 1.4 equiv) and 20.0 mL of acetone. The resulting solution was stirred for 30 minutes at 60 °C under nitrogen atmosphere. 1-bromo-3-methylbut-2-ene (20.0 mmol, 2 equiv) was added dropwise at room temperature. Then the resulting solution was stirred for 2 hours at 60 °C. On completion, the reaction was extracted with EtOAc, and the combined organic layers were washed with NaOH solution (30.0 mL), dried over Na_2SO_4, filtered, and the combined organic solutions concentrated under vacuum. Then the mixture was purified by flash column chromatography to yield the aldehyde derivatives [81]. The product was next used for **Method A**.

Method D:

Procedure for Preparation of Styrene **1-38 - 1-46, 1-49 - 1-52**.

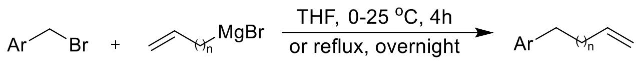

The Benzyl bromide (5.0 mmol) and 15.0 mL anhydrous THF were added to a flame dried 100 mL round-bottom flask. Alkylmagnesium bromide (7.5 mmol, 1 M in diethylether) was added dropwise at 0 °C. The reaction was stirred for 4 h at room temperature or reflux for 12 hours under N_2. After consumption of all starting material (as monitored by TLC), then quenched with saturated aqueous NH_4Cl. The aqueous layer was extracted with DCM (3 x 20.0 mL) and the combined organic layers were dried over Na_2SO_4, filtered off and concentrated under reduced pressure. The crude product was purified by flash chromatography (eluent: petroleum ether), affording the desired product. [81]

Method E:

Procedure for Preparation of Styrene **1-47, 1-48**.

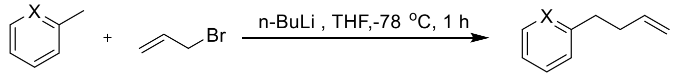

A solution of ^{n}BuLi (1.05 equiv) was added dropwise to a solution of 2-methylpyridine (10.0 mmol) in THF (30.0 mL) at −78 °C under nitrogen atmosphere and the reaction mixture was allowed to stir at this temperature for 15 min. The solution was then brought to 0 °C for 15 min, after which allyl bromide (1.05 equiv) was added at −78 °C. The resultant mixture was stirred for 30 min at –78 °C, after the reaction finished that detected by TLC, quenched with MeOH (2.0 mL) and stirred for 30 min, brought to room temperature, and diluted with H_2O (50.0 mL) and ethyl acetate (50.0 mL). The reaction mixture was extracted with ethyl acetate, and combined organics were washed with brine, dried over anhydrous Na_2SO_4, filtered off and concentrated under reduced pressure, the residue was purified by column chromatography on silica gel (petroleum ether / ethyl acetate 50: 1) as eluent to afford the product. [81]

Method F:

Procedure for Preparation of Styrene **1-53**.

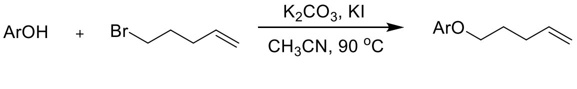

To a solution of aromatic alcohol (5.3 mmol, 1.0 equiv), K_2CO_3 (7.95 mmol, 1.5 equiv) and KI (10.6 mmol, 2.0 equiv) in CH_3CN (10.0 mL) was added 5-bromopent-1-ene (10.6 mmol, 2.0 equiv), and the mixture was refluxed for 12 hours. After the filtration, the combined organic phase was dried over anhydrous Na_2SO_4 and concentrated in vacuo. The resulting residue was purified by silica gel flash chromatography to

provide the corresponding alkene. [81]

Method G:

Procedure for Preparation of Styrene **1-54.**

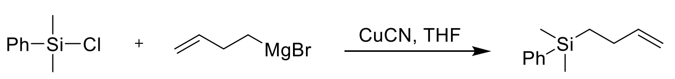

A 100.0 mL roundbottomed flask equipped with a magnetic stir bar was charged with CuCN (5 mol%, 0.045 g) and 20.0 mL of THF. The solution was cooled to 0 ºC, 3-Butenylmagnesium bromide solution (12 mmol, 1.2 equiv) and Phenyl dimethyl chlorosilane (10 mmol, 1.0 equiv) was added. The resulting solution was stirred for 24 hours at room temperature. The reaction mixture was monitored by TLC for completion. On completion, the reaction was quenched with saturated aqueous NH_4Cl (30.0 mL). The aqueous layer was extracted with ether, and the combined organic layers were washed with brine (30.0 mL), dried over Na_2SO_4, filtered, and the combined organic solutions concentrated under vacuum. Then the mixture was purified by flash column chromatography to yield the styrene derivetives. [81,82]

All other compounds, except the aforementioned substrates, are commercially available.

Preparation of polyfluoroaromatics 3:

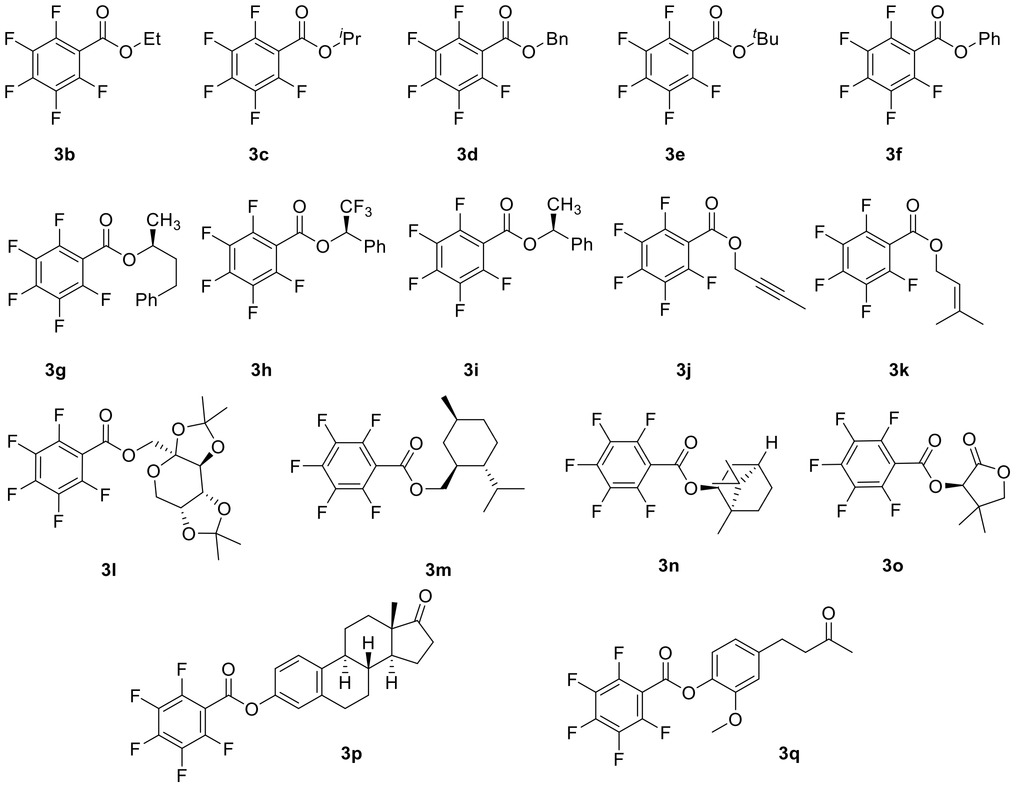

Table **S3** Substrate of polyfluoroaromatics

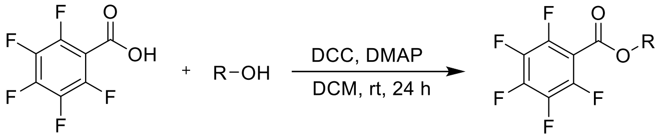

To a solution of pentafluorobenzoic acid (1.06 g, 5.0 mmol) in DCM (15.0 mL) was added DCC (1.24 g, 6.0 mmol), DMAP (61.0 mg, 0.5 mmol), and alcohol (6.0 mmol). The mixture was stirred for 24 hours at room temperature. After filtration, the mixture was concentrated under reduced pressure. The residue was purified by flash column chromatography on silica gel to afford the products as colorless oil. [83]

Preparation of 4CzIPN, 4BrCzIPN:

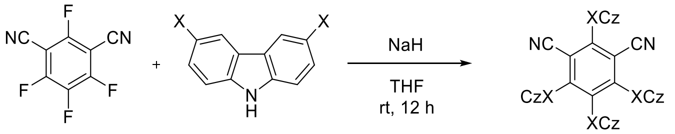

NaH (60% in oil, 0.60 g, 15 mmol) was added slowly to a stirred solution of corresponding carbazole (10.0 mmol) in dry THF (40 mL) under a nitrogen atmosphere at room temperature. After 0.5 h, tetrafluoroisophthalonitrile (0.40 g, 2.0 mmol), was added. After stirred at room temperature for 12 hours, 2mL water was added to the reaction mixture to quench the excess NaH. The resulting mixture was then concentrated under reduced pressure and washed by water and EtOH to yield the crude product, which was purified by recrystalization from hexane / DCM or acetone / $CHCl_3$ corresponding products. [84]

Gram-Scale synthesis

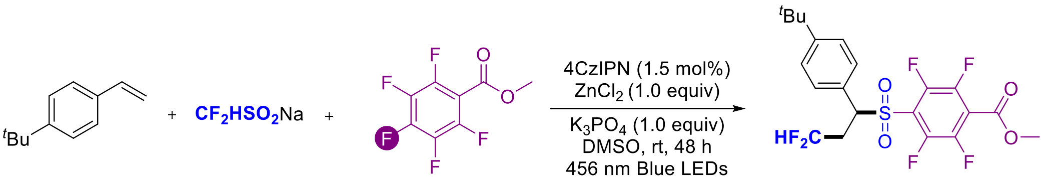

1-6 **2a** **3a** **8**

In a nitrogen-filled glovebox, a flame-dried screw-cap reaction tube equipped with a Teflon-coated magnetic stir bar were charged with 4CzIPN (24.0 mg, 1.5 mol%), $ZnCl_2$ (272.0 mg, 1.0 equiv), K_3PO_4 (425.0 mg, 1.0 equiv), and dry DMSO (20.0 mL). Then styrene **1-6** (320.5 mg, 2.0 mmol), $NaSO_2CF_2H$ **2a** (560.0 mg, 2.0 equiv) and methyl pentafluorobenzoate **3a** (904.0 mg, 2.0 equiv) were added. The reaction mixture was irradiated with 456 nm Blue LEDs at room temperature for 48 hours. After the reaction, ethyl acetate and water were poured into the mixture. The organic layer was washed with brine, dried over Na_2SO_4 and filtered. And the reaction mixture was concentrated in vacuo. The resulting crude product was purified by flash column chromatography on silica gel (petroleum ether: ether acetate = 10:1) to obtain product **8** as white solid (0.61 g, 75%).

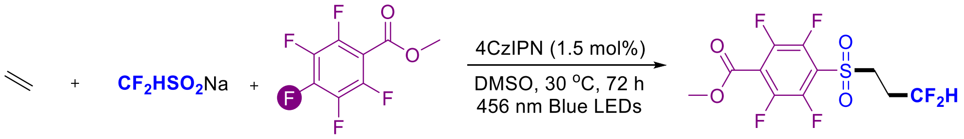

2a **3a** **50**

In a nitrogen-filled glovebox, a flame-dried screw-cap reaction tube equipped with a Teflon-coated magnetic stir bar were charged with 4CzIPN (60.0 mg, 1.5 mol%), dry DMSO (50.0 mL). Then methyl pentafluorobenzoate **3a** (1.13 g, 5.0 mmol), ethylene (1 atm) and $NaSO_2CF_2H$ **2a** (2.0 equiv) were added. The reaction mixture was irradiated with 456 nm Blue LEDs at 30 ºC for 72 hours. After the reaction, ethyl acetate and water were poured into the mixture. The organic layer was washed with brine, dried over Na_2SO_4 and filtered. And the reaction mixture was concentrated in vacuo. The resulting crude product was purified by flash column chromatography on silica gel (petroleum ether: ether acetate = 8:1) to obtain product **50** as colorless oil (1.42 g, 81%).

Mechanism studies

a) Control experiments

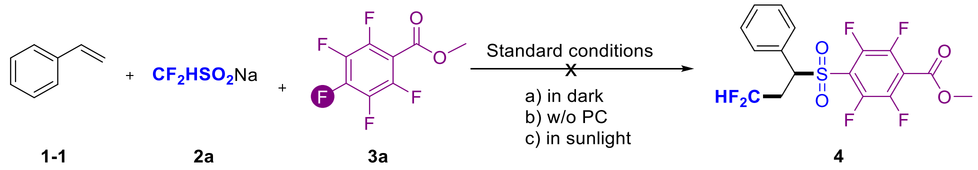

Into a nitrogen-filled glove box, a vial (10.0 mL) equipped with a magnetic stir bar was charged with 4CzIPN (2.4 mg, 1.5 mol%), $ZnCl_2$ (27.2 mg, 1.0 equiv), K_3PO_4 (42.5 mg, 1.0 equiv), and dry DMSO (2.0 mL). Then styrene **1-1** (21.3 mg, 0.2 mmol), $NaSO_2CF_2H$ **2a** (56.0 mg, 2.0 equiv) and methyl pentafluorobenzoate **3a** (90.4 mg, 2.0 equiv) were added. The vial was removed from the glovebox, and then the reaction mixture was stirred in dark at room temperature for 48 hours. After the reaction, ethyl acetate and water were poured into the mixture. The organic layer was washed with brine, dried over Na_2SO_4 and filtered, then the solvent was evaporated under vacuum. The residue was analyzed by **^{1}H NMR**, the product **4** was not detected.

Into a nitrogen-filled glove box, a vial (10.0 mL) equipped with a magnetic stir bar was charged with $ZnCl_2$ (27.2 mg, 1.0 equiv), K_3PO_4 (42.5 mg, 1.0 equiv), and dry DMSO (2.0 mL). Then styrene **1-1** (21.3 mg, 0.2 mmol), $NaSO_2CF_2H$ **2a** (56.0 mg, 2.0 equiv) and methyl pentafluorobenzoate **3a** (90.4 mg, 2.0 equiv) were added. The vial was removed from the glovebox, and then the reaction mixture was stirred with 456 nm Blue LEDs at room temperature for 48 hours. After the reaction, ethyl acetate and water were poured into the mixture. The organic layer was washed with brine, dried over Na_2SO_4 and filtered, then the solvent was evaporated under vacuum. The residue was analyzed by **^{1}H NMR**, the product **4** was not detected.

Into a nitrogen-filled glove box, a vial (10.0 mL) equipped with a magnetic stir bar was charged with 4CzIPN (2.4 mg, 1.5 mol%), $ZnCl_2$ (27.2 mg, 1.0 equiv), K_3PO_4 (42.5 mg, 1.0 equiv), and dry DMSO (2.0 mL). Then styrene **1-1** (21.3 mg, 0.2 mmol), $NaSO_2CF_2H$ **2a** (56.0 mg, 2.0 equiv) and methyl pentafluorobenzoate **3a** (90.4 mg, 2.0 equiv) were added. The vial was removed from the glovebox, and then the reaction mixture was stirred in the sunlight at room temperature for 48 hours. After the reaction, ethyl acetate and water were poured into the mixture. The organic layer was washed with brine, dried over Na_2SO_4 and filtered, then the solvent was evaporated under vacuum. The residue was analyzed by **^{1}H NMR**, the product **4** was not detected.

b) Radical capture experiment

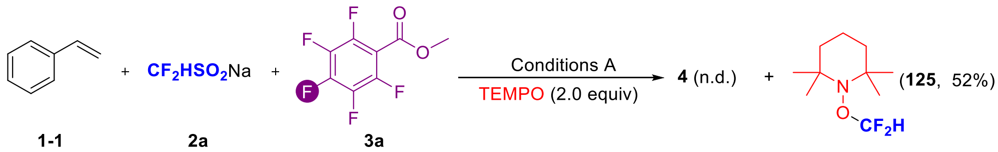

Into a nitrogen-filled glove box, a vial (10.0 mL) equipped with a magnetic stir bar was charged with 4CzIPN (2.4 mg, 1.5 mol%), $ZnCl_2$ (27.2 mg, 1.0 equiv), K_3PO_4 (42.5 mg,

1.0 equiv), and dry DMSO (2.0 mL). Then styrene **1-1** (21.3 mg, 0.2 mmol), $NaSO_2CF_2H$ **2a** (56.0 mg, 2.0 equiv) and methyl pentafluorobenzoate **3a** (90.4 mg, 2.0 equiv) were added. Finally, TEMPO (0.4 mmol, 2.0 equiv) was added to the mixture. The vial was removed from the glovebox, and then the reaction mixture was irradiated with 456 nm Blue LEDs at room temperature for 48 hours. After the reaction finished that was monitored by TLC, The TEMPO product **125** was detected by **^{19}F NMR** and **GC-MS**.

GC-MS of the 125

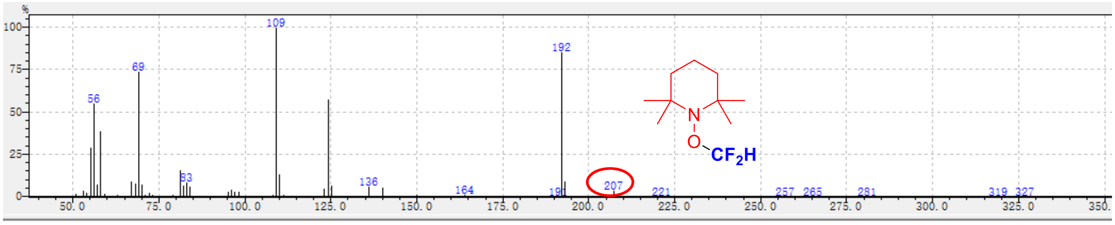

^{19}F NMR (565 MHz, $CDCl_3$) spectrum of 125

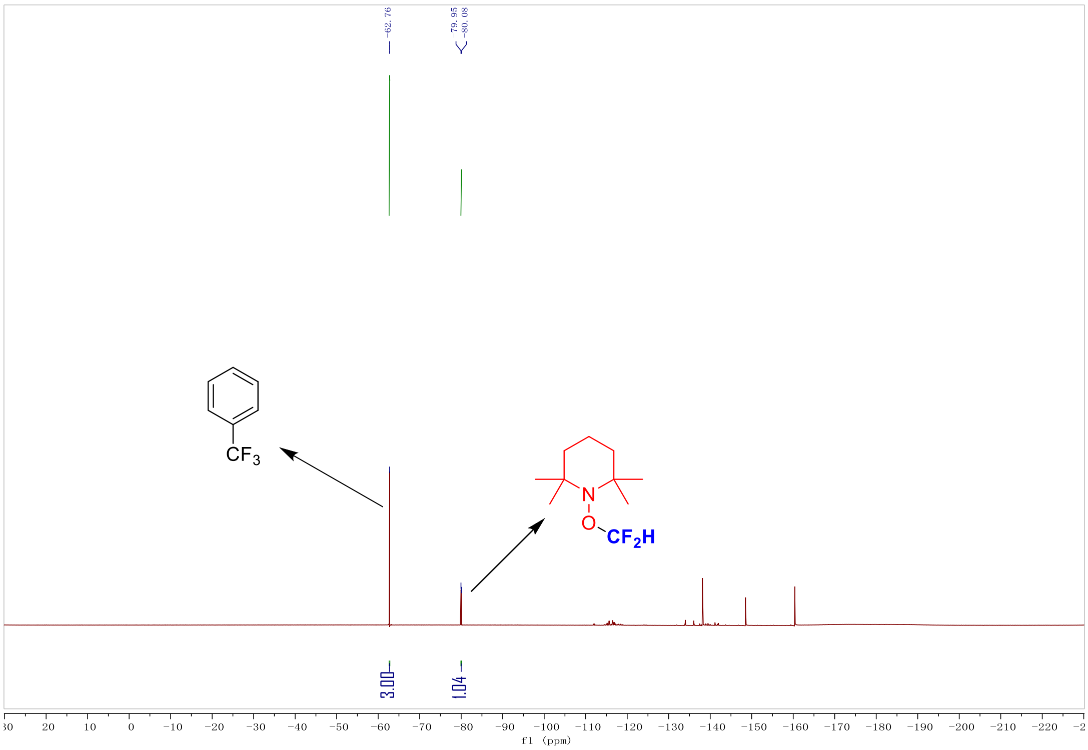

c) Radical clock Experiment

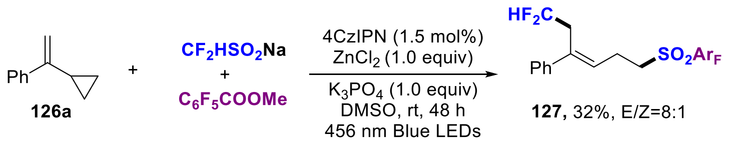

Into a nitrogen-filled glove box, a vial (10.0 mL) equipped with a magnetic stir bar was charged with 4CzIPN (2.4 mg, 1.5 mol%), $ZnCl_2$ (27.2 mg, 1.0 equiv), K_3PO_4 (42.5 mg, 1.0 equiv), and dry DMSO (2.0 mL). Then alkene **126a** (0.2 mmol), $NaSO_2CF_2H$ **2a** (56.0 mg, 2.0 equiv) and methyl pentafluorobenzoate **3a** (90.4 mg, 2.0 equiv) were added. The vial was removed from the glovebox, and then the reaction mixture was

stirred with 456 nm Blue LEDs at room temperature for 48 hours. After the reaction, ethyl acetate and water were poured into the mixture. The organic layer was washed with brine, dried over Na_2SO_4 and filtered. And the reaction mixture was concentrated in vacuo. The resulting crude product was purified by flash column chromatography on silica gel (petroleum ether: ether acetate = 12:1) to obtain product **127**.

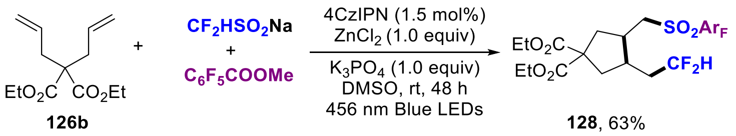

Into a nitrogen-filled glove box, a vial (10.0 mL) equipped with a magnetic stir bar was charged with 4CzIPN (2.4 mg, 1.5 mol%), $ZnCl_2$ (27.2 mg, 1.0 equiv), K_3PO_4 (42.5 mg, 1.0 equiv), and dry DMSO (2.0 mL). Then alkene **126b** (0.2 mmol), $NaSO_2CF_2H$ **2a** (56.0 mg, 2.0 equiv) and methyl pentafluorobenzoate **3a** (90.4 mg, 2.0 equiv) were added. The vial was removed from the glovebox, and then the reaction mixture was stirred with 456 nm Blue LEDs at room temperature for 48 hours. After the reaction, ethyl acetate and water were poured into the mixture. The organic layer was washed with brine, dried over Na_2SO_4 and filtered. And the reaction mixture was concentrated in vacuo. The resulting crude product was purified by flash column chromatography on silica gel (petroleum ether: ether acetate = 10:1) to obtain product **128**.

d) By-product analysis

(1)

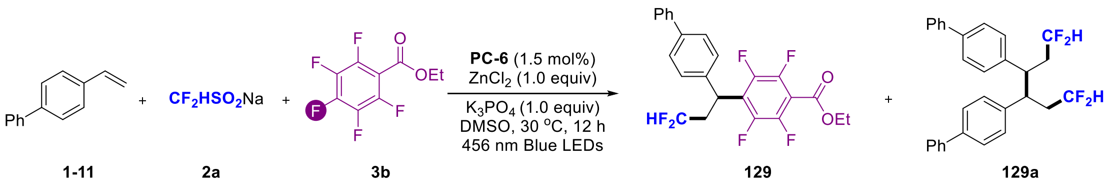

In a nitrogen-filled glovebox, a flame-dried screw-cap reaction tube equipped with a Teflon-coated magnetic stir bar were charged with **PC-6** (3.0 mg, 1.5 mol%), $ZnCl_2$ (27.2 mg, 1.0 equiv), K_3PO_4 (42.5 mg, 1.0 equiv), and dry DMSO (2.0 mL). Then styrene **1-11** (0.2 mmol), $NaSO_2CF_2H$ **2a** (2.0 equiv) and ethyl pentafluorobenzoate **3b** (2.0 equiv) were added. The reaction mixture was irradiated with 456 nm Blue LEDs at 30 °C for 12 hours, until the reaction was complete as indicated by TLC. After the reaction, ethyl acetate and water were poured into the mixture. The organic layer was washed with brine, dried over Na_2SO_4 and filtered. And the reaction mixture was concentrated in vacuo. The resulting crude product was purified by flash column chromatography on silica gel (petroleum ether: ether acetate = 18:1) to obtain product **129** and **129a**. The details and characterization datas of the products were stated below.

(2)

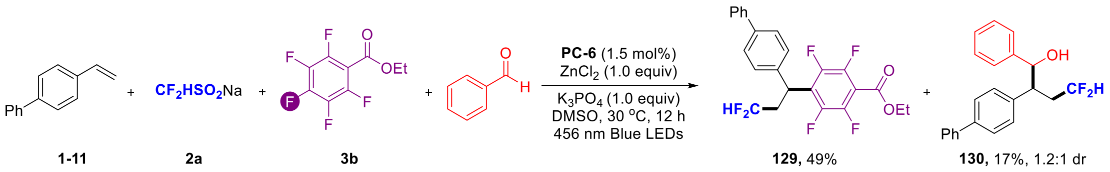

In a nitrogen-filled glovebox, a flame-dried screw-cap reaction tube equipped with a Teflon-coated magnetic stir bar were charged with **PC-6** (3.0 mg, 1.5 mol%), $ZnCl_2$ (27.2 mg, 1.0 equiv), K_3PO_4 (42.5 mg, 1.0 equiv), and dry DMSO (2.0 mL). Then styrene **1-11** (0.2 mmol), $NaSO_2CF_2H$ **2a** (2.0 equiv) ethyl pentafluorobenzoate **3b** (2.0 equiv) and benzaldehyde (0.2 mmol) were added. The reaction mixture was irradiated with 456 nm Blue LEDs at 30 °C for 12 hours, until the reaction was complete as indicated by TLC. After the reaction, ethyl acetate and water were poured into the mixture. The organic layer was washed with brine, dried over Na_2SO_4 and filtered. And the reaction mixture was concentrated in vacuo. The resulting crude product was purified by flash column chromatography on silica gel (petroleum ether: ether acetate = 12:1) to obtain product **129** and **130**. The details and characterization datas of the products were stated below.

e) Two-step synthesis

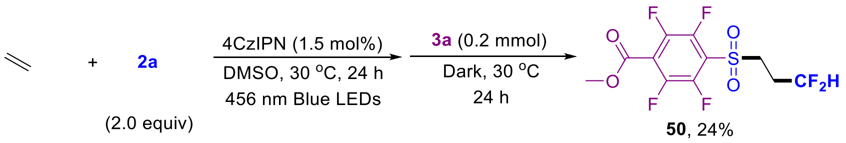

In a schlenk tube, the tubes were filled with 4CzIPN (2.4 mg, 1.5 mol%), dry DMSO (2.0 mL). Then $NaSO_2CF_2H$ **2a** (56.0 mg, 2.0 equiv) was added. Then the nitrogen in the reaction device is pumped away and filled with ethylene gas. The reaction mixture was irradiated with 456 nm Blue LEDs at 30 °C for 24 hours. Then take the reaction device into the glovebox again and methyl pentafluorobenzoate **3a** (45.2 mg, 0.2 mmol) were added. The schlenk tube was removed from the glovebox and then the reaction at 30 °C for 24 hours in dark. The yield of **50** was determined via **^{1}H NMR** analysis of the crude reaction mixture using dibromomethane as an internal standard.

f) Identification of reaction mixture

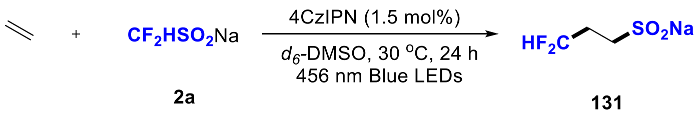

In a schlenk tube, the tubes were filled with 4CzIPN (2.4 mg, 1.5 mol%), dry d_6-DMSO (2.0 mL). Then $NaSO_2CF_2H$ **2a** (28.0 mg, 0.2 mmol) was added. Then the nitrogen in the reaction device is pumped away and filled with ethylene gas. The reaction mixture was irradiated with 456 nm Blue LEDs at 30 °C for 24 hours. The product of **131** was determined via **^{1}H NMR**, **^{13}C NMR, ^{19}F NMR** and **HRMS** analysis of the crude reaction mixture.

^{1}H NMR (600 MHz, DMSO-*d*$_6$) *δ* 6.20 (tt, *J* = 57.5, 4.5 Hz, 1H), 2.54 – 2.51 (m, 2H), 2.15 – 2.02 (m, 2H).
^{13}C NMR (151 MHz, DMSO-*d*$_6$) *δ* 117.58 (t, *J* = 236.8 Hz), 44.59 (t, *J* = 5.4 Hz), 30.82 (t, *J* = 21.7 Hz).
^{19}F NMR (565 MHz, DMSO-*d*$_6$) *δ* -117.97 (dt, *J* = 57.3, 18.0 Hz), -125.29 (d, *J* = 54.3 Hz, CF_2HSO_2Na).
HRMS (ESI) (m/z): calcd for calcd for $C_3H_5F_2O_2S^-$, 142.9984; found, 142.9972.

HRMS test for reaction mixture

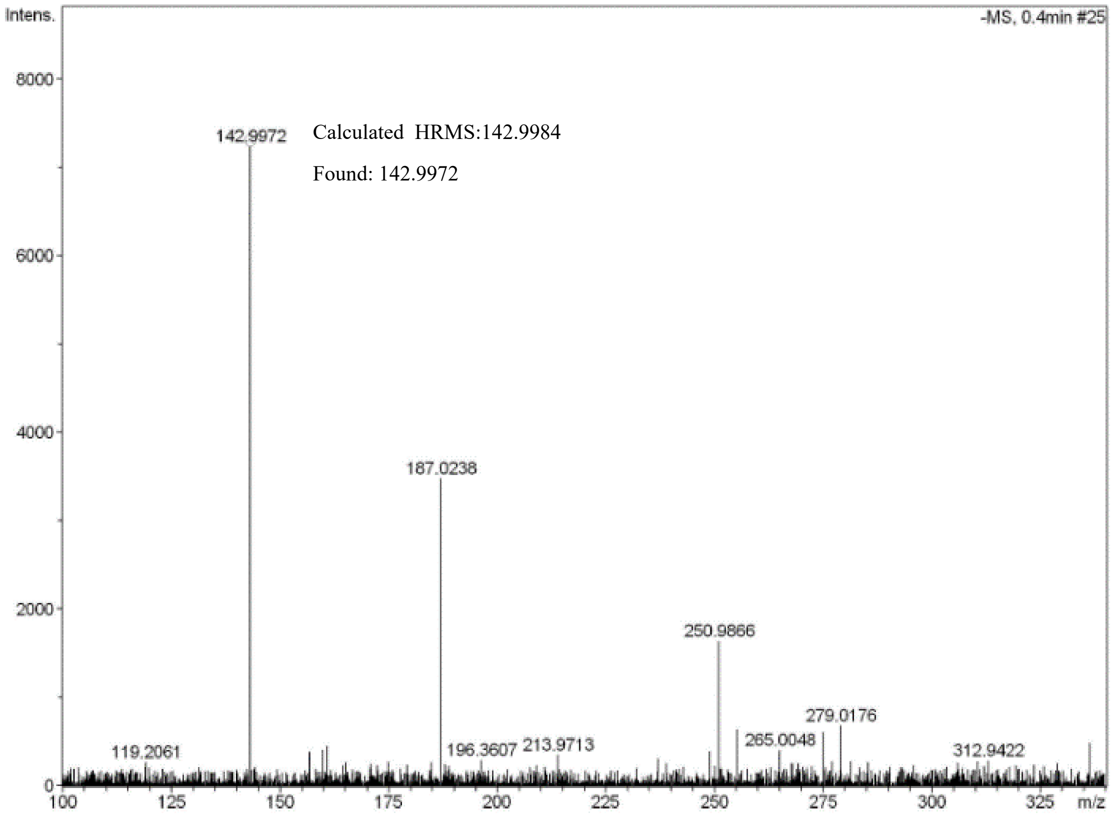

^{1}H NMR of **131** and **2a** mixture

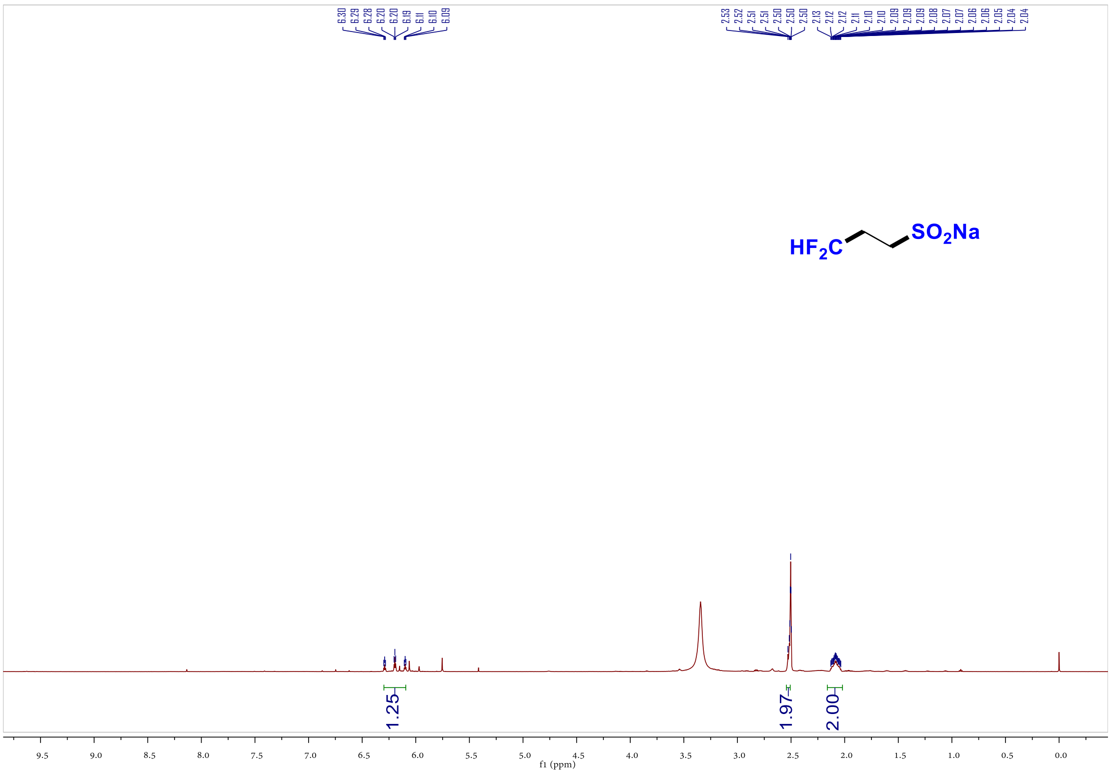

^{13}C NMR of **131** and **2a** mixture

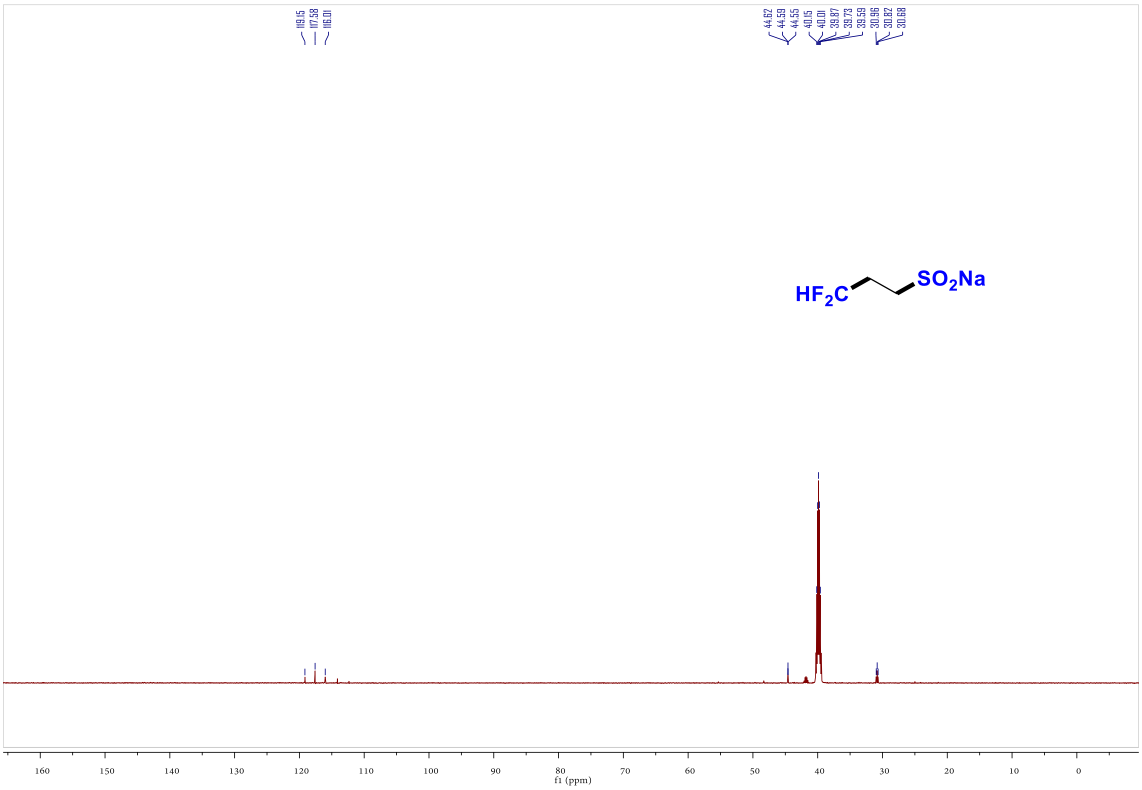

^{19}F NMR of **131** and **2a** mixture

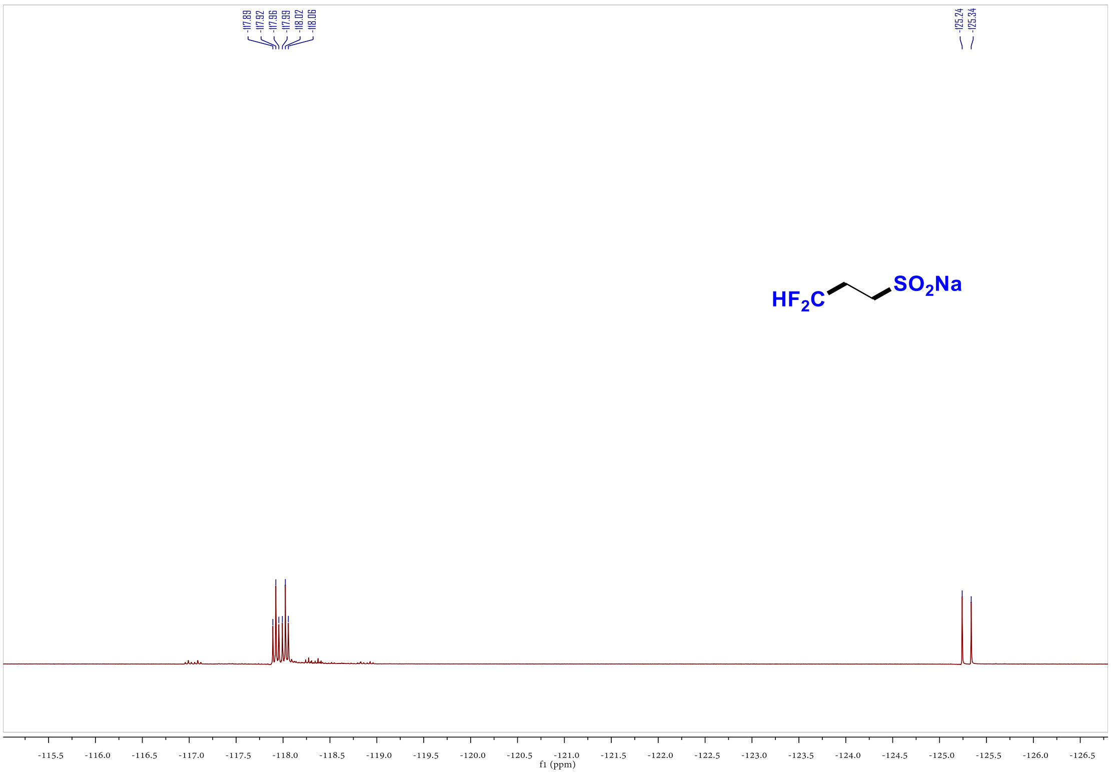

g) Monitoring of reaction kinetics

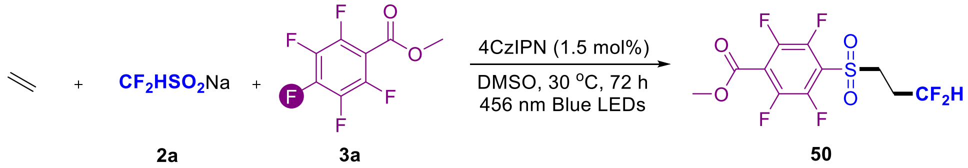

Following **Conditons C**, as shown above. The intermediate **131**, **3a** and **50** contents in the system were monitored at different reaction times. The yield of **3a**, **50** and intermediate **131** were determined via **^{19}F NMR** analysis of the crude reaction mixture using benzo trifluoride as an internal standard.

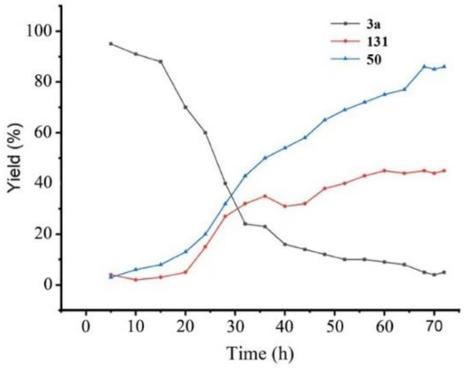

Figure **S1**. Monitoring of reaction kinetics

Time(h)	5	10	15	20	24	28	32	36	40
**3a**	95	91	88	70	60	40	24	23	16
**50**	3	6	8	13	20	32	43	50	54
**131**	4	2	3	5	15	27	32	35	31

Time(h)	44	48	52	56	60	64	68	70	72
**3a**	14	12	10	10	9	8	5	4	5
**50**	58	65	69	72	75	77	86	85	86
**131**	32	38	40	43	45	44	45	44	45

h) Emission quenching experiment

Emission intensities were recorded using a spectrofluorometer (Edinburgh FS5) at ambient temperature. 4CzIPN solutions were excited at 390 nm or 430 nm, and the emission intensity at 550 nm was observed. Firstly, the emission spectrum of a 5 x 10^{-5} M solution of 4CzIPN in DMSO was collected. Then, an appropriate amount of quencher was added to the measured solution, and the emission spectrum of the sample was collected. Stern-Volmer emission quenching studies have shown that only $NaSO_2CF_2H$ **2a** can quench the excited photosensitizer.

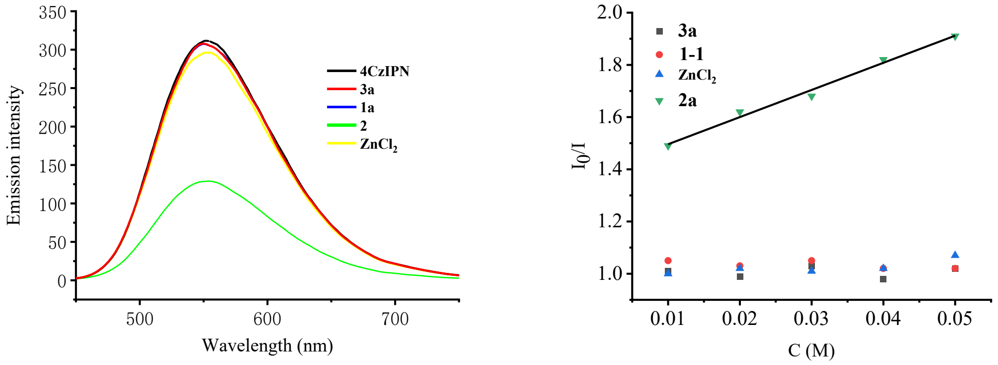

Figure **S2**. 4CzIPN emission quenching by styrene **1-1,** $NaSO_2CF_2H$ **2a,** methyl pentafluorobenzoate **3a** and $ZnCl_2$

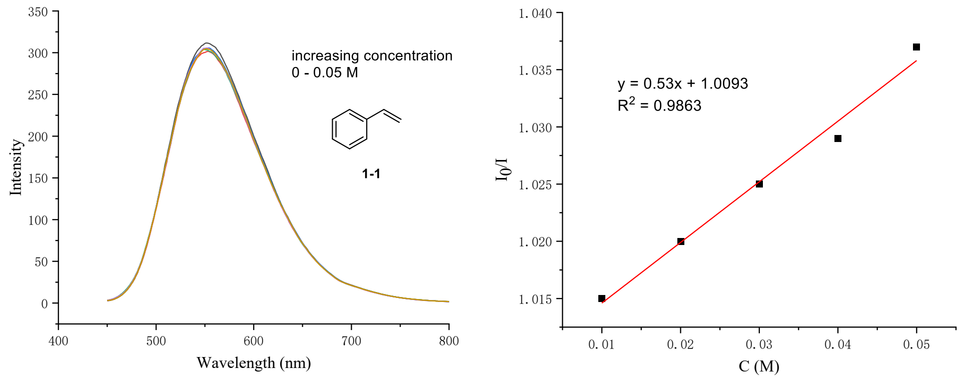

Figure **S3**. 4CzIPN emission quenching by styrene **1-1**

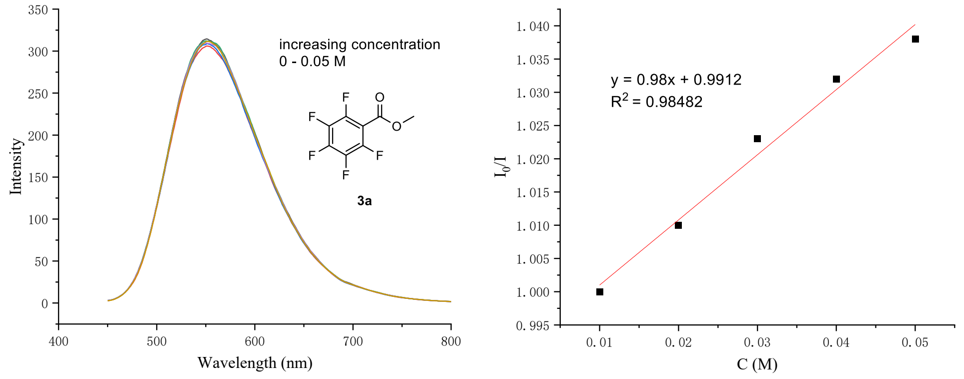

Figure **S4**. 4CzIPN emission quenching by methyl pentafluorobenzoate **3a**

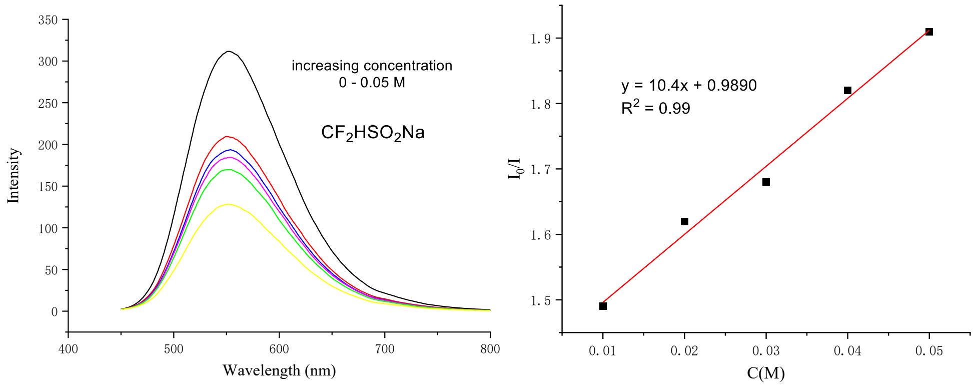

Figure **S5**. 4CzIPN emission quenching by $Na_2SO_2CF_2H$ **2a**

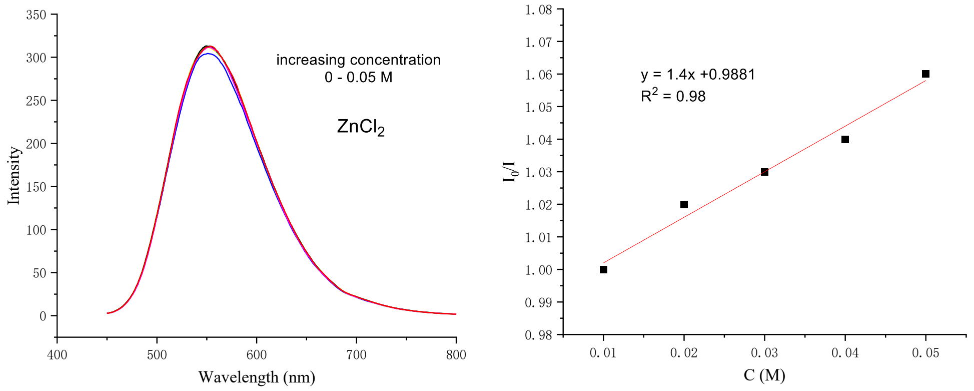

Figure **S6**. 4CzIPN emission quenching by $ZnCl_2$

Emission intensities were recorded using a spectrofluorometer (Edinburgh FS5) at ambient temperature. **PC-6** solutions were excited at 380 nm, and the emission intensity at 520 nm was observed. Stern-Volmer emission quenching studies have shown that only $NaSO_2CF_2H$ **2a** can quench the excited photosensitizer (Figuer S7 - Figuer S11).

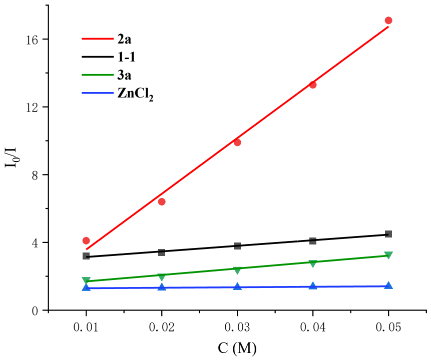

Figure S7. **PC-6** emission quenching by styrene **1-1,** $NaSO_2CF_2H$ **2a,** methyl pentafluorobenzoate **3a** and $ZnCl_2$

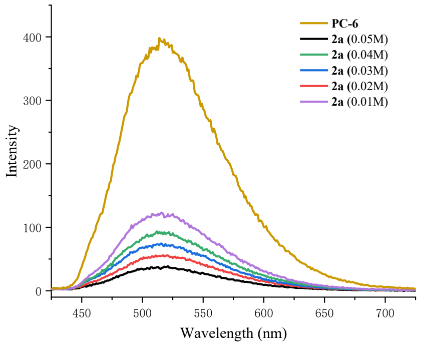

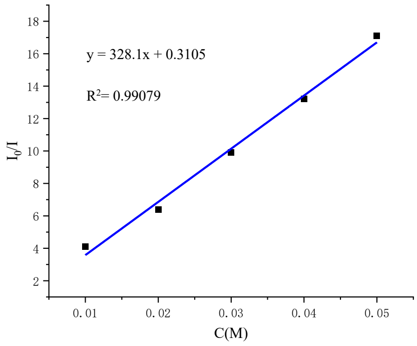

Figure **S8**. **PC-6** emission quenching by styrene **2a**

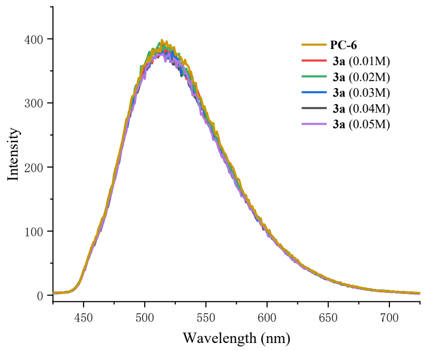

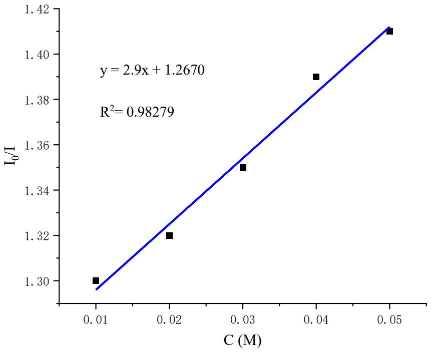

Figure **S9**. **PC-6** emission quenching by styrene **3a**

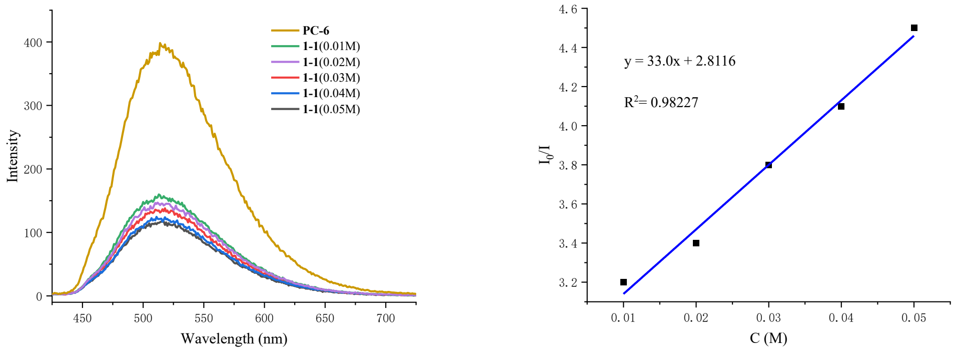

Figure **S10**. **PC-6** emission quenching by styrene **1-1**

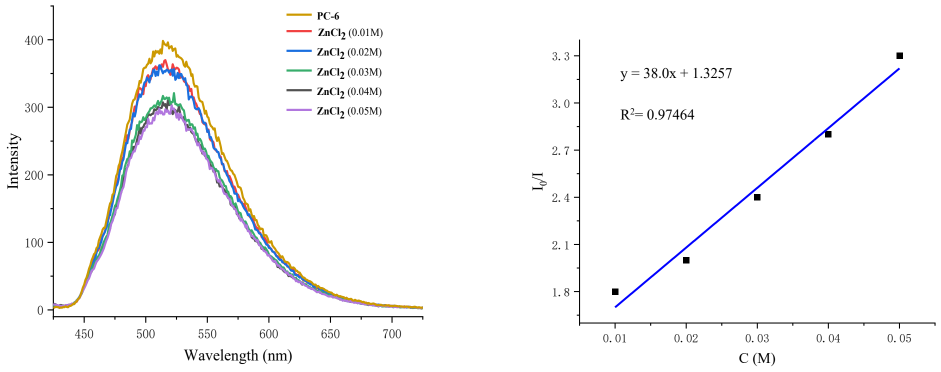

Figure **S11**. **PC-6** emission quenching by styrene **ZnCl₂**

i) Characterization of a newly formed photocatalyst

(1)

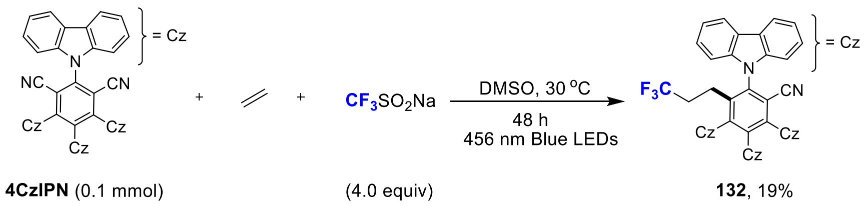

In a nitrogen-filled glovebox, a flame-dried screw-cap reaction tube equipped with a Teflon-coated magnetic stir bar were charged with 4CzIPN (0.1 mmol), ethylene (1 atm, balloon), $NaSO_2CF_3$ (4.0 equiv) and DMSO (2 mL) were added. The reaction mixture was irradiated with 456 nm Blue LEDs at 30 °C for 48 hours, until the reaction was complete as indicated by TLC. After the reaction, ethyl acetate and water were poured into the mixture. The organic layer was washed with brine, dried over Na_2SO_4 and filtered. And the reaction mixture was concentrated in vacuo. The resulting crude product was purified by flash column chromatography on silica gel (petroleum ether: ether acetate = 8:1) to obtain product **132**.

(2)

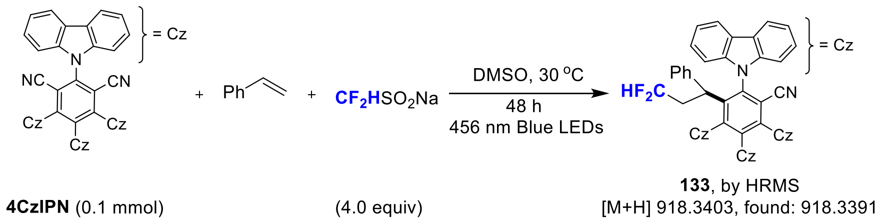

In a nitrogen-filled glovebox, a flame-dried screw-cap reaction tube equipped with a Teflon-coated magnetic stir bar were charged with 4CzIPN (0.1 mmol), styrene (4.0 equiv), $NaSO_2CF_2H$ **2a** (4.0 equiv) and DMSO (2 mL) were added. The reaction mixture was irradiated with 456 nm Blue LEDs at 30 ºC for 48 hours, until the reaction was complete as indicated by TLC. After the reaction, ethyl acetate and water were poured into the mixture. The organic layer was washed with brine, dried over Na_2SO_4 and filtered. And the reaction mixture was concentrated in vacuo. The product of **133** was determined via **HRMS** analysis of the crude reaction mixture.

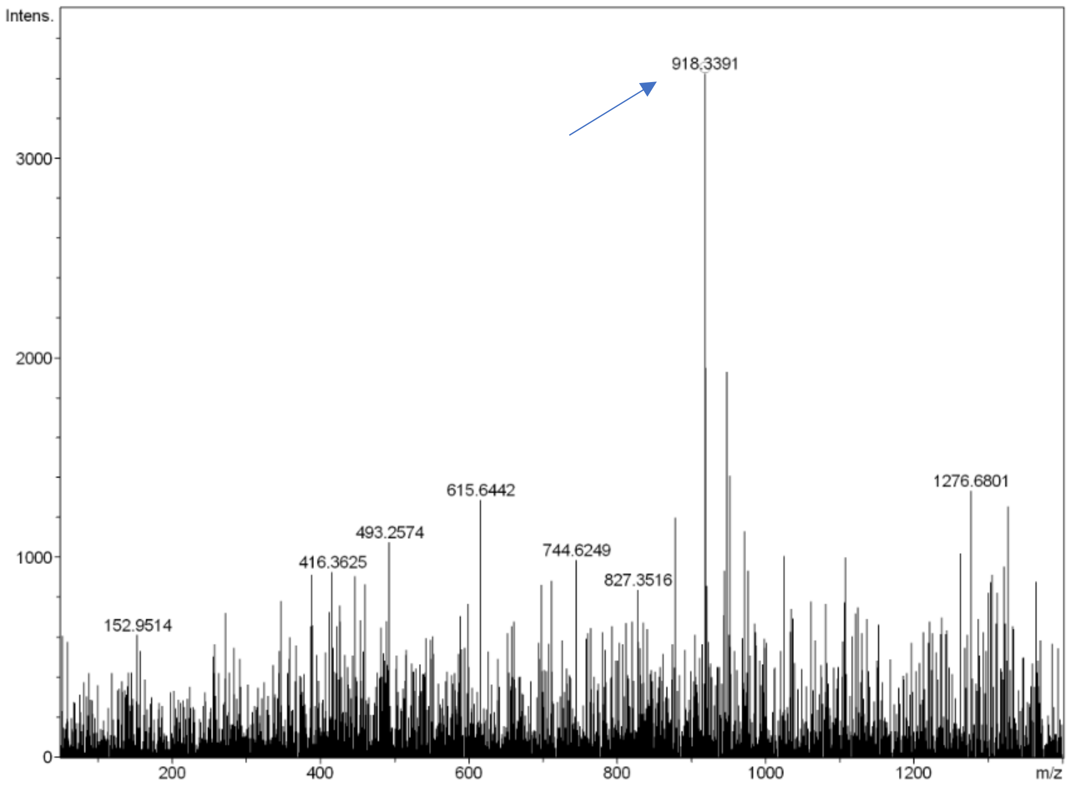

HRMS test for reaction mixture

j) Catalytic activity of photocatalyst 132 VS 4CzIPN.

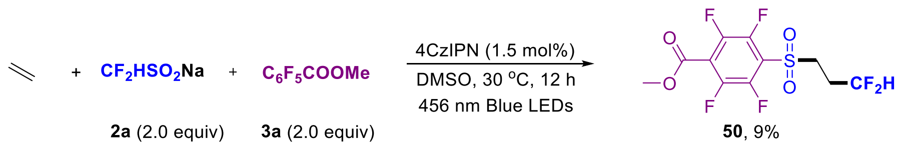

In a nitrogen-filled glovebox, a flame-dried screw-cap reaction tube equipped with a Teflon-coated magnetic stir bar were charged with 4CzIPN (0.80 mg, 0.5 mol%), dry DMSO (2.0 mL). Then methyl pentafluorobenzoate **3a** (2.0 equiv), ethylene (1 atm)

and $NaSO_2CF_2H$ **2a** (2.0 equiv) were added. The reaction mixture was irradiated with 456 nm Blue LEDs at 30 °C for 12 hours. After the reaction, ethyl acetate and water were poured into the mixture. The organic layer was washed with brine, dried over Na_2SO_4 and filtered. And the reaction mixture was concentrated in vacuo. The resulting crude product was purified by flash column chromatography on silica gel (petroleum ether: ether acetate = 8:1) to obtain product **50** by 9% yield.

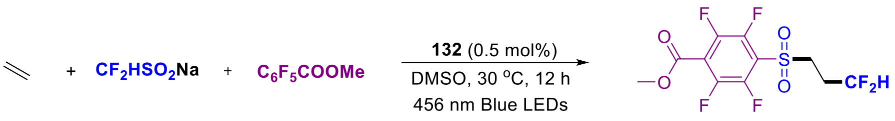

2a (2.0 equiv) **3a** (2.0 equiv) **50**, 15%

In a nitrogen-filled glovebox, a flame-dried screw-cap reaction tube equipped with a Teflon-coated magnetic stir bar were charged with **132** (0.5 mol%), dry DMSO (2.0 mL). Then methyl pentafluorobenzoate **3a** (2.0 equiv), ethylene (1 atm) and $NaSO_2CF_2H$ **2a** (2.0 equiv) were added. The reaction mixture was irradiated with 456 nm Blue LEDs at 30 °C for 12 hours. After the reaction, ethyl acetate and water were poured into the mixture. The organic layer was washed with brine, dried over Na_2SO_4 and filtered. And the reaction mixture was concentrated in vacuo. The resulting crude product was purified by flash column chromatography on silica gel (petroleum ether: ether acetate = 8:1) to obtain product **50** by 15% yield.

k) Potential role of PC as a reversible radical buffer.

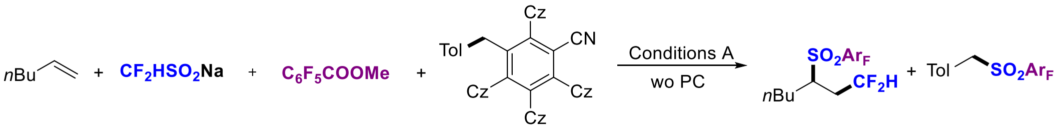

0.2mmol **2a** (2.0 equiv) **3a** (2.0 equiv) **134**, 20 mol% **135**, 65% **136**, 7%

Into a nitrogen-filled glove box, a vial (10.0 mL) equipped with a magnetic stir bar was charged with **134** (20 mol%), $ZnCl_2$ (27.2 mg, 1.0 equiv), K_3PO_4 (42.5 mg, 1.0 equiv), and dry DMSO (2.0 mL). Then 1-hexene (0.2 mmol), $NaSO_2CF_2H$ **2a** (56.0 mg, 2.0 equiv) and methyl pentafluorobenzoate **3a** (90.4 mg, 2.0 equiv) were added. The vial was removed from the glovebox, and then the reaction mixture was stirred 456 nm Blue LEDs at room temperature for 48 hours. After the reaction, ethyl acetate and water were poured into the mixture. The organic layer was washed with brine, dried over Na_2SO_4 and filtered, then the solvent was evaporated under vacuum. The residue was analyzed by **^{1}H NMR**, the product **135** and **136** were detected.

l) The D-labeled experiment.

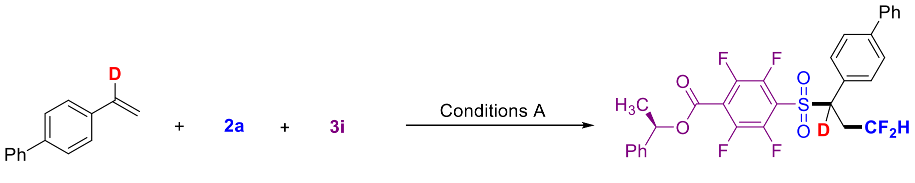

***d₁*-1-11** 0.4 mmol 0.4 mmol **137**, 51%, >95%D

In a nitrogen-filled glovebox, a flame-dried screw-cap reaction tube equipped with a Teflon-coated magnetic stir bar were charged with 4CzIPN (2.4 mg, 1.5 mol%), $ZnCl_2$ (27.2 mg, 1.0 equiv), K_3PO_4 (42.5 mg, 1.0 equiv), and dry DMSO (2.0 mL). Then styrene ***d_1*-1-11** (0.2 mmol), $NaSO_2CF_2H$ **2a** (2.0 equiv) and **3i** (2.0 equiv) were added. The reaction mixture was irradiated with 456 nm Blue LEDs at room tempurature for 48 hours, until the reaction was complete as indicated by TLC. After the reaction, ethyl acetate and water were poured into the mixture. The organic layer was washed with brine, dried over Na_2SO_4 and filtered. And the reaction mixture was concentrated in vacuo. The resulting crude product was purified by flash column chromatography on silica gel (petroleum ether: ether acetate = 8:1) to obtain product **137**. The details and characterization datas of the products were stated below.

m) The effect of light-irradiation on diastereoselectivity of S_NAr.

(1)

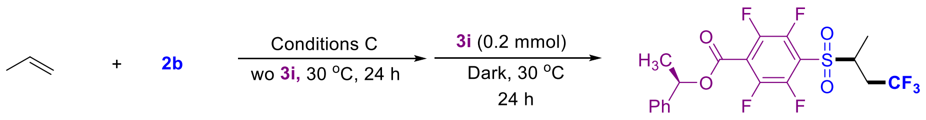

123, 39% 4:1 dr

In a schlenk tube, the tubes were filled with **PC-1** (2.4 mg, 1.5 mol%), dry DMSO (2.0 mL). Then $NaSO_2CF_3$ **2b** (63.2 mg, 2.0 equiv) was added. Then the nitrogen in the reaction device is pumped away and filled with propylene gas. The reaction mixture was irradiated with 456 nm Blue LEDs at 30 °C for 24 hours. Then take the reaction device into the glovebox again and **3i** (0.2 mmol) were added. The schlenk tube was removed from the glovebox and then the reaction at 30 °C for 24 hours in dark. The yield of **123** was determined via **^{1}H NMR** analysis of the crude reaction mixture using dibromomethane as an internal standard. The diastereomeric ratio (d.r.) was assessed by **^{1}H NMR** in $CDCl_3$ of the reaction mixture.

^{1}H NMR (600 MHz, $CDCl_3$) δ 7.44 – 7.37 (m, 4.21H), 7.37 – 7.33 (m, 1.20H), 6.23 – 6.15 (m, 1.16H), 3.69 (dd, J = 14.3, 2.7 Hz, 0.26H), 3.59 (ddt, J = 13.6, 9.5, 4.8 Hz, 1.00H), 3.05 – 2.95 (m, 0.24H), 2.95 – 2.85 (m, 1.02H), 2.45– 2.39 (m, 1.16H), 1.72 – 1.70 (m, 3.40H), 1.55 (d, J = 7.5 Hz, 3.03H), 1.44 (d, J = 7.0 Hz, 0.62H).

^{13}C NMR (151 MHz, $CDCl_3$) δ 157.26, 146.07 – 145.37 (m), 144.35 – 143.42 (m), 139.71, 139.68, 128.76, 128.67, 128.65, 126.26, 126.24, 125.39 (t, J = 279.6 Hz), 119.03 (t, J = 17.1 Hz), 118.47 (t, J = 14.1 Hz), 76.61, 76.56, 57.48, 56.74, 34.06 (q, J = 28.9 Hz), 33.12 (q, J = 30.7 Hz), 22.00, 21.98, 13.35, 13.27.

^{19}F NMR (565 MHz, $CDCl_3$) δ -63.23 (t, J = 10.3 Hz), -73.45 (t, J = 8.2 Hz), -133.56 – -133.72 (m), -134.93 – -135.08 (m), -135.13 – -135.34 (m).

^{1}H NMR (600 MHz, CDCl$_3$) spectrum of 123

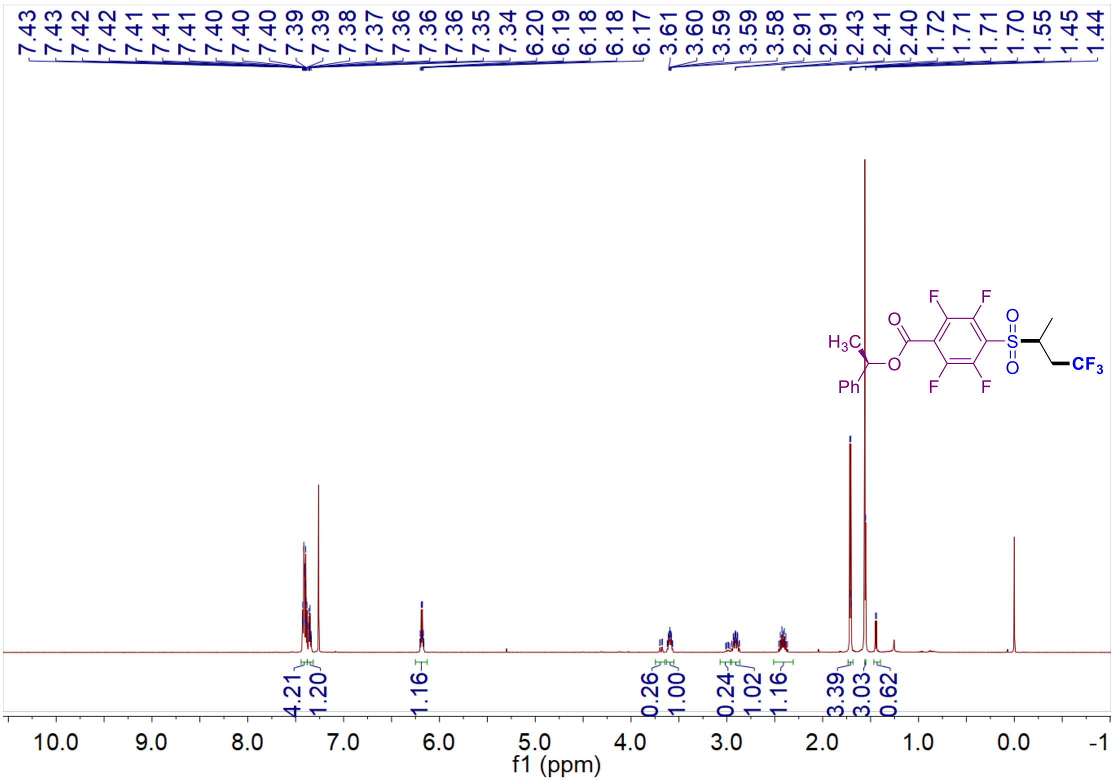

^{13}C NMR (151 MHz, CDCl$_3$) spectrum of 123

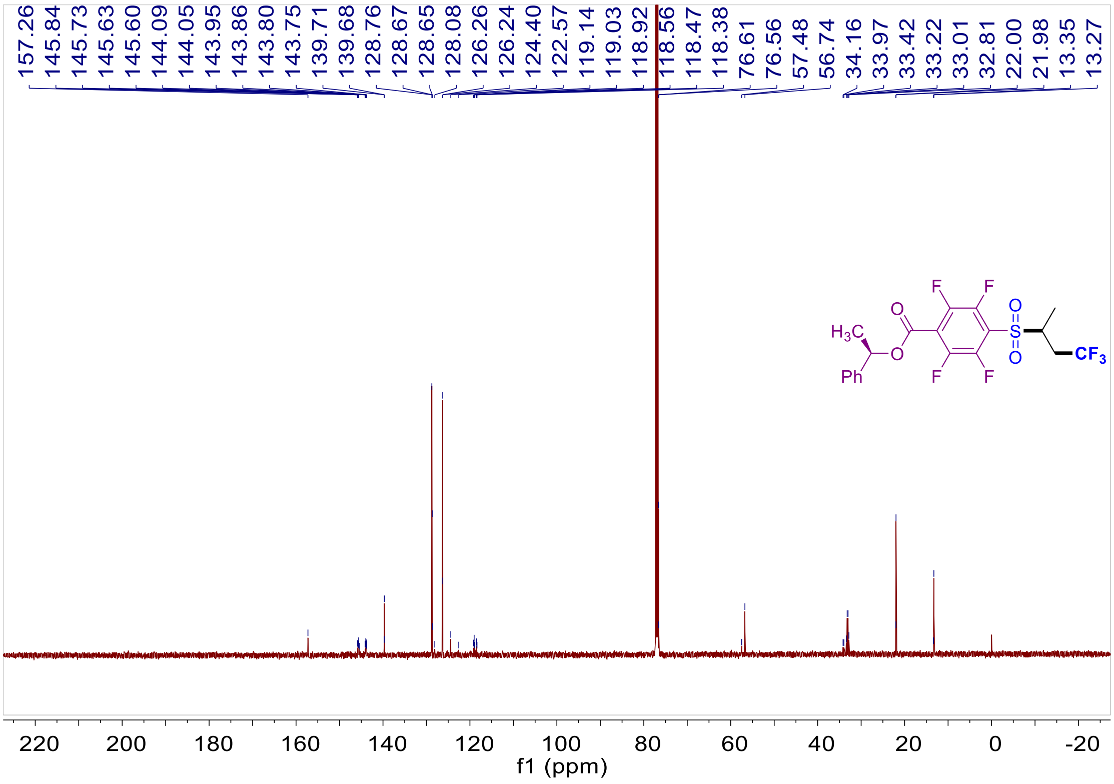

^{19}F NMR (565 MHz, $CDCl_3$) spectrum of 123

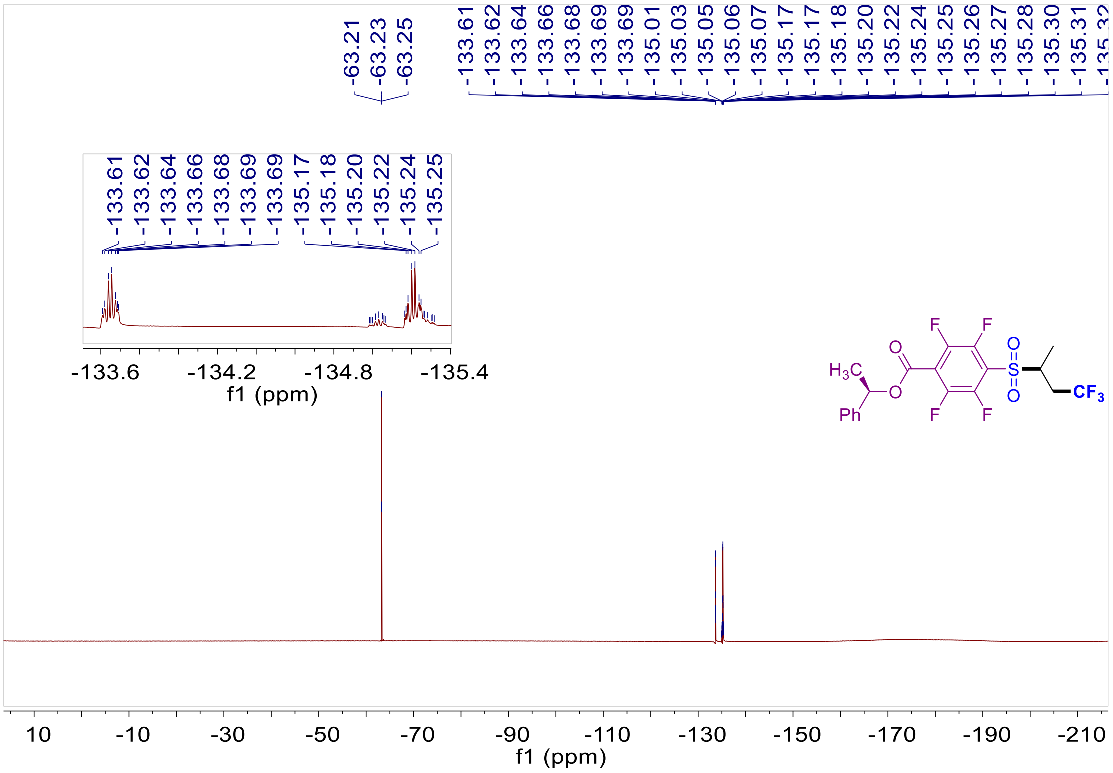

(2)

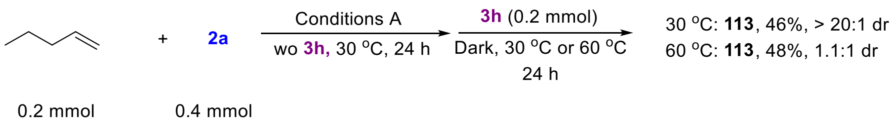

Into a nitrogen-filled glove box, a vial (10.0 mL) equipped with a magnetic stir bar was charged with 4CzIPN (2.4 mg, 1.5 mol%), $ZnCl_2$ (27.2 mg, 1.0 equiv), K_3PO_4 (42.5 mg, 1.0 equiv), and dry DMSO (2.0 mL). Then 1-Pentene (14.2 mg, 0.2 mmol) and $NaSO_2CF_2H$ **2a** (56.0 mg, 2.0 equiv) were added. The vial was removed from the glovebox, and then the reaction mixture was stirred at 30 °C with 456 nm Blue LEDs for 24 hours. Then **3h** (0.2 mmol) was added and was removed from the glovebox and then the reaction at 30 °C or 60 °C for 24 hours in dark After the reaction, ethyl acetate and water were poured into the mixture. The organic layer was washed with brine, dried over Na_2SO_4 and filtered, then the solvent was evaporated under vacuum. The residue was analyzed by **^{1}H NMR**, the product **113** was detected. The diastereomeric ratio (d.r.) was assessed by **^{1}H NMR** in $CDCl_3$ of the reaction mixture.

NMR analysis of the product at 60 °C:

^{1}H NMR (600 MHz, $CDCl_3$) δ 7.57 – 7.52 (m, 3.45H), 7.51 – 7.42 (m, 5.28H), 6.37 (q, J = 6.5 Hz, 1.79H), 6.15 (tdd, J = 56.2, 5.4, 3.6 Hz, 1.97H), 3.47 (qt, J = 9.1, 5.0 Hz, 1.89H), 2.63 – 2.44 (m, 1.96H), 2.28 (qq, J = 15.6, 5.1 Hz, 1.93H), 1.93 (dtt, J = 20.3, 10.4, 5.2 Hz, 1.93H), 1.75 (m, 2.03H), 1.60 (dddd, J = 13.4, 10.5, 7.4, 4.9 Hz, 0.98H), 1.55 – 1.49 (m, 1.17H), 1.47 – 1.24 (m, 2.22H), 0.96 (t, J = 7.3 Hz, 3H), 0.91 (t, J = 7.2 Hz, 2.88H).

^{13}C NMR (151 MHz, CDCl₃) δ 156.30, 146.20 – 145.53 (m), 144.94 – 143.45 (m), 130.60, 129.53, 128.99, 128.20, 122.55 (q, J = 280.7 Hz), 121.23 – 120.69 (m), 116.35 (t, J = 15.6 Hz), 114.42 (t, J = 240.6 Hz), 74.43 (q, J = 34.1 Hz), 61.21 (t, J = 4.9 Hz), 61.01 (t, J = 5.0 Hz), 32.19 (t, J = 23.5 Hz), 30.32, 28.04 (d, J = 3.0 Hz), 22.24, 19.36, 13.58, 13.53(d, J = 3.5 Hz).

^{19}F NMR (565 MHz, CDCl₃) δ-75.89 (dd, J = 6.2, 3.1 Hz), -114.12 – -115.04 (m), -116.97 – -118.28 (m), -133.41 – -133.59 (m), -133.94 – -134.18 (m).

^{1}H NMR (600 MHz, CDCl₃) spectrum of 113

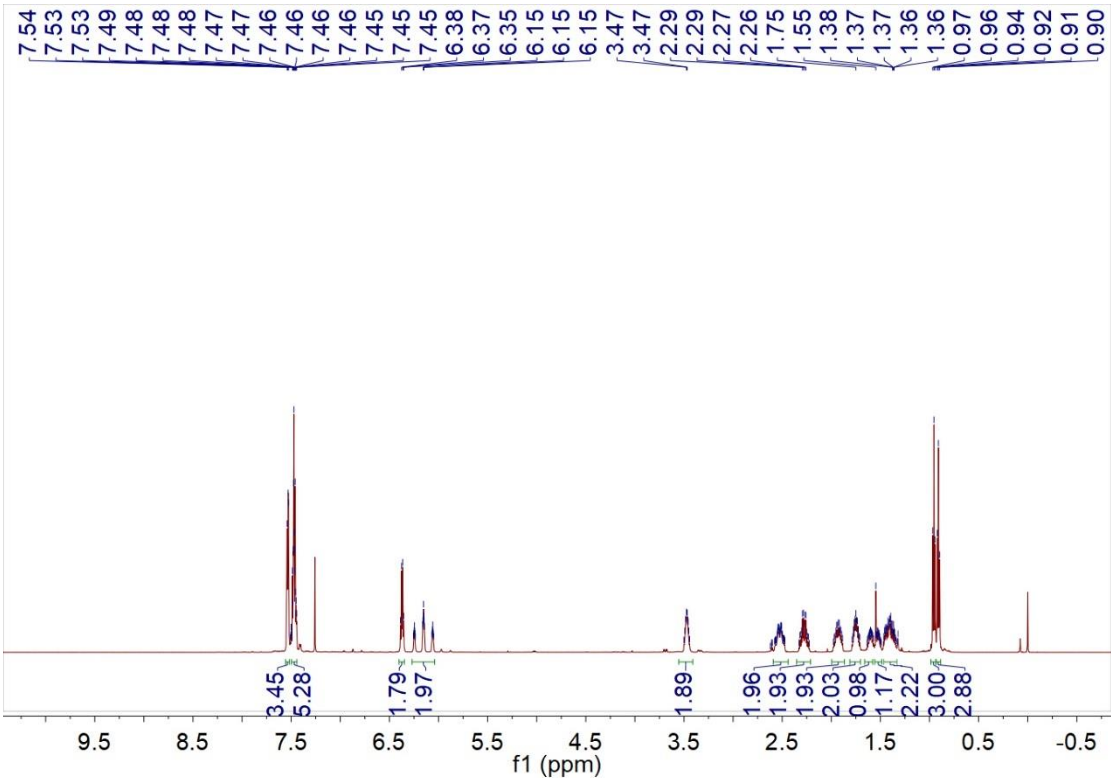

^{13}C NMR (151 MHz, $CDCl_3$) spectrum of 113

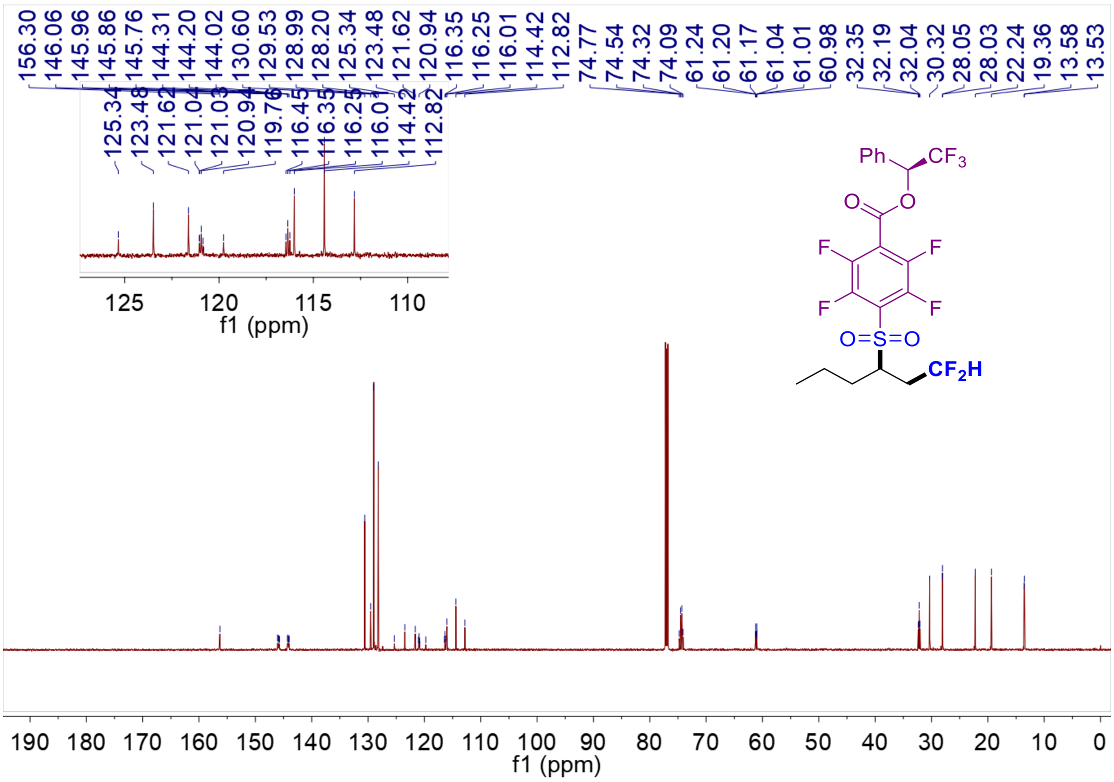

^{19}F NMR (565 MHz, $CDCl_3$) spectrum of 113

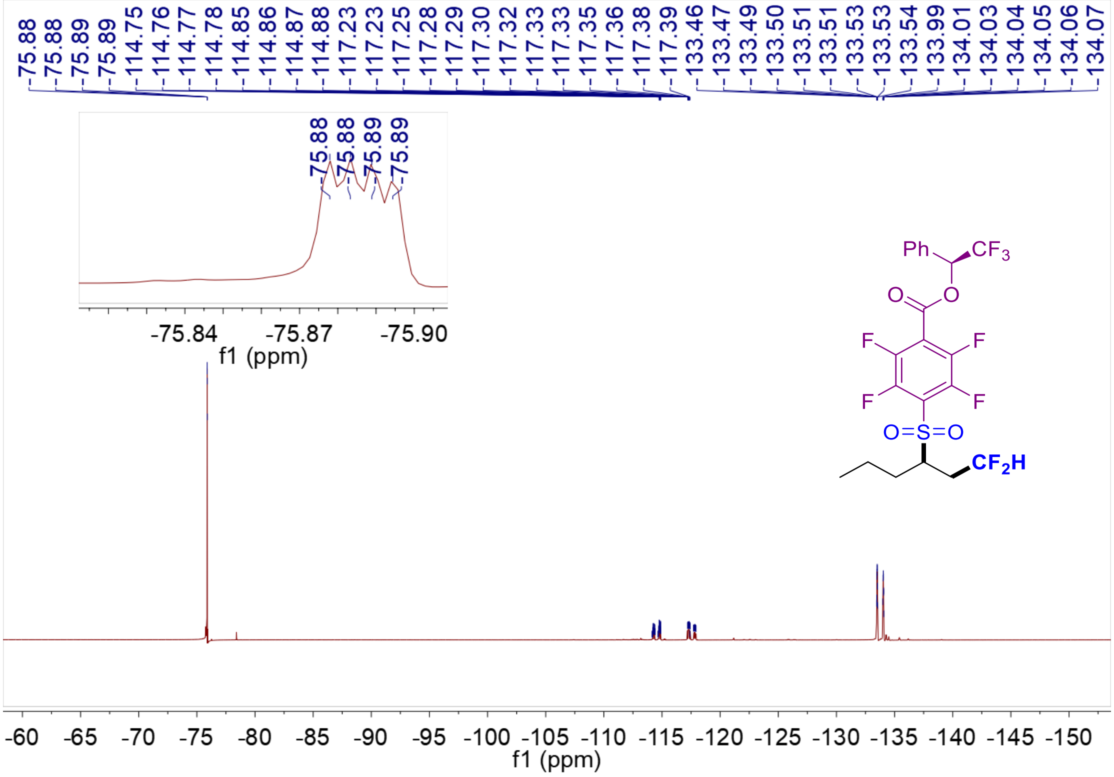

n) the effect of PC on *ee* value of sodium sulfonate in the reaction system.

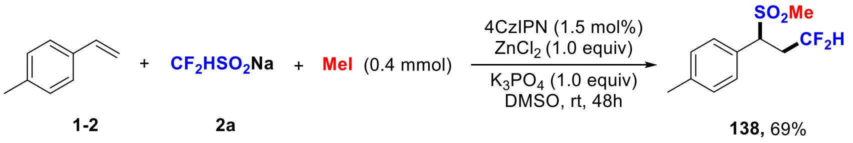

In a nitrogen-filled glovebox, a flame-dried screw-cap reaction tube equipped with a Teflon-coated magnetic stir bar were charged with 4CzIPN (2.4 mg, 1.5 mol%), $ZnCl_2$ (27.2 mg, 1.0 equiv), K_3PO_4 (42.5 mg, 1.0 equiv), and dry DMSO (2.0 mL). Then styrene **1-2** (0.2 mmol), $NaSO_2CF_2H$ **2a** (56.0 mg, 2.0 equiv) and Iodomethane (56.8 mg, 2.0 equiv) were added. The reaction mixture was irradiated with 456 nm Blue LEDs at room temperature for 48 hours, until the reaction was complete as indicated by TLC. After the reaction, ethyl acetate and water were poured into the mixture. The organic layer was washed with brine, dried over Na_2SO_4 and filtered. And the reaction mixture was concentrated in vacuo. The resulting crude product was purified by flash column chromatography on silica gel (petroleum ether: ether acetate = 12:1) to obtain product **138**.

(1)

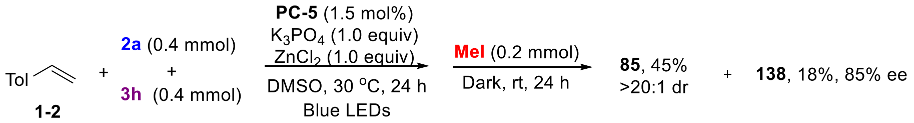

In a nitrogen-filled glovebox, a flame-dried screw-cap reaction tube equipped with a Teflon-coated magnetic stir bar were charged with **PC-5** (2.6 mg, 1.5 mol%), $ZnCl_2$ (27.2 mg, 1.0 equiv), K_3PO_4 (42.5 mg, 1.0 equiv), and dry DMSO (2.0 mL). Then styrene **1-2** (0.2 mmol), $NaSO_2CF_2H$ **2a** (56.0 mg, 2.0 equiv) and **3h** (0.4mmol, 2.0 equiv) were added. The reaction mixture was irradiated with 456 nm Blue LEDs at room temperature for 24 hours. Then iodomethane (28.4 mg, 1.0 equiv) were added. The reaction mixture was irradiated in dark at room temperature for 24 hours, until the reaction was complete as indicated by TLC. After the reaction, ethyl acetate and water were poured into the mixture. The organic layer was washed with brine, dried over Na_2SO_4 and filtered. And the reaction mixture was concentrated in vacuo. The resulting crude product was purified by flash column chromatography on silica gel (petroleum ether: ether acetate = 12:1) to obtain product **85** and **138**. The details and characterization datas of the products were stated below.

HPLC analysis: Daicel Chiralpak OD-H column (98:2 hexane: 2-propanol, 0.7 mL/min, 25 °C, 254 nm); t_R (minor) = 20.488 min, t_R (major) = 30.398 min.

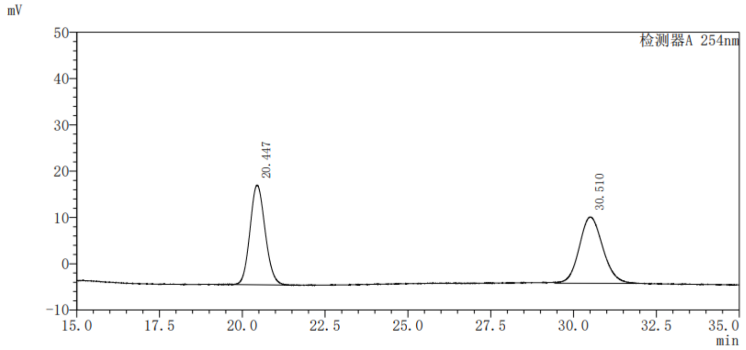

Peak	PetTime	Type	Area	Hight	Area%
1	20.447	M	686421	21543	49.120
2	30.510	M	711021	14333	50.880

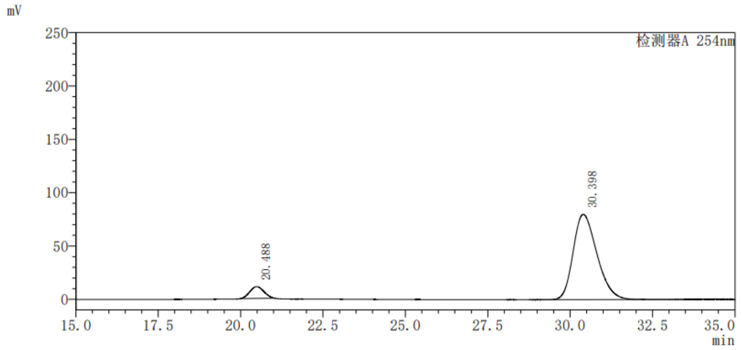

Peak	PetTime	Type	Area	Hight	Area%
1	20.488	M	325827	11062	7.486
2	30.398	M	4026722	79967	92.514

(2)

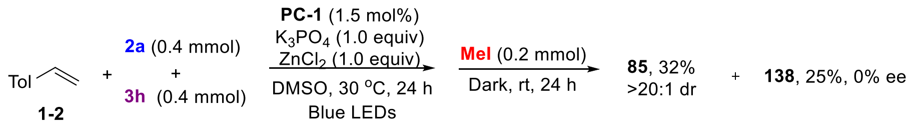

In a nitrogen-filled glovebox, a flame-dried screw-cap reaction tube equipped with a Teflon-coated magnetic stir bar were charged with **PC-1** (2.4 mg, 1.5 mol%), $ZnCl_2$ (27.2 mg, 1.0 equiv), K_3PO_4 (42.5 mg, 1.0 equiv), and dry DMSO (2.0 mL). Then styrene **1-2** (0.2 mmol), $NaSO_2CF_2H$ **2a** (56.0 mg, 2.0 equiv) and **3h** (0.4mmol, 2.0 equiv) were added. The reaction mixture was irradiated with Blue LEDs at 30 °C for 24 hours. Then iodomethane (28.4 mg, 1.0 equiv) were added. The reaction mixture was irradiated in dark at room temperature for 24 hours, until the reaction was complete as indicated by TLC. After the reaction, ethyl acetate and water were poured into the mixture. The organic layer was washed with brine, dried over Na_2SO_4 and filtered. And the reaction mixture was concentrated in vacuo. The resulting crude product was purified by flash column chromatography on silica gel (petroleum ether: ether acetate = 12:1) to obtain product **85** and **138**. The details and characterization data of the products were stated below.

HPLC analysis: Daicel Chiralpak OD-H column (98:2 hexane: 2-propanol, 0.7 mL/min, 25 °C, 254 nm); t_R (minor) = 20.796 min, t_R (major) = 30.795 min.

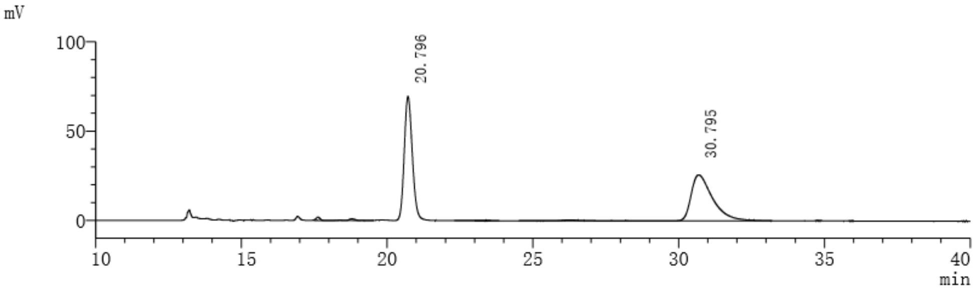

Peak	PetTime	Type	Area	Hight	Area%
1	20.796	M	1339688	69534	50.4832
2	30.795	M	1314040	25777	49.5168

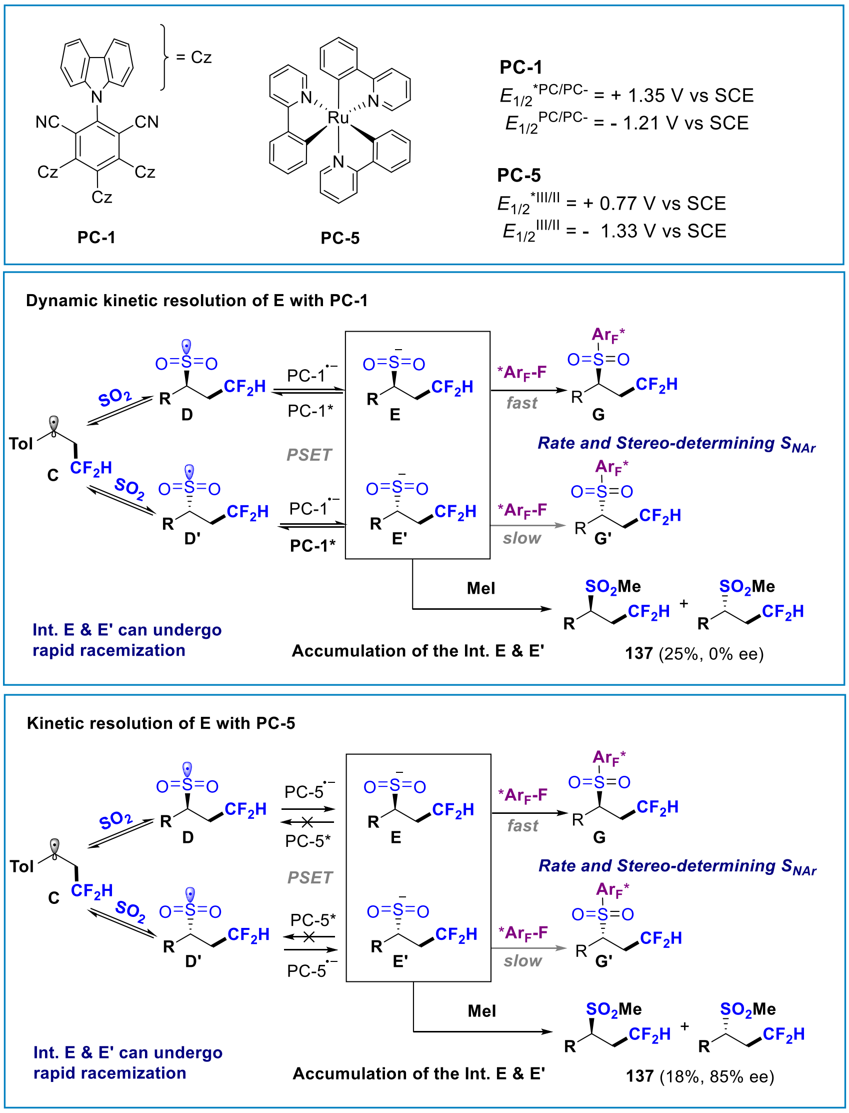

Figure **S12.** Rationale for the PC-dependent *ee* value of the sulfinate anion.

o) Investigation of styrene derivatives in chemo-divergent transformation with PC-1 and PC-6.

The reactivity of styrene derivatives with diverse functional groups was investigated under **Conditions A** (using **PC-1** as the photosensitizer) and **Conditions B** (using **PC-6** as the photosensitizer). The results demonstrate that the **PC-1** system favors the formation of SO_2-reintegration products, whereas the **PC-6** system preferentially leads to the generation of SO_2-release products. Notably, across both photocatalytic systems, electron-deficient styrene derivatives consistently exhibit a stronger tendency toward SO_2 release compared to their electron-rich counterparts.

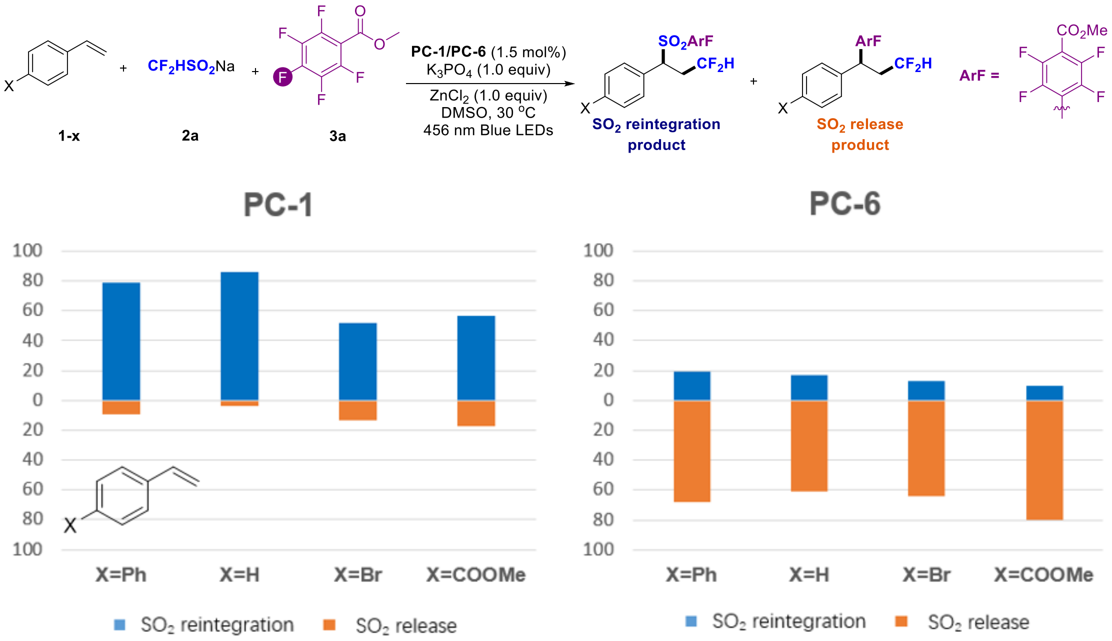

Fig. **S13** Investigation of styrene derivatives in chemo-divergent transformation with

PC-1 and **PC-6**

p) Investigation of the role for $ZnCl_2$.

First, we mixed sodium difluoromethanesulfinate (CF_2HSO_2Na) with $ZnCl_2$ and allowed the mixture to react for 12 hours. NMR analysis confirmed the formation of a new species, suggesting the generation of a new intermediate.

^{1}H NMR (600 MHz, DMSO-*d6*) spectrum of CF_2HSO_2Na

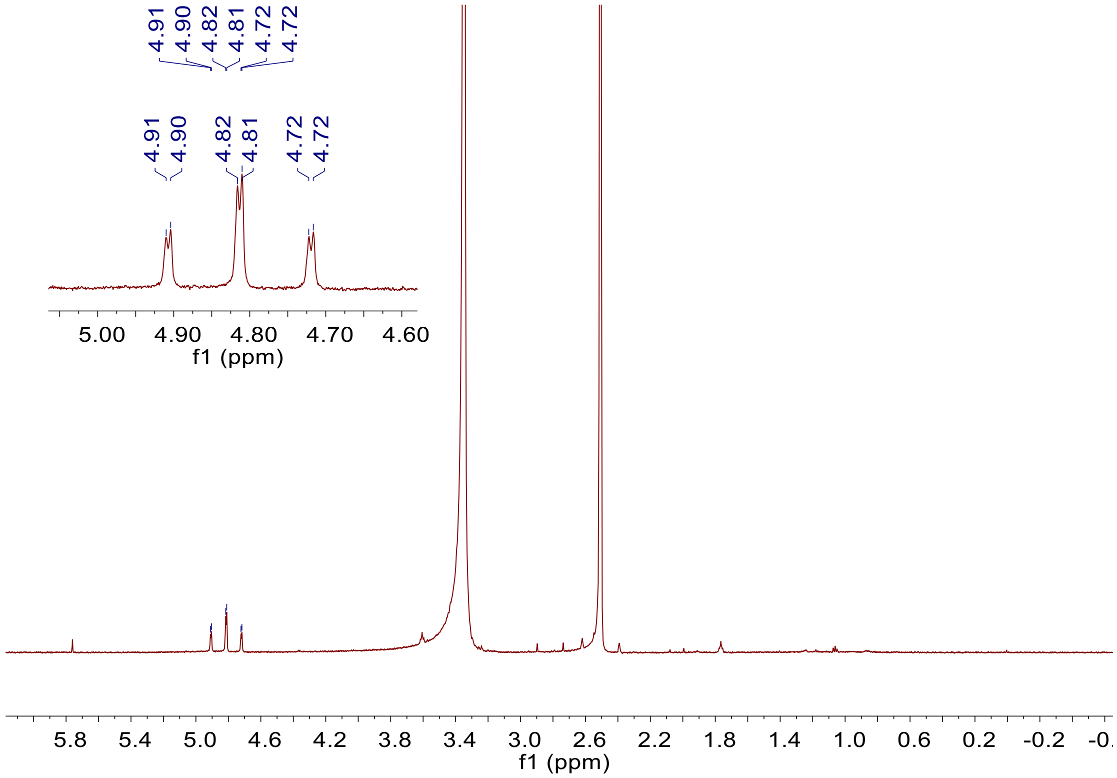

^{19}F NMR (565 MHz, DMSO-*d6*) spectrum of CF_2HSO_2Na

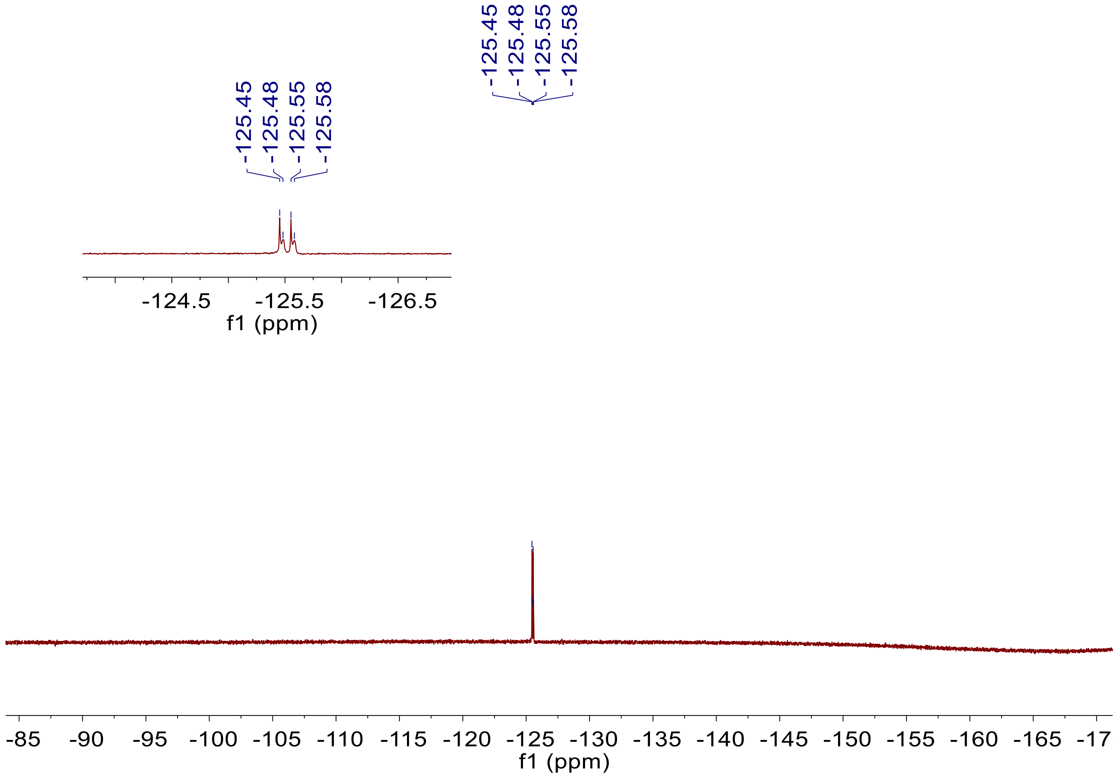

^{1}H NMR (600 MHz, DMSO-*d6*) spectrum of CF_2HSO_2Na + $ZnCl_2$

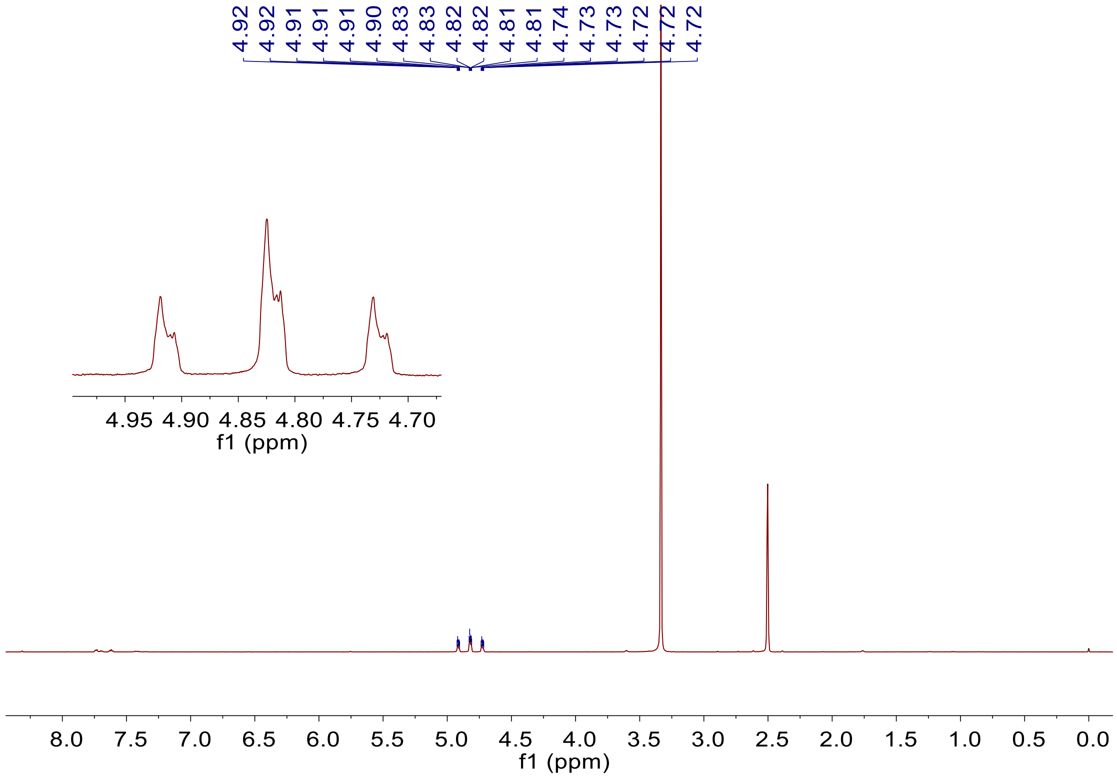

^{19}F NMR (565 MHz, DMSO-*d6*) spectrum of CF_2HSO_2Na + $ZnCl_2$

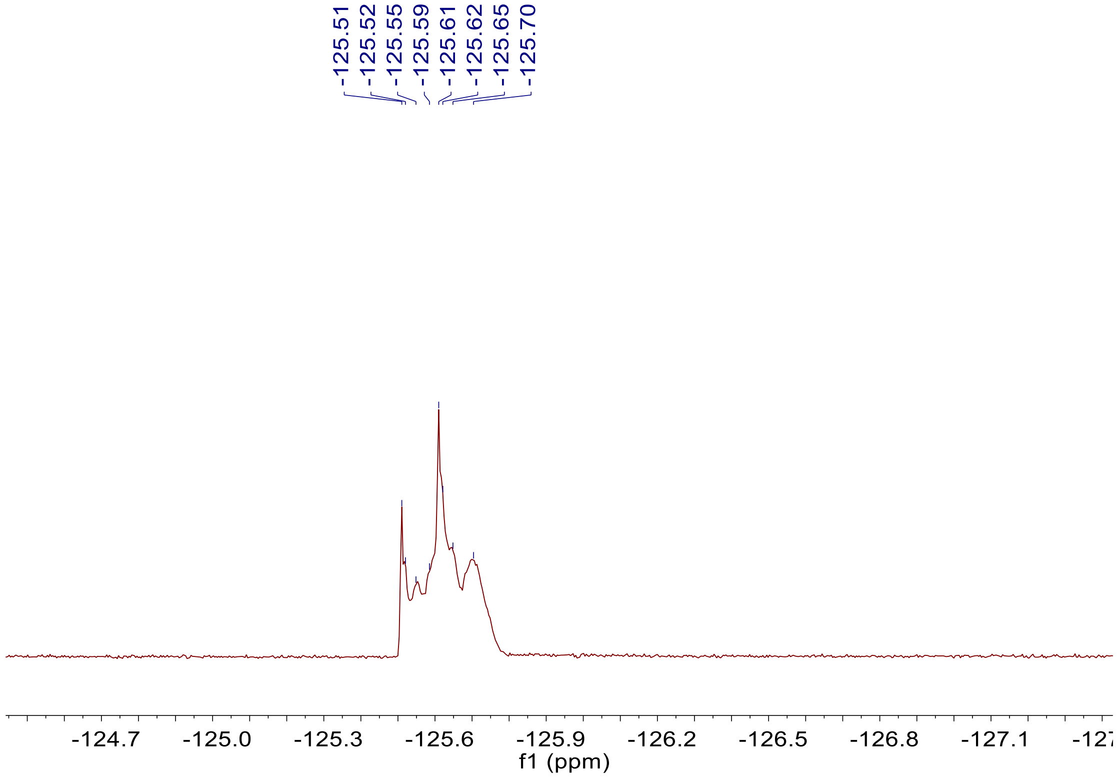

Further evaluation revealed that the presence of $ZnCl_2$ significantly increased the yield of the intermediate RSO_2M product compared to the $ZnCl_2$-free conditions.

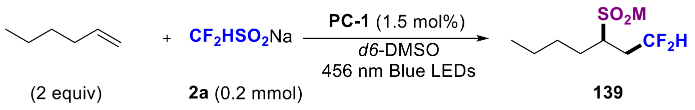

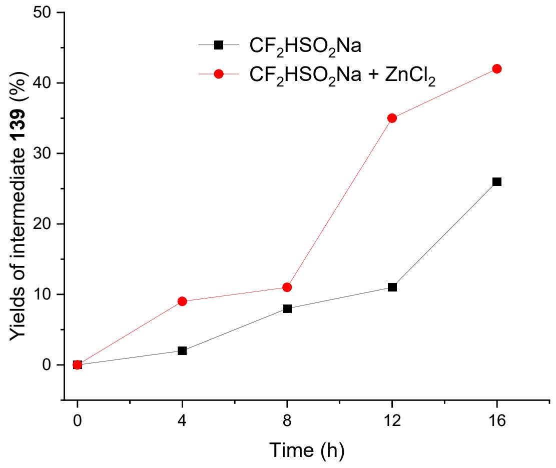

Figure **S14**. Reaction Monitoring

Time(h)	0	4	8	12	16
**2a**	0	2	8	11	26
**2a+ZnCl₂**	0	9	12	35	42

q) Determination of the enantiomeric and diastereomeric purity of product 117 by HPLC

To further rigorously confirm the stereoselectivity, a racemic sample of compound **117** was prepared via a one-pot, two-step procedure with *Rac*-3i (1:1 dr). Chiral HPLC separation of this sample revealed the presence of four stereoisomers. Under identical analytical conditions, our synthesized product **117**, obtained using chiral *R*-3i with standard **Conditions A**, exhibited a diastereomeric ratio (dr) of 15:1 and an enantiomeric excess (ee) of >99%.

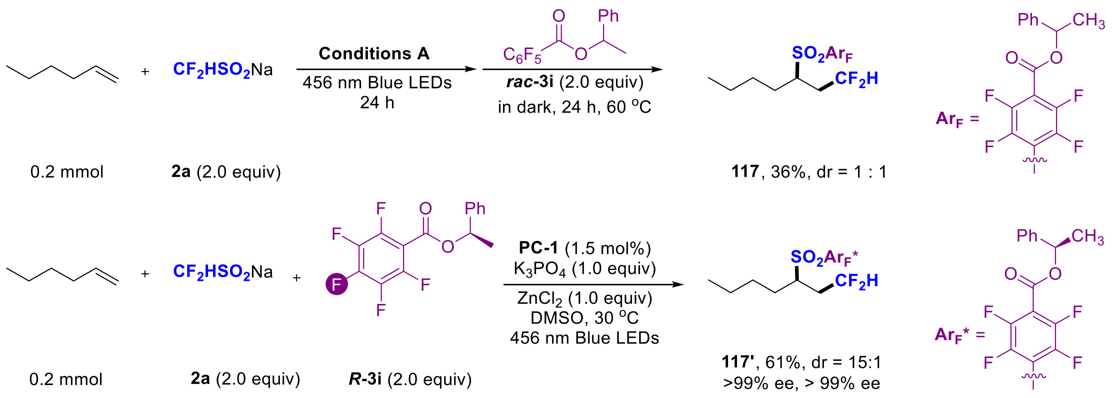

HPLC analysis: Daicel Chiralpak IA column (98:2 hexane: 2-propanol, 0.8 mL/min, 25 °C, 254 nm); t_R (major) = 13.077 min, t_R (minor) = 14.185 min.

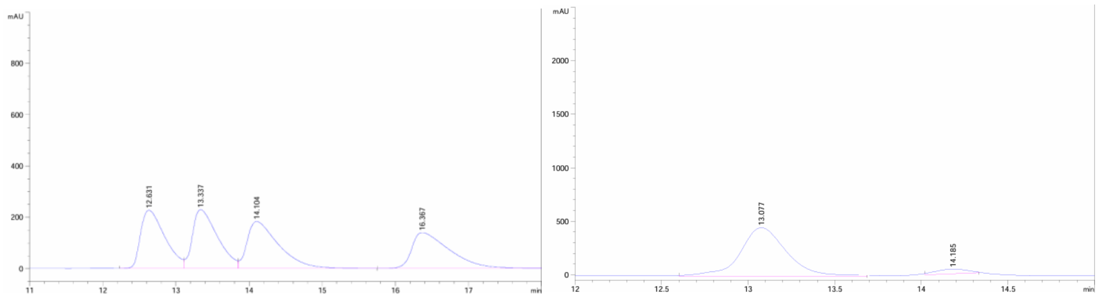

Peak	PetTime	Area	Area%	Peak	PetTime	Area	Area%
1	12.631	5195.07227	24.6628	1	13.077	8342.40625	93.9751
2	13.337	5426.33740	25.7607	2	14.185	534.84888	6.0249
3	14.104	5464.67578	25.9428				
4	16.367	5978.28027	24.6337				

Figure **S15.** Determination of the enantiomeric and diastereomeric purity of product **117** by HPLC

l) X-ray crystallographic data of 10 and 113.

A single crystal of **10** suitable for X-ray crystallography was obtained by crystallization via Evaporation from its DCM/ petroleum ether solution. And the crystal structure of compound **10** has been deposited at the Cambridge Crystallographic Data Centre (CCDC 2304760).

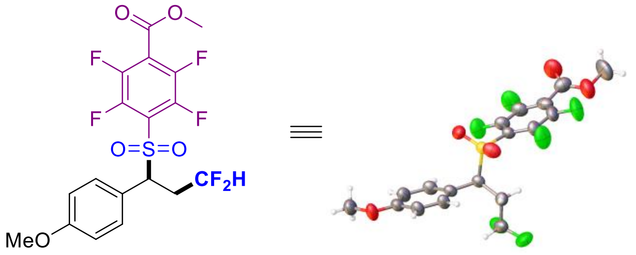

Figure **S16** Crystal data and structure refinement for CCDC 2304760.

A single crystal of **113** suitable for X-ray crystallography was obtained by crystallization via Evaporation from its DCM/ petroleum ether solution. And the crystal structure of compound **113** has been deposited at the Cambridge Crystallographic Data Centre (CCDC 2411985).

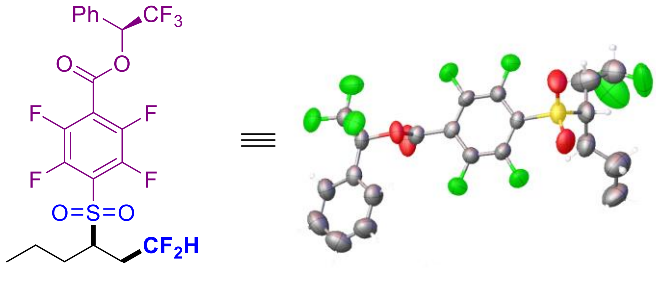

Figure **S17** Crystal data and structure refinement for CCDC 2411985.

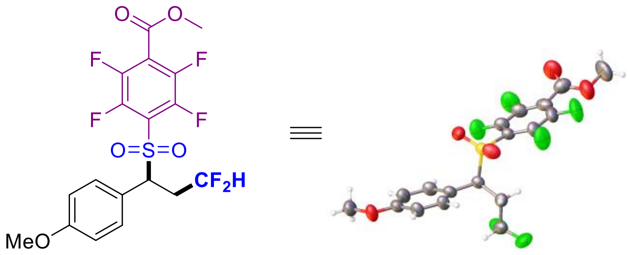

Identification code	xiongt230227_1_0m
Empirical formula	$C_{18}H_{14}F_6O_5S$
Formula weight	456.35
Temperature/K	292.86
Crystal system	triclinic
Space group	P-1
a/Å	9.2504(7)
b/Å	10.1808(7)
c/Å	10.2513(8)
α/°	89.565(4)
β/°	75.374(3)
γ/°	86.345(3)
Volume/Å^3	932.21(12)
Z	1
ρ_{calc}g/cm^3	0.813
μ/mm^{-1}	1.191
F(000)	232.0
Crystal size/mm^3	? × ? × ?
Radiation	CuKα (λ = 1.54184)
2Θ range for data collection/°	8.704 to 127.536
Index ranges	-10 ≤ h ≤ 10, -11 ≤ k ≤ 11, -11 ≤ l ≤ 11
Reflections collected	12067
Independent reflections	3048 [R_{int} = 0.0429, R_{sigma} = 0.0366]
Data/restraints/parameters	3048/0/273
Goodness-of-fit on F^2	1.033
Final R indexes [I>=2σ (I)]	R_1 = 0.0841, wR_2 = 0.2263
Final R indexes [all data]	R_1 = 0.0955, wR_2 = 0.2387
Largest diff. peak/hole / e Å^{-3}	1.25/-0.60

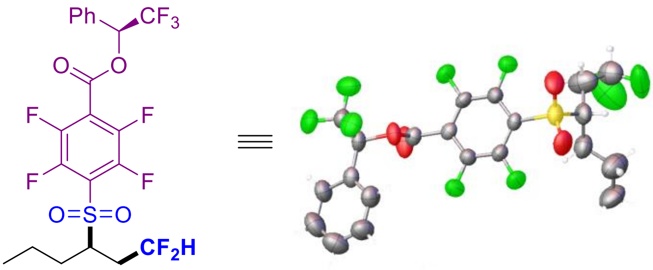

Identification code	cu_zhenggf240923_1
Empirical formula	$C_{22}H_{18}F_8O_4S$
Formula weight	530.42
Temperature/K	298.0
Crystal system	orthorhombic
Space group	$P2_12_12_1$
a/Å	5.3579(2)
b/Å	15.2973(5)
c/Å	27.0031(9)
α/°	90
β/°	90
γ/°	90
Volume/Å^3	2213.21(13)
Z	4
ρ_{calc}g/cm^3	1.592
μ/mm^{-1}	2.198
F(000)	1080.0
Crystal size/mm^3	0.22 × 0.2 × 0.2
Radiation	CuKα (λ = 1.54178)
2Θ range for data collection/°	6.546 to 127.552
Index ranges	-6 ≤ h ≤ 6, -17 ≤ k ≤ 15, -31 ≤ l ≤ 26
Reflections collected	21842
Independent reflections	3620 [R_{int} = 0.0715, R_{sigma} = 0.0565]
Data/restraints/parameters	3620/0/317
Goodness-of-fit on F^2	1.056
Final R indexes [I>=2σ (I)]	R_1 = 0.0555, wR_2 = 0.1442
Final R indexes [all data]	R_1 = 0.0654, wR_2 = 0.1505
Largest diff. peak/hole / e Å^{-3}	0.36/-0.32
Flack parameter	0.026(11)

DFT Calculations

Computational Details

All of the calculations were performed using Gaussian 16 program.[85] Geometry optimization were performed in the gas phase using M06-2X functional[86] including Grimme empirical dispersion correction (D3BJ)[87, 88] with a basis set of 6-31G(d)[89, 90] in gas phase. Frequency calculations have been performed to verify the optimized structures as local minima or transition state and to obtain Gibbs free energy at 298 K.[91] Intrinsic reaction coordinate (IRC) calculations were carried out to make sure that every transition state links relevant intermediates.[92, 93] The M06-2X/6-311+G(d,p) was used for the solution phase single-point energy calculations.[94] The SMD solvation model with DMSO as the solvent was employed to account for solvation effect.[95, 96] The three-dimensional (3D) structures were depicted using vmd software.[97]

Identification and quantification of Non-Covalent Interactions using AIM

The presence of a bond critical point between a pair of atoms is generally regarded as an indicator of interatomic non-covalent interaction in the AIM. An interacting pair of atoms is characterized by the presence of a bond path and a bond critical point (bcp) along the bond path. The shared electron density shows a minimum at the bcp. The value of the electron density electron densities (ρ), Laplacian of electron density ($\Delta 2\rho$), potential electron density (V) and Kinetic Energy density (K) at such bond critical points provides valuable information about the nature of such interaction and sometime correlates with the strength of such interaction. Summaries of AIM analyses of the stereocontrolling TS structures are presented below.

In general, relatively higher ρbcp values for a given type of interaction imply a stronger interaction. In the present case, ρbcp is employed as an approximate measure of the strength of individual interactions as well as for comparison of the NCIs between the competing transition states.

Table S4. Estimation of Noncovalent Interaction Energies Using Espinosa Equation (E_{NI} = 1/2V(r_{cp})) in the Enantiocontrolling transition state **TS1-maj**.

Bond Id	Noncovalent Interactions	Distance	a.u				E_{NI}
			ρ	Δ2ρ	V	K	
**1a**	Na···O	2.15	0.032	0.23	-0.038	-0.0097	-11.9
**1b**	Na···F	2.25	0.021	0.14	-0.029	-0.0055	-9.1
**1c**	Na···F	2.32	0.017	0.11	-0.022	-0.0044	-6.9
**1d**	π···π	3.33	0.0075	0.021	-0.0038	-0.00078	-1.2
	π···π	3.36	0.0069	0.019	-0.003	-0.00089	-0.9
**1e**	π···π	3.31	0.0057	0.021	-0.0031	-0.0011	-1.0
**1f**	π···π	3.34	0.0040	0.018	-0.0024	-0.00098	-0.8
**1g**	C−H···π	2.87	0.0062	0.023	-0.0033	-0.0012	-1.0
Total E_{NI}							-32.8

Table S5. Estimation of Noncovalent Interaction Energies Using Espinosa Equation (E_{NI} = 1/2V(r_{cp})) in the Enantiocontrolling transition state **TS1-min**.

Bond Id	Noncovalent Interactions	Distance	a.u				E_{NI}
			ρ	Δ2ρ	V	K	
**2a**	Na···O	2.15	0.031	0.22	-0.036	-0.0096	-11.3
**2b**	Na···F	2.26	0.020	0.14	-0.024	-0.0054	-7.5
**2c**	Na···F	2.33	0.016	0.11	-0.019	-0.0044	-6.0
**2d**	C−H···F	2.42	0.0088	0.038	-0.0070	-0.0013	-2.2
**2e**	π···π	3.25	0.0085	0.023	-0.0041	-0.00087	-1.3
**2f**	C−H···π	2.82	0.0063	0.021	-0.0032	-0.0010	-1.0
Total E_{NI}							-29.3

Table S6. Estimation of Noncovalent Interaction Energies Using Espinosa Equation (E_{NI} = 1/2V(r_{cp})) in the Enantiocontrolling transition state **TS2-maj**.

Bond Id	Noncovalent Interactions	Distance	a.u				E_{NI}
			ρ	Δ2ρ	V	K	
**3a**	Na···O	2.14	0.032	0.23	-0.037	-0.0099	-11.6
**3b**	Na···F	2.29	0.019	0.13	-0.023	-0.0049	-7.3
**3c**	Na···F	2.33	0.016	0.11	-0.019	-0.0043	-6.0
**3d**	C−H···F	2.39	0.0096	0.041	-0.0078	-0.0013	-2.4
**3e**	C−H···F	2.69	0.0088	0.028	-0.0047	-0.0011	-1.5
**3f**	C−H···F	2.68	0.0050	0.024	-0.0034	-0.0014	-1.1
**3g**	C−H···π	2.72	0.0068	0.026	-0.0037	-0.00144	-1.2
**3h**	C−H···π	2.96	0.0046	0.015	-0.0020	-0.00093	-0.6
Total E_{NI}							-31.6

Table S7. Estimation of Noncovalent Interaction Energies Using Espinosa Equation (E_{NI} = 1/2V(r_{cp})) in the Enantiocontrolling transition state **TS2-min**.

Bond Id	Noncovalent Interactions	Distance	a.u				E_{NI}
			ρ	Δ2ρ	V	K	
**4a**	Na···O	2.14	0.032	0.23	-0.038	-0.010	-11.9
**4b**	Na···F	2.25	0.021	0.14	-0.025	-0.0055	-7.8
**4c**	Na···F	2.37	0.015	0.10	-0.017	-0.0039	-5.3
**4d**	C−H···F	2.63	0.0048	0.024	-0.0032	-0.0014	-1.0
**4e**	C−H···F	2.68	0.0094	0.032	-0.0049	-0.0015	-1.5
**4f**	C−H···π	2.89	0.0047	0.016	-0.0022	-0.00095	-0.7
**4g**	C−H···π	2.86	0.0058	0.018	-0.0027	-0.00093	-0.8
Total E_{NI}							-29.0

Cartesian Coordinates of reported structures.
TS1-maj

Zero-point correction= 0.365960 (Hartree/Particle)
Thermal correction to Energy= 0.401686
Thermal correction to Enthalpy= 0.402630
Thermal correction to Gibbs Free Energy= 0.296355
Sum of electronic and zero-point Energies= -2782.103287
Sum of electronic and thermal Energies= -2782.067561
Sum of electronic and thermal Enthalpies= -2782.066617
Sum of electronic and thermal Free Energies= -2782.172893

C	0.40161400	-1.24240700	0.90718700
C	-0.83435500	-1.49820700	1.44118700
C	-1.97734400	-1.70972700	0.61868500
C	-1.66531800	-1.84558300	-0.75938000
C	-0.43065100	-1.57548300	-1.29334500
C	0.67322500	-1.28641700	-0.47867700
C	1.96261500	-0.93429400	-1.08126600
O	2.12881900	-0.54656700	-2.21260600
O	2.98921200	-1.10002600	-0.20398700
C	4.22739200	-0.53262900	-0.60334400
F	-0.31611200	-1.62612500	-2.61522200
F	1.35219800	-0.90601700	1.77602800
F	-1.04224000	-1.35857100	2.75814700
F	-2.73943600	-2.09129600	-1.59115900
F	-2.86415300	-2.76186500	1.08213500
S	-3.44220100	-0.26422000	0.89974400
O	-4.74127900	-0.83974900	0.39783600
O	-3.47755100	0.36302300	2.22823900
C	-3.03406300	1.03856900	-0.32128700
H	-3.29226800	0.58443200	-1.28353500
H	4.33388900	-0.58652500	-1.69012200
Na	-4.54437500	-2.82780300	-0.37957600
C	5.29529700	-1.43111700	-0.00095200
C	4.34037400	0.89216600	-0.11851400
C	3.92044900	1.21611200	1.17379800
C	4.85311200	1.88268900	-0.95231400
C	4.02198400	2.52751400	1.62613100
H	3.49676200	0.44221600	1.80552000
C	4.94890600	3.19698800	-0.49943100
H	5.16725000	1.62707400	-1.96078800
C	4.53438500	3.51959800	0.79041800
H	3.69739700	2.77789900	2.63167200

H	5.34189600	3.96764900	-1.15499000
H	4.60677500	4.54362500	1.14353300
F	6.50867700	-0.96398000	-0.32057800
F	5.19453300	-2.67705200	-0.47391700
F	5.21226000	-1.49152100	1.33109700
C	-3.95088300	2.23903400	-0.05705000
H	-3.67410400	3.02518000	-0.76766100
H	-3.78413900	2.62108100	0.95463300
C	-5.42766500	1.96258800	-0.24959800
H	-5.87591800	1.31810300	0.50794400
F	-5.62341400	1.38389500	-1.46996600
C	-1.57612300	1.44047300	-0.31292100
C	-0.90393400	1.53167000	-1.53516500
C	-0.90667100	1.78747300	0.86442200
C	0.41457100	1.97110100	-1.58836600
H	-1.41635600	1.24650400	-2.45167700
C	0.42156100	2.20623200	0.80954500
H	-1.41654200	1.70807300	1.82168100
C	1.08131500	2.31016500	-0.41349400
H	0.93039600	2.01716200	-2.54135400
H	0.94566600	2.45449600	1.72768900
H	2.11280100	2.64853400	-0.44951400
F	-6.08703800	3.15090400	-0.25290500

TS1-min

Zero-point correction= 0.366093 (Hartree/Particle)
Thermal correction to Energy= 0.401858
Thermal correction to Enthalpy= 0.402803
Thermal correction to Gibbs Free Energy= 0.296347
Sum of electronic and zero-point Energies= -2782.097531
Sum of electronic and thermal Energies= -2782.061765
Sum of electronic and thermal Enthalpies= -2782.060821
Sum of electronic and thermal Free Energies= -2782.167277

C	0.30476400	-0.80323400	1.26805000
C	-0.89883300	-0.70715600	1.92054000
C	-2.11066300	-1.13995600	1.32015300
C	-1.92682200	-1.86207000	0.11714900
C	-0.72757000	-1.94741900	-0.54430100
C	0.45293000	-1.43799000	0.01300400
C	1.66232000	-1.35121700	-0.81493500
O	1.68826100	-1.44834100	-2.01806500
O	2.76534600	-1.10340600	-0.06508700
C	3.93122900	-0.68251400	-0.75812200

F	-0.73005600	-2.55930900	-1.72266700
F	1.34190500	-0.23704100	1.87722300
F	-0.98943400	-0.02815100	3.06968400
F	-3.05876200	-2.39602200	-0.45604400
F	-3.04110900	-1.78399600	2.20443400
S	-3.51096000	0.47577700	1.07153600
O	-4.80848200	-0.17842600	0.64429700
O	-3.61582100	1.42873300	2.18644700
C	-2.95355900	1.43961700	-0.40583300
H	-3.51509800	2.37153200	-0.25768000
H	3.74555300	-0.68157000	-1.83578400
Na	-4.84131900	-2.29276600	0.96847300
C	4.99252900	-1.74001300	-0.49684200
C	4.34058700	0.68154800	-0.26264900
C	4.76233200	1.65707800	-1.16313900
C	4.28506200	0.97017900	1.10297000
C	5.12549000	2.92201200	-0.70479600
H	4.80040700	1.42945800	-2.22543600
C	4.64955700	2.23193700	1.55877100
H	3.93273000	0.21148200	1.79357600
C	5.06940500	3.20939700	0.65631600
H	5.44628600	3.68129100	-1.41086300
H	4.60060200	2.45648400	2.61961400
H	5.34872900	4.19542900	1.01447600
F	6.12747300	-1.41282800	-1.12621500
F	4.59275700	-2.93228600	-0.94899800
F	5.26281200	-1.86710600	0.80615400
C	-3.41182500	0.78014900	-1.70615600
H	-3.27964600	-0.30641800	-1.69296400
H	-2.82327400	1.17803000	-2.53846000
C	-4.85296300	1.09516700	-2.04605000
H	-5.57569100	0.79442900	-1.28723000
F	-4.97386100	2.43742400	-2.24498600
C	-1.47001800	1.74341000	-0.29197900
C	-1.01604700	2.50851300	0.79398100
C	-0.54212900	1.30646600	-1.23944200
C	0.33064400	2.82156400	0.92281300
H	-1.72721400	2.84504100	1.54267000
C	0.80634900	1.64902400	-1.12043900
H	-0.84658800	0.68713800	-2.07838400
C	1.24887000	2.40296600	-0.04097300
H	0.66515600	3.40525800	1.77465100
H	1.51010500	1.30599700	-1.87423500
H	2.29894300	2.66519700	0.04880100

F	-5.16454600	0.47886700	-3.21856600

TS2-maj

Zero-point correction= 0.370338 (Hartree/Particle)
Thermal correction to Energy= 0.405755
Thermal correction to Enthalpy= 0.406699
Thermal correction to Gibbs Free Energy= 0.300236
Sum of electronic and zero-point Energies= -2669.013630
Sum of electronic and thermal Energies= -2668.978212
Sum of electronic and thermal Enthalpies= -2668.977268
Sum of electronic and thermal Free Energies= -2669.083731

C	0.26638400	-1.04069300	1.11165100
C	-0.95335200	-1.09981300	1.73880900
C	-2.14934600	-1.33110600	1.01496100
C	-1.94209000	-1.75682300	-0.31644200
C	-0.72264900	-1.71408300	-0.94814000
C	0.44686500	-1.34529900	-0.25894400
C	1.72170400	-1.23869400	-0.99057600
O	1.90493300	-1.58702600	-2.12881200
O	2.67701000	-0.64563700	-0.22821900
C	3.94606800	-0.43458600	-0.82808900
F	-0.70130900	-2.03687300	-2.23334900
F	1.29157600	-0.68992500	1.88378700
F	-1.06568800	-0.75711100	3.02660300
F	-3.06878200	-2.09766400	-1.02514100
F	-3.14220200	-2.08350900	1.68971400
S	-3.49747400	0.42980600	1.11121100
O	-4.82553100	-0.06606600	0.56259700
O	-3.56029700	1.13232800	2.40333000
C	-2.92099900	1.64946500	-0.13200400
H	-3.42723200	2.56548200	0.20028300
H	3.86328200	-0.50234700	-1.91636600
Na	-4.93579500	-2.17830800	0.32283000
C	4.84578400	-1.57930000	-0.37620300
C	4.48992200	0.90038600	-0.38551100
C	5.37083100	1.59610100	-1.21103600
C	4.14746800	1.41905300	0.86379400
C	5.89986100	2.81498400	-0.79644900
H	5.64118500	1.18372900	-2.17958900
C	4.66938500	2.64266100	1.27086700
H	3.46365300	0.86706900	1.50120200
C	5.54587300	3.34114700	0.44303600
H	6.58227700	3.35564200	-1.44453600

H	4.39357600	3.04940700	2.23888000
H	5.95215900	4.29539200	0.76370600
F	6.03955200	-1.48679800	-0.97540100
F	4.30962000	-2.76209300	-0.69441300
F	5.04013900	-1.56277400	0.94476300
C	-3.38837900	1.26015100	-1.53639900
H	-3.34056900	0.17836300	-1.69544100
H	-2.73042100	1.72753900	-2.27810300
C	-4.78994500	1.73483400	-1.84463700
H	-5.54434800	1.38119700	-1.13995300
C	-1.40210900	1.84624500	-0.07177400
C	-0.79526800	2.23304500	1.27747100
H	-1.17098800	2.63892600	-0.79561400
H	-0.90601500	0.94499100	-0.45604100
C	0.71039200	2.45226500	1.13348500
H	-1.28322900	3.14169000	1.64992500
H	-0.98564400	1.46507000	2.03361900
H	1.17688500	2.66055000	2.10060500
H	1.19923300	1.56574600	0.71298300
H	0.92778300	3.29273200	0.46571100
F	-4.80560100	3.09707300	-1.84287200
F	-5.13126000	1.32466100	-3.09704200

TS2-maj

Zero-point correction=	0.370152 (Hartree/Particle)
Thermal correction to Energy=	0.405666
Thermal correction to Enthalpy=	0.406610
Thermal correction to Gibbs Free Energy=	0.299336
Sum of electronic and zero-point Energies=	-2669.009796
Sum of electronic and thermal Energies=	-2668.974282
Sum of electronic and thermal Enthalpies=	-2668.973337
Sum of electronic and thermal Free Energies=	-2669.080612

C	0.35311100	-1.04133700	1.03095800
C	-0.91191500	-1.14497500	1.55570700
C	-2.00080600	-1.59339800	0.77121600
C	-1.63609200	-2.15482300	-0.47148800
C	-0.36837700	-2.07001700	-0.99535700
C	0.68953300	-1.49586500	-0.26635500
C	2.01479100	-1.35364500	-0.89601300
O	2.33711000	-1.81982700	-1.95869800
O	2.83610700	-0.57822600	-0.14133400
C	4.13317800	-0.31625200	-0.65474200

F	-0.18762700	-2.55829400	-2.21440900
F	1.26508200	-0.49691900	1.83266500
F	-1.17765600	-0.66479200	2.77415700
F	-2.64629000	-2.71784900	-1.20567800
F	-2.99179000	-2.33348900	1.44726200
S	-3.53584100	0.04138300	0.51830200
O	-4.71602800	-0.65041700	-0.14315600
O	-3.82657600	0.83752400	1.72544300
C	-2.96622000	1.20608200	-0.77959500
H	-3.14108100	0.65429000	-1.71116400
H	4.16358600	-0.53042400	-1.72663000
Na	-4.63926500	-2.76385700	-0.05358600
C	5.08417000	-1.29549600	0.02403400
C	4.50838300	1.11287600	-0.35357500
C	3.99716000	1.74904100	0.77824900
C	5.40219800	1.78353200	-1.18594500
C	4.36409800	3.06136500	1.05841900
H	3.30497500	1.21578500	1.42254700
C	5.77585800	3.09288400	-0.89703700
H	5.80450600	1.28072000	-2.06150800
C	5.25343600	3.73412700	0.22298900
H	3.95739200	3.55798800	1.93395600
H	6.46913400	3.61300900	-1.55039300
H	5.53863500	4.75783800	0.44471900
F	6.31625300	-1.16218200	-0.48271100
F	4.68786300	-2.55768700	-0.16946100
F	5.15048400	-1.08687500	1.34113800
C	-3.82698700	2.46835200	-0.74539500
H	-3.38873300	3.18559000	-1.44868400
H	-3.81698100	2.91493300	0.25405800
C	-5.27636900	2.27270700	-1.14695300
H	-5.41256500	1.55299200	-1.96220900
C	-1.46844400	1.50739400	-0.65194900
C	-0.99855100	2.14282300	0.65852600
H	-1.20930700	2.17665700	-1.48388800
H	-0.89917800	0.58667200	-0.82606200
C	0.50176100	2.42846500	0.60077700
H	-1.54855100	3.07263300	0.84524000
H	-1.22253400	1.48678300	1.50729400
H	1.06886000	1.52402100	0.35318300
H	0.73271000	3.17689700	-0.16486600
H	0.87423200	2.80136600	1.55925500
F	-6.01999100	1.86475300	-0.09331500
F	-5.78056200	3.47301000	-1.55021600

Characterization data of new compounds

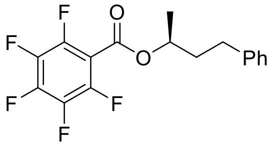

(S)-4-phenylbutan-2-yl 2,3,4,5,6-pentafluorobenzoate (3g)

This compound was obtained as a colorless oil by the general procedure.

^{1}H NMR (600 MHz, Chloroform-*d*) δ 7.28 (t, J = 7.6 Hz, 2H), 7.19 (td, J = 7.5, 6.9, 1.5 Hz, 3H), 5.27 – 5.19 (m, 1H), 2.76 (ddd, J = 13.9, 10.1, 5.5 Hz, 1H), 2.68 (ddd, J = 13.8, 9.9, 6.5 Hz, 1H), 2.11 – 2.01 (m, 1H), 1.98 – 1.88 (m, 1H), 1.40 (d, J = 6.3 Hz, 3H).

^{13}C NMR (151 MHz, Chloroform-*d*) δ 158.65, 146.20 – 145.90 (m), 144.66 – 144.19 (m), 144.05 – 143.71 (m), 142.49 – 142.01 (m), 141.08, 138.62 – 138.29 (m), 137.14 – 136.66 (m), 128.53, 128.34, 126.10, 74.02, 37.48, 31.59, 19.94.

^{19}F NMR (565 MHz, Chloroform-*d*) δ -138.69 – -138.78 (m), -149.22 – -149.36 (m), -160.39 – -160.53 (m).

HRMS (ESI) (m/z): calcd for $C_{17}H_{13}F_5NaO_2$ ([M + Na]$^+$), 367.0728; found, 367.0734.

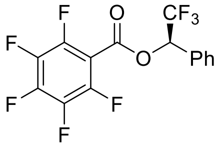

(R)-2,2,2-trifluoro-1-phenylethyl 2,3,4,5,6-pentafluorobenzoate (3h)

This compound was obtained as a colorless oil by the general procedure.

^{1}H NMR (600 MHz, Chloroform-*d*) δ 7.55 – 7.52 (m, 2H), 7.49 – 7.42 (m, 3H), 6.35 (q, J = 6.6 Hz, 1H).

^{13}C NMR (151 MHz, Chloroform-*d*) δ 157.11, 147.05 – 146.68 (m), 145.27 – 145.00 (m), 143.33 – 143.02 (m), 138.90 – 138.50 (m), 137.22 – 136.86 (m), 130.41, 130.00, 128.92, 128.23, 122.72 (q, J = 280.7 Hz), 73.83 (q, J = 33.5 Hz).

^{19}F NMR (565 MHz, Chloroform-*d*) δ -75.94 (d, J = 6.2 Hz), -136.41 – -136.53 (m), -146.14 (tt, J = 21.6, 5.8 Hz), -159.56 – -159.71 (m).

HRMS (ESI) (m/z): calcd for $C_{15}H_6F_8NaO_2$ ([M + Na]$^+$), 393.0132; found, 393.0123.

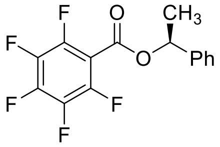

(S)-1-phenylethyl 2,3,4,5,6-pentafluorobenzoate (3i)

This compound was obtained as a colorless oil by the general procedure.

^{1}H NMR (600 MHz, Chloroform-*d*) δ 7.42 (d, J = 6.9 Hz, 2H), 7.38 (t, J = 7.4 Hz, 2H), 7.35 – 7.31 (m, 1H), 6.15 (q, J = 6.6 Hz, 1H), 1.68 (d, J = 6.6 Hz, 3H).

^{13}C NMR (151 MHz, Chloroform-*d*) δ 158.29, 146.63 – 146.07 (m), 144.84 – 144.37 (m), 144.32 – 143.72 (m), 142.65 – 142.07 (m), 140.29, 138.72 – 138.30 (m), 137.16 – 136.56 (m), 128.69, 128.41, 126.22, 75.56, 22.17.

^{19}F NMR (565 MHz, Chloroform-*d*) δ -138.16 – -138.27 (m), -148.77 (t, J = 20.9 Hz), -160.37 – -160.50 (m).

HRMS (ESI) (m/z): calcd for $C_{15}H_9F_5NaO_2$ ([M + Na]$^+$), 339.0415; found, 339.0407.

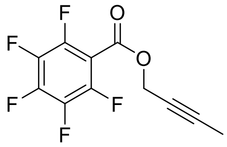

but-2-yn-1-yl 2,3,4,5,6-pentafluorobenzoate (3j)
This compound was obtained as a colorless oil by the general procedure.
^{1}H NMR (600 MHz, Chloroform-*d*) δ 4.94 (q, J = 2.4 Hz, 1H), 1.89 (t, J = 2.4 Hz, 1H).
^{13}C NMR (151 MHz, Chloroform-*d*) δ 158.51, 146.48 – 146.09 (m), 144.87 – 144.47 (m), 144.41 – 144.06 (m), 142.72 – 142.35 (m), 138.54 (ddd, J = 17.8, 12.7, 5.7 Hz), 137.05 – 136.68 (m), 107.99 – 107.58 (m), 84.67, 71.85, 54.92, 3.61.
^{19}F NMR (565 MHz, Chloroform-*d*) δ -137.55 – -137.71 (m), -148.09 (t, J = 21.1 Hz), -160.21 – -160.47 (m).
HRMS (ESI) (m/z): calcd for $C_{11}H_5F_5NaO_2$ ([M + Na]$^+$), 287.0102; found, 287.0113.

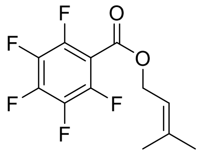

3-methylbut-2-en-1-yl 2,3,4,5,6-pentafluorobenzoate (3k)
This compound was obtained as a colorless oil by the general procedure.
^{1}H NMR (600 MHz, Chloroform-*d*) δ 5.44 (tdt, J = 7.4, 2.9, 1.5 Hz, 1H), 4.87 (d, J = 7.3 Hz, 2H), 1.80 (s, 3H), 1.77 (s, 3H).
^{13}C NMR (151 MHz, Chloroform-*d*) δ 159.10, 146.34 – 145.84 (m), 144.82 – 144.26 (m), 144.14 – 143.68 (m), 142.76 – 141.87 (m), 141.25, 138.93 – 138.18 (m), 137.23 – 136.44 (m), 117.31, 108.82 – 108.43 (m), 63.66, 25.80, 18.12.
^{19}F NMR (565 MHz, Chloroform-*d*) δ -138.37 – -138.50 (m), -149.13 (t, J = 20.7 Hz), -160.50 – -160.66 (m).
HRMS (ESI) (m/z): calcd for $C_{12}H_9F_5NaO_2$ ([M + Na]$^+$), 303.0415; found, 303.0426.

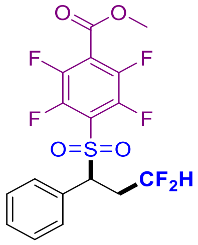

methyl-4-((3,3-difluoro-1-phenylpropyl)sulfonyl)-2,3,5,6-tetrafluorobenzoate (4)
This compound was obtained in 86% (73.3 mg) yield as white solid by the general procedure.
^{1}H NMR (500 MHz, $CDCl_3$) δ 7.39 – 7.29 (m, 5H), 5.78 (tdd, J = 56.0, 5.5, 3.5 Hz, 1H), 4.59 (dd, J = 10.0, 5.0 Hz, 1H), 3.98 (s, 3H), 3.02 – 2.90 (m, 1H), 2.85 – 2.71 (m, 1H).
^{13}C NMR (151 MHz, $CDCl_3$) δ 158.56, 144.54 (dm, J = 264.3 Hz),130.51, 129.73, 129.21, 119.20 (t, J = 15.1 Hz), 117.90 (t, J = 16.6 Hz), 114.44 (t, J = 240.1 Hz), 67.21 (t, J = 6.0 Hz), 53.91, 32.41 (t, J = 24.0 Hz).

^{19}F NMR (565 MHz, $CDCl_3$) δ -115.90 – -117.38 (m), -133.93 – -134.05 (m), -135.94 – -136.14 (m).

HRMS (ESI) (m/z): calcd for $C_{17}H_{12}F_6NaO_4S$ ([M + Na]$^+$): 449.0253; found: 449.0242.

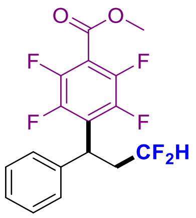

methyl (S)-4-(3,3-difluoro-1-phenylpropyl)-2,3,5,6-tetrafluorobenzoate (5)

This compound was obtained in 68% (49.2 mg) yield as yellow oil by the general procedure.

^{1}H NMR (600 MHz, $CDCl_3$) δ 7.38 – 7.31 (m, 4H), 7.31 – 7.26 (m, 1H), 5.78 (tt, J = 56.1, 4.5 Hz, 1H), 4.71 (t, J = 8.0 Hz, 1H), 3.96 (s, 3H), 2.81 (tdd, J = 16.6, 7.8, 4.5 Hz, 2H).

^{13}C NMR (151 MHz, $CDCl_3$) δ 160.02, 145.99 – 145.24 (m), 144.64 – 143.52 (m), 138.96, 129.18, 127.90, 127.46, 124.78 (t, J = 15.4 Hz), 115.69 (t, J = 240.1 Hz), 111.30 (t, J = 15.8 Hz), 53.29, 36.58 (t, J = 21.8 Hz), 35.47 (t, J = 6.2 Hz).

^{19}F NMR (565 MHz, $CDCl_3$) δ-115.28 – -118.64 (m), -138.71 – -138.96 (m), -140.88 – -141.23 (m).

HRMS (ESI) (m/z): calcd for $C_{17}H_{12}F_6NaO_2$ ([M + Na]$^+$): 385.0634; found: 385.0644.

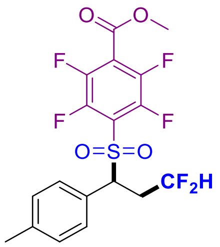

methyl-4-((3,3-difluoro-1-(p-tolyl)propyl)sulfonyl)-2,3,5,6-tetrafluorobenzoate (6)

This compound was obtained in 87% (76.6 mg) yield as white solid by the general procedure.

^{1}H NMR (500 MHz, $CDCl_3$) δ 7.18 (d, J = 10.0 Hz, 2H), 7.13 (d, J = 5.0 Hz, 2H), 5.74 (tdd, J = 56.0 ,5.5 3.5 Hz, 1H), 4.55 (dd, J = 10.0, 5.0 Hz, 1H), 3.97 (s, 3H), 2.98 – 2.85 (m, 1H), 2.80 – 2.68 (m, 1H), 2.31 (s, 3H).

^{13}C NMR (151 MHz, $CDCl_3$) δ 158.63, 144.40 (dm, J = 262.7 Hz), 140.73, 130.15, 129.09, 126.19, 119.38 (t, J = 14.6 Hz), 117.78 (t, J = 16.8 Hz), 114.54 (t, J = 241.4 Hz), 66.96 (t, J = 5.4 Hz), 53.88, 32.41 (t, J = 24.0 Hz), 21.18.

^{19}F NMR (565 MHz, $CDCl_3$) δ -116.58 – -116.97 (m), -134.03 – -134.18 (m), -136.07 – -136.20 (m).

HRMS (ESI) (m/z): calcd for $C_{18}H_{14}F_6NaO_4S$ ([M + Na]$^+$), 463.0409; found, 463.0418.

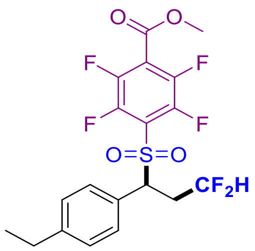

methyl-4-((1-(4-ethylphenyl)-3,3-difluoropropyl)sulfonyl)-2,3,5,6-tetra-fluoro-benzoate (7)

This compound was obtained in 60% (54.5 mg) yield as white solid by the general procedure.

^{1}H NMR (500 MHz, $CDCl_3$) δ 7.20 (d, J = 5.0 Hz, 2H), 7.14 (d, J = 5.0 Hz, 2H), 5.76 (tdd, J = 55.9, 6.0, 3.5 Hz, 1H), 4.55 (dd, J = 10.0, 5.0 Hz, 1H), 3.97 (s, 3H), 2.99 – 2.85 (m, 1H), 2.84 – 2.67 (m, 1H), 2.60 (q, J = 10.0 Hz, 2H), 1.17 (t, J = 10.0 Hz, 3H).

^{13}C NMR (151 MHz, $CDCl_3$) δ 158.61, 147.03, 144.38 (dm, J = 261.8 Hz), 129.18, 128.93, 126.38, 119.34 (t, J = 14.5 Hz), 117.79 (t, J = 16.7 Hz), 114.52 (t, J = 241.4 Hz), 67.09 (t, J = 5.2 Hz), 53.89, 32.40 (t, J = 24.1 Hz), 28.53, 15.19.

^{19}F NMR (565 MHz, $CDCl_3$) δ -116.06 – -117.40 (m), -133.89 – -134.05 (m), -136.19 – -136.39 (m).

HRMS (ESI) (m/z): calcd for $C_{19}H_{16}F_6NaO_4S$ ($[M + Na]^+$), 477.0566; found, 477.0570.

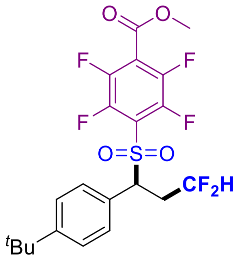

methyl-4-((1-(4-(tert-butyl)phenyl)-3,3-difluoropropyl)sulfonyl)-2,3,5,6-tetra-fluorobenzoate (8)

This compound was obtained in 74% (71.4 mg) yield as white solid by the general procedure.

^{1}H NMR (500 MHz, $CDCl_3$) δ 7.33 (d, J = 5.0 Hz, 2H), 7.20 (d, J = 10.0 Hz, 2H), 5.79 (tdd, J = 56.0, 6.0, 3.5 Hz, 1H), 4.55 (dd, J = 10.5, 5.0 Hz, 1H), 3.97 (s, 3H), 2.98 – 2.87 (m, 1H), 2.84 – 2.72 (m, 1H), 1.26 (s, 9H).

^{13}C NMR (151 MHz, $CDCl_3$) δ 158.57, 153.96, 144.30 (dm, J = 262.3 Hz), 128.96, 126.31, 126.12, 119.23 (t, J = 14.4 Hz), 117.82 (t, J = 17.2 Hz), 114.52 (t, J = 241.4 Hz), 67.15 (t, J = 5.8 Hz), 53.89, 34.77, 32.27 (t, J = 24.0 Hz), 31.00.

^{19}F NMR (565 MHz, $CDCl_3$) δ -116.46 – -116.85 (m), -133.72 – -133.97 (m), -136.47 – -136.84 (m).

HRMS (ESI) (m/z): calcd for $C_{21}H_{20}F_6NaO_4S$ ($[M + Na]^+$), 505.0878; found, 505.0890.

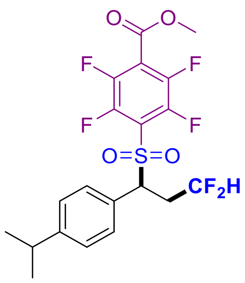

methyl-4-((3,3-difluoro-1-(4-isopropylphenyl)propyl)sulfonyl)-2,3,5,6-tetrafluoro-benzoate (9)

This compound was obtained in 71% (66.5 mg) yield as white solid by the general procedure.

^{1}H NMR (500 MHz, $CDCl_3$) δ 7.27 – 7.18 (m, 2H), 7.14 – 7.10 (m, 1H), 7.08 (s, 1H), 5.81 (tdd, J = 56.0, 6.0, 3.5 Hz, 1H), 4.57 (dd, J = 10.0, 5.0 Hz, 1H), 3.97 (s, 3H), 3.03 – 2.93 (m, 1H), 2.86 – 2.72 (m, 2H), 1.14 (t, J = 6.5 Hz, 6H).

^{13}C NMR (151 MHz, $CDCl_3$) δ 158.50, 150.42, 144.31 (dm, J = 243.3 Hz), 129.57, 129.45, 128.64, 127.17, 126.45, 119.20 (d, J = 14.5 Hz), 117.84 (d, J = 17.0 Hz), 114.52 (t, J = 241.5 Hz), 67.51 (t, J = 5.8 Hz), 53.85, 33.93, 32.26 (t, J = 24.0 Hz), 23.69.

^{19}F NMR (565 MHz, $CDCl_3$) δ -115.77 – -117.29 (m), -133.83 – -133.99 (m), -136.47 – -136.57 (m).

HRMS (ESI) (m/z): calcd for $C_{20}H_{18}F_6NaO_4S$ ([M + Na]$^+$), 491.0728; found, 491.0725.

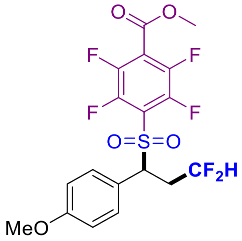

methyl-4-((3,3-difluoro-1-(4-methoxyphenyl)propyl)sulfonyl)-2,3,5,6-tetrafluoro-benzoate (10)

This compound was obtained in 90% (82.1 mg) yield as white solid by the general procedure.

^{1}H NMR (500 MHz, $CDCl_3$) δ 7.22 (d, J = 5.0 Hz, 2H), 6.83 (d, J = 5.0 Hz, 2H), 5.75 (tdd, J = 56.0, 3.5 Hz, 1H), 4.54 (dd, J = 15.0, 4.5 Hz, 1H), 3.98 (s, 3H), 3.78 (s, 3H), 2.96 – 2.85 (m, 1H), 2.80 – 2.64 (m, 1H).

^{13}C NMR (151 MHz, $CDCl_3$) δ 161.06, 158.62, 144.39, (dm, J = 261.2 Hz), 130.55, 120.81, 119.46 (t, J = 15.1 Hz), 117.79 (t, J = 16.6 Hz), 114.87, 114.53 (t, J = 241.5 Hz), 66.65 (t, J = 4.5Hz), 55.36, 53.89, 32.46 (t, J = 23.0 Hz).

^{19}F NMR (565 MHz, $CDCl_3$) δ -116.11 – -117.47 (m), -133.98 – -134.18 (m), -135.95 – -136.23 (m).

HRMS (ESI) (m/z): calcd for $C_{18}H_{14}F_6NaO_5S$ ([M + Na]$^+$), 479.3354; found, 479.3361.

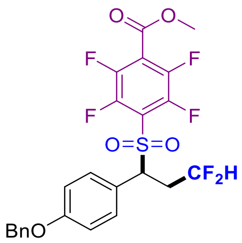

methyl-4-((1-(4-(benzyloxy)phenyl)-3,3-difluoropropyl)sulfonyl)-2,3,5,6-tetra-fluorobenzoate (11)

This compound was obtained in 51% (54.3 mg) yield as white solid by the general procedure.

^{1}H NMR (500 MHz, $CDCl_3$) δ 7.42 – 7.31 (m, 5H), 7.23 (d, J = 5.0 Hz, 2H), 6.92 (d, J = 10.0 Hz, 2H), 5.77 (tdd, J = 56.0, 6.0, 3.5Hz, 1H), 5.04 (s, 2H), 4.55 (dd, J = 10.5, 4.5 Hz, 1H), 4.00 (s, 3H), 2.99 – 2.85 (m, 1H), 2.81 – 2.65 (m, 1H).

^{13}C NMR (151 MHz, $CDCl_3$) δ 160.22, 158.62, 144.41 (dm, J = 261.2 Hz), 136.14, 130.59, 128.67, 128.23, 127.46, 121.09, 119.47 (t, J = 14.5 Hz), 117.80 (t, J = 17.0 Hz), 115.77, 114.51 (t, J = 241.5 Hz), 70.11, 66.65 (t, J = 6.0 Hz), 53.91, 32.47 (t, J = 24.0 Hz).

^{19}F NMR (565 MHz, $CDCl_3$) δ -116.02 – -117.50 (m), -133.94 – -134.06 (m), -135.84 – -135.98 (m).

HRMS (ESI) (m/z): calcd for $C_{24}H_{18}F_6NaO_5S$ ($[M + Na]^+$), 555.0671; found, 555.0670.

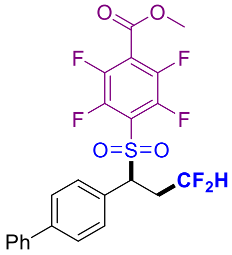

methyl (S)-4-((1-([1,1'-biphenyl]-4-yl)-3,3-difluoropropyl)sulfonyl)-2,3,5,6-tetra-fluorobenzoate (12)

This compound was obtained in 69% (69.3 mg) yield as white solid by the general procedure.

^{1}H NMR (500 MHz, $CDCl_3$) δ 7.59 – 7.53 (m, 4H), 7.45 (t, J = 7.6 Hz, 2H), 7.39 (d, J = 7.7 Hz, 3H), 5.83 (tdd, J = 55.8, 5.9, 3.5 Hz, 1H), 4.65 (dd, J = 10.3, 4.6 Hz, 1H), 3.97 (s, 3H), 2.98 (dt, J = 13.9, 5.3 Hz, 1H), 2.88 – 2.77 (m, 1H).

^{13}C NMR (151 MHz, $CDCl_3$) δ 158.56, 145.76 – 144.99 (m), 144.24 – 142.26 (m), 139.48, 129.70, 128.97, 128.13, 128.04, 127.12, 121.47, 119.42 (d, J = 23.8 Hz), 114.45 (t, J = 241.7 Hz), 67.00, 53.90, 32.59 (t, J = 24.0 Hz).

^{19}F NMR (565 MHz, $CDCl_3$)) δ -115.82 – -117.32 (m), -133.70 – -134.01 (m), -135.73 – -135.96 (m).

HRMS (ESI) (m/z): calcd for $C_{23}H_{16}F_6NaO_4S$ ($[M + Na]^+$), 525.0566; found, 525.0551.

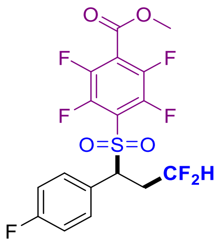

methyl-4-((3,3-difluoro-1-(4-fluorophenyl)propyl)sulfonyl)-2,3,5,6-tetrafluoro-benzoate (13)

This compound was obtained in 70% (62.2 mg) yield as white solid by the general procedure.

^{1}H NMR (500 MHz, $CDCl_3$) δ 7.32 (dd, J = 10.0, 5.0 Hz, 2H), 7.05 (t, J = 10.0 Hz, 2H), 5.79 (tdd, J = 55.5, 6.0, 3.5 Hz, 1H), 4.59 (dd, J = 10.0, 4.5 Hz, 1H), 3.99 (s, 3H), 3.02 – 2.86 (m, 1H), 2.83 – 2.64 (m, 1H).

^{13}C NMR (151 MHz, $CDCl_3$) δ 164.53, 162.86, 158.49, 144.59 (dm, J = 270.3 Hz), 131.26 (d, J = 8.6 Hz), 125.34 (d, J = 3.5 Hz), 119.04 (t, J = 14.7 Hz), 118.13 (t, J = 16.8 Hz), 116.72 (d, J = 21.4 Hz), 114.28 (t, J = 241.9 Hz), 66.27 (t, J = 4.5 Hz), 53.96, 32.59 (t, J = 23.9 Hz).

^{19}F NMR (565 MHz, $CDCl_3$) δ -109.12 (td, J = 8.1, 4.0 Hz), -115.63 – -118.41 (m), -133.45 – -134.64 (m), -135.17 – -136.08 (m).

HRMS (ESI) (m/z): calcd for $C_{17}H_{11}F_7NaO_4S$ ($[M + Na]^+$), 467.0158; found, 467.0144.

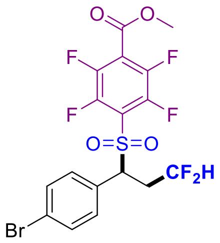

methyl-4-((1-(4-bromophenyl)-3,3-difluoropropyl)sulfonyl)-2,3,5,6-tetrafluoro-benzoate (14)

This compound was obtained in 52% (52.5 mg) yield as white solid by the general procedure.

^{1}H NMR (500 MHz, $CDCl_3$) δ 7.50 (d, J = 5.0 Hz, 2H), 7.21 (d, J = 5.0 Hz, 2H), 5.79 (tdd, J = 55.5, 5.0, 3.5 Hz, 1H), 4.57 (dd, J = 10.0, 5.0 Hz, 1H), 3.99 (s, 3H), 3.00 – 2.85 (m, 1H), 2.79 – 2.65 (m, 1H).

^{13}C NMR (151 MHz, $CDCl_3$) δ 158.46, 144.45 (dm, J = 261.2 Hz), 132.75, 130.81, 128.50, 125.03, 119.03 (t, J = 14.3 Hz), 118.23 (t, J = 16.8 Hz), 114.21 (t, J = 241.8 Hz), 66.43 (t, J = 5.3 Hz), 53.96, 32.57 (t, J = 23.8 Hz).

^{19}F NMR (565 MHz, $CDCl_3$) δ -115.46 – -117.88 (m), -133.35 – -134.55 (m), -135.04 – -135.93 (m).

HRMS (ESI) (m/z): calcd for $C_{17}H_{11}BrF_6NaO_4S$ ($[M + Na]^+$), 528.2354; found, 528.2349.

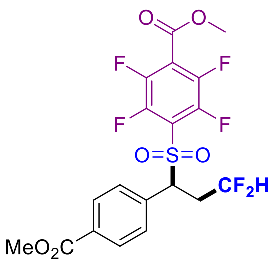

methyl (S)-4-((3,3-difluoro-1-(4-(methoxycarbonyl)phenyl)propyl)sulfonyl)-2,3,5,6-tetra-fluorobenzoate (15)

This compound was obtained in 57% (55.1 mg) yield as a yellow oil by the general procedure.

^{1}H NMR (500 MHz, $CDCl_3$) δ 8.00 (d, J = 8.1 Hz, 2H), 7.41 (d, J = 8.1 Hz, 2H), 5.80 (tdd, J = 55.6, 5.3, 3.6 Hz, 1H), 4.66 (dd, J = 10.1, 4.6 Hz, 1H), 3.98 (s, 3H), 3.91 (s, 3H), 3.10 – 2.91 (m, 1H), 2.84 – 2.69 (m, 1H).

^{13}C NMR (151 MHz, $CDCl_3$) δ 166.38, 159.86, 145.62 (dd, J = 23.0, 6.4 Hz), 143.85, 130.41 , 129.81, 123.92, 115.42 (t, J = 240.4 Hz), 111.72 (t, J = 16.2 Hz), 53.32, 52.22, 36.34 (t, J = 23.1 Hz), 35.21 (t, J = 5.3 Hz).

^{19}F NMR (565 MHz, $CDCl_3$) δ -115.88 – -118.17 (m), -138.39 – -138.69 (m), -140.72 – -141.18 (m).

HRMS (ESI) (m/z): calcd for $C_{15}H_{15}F_6O_6S$ ([M + H]$^+$), 485.0489; found, 485.0496.

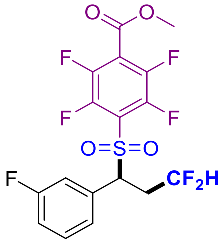

methyl-4-((3,3-difluoro-1-(3-fluorophenyl)propyl)sulfonyl)-2,3,5,6-tetrafluoro-benzoate (16)

This compound was obtained in 65% (57.8 mg) yield as white solid by the general procedure.

^{1}H NMR (500 MHz, $CDCl_3$) δ 7.32 (td, J = 10.0, 5.5 Hz, 1H), 7.14 – 7.05 (m, 3H), 5.81 (tdd, J = 55.5, 5.5, 3.5 Hz, 1H), 4.59 (dd, J = 10.0, 4.5 Hz, 1H), 3.99 (s, 3H), 3.02 – 2.87 (m, 1H), 2.82 – 2.67 (m, 1H).

^{13}C NMR (151 MHz, $CDCl_3$) δ 163.68, 162.03, 158.46, 144.44 (dm, J = 262.7 Hz), 131.89 (d, J = 7.4 Hz), 131.11 (d, J = 8.2 Hz), 125.14 (d, J = 3.1 Hz), 118.98 (t, J = 14.4 Hz), 118.22 (t, J = 16.8 Hz), 117.04 (q, J = 250.8 Hz), 114.19 (t, J = 241.8 Hz), 66.53 (d, J = 6.0 Hz), 53.96, 32.61 (t, J = 23.9 Hz).

^{19}F NMR (565 MHz, $CDCl_3$) δ -109.82 (q, J = 8.2 Hz), -115.75 – -117.48 (m), -133.90 – -134.03 (m), -135.46 – -135.59 (m).

HRMS (ESI) (m/z): calcd for $C_{17}H_{11}F_7NaO_4S$ ([M + Na]$^+$), 467.0158; found, 467.0165.

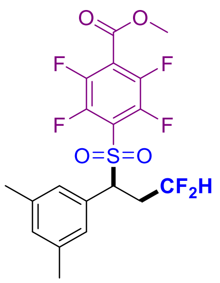

methyl-4-((1-(3,5-dimethylphenyl)-3,3-difluoropropyl)sulfonyl)-2,3,5,6-tetrafluorobenzoate (17)

This compound was obtained in 86% (78.1 mg) yield as white solid by the general procedure.

^{1}H NMR (500 MHz, $CDCl_3$) δ 7.07 (d, J = 7.5 Hz, 2H), 6.99 (dd, J = 8.5, 2.0 Hz, 1H), 5.73 (tdd, J = 56.0, 6.0, 3.5 Hz, 1H), 4.52 (dd, J = 10.5, 4.5 Hz, 1H), 3.99 (s, 3H), 2.96 – 2.83 (m, 1H), 2.83 – 2.67 (m, 1H), 2.21 (d, J = 8.0 Hz, 6H).

^{13}C NMR (151 MHz, $CDCl_3$) δ 158.65, 144.82 (dm, J = 259.5 Hz), 139.40, 138.07, 130.59, 130.08, 126.43, 119.49 (t, J = 14.2 Hz), 117.73 (t, J = 17.0 Hz), 114.56 (t, J = 241.3 Hz), 67.08 (t, J = 5.5 Hz), 53.91, 32.51 (t, J = 24.1 Hz), 19.68.

^{19}F NMR (565 MHz, $CDCl_3$) δ -116.64 – -116.86 (m), -133.93 – -134.06 (m), -136.21 – -136.33 (m).

HRMS (ESI) (m/z): calcd for $C_{19}H_{16}F_6NaO_4S$ ([M + Na]$^+$), 477.0566; found, 477.0569.

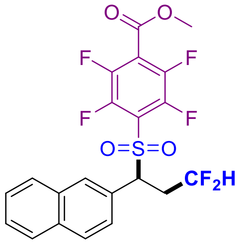

methyl-4-((3,3-difluoro-1-(naphthalen-2-yl)propyl)sulfonyl)-2,3,5,6-tetrafluorobenzoate (18)

This compound was obtained in 43% (41.0 mg) yield as white solid by the general procedure.

^{1}H NMR (500 MHz, $CDCl_3$) δ 7.87 – 7.76 (m, 4H), 7.54 (qt, J = 8.5, 5.0 Hz, 2H), 7.42 (dd, J = 8.5, 2.0 Hz, 1H), 5.77 (tdd, J = 56.0, 5.5, 3.5 Hz, 1H), 4.78 (dd, J = 10.0, 4.5 Hz, 1H), 3.95 (s, 3H), 3.09 – 2.96 (m, 1H), 2.97 – 2.82 (m, 1H).

^{13}C NMR (151 MHz, $CDCl_3$) δ 159.98, 144.82 (dm, J = 262.7 Hz), 136.20, 133.37, 132.69, 129.12, 127.88, 127.64, 126.67, 126.46, 126.25, 125.24, 124.66 (t, J = 15.6 Hz), 115.71 (t, J = 240.3 Hz), 111.38 (t, J = 16.0 Hz), 53.27, 36.60 (t, J = 21.7 Hz), 35.60 (d, J = 6.5 Hz).

^{19}F NMR (565 MHz, $CDCl_3$) δ -115.68 – -118.04 (m), -138.68 – -138.85 (m), -140.91 – -141.07 (m).

HRMS (ESI) (m/z): calcd for $C_{21}H_{14}F_6NaO_4S$ ([M + Na]$^+$), 499.0409; found, 499.0403.

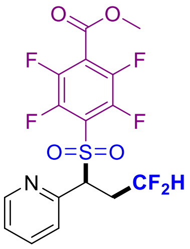

methyl-4-((3,3-difluoro-1-(pyridin-2-yl)propyl)sulfonyl)-2,3,5,6-tetrafluoro-benzoate (19)

This compound was obtained in 44% (37.6 mg) yield as white solid by the general procedure.

^{1}H NMR (500 MHz, $CDCl_3$) δ 8.42 (d, J = 4.5 Hz, 1H), 7.77 (td, J = 7.8, 1.8 Hz, 1H), 7.53 (d, J = 7.8 Hz, 1H), 7.31 (ddd, J = 7.7, 4.8, 1.1 Hz, 1H), 6.05 – 5.69 (m, 1H), 4.75 (dd, J = 10.0, 4.6 Hz, 1H), 4.00 (s, 3H), 3.08 – 2.91 (m, 2H).

^{13}C NMR (151 MHz, $CDCl_3$) δ 158.68, 149.76, 149.34, 146.05 – 145.64 (m), 145.40 – 144.95 (m), 144.22 – 143.93 (m), 143.63 – 143.16 (m), 137.34, 126.35, 124.67, 118.52, 118.21 (t, J = 17.0 Hz), 114.47 (t, J = 241.4 Hz), 68.70 (t, J = 5.5 Hz), 53.92, 31.70 (t, J = 23.7 Hz).

^{19}F NMR (565 MHz, $CDCl_3$) δ -116.48 (dt, J = 56.2, 15.8 Hz), -133.07 – -133.40 (m), -136.30 – -136.54 (m).

HRMS (ESI) (m/z): calcd for $C_{16}H_{12}F_6NO_4S$ ([M + H]$^+$), 428.0386; found, 428.0380.

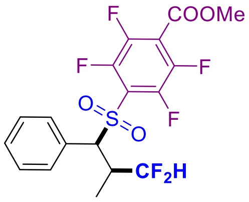

methyl 4-(((1S,2R)-3,3-difluoro-2-methyl-1-phenylpropyl)sulfonyl)-2,3,5,6-tetrafluorobenzoate (20)

This compound was obtained in 47% (41.3 mg) yield as a colorless oil by the general procedure.

^{1}H NMR (500 MHz, $CDCl_3$) δ 7.37 – 7.26 (m, 8.3H), 6.29 (td, J = 55.9, 2.7 Hz, 1H), 5.50 – 5.11 (m, 0.77H), 4.56 (d, J = 8.4 Hz, 1H), 4.44 (d, J = 10.9 Hz, 0.73H), 3.95 (d, J = 5.2 Hz, 5.15H), 3.26 – 3.04 (m, 1.72H), 1.58 (d, J = 6.7 Hz, 2.3H), 1.06 (d, J = 7.0 Hz, 3H).

^{13}C NMR (151 MHz, $CDCl_3$) δ 158.57, 145.26 – 144.83 (m), 143.43 – 143.01 (m), 130.48, 130.34, 130.19, 129.70, 129.65, 129.35, 129.31, 128.86, 117.34 – 113.90 (m), 73.91 (d, J = 7.8 Hz), 72.29 (d, J = 4.4 Hz), 53.82, 53.79, 39.08 – 38.68 (m), 37.33 (t, J = 21.8 Hz), 10.12 (t, J = 5.0 Hz), 9.85 (t, J = 4.9 Hz).

^{19}F NMR (565 MHz, $CDCl_3$) δ -122.11 (dddd, J = 1698.4, 283.6, 55.7, 6.2 Hz), -130.58 – -131.40 (m), -134.41 – -134.56 (m), -134.58 – -134.78 (m), -136.16 – -136.40 (m), -136.42 – -136.63 (m).

HRMS (ESI) (m/z): calcd for $C_{18}H_{14}F_6NaO_4S$ ([M + Na]$^+$), 463.0410; found, 463.0416.

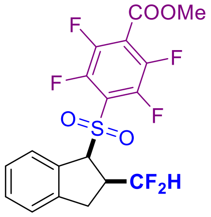

methyl 4-(((1S,2R)-2-(difluoromethyl)-2,3-dihydro-1H-inden-1-yl)sulfonyl)-2,3,5,6-tetrafluoro-benzoate (21)

This compound was obtained in 53% (49.9 mg) yield as a colorless oil by the general procedure.

^{1}H NMR (600 MHz, $CDCl_3$) δ 7.45 (d, J = 7.7 Hz, 1H), 7.41 (dd, J = 7.5, 1.1 Hz, 1H), 7.34 – 7.28 (m, 2H), 5.74 (td, J = 56.0, 4.6 Hz, 1H), 5.05 – 4.97 (m, 1H), 4.03 (s, 3H), 3.49 (dd, J = 17.0, 9.1 Hz, 1H), 3.39 – 3.26 (m, 1H), 3.05 (dd, J = 16.9, 2.2 Hz, 1H).

^{13}C NMR (151 MHz, $CDCl_3$) δ 158.66, 146.00 – 145.27 (m), 143.98, 144.26 – 143.60 (m), 130.83, 130.30, 127.73, 127.10, 125.42, 119.90 (t, J = 14.7 Hz), 118.21 (t, J = 16.8 Hz), 115.81 (t, J = 244.2 Hz), 72.04 (d, J = 4.5 Hz), 53.94, 44.04 (t, J = 21.1 Hz), 31.89 – 31.80 (m).

^{19}F NMR (565 MHz, $CDCl_3$) δ -120.50 – -125.08 (m), -133.47 – -133.59 (m), -135.43 – -135.59 (m).

HRMS (ESI) (m/z): calcd for $C_{18}H_{12}F_6NaO_4S$ ([M + Na]$^+$), 461.0253; found, 461.0259.

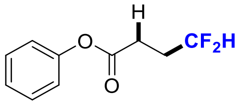

phenyl 4,4-difluorobutanoate (22)

This compound was obtained in 35% (14.0 mg) yield as a colorless oil by the general procedure.

^{1}H NMR (500 MHz, $CDCl_3$) δ 7.38 (dd, J = 8.5, 7.4 Hz, 2H), 7.24 (d, J = 7.0 Hz, 1H), 7.10 – 7.06 (m, 2H), 6.00 (tt, J = 56.5, 4.1 Hz, 1H), 2.77 (t, J = 7.4 Hz, 2H), 2.28 (ttd, J = 17.5, 7.4, 4.1 Hz, 2H).

^{13}C NMR (151 MHz, $CDCl_3$) δ 170.66, 150.46, 129.50, 126.04, 121.40, 115.91 (t, J = 239.1 Hz), 29.29 (t, J = 22.3 Hz), 26.96 (t, J = 6.0 Hz).

^{19}F NMR (565 MHz, $CDCl_3$) δ -117.67 (dt, J = 57.0, 17.8 Hz).

HRMS (ESI) (m/z): calcd for $C_{10}H_{11}F_2O_2$ ([M + H]$^+$), 201.0722; found, 201.0731.

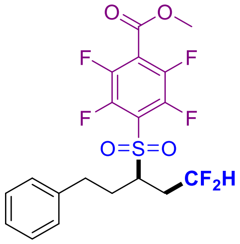

methyl-4-((1,1-difluoro-5-phenylpentan-3-yl)sulfonyl)-2,3,5,6-tetrafluoro-benzoate (23)

This compound was obtained in 60% (54.5 mg) yield as a yellow oil by the general procedure.

^{1}H NMR (500 MHz, $CDCl_3$) δ 7.26 – 7.18 (m, 3H), 7.02 – 6.98 (m, 2H), 6.19 (tdd, J =

56.5, 6.0, 3.5 Hz, 1H), 4.04 (s, 3H), 3.44 – 3.29 (m, 1H), 2.92 (dt, J = 12.5, 6.0 Hz, 1H), 2.72 – 2.54 (m, 2H), 2.38 – 2.22 (m, 1H), 2.17 – 2.04 (m, 2H).

^{13}C NMR (151 MHz, $CDCl_3$) δ 158.58, 144.46 (dm, J = 243.3 Hz), 138.80, 128.75, 128.24, 126.89, 119.16 (t, J = 14.4 Hz), 118.22 (t, J = 17.2 Hz), 114.51 (t, J = 240.6 Hz), 58.98 (t, J = 4.5 Hz), 53.99, 32.24 (t, J = 21.0 Hz), 31.74, 30.86.

^{19}F NMR (565 MHz, $CDCl_3$) δ -113.57 (ddt, J = 287.1, 56.0, 13.1 Hz), -117.82 (dddd, J = 286.7, 56.3, 23.0, 14.8 Hz), -134.23 – -134.39 (m), -135.33 – -135.56 (m).

HRMS (ESI) (m/z): calcd for $C_{19}H_{16}F_6NaO_4S$ ($[M + Na]^+$), 477.0566; found, 477.0569.

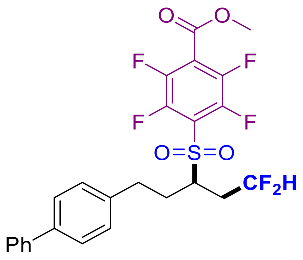

methyl-4-((5-([1,1'-biphenyl]-4-yl)-1,1-difluoropentan-3-yl)sulfonyl)-2,3,5,6-tetrafluorobenzoate (24)

This compound was obtained in 76% (80.6 mg) yield as a yellow oil by the general procedure.

^{1}H NMR (500 MHz, $CDCl_3$) δ 7.55 – 7.51 (m, 2H), 7.44 (d, J = 8.0 Hz, 2H), 7.40 (t, J = 7.5 Hz, 2H), 7.33 – 7.28 (m, 1H), 7.05 (d, J = 8.0 Hz, 2H), 6.18 (tdd, J = 56.0, 6.0, 3.5 Hz, 1H), 3.81 (s, 3H), 3.39 (tt, J = 8.0, 4.5 Hz, 1H), 2.92 (dt, J = 13.9, 6.0 Hz, 1H), 2.71 – 2.55 (m, 2H), 2.36 – 2.21 (m, 1H), 2.18 – 2.04 (m, 2H).

^{13}C NMR (151 MHz, $CDCl_3$) δ 158.41, 144.54 (dm, J = 242.5 Hz), 140.38, 139.77, 137.87, 128.74 (d, J = 6.9 Hz), 127.39, 127.34, 126.98, 119.20 (t, J = 14.9 Hz), 118.08 (t, J = 15.2 Hz), 114.55 (t, J = 240.5 Hz), 59.09 (t, J = 4.8 Hz), 53.79, 32.27 (t, J = 23.7 Hz), 31.38, 30.79.

^{19}F NMR (565 MHz, $CDCl_3$) δ -113.54 (ddt, J = 287.0, 55.9, 13.4 Hz), -117.80 (dddd, J = 286.6, 56.3, 22.9, 15.2 Hz), -134.27 – -134.42 (m), -135.14 – -135.29 (m).

HRMS (ESI) (m/z): calcd for $C_{25}H_{20}F_6NaO_4S$ ($[M + Na]^+$), 553.0879; found, 553.0872.

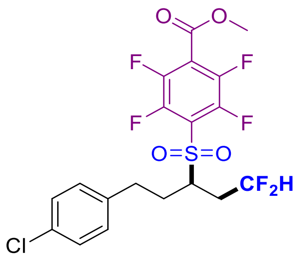

methyl-4-((5-(4-chlorophenyl)-1,1-difluoropentan-3-yl)sulfonyl)-2,3,5,6-tetra-fluorobenzoate (25)

This compound was obtained in 67% (65.5 mg) yield as a yellow oil by the general procedure.

^{1}H NMR (500 MHz, $CDCl_3$) δ 7.21 (d, J = 10.0 Hz, 2H), 6.98 (d, J = 5.0 Hz, 2H), 6.16 (tdd, J = 56.5, 5.5, 3.5 Hz, 1H), 4.04 (s, 3H), 3.37 (tt, J = 8.0, 4.5 Hz, 1H), 2.89 (m, 1H), 2.61 (dt, J = 14.0, 8.0 Hz, 2H), 2.35 – 2.20 (m, 1H), 2.18 – 2.00 (m, 2H).

^{13}C NMR (151 MHz, $CDCl_3$) δ 158.47, 144.64 (dm, J = 242.7 Hz), 137.43, 132.76, 129.63, 128.89, 119.19 (t, J = 15.1 Hz), 118.30 (t, J = 17.0 Hz), 114.43 (t, J = 240.7 Hz), 59.13 (d, J = 5.2 Hz), 53.99, 32.29 (t, J = 23.5 Hz), 31.20, 30.59.
^{19}F NMR (565 MHz, $CDCl_3$) δ -113.82 (ddt, J = 286.9, 55.8, 14.1 Hz), -117.78 (dddd, J = 287.1, 56.3, 21.6, 16.3 Hz), -134.18 – -134.31 (m), -135.16 – -135.28 (m).
HRMS (ESI) (m/z): calcd for $C_{19}H_{15}ClF_6NaO_4S$ ($[M + Na]^+$), 511.0181; found, 511.0185.

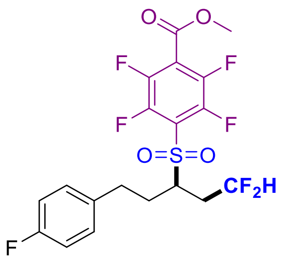

methyl-4-((1,1-difluoro-5-(4-fluorophenyl)pentan-3-yl)sulfonyl)-2,3,5,6-tetra-fluorobenzoate (26)
This compound was obtained in 80% (75.6 mg) yield as a yellow oil by the general procedure.
^{1}H NMR (600 MHz, $CDCl_3$) δ 7.04 – 6.99 (m, 2H), 6.95 (t, J = 10.0 Hz, 2H), 6.17 (tdd, J = 56.0, 5.5, 3.3 Hz, 1H), 4.05 (s, 3H), 3.39 (dq, J = 8.1, 4.4, 4.0 Hz, 1H), 2.91 (dt, J = 13.6, 6.5 Hz, 1H), 2.73 – 2.57 (m, 2H), 2.29 (dtd, J = 21.0, 15.3, 5.3 Hz, 1H), 2.18 – 2.04 (m, 2H).
^{13}C NMR (151 MHz, $CDCl_3$) δ 162.51, 160.88, 158.48, 144.59 (dm, J = 242.7 Hz), 129.77 (d, J = 8.0 Hz), 119.25 (q, J = 253.1 Hz), 118.34 (t, J = 18.0 Hz), 115.71, 115.57, 114.41 (t, J = 241.3 Hz), 59.14, 54.01, 32.33 (t, J = 23.6 Hz), 31.04.
^{19}F NMR (565 MHz, $CDCl_3$) δ -113.79 (ddt, J = 286.9, 55.8, 13.9 Hz), -115.69 (dd, J = 9.8, 4.2 Hz), -117.78 (dddd, J = 286.9, 56.5, 21.7, 16.0 Hz), -134.11 – -134.24 (m), -135.29 – -135.41 (m).
HRMS (ESI) (m/z): calcd for $C_{19}H_{16}F_7O_4S$ ($[M + H]^+$), 473.0579; found, 473.0580.

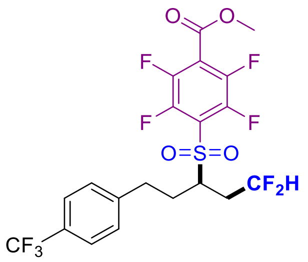

methyl-4-((1,1-difluoro-5-(4-(trifluoromethyl)phenyl)pentan-3-yl)sulfonyl)-2,3,5,6-tetrafluoro-benzoate (27)
This compound was obtained in 45% (47.0 mg) yield as a yellow oil by the general procedure.
^{1}H NMR (500 MHz, $CDCl_3$) δ 7.51 (d, J = 5.0 Hz, 2H), 7.19 (d, J = 10.0 Hz, 2H), 6.16 (tdd, J = 56.0, 5.5, 3.5 Hz, 1H), 4.02 (s, 3H), 3.43 – 3.35 (m, 1H), 2.98 (m, 1H), 2.74 (dt, J = 14.0, 8.1 Hz, 1H), 2.69 – 2.53 (m, 1H), 2.37 – 2.23 (m, 1H), 2.24 – 2.06 (m, 2H).

^{13}C NMR (151 MHz, $CDCl_3$) δ 158.41, 144.64 (dm, J = 246.1 Hz), 143.19, 129.27 (q, J = 32.7 Hz), 128.69, 125.69 (q, J = 3.8 Hz), 124.08 (q, J = 264.3 Hz), 119.16 (t, J = 14.4 Hz), 118.41 (t, J = 17.0 Hz), 114.40 (t, J = 240.7 Hz), 59.26 (t, J = 4.5 Hz), 53.97, 32.25 (t, J = 23.4 Hz), 31.63, 30.28.
^{19}F NMR (565 MHz, $CDCl_3$) δ -62.65 (s), -113.93 (dddd, J = 287.1, 55.9, 15.9, 8.1 Hz), -117.07 – -118.45 (m), -134.17 – -134.41 (m), -135.06 – -135.22 (m).
HRMS (ESI) (m/z): calcd for $C_{20}H_{15}F_9NaO_4S$ ([M + Na]$^+$), 545.0440; found, 545.0456.

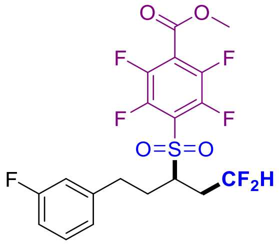

methyl-4-((1,1-difluoro-5-(3-fluorophenyl)pentan-3-yl)sulfonyl)-2,3,5,6-tetra-fluorobenzoate (28)
This compound was obtained in 71% (67.1 mg) yield as a yellow oil by the general procedure.
^{1}H NMR (600 MHz, $CDCl_3$) δ 7.23 (td, J = 8.0, 6.0 Hz, 1H), 6.92 (td, J = 8.5, 2.6 Hz, 1H), 6.85 (d, J = 10.0 Hz, 1H), 6.75 (dt, J = 9.7, 2.0 Hz, 1H), 6.17 (tdd, J = 56.1, 5.4, 3.3 Hz, 1H), 4.04 (s, 3H), 3.39 (tt, J = 8.0, 4.6 Hz, 1H), 2.93 (m, 1H), 2.70 – 2.58 (m, 2H), 2.35 – 2.25 (m, 1H), 2.21 – 2.12 (m, 1H), 2.14 – 2.05 (m, 1H).
^{13}C NMR (151 MHz, $CDCl_3$) δ 163.70, 162.06, 158.50, 144.21 (dm, J = 260.9 Hz), 141.48 (d, J = 7.1 Hz), 127.33 (q, J = 258.5 Hz), 124.04 (d, J = 2.9 Hz), 119.18 (t, J = 14.1 Hz), 118.29 (t, J = 16.6 Hz), 115.12 (d, J = 21.2 Hz), 114.43 (t, J = 240.6 Hz), 113.87 (d, J = 20.9 Hz), 59.17, 53.99, 32.25 (t, J = 23.6 Hz), 31.54, 30.41.
^{19}F NMR (565 MHz, $CDCl_3$) δ -112.41 – -112.51 (m), -113.83 (ddt, J = 287.0, 55.9, 14.2 Hz), -117.76 (dddd, J = 287.0, 56.2, 21.7, 16.2 Hz), -134.17 – -134.41 (m), -135.13 – -135.27 (m).
HRMS (ESI) (m/z): calcd for $C_{19}H_{15}F_7NaO_4S$ ([M + Na]$^+$), 495.0477; found, 495.0465.

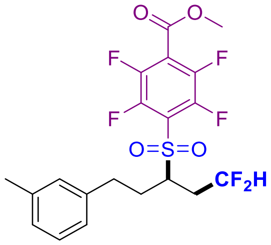

methyl-4-((1,1-difluoro-5-(m-tolyl)pentan-3-yl)sulfonyl)-2,3,5,6-tetrafluoro-benzoate (29)
This compound was obtained in 56% (52.5 mg) yield as a yellow oil by the general procedure.
^{1}H NMR (500 MHz, $CDCl_3$) δ 7.09 (t, J = 10.0 Hz, 1H), 7.00 (d, J = 5.0 Hz, 1H), 6.85 (s, 1H), 6.78 (d, J = 5.0 Hz, 1H), 6.20 (tdd, J = 56.0, 5.5, 3.5 Hz, 1H), 4.04 (s, 3H), 3.45 – 3.29 (m, 1H), 2.87 (dt, J = 13.9, 6.0 Hz, 1H), 2.74 – 2.57 (m, 1H), 2.54 (dt, J = 13.9, 8.0 Hz, 1H), 2.35 – 2.30 (m, 1H), 2.28 (s, 3H), 2.07 (m, 2H).
^{13}C NMR (151 MHz, $CDCl_3$) δ 158.55, 144.52 (dm, J = 273.3 Hz), 138.77, 138.55,

129.18, 128.62, 127.56, 125.11, 119.27 (t, J = 14.4 Hz), 118.08 (t, J = 16.8 Hz), 114.55 (t, J = 240.4 Hz), 59.05 (t, J = 5.1 Hz), 53.93, 32.23 (t, J = 23.8 Hz), 31.69, 30.90, 21.16.
^{19}F NMR (565 MHz, $CDCl_3$) δ -113.56 (ddt, J = 287.2, 56.0, 13.3 Hz), -117.78 (dddd, J = 287.1, 56.8, 23.5, 15.3 Hz), -134.30 – -134.44 (m), -135.55 – -135.65 (m).
HRMS (ESI) (m/z): calcd for $C_{20}H_{18}F_6NaO_4S$ ([M + Na]$^+$), 491.0728; found, 491.0735.

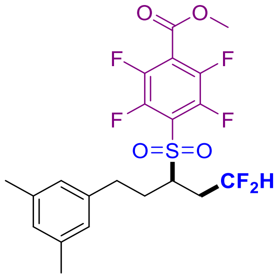

methyl-4-((5-(3,5-dimethylphenyl)-1,1-difluoropentan-3-yl)sulfonyl)-2,3,5,6-tetra-fluorobenzoate (30)
This compound was obtained in 71% (68.5 mg) yield as a yellow oil by the general procedure.
^{1}H NMR (500 MHz, $CDCl_3$) δ 6.82 (s, 1H), 6.63 (s, 2H), 6.21 (tdd, J = 56.5, 6.0, 3.5 Hz, 1H), 4.03 (s, 3H), 3.45 – 2.56 (m, 1H), 2.82 (dt, J = 13.5, 6.0 Hz, 1H), 2.71 – 2.56 (m, 1H), 2.49 (dt, J = 13.5, 8.0 Hz, 1H), 2.39 – 2.28 (m, 1H), 2.23 (s, 6H), 2.14 – 2.04 (m, 2H).
^{13}C NMR (151 MHz, $CDCl_3$) δ 158.54, 144.55 (dm, J = 261.3 Hz), 138.76, 138.43, 128.40, 126.10, 119.40 (t, J = 14.2 Hz), 117.90 (t, J = 16.6 Hz), 114.56 (t, J = 240.5 Hz),59.10, 53.89, 32.24 (t, J = 23.9 Hz), 31.63, 30.97, 21.03.
^{19}F NMR (565 MHz, $CDCl_3$) δ -113.52 (ddt, J = 287.2, 56.0, 13.3 Hz), -117.78 (dddd, J = 287.2, 56.8, 23.5, 14.8 Hz), -134.04 – -134.80 (m), -135.36 – -136.02 (m).
HRMS (ESI) (m/z): calcd for $C_{21}H_{20}F_6NaO_4S$ ([M + Na]$^+$), 505.0878; found, 505.0885.

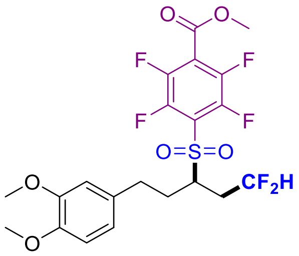

methyl-4-((5-(3,4-dimethoxyphenyl)-1,1-difluoropentan-3-yl)sulfonyl)-2,3,5,6-tetrafluorobenzoate (31)
This compound was obtained in 84% (86.4 mg) yield as a yellow oil by the general procedure.
^{1}H NMR (500 MHz, $CDCl_3$) δ 6.73 (d, J = 10.0 Hz, 1H), 6.56 (d, J = 5.0 Hz, 1H), 6.53 (dd, J = 8.0, 2.0 Hz, 1H), 6.20 (tdd, J = 56.5, 6.0, 3.5 Hz, 1H), 4.04 (s, 3H), 3.86 (s, 3H), 3.82 (s, 3H), 3.43 (dt, J = 8.0, 4.0 Hz, 1H), 2.88 (dt, J = 14.0, 6.0 Hz, 1H), 2.73 – 2.57 (m, 1H), 2.60 – 2.50 (m, 1H), 2.35 – 2.23 (m, 1H), 2.14 – 2.01 (m, 2H).
^{13}C NMR (151 MHz, $CDCl_3$) δ 158.52, 149.10, 147.91, 144.63 (dm, J = 262.7 Hz), 131.17, 120.06, 119.40 (d, J = 14.1 Hz), 117.95 (d, J = 17.1 Hz), 114.55 (t, J = 240.5 Hz), 111.59, 111.26, 59.01 (d, J = 5.2 Hz), 55.79, 55.76, 53.93, 32.29 (t, J = 23.7 Hz),

31.38, 31.01.

^{19}F NMR (565 MHz, $CDCl_3$) δ -113.25 (ddt, J = 286.4, 56.0, 13.1 Hz), -117.78 (dddd, J = 286.6, 56.4, 23.2, 15.1 Hz), -134.19 – -134.34 (m), -135.32 – -134.47 (m).

HRMS (ESI) (m/z): calcd for $C_{21}H_{20}F_6NaO_6S$ ($[M + Na]^+$), 537.0777; found, 537.0771.

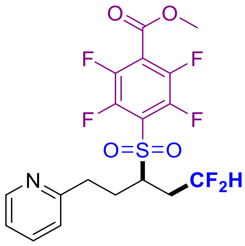

methyl-4-((1,1-difluoro-5-(pyridin-2-yl)pentan-3-yl)sulfonyl)-2,3,5,6-tetrafluoro-benzoate (32)

This compound was obtained in 91% (82.9 mg) yield as a yellow oil by the general procedure.

^{1}H NMR (500 MHz, $CDCl_3$) δ 8.33 (ddd, J = 5.0, 2.0, 1.0 Hz, 1H), 7.58 (td, J = 8.0, 2.0 Hz, 1H), 7.14 – 7.06 (m, 2H), 6.20 (tdd, J = 56.5, 6.0, 3.5 Hz, 1H), 4.03 (s, 3H), 3.55 – 3.50 (m, 1H), 3.04 (dt, J = 15.0, 6.0 Hz, 1H), 2.86 – 2.80 (m, 1H), 2.69 – 2.56 (m, 1H), 2.45 – 2.38 (m, 1H), 2.35 – 2.24 (m, 1H), 2.18 – 2.11 (m, 1H).

^{13}C NMR (151 MHz, $CDCl_3$) δ 158.66, 158.44, 149.36, 144.63 (dm, J = 261.1 Hz), 136.63, 123.47, 121.83, 119.59 (t, J = 14.1 Hz), 118.08 (t, J = 17.0 Hz), 114.56 (t, J = 240.4 Hz), 59.29 (t, J = 5.2 Hz), 53.95, 33.07, 32.05 (t, J = 24.0 Hz), 27.80.

^{19}F NMR (565 MHz, $CDCl_3$) δ -113.79 (dddd, J = 286.7, 56.0, 16.0, 11.2 Hz), -117.62 (dddd, J = 287.0, 56.4, 23.3, 13.7 Hz), -134.13 – -134.29 (m), -135.72 – -135.84 (m).

HRMS (ESI) (m/z): calcd for $C_{18}H_{16}F_6NO_4S$ ($[M + H]^+$), 456.0699; found, 456.0694.

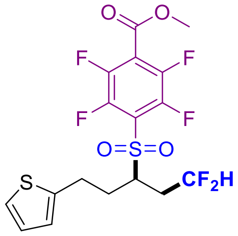

methyl-4-((1,1-difluoro-5-(thiophen-2-yl)pentan-3-yl)sulfonyl)-2,3,5,6-tetra-fluorobenzoate (33)

This compound was obtained in 63% (58.0 mg) yield as a yellow oil by the general procedure.

^{1}H NMR (500 MHz, $CDCl_3$) δ 7.10 (dd, J = 5.5, 1.0 Hz, 1H), 6.88 (dd, J = 5.0, 3.5 Hz, 1H), 6.76 – 6.72 (m, 1H), 6.18 (tdd, J = 56.0, 5.5, 3.5 Hz, 1H), 4.03 (s, 3H), 3.42 (tt, J = 8.0, 4.5 Hz, 1H), 3.11 (dt, J = 15.0, 5.5 Hz, 1H), 2.92 – 2.86 (m, 1H), 2.74 – 2.56 (m, 1H), 2.31 – 2.20 (m, 1H), 2.19 – 2.04 (m, 2H).

^{13}C NMR (151 MHz, $CDCl_3$) δ 158.61, 144.67 (dm, J = 262.3 Hz), 141.13, 127.08, 125.95, 124.49, 119.07 (t, J = 14.2 Hz), 118.25 (t, J = 16.8 Hz), 114.46 (t, J = 240.6 Hz), 58.92 (t, J = 5.0 Hz), 54.01, 32.18 (t, J = 23.7 Hz), 31.28, 26.10.

^{19}F NMR (565 MHz, $CDCl_3$) δ -113.57 (ddt, J = 286.7, 56.0, 13.5 Hz), -117.73 (dddd, J = 286.6, 56.2, 22.8, 15.2 Hz), -133.86 – -134.73 (m), -135.17 – -136.01 (m).

HRMS (ESI) (m/z): calcd for $C_{17}H_{15}F_6NO_4S_2$ ($[M + H]^+$), 461.0238; found, 461.0235.

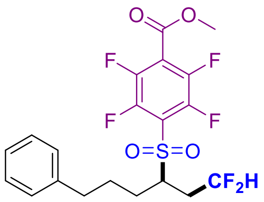

methyl-4-((1,1-difluoro-6-phenylheptan-3-yl)sulfonyl)-2,3,5,6-tetrafluorobenzoate (34)

This compound was obtained in 70% (65.6 mg) yield as a yellow oil by the general procedure.

^{1}H NMR (500 MHz, $CDCl_3$) δ 7.23 (t, J = 5.0 Hz, 2H), 7.16 (t, J = 5.0 Hz, 1H), 7.08 (d, J = 10.0 Hz, 2H), 6.13 (tdd, J = 56.2, 5.5, 3.5 Hz, 1H), 4.04 (s, 3H), 3.41 (tt, J = 8.2, 4.6 Hz, 1H), 2.69 – 2.57 (m, 2H), 2.59 – 2.44 (m, 1H), 2.31 – 2.18 (m, 1H), 1.98 – 1.87 (m, 1H), 1.89 – 1.77 (m, 1H), 1.77 – 1.65 (m, 2H).

^{13}C NMR (151 MHz, $CDCl_3$) δ 158.58, 144.63 (dm, J = 261.7 Hz), 140.26, 128.47, 128.24, 126.29, 119.50 (t, J = 14.3 Hz), 118.15 (t, J = 16.8 Hz), 114.48 (t, J = 240.5 Hz), 60.70 (t, J = 5.0 Hz), 53.97, 34.61, 31.75 (t, J = 23.6 Hz), 27.35, 27.03.

^{19}F NMR (565 MHz, $CDCl_3$) δ -114.19 (ddt, J = 287.0, 56.0, 14.2 Hz), -117.74 (dddd, J = 286.7, 56.2, 21.7, 15.3 Hz), -134.10 – -134.25 (m), -135.16 – -135.33 (m).

HRMS (ESI) (m/z): calcd for $C_{20}H_{18}F_6O_4S$ ($[M + H]^+$), 469.0981; found, 469.0981.

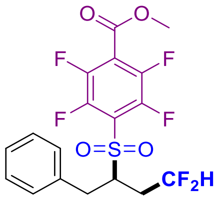

methyl-4-((4,4-difluoro-1-phenylbutan-2-yl)sulfonyl)-2,3,5,6-tetrafluorobenzoate (35)

This compound was obtained in 75% (66.0 mg) yield as a yellow oil by the general procedure.

^{1}H NMR (500 MHz, $CDCl_3$) δ 7.24 – 7.11 (m, 5H), 6.05 (tt, J = 56.5, 4.5 Hz, 1H), 4.01 (s, 3H), 4.01 – 3.90 (m, 1H), 3.29 (dd, J = 14.5, 8.0 Hz, 1H), 3.13 (dd, J = 14.5, 6.0 Hz, 1H), 2.80 – 2.63 (m, 1H), 2.33 – 2.16 (m, 1H).

^{13}C NMR (151 MHz, $CDCl_3$) δ 158.51, 144.17 (dm, J = 262.3 Hz), 134.65, 128.83, 128.80, 127.83, 120.62 (t, J = 14.0 Hz), 117.73 (t, J = 16.9 Hz), 114.57 (t, J = 240.8 Hz), 62.34 (d, J = 5.0 Hz), 53.90, 35.91, 32.23 (t, J = 23.8 Hz).

^{19}F NMR (565 MHz, $CDCl_3$) δ -114.39 (dddd, J = 286.7, 56.1, 16.8, 12.6 Hz), -117.54 (dddd, J = 286.6, 56.2, 21.0, 13.9 Hz), -133.95 – -134.07 (m), -135.95 – -136.12 (m).

HRMS (ESI) (m/z): calcd for $C_{18}H_{14}F_6NaO_4S$ ($[M + Na]^+$), 463.0409; found, 463.0403.

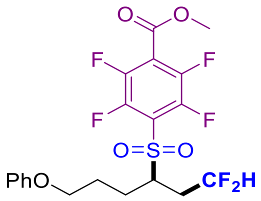

methyl-4-((1,1-difluoro-6-phenoxyhexan-3-yl)sulfonyl)-2,3,5,6-tetrafluoro-benzoate (36)

This compound was obtained in 80% (77.5 mg) yield as a yellow oil by the general procedure.

^{1}H NMR (500 MHz, $CDCl_3$) δ 7.26 (t, J = 10.0 Hz, 2H), 6.94 (t, J = 7.0 Hz, 1H), 6.79 (d, J = 8.0 Hz, 2H), 6.18 (tt, J = 56.0, 4.5 Hz, 1H), 4.01 (s, 3H), 3.95 (t, J = 5.5 Hz, 2H), 3.61 (t, J = 6.0 Hz, 1H), 2.65 – 2.51 (m, 1H), 2.31 (m, 1H), 2.23 – 2.12 (m, 1H), 2.09 – 1.85 (m, 3H).

^{13}C NMR (151 MHz, $CDCl_3$) δ 158.32, 14.71 (dm, J = 255.7 Hz), 129.55, 121.10, 119.66, 118.19, 114.45 (t, J = 240.7 Hz), 114.23, 66.18, 60.45 (t, J = 4.5 Hz), 53.92, 32.17 (t, J = 23.6 Hz), 25.50, 25.47.

^{19}F NMR (565 MHz, $CDCl_3$) δ -114.35 (ddt, J = 287.2, 56.0, 14.6 Hz), -117.57 (dddd, J = 287.2, 56.2, 21.1, 15.6 Hz), -133.95 – -134.23 (m), -135.01 – -135.26 (m).

HRMS (ESI) (m/z): calcd for $C_{20}H_{18}F_6NaO_5S$ ($[M + Na]^+$), 507.0671; found, 507.0680.

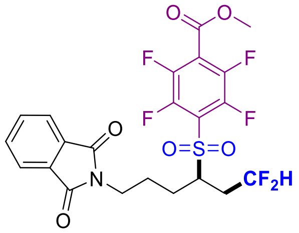

methyl-4-((6-(1,3-dioxoisoindolin-2-yl)-1,1-difluorohexan-3-yl)sulfonyl)-2,3,5,6-tetrafluoro-benzoate (37)

This compound was obtained in 83% (89.2 mg) yield as a yellow oil by the general procedure.

^{1}H NMR (500 MHz, $CDCl_3$) δ 7.78 (dd, J = 5.4, 3.1 Hz, 2H), 7.70 (dd, J = 5.5, 3.1 Hz, 2H), 6.14 (tdd, J = 56.5, 5.5, 3.5 Hz, 1H), 4.02 (s, 3H), 3.65 (dt, J = 12.0, 6.5 Hz, 2H), 3.60 – 3.53 (m, 1H), 2.60 – 2.47 (m, 1H), 2.30 – 2.18 (m, 1H), 1.95 – 1.87 (m, 2H), 1.86 – 1.75 (m, 2H).

^{13}C NMR (151 MHz, $CDCl_3$) δ 168.28, 158.65, 144.47 (dm, J = 244.2 Hz), 134.18, 131.75, 123.34, 119.41 (t, J = 14.2 Hz), 118.26 (t, J = 17.0 Hz), 114.48 (t, J = 240.6 Hz), 60.09 (t, J = 4.9 Hz), 53.94, 36.45, 31.59 (t, J = 23.5 Hz), 25.59, 24.87.

^{19}F NMR (565 MHz, $CDCl_3$) δ -114.16 (ddt, J = 287.0, 55.9, 14.2 Hz), -117.72 (dddd, J = 287.1, 56.3, 21.8, 16.1 Hz), -133.78 – -133.92 (m), -135.28 – -135.53 (m).

HRMS (ESI) (m/z): calcd for $C_{22}H_{18}F_6O_6S$ ($[M + H]^+$), 538.0671; found, 538.0680.

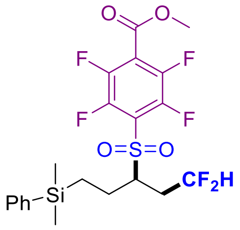

methyl-4-((5-(dimethyl(phenyl)silyl)-1,1-difluoropentan-3-yl)sulfonyl)-2,3,5,6-tetrafluorobenzoate (38)

This compound was obtained in 65% (66.6 mg) yield as a yellow oil by the general procedure.

^{1}H NMR (500 MHz, $CDCl_3$) δ 7.40 (dd, J = 7.5, 2.0 Hz, 2H), 7.36 – 7.29 (m, 3H), 6.10 (tdd, J = 56.0, 5.0, 3.5 Hz, 1H), 4.04 (s, 3H), 3.34 (tt, J = 8.5, 5.0 Hz, 1H), 2.58 – 2.41

(m, 1H), 2.29 (m, 1H), 1.88 – 1.77 (m, 1H), 1.76 – 1.63 (m, 1H), 0.99 (td, J = 13.5, 4.5 Hz, 1H), 0.71 (td, J = 13.5, 4.5 Hz, 1H), 0.29 (d, J = 1.5 Hz, 6H).
^{13}C NMR (151 MHz, $CDCl_3$) δ 158.58, 144.52 (dm, J = 261.9 Hz), 137.21, 133.32, 129.37, 127.93, 119.77 (t, J = 14.5 Hz), 118.04 (t, J = 17.0 Hz), 114.52 (t, J = 240.5 Hz), 63.22 (t, J = 4.5 Hz), 53.95, 31.24 (t, J = 23.6 Hz), 23.31, 12.10, -3.40, -3.74.
^{19}F NMR (565 MHz, $CDCl_3$) δ -114.26 (ddt, J = 286.5, 56.1, 14.2 Hz), -117.47 (dddd, J = 286.5, 56.3, 21.3, 15.5 Hz), -134.17 – -134.39 (m), -135.14 – -135.34 (m).
HRMS (ESI) (m/z): calcd for $C_{21}H_{22}F_6NaO_4SSi$ ($[M + Na]^+$), 535.0804; found, 535.0794.

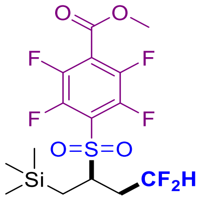

methyl-4-((4,4-difluoro-1-(trimethylsilyl)butan-2-yl)sulfonyl)-2,3,5,6-tetrafluoro-benzoate (39)

This compound was obtained in 63% (55.0 mg) yield as a yellow oil by the general procedure.
^{1}H NMR (500 MHz, $CDCl_3$) δ 6.22 (tdd, J = 56.5, 7.0, 3.0 Hz, 1H), 4.02 (s, 3H), 3.54 (dd, J = 12.0, 8.5 Hz, 1H), 2.60 – 2.43 (m, 1H), 2.26 – 2.10 (m, 1H), 1.11 (dt, J = 14.5, 2.0 Hz, 1H), 0.96 (dd, J = 14.5, 12.0 Hz, 1H), 0.09 (s, 9H).
^{13}C NMR (151 MHz, $CDCl_3$) δ 158.62, 145.98 (dm, J = 241.5 Hz), 119.43 (t, J = 14.9 Hz), 118.12 (t, J = 16.9 Hz), 114.50 (t, J = 240.0 Hz), 59.45, 53.95, 34.42 (t, J = 24.0 Hz), 16.34, 1.25.
^{19}F NMR (565 MHz, $CDCl_3$) δ -113.28 (dddd, J = 288.5, 55.7, 14.0, 9.4 Hz), -118.15 (dddd, J = 288.5, 57.0, 25.8, 14.5 Hz), -133.61 – -133.77 (m), -135.24 – -135.43 (m).
HRMS (ESI) (m/z): calcd for $C_{15}H_{19}F_6O_4SSi$ ($[M + H]^+$), 437.0599; found, 437.0603.

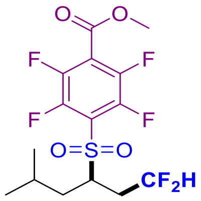

methyl-4-((1,1-difluoro-5-methylhexan-3-yl)sulfonyl)-2,3,5,6-tetrafluorobenzoate (40)

This compound was obtained in 57% (46.3 mg) yield as colorless oil by the general procedure.
^{1}H NMR (500 MHz, $CDCl_3$) δ 6.16 (tdd, J = 56.5, 5.5, 3.5 Hz, 1H), 4.02 (s, 3H), 3.50 (dt, J = 8.5, 4.0 Hz, 1H), 2.58 – 2.42 (m, 1H), 2.26 – 2.14 (m, 1H), 1.87 – 1.70 (m, 1H), 1.72 – 1.58 (m, 2H), 0.96 (d, J = 6.5 Hz, 3H), 0.85 (d, J = 6.5 Hz, 3H).
^{13}C NMR (151 MHz, $CDCl_3$) δ 158.60, 144.73 (dm, J = 262.7 Hz), 119.65 (t, J = 14.5 Hz), 118.20 (t, J = 17.0 Hz), 114.46 (t, J = 240.4 Hz), 59.54 (t, J = 5.0 Hz), 53.97, 37.23, 32.80 (t, J = 24.0 Hz), 25.13, 22.92, 20.97.
^{19}F NMR (565 MHz, $CDCl_3$) δ -114.15 (ddt, J = 287.4, 56.1, 14.0 Hz), -117.78 (dddd, J = 287.3, 56.2, 22.5, 15.1 Hz), -134.04 – -134.19 (m), -135.23 – -135.40 (m).

HRMS (ESI) (m/z): calcd for $C_{15}H_{16}F_6NaO_4S$ ([M + Na]$^+$), 429.0565; found, 429.0573.

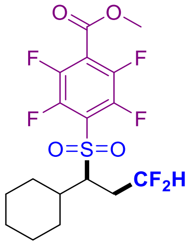

methyl-4-((1-cyclohexyl-3,3-difluoropropyl)sulfonyl)-2,3,5,6-tetrafluorobenzoate (41)

This compound was obtained in 68% (58.8 mg) yield as a yellow oil by the general procedure.

^{1}H NMR (500 MHz, $CDCl_3$) δ 6.08 (tdd, J = 56.5, 5.5, 3.0 Hz, 1H), 4.01 (s, 3H), 3.32 (dt, J = 10.0,5.0 Hz, 1H), 2.60 – 2.47 (m, 1H), 2.38 – 2.24 (m, 1H), 2.14 – 2.01 (m, 2H), 1.83 – 1.74 (m, 2H), 1.68 (d, J = 12.9 Hz, 1H), 1.55 (d, J = 10.0 Hz, 1H), 1.35 – 1.20 (m, 3H), 1.18 – 1.08 (m, 2H).

^{13}C NMR (151 MHz, $CDCl_3$) δ 158.68, 144.7 (dm, J = 265.7 Hz), 120.86 (t, J = 14.5 Hz), 117.95 (t, J = 17.0 Hz), 114.78 (t, J = 240.5 Hz), 65.94 (d, J = 5.5 Hz), 53.92, 37.70, 31.21, 29.28 (t, J = 23.5 Hz), 27.52, 26.38, 25.79, 25.69.

^{19}F NMR (565 MHz, $CDCl_3$) δ -114.66 (ddt, J = 285.2, 56.2, 14.4 Hz), -117.88 (dddd, J = 285.1, 56.5, 22.2, 17.0 Hz), -134.75 – -134.89 (m), -135.53 – -135.65 (m).

HRMS (ESI) (m/z): calcd for $C_{17}H_{19}F_6O_4S$ ([M + H]$^+$), 433.0910; found, 433.0908.

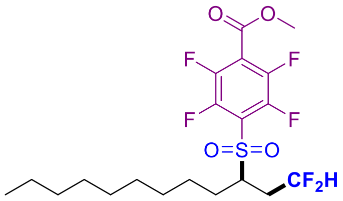

methyl-4-((1,1-difluorododecan-3-yl)sulfonyl)-2,3,5,6-tetrafluorobenzoate (42)

This compound was obtained in 40% (38.1 mg) yield as colorless oil by the general procedure.

^{1}H NMR (500 MHz, $CDCl_3$) δ 6.15 (tdd, J = 56.3, 5.3, 3.6 Hz, 1H), 4.03 (s, 3H), 3.44 (dt, J = 10.0, 5.0 Hz, 1H), 2.57 – 2.45 (m, 1H), 2.26 (m, 1H), 1.97 – 1.85 (m, 1H), 1.78 – 1.67 (m, 1H), 1.57 – 1.46 (m, 1H), 1.43 – 1.32 (m, 1H), 1.32 – 1.21 (m, 12H), 0.87 (t, J = 6.9 Hz, 3H).

^{13}C NMR (151 MHz, $CDCl_3$) δ 158.60, 144.38 (dm, J = 259.7 Hz), 119.81 (t, J = 15.0 Hz), 118.17 (t, J = 17.0 Hz), 114.47 (t, J = 240.6 Hz), 61.11 (t, J = 4.8 Hz), 53.97, 32.15 (t, J = 23.5 Hz), 31.79, 29.34, 29.18, 29.12, 29.04, 28.32, 25.96, 22.63, 14.06.

^{19}F NMR (565 MHz, $CDCl_3$) δ -114.48 (ddt, J = 286.7, 56.1, 14.5 Hz), -117.62 (dddd, J = 287.0, 56.6, 21.4, 15.6 Hz), -133.83 – -134.55 (m), -134.94 – -135.83 (m).

HRMS (ESI) (m/z): calcd for $C_{20}H_{26}F_6NaO_4S$ ([M + Na]$^+$), 499.1354; found, 499.1360.

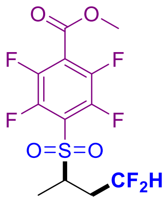

methyl-4-((4,4-difluorobutan-2-yl)sulfonyl)-2,3,5,6-tetrafluorobenzoate (43)

This compound was obtained in 64% (46.6 mg) yield as colorless oil by the general procedure.

^{1}H NMR (500 MHz, $CDCl_3$) δ 6.09 (tt, J = 55.5, 4.0 Hz, 1H), 4.03 (s, 3H), 3.60 (td, J = 10.0, 5.0 Hz, 1H), 2.67 – 2.51 (m, 1H), 2.22 – 2.10 (m,1H), 1.50 (d, J = 8.0 Hz, 3H).

^{13}C NMR (151 MHz, $CDCl_3$) δ 158.57, 146.00 – 145.39 (m), 144.25 – 143.65 (m), 119.06 (t, J = 19.2 Hz), 118.29 (d, J = 17.1 Hz), 114.46 (t, J = 241.3 Hz), 56.73 (t, J = 4.7 Hz), 53.99, 33.34 (t, J = 22.9 Hz), 13.96.

^{19}F NMR (565 MHz, $CDCl_3$) δ -114.59 (ddt, J = 286.4, 56.0, 16.7 Hz), -117.58 (dd, J = 286.2, 56.0 Hz), -133.78 – -134.04 (m), -136.37 – -136.67 (m).

HRMS (ESI) (m/z): calcd for $C_{12}H_{10}F_6NaO_4S$ ([M + Na]$^+$), 387.0096; found, 387.0085.

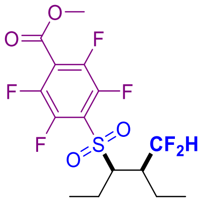

methyl 4-(4-(difluoromethyl)hexan-3-yl)sulfonyl)-2,3,5,6-tetrafluorobenzoate (44)

This compound was obtained in 51% (41.4 mg) yield as colorless oil by the general procedure.

^{1}H NMR (500 MHz, $CDCl_3$) δ 6.29 (tdd, J = 57.0, 55.5, 4.5 Hz, 1H), 5.90 (td, J = 56.0, 3.5 Hz, 1H), 4.07 (s, 6H), 3.54 – 3.47 (m, 1H), 3.43 – 3.34 (m, 1H), 2.49 – 2.29 (m, 2H), 2.15 – 2.03 (m, 1H), 2.01 – 1.83 (m, 5H), 1.80 – 1.66 (m, 1H), 1.64 – 1.52 (m, 1H), 1.12 – 0.99 (m, 12H).

^{13}C NMR (151 MHz, $CDCl_3$) δ 158.69, 158.63, 145.78 – 145.30 (m), 144.15 – 143.52 (m), 121.71 – 120.86 (m), 117.96 (q, J = 16.8 Hz), 116.55, (t, J = 241.3 Hz), 116.10 (t, J = 241.7 Hz), 67.66 – 67.43 (m), 65.89 (d, J = 5.2 Hz), 53.94, 44.47 (t, J = 20.8 Hz), 43.14 (t, J = 18.6 Hz), 19.42, 18.27 (d, J = 3.6 Hz), 18.09 (d, J = 2.0 Hz), 17.78 (dd, J = 5.3, 2.4 Hz), 13.11 – 12.97 (m), 12.73 (d, J = 2.0 Hz), 12.41, 12.32.

^{19}F NMR (565 MHz, $CDCl_3$) δ -115.70 – -124.52 (m), -134.01 – -135.02 (m), -135.35 – -135.59 (m).

HRMS (ESI) (m/z): calcd for $C_{15}H_{16}F_6NaO_4S$ ([M + Na]$^+$), 429.0565; found, 429.0568.

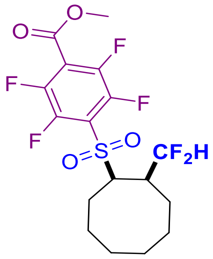

methyl 4-(-2-(difluoromethyl)cyclooctyl)sulfonyl)-2,3,5,6-tetrafluorobenzoate (45)

This compound was obtained in 60% (51.9 mg) yield as colorless oil by the general procedure.

^{1}H NMR (500 MHz, $CDCl_3$) δ 5.60 (tdd, J = 56.5, 10.0, 3.5 Hz, 2H), 4.03 (s, 6H), 3.38 (tt, J = 9.5, 3.0 Hz, 1H), 3.29 (tt, J = 8.8, 4.4 Hz, 1H), 2.38 – 2.27 (m, 1H), 2.26 – 2.15 (m, 1H), 2.05 – 1.71 (m, 16H), 1.63 – 1.29 (m, 7H).

^{13}C NMR (151 MHz, $CDCl_3$) δ 158.74, 145.89 – 145.34 (m), 144.16 – 143.61 (m), 120.49 – 120.07 (m), 118.80 (t, J = 241.5 Hz), 118.66 (t, J = 241.0 Hz), 117.76 (t, J = 17.1 Hz), 117.18, 117.05, 66.26, 65.61, 53.94, 41.52 (t, J = 18.7 Hz), 41.10 (t, J = 18.5 Hz), 25.19 (d, J = 5.1 Hz), 25.08 – 24.85 (m), 24.60 (d, J = 2.2 Hz), 24.36, 24.24 (t, J = 4.7 Hz), 22.77 (t, J = 4.6 Hz), 22.68.
^{19}F NMR (565 MHz, $CDCl_3$) δ -121.73 – -123.83 (m), -133.95 – -134.33 (m), -135.37 – -135.87 (m).
HRMS (ESI) (m/z): calcd for $C_{17}H_{18}F_6NaO_4S$ ($[M + Na]^+$), 455.0722; found, 455.0734.

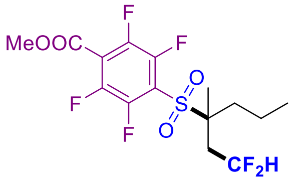

methyl 4-((1,1-difluoro-3-methylhexan-3-yl)sulfonyl)-2,3,5,6-tetrafluorobenzoate (46)
This compound was obtained in 48% yield (38.8 mg) as a colorless oil by the general procedure.
^{1}H NMR (600 MHz, $CDCl_3$) δ 6.33 (tt, J = 55.8, 4.7 Hz, 1H), 4.04 (s, 3H), 2.55 – 2.37 (m, 1H), 2.36 – 2.25 (m, 1H), 1.90 (td, J = 13.3, 4.0 Hz, 1H), 1.70 (dd, J = 13.1, 4.5 Hz, 1H), 1.54 – 1.50 (m, 1H), 1.45 (s, 3H), 1.43 – 1.38 (m, 1H), 0.98 (t, J = 7.2 Hz, 3H).
^{13}C NMR (151 MHz, $CDCl_3$) δ 158.65, 114.68 (t, J = 239.6 Hz), 67.35, 54.01, 37.00 (t, J = 23.8 Hz), 35.58, 29.71, 19.13, 17.00, 14.29.
^{19}F NMR (565 MHz, $CDCl_3$) δ -110.30 – -112.40 (m), -130.05 – -130.48 (m), -135.07 – -135.51 (m).
HRMS (ESI) (m/z): calcd for $C_{15}H_{16}F_6NaO_4S$ ([M + Na]+), 429.0566; found, 429.0567.

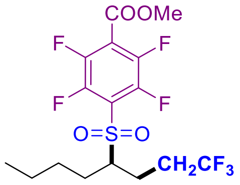

methyl (R)-2,3,5,6-tetrafluoro-4-((1,1,1-trifluorooctan-4-yl)sulfonyl)benzoate (47)
This compound was obtained in 65% (56.9 mg) yield as a colorless oil by the general procedure.
^{1}H NMR (600 MHz, $CDCl_3$) δ 3.96 (s, 3H), 3.50 (tt, J = 6.4, 4.0 Hz, 1H), 2.62 – 2.46 (m, 1H), 2.27 – 2.10 (m, 1H), 2.08 – 1.96 (m, 1H), 1.79 – 1.72 (m, 1H), 1.61 (s, 1H), 1.57 – 1.53 (m, 1H), 1.52 – 1.42 (m, 1H), 1.41 – 1.35 (m, 1H), 1.32 – 1.24 (m, 2H), 0.84 (t, J = 7.4 Hz, 3H).
^{13}C NMR (151 MHz, $CDCl_3$) δ 157.73, 145.12 – 144.14 (m), 143.51 – 142.28 (m), 121.20 (t, J = 240.0 Hz), 119.48 (d, J = 14.8 Hz), 116.84 (t, J = 17.0 Hz), 60.22, 52.87, 35.10 (t, J = 25.4 Hz), 27.38, 27.09, 23.13 (td, J = 27.1, 3.5 Hz), 21.39, 12.61 (d, J = 3.4 Hz).
^{19}F NMR (565 MHz, $CDCl_3$) δ -90.29 – -92.45 (m), -134.54 (dt, J = 20.6, 8.9 Hz), -135.89 (dt, J = 21.5, 10.1 Hz).
HRMS (ESI) (m/z): calcd for $C_{16}H_{17}F_7NaO_4S$ ($[M + Na]^+$), 461.0628; found, 461.0639.

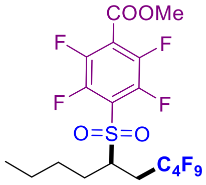

methyl (R)-2,3,5,6-tetrafluoro-4-((10,10,10,10,10,10,10,10,10-nonafluoro-10-deca-7,9-diyn-5-yl)-sulfonyl)benzoate (48)

This compound was obtained in 55% (63.1 mg) yield as a colorless oil by the general procedure.

^{1}H NMR (600 MHz, $CDCl_3$) δ 4.04 (s, 3H), 3.60 (tdd, J = 6.9, 4.9, 3.5 Hz, 1H), 2.95 – 2.85 (m, 1H), 2.48 (ddt, J = 30.4, 16.6, 7.1 Hz, 1H), 2.13 (ddt, J = 16.0, 10.8, 5.4 Hz, 1H), 1.94 – 1.81 (m, 1H), 1.56 – 1.49 (m, 1H), 1.48 – 1.40 (m, 1H), 1.39 – 1.33 (m, 2H), 0.92 (t, J = 7.3 Hz, 3H).

^{13}C NMR (151 MHz, $CDCl_3$) δ 158.53, 146.87 – 144.82 (m), 144.27 – 143.26 (m), 121.02 – 119.39 (m), 119.03 – 117.82 (m), 59.73, 53.99, 29.32 (t, J = 21.7 Hz), 28.40, 27.89, 22.31, 13.56.

^{19}F NMR (565 MHz, $CDCl_3$) δ -80.97 (t, J = 9.9 Hz), -111.50 – -113.67 (m), -124.17 (dt, J = 41.5, 7.7 Hz), -125.81 (t, J = 12.9 Hz), -133.77 – -134.54 (m), -134.88 – -135.61 (m).

HRMS (ESI) (m/z): calcd for $C_{18}H_{15}F_{13}NaO_4S$ ($[M + Na]^+$), 597.0376; found, 597.0383.

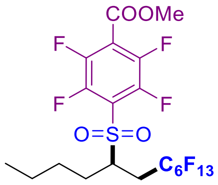

methyl (R)-2,3,5,6-tetrafluoro-4-((12,12,12,12,12,12,12,12,12,12,12,12,12-tridecafluoro-12-dodeca-7,9,11-triyn-5-yl)sulfonyl)benzoate (49)

This compound was obtained in 37% (49.8 mg) yield as a colorless oil by the general procedure.

^{1}H NMR (600 MHz, $CDCl_3$) δ 4.04 (s, 3H), 3.60 (tdd, J = 6.8, 4.9, 3.4 Hz, 1H), 2.99 – 2.83 (m, 1H), 2.55 – 2.42 (m, 1H), 2.21 – 2.10 (m, 1H), 1.95 – 1.82 (m, 1H), 1.53 (dd, J = 13.2, 5.9 Hz, 1H), 1.49 – 1.40 (m, 1H), 1.40 – 1.33 (m, 2H), 0.93 (t, J = 7.3 Hz, 3H).

^{13}C NMR (151 MHz, $CDCl_3$) δ 158.53, 145.97 – 145.24 (m), 144.27 – 143.38 (m), 119.84, 118.38, 59.74, 53.98, 29.58 (d, J = 37.8 Hz), 28.38, 27.89, 22.30, 13.55.

^{19}F NMR (565 MHz, $CDCl_3$) δ -80.75 (t, J = 10.1 Hz), -111.27 – -113.64 (m), -121.36 – -122.03 (m), -122.40 – -122.84 (m), -122.92 – -123.51 (m), -126.10 (t, J = 14.8 Hz), -133.57 – -134.54 (m), -134.95 – -135.53 (m).

HRMS (ESI) (m/z): calcd for $C_{20}H_{15}F_{17}NaO_4S$ ($[M + Na]^+$), 697.0312; found, 697.0305.

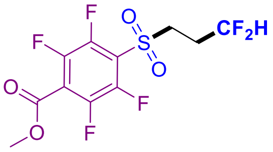

methyl 4-((3,3-difluoropropyl)sulfonyl)-2,3,5,6-tetrafluorobenzoate (50)

This compound was obtained in 91% (63.7 mg) yield as yellow oil liquid by the general procedure.

^{1}H NMR (500 MHz, $CDCl_3$) δ 6.06 (tt, J = 55.5, 3.5 Hz, 1H), 4.02 (s, 3H), 3.57 – 3.54 (m, 2H), 2.48 – 2.38 (m, 2H).

^{13}C NMR (151 MHz, $CDCl_3$) δ 158.53, 144.83 (dm, J = 262.7 Hz), 120.55 (t, J = 14.5 Hz), 118.24 (t, J = 17.0 Hz), 114.01 (t, J = 243.0 Hz), 54.01, 50.60 (t, J = 5.5 Hz), 27.03 (t, J = 23.0 Hz).

^{19}F NMR (565 MHz, $CDCl_3$) δ -117.66 (dt, J = 56.0, 16.5 Hz), -134.77 – -134.87 (m), -135.13 – -135.24 (m).

HRMS (ESI) (m/z): calcd for $C_{11}H_8F_6NaO_4S$ ([M + Na]$^+$), 372.9940; found, 372.9947.

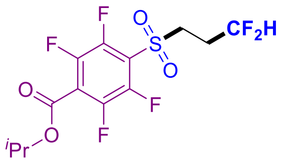

isopropyl 4-((3,3-difluoropropyl)sulfonyl)-2,3,5,6-tetrafluorobenzoate (51)

This compound was obtained in 92% (69.6 mg) yield as yellow oil liquid by the general procedure.

^{1}H NMR (500 MHz, $CDCl_3$) δ 6.07 (tt, J = 55.5, 3.5 Hz, 1H), 5.37 – 5.30 (m, 1H), 3.76 – 3.50 (m, 2H), 2.65 – 2.23 (m, 2H), 1.39 (d, J = 10.0 Hz, 6H).

^{13}C NMR (151 MHz, $CDCl_3$) δ 157.50, 144.47 (dm, J = 259.7 Hz), 120.12 (t, J = 10.5 Hz), 119.22 (t, J = 16.6 Hz), 113.85 (t, J = 241.2 Hz), 72.29, 50.40 (t, J = 5.5 Hz),26.78 (t, J = 23.0 Hz), 21.63.

^{19}F NMR (565 MHz, $CDCl_3$) δ -117.65 (dt, J = 55.8, 17.2 Hz), -134.96 – -135.19 (m), -135.98 – -136.25 (m).

HRMS (ESI) (m/z): calcd for $C_{13}H_{12}F_6NaO_4S$ ([M + Na]$^+$), 401.0253; found, 401.0258.

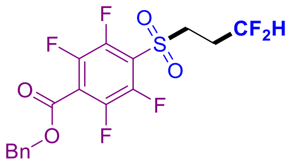

benzyl 4-((3,3-difluoropropyl)sulfonyl)-2,3,5,6-tetrafluorobenzoate (52)

This compound was obtained in 89% (75.8 mg) yield as yellow oil liquid by the general procedure.

^{1}H NMR (500 MHz, $CDCl_3$) δ 7.42 – 7.37 (m, 5H), 6.05 (tt, J = 55.5, 3.5 Hz, 1H), 5.43 (s, 2H), 3.55 – 3.52 (m, 2H), 2.46 – 2.35 (m, 2H).

^{13}C NMR (151 MHz, $CDCl_3$) δ 157.92, 144.7 (dm, J = 259.7 Hz), 134.01, 129.00, 128.81, 128.51, 120.53 (t, J = 13.6 Hz), 118.22 (t, J = 16.6 Hz), 114.06 (t, J = 241.2 Hz), 72.29, 50.59 (t, J = 5.2 Hz), 27.04 (t, J = 23.1 Hz).

^{19}F NMR (565 MHz, CDCl) δ -117.64 (dt, J = 55.5, 17.0 Hz), -134.80 – -134.93 (m), -135.08 – -135.21 (m).

HRMS (ESI) (m/z): calcd for $C_{17}H_{12}F_6NaO_4S$ ([M + Na]$^+$), 449.0253; found, 449.0249.

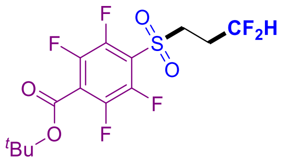

tert-butyl 4-((3,3-difluoropropyl)sulfonyl)-2,3,5,6-tetrafluorobenzoate (53)

This compound was obtained in 90% (70.6 mg) yield as yellow oil liquid by the general procedure.

^{1}H NMR (500 MHz, $CDCl_3$) δ 6.05 (tt, J = 55.5, 3.5 Hz, 1H), 3.57 – 3.48 (m, 2H), 2.46 – 2.35 (m, 2H), 1.60 (s, 9H).

^{13}C NMR (151 MHz, $CDCl_3$) δ 156.84, 144.37 (dm J = 261.3 Hz), 120.24 (t, J = 18.0 Hz), 119.58 (t, J = 14.5 Hz), 114.10 (t, J = 241.5 Hz), 86.45, 50.55 (t, J = 5.5 Hz), 28.01, 27.08 (t, J = 23.5 Hz).

^{19}F NMR (565 MHz, $CDCl_3$) δ -117.66 (dt, J = 55.5, 17.0 Hz), -135.10 – -135.31 (m), -136.95 – -137.12 (m).

HRMS (ESI) (m/z): calcd for $C_{14}H_{14}F_6NaO_4S$ ($[M + Na]^+$), 415.0409; found, 415.0415.

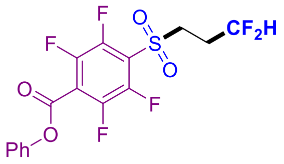

phenyl 4-((3,3-difluoropropyl)sulfonyl)-2,3,5,6-tetrafluorobenzoate (54)

This compound was obtained in 51% (42.1 mg) yield as yellow oil liquid by the general procedure.

^{1}H NMR (500 MHz, $CDCl_3$) δ 7.46 (t, J = 10.0 Hz, 2H), 7.34 (t, J = 5.0 Hz, 1H), 7.28 – 7.23 (m, 2H), 6.07 (tt, J = 55.7, 3.6 Hz 1H), 3.76 – 3.32 (m, 2H), 2.68 – 2.28 (m, 2H).

^{13}C NMR (151 MHz, $CDCl_3$) δ 156.55, 149.83, 144.93 (dm J = 264.7 Hz), (m), 129.87, 127.11, 121.06, 117.92 (t, J = 14.5 Hz), 114.01 (t, J = 241.4 Hz), 109.90 (d, J = 14.5 Hz), 50.66, 27.07.

^{19}F NMR (565 MHz, $CDCl_3$) δ -117.62 (dt, J = 55.7, 17.2 Hz), -134.32 – -134.45 (m), -134.49 – -134.64 (m).

HRMS (ESI) (m/z): calcd for $C_{16}H_{10}F_6NaO_4S$ ($[M + Na]^+$), 435.2922; found, 415.2937.

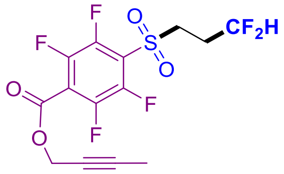

but-2-yn-1-yl 4-((3,3-difluoropropyl)sulfonyl)-2,3,5,6-tetrafluorobenzoate (55)

This compound was obtained in 73% (56.7 mg) yield as yellow oil liquid by the general procedure.

^{1}H NMR (500 MHz, $CDCl_3$) δ 6.05 (tt, J = 55.5, 3.5 Hz, 1H), 4.96 (q, J = 2.4 Hz, 2H), 3.60 – 3.48 (m, 2H), 2.47 – 2.37 (m, 2H), 1.88 (t, J = 2.4 Hz, 3H).

^{13}C NMR (151 MHz, $CDCl_3$) δ 157.56, 144.67 (dm J = 264.3 Hz), 124.58 (t, J = 10.5 Hz), 117.85 (t, J = 15.0 Hz), 114.04 (t, J = 241.1 Hz), 85.29, 71.41, 55.66, 50.57 (t, J = 5.5 Hz), 27.04 (t, J = 23.0 Hz), 3.67.

^{19}F NMR (565 MHz, $CDCl_3$) δ -117.67 (dt, J = 55.5, 17.0 Hz), -134.71 – -134.88 (m).

HRMS (ESI) (m/z): calcd for $C_{14}H_{10}F_6NaO_4S$ ($[M + Na]^+$), 411.0096; found, 411.0097.

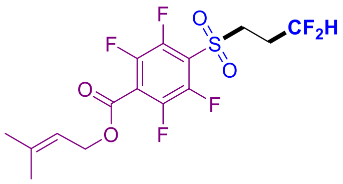

3-methylbut-2-en-1-yl 4-((3,3-difluoropropyl)sulfonyl)-2,3,5,6-tetrafluorobenzoate (56)

This compound was obtained in 49% (39.6 mg) yield as yellow oil liquid by the general procedure.

^{1}H NMR (500 MHz, CDCl3) δ 6.06 (tt, J = 55.5, 3.5 Hz, 1H), 5.43 (s, 1H), 4.90 (d, J = 10.0 Hz, 2H), 3.58 – 3.52 (m, 2H), 2.51 – 2.35 (m, 2H), 1.78 (d, J = 15.0 Hz, 6H).

^{13}C NMR (151 MHz, CDCl3) δ 158.05, 144.63 (dm, J = 264.8 Hz), 142.03, 116.79, 115.62, 114.02 (t, J = 241.5 Hz), 64.33, 50.57, 27.08 (t, J = 23.0 Hz), 25.82, 18.15.

^{19}F NMR (565 MHz, CDCl3) δ -117.64 (dt, J = 55.8, 17.4 Hz), -134.87 – -134.99 (m), -135.37 – -135.48 (m).

HRMS (ESI) (m/z): calcd for $C_{15}H_{14}F_6NaO_4S$ ([M + Na]$^+$), 427.0415; found, 427.0414.

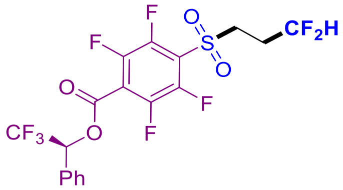

(S)-2,2,2-trifluoro-1-phenylethyl-4-((3,3-difluoropropyl)sulfonyl)-2,3,5,6-tetrafluorobenzoate (57)

This compound was obtained in 45% (44.5 mg) yield as yellow oil liquid by the general procedure.

^{1}H NMR (500 MHz, CDCl3) δ 7.53 – 7.41 (m, 5H), 6.34 (q, J = 6.5 Hz, 1H), 6.04 (tt, J = 55.5, 3.5 Hz, 1H), 3.59 – 3.51 (m, 2H), 2.50 – 2.35 (m, 2H).

^{13}C NMR (151 MHz, CDCl3) δ 156.22, 144.91 (dm, J = 261.6 Hz), 130.61, 129.50, 129.00, 128.17, 123.37 (q, J = 283.1 Hz), 116.42 (t, J = 15.4 Hz), 114.00 (t, J = 241.3 Hz), 74.44 (q, J = 34.1 Hz), 50.63 (t, J = 5.5 Hz), 27.01 (t, J = 23.1 Hz).

^{19}F NMR (565 MHz, CDCl3) δ -75.92 (d, J = 6.7 Hz), -117.67 (dt, J = 55.8, 16.9 Hz), -133.86 – -133.99 (m), -134.17 – -134.34 (m).

HRMS (ESI) (m/z): calcd for $C_{18}H_{12}F_9O_4S$ ([M + H]$^+$), 495.0307; found, 495.0302.

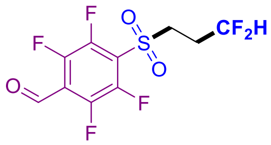

4-((3,3-difluoropropyl)sulfonyl)-2,3,5,6-tetrafluorobenzaldehyde (58)

This compound was obtained in 81% (51.8 mg) yield as yellow oil liquid by the general procedure.

^{1}H NMR (500 MHz, CDCl3) δ 10.34 (s, 1H), 6.06 (tt, J = 55.5, 3.5 Hz, 1H), 3.60 – 3.57 (m, 2H), 2.57 – 2.36 (m, 2H).

^{13}C NMR (151 MHz, CDCl3) δ 181.07, 146.74 (dm, J = 282.4 Hz), 144.78 (dm, J = 272.3 Hz), 122.98 (t, J = 15.1 Hz), 119.01 (t, J = 9.9 Hz), 113.97 (t, J = 241.3 Hz),

50.63, 26.98 (t, J = 23.0 Hz).
^{19}F NMR (565 MHz, $CDCl_3$) δ -117.66 (dt, J = 55.8, 16.9 Hz), -134.63 – -134.91 (m), -140.72 – -141.23 (m).
HRMS (ESI) (m/z): calcd for $C_{10}H_6F_6NaO_3S$ ([M + Na]$^+$), 342.9834; found, 342.9826.

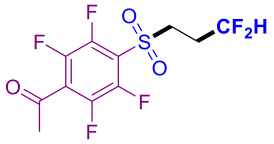

1-(4-((3,3-difluoropropyl)sulfonyl)-2,3,5,6-tetrafluorophenyl)ethan-1-one (59)
This compound was obtained in 79% (52.8 mg) yield as yellow oil liquid by the general procedure.
^{1}H NMR (500 MHz, $CDCl_3$) δ 6.06 (tt, J = 55.5, 3.5 Hz, 1H), 3.59 – 3.53 (m, 2H), 2.66 (s, 3H), 2.43 (m, 2H).
^{13}C NMR (151 MHz, $CDCl_3$) δ 190.36, 144.83 (dm, J = 263.5 Hz), 143.25 (dm, J = 261.3 Hz), 124.94, 120.14, 114.03 (t, J = 241.6 Hz), 50.60, 32.25, 27.05 (t, J = 23.1 Hz).
^{19}F NMR (565 MHz, $CDCl_3$) δ -117.63 (dt, J = 55.5, 16.9 Hz), -134.38 – -134.46 (m), -137.90 – -134.97 (m).
HRMS (ESI) (m/z): calcd for $C_{11}H_8F_6NaO_3S$ ([M + Na]$^+$), 356.9991; found, 356.9997.

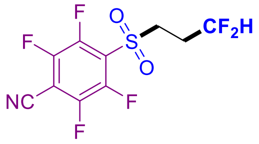

4-((3,3-difluoropropyl)sulfonyl)-2,3,5,6-tetrafluorobenzonitrile (60)
This compound was obtained in 61% (38.7 mg) yield as yellow oil liquid by the general procedure.
^{1}H NMR (500 MHz, $CDCl_3$) δ 6.07 (tt, J = 55.5, 3.5 Hz, 1H), 3.76 – 3.50 (m, 2H), 2.65 – 2.23 (m, 2H).
^{13}C NMR (151 MHz, $CDCl_3$) δ 147.44 (dm, J = 255.3 Hz), 144.70 (dm, J = 251.3 Hz), 123.90, 113.85 (t, J = 241.5 Hz), 105.90 (t, J = 3.5 Hz), 99.91 (d, J = 16.8 Hz), 50.78 (t, J = 5.5 Hz), 26.92 (t, J = 23.1 Hz).
^{19}F NMR (565 MHz, $CDCl_3$) δ -117.69 (dt, J = 56.0, 17.0 Hz), -127.30 – -127.55 (m), -132.54 – -132.87 (m).
HRMS (ESI) (m/z): calcd for $C_{10}H_5F_6NNaO_2S$ ([M + Na]$^+$), 339.9837; found, 339.9840.

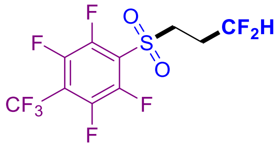

1-((3,3-difluoropropyl)sulfonyl)-2,3,5,6-tetrafluoro-4-(trifluoromethyl)benzene (61)
This compound was obtained in 77% (55.4 mg) yield as yellow oil liquid by the general procedure.
^{1}H NMR (500 MHz, $CDCl_3$) δ 6.05 (tt, J = 55.0, 3.5 Hz, 1H), 3.61 – 3.53 (m, 2H), 2.52

– 2.37 (m, 2H).

^{13}C NMR (151 MHz, $CDCl_3$) δ 144.65 (dm, J = 263.5 Hz), 122.09 (t, J = 14.5 Hz), 119.93 (q, J = 263.5 Hz), 115.44 – 114.79 (m), 113.97 (t, J = 241.2 Hz), 50.63 (t, J = 5.4 Hz), 26.90 (t, J = 23.2 Hz).

^{19}F NMR (565 MHz, $CDCl_3$) δ -56.95 (dt, J = 21.6, 21.0, 8.8 Hz), -117.65 – -117.92 (m), -133.78 – -134.00 (m), -135.40 – -135.72 (m).

HRMS (ESI) (m/z): calcd for $C_{10}H_5F_9NaO_2S$ ([M + Na]$^+$), 382.9759; found, 382.9754.

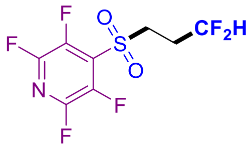

4-((3,3-difluoropropyl)sulfonyl)-2,3,5,6-tetrafluoropyridine (62)

This compound was obtained in 77% (45.2 mg) yield as yellow oil liquid by the general procedure.

^{1}H NMR (500 MHz, $CDCl_3$) δ 6.06 (tt, J = 55.5, 3.5 Hz, 1H), 3.62 – 3.59 (m, 2H), 2.57 – 2.36 (m, 2H).

^{13}C NMR (151 MHz, $CDCl_3$) δ 148.13 – 147.20 (m), 146.11 – 145.67 (m), 145.67 – 145.29 (m), 144.30 – 143.52 (m), 130.20, 113.97 (t, J = 241.3 Hz), 50.44, 26.98 (t, J = 23.0 Hz).

^{19}F NMR (565 MHz, $CDCl_3$) δ -84.31 – -84.61 (m), -117.72 (dt, J = 55.9, 16.9 Hz), -136.75 – -136.94 (m).

HRMS (ESI) (m/z): calcd for $C_8H_5F_6NNaO_2S$ ([M + Na]$^+$), 315.98371; found, 315.9844.

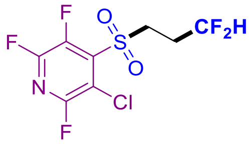

3-chloro-4-((3,3-difluoropropyl)sulfonyl)-2,5,6-trifluoropyridine (63)

This compound was obtained in 89% (55.1 mg) yield as yellow oil liquid by the general procedure.

^{1}H NMR (500 MHz, $CDCl_3$) δ 6.06 (tt, J = 56.0, 3.5 Hz, 1H), 3.59 – 3.53 (m, 2H), 2.49 – 2.40 (m, 2H).

^{13}C NMR (151 MHz, $CDCl_3$) δ 153.06 – 150.97 (m), 149.50 – 147.22 (m), 141.20 (ddd, J = 272.8, 28.6, 6.9 Hz), 138.46 (d, J = 10.5 Hz), 113.95 (t, J = 241.3 Hz), 113.43 – 112.93 (m), 49.59 (q, J = 3.1 Hz), 26.79 (t, J = 23.1 Hz).

^{19}F NMR (565 MHz, $CDCl_3$) δ -68.03 (ddd, J = 30.3, 17.7, 12.1 Hz), -82.71 (ddd, J = 23.1, 16.2, 11.7 Hz), -117.61 – -117.84 (m), -135.84 (ddd, J = 30.5, 22.5, 8.0 Hz).

HRMS (ESI) (m/z): calcd for $C_8H_5ClF_5NNaO_2S$ ([M + Na]$^+$), 331.9456; found, 331.9468.

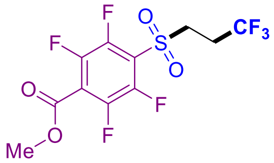

methyl 2,3,5,6-tetrafluoro-4-((3,3,3-trifluoropropyl)sulfonyl)benzoate (64)

This compound was obtained in 83% (61.1 mg) yield as yellow oil liquid by the general procedure.

^{1}H NMR (500 MHz, CDCl$_3$) δ 4.03 (s, 3H), 3.62 – 3.56 (m, 2H), 2.79 – 2.65 (m, 2H).

^{13}C NMR (151 MHz, CDCl$_3$) δ 158.45, 145.63, 143.94, 125.01 (q, J = 276.7 Hz), 120.23 (d, J = 13.8 Hz), 118.51, 54.06, 50.86, 27.39 (q, J = 32.1 Hz).

^{19}F NMR (565 MHz, CDCl$_3$) δ -65.77 (t, J = 9.6 Hz), -134.66 – -134.74 (m), -134.85 – -134.96 (m).

HRMS (ESI) (m/z): calcd for $C_{11}H_8F_7O_4S$ ([M + H]$^+$), 369.0026; found, 369.0019.

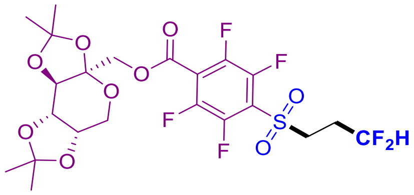

((3aR,5aS,8aS,8bR)-2,2,7,7-tetramethyltetrahydro-3aH-bis([1,3]dioxolo)[4,5-b:4',5'-d]pyran-3a-yl)methyl 4-((3,3-difluoropropyl)sulfonyl)-2,3,5,6-tetrafluoro-benzoate (65)

This compound was obtained in 64% (74.0 mg) yield as as white solid by the general procedure.

^{1}H NMR (500 MHz, CDCl$_3$) δ 6.06 (tt, J = 55.5, 3.5 Hz, 1H), 4.69 (d, J = 11.6 Hz, 1H), 4.63 (dd, J = 8.0, 2.5 Hz, 1H), 4.37 (d, J = 11.5 Hz, 1H), 4.32 (d, J = 2.5 Hz, 1H), 4.25 (dd, J = 8.0, 2.0 Hz, 1H), 3.92 (dd, J = 13.0, 2.0 Hz, 1H), 3.79 (d, J = 13.0 Hz, 1H), 3.59 – 3.54 (m, 2H), 2.52 – 2.36 (m, 2H), 1.54 (s, 3H), 1.45 (s, 3H), 1.33 (s, 3H), 1.32 (s, 3H).

^{13}C NMR (151 MHz, CDCl$_3$) δ 157.49, 151.68 – 150.88 (m), 145.96 – 144.95 (m), 144.71 – 143.18 (m), 114.00 (t, J = 271.5 Hz), 109.23 (d, J = 4.0 Hz), 100.78, 70.65 (d, J = 7.5 Hz), 69.91, 67.79, 61.50, 50.60, 27.04 (t, J = 23.0 Hz), 26.49, 25.81, 25.01, 24.05.

^{19}F NMR (565 MHz, CDCl$_3$) δ -117.64 (dt, J = 56.0, 16.5 Hz), -134.25 – -134.39 (m), -134.50 – -134.66 (m).

HRMS (ESI) (m/z): calcd for $C_{22}H_{24}F_6NaO_9S$ ([M + Na]$^+$), 601.0937; found, 601.0941.

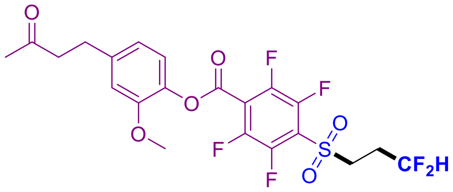

2-methoxy-4-(3-oxobutyl)phenyl 4-((3,3-difluoropropyl)sulfonyl)-2,3,5,6-tetra-fluorobenzoate (66)

This compound was obtained in 57% (58.4 mg) yield as as white solid by the general procedure.

^{1}H NMR (500 MHz, CDCl$_3$) δ 7.06 (d, J = 8.0 Hz, 1H), 6.86 (d, J = 2.0 Hz, 1H), 6.80 (dd, J = 8.0, 2.0 Hz, 1H), 6.06 (tt, J = 55.5, 3.5 Hz, 1H), 3.84 (s, 3H), 3.57 (dd, J = 9.0, 6.5 Hz, 2H), 2.90 (t, J = 7.5 Hz, 2H), 2.78 (t, J = 7.5 Hz, 2H), 2.51 – 2.39 (m, 2H), 2.15 (s, 3H).

^{13}C NMR (151 MHz, CDCl$_3$) δ 207.47, 150.34, 145.03 (dm, J = 263.5 Hz) 141.51,

137.03, 122.02, 120.76 (d, J = 14.6 Hz), 120.51, 114.53 (t, J = 241.4 Hz), 117.91 (d, J = 16.4 Hz), 114.06, 113.03, 55.98, 50.62 (t, J = 5.3 Hz), 44.99, 30.11, 29.56, 27.08 (t, J = 23.1 Hz).

^{19}F NMR (565 MHz, $CDCl_3$) δ -117.61 (dt, J = 55.9, 16.9 Hz), -134.14 – -134.26 (m), -134.70 – -134.87 (m).

HRMS (ESI) (m/z): calcd for $C_{21}H_{18}F_6NaO_6S$ ($[M + Na]^+$), 535.0620; found, 535.0628.

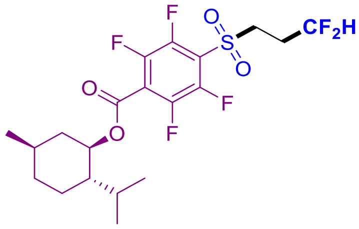

(1R,2S,5R)-2-isopropyl-5-methylcyclohexyl 4-((3,3-difluoropropyl)sulfonyl)-2,3,5,6-tetrafluoro-benzoate (67)

This compound was obtained in 50% (47.4 mg) yield as white solid by the general procedure.

^{1}H NMR (500 MHz, $CDCl_3$) δ 6.06 (tt, J = 55.5, 4.0 Hz, 1H), 5.02 (td, J = 11.0, 4.5 Hz, 1H), 3.54 (t, J = 7.5 Hz, 2H), 2.43 (ddd, J = 20.5, 10.0, 3.5 Hz, 2H), 2.15 (d, J = 11.5 Hz, 1H), 1.93 (ddd, J = 13.0, 7.5, 4.5 Hz, 1H), 1.73 (tt, J = 8.0, 5.0 Hz, 2H), 1.60 – 1.43 (m, 2H), 1.24 – 1.07 (m, 2H), 0.93 (dd, J = 18.0, 5.5 Hz, 7H), 0.80 (d, J = 7.0 Hz, 3H).

^{13}C NMR (151 MHz, $CDCl_3$) δ 157.69, 145.86 – 144.87 (m), 143.81 – 143.17 (m), 120.19 – 119.78 (m), 119.41 (t, J = 18.1 Hz), 114.06 (t, J = 241.3 Hz), 78.81, 50.57 (t, J = 4.6 Hz), 46.82, 40.53, 33.97, 31.53, 27.09 (t, J = 23.1 Hz), 25.99, 23.07, 21.93, 20.75, 15.86.

^{19}F NMR (565 MHz, $CDCl_3$) δ -117.64 (dt, J = 55.4, 17.0 Hz), -134.83 – -134.98 (m), -135.95 – -136.13 (m).

HRMS (ESI) (m/z): calcd for $C_{20}H_{24}F_6NaO_4S$ ($[M + Na]^+$), 497.1192; found, 497.1195.

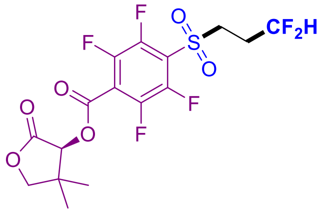

(S)-4,4-dimethyl-2-oxotetrahydrofuran-3-yl 4-((3,3-difluoropropyl)sulfonyl)-2,3,5,6-tetrafluoro-benzoate (68)

This compound was obtained in 60% (53.8 mg) yield as white solid by the general procedure.

^{1}H NMR (500 MHz, $CDCl_3$) δ 6.05 (tt, J = 55.5, 3.5 Hz, 1H), 5.59 (s, 1H), 4.11 (s, 2H), 3.69 – 3.48 (m, 2H), 2.56 – 2.32 (m, 2H), 1.29 (s, 3H), 1.18 (s, 3H).

^{13}C NMR (151 MHz, $CDCl_3$) δ 170.61, 157.17, 146.46 – 145.25 (m), 144.88 – 143.57 (m), 121.36 (t, J = 14.8 Hz), 116.56 (t, J = 15.8 Hz), 114.10 (t, J = 241.1 Hz), 77.66, 76.23, 50.66 (d, J = 5.0 Hz), 40.39, 27.03 (t, J = 23.2 Hz), 22.84, 19.92.

^{19}F NMR (565 MHz, $CDCl_3$) δ -117.64 (dt, J = 55.9, 16.9 Hz), -133.86 – -134.02 (m),

-134.41 – -134.67 (m).

HRMS (ESI) (m/z): calcd for $C_{16}H_{14}F_6NaO_6S$ ([M + Na]$^+$), 471.0307; found, 471.0312.

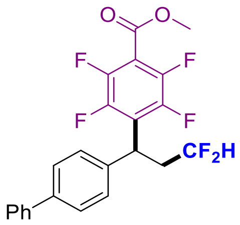

methyl (S)-4-(1-([1,1'-biphenyl]-4-yl)-3,3-difluoropropyl)-2,3,5,6-tetrafluorobenzoate (69)

This compound was obtained in 61% (53.4 mg) yield as yellow oil by the general procedure.

^{1}H NMR (600 MHz, $CDCl_3$) δ 7.56 (td, J = 8.2, 1.5 Hz, 4H), 7.47 – 7.40 (m, 4H), 7.38 – 7.33 (m, 1H), 5.81 (tt, J = 56.1, 4.5 Hz, 1H), 4.75 (t, J = 8.0 Hz, 1H), 3.96 (s, 3H), 2.94 – 2.76 (m, 2H).

^{13}C NMR (151 MHz, $CDCl_3$) δ 160.02, 144.97 (dm, J = 265.8 Hz), 140.92, 140.25, 137.88, 128.85, 128.15, 127.89, 127.86, 127.58, 127.07, 124.70 (t, J = 15.5 Hz), 118.07 – 113.60 (m), 111.34 (t, J = 15.8 Hz), 53.30, 36.59 (t, J = 21.6 Hz), 35.15 (t, J = 6.1 Hz).

^{19}F NMR (565 MHz, $CDCl_3$) δ -115.12 – -118.80 (m), -137.28 – -139.88 (m), -140.54 – -141.91 (m).

HRMS (ESI) (m/z): calcd for $C_{23}H_{17}F_6NaO_2$ ([M + Na]$^+$): 439.1127; found: 439.1128.

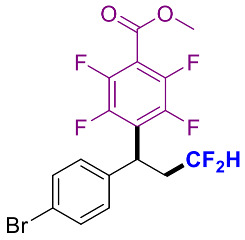

methyl (S)-4-(1-(4-bromophenyl)-3,3-difluoropropyl)-2,3,5,6-tetrafluorobenzoate (70)

This compound was obtained in 65% (57.1 mg) yield as yellow oil by the general procedure.

^{1}H NMR (600 MHz, $CDCl_3$) δ 7.32 (d, J = 8.5 Hz, 2H), 7.27 (d, J = 8.6 Hz, 2H), 5.78 (tt, J = 55.9, 4.5 Hz, 1H), 4.69 (t, J = 8.0 Hz, 1H), 3.96 (s, 3H), 2.79 (tdd, J = 15.6, 9.0, 4.7 Hz, 2H).

^{13}C NMR (151 MHz, $CDCl_3$) δ 159.91, 146.38 – 145.18 (m), 144.39 – 143.19 (m), 137.44, 133.87, 129.35, 128.83, 124.24 (t, J = 15.3 Hz), 115.46 (t, J = 240.6 Hz), 53.34, 36.57 (d, J = 21.1 Hz), 34.79.

^{19}F NMR (565 MHz, $CDCl_3$) δ -115.81 – -118.56 (m), -137.04 – -139.32 (m), -139.58 – -142.12 (m).

HRMS (ESI) (m/z): calcd for $C_{17}H_{12}BrF_6O_2$ ([M + H]$^+$): 440.9920; found: 440.9925.

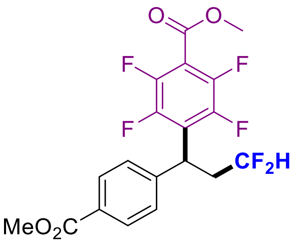

methyl (S)-4-(3,3-difluoro-1-(4-(methoxycarbonyl)phenyl)propyl)-2,3,5,6-tetra-fluorobenzoate (71)

This compound was obtained in 80% (67.2 mg) yield as yellow oil by the general procedure.

^{1}H NMR (500 MHz, $CDCl_3$) δ 8.00 (d, J = 8.4 Hz, 2H), 7.40 (d, J = 8.1 Hz, 2H), 5.79 (tt, J = 55.9, 4.4 Hz, 1H), 4.76 (t, J = 7.9 Hz, 1H), 3.95 (s, 3H), 3.90 (s, 3H), 2.89 – 2.74 (m, 2H).

^{13}C NMR (151 MHz, $CDCl_3$) δ 166.38, 159.86, 146.67 – 145.01 (m), 143.85, 130.41, 129.81, 127.52, 123.92 (t, J = 15.7 Hz), 115.42 (t, J = 240.4 Hz), 111.66 (d, J = 16.0 Hz), 53.32, 52.22, 36.34 (t, J = 23.1 Hz), 35.21 (t, J = 5.3 Hz).

^{19}F NMR (565 MHz, $CDCl_3$) δ -114.06 – -119.28 (m), -136.81 – -139.20 (m), -139.36 – -142.34 (m).

HRMS (ESI) (m/z): calcd for $C_{19}H_{14}F_6NaO_4$ ($[M + Na]^+$): 443.0688; found: 443.0695.

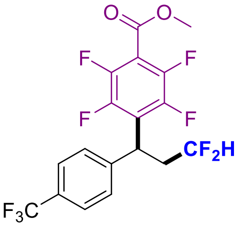

methyl (S)-4-(3,3-difluoro-1-(4-(trifluoromethyl)phenyl)propyl)-2,3,5,6-tetrafluorobenzoate (72)

This compound was obtained in 68% (58.5 mg) yield as yellow oil by the general procedure.

^{1}H NMR (600 MHz, $CDCl_3$) δ 7.62 (d, J = 8.1 Hz, 2H), 7.47 (d, J = 8.0 Hz, 2H), 5.81 (tt, J = 55.9, 4.8 Hz, 1H), 4.79 (t, J = 7.9 Hz, 1H), 3.97 (s, 3H), 2.93 – 2.72 (m, 2H).

^{13}C NMR (151 MHz, $CDCl_3$) δ 159.82, 146.52 – 144.83 (m), 144.61 – 143.64 (m), 142.91, 130.26 (q, J = 32.8 Hz), 127.93, 126.15 (d, J = 4.3 Hz), 123.80 (q, J = 271.8 Hz), 123.76 (t, J = 15.3 Hz), 115.36 (t, J = 240.6 Hz), 111.85 (t, J = 15.9 Hz), 53.34, 36.32 (t, J = 21.7 Hz), 35.24 – 34.98 (m).

^{19}F NMR (565 MHz, $CDCl_3$) δ -62.78, -115.79 – -118.22 (m), -138.16 – -138.54 (m), -141.05 (dd, J = 20.8, 12.3 Hz).

HRMS (ESI) (m/z): calcd for $C_{18}H_{11}F_9NaO_2$ ($[M + Na]^+$): 453.0508; found: 453.0505.

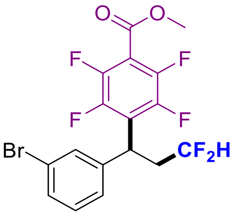

methyl (S)-4-(1-(3-bromophenyl)-3,3-difluoropropyl)-2,3,5,6-tetrafluorobenzoate (73)

This compound was obtained in 74% (65.0 mg) yield as yellow oil by the general procedure.

^{1}H NMR (600 MHz, $CDCl_3$) δ 7.47 (s, 1H), 7.42 (dt, J = 8.0, 1.4 Hz, 1H), 7.28 (s, 1H), 7.22 (t, J = 7.7 Hz, 1H), 5.79 (tt, J = 55.9, 4.4 Hz, 1H), 4.68 (t, J = 8.0 Hz, 1H), 3.97 (s, 3H), 2.87 – 2.70 (m, 2H).

^{13}C NMR (151 MHz, $CDCl_3$) δ 159.90, 146.85 – 144.70 (m), 144.70 – 143.25 (m), 141.15, 131.13, 130.70, 130.60, 126.11, 123.96 (t, J = 15.3 Hz), 123.16, 115.40 (t, J = 240.6 Hz), 111.70 (t, J = 15.8 Hz), 53.35, 36.39 (t, J = 21.5 Hz), 34.97 (t, J = 5.1 Hz).

^{19}F NMR (565 MHz, $CDCl_3$) δ -115.37 – -119.12 (m), -138.30 – -138.60 (m), -140.68 – -141.14 (m).

HRMS (ESI) (m/z): calcd for $C_{17}H_{11}BrF_6NaO_2$ ($[M + Na]^+$): 462.9738; found: 462.9735.

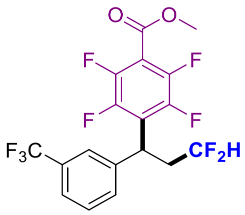

methyl (S)-4-(3,3-difluoro-1-(3-(trifluoromethyl)phenyl)propyl)-2,3,5,6-tetrafluorobenzoate (74)

This compound was obtained in 77% (58.5 mg) yield as yellow oil by the general procedure.

^{1}H NMR (600 MHz, $CDCl_3$) δ 7.56 (dd, J = 16.6, 8.5 Hz, 3H), 7.51 – 7.44 (m, 1H), 5.81 (tt, J = 55.8, 4.2 Hz, 1H), 4.79 (t, J = 8.0 Hz, 1H), 3.97 (s, 3H), 2.96 – 2.70 (m, 2H).

^{13}C NMR (151 MHz, $CDCl_3$) δ 159.84, 146.56 – 144.96 (m), 144.96 – 143.23 (m), 139.99, 131.61 (q, J = 32.5 Hz), 130.92, 129.74 (q, J = 273.0 Hz), 124.86 (d, J = 3.6 Hz), 123.79 (t, J = 15.3 Hz), 115.34 (t, J = 240.6 Hz), 111.86 (t, J = 15.9 Hz), 53.34, 36.39 (t, J = 21.6 Hz), 35.02 (t, J = 5.9 Hz).

^{19}F NMR (565 MHz, $CDCl_3$) δ -62.71, -115.46 – -118.55 (m), -137.40 – -139.17 (m), -141.08 (dd, J = 21.6, 12.5 Hz).

HRMS (ESI) (m/z): calcd for $C_{18}H_{11}F_9NaO_2$ ($[M + Na]^+$): 453.0508; found: 453.0506.

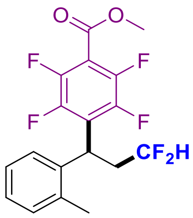

methyl (S)-4-(3,3-difluoro-1-(o-tolyl)propyl)-2,3,5,6-tetrafluorobenzoate (75)

This compound was obtained in 50% (37.6 mg) yield as yellow oil by the general procedure.

^{1}H NMR (600 MHz, $CDCl_3$) δ 7.44 – 7.37 (m, 1H), 7.25 – 7.15 (m, 3H), 5.76 (tt, J =

56.1, 4.5 Hz, 1H), 4.84 (t, J = 7.9 Hz, 1H), 3.96 (s, 3H), 2.87 – 2.63 (m, 2H), 2.36 (s, 3H).
^{13}C NMR (151 MHz, $CDCl_3$) δ 160.03, 146.78 – 144.97 (m), 144.75 – 143.46 (m), 136.19, 131.12, 127.80, 126.73, 124.24 (t, J = 15.8 Hz), 115.42 (t, J = 242.6 Hz), 111.31 (t, J = 15.7 Hz), 53.30, 37.50 (t, J = 22.4 Hz), 31.90 (t, J = 6.0 Hz), 20.66.
^{19}F NMR (565 MHz, $CDCl_3$) δ -115.65 – -117.84 (m), -138.75 – -139.03 (m), -139.84 – -140.27 (m).
HRMS (ESI) (m/z): calcd for $C_{18}H_{14}F_6NaO_2$ ([M + Na]$^+$): 399.0790; found: 399.0776.

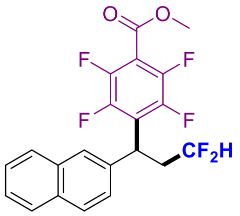

methyl (S)-4-(3,3-difluoro-1-(naphthalen-2-yl)propyl)-2,3,5,6-tetrafluorobenzoate (76)
This compound was obtained in 73% (60.2 mg) yield as yellow oil by the general procedure.
^{1}H NMR (500 MHz, $CDCl_3$) δ 7.85 – 7.77 (m, 4H), 7.49 (tt, J = 6.9, 5.3 Hz, 2H), 7.42 (d, J = 8.5 Hz, 1H), 5.82 (tt, J = 56.1, 4.6 Hz, 1H), 4.87 (t, J = 8.0 Hz, 1H), 3.95 (s, 3H), 3.02 – 2.80 (m, 2H).
^{13}C NMR (151 MHz, $CDCl_3$) δ 160.01, 146.22 – 145.12 (m), 144.44 – 143.80 (m), 136.19, 133.36, 132.68, 129.13, 127.89, 127.65, 126.69, 126.47, 126.25, 125.25, 124.67 (t, J = 15.5 Hz), 115.72 (t, J = 240.3 Hz), 111.31 (d, J = 15.7 Hz), 53.30, 36.58 (t, J = 21.4 Hz), 35.61.
^{19}F NMR (565 MHz, $CDCl_3$) δ -115.74 – -118.20 (m), -138.66 – -138.81 (m), -140.84 – -141.08 (m).
HRMS (ESI) (m/z): calcd for $C_{21}H_{14}F_6NaO_2$ ([M + Na]$^+$): 435.0790; found: 435.0786.

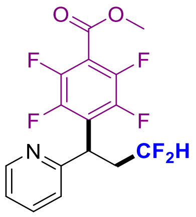

methyl (R)-4-(3,3-difluoro-1-(pyridin-2-yl)propyl)-2,3,5,6-tetrafluorobenzoate (77)
This compound was obtained in 89% (64.6 mg) yield as yellow oil by the general procedure.
^{1}H NMR (500 MHz, $CDCl_3$) δ 8.55 (d, J = 5.0 Hz, 1H), 7.63 (td, J = 8.0, 2.0 Hz, 1H), 7.19 (dd, J = 7.5, 5.0 Hz, 1H), 7.14 (d, J = 8.0 Hz, 1H), 5.95 (tt, J = 56.5, 4.5 Hz, 1H), 4.86 (t, J = 7.5 Hz, 1H), 3.96 (s, 3H), 3.21 – 3.06 (m, 1H), 2.75 – 2.59 (m, 1H).
^{13}C NMR (151 MHz, $CDCl_3$) δ 160.07, 157.76, 149.51, 144.91 (dm, J = 243.1 Hz), 136.95, 124.00 (t, J = 15.8 Hz), 122.53, 122.09, 116.00 (t, J = 240.0 Hz), 111.59, 53.30, 37.03 (t, J = 6.0 Hz), 35.67 (t, J = 21.1 Hz).
^{19}F NMR (565 MHz, $CDCl_3$) δ -115.28 – -118.11 (m), -138.96 – -139.21 (m), -140.39

– -140.68 (m).
HRMS (ESI) (m/z): calcd for $C_{16}H_{11}F_6NNaO_2$ ([M + Na]$^+$): 386.0586; found: 386.0590.

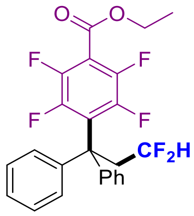

methyl 4-(3,3-difluoro-1,1-diphenylpropyl)-2,3,5,6-tetrafluorobenzoate (78)
This compound was obtained in 81% (73.2 mg) yield as yellow oil by the general procedure.
^{1}H NMR (600 MHz, $CDCl_3$) δ 7.39 – 7.33 (m, 8H), 7.29 (td, J = 5.9, 2.5 Hz, 2H), 5.49 – 5.15 (m, 1H), 4.43 (q, J = 7.1 Hz, 2H), 3.33 (td, J = 15.3, 4.6 Hz, 2H), 1.39 (t, J = 7.1 Hz, 3H).
^{13}C NMR (151 MHz, $CDCl_3$) δ 159.48, 146.11 (dd, J = 17.2, 8.5 Hz), 144.41 (dt, J = 22.7, 6.8 Hz), 142.31, 128.58, 127.46, 127.37, 116.00 (t, J = 240.1 Hz), 111.82 (d, J = 16.3 Hz), 62.77, 53.17, 45.12 – 44.15 (m), 14.08.
^{19}F NMR (565 MHz, $CDCl_3$) δ -114.16 (dt, J = 55.7, 15.5 Hz), -129.88 – -130.06 (m), -139.06 – -139.18 (m).
HRMS (ESI) (m/z): calcd for $C_{24}H_{18}F_6NaO_2$ ([M + Na]$^+$): 475.1103; found: 475.1101.

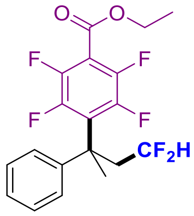

methyl 4-(4,4-difluoro-2-phenylbutan-2-yl)-2,3,5,6-tetrafluorobenzoate (79)
This compound was obtained in 74% (57.7 mg) yield as yellow oil by the general procedure.
^{1}H NMR (600 MHz, $CDCl_3$) δ 7.36 – 7.25 (m, 3H), 7.18 (dd, J = 7.4, 1.7 Hz, 2H), 5.91 – 5.46 (m, 1H), 4.44 (q, J = 7.1 Hz, 2H), 3.05 – 2.93 (m, 1H), 2.82 – 2.64 (m, 1H), 1.89 (t, J = 2.7 Hz, 3H), 1.40 (t, J = 7.1 Hz, 3H).
^{13}C NMR (151 MHz, $CDCl_3$) δ 159.53, 146.46, 145.96 (dd, J = 8.0, 4.5 Hz), 145.33 – 144.65 (m), 144.48 – 143.97 (m), 129.03 – 128.62 (m), 128.64, 127.10, 125.03, 118.47 – 114.14 (m), 111.82 (t, J = 16.4 Hz), 62.76, 45.30 – 44.00 (m), 28.15 (t, J = 5.2 Hz), 14.08.
^{19}F NMR (565 MHz, $CDCl_3$) δ -111.17 – -111.59 (m), -135.97 – -136.14 (m), -139.42 – -139.65 (m).
HRMS (ESI) (m/z): calcd for $C_{19}H_{16}F_6NaO_2$ ([M + Na]$^+$): 413.0947; found: 413.0952.

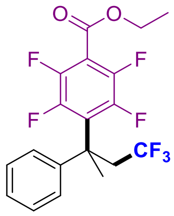

ethyl 2,3,5,6-tetrafluoro-4-(4,4,4-trifluoro-2-phenylbutan-2-yl)benzoate (80)

This compound was obtained in 78% (63.6 mg) yield as yellow oil by the general procedure.

^{1}H NMR (600 MHz, $CDCl_3$) δ 7.32 (dd, J = 8.4, 7.0 Hz, 2H), 7.28 – 7.23 (m, 1H), 7.16 – 7.11 (m, 2H), 4.44 (q, J = 7.1 Hz, 2H), 3.38 (dq, J = 15.1, 10.9 Hz, 1H), 2.95 (dq, J = 15.2, 10.5 Hz, 1H), 1.97 (d, J = 3.7 Hz, 3H), 1.40 (t, J = 7.1 Hz, 3H).

^{13}C NMR (151 MHz, $CDCl_3$) δ 159.52, 147.18, 146.99 – 145.48 (m), 145.20 – 143.73 (m), 128.85, 128.09 (q, J = 272.3 Hz), 127.10, 126.76 (t, J = 12.0 Hz), 124.55, 111.97 (t, J = 16.2 Hz), 62.73, 44.49, 43.61 – 42.40 (m), 29.60, 14.07.

^{19}F NMR (565 MHz, $CDCl_3$) δ -59.49 (t, J = 10.6 Hz), -136.41 – -136.66 (m), -139.59 – -140.14 (m).

HRMS (ESI) (m/z): calcd for $C_{19}H_{15}F_7NaO_2$ ($[M + Na]^+$): 431.0852; found: 431.0849.

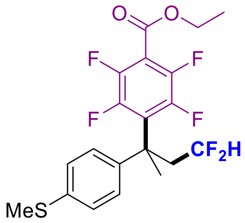

ethyl 4-(4,4-difluoro-2-(4-(methylthio)phenyl)butan-2-yl)-2,3,5,6-tetrafluoro-benzoate (81)

This compound was obtained in 67% (58.4 mg) yield as yellow oil by the general procedure.

^{1}H NMR (600 MHz, $CDCl_3$) δ 7.20 (d, J = 8.5 Hz, 2H), 7.09 (d, J = 8.6 Hz, 2H), 5.79 – 5.45 (m, 1H), 4.44 (q, J = 7.1 Hz, 2H), 2.97 (qd, J = 14.9, 5.4 Hz, 1H), 2.68 (ddt, J = 18.9, 15.4, 3.7 Hz, 1H), 2.47 (s, 3H), 1.87 (d, J = 2.7 Hz, 3H), 1.40 (t, J = 7.1 Hz, 3H).

^{13}C NMR (151 MHz, $CDCl_3$) δ 159.47, 146.87 – 145.61 (m), 144.94 – 143.59 (m), 143.21, 137.48, 127.93 (t, J = 11.9 Hz), 126.75, 125.57, 116.02 (t, J = 239.7 Hz), 111.87 (t, J = 16.3 Hz), 62.76, 44.67 – 44.20 (m), 28.12 – 27.99 (m), 15.64, 14.08.

^{19}F NMR (565 MHz, $CDCl_3$) δ -111.35 – -111.62 (m), -135.86 – -136.13 (m), -139.29 – -139.63 (m).

HRMS (ESI) (m/z): calcd for $C_{20}H_{18}F_6NaO_2S$ ($[M + Na]^+$): 459.0823; found: 459.0829.

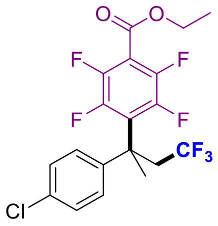

ethyl 4-(2-(4-chlorophenyl)-4,4,4-trifluorobutan-2-yl)-2,3,5,6-tetrafluorobenzoate (82)

This compound was obtained in 71% (62.7 mg) yield as yellow oil by the general procedure.

^{1}H NMR (600 MHz, $CDCl_3$) δ 7.30 (d, J = 8.7 Hz, 2H), 7.08 (d, J = 8.7 Hz, 2H), 4.45 (q, J = 7.1 Hz, 2H), 3.33 (dq, J = 15.1, 10.8 Hz, 1H), 2.93 (dq, J = 15.1, 10.3 Hz, 1H), 1.94 (t, J = 3.5 Hz, 3H), 1.40 (t, J = 7.1 Hz, 3H).

^{13}C NMR (151 MHz, $CDCl_3$) δ 159.38, 146.80 – 146.43 (m), 145.87 (dd, J = 17.1, 4.7 Hz), 145.60, 145.19 – 144.75 (m), 144.43 – 143.97 (m), 133.04, 129.00, 126.08, 125.60 (q, J = 281.4 Hz), 112.25 (t, J = 16.1 Hz), 62.80, 44.16, 43.20 (q, J = 26.3 Hz), 29.41, 14.06.
^{19}F NMR (565 MHz, $CDCl_3$) δ -59.50 (t, J = 10.5 Hz), -136.43 (dd, J = 21.1, 11.6 Hz), -139.30 – -139.57 (m).
HRMS (ESI) (m/z): calcd for $C_{19}H_{15}ClF_7NaO_2$ ($[M + H]^+$): 443.0643; found: 443.0645.

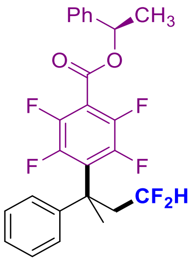

(R)-1-phenylethyl 4-(4,4-difluoro-2-phenylbutan-2-yl)-2,3,5,6-tetrafluorobenzoate (83)
This compound was obtained in 45% (41.9mg) yield as yellow oil by the general procedure.
^{1}H NMR (600 MHz, $CDCl_3$) δ 7.45 – 7.41 (m, 3.2H), 7.38 (dd, J = 7.2, 2.0 Hz, 3.2H), 7.35 – 7.31 (m, 2.4H), 7.27 (d, J = 7.9 Hz, 1.2H), 7.26 – 7.23 (m, 1.7H), 7.21 – 7.14 (m, 2.0H), 7.05 (dt, J = 7.1, 2.0 Hz, 1.2H), 6.16 (q, J = 6.6 Hz, 1.6H), 5.63 (tdt, J = 55.9, 12.4, 4.6 Hz, 1.7H), 2.96 (q, J = 14.9 Hz, 1.7H), 2.80 – 2.58 (m, 1.7H), 1.88 (dt, J = 5.5, 2.6 Hz, 5H), 1.68 (dd, J = 6.6, 3.6 Hz, 5H).
^{13}C NMR (151 MHz, $CDCl_3$) δ 158.65, 148.60, 146.56 – 145.96 (m), 145.09 – 144.26 (m), 140.39, 140.33, 134.85, 130.15, 128.85, 128.67, 128.38, 128.35, 127.43, 126.24, 125.36, 125.03, 123.35, 115.94 (td, J = 240.3, 49.2 Hz), 112.11, 75.44, 75.33, 45.04 – 43.78 (m), 28.21 (t, J = 5.8 Hz), 22.18, 22.17.
^{19}F NMR (565 MHz, $CDCl_3$) δ -111.48 (ddt, J = 56.2, 32.0, 16.5 Hz), -135.78 – -136.37 (m), -138.49 – -138.87 (m), -139.03 – -139.34 (m).
HRMS (ESI) (m/z): calcd for $C_{25}H_{21}F_6O_2$ ($[M + H]^+$): 467.1440; found: 467.1439.

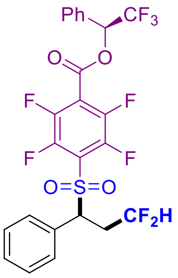

(S)-2,2,2-trifluoro-1-phenylethyl 4-(((S)-3,3-difluoro-1-phenylpropyl)sulfonyl)-2,3,5,6-tetrafluoro-benzoate (84)
This compound was obtained in 80% (91.3 mg) yield as white solid by the general procedure.
^{1}H NMR (600 MHz, $CDCl_3$) δ 7.52 – 7.50 (m, 2H), 7.49 – 7.44 (m, 3H), 7.41 – 7.38 (m, 1H), 7.36 (t, J = 7.5 Hz, 2H), 7.32 (d, J = 8.5 Hz, 2H) 6.32 (q, J = 6.5 Hz, 1H), 5.79 (tdd, J = 55.5, 5.5, 3.5 Hz, 1H), 4.61 (dd, J = 10.0, 4.0 Hz, 1H), 3.04 – 2.92 (m, 1H),

2.86 – 2.73 (m, 1H).

^{13}C NMR (151 MHz, $CDCl_3$) δ 156.25, 144.35 (dm, J = 251.5 Hz), 130.59, 129.52, 129.36, 129.22, 128.98, 128.71, 128.18, 122.51 (q, J = 280.5 Hz), 120.38 (t, J = 14.5 Hz), 116.11 (d, J = 15.0 Hz), 114.37 (t, J = 241.5 Hz), 74.35 (q, J = 34.1 Hz), 67.32 (t, J = 6.0 Hz), 32.49 (t, J = 24.0 Hz), 29.70.

^{19}F NMR (565 MHz, $CDCl_3$) δ -75.90 (d, J = 6.3 Hz), -115.93 – -117.38 (m), -133.21 – -133.44 (m), -134.58 – -134.80 (m).

HRMS (ESI) (m/z): calcd for $C_{24}H_{15}F_9NaO_4S$ ($[M + Na]^+$): 593.4148; found: 593.4144.

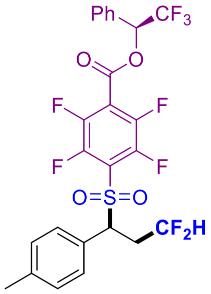

(S)-2,2,2-trifluoro-1-phenylethyl 4-(((S)-3,3-difluoro-1-(p-tolyl)propyl)sulfonyl)-2,3,5,6-tetra-fluorobenzoate (85)

This compound was obtained in 81% (94.7 mg) yield as white solid by the general procedure.

^{1}H NMR (600 MHz, $CDCl_3$) δ 7.51 (d, J = 7.4 Hz, 2H), 7.49 – 7.43 (m, 3H), 7.20 (d, J = 8.1 Hz, 2H), 7.15 (d, J = 7.9 Hz, 2H), 6.33 (q, J = 6.5 Hz, 1H), 5.76 (tdd, J = 55.8, 5.9, 3.4 Hz, 1H), 4.58 (dd, J = 10.6, 4.5 Hz, 1H), 3.00 – 2.88 (m, 1H), 2.83 – 2.69 (m, 1H), 2.33 (s, 3H).

^{13}C NMR (151 MHz, $CDCl_3$) δ 156.31, 144.64 (dm, J = 261.5 Hz), 140.86, 130.59, 130.22, 129.54, 129.08, 128.98, 128.18, 126.12, 122.53 (q, J = 280.7 Hz), 120.57 (t, J = 14.6 Hz), 115.90 (d, J = 15.5 Hz), 114.46 (t, J = 241.4 Hz), 74.34 (q, J = 34.1 Hz), 67.10 (t, J = 5.7 Hz), 32.49 (t, J = 24.1 Hz), 21.18.

^{19}F NMR (565 MHz, $CDCl_3$) δ -75.91 (d, J = 6.5 Hz), -116.55 – -116.85 (m), -133.26 – -133.44 (m), -134.67 – -134.88 (m).

HRMS (ESI) (m/z): calcd for $C_{25}H_{17}F_9NaO_4S$ ($[M + Na]^+$): 607.4418; found: 607.4423.

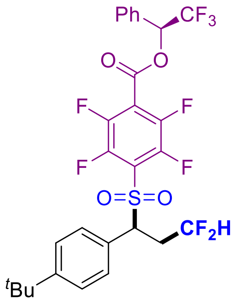

(S)-2,2,2-trifluoro-1-phenylethyl 4-(((S)-1-(4-(tert-butyl)phenyl)-3,3-difluoropropyl)sulfonyl)-2,3,5,6-tetrafluorobenzoate (86)

This compound was obtained in 84% (105.2 mg) yield as white solid by the general procedure.

^{1}H NMR (600 MHz, $CDCl_3$) δ 7.53 – 7.43 (m, 5H), 7.34 (d, J = 8.4 Hz, 2H), 7.22 (d,

J = 6.0 Hz, 2H), 6.33 (q, J = 6.5 Hz, 1H), 5.81 (tdd, J = 55.7, 5.9, 3.5 Hz, 1H), 4.58 (dd, J = 10.1, 3.9 Hz, 1H), 3.01 – 2.89 (m, 1H), 2.86 – 2.73 (m, 1H), 1.25 (s, 9H).
^{13}C NMR (151 MHz, $CDCl_3$) δ 156.24, 154.12, 144.58 (dm, J = 265.4 Hz), 130.58, 129.53, 128.97, 128.16, 126.35, 126.09, 123.98 (q, J = 281.6 Hz), 120.41 (t, J = 14.9 Hz), 115.92, 114.46 (t, J = 241.5 Hz), 74.34 (q, J = 34.2 Hz), 67.30 (t, J = 5.6 Hz), 34.76, 32.28 (t, J = 24.2 Hz), 30.97.
^{19}F NMR (565 MHz, $CDCl_3$) δ -75.92 (d, J = 6.3 Hz), -116.49 – -116.75 (m), -133.05 – -133.27 (m), -135.15 – -135.51 (m).
HRMS (ESI) (m/z): calcd for $C_{28}H_{13}F_9NaO_4S$ ([M + Na]$^+$): 649.5227; found: 649.5236.

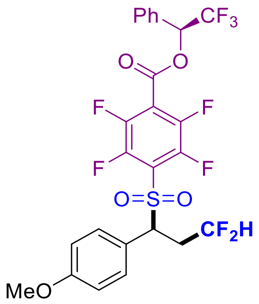

(S)-2,2,2-trifluoro-1-phenylethyl 4-(((S)-3,3-difluoro-1-(4-methoxyphenyl)-propyl)sulfonyl)-2,3,5,6-tetrafluorobenzoate (87)
This compound was obtained in 73% (87.7 mg) yield as white solid by the general procedure.
^{1}H NMR (600 MHz, $CDCl_3$) δ 7.51 (d, J = 12.0 Hz, 2H), 7.49 – 7.43 (m, 3H), 7.24 (d, J = 6.0 Hz, 2H), 6.86 (d, J = 6.0 Hz, 2H), 6.33 (q, J = 6.5 Hz, 1H), 5.76 (tdd, J = 55.9, 6.1, 3.5 Hz, 1H), 4.57 (dd, J = 10.6, 4.4 Hz, 1H), 3.79 (s, 3H), 2.98 – 2.86 (m, 1H), 2.81 – 2.68 (m, 1H).
^{13}C NMR (151 MHz, $CDCl_3$) δ 161.13, 156.30, 144.59 (dm, J = 267.9 Hz), 130.59, 130.56, 129.53, 128.98, 128.19, 122.06 (q, J = 280.7 Hz), 120.70 (t, J = 14.1 Hz), 114.94, 114.31 (t, J = 241.5 Hz), 74.34 (q, J = 33.8 Hz), 66.76 (t, J = 5.4 Hz), 55.36, 32.52 (t, J = 23.9 Hz).
^{19}F NMR (565 MHz, $CDCl_3$) δ -75.88 (d, J = 6.2 Hz), -116.60 – -116.88 (m), -133.33 – -133.47 (m), -134.62 – -134.78 (m).
HRMS (ESI) (m/z): calcd for $C_{25}H_{17}F_9NaO_5S$ ([M + Na]$^+$): 623.0545; found: 623.0550.

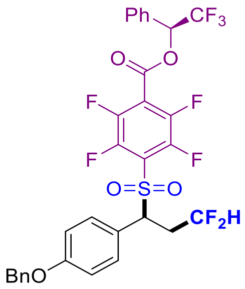

(S)-2,2,2-trifluoro-1-phenylethyl 4-(((S)-1-(4-(benzyloxy)phenyl)-3,3-difluoropropyl)sulfonyl)-2,3,5,6-tetrafluorobenzoate (88)
This compound was obtained in 58% (78.5 mg) yield as white solid by the general procedure.
^{1}H NMR (600 MHz, $CDCl_3$) δ 7.52 (d, J = 6.0 Hz, 2H), 7.48 – 7.42 (m, 3H), 7.40 –

7.35 (m, 4H), 7.34 – 7.29 (m, 1H), 7.24 (d, J = 8.5 Hz, 2H), 6.93 (d, J = 6.0 Hz, 2H), 6.34 (q, J = 6.5 Hz, 1H), 5.77 (tdd, J = 55.8, 6.0, 3.4 Hz, 1H), 5.04 (s, 2H), 4.56 (dd, J = 10.4, 4.6 Hz, 1H), 3.00 – 2.85 (m, 1H), 2.80 – 2.67 (m, 1H).

^{13}C NMR (151 MHz, $CDCl_3$) δ 160.29, 156.31, 144.66 (dm, J = 255.2 Hz), 136.09, 130.61, 129.53, 128.99, 128.66, 128.23, 128.20, 127.44 (d, J = 2.9 Hz), 122.47 (q, J = 279.6 Hz), 120.96, 120.65 (t, J = 14.5 Hz), 114.79 (t, J = 244.5 Hz), 74.35 (q, J = 34.1 Hz), 70.14, 66.77 (t, J = 5.9 Hz), 32.52 (t, J = 24.0 Hz).

^{19}F NMR (565 MHz, $CDCl_3$) δ -75.84 (d, J = 6.0 Hz), -116.58 – -116.85 (m), -133.27 – -133.48 (m), -134.55 – -134.72 (m).

HRMS (ESI) (m/z): calcd for $C_{31}H_{21}F_9NaO_5S$ ($[M + Na]^+$): 699.0858; found: 699.0852.

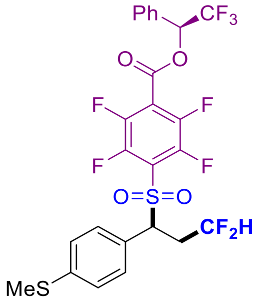

(S)-2,2,2-trifluoro-1-phenylethyl 4-(((S)-3,3-difluoro-1-(4-(methylthio)phenyl)propyl)sulfonyl)-2,3,5,6-tetrafluorobenzoate (89)

This compound was obtained in 62% (76.4 mg) yield as white solid by the general procedure.

^{1}H NMR (600 MHz, $CDCl_3$) δ 7.98 (d, J = 6.5 Hz, 1H), 7.87 (t, J = 1.9 Hz, 1H), 7.59 (d, J = 8.5 Hz, 1H), 7.53 – 7.44 (m, 6H), 6.33 (q, J = 6.5 Hz, 1H), 5.84 (tdd, J = 55.6, 5.2, 3.6 Hz, 1H), 4.69 (dd, J = 10.1, 4.8 Hz, 1H), 3.05 – 2.93 (m, 1H), 2.90 – 2.77 (m, 1H), 2.58 (s, 3H).

^{13}C NMR (151 MHz, $CDCl_3$) δ 156.11, 14.71 (dm, J = 265.8 Hz), 138.03, 133.61, 130.60, 130.31, 130.25, 129.88, 129.47, 128.99, 128.17, 122.50 (q, J = 280.7 Hz), 114.13 (t, J = 242.0 Hz), 74.43 (q, J = 34.1 Hz), 66.91 (t, J = 5.0 Hz), 32.59 (t, J = 23.7 Hz), 26.51.

^{19}F NMR (565 MHz, $CDCl_3$) δ -75.90 (d, J = 6.5 Hz), -115.14 – -117.74 (m), -132.55 – -133.60 (m), -133.94 – -134.60 (m).

HRMS (ESI) (m/z): calcd for $C_{25}H_{18}F_9O_4S_2$ ($[M + H]^+$): 617.0497; found: 617.0505.

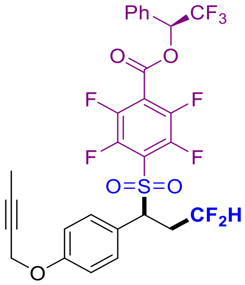

(S)-2,2,2-trifluoro-1-phenylethyl 4-(((S)-1-(4-(but-2-yn-1-yloxy)phenyl)-3,3-difluoropropyl)-sulfonyl)-2,3,5,6-tetrafluorobenzoate (90)

This compound was obtained in 82% (104.7 mg) yield as white solid by the general procedure.

^{1}H NMR (600 MHz, $CDCl_3$) δ 7.51 (d, J = 7.4 Hz, 2H), 7.50 – 7.42 (m, 3H), 7.26 (d, J = 6.0 Hz, 2H), 6.93 (d, J = 6.0 Hz, 2H), 6.33 (q, J = 6.5 Hz, 1H), 5.78 (tdd, J = 55.8, 5.9, 3.4 Hz, 1H), 4.67 – 4.61 (m, 2H), 4.57 (dd, J = 10.4, 4.6 Hz, 1H), 3.01 – 2.84 (m, 1H), 2.82 – 2.68 (m, 1H), 1.83 (d, J = 5.5 Hz, 3H).

^{13}C NMR (151 MHz, $CDCl_3$) δ 159.34, 156.27, 144.58 (dm, J = 264.2 Hz), 130.58, 130.52, 129.53, 128.98, 128.20, 122.52 (q, J = 280.6 Hz), 121.22, 120.61 (t, J = 14.6 Hz), 115.86, 115.77 113.75 (t, J = 241.3 Hz), 84.49, 74.34 (q, J = 34.3 Hz), 73.17, 66.75 (t, J = 5.7 Hz), 56.53, 32.53 (t, J = 24.0 Hz), 3.53.

^{19}F NMR (565 MHz, $CDCl_3$) δ -75.86 (d, J = 6.2 Hz), -116.06 – -117.44 (m), -133.20 – -133.54 (m), -134.51 – -134.86 (m).

HRMS (ESI) (m/z): calcd for $C_{28}H_{19}F_9NaO_5S$ ($[M + Na]^+$): 661.0702; found: 661.0698.

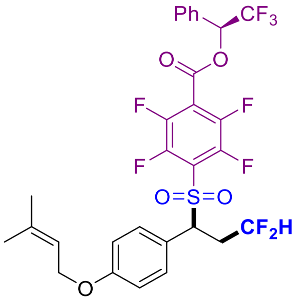

(S)-2,2,2-trifluoro-1-phenylethyl 4-(((S)-3,3-difluoro-1-(4-((3-methylbut-2-en-1-yl)oxy)phenyl)-propyl)sulfonyl)-2,3,5,6-tetrafluorobenzoate (91)

This compound was obtained in 87% (113.9 mg) yield as white solid by the general procedure.

^{1}H NMR (600 MHz, $CDCl_3$) δ 7.51 (d, J = 7.4 Hz, 2H), 7.49 – 7.43 (m, 3H), 7.22 (d, J = 8.5 Hz, 2H), 6.86 (d, J = 8.0 Hz, 2H), 6.33 (q, J = 6.5 Hz, 1H), 5.77 (tdd, J = 55.8, 6.0, 3.4 Hz, 1H), 5.46 – 5.41 (m, 1H), 4.56 (dd, J = 10.2, 4.4 Hz, 1H), 4.48 (d, J = 6.0 Hz, 2H), 3.01 – 2.86 (m, 1H), 2.81 – 2.68 (m, 1H), 1.78 (d, J = 1.4 Hz, 3H), 1.72 (d, J = 1.6 Hz, 3H).

^{13}C NMR (151 MHz, $CDCl_3$) δ 160.45, 156.31, 144.58 (dm, J = 254.2 Hz), 138.89, 130.58, 130.52, 129.53, 128.98, 128.19, 122.46 (q, J = 284.1 Hz), 120.47, 118.90, 115.63, 115.44, (t, J = 240.1 Hz),114.43 74.34 (q, J = 34.1 Hz), 66.80 (t, J = 5.3 Hz), 64.97, 32.51 (t, J = 24.0 Hz), 25.76, 18.19.

^{19}F NMR (565 MHz, $CDCl_3$) δ -75.87 (d, J = 6.3 Hz), -116.64 – -116.89 (m), -133.27 – -133.45 (m), -134.64 – -134.77 (m).

HRMS (ESI) (m/z): calcd for $C_{29}H_{24}F_9O_5S$ ($[M + H]^+$): 655.1195; found: 655.1204.

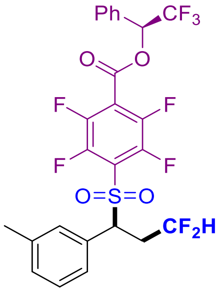

(S)-2,2,2-trifluoro-1-phenylethyl 4-(((S)-3,3-difluoro-1-(m-tolyl)propyl)sulfonyl)-

2,3,5,6-tetra-fluorobenzoate (92)

This compound was obtained in 55% (64.3 mg) yield as white solid by the general procedure.

^{1}H NMR (600 MHz, $CDCl_3$) δ 7.53 – 7.44 (m, 5H), 7.22 (t, J = 7.5 Hz, 1H), 7.18 (d, J = 8.0 Hz, 1H), 7.13 (s, 1H), 7.09 (d, J = 7.8 Hz, 1H), 6.33 (q, J = 6.5 Hz, 1H), 5.78 (tdd, J = 55.9, 5.9, 3.5 Hz, 1H), 4.58 – 4.54 (m, 1H), 3.01 – 2.87 (m, 1H), 2.85 – 2.71 (m, 1H), 2.31 (s, 3H).

^{13}C NMR (151 MHz, $CDCl_3$) δ 156.37, 144.64 (dm, J = 264.3 Hz), 138.07, 131.43, 130.49, 130.27, 129.43, 128.88, 128.20, 128.03, 127.15, 126.96, 122.41 (q, J = 281.6 Hz), 120.64 (t, J = 15.5 Hz), 116.22 (t, J = 15.4 Hz), 114.27 (t, J = 241.1 Hz), 74.39 (q, J = 34.1 Hz), 62.20 (t, J = 5.6 Hz), 33.71 (t, J = 23.6 Hz), 19.43.

^{19}F NMR (565 MHz, $CDCl_3$) δ -75.97 (d, J = 6.8 Hz), -116.61 – -116.94 (m), -132.79 – -133.11 (m), -134.38 – -134.69 (m).

HRMS (ESI) (m/z): calcd for $C_{25}H_{17}F_9NaO_4S$ ($[M + Na]^+$): 607.4418; found: 607.4415.

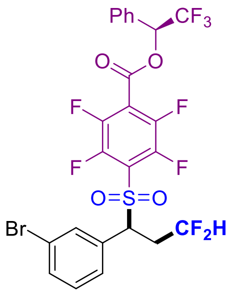

(S)-2,2,2-trifluoro-1-phenylethyl 4-(((S)-1-(3-bromophenyl)-3,3-difluoropropyl)-sulfonyl)-2,3,5,6-tetrafluorobenzoate (93)

This compound was obtained in 71% (92.2 mg) yield as white solid by the general procedure.

^{1}H NMR (600 MHz, $CDCl_3$) δ 7.54 (d, J = 6.0 Hz, 1H), 7.53 – 7.50 (m, 2H), 7.50 – 7.43 (m, 4H), 7.29 (d, J = 7.8 Hz, 1H), 7.26 (t, J = 3.9 Hz, 1H), 6.34 (q, J = 6.5 Hz, 1H), 5.84 (tdd, J = 55.6, 5.4, 3.6 Hz, 1H), 4.56 (dd, J = 10.2, 4.6 Hz, 1H), 3.01 – 2.86 (m, 1H), 2.82 – 2.69 (m, 1H).

^{13}C NMR (151 MHz, $CDCl_3$) δ 156.13, 144.75 (dm, J = 261.9 Hz), 133.74, 132.27, 131.58, 130.93, 130.61, 129.49, 128.99, 128.18, 127.94, 123.40, 122.50 (q, J = 280.8 Hz), 119.95 (t, J = 14.5 Hz), 116.49 (t, J = 15.5 Hz), 114.09 (t, J = 241.9 Hz), 74.42 (q, J = 34.0 Hz), 66.63 (t, J = 5.0 Hz), 32.54 (t, J = 23.8 Hz).

^{19}F NMR (565 MHz, $CDCl_3$) δ -75.89 (d, J = 6.3 Hz), -115.25 – -117.72 (m), -133.16 – -133.28 (m), -134.17 – -134.31 (m).

HRMS (ESI) (m/z): calcd for $C_{24}H_{14}BrF_9NaO_4S$ ($[M + Na]^+$): 670.9545; found: 670.9551.

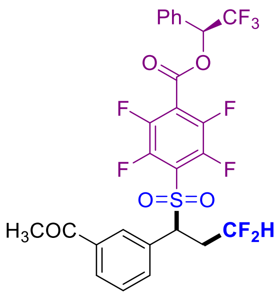

(S)-2,2,2-trifluoro-1-phenylethyl 4-(((S)-1-(3-acetylphenyl)-3,3-difluoropropyl)-sulfonyl)-2,3,5,6-tetrafluorobenzoate (94)

This compound was obtained in 49% (59.9 mg) yield as white solid by the general procedure.

^{1}H NMR (600 MHz, $CDCl_3$) δ 7.51 (d, J = 7.4 Hz, 2H), 7.51 – 7.42 (m, 3H), 7.23 (d, J = 8.2 Hz, 2H), 7.18 (dd, J = 8.5, 2.5 Hz, 2H), 6.33 (q, J = 6.5 Hz, 1H), 5.77 (tdd, J = 55.8, 5.9, 3.5 Hz, 1H), 4.57 (dd, J = 10.5, 4.5 Hz, 1H), 3.00 – 2.86 (m, 1H), 2.82 – 2.69 (m, 1H), 2.45 (s, 3H).

^{13}C NMR (151 MHz, $CDCl_3$) δ 156.26, 144.64 (dm, J = 263.4 Hz), 142.53, 130.59, 129.51,129.49, 128.99, 128.19, 126.52, 125.11, 122.46 (q, J = 279.6 Hz),116.12 (t, J = 15.5 Hz), 114.35 (t, J = 241.7 Hz), 74.38 (q, J = 33.9 Hz), 66.86 (t, J = 5.7 Hz), 32.54 (t, J = 24.0 Hz), 14.99.

^{19}F NMR (565 MHz, $CDCl_3$) δ -75.86 (d, J = 6.7 Hz), -115.96 – -117.37 (m), -133.26 – -133.43 (m), -134.28 – -134.54 (m).

HRMS (ESI) (m/z): calcd for $C_{26}H_{18}F_9O_5S$ ([M + H]$^+$): 613.0726; found: 613.0725.

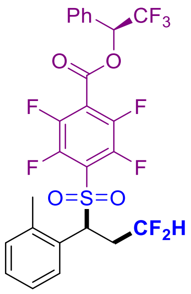

(S)-2,2,2-trifluoro-1-phenylethyl 4-(((S)-3,3-difluoro-1-(o-tolyl)propyl)sulfonyl)-2,3,5,6-tetra-fluorobenzoate (95)

This compound was obtained in 51% (59.6 mg) yield as white solid by the general procedure.

^{1}H NMR (600 MHz, $CDCl_3$) δ 7.59 (d, J = 6.5 Hz, 1H), 7.52 (d, J = 7.4 Hz, 2H), 7.50 – 7.42 (m, 3H), 7.32 – 7.26 (m, 2H), 7.18 (t, J = 6.4 Hz, 1H), 6.34 (q, J = 6.5 Hz, 1H), 5.67 (tdd, J = 56.0, 6.0, 4.5 Hz, 1H), 5.03 (t, J = 7.4 Hz, 1H), 2.86 – 2.75 (m, 2H), 2.30 (s, 3H).

^{13}C NMR (151 MHz, $CDCl_3$) δ 156.26, 144.86 (dm, J = 264.3 Hz), 138.28, 131.43, 130.59, 130.20, 129.53, 128.98, 128.20, 128.13, 127.15, 127.01, 122.53 (q, J = 280.8 Hz), 120.96 (t, J = 14.5 Hz), 116.22 (t, J = 15.4 Hz), 114.40 (t, J = 241.4 Hz), 74.39 (q, J = 34.1 Hz), 62.13 (t, J = 5.6 Hz), 33.86 (t, J = 23.6 Hz), 19.20.

^{19}F NMR (565 MHz, $CDCl_3$) δ -75.88 (d, J = 6.8 Hz), -116.63 – -116.84 (m), -132.79

– -133.01 (m), -134.38 – -134.57 (m).
HRMS (ESI) (m/z): calcd for $C_{25}H_{17}F_9NaO_4S$ ([M + Na]$^+$): 607.4418; found: 607.4422.

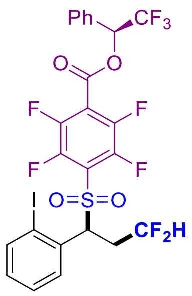

(S)-2,2,2-trifluoro-1-phenylethyl 4-(((S)-3,3-difluoro-1-(2-iodophenyl)propyl)-sulfonyl)-2,3,5,6-tetrafluorobenzoate (96)
This compound was obtained in 63% (87.7 mg) yield as white solid by the general procedure.
^{1}H NMR (600 MHz, $CDCl_3$) δ 7.74 (d, J = 6.0 Hz, 1H), 7.62 (t, J = 6.0 Hz, 1H), 7.53 – 7.51 (m, 2H), 7.50 – 7.44 (m, 3H), 7.34 (d, J = 10.4 Hz, 1H), 7.12 (t, J = 7.8 Hz, 1H), 6.34 (q, J = 6.5 Hz, 1H), 5.85 (tdd, J = 55.6, 5.4, 3.6 Hz, 1H), 4.53 (dd, J = 10.5, 4.5 Hz, 1H), 3.01 – 2.87 (m, 1H), 2.82 – 2.69 (m, 1H).
^{13}C NMR (151 MHz, $CDCl_3$) δ 156.14, 144.79 (dm, J = 264.2 Hz), 139.65, 138.23, 131.56, 130.99, 130.61, 129.50, 128.99, 128.51, 128.21, 122.52 (q, J = 280.7 Hz), 119.86 (d, J = 13.9 Hz), 116.51 (t, J = 15.4 Hz), 114.11 (t, J = 242.0 Hz), 94.64, 74.41 (q, J = 34.1 Hz), 66.56 (t, J = 5.4 Hz), 32.41 (t, J = 23.7 Hz).
^{19}F NMR (565 MHz, $CDCl_3$) δ -75.85 (d, J = 6.4 Hz), -115.33 – -117.70 (m), -133.10 – -133.25 (m), -134.20 – -134.33 (m).
HRMS (ESI) (m/z): calcd for $C_{24}H_{12}F_9INaO_4S$ ([M + Na]$^+$): 718.9406; found: 718.9420.

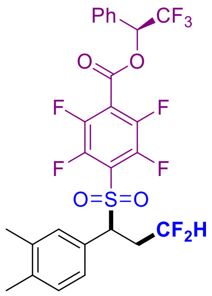

(S)-2,2,2-trifluoro-1-phenylethyl 4-(((S)-1-(3,4-dimethylphenyl)-3,3-difluoro-propyl)sulfonyl)-2,3,5,6-tetrafluorobenzoate (97)
This compound was obtained in 85% (101.7 mg) yield as white solid by the general procedure.
^{1}H NMR (600 MHz, $CDCl_3$) δ 7.54 – 7.41 (m, 5H), 7.11 – 7.06 (m, 2H), 7.02 (d, J = 7.9 Hz, 1H), 6.33 (q, J = 6.5 Hz, 1H), 5.75 (tdd, J = 55.8, 6.1, 3.3 Hz, 1H), 4.54 (dd, J = 10.0, 5.0 Hz, 1H), 2.96 – 2.85 (m, 1H), 2.84 – 2.70 (m, 1H), 2.22 (s, 3H), 2.21 (s, 3H).
^{13}C NMR (151 MHz, $CDCl_3$) δ 156.31, 144.74 (dm, J = 260.6 Hz), 139.50, 138.11, 130.64, 130.60, 130.10, 129.53, 128.98, 128.17, 126.61, 126.37, 122.53 (q, J = 280.7

Hz), 120.61 (t, J = 14.8 Hz), 115.94 (t, J = 14.8 Hz), 114.51 (t, J = 241.5 Hz), 74.34 (q, J = 34.0 Hz), 67.23 (t, J = 5.9 Hz), 32.51 (t, J = 24.0 Hz), 19.61, 19.52.
^{19}F NMR (565 MHz, $CDCl_3$) δ -75.92 (d, J = 6.3 Hz), -116.55 – -116.88 (m), -133.24 – -133.42 (m), -134.96 – -135.12 (m).
HRMS (ESI) (m/z): calcd for $C_{26}H_{20}F_9O_4S$ ($[M + H]^+$): 599.0933; found: 599.0945.

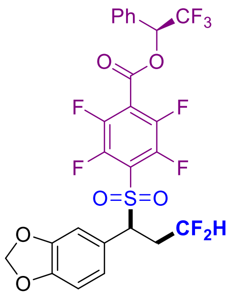

(S)-2,2,2-trifluoro-1-phenylethyl-4-(((S)-1-(benzo[d][1,3]dioxol-5-yl)-3,3-difluoro-propyl)sulfonyl)-2,3,5,6-tetrafluorobenzoate (98)
This compound was obtained in 66% (81.1 mg) yield as white solid by the general procedure.
^{1}H NMR (600 MHz, $CDCl_3$) δ 7.52 (d, J = 6.5 Hz, 2H), 7.50 – 7.43 (m, 3H), 6.88 (s, 1H), 6.73 (t, J = 1.4 Hz, 2H), 6.34 (q, J = 6.5 Hz, 1H), 6.01 (t, J = 1.7 Hz, 1H), 5.99 (d, J = 1.5 Hz, 1H), 5.78 (tdd, J = 55.8, 6.0, 3.4 Hz, 1H), 4.52 (dd, J = 10.4, 5.0 Hz, 1H), 2.96 – 2.84 (m, 1H), 2.77 – 2.62 (m, 1H).
^{13}C NMR (151 MHz, $CDCl_3$) δ 156.29, 149.48, 148.73, 144.74 (dm, J = 266.1 Hz), 130.60, 129.52, 128.98, 128.20, 123.72, 122.62 (q, J = 280.7 Hz), 122.41, 120.58 (t, J = 14.6 Hz), 116.08 (t, J = 15.5 Hz), 114.38 (t, J = 241.2 Hz), 108.97, 108.77, 101.88, 74.38 (q, J = 34.1 Hz), 67.01 (t, J = 5.7 Hz), 32.66 (t, J = 24.0 Hz).
^{19}F NMR (565 MHz, $CDCl_3$) δ -75.87 (d, J = 6.3 Hz), -116.09 – -117.40 (m), -133.30 – -133.57 (m), -134.32 – -134.66 (m).
HRMS (ESI) (m/z): calcd for $C_{25}H_{15}F_9NaO_6S$ ($[M + Na]^+$): 637.0338; found: 637.0343.

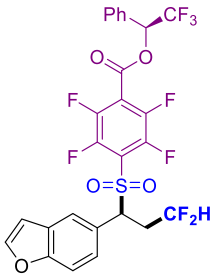

(S)-2,2,2-trifluoro-1-phenylethyl 4-(((S)-1-(benzofuran-5-yl)-3,3-difluoropropyl)-sulfonyl)-2,3,5,6-tetrafluorobenzoate (99)
This compound was obtained in 53% (64.7 mg) yield as white solid by the general procedure.
^{1}H NMR (600 MHz, $CDCl_3$) δ 7.66 (t, J = 2.5 Hz, 1H), 7.63 (d, J = 2.2 Hz, 1H), 7.51 – 7.41 (m, 6H), 7.24 (d, J = 8.6 Hz, 1H), 6.76 (d, J = 6.0 Hz, 1H), 6.31 (q, J = 6.5 Hz, 1H), 5.76 (tdd, J = 55.8, 6.0, 3.4 Hz, 1H), 4.73 (dd, J = 10.0, 5.5 Hz, 1H), 3.06 – 2.93 (m, 1H), 2.90 – 2.76 (m, 1H).
^{13}C NMR (151 MHz, $CDCl_3$) δ 156.22, 155.57, 146.67, 144.52 (dm, J = 258.7 Hz),

130.58, 129.51, 128.97, 128.56, 128.17, 125.08, 123.65, 122.68 (q, J = 277.7 Hz), 122.41, 120.54, 114.83 (t, J = 241.7 Hz), 112.57, 106.56, 74.33 (q, J = 34.1 Hz), 67.33 (t, J = 5.9 Hz), 33.02 (t, J = 24.0 Hz).
^{19}F NMR (565 MHz, $CDCl_3$) δ -75.90 (d, J = 6.6 Hz), -116.61 – -116.92 (m), -133.36 – -133.52 (m), -134.47 – -134.70 (m).
HRMS (ESI) (m/z): calcd for $C_{26}H_{15}F_9NaO_5S$ ($[M + Na]^+$): 633.0389; found: 633.0385.

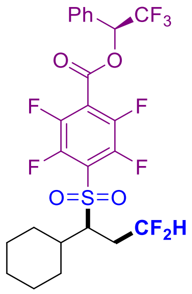

(S)-2,2,2-trifluoro-1-phenylethyl 4-(((S)-1-cyclohexyl-3,3-difluoropropyl)-sulfonyl)-2,3,5,6-tetra-fluorobenzoate (100)
This compound was obtained in 68% (78.4 mg) yield as yellow oil by the general procedure.
^{1}H NMR (600 MHz, $CDCl_3$) δ 7.54 (d, J = 6.0 Hz, 2H), 7.51 – 7.44 (m, 3H), 6.37 (q, J = 6.5 Hz, 1H), 6.11 (tdd, J = 56.5, 5.6, 3.2 Hz, 1H), 3.36 (dt, J = 7.1, 3.1 Hz, 1H), 2.64 -2.51 (m, 1H), 2.40 – 2.28 (m, 1H), 2.18 – 2.11 (m, 1H), 2.10 – 2.03 (m, 1H), 1.85 – 1.79 (m, 2H), 1.71 (d, J = 13.3 Hz, 1H), 1.58 (d, J = 11.8 Hz, 1H), 1.36 – 1.24 (m, 3H), 1.21 – 1.11 (m, 2H).
^{13}C NMR (151 MHz, $CDCl_3$) δ 156.37, 144.97 (dm, J = 252.2 Hz), 130.58, 129.56, 128.98, 128.21, 122.79 (q, J = 280.1 Hz), 122.16 (t, J = 14.1 Hz) 116.18 (t, J = 14.2 Hz), 114.82 (t, J = 240.3 Hz), 74.40 (q, J = 34.1 Hz), 66.07 (d, J = 6.0 Hz), 37.74, 31.24, 29.36 (t, J = 23.5 Hz), 27.58, 26.39, 25.79, 25.69.
^{19}F NMR (565 MHz, $CDCl_3$) δ -75.85 (d, J = 6.3 Hz), -114.23 – -118.13 (m), -134.13 – -134.19 (m).
HRMS (ESI) (m/z): calcd for $C_{24}H_{21}F_9NaO_4S$ ($[M + Na]^+$): 599.0909; found: 599.0912.

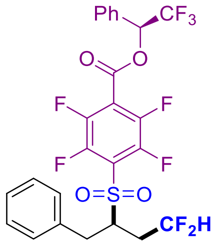

(S)-2,2,2-trifluoro-1-phenylethyl 4-(((R)-4,4-difluoro-1-phenylbutan-2-yl) sulfonyl)-2,3,5,6-tetra-fluorobenzoate (101)
This compound was obtained in 71% (83.0 mg) yield as yellow oil by the general procedure.
^{1}H NMR (600 MHz, $CDCl_3$) δ 7.53 (d, J = 8.0 Hz, 2H), 7.51 – 7.46 (m, 3H), 7.20 (dd, J = 8.1, 7.0 Hz, 2H), 7.15 – 7.10 (m, 3H), 6.34 (q, J = 6.5, 5.8 Hz, 1H), 6.09 (tt, J = 56.3, 4.8 Hz, 1H), 3.96 (dq, J = 8.7, 6.1 Hz, 1H), 3.28 (dd, J = 14.9, 8.7 Hz, 1H), 3.18 (dd, J = 14.8, 6.1 Hz, 1H), 2.84 – 2.70 (m, 1H), 2.35 – 2.21 (m, 1H).

^{13}C NMR (151 MHz, $CDCl_3$) δ 156.13, 144.37 (dm, J = 261.9 Hz), 134.70, 130.60, 129.57, 128.80, 128.78, 128.12, 127.81, 122.57 (q, J = 280.6 Hz), 121.78 (t, J = 14.1 Hz), 155.89 (t, J = 15.1 Hz), 114.89 (t, J = 241.3 Hz), 74.31 (q, J = 34.7 Hz), 62.36 (t, J = 4.3 Hz), 36.12, 32.25 (t, J = 23.6 Hz).
^{19}F NMR (565 MHz, $CDCl_3$) δ -75.97 (d, J = 6.6 Hz), -113.52 – -118.23 (m), -133.24 – -133.54 (m), -134.72 – -135.00 (m).
HRMS (ESI) (m/z): calcd for $C_{25}H_{17}F_9NaO_4S$ ($[M + Na]^+$): 607.0596; found: 607.0588.

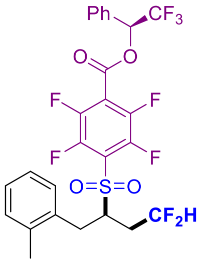

(S)-2,2,2-trifluoro-1-phenylethyl 4-(((R)-4,4-difluoro-1-(o-tolyl)butan-2-yl)sulfonyl)-2,3,5,6-tetra-fluorobenzoate (102)
This compound was obtained in 69% (82.6 mg) yield as yellow oil by the general procedure.
^{1}H NMR (600 MHz, $CDCl_3$) δ 7.53 (d, J = 6.0 Hz, 2H), 7.51 – 7.44 (m, 3H), 7.09 (d, J = 6.6 Hz, 1H), 7.06 – 6.98 (m, 3H), 6.34 (q, J = 6.5 Hz, 1H), 6.21 (tdd, J = 56.3, 6.0, 3.3 Hz, 1H), 4.06 – 3.98 (m, 1H), 3.37 (dd, J = 14.8, 8.8 Hz, 1H), 3.13 (dd, J = 14.8, 6.5 Hz, 1H), 2.86 – 2.71 (m, 1H), 2.30 (s, 3H), 2.29 – 2.19 (m, 1H).
^{13}C NMR (151 MHz, $CDCl_3$) δ 156.15, 144.37 (dm, J = 268.1 Hz), 136.63, 132.69, 130.86, 130.60, 129.92 (d, J = 3.3 Hz), 129.59, 129.00, 128.12, 128.03, 123.50 (q, J = 280.0 Hz), 121.91 (t, J = 14.1 Hz), 115.73 (t, J = 13.5 Hz), 114.58 (t, J = 243.5 Hz), 74.29 (q, J = 34.9 Hz), 60.93, 33.74, 32.33 (t, J = 23.6 Hz), 19.08.
^{19}F NMR (565 MHz, $CDCl_3$) δ -75.97 (d, J = 6.6 Hz), -113.33 – -118.19 (m), -132.32 – -133.80 (m), -134.49 – -135.32 (m).
HRMS (ESI) (m/z): calcd for $C_{26}H_{19}F_9NaO_4S$ ($[M + Na]^+$): 621.0953; found: 621.0956.

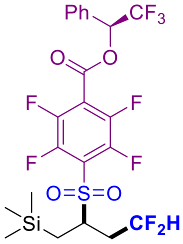

(S)-2,2,2-trifluoro-1-phenylethyl 4-(((S)-4,4-difluoro-1-(trimethylsilyl)butan-2-yl)sulfonyl)-2,3,5,6-tetrafluorobenzoate (103)
This compound was obtained in 88% (102.9 mg) yield as yellow oil by the general procedure.
^{1}H NMR (600 MHz, $CDCl_3$) δ 7.42 (d, J = 6.5 Hz, 2H), 7.39 – 7.32 (m, 3H), 6.26 (q, J = 6.5 Hz, 1H), 6.12 (tdd, J = 56.5, 6.5, 3.5 Hz, 1H), 3.45 (dd, J = 12.0, 8.5 Hz, 1H), 2.51 – 2.32 (m, 1H), 2.16 – 2.02 (m, 1H), 1.04 (dt, J = 14.5, 2.0 Hz, 1H), 0.87 (dd, J = 14.5, 12.0 Hz, 1H), 0.01 (s, 9H).

^{13}C NMR (151 MHz, $CDCl_3$) δ 156.54, 146.20 (d, J = 22.1 Hz), 144.45 (d, J = 19.7 Hz), 130.81, 129.75, 129.20, 128.41, 122.76 (q, J = 280.6 Hz), 120.78 (t, J = 14.9 Hz), 116.49 (t, J = 15.7 Hz), 114.67 (t, J = 240.0 Hz), 74.65 (q, J = 34.0 Hz), 59.85 (d, J = 7.4 Hz), 34.70 (t, J = 23.8 Hz), 16.51, -0.99.
^{19}F NMR (565 MHz, $CDCl_3$) δ -75.85 (dd, J = 6.5, 1.5 Hz), -112.45 – -118.58 (m), -133.05 (tdd, J = 15.5, 6.0, 2.5 Hz), -133.71 – -134.26 (m).
HRMS (ESI) (m/z): calcd for $C_{22}H_{21}F_9NaO_4SSi$ ($[M + Na]^+$): 603.0678; found: 603.0682.

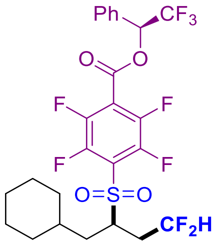

(S)-2,2,2-trifluoro-1-phenylethyl 4-(((R)-1-cyclohexyl-4,4-difluorobutan-2-yl)sulfonyl)-2,3,5,6-tetrafluorobenzoate (104)
This compound was obtained in 74% (87.4 mg) yield as yellow oil by the general procedure.
^{1}H NMR (600 MHz, $CDCl_3$) δ 7.57 – 7.53 (m, 2H), 7.47 (tt, J = 7.6, 5.0 Hz, 3H), 6.37 (q, J = 6.4 Hz, 1H), 6.23 (tt, J = 55.5, 4.5 Hz, 1H), 3.58 (m, 1H), 2.63 – 2.44 (m, 1H), 2.31 – 2.16 (m, 1H), 1.75 – 1.65 (m, 6H), 1.61 (ddd, J = 14.3, 9.6, 4.7 Hz, 1H), 1.47 – 1.39 (m, 1H), 1.30 – 1.19 (m, 2H), 1.14 (tt, J = 12.7, 3.0 Hz, 1H), 1.02 – 0.92 (m, 1H), 0.81 – 0.68 (m, 1H).
^{13}C NMR (151 MHz, $CDCl_3$) δ 156.30, 145.04 (dm, J = 262.7 Hz), 130.61, 129.53, 128.99, 128.21, 122.55 (q, J = 280.9 Hz), 120.87 (t, J = 14.5 Hz), 116.33 (t, J = 15.3 Hz), 114.43 (t, J = 240.4 Hz), 74.44 (q, J = 34.1 Hz), 58.94 (d, J = 5.6 Hz), 35.85, 34.32, 33.67, 32.90 (t, J = 23.6 Hz), 31.80, 26.09, 26.00, 25.71.
^{19}F NMR (565 MHz, $CDCl_3$) δ -75.86 (d, J = 6.8 Hz), -113.61 – -118.03 (m), -133.30 – -133.50 (m), -133.88 – -134.03 (m).
HRMS (ESI) (m/z): calcd for $C_{25}H_{23}F_9NaO_4S$ ($[M + Na]^+$): 613.1066; found: 613.1054.

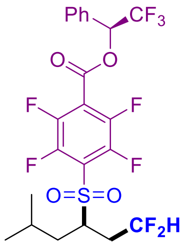

(S)-2,2,2-trifluoro-1-phenylethyl 4-(((R)-1,1-difluoro-5-methylhexan-3-yl)sulfonyl)-2,3,5,6-tetra-fluorobenzoate (105)
This compound was obtained in 91% (100.2 mg) yield as white solid by the general procedure.
^{1}H NMR (600 MHz, $CDCl_3$) δ 7.54 (d, J = 7.0 Hz, 2H), 7.52 – 7.39 (m, 3H), 6.38 (q, J = 6.5 Hz, 1H), 6.18 (tt, J = 50.5, 4.5 Hz, 1H), 3.54 (q, J = 8.6 Hz, 1H), 2.60 – 2.46 (m, 1H), 2.35 – 2.17 (m, 1H), 1.84 – 1.76 (m, 1H), 1.74 – 1.69 (m, 1H), 1.67 – 1.61 (m, 1H), 0.99 (d, J = 6.5 Hz, 3H), 0.88 (d, J = 6.5 Hz, 3H).

^{13}C NMR (151 MHz, $CDCl_3$) δ 156.30, 144.69 (dm, J = 270.2 Hz), 130.61, 129.52, 129.00, 128.21, 122.54 (q, J =280.7 Hz), 120.83 (t, J = 14.4 Hz), 116.37 (t, J = 15.3 Hz), 114.39 (t, J = 240.6 Hz), 74.45 (q, J = 34.1 Hz), 59.66 (t, J = 4.9 Hz), 37.22, 32.86 (t, J = 23.6 Hz), 25.16, 22.94, 21.01.
^{19}F NMR (565 MHz, $CDCl_3$) δ -75.85 (d, J = 6.5 Hz), -113.66 – -118.11 (m), -133.34 – -133.49 (m), -133.81 – -134.03 (m).
HRMS (ESI) (m/z): calcd for $C_{22}H_{19}F_9NaO_4S$ ($[M + Na]^+$): 573.0753; found: 573.0746.

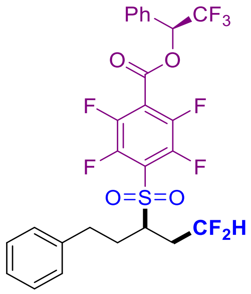

(S)-2,2,2-trifluoro-1-phenylethyl 4-(((R)-1,1-difluoro-5-phenylpentan-3-yl)sulfonyl)-2,3,5,6-tetra-fluorobenzoate (106)
This compound was obtained in 90% (107.7 mg) yield as yellow oil by the general procedure.
^{1}H NMR (600 MHz, $CDCl_3$) δ 7.56 (d, J = 6.0 Hz, 2H), 7.52 – 7.44 (m, 3H), 7.23 – 7.19 (m, 2H), 7.16 (q, J = 7.2, 6.7 Hz, 1H), 7.01 (d, J = 6.0 Hz, 2H), 6.38 (q, J = 6.5 Hz, 1H), 6.21 (tdd, J = 56.3, 6.0, 3.3 Hz, 1H), 3.40 – 3.35 (m, 1H), 2.98 – 2.88 (m, 1H), 2.74 – 2.63 (m, 1H), 2.62 – 2.54 (m, 1H), 2.37 – 2.25 (m, 1H), 2.17 – 2.06 (m, 2H).
^{13}C NMR (151 MHz, $CDCl_3$) δ 156.25, 144.77 (dm, J = 261.4 Hz), 138.78, 129.54, 129.03, 128.73, 128.28, 128.18, 126.86, 125.73 (q, J = 281.8 Hz), 114.46 (t, J = 240.6 Hz), 74.44 (q, J = 35.6 Hz), 58.89, 32.29 (t, J = 23.4 Hz), 31.74 31.01.
^{19}F NMR (565 MHz, $CDCl_3$) δ -75.86 (d, J = 6.5 Hz), -112.96 – -118.45 (m), -133.59 – -133.78 (m), -134.08 – -134.31 (m).
HRMS (ESI) (m/z): calcd for $C_{26}H_{20}F_9O_4S$ ($[M + H]^+$): 599.0933; found: 599.0924.

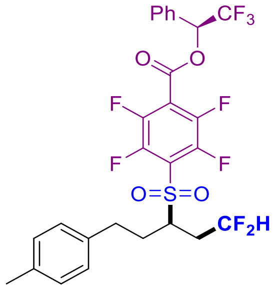

(S)-2,2,2-trifluoro-1-phenylethyl 4-(((R)-1,1-difluoro-5-(p-tolyl)pentan-3-yl)sulfonyl)-2,3,5,6-tetra-fluorobenzoate (107)
This compound was obtained in 92% (112.7 mg) yield as white solid by the general procedure.
^{1}H NMR (600 MHz, $CDCl_3$) δ 7.55 (d, J = 6.5 Hz, 2H), 7.51 – 7.45 (m, 3H), 6.95 (d, J = 8.4 Hz, 1H), 6.79 (d, J = 8.2 Hz, 1H), 6.62 (s, 2H), 6.38 (q, J = 6.5 Hz, 1H), 6.23 (tdd, J = 56.4, 5.7, 2.5 Hz, 1H), 3.46 – 3.41 (m, 1H), 2.86 – 2.80 (m, 1H), 2.75 – 2.60 (m, 1H), 2.48 (ddd, J = 14.7, 9.2, 6.1 Hz, 1H), 2.36 – 2.26 (m, 1H), 2.20 (s, 2H), 2.14

– 1.99 (m, 2H).

^{13}C NMR (151 MHz, $CDCl_3$) δ 156.25, 144.72 (dm, J = 278.4 Hz), 138.73, 138.46, 130.61, 129.56, 128.99, 128.37, 128.23, 126.11, 122.59 (q, J = 276.1 Hz), 121.66, 120.49 (t, J = 14.3 Hz), 114.67 (t, J = 240.2 Hz), 74.42 (q, J = 33.9 Hz), 59.04, 32.26 (t, J = 24.2 Hz), 31.61, 31.09, 20.96.

^{19}F NMR (565 MHz, $CDCl_3$) δ -75.74 (t, J = 6.8 Hz), -113.02 – -118.32 (m), -133.54 – -133.95 (m), -134.14 – -134.54 (m).

HRMS (ESI) (m/z): calcd for $C_{27}H_{21}F_9NaO_4S$ ([M + Na]$^+$): 635.0909; found: 635.0916.

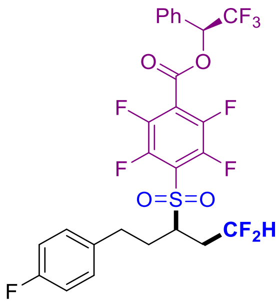

(S)-2,2,2-trifluoro-1-phenylethyl 4-(((R)-1,1-difluoro-5-(4-fluorophenyl)pentan-3-yl)sulfonyl)-2,3,5,6-tetrafluorobenzoate (108)

This compound was obtained in 88% (108.5 mg) yield as white solid by the general procedure.

^{1}H NMR (600 MHz, $CDCl_3$) δ 7.54 (d, J = 8.5 Hz, 2H), 7.51 – 7.45 (m, 3H), 7.02 (t, J = 6.5 Hz, 2H), 6.93 (td, J = 8.5, 5.8 Hz, 2H), 6.38 (q, J = 6.5 Hz, 1H), 6.17 (tdd, J = 56.2, 5.1, 2.5 Hz, 1H), 3.43 – 3.38 (m, 1H), 2.91 (ddd, J = 13.4, 7.2, 5.5 Hz, 1H), 2.65 – 2.54 (m, 2H), 2.36 – 2.24 (m, 1H), 2.16 – 2.05 (m, 2H).

^{13}C NMR (151 MHz, $CDCl_3$) δ 162.52, 156.19, 144.63 (dm, J = 241.7 Hz), 134.59 (t, J = 3.0 Hz), 130.62, 129.85, 129.80, 129.52, 129.01, 128.20 (d, J = 4.5 Hz), 122.46 (q, J = 281.7 Hz), 120.39 (t, J = 15.1 Hz), 116.47 (t, J = 15.6 Hz), 115.68, 114.40 (t, J = 240.7 Hz), 74.52 (q, J = 34.1 Hz), 59.14, 32.31 (t, J = 23.7 Hz), 31.03, 30.82.

^{19}F NMR (565 MHz, $CDCl_3$) δ -75.85 (t, J = 5.2 Hz), -112.60 – -114.64 (m), -115.55 (ddd, J = 24.2, 9.4, 4.3 Hz), -116.76 – -118.99 (m), -133.42 – -133.67 (m), -133.93 – -134.27 (m).

HRMS (ESI) (m/z): calcd for $C_{26}H_{18}F_{10}NaO_4S$ ([M + Na]$^+$): 639.0658; found: 639.0559.

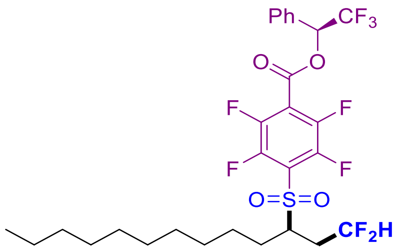

(S)-2,2,2-trifluoro-1-phenylethyl 4-(((R)-1,1-difluorotridecan-3-yl)sulfonyl)-2,3,5,6-tetrafluoro-benzoate (109)

This compound was obtained in 50% (63.5 mg) yield as yellow oil by the general procedure.

^{1}H NMR (600 MHz, $CDCl_3$) δ 7.54 (d, J = 6.5 Hz, 2H), 7.52 – 7.41 (m, 3H), 6.37 (q, J = 6.5 Hz, 1H), 6.16 (tdd, J = 56.2, 5.3, 3.6 Hz, 1H), 3.46 (tt, J = 9.3, 4.9 Hz, 1H), 2.64 – 2.45 (m, 1H), 2.37 – 2.23 (m, 1H), 1.96 – 1.86 (m, 1H), 1.78 – 1.70 (m, 1H), 1.46 – 1.23 (m, 16H), 0.88 (t, J = 7.0 Hz, 3H).
^{13}C NMR (151 MHz, $CDCl_3$) δ 156.29, 145.14 (dm, J = 250.5 Hz), 130.61, 129.52, 129.00, 128.19, 122.33 (q, J = 279.5 Hz), 120.96 (t, J = 15.5 Hz), 116.35 (t, J = 15.1 Hz), 114.41 (t, J = 240.5 Hz), 74.44 (q, J = 34.1 Hz), 61.23 (t, J = 5.6 Hz), 32.28 (t, J = 23.9 Hz), 31.84, 29.39, 29.23, 29.13, 29.08, 28.33, 25.99, 22.65, 14.08.
^{19}F NMR (565 MHz, $CDCl_3$) δ -75.86 (d, J = 6.5 Hz), -113.95 – -118.22 (m), -133.11 – -133.72 (m), -133.74 – -134.14 (m).
HRMS (ESI) (m/z): calcd for $C_{28}H_{32}F_9O_4S$ ($[M + H]^+$): 635.1872; found: 635.1869.

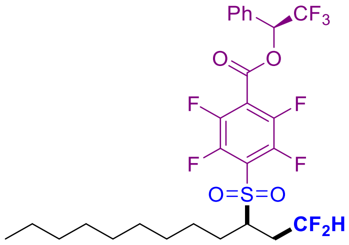

(S)-2,2,2-trifluoro-1-phenylethyl 4-(((R)-1,1-difluorododecan-3-yl)sulfonyl)-2,3,5,6-tetrafluoro-benzoate (110)

This compound was obtained in 58% (72.0 mg) yield as yellow oil by the general procedure.
^{1}H NMR (600 MHz, $CDCl_3$) δ 7.53 (d, J = 8.5 Hz, 2H), 7.51 – 7.44 (m, 3H), 6.37 (q, J = 6.5 Hz, 1H), 6.16 (tdd, J = 56.2, 5.3, 3.6 Hz, 1H), 3.46 (tt, J = 9.0, 4.8 Hz, 1H), 2.63 – 2.46 (m, 1H), 2.38 – 2.22 (m, 1H), 1.99 – 1.86 (m, 1H), 1.81 – 1.70 (m, 1H), 1.57 – 1.46 (m, 1H), 1.44 – 1.18 (m, 13H), 0.87 (t, J = 7.0 Hz, 3H).
^{13}C NMR (151 MHz, $CDCl_3$) δ 156.29, 145.06 (dm, J = 246.2 Hz), 130.61, 129.52, 129.00, 128.20, 122.47 (q, J = 281.6 Hz), 120.90 (t, J = 15.5 Hz), 116.35 (t, J = 16.0 Hz), 114.23 (t, J = 246.3 Hz), 74.44 (q, J = 34.3 Hz), 61.22 (t, J = 4.6 Hz), 32.19 (t, J = 23.6 Hz), 31.78, 29.34, 29.17, 29.13, 29.07, 28.33, 25.98, 22.62.
^{19}F NMR (565 MHz, $CDCl_3$) δ -75.87 (d, J = 6.4 Hz), -113.95 – -117.97 (m), -133.06 – -133.66 (m), -133.94 – -134.06 (m).
HRMS (ESI) (m/z): calcd for $C_{27}H_{29}F_9NaO_4S$ ($[M + Na]^+$): 643.1535; found: 643.1528.

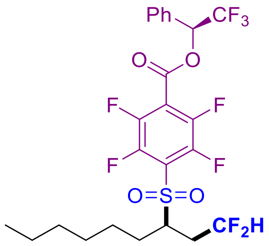

(S)-2,2,2-trifluoro-1-phenylethyl 4-(((R)-1,1-difluorononan-3-yl)sulfonyl)-2,3,5,6-tetrafluoro-enzoate (111)

This compound was obtained in 77% (89.1 mg) yield as yellow oil by the general procedure.
^{1}H NMR (600 MHz, $CDCl_3$) δ 7.58 – 7.52 (m, 2H) 7.51 – 7.42 (m, 3H),6.37 (q, J = 6.5 Hz, 1H), 6.15 (tdd, J = 56.2, 5.2, 3.6 Hz, 1H), 3.51 – 3.42 (m, 1H), 2.60 – 2.44 (m, 1H),

2.35 – 2.21 (m, 1H), 1.94 (ddt, *J* = 15.1, 10.4, 5.1 Hz, 1H), 1.84 – 1.69 (m, 1H), 1.57 – 1.49 (m, 1H), 1.43 – 1.23 (m, 7H),0.87 (t, *J* = 7.0 Hz, 3H).

^{13}C NMR (151 MHz, $CDCl_3$) δ 156.29, 145.21 (dm, *J* = 241.6 Hz), 130.59, 129.53, 128.99, 128.19, 122.55 (q, *J* = 280.7 Hz), 120.96 (t, *J* = 14.6 Hz), 116.35 (t, *J* = 15.5 Hz), 114.43 (t, *J* = 240.6 Hz), 74.43 (q, *J* = 34.1 Hz), 61.22 (t, *J* = 5.2 Hz), 32.18 (t, *J* = 23.5 Hz), 31.28, 28.70, 28.31, 25.92, 22.41, 13.90.

^{19}F NMR (565 MHz, $CDCl_3$) δ -75.89 (d, *J* = 6.3 Hz), -114.04 – -117.97 (m), -133.38 – -133.62 (m), -133.95 – -134.15 (m).

HRMS (ESI) (m/z): calcd for $C_{24}H_{23}F_9NaO_4S$ ($[M + Na]^+$): 601.1066; found: 601.1065.

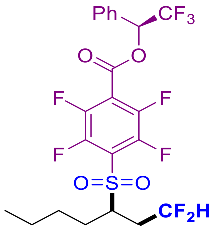

(S)-2,2,2-trifluoro-1-phenylethyl 4-(((R)-1,1-difluoroheptan-3-yl)sulfonyl)-2,3,5,6-tetrafluoro-benzoate (112)

This compound was obtained in 65% (71.6 mg) yield as yellow oil by the general procedure.

^{1}H NMR (600 MHz, $CDCl_3$) δ 7.55 – 7.52 (m, 2H), 7.51 – 7.44 (m, 3H), 6.37 (q, *J* = 6.5 Hz, 1H), 6.15 (tdd, *J* = 56.2, 5.2, 3.6 Hz, 1H), 3.53 – 3.40 (m, 1H), 2.60 – 2.45 (m, 1H), 2.32 – 2.20 (m, 1H), 1.99 – 1.89 (m, 1H), 1.84 – 1.72 (m, 1H), 1.55 – 1.48 (m, 1H), 1.44 – 1.30 (m, 3H), 0.91 (t, *J* = 7.2 Hz, 3H).

^{13}C NMR (151 MHz, $CDCl_3$) δ 156.29, 144.91 (dm, *J* = 245.8 Hz), 130.60, 129.53, 128.99, 128.19, 122.54 (q, *J* = 280.6 Hz), 120.91 (d, *J* = 14.8 Hz), 116.36 (t, *J* = 15.6 Hz), 114.41 (t, *J* = 240.6 Hz), 74.44 (q, *J* = 34.0 Hz), 61.19 (d, *J* = 5.2 Hz), 32.21 (t, *J* = 23.6 Hz), 28.06, 22.24, 13.59.

^{19}F NMR (565 MHz, $CDCl_3$) δ -75.87 (d, *J* = 6.2 Hz), -113.75 – -118.05 (m), -133.04 – -133.59 (m), -133.72 – -134.24 (m).

HRMS (ESI) (m/z): calcd for $C_{22}H_{19}F_9NaO_4S$ ($[M + Na]^+$): 573.0753; found: 573.0752.

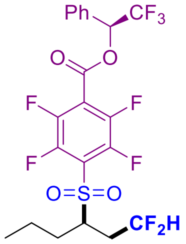

(S)-2,2,2-trifluoro-1-phenylethyl 4-(((R)-1,1-difluorohexan-3-yl)sulfonyl)-2,3,5,6-tetrafluoro-benzoate (113)

This compound was obtained in 87% (93.2 mg) yield as white solid by the general procedure.

^{1}H NMR (600 MHz, $CDCl_3$) δ 7.57 – 7.51 (m, 2H), 7.51 – 7.42 (m, 3H), 6.37 (q, *J* = 6.5 Hz, 1H), 6.15 (tdd, *J* = 56.2, 5.2, 3.6 Hz, 1H), 3.48 (tt, *J* = 9.0, 4.9 Hz, 1H), 2.58 – 2.47 (m, 1H), 2.36 – 2.21 (m, 1H), 1.96 – 1.87 (m, 1H), 1.83 – 1.72 (m, 1H), 1.61 (ddd, *J* = 8.9, 7.4, 4.0 Hz, 1H), 1.52 – 1.42 (m, 1H), 0.96 (t, *J* = 7.3 Hz, 3H).

^{13}**C NMR** (151 MHz, $CDCl_3$) δ 156.30, 145.01 (dm, J = 241.4 Hz), 130.60, 129.53, 128.99, 128.19, 122.55 (q, J = 280.6 Hz), 120.94 (t, J = 14.4 Hz), 116.36 (t, J = 15.6 Hz), 114.41 (t, J = 240.6 Hz), 74.44 (q, J = 34.1 Hz), 61.02 (t, J = 5.0 Hz), 32.20 (t, J = 23.5 Hz), 30.32, 19.36, 13.53.
^{19}**F NMR (**565 MHz, $CDCl_3$) δ -75.87 (d, J = 6.4 Hz), -113.74 – -118.60 (m), -133.35 – -133.59 (m), -133.77 – -134.15 (m).
HRMS (ESI) (m/z): calcd for $C_{21}H_{17}F_9NaO_4S$ ($[M + Na]^+$): 559.0596; found: 559.0585.

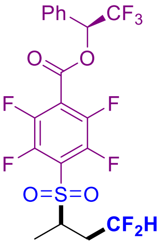

(S)-2,2,2-trifluoro-1-phenylethyl 4-(((R)-4,4-difluorobutan-2-yl)sulfonyl)-2,3,5,6-tetrafluoro-benzoate (114)
This compound was obtained in 63% (64.1 mg) yield as yellow oil by the general procedure.
^{1}**H NMR** (600 MHz, $CDCl_3$) δ 7.55 – 7.52 (m, 2H), 7.51 – 7.40 (m, 3H), 6.37 (q, J = 6.5 Hz, 1H), 6.21 – 5.99 (m, 1H), 3.68 – 3.57 (m, 1H), 2.67 – 2.54 (m, 1H), 2.28 – 2.12 (m, 1H), 1.53 (d, J = 7.0 Hz, 3H).
^{13}**C NMR** (151 MHz, $CDCl_3$) δ 156.25, 145.07 (dm, J = 248.4 Hz), 130.61, 129.51, 129.00, 128.18, 121.05 (q, J = 281.6 Hz), 116.51 (t, J = 15.6 Hz), 115.22 (t, J = 241.4 Hz), 114.57 (t, J = 243.2 Hz), 112.22 (t, J = 22.5 Hz), 75.37 – 73.74 (m), 56.81 (t, J = 4.8 Hz), 56.54 (t, J = 4.9 Hz), 33.40 (t, J = 23.1 Hz), 33.31 (t, J = 22.9 Hz), 13.95.
^{19}**F NMR** (565 MHz, $CDCl_3$) δ -75.89 (d, J = 6.8 Hz), -114.17 – -118.00 (m), -133.00 – -133.23 (m), -133.91 – -134.05 (m).
HRMS (ESI) (m/z): calcd for $C_{19}H_{13}F_9NaO_4S$ ($[M + Na]^+$): 531.0283; found: 531.0276.

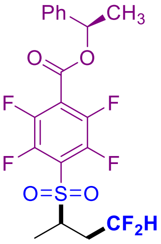

(R)-1-phenylethyl 4-(((R)-4,4-difluorobutan-2-yl)sulfonyl)-2,3,5,6-tetrafluorobenzoate (115)
This compound was obtained in 80% (73.6 mg) yield as yellow oil by the general procedure.
^{1}**H NMR** (600 MHz, $CDCl_3$) δ 7.45 – 7.38 (m, 4H), 7.36 – 7.33 (m, 1H), 6.24 – 5.98 (m, 2H), 5.85 (td, J = 56.0, 2.7 Hz, 0.1H), 3.69 –3.64 (m, 0.07H), 3.63 – 3.55 (m, 1H), 2.66 – 2.52 (m, 1H), 2.24 – 2.11 (m, 1H), 1.71 (d, J = 6.6 Hz, 3H), 1.50 (d, J = 6.9 Hz, 2.8H)., 1.30 (d, J = 7.0 Hz, 0.18H).
^{13}**C NMR** (151 MHz, $CDCl_3$) δ 157.35, 144.68 (dm, J = 251.2 Hz), 139.74, 128.75, 128.64, 126.25, 118.72 (d, J = 17.0 Hz), 114.49 (t, J = 241.2 Hz), 56.73 (t, J = 5.0 Hz), 33.33 (t, J = 22.9 Hz), 21.98, 13.92.

^{19}**F NMR** (565 MHz, CDCl3) δ -116.06 (dddt, *J* = 1695.0, 286.4, 55.8, 16.7 Hz), -133.75 – -133.90 (m), -135.44 – -135.58 (m).
HRMS (ESI) (m/z): calcd for $C_{19}H_{16}F_6NaO_4S$ ([M + Na]$^+$): 477.0566; found: 477.0562.

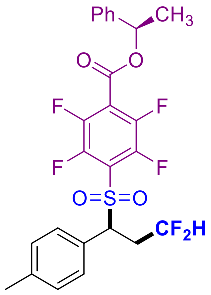

(R)-1-phenylethyl 4-(((S)-3,3-difluoro-1-(p-tolyl)propyl)sulfonyl)-2,3,5,6-tetrafluorobenzoate (116)
This compound was obtained in 76% (80.6 mg) yield as yellow oil by the general procedure.
^{1}**H NMR** (600 MHz, CDCl3) δ 7.43 – 7.37 (m, 4H), 7.35 (d, *J* = 6.4 Hz, 1H), 7.18 (d, *J* = 6.9 Hz, 2H), 7.13 (d, *J* = 7.4 Hz, 2H), 6.14 (q, *J* = 6.6 Hz, 1H), 5.75 (tdd, *J* = 55.8, 6.0, 3.5 Hz, 1H), 4.55 (dd, *J* = 10.5, 4.6 Hz, 1H), 3.03 – 2.87 (m, 1H), 2.82 – 2.69 (m, 1H), 2.32 (s, 3H), 1.68 (d, *J* = 6.6 Hz, 3H).
^{13}**C NMR** (151 MHz, CDCl3) δ 157.42, 144.31 (dm, *J* = 244.2 Hz), 140.72, 139.77, 130.18, 129.07, 128.73, 128.63, 126.25, 119.30 (t, *J* = 14.4 Hz), 118.24 (t, *J* = 16.7 Hz), 114.51 (t, *J* = 241.5 Hz), 76.40, 66.98 (t, *J* = 5.4 Hz), 32.52 (t, *J* = 24.1 Hz), 21.99, 21.20.
^{19}**F NMR** (565 MHz, CDCl3) δ -116.72 (ddt, *J* = 56.1, 50.3, 13.4 Hz), -133.75 – -134.36 (m), -136.07 – -136.46 (m).
HRMS (ESI) (m/z): calcd for $C_{25}H_{20}F_6NaO_4S$ ([M + Na]$^+$): 531.1059; found: 531.1054.

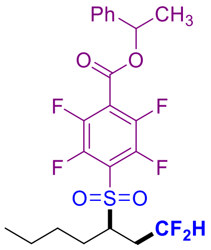

1-phenylethyl 4-(((R)-1,1-difluoroheptan-3-yl)sulfonyl)-2,3,5,6-tetrafluoro-benzoate (117)
This compound was obtained in 61% (60.5 mg) yield as yellow oil by the general procedure. The dr value is determined by ^{1}H NMR.
^{1}**H NMR** (600 MHz, CDCl3) δ 7.44 – 7.38 (m, 8H), 7.37 – 7.33 (m, 2H), 6.29 – 6.03 (m, 4H), 3.44 (dq, *J* = 10.5, 5.0 Hz, 2H), 2.57 – 2.45 (m, 2H), 2.26 (tdt, *J* = 15.6, 10.4, 5.0 Hz, 2H), 1.90 (dtd, *J* = 20.1, 10.0, 5.3 Hz, 2H), 1.76 – 1.69 (m, 9H), 1.63 – 1.56 (m, 1H), 1.53 – 1.48 (m, 1H), 1.44 – 1.25 (m, 5H), 0.94 (t, *J* = 7.3 Hz, 3H), 0.90 (t, *J* = 7.2 Hz, 3H).
^{13}**C NMR** (151 MHz, CDCl3) δ 157.41 , 145.87 – 145.14 (m), 144.28 – 143.33 (m), 139.74 , 128.77 , 128.67 , 126.28 , 119.60 (d, *J* = 14.6 Hz), 118.71 (d, *J* = 16.8 Hz),

114.47 (t, J = 240.6 Hz), 61.15 (d, J = 4.6 Hz), 76.53, 60.94 (t, J = 4.5 Hz), 32.18 (t, J = 23.5 Hz), 30.36 , 28.06 , 22.24 , 21.98 , 19.37 , 13.61 , 13.55 .

^{19}F NMR (565 MHz, $CDCl_3$) δ -113.77 – -118.19 (m), -134.02 – -134.28 (m), -135.23 – -135.57 (m).

HRMS (ESI) (m/z): calcd for $C_{22}H_{22}F_6NaO_4S$ ($[M + Na]^+$): 519.1036; found: 519.1044.

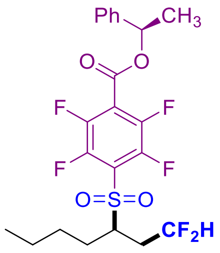

(R)-1-phenylethyl 4-(((R)-1,1-difluoroheptan-3-yl)sulfonyl)-2,3,5,6-tetrafluoro-benzoate (117')

^{1}H NMR (600 MHz, $CDCl_3$) δ 7.41 (dd, J = 13.8, 7.2 Hz, 4H), 7.37 – 7.33 (m, 1H), 6.28 – 6.04 (m, 2H), 3.44 (dq, J = 7.5, 4.5 Hz, 1H), 2.51 (dddd, J = 25.1, 12.5, 6.9, 3.5 Hz, 1H), 2.27 (tdt, J = 15.6, 10.5, 5.3 Hz, 1H), 1.92 (ddt, J = 15.2, 10.4, 4.9 Hz, 1H), 1.75 (dt, J = 9.8, 4.7 Hz, 1H), 1.71 (d, J = 6.6 Hz, 3H), 1.51 (tdd, J = 9.8, 6.2, 4.5 Hz, 1H), 1.34 (ddddd, J = 29.4, 22.1, 15.4, 7.0, 4.7 Hz, 3H), 0.94 (t, J = 7.5 Hz, 0.2H), 0.90 (t, J = 7.2 Hz, 3H).

^{13}C NMR (151 MHz, $CDCl_3$) δ 157.41, 146.20 – 145.06 (m), 144.73 – 143.52 (m), 139.76, 128.76, 128.66, 126.28, 119.65 (t, J = 14.5 Hz), 118.64 (t, J = 16.9 Hz), 114.49 (t, J = 240.6 Hz), 76.52, 61.14 (t, J = 5.0 Hz), 32.18 (t, J = 23.5 Hz), 28.06, 22.24, 21.98, 13.60.

^{19}F NMR (565 MHz, Chloroform-*d*) δ -114.00 – -118.28 (m), -133.89 – -134.30 (m), -135.27 – -135.66 (m).

HRMS (ESI) (m/z): calcd for $C_{22}H_{22}F_6NaO_4S$ ($[M + Na]^+$): 519.1036; found: 519.1029.

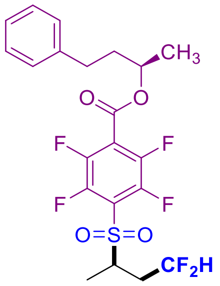

(R)-4-phenylbutan-2-yl 4-(((R)-4,4-difluorobutan-2-yl)sulfonyl)-2,3,5,6-tetra-fluorobenzoate (118)

This compound was obtained in 75% (72.4 mg) yield as yellow oil by the general procedure.

^{1}H NMR (600 MHz, $CDCl_3$) δ 7.29 (t, J = 7.4 Hz, 2H), 7.22 – 7.16 (m, 3H), 6.10 (tt, J = 55.8, 4.1 Hz, 1H), 5.87 (td, J = 56.0, 2.6 Hz, 0.1H), 5.41 – 5.20 (m, 1H), 3.67 (dd, J = 14.4, 4.4 Hz, 0.07H), 3.61 (td, J = 7.5, 5.0 Hz, 1H), 2.77 (td, J = 9.7, 5.0 Hz, 1H), 2.69 (ddd, J = 13.9, 9.7, 6.6 Hz, 1H), 2.65 – 2.55 (m, 1H), 2.24 – 2.10 (m, 1H), 2.12 – 2.02 (m, 1H), 2.00 – 1.91 (m, 1H), 1.51 (d, J = 6.9 Hz, 3H), 1.42 (d, J = 6.3 Hz, 3H), 1.32 (d, J = 7.0 Hz, 0.2H).

^{13}C NMR (151 MHz, $CDCl_3$) δ 157.68, 146.28 – 144.96 (m), 144.18 – 143.36 (m), 140.82, 128.54, 128.30, 126.16, 119.19 (t, J = 17.8 Hz), 118.70 (t, J = 14.5 Hz), 114.50 (t, J = 241.2 Hz), 75.04, 56.74 (t, J = 4.8 Hz), 37.34, 33.35 (t, J = 22.9 Hz), 31.52, 19.90, 13.95.
^{19}F NMR (565 MHz, $CDCl_3$) δ -113.53 – -118.25 (m), -132.55 – -134.28 (m), -134.99 – -135.19 (m), -135.59 – -136.32 (m), -136.05 (dd, J = 19.4, 8.0 Hz).
HRMS (ESI) (m/z): calcd for $C_{21}H_{20}F_6NaO_4S$ ($[M + Na]^+$): 505.0879; found: 505.0867.

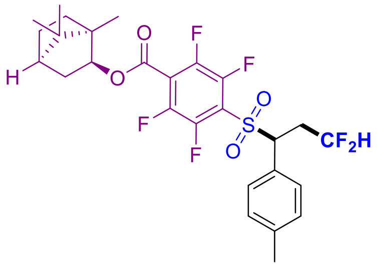

(1R,2S,4R)-1,7,7-trimethylbicyclo[2.2.1]heptan-2-yl 4-(((S)-3,3-difluoro-1-(p-tolyl)propyl)-sulfonyl)-2,3,5,6-tetrafluorobenzoate (119)
This compound was obtained in 70% (78.8 mg) yield as yellow oil by the general procedure.
^{1}H NMR (600 MHz, $CDCl_3$) δ 7.20 (d, J = 8.0 Hz, 2H), 7.15 (d, J = 7.9 Hz, 2H), 5.76 (tdd, J = 55.9, 6.0, 3.5 Hz, 1H), 5.18 (dd, J = 3.6, 1.9 Hz, 1H), 4.57 (dd, J = 10.4, 4.6 Hz, 1H), 3.00 – 2.87 (m, 1H), 2.83 – 2.69 (m, 1H), 2.52 – 2.43 (m, 1H), 2.33 (s, 3H), 1.89 (ddd, J = 13.5, 9.4, 4.3 Hz, 1H), 1.83 – 1.72 (m, 1H), 1.36 (tt, J = 13.2, 2.4 Hz, 1H), 1.30 – 1.24 (m, 2H), 1.13 1.14 (t, J = 3.3 Hz, 1H), 0.94 (s, 3H), 0.92 – 0.89 (m, 6H).
^{13}C NMR (151 MHz, $CDCl_3$) δ 158.44, 144.37 (dm, J = 257.1 Hz), 140.72, 130.17, 129.10, 126.22, 119.12 (t, J = 15.5 Hz), 118.59 (t, J = 16.6 Hz), 114.52 (t, J = 241.5 Hz), 84.26, 66.97 (d, J = 6.5 Hz), 49.11, 48.01, 44.82, 36.64, 32.54 (t, J = 24.1 Hz), 29.70, 27.90, 27.06, 21.21, 19.66, 18.82, 13.35.
^{19}F NMR (565 MHz, $CDCl_3$) δ -116.51 – -116.91 (m), -133.93 – -134.22 (m), -136.33 – -136.58 (m).
HRMS (ESI) (m/z): calcd for $C_{27}H_{28}F_6NaO_4S$ ($[M + Na]^+$): 585.1505; found: 585.1512.

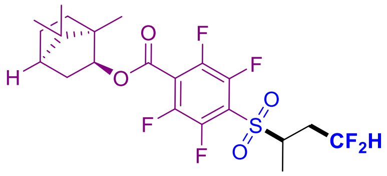

(1R,2S,4R)-1,7,7-trimethylbicyclo[2.2.1]heptan-2-yl 4-(((R)-4,4-difluorobutan-2-yl)sulfonyl)-2,3,5,6-tetrafluorobenzoate (120)
This compound was obtained in 51% (49.6 mg) yield as white solid by the general procedure.
^{1}H NMR (600 MHz, $CDCl_3$) δ 6.11 (tt, J = 55.8, 4.2 Hz, 1H), 5.88 (td, J = 56.0, 2.7 Hz, 0.2H), 5.26 – 5.18 (m, 1.1H), 3.73 – 3.63 (m, 0.10H), 3.65 – 3.56 (m, 1H), 2.68 – 2.55 (m, 1H), 2.54 – 2.44 (m, 1H), 2.27 – 2.11 (m, 1H), 1.98 – 1.89 (m, 1H), 1.87 – 1.76 (m, 2H), 1.52 (d, J = 6.9 Hz, 3H), 1.47– 1.34 (m, 1H), 1.32 (d, J = 7.0 Hz, 0.3H),

1.28 (ddd, J = 11.5, 9.3, 4.3 Hz, 1H), 1.16 (dd, J = 14.0, 3.4 Hz, 1H), 0.96 (s, 3H), 0.92 (d, J = 1.7 Hz, 6H).

^{13}C NMR (151 MHz, $CDCl_3$) δ 158.37, 144.54 (dm, J = 241.8 Hz), 119.10 (t, J = 17.4 Hz), 118.73 (t, J = 14.8 Hz), 117.16 (t, J = 244.5 Hz), 114.49 (t, J = 241.2 Hz), 84.39, 84.35, 57.20 (t, J = 4.6 Hz), 56.72 (t, J = 5.1 Hz), 49.15, 48.04, 44.83, 33.34 (t, J = 22.9 Hz), 33.02 (t, J = 21.0 Hz), 27.91, 27.09, 19.66, 18.83, 13.96, 13.36.

^{19}F NMR (565 MHz, $CDCl_3$) δ -116.14– -116.28 (m), -133.61 – -133.98 (m), -134.98 – -135.21 (m), -135.83 – -136.00 (m), -136.00 – -136.11 (m).

HRMS (ESI) (m/z): calcd for $C_{21}H_{24}F_6NaO_4S$ ($[M + Na]^+$): 509.1192; found: 509.1185.

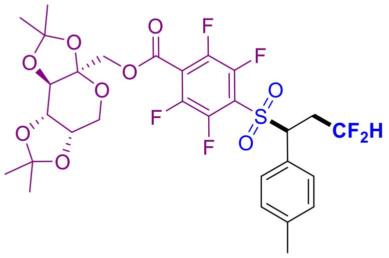

((3aR,5aS,8aS,8bR)-2,2,7,7-tetramethyltetrahydro-3aH-bis([1,3]dioxolo)[4,5-b:4',5'-d]pyran-3a-yl)methyl 4-(((S)-3,3-difluoro-1-(p-tolyl)propyl)sulfonyl)-2,3,5,6-tetrafluorobenzoate (121)

This compound was obtained in 64% (68.2 mg) yield as yellow oil by the general procedure.

^{1}H NMR (600 MHz, $CDCl_3$) δ 7.19 (d, J = 8.2 Hz, 2H), 7.13 (d, J = 7.9 Hz, 2H), 5.76 (tdd, J = 55.9, 6.0, 3.5 Hz, 1H), 4.68 – 4.61 (m, 2H), 4.57 (dd, J = 10.4, 4.6 Hz, 1H), 4.36 – 4.30 (m, 2H), 4.25 (d, J = 7.9 Hz, 1H), 3.93 (dt, J = 13.0, 1.6 Hz, 1H), 3.79 (d, J = 13.0 Hz, 1H), 3.01 – 2.90 (m, 1H), 2.80 – 2.67 (m, 1H), 2.33 (s, 3H), 1.54 (s, 3H), 1.44 (d, J = 6.5 Hz, 3H), 1.34 (d, J = 3.5 Hz, 3H), 1.28 (d, J = 2.0 Hz, 3H).

^{13}C NMR (151 MHz, $CDCl_3$) δ 157.55, 145.28 (t, J = 18.2 Hz), 143.54 (t, J = 19.1 Hz), 140.74, 130.20, 129.02, 126.27, 119.68 (t, J = 14.6 Hz), 117.52 (t, J = 16.3 Hz), 114.48 (t, J = 241.5 Hz), 109.21, 109.14, 100.79, 70.65, 70.57, 69.91, 67.57, 66.96 (d, J = 6.6 Hz), 61.48, 32.47 (t, J = 24.1 Hz), 26.48, 25.79, 24.88, 24.02, 21.21.

^{19}F NMR (565 MHz, $CDCl_3$) δ -116.55 – -116.89 (m), -133.75 – -133.92 (m), -135.08 – -135.27 (m).

HRMS (ESI) (m/z): calcd for $C_{29}H_{30}F_6NaO_9S$ ($[M + Na]^+$): 691.1407; found: 691.1399.

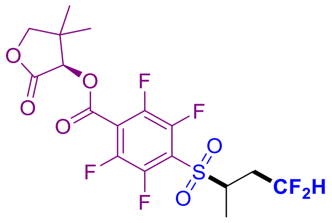

(R)-4,4-dimethyl-2-oxotetrahydrofuran-3-yl 4-(((R)-4,4-difluorobutan-2-yl)sulfonyl)-2,3,5,6-tetra-fluorobenzoate (122)

This compound was obtained in 68% (62.9 mg) yield as white solid by the general procedure.

¹H NMR (600 MHz, $CDCl_3$) δ 6.11 (tt, J = 55.8, 4.1 Hz, 1H), 5.62 (s, 1.12H), 4.13 (d, J = 3.8 Hz, 2H), 4.11 (d, J = 3.5 Hz, 0.14H),3.62 (td, J = 7.6, 5.0 Hz, 1.06H), 2.67 – 2.58 (m, 1.07H), 2.18 (qdd, J = 15.6, 8.4, 4.2 Hz, 1.07H), 1.53 (d, J = 6.9 Hz, 3H), 1.32 (s, 3H), 1.21 (s, 3H).

¹³C NMR (151 MHz, $CDCl_3$) δ 170.52, 157.18, 146.95 – 145.39 (m), 145.19 – 143.35 (m), 119.99 (t, J = 14.9 Hz), 116.76 (t, J = 15.8 Hz), 114.44 (t, J = 241.3 Hz), 77.67, 76.21, 56.83 (t, J = 5.1 Hz), 40.42, 33.32 (t, J = 23.0 Hz), 33.11 (t, J = 20.5 Hz), 22.91, 19.95, 13.94.

¹⁹F NMR (565 MHz, $CDCl_3$) δ -114.16 – -117.89 (m), -133.21 – -133.37 (m), -133.48 – -133.69 (m), -133.75 – -133.93 (m), -134.05 – -134.21 (m).

HRMS (ESI) (m/z): calcd for $C_{17}H_{16}F_6NaO_6S$ ([M + Na]$^+$): 485.0464; found: 485.0453.

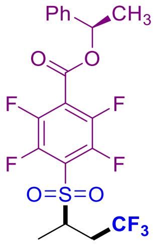

(R)-1-phenylethyl 2,3,5,6-tetrafluoro-4-(((R)-4,4,4-trifluorobutan-2-yl)sulfonyl)benzoate (123)

This compound was obtained in 74% (69.9 mg) yield as white solid by the general procedure.

¹H NMR (600 MHz, $CDCl_3$) δ 7.43 – 7.38 (m, 4H), 7.37 – 7.33 (m, 1H), 6.18 (q, J = 6.7 Hz, 1.1H), 3.70 – 3.65 (m, 0.08H), 3.62 – 3.56 (m, 1H), 3.03 – 2.95 (m, 0.1H), 2.95 – 2.86 (m, 1H), 2.46 – 2.37 (m, 1H), 1.71 (d, J = 6.6 Hz, 3.2H), 1.56 (d, J = 7.0 Hz, 3H), 1.44 (d, J = 6.9 Hz, 0.27H).

¹³C NMR (151 MHz, $CDCl_3$) δ 157.27, 145.32 (dm, J = 264.3 Hz), 139.72, 139.69, 128.76, 128.67(q, J = 280.6 Hz), 126.26, 119.03 (t, J = 16.9 Hz), 118.46 (t, J = 244.3 Hz), 76.61, 76.56, 57.48, 56.74, 34.06 (q, J = 29.0 Hz), 33.11 (q, J = 30.6 Hz), 22.00, 21.97, 13.33, 13.26.

¹⁹F NMR (565 MHz, $CDCl_3$) δ -63.23 (t, J = 10.1 Hz), -73.45 (t, J = 8.7 Hz), -133.58 – -133.75 (m), -134.99 – -135.15 (m), -135.16 – -135.31 (m).

HRMS (ESI) (m/z): calcd for $C_{19}H_{15}F_7NaO_4S$ ([M + Na]$^+$): 495.0471; found: 495.0462.

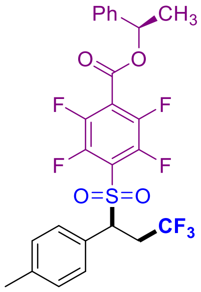

(R)-1-phenylethyl 2,3,5,6-tetrafluoro-4-(((S)-3,3,3-trifluoro-1-(p-tolyl)propyl)sulfonyl)benzoate (124)

This compound was obtained in 85% (94.3 mg) yield as white solid by the general procedure.

^{1}**H NMR** (600 MHz, $CDCl_3$) δ 7.41 – 7.38 (m, 4H), 7.35 (d, J = 6.0, 1H), 7.18 (d, J = 7.8 Hz, 2H), 7.12 (d, J = 7.8 Hz, 2H), 6.14 (q, J = 6.6 Hz, 1H), 4.61 (d, J = 10.8 Hz, 1H), 3.31 – 3.16 (m, 1H), 3.13 – 3.02 (m, 1H), 2.31 (s, 3H), 1.68 (d, J = 6.6 Hz, 3H).
^{13}**C NMR** (151 MHz, $CDCl_3$) δ 157.36, 144.86 (dm, J = 245.2 Hz), 140.75, 139.74, 130.04, 129.03, 128.74, 128.64, 126.25, 125.98, 125.13 (q, J = 277.6 Hz), 118.92 (t, J = 15.7 Hz), 118.39 (t, J = 16.6 Hz), 76.43, 66.84, 32.38 (q, J = 30.5 Hz), 21.97, 21.21.
^{19}**F NMR** (565 MHz, $CDCl_3$) δ -63.19 (t, J = 9.6 Hz), -133.74 – -134.35 (m), -135.93 – -136.60 (m).
HRMS (ESI) (m/z): calcd for $C_{25}H_{19}F_7NaO_4S$ ($[M + Na]^+$): 571.0784; found: 571.0793.

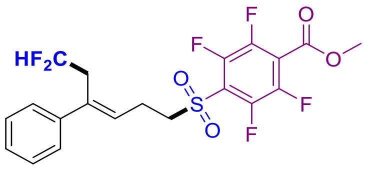

methyl-4-((6,6-difluoro-4-phenylhex-3-en-1-yl)sulfonyl)-2,3,5,6-tetrafluoro-benzoate (127)

This compound was obtained in 32% (29.8 mg) yield as white solid by the general procedure.

^{1}**H NMR** (500 MHz, $CDCl_3$) δ 7.36 – 7.25 (m, 3H), 7.24 (dt, J = 8.0, 2.1 Hz, 2H), 7.03 (d, J = 7.1 Hz, 0.18H), 5.83 – 5.57 (m, 2H), 4.03 (s, 0.28H), 4.00 (s, 2.70H), 3.52 (t, J = 7.5 Hz, 2H), 3.35 – 3.27 (m, 0.2H), 3.08 (td, J = 16.7, 4.7 Hz, 2H), 2.84 (q, J = 7.5 Hz, 2H), 2.45 (q, J = 7.7 Hz, 0.15H).
^{13}**C NMR** (151 MHz, $CDCl_3$) δ 158.59, 145.40 (dd, J = 37.9, 12.2 Hz), 143.68 (dd, J = 34.0, 12.9 Hz), 140.63, 135.29 (t, J = 5.7 Hz), 128.72, 128.04, 127.37, 126.17, 121.36 (t, J = 14.3 Hz), 117.64 (t, J = 16.8 Hz), 115.35 (t, J = 241.4 Hz), 56.90, 53.88, 43.76 (t, J = 22.7 Hz), 35.35 (t, J = 22.8 Hz), 29.71, 22.29.
^{19}**F NMR** (565 MHz, $CDCl_3$) δ -114.81 (dt, J = 56.4, 16.8 Hz), -116.24 (dt, J = 56.1, 15.9 Hz), -134.98 – -135.19 (m), -135.40 – -135.51 (m).
HRMS (ESI) (m/z): calcd for $C_{20}H_{16}F_6NaO_4S$ ($[M + Na]^+$), 489.0566; found, 489.0569.

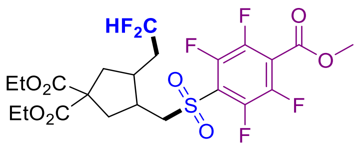

diethyl 3-(2,2-difluoroethyl)-4-(((2,3,5,6-tetrafluoro-4-(methoxycarbonyl)phenyl)sulfonyl)-methyl)cyclopentane-1,1-dicarboxylate (128)

This compound was obtained in 63% (74.5 mg) yield as white solid by the general procedure.

^{1}**H NMR** (500 MHz, $CDCl_3$) δ 5.86 (tt, J = 56.2, 4.2 Hz, 1H), 4.26 – 4.13 (m, 4H), 4.01 (s, 3H), 3.41 – 3.33 (m, 2H), 2.78 – 2.67 (m, 1H), 2.63 – 2.54 (m, 1H), 2.50 – 2.40 (m, 3H), 2.15 – 2.04 (m, 1H), 1.93 – 1.74 (m, 2H), 1.24 (td, J = 7.1, 2.8 Hz, 6H).
^{13}**C NMR** (151 MHz, $CDCl_3$) δ 172.40, 171.62, 158.68, 145.64 (dm, J = 264.4 Hz), 121.37 (t, J = 14.4 Hz), 118.00 – 117.70 (m), 116.32, 114.73, 62.13, 61.93, 58.03, 56.95, 53.93, 38.33, 38.08, 36.88 (t, J = 4.6 Hz), 36.56, 33.35 (t, J = 21.1 Hz), 13.96 (d, J = 4.4 Hz).

^{19}F NMR (565 MHz, $CDCl_3$) δ -114.50 – -116.49 (m), -134.94 – -135.17 (m), -135.48 – -135.67 (m).
HRMS (ESI) (m/z): calcd for $C_{22}H_{24}F_6NaO_8S$ ([M + Na]$^+$), 585.0998; found, 585.0992.

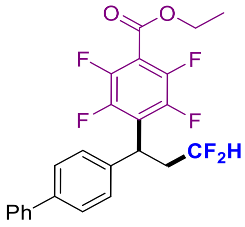

ethyl (S)-4-(1-([1,1'-biphenyl]-4-yl)-3,3-difluoropropyl)-2,3,5,6-tetrafluorobenzoate (129)
This compound was obtained in 58% (52.4 mg) yield as yellow oil by the general procedure.
^{1}H NMR (600 MHz, $CDCl_3$) δ 7.58 – 7.52 (m, 4H), 7.46 – 7.38 (m, 4H), 7.37 – 7.33 (m, 1H), 5.81 (tt, J = 56.1, 4.5 Hz, 1H), 4.75 (t, J = 8.0 Hz, 1H), 4.43 (q, J = 7.1 Hz, 2H), 2.88 – 2.80 (m, 2H), 1.38 (t, J = 7.1 Hz, 3H).
^{13}C NMR (151 MHz, $CDCl_3$) δ 159.50, 145.57, 143.85, 140.89, 140.28, 137.94, 128.84, 127.87, 127.84, 127.56, 127.06, 124.41 (t, J = 15.6 Hz), 115.70 (t, J = 240.2 Hz), 112.03 – 111.63 (m), 62.73, 36.63 (t, J = 21.7 Hz), 35.15, 14.07.
^{19}F NMR (565 MHz, $CDCl_3$) δ -115.34 – -118.49 (m), -137.53 – -139.84 (m), -140.28 – -141.62 (m).
HRMS (ESI) (m/z): calcd for $C_{24}H_{18}F_6NaO_2$ ([M + Na]$^+$), 475.1104; found, 475.1104.

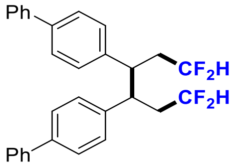

4,4''-((3R,4S)-1,1,6,6-tetrafluorohexane-3,4-diyl)di-1,1'-biphenyl (129a)
This compound was obtained in 11% (10.2 mg) yield as yellow oil by the general procedure.
^{1}H NMR (600 MHz, $CDCl_3$) δ 7.58 – 7.52 (m, 4H), 7.45 (d, J = 8.2 Hz, 4H), 7.42 (t, J = 7.7 Hz, 4H), 7.34 – 7.31 (m, 2H), 6.99 (d, J = 8.2 Hz, 4H), 5.61 – 5.39 (m, 2H), 3.19 (d, J = 10.6 Hz, 2H), 2.37 (dd, J = 23.2, 12.2 Hz, 2H), 2.21 (ddt, J = 31.2, 16.3, 9.0 Hz, 2H).
^{13}C NMR (151 MHz, $CDCl_3$) δ 140.38, 140.01, 137.83, 129.27, 128.77, 127.36, 126.91, 126.90, 116.48 (t, J = 239.2 Hz), 44.98 (d, J = 7.9 Hz), 37.97 (t, J = 21.4 Hz).
^{19}F NMR (565 MHz, $CDCl_3$) δ -114.71 (dddd, J = 284.1, 56.2, 11.9, 7.9 Hz), -117.94 (dddd, J = 284.1, 57.2, 31.1, 13.8 Hz).
HRMS (ESI) (m/z): calcd for $C_{30}H_{26}F_4Na$ ([M + Na]$^+$), 485.1863; found, 485.1872.

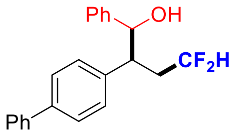

(1S,2S)-2-([1,1'-biphenyl]-4-yl)-4,4-difluoro-1-phenylbutan-1-ol (130)

This compound was obtained in 17% (14.5 mg) yield as yellow oil by the general procedure.

^{1}H NMR (600 MHz, $CDCl_3$) δ 7.59 (td, J = 7.3, 6.6, 1.7 Hz, 3.8H), 7.56 – 7.54 (m, 2H), 7.49 (d, J = 8.2 Hz, 2H), 7.43 (dt, J = 15.0, 7.7 Hz, 4.1H), 7.37 – 7.25 (m, 10H), 7.24 – 7.17 (m, 5H), 5.72 – 5.52 (m, 1H), 5.51 – 5.33 (m, 1H), 4.85 (dd, J = 6.6, 3.1 Hz, 1H), 4.83 (dd, J = 7.8, 2.4 Hz, 0.8H), 3.22 – 3.14 (m, 1.8H), 2.56 – 2.44 (m, 1H), 2.43 – 2.31 (m, 1H), 2.26 – 2.12 (m, 1H), 2.09 – 2.00 (m, 0.8H), 1.99 (d, J = 3.3 Hz, 1H), 1.93 (d, J = 2.8 Hz, 0.8H).

^{13}C NMR (151 MHz, $CDCl_3$) δ 142.14, 141.51, 140.56, 140.49, 139.98, 139.04, 138.05, 129.06, 128.83, 128.79, 128.59, 128.32, 127.78, 127.57, 127.42, 127.33, 127.25, 127.05, 126.97, 126.83, 126.28, 117.01 (t, J = 238.5 Hz), 116.49 (t, J = 238.3 Hz), 78.13 , 77.98 , 48.07 – 47.87 (m), 47.72 – 47.52 (m), 36.78 (t, J = 21.7 Hz), 34.92 (t, J = 21.7 Hz).

^{19}F NMR (565 MHz, $CDCl_3$) δ -114.88 – -118.02 (m).

HRMS (ESI) (m/z): calcd for $C_{22}H_{21}F_6O$ ($[M + H]^+$): 339.1555; found: 339.1571.

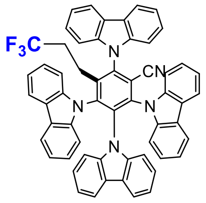

(4s,6s)-2,3,4,6-tetra(9H-carbazol-9-yl)-5-(3,3,3-trifluoropropyl)benzonitrile (132)

This compound was obtained in 19% (16.3 mg) yield as yellow solid by the general procedure.

^{1}H NMR (600 MHz, $CDCl_3$) δ 8.24 (d, J = 7.7 Hz, 2H), 7.70 (dd, J = 7.7, 5.8 Hz, 6H), 7.55 (d, J = 8.0 Hz, 2H), 7.47 (t, J = 7.4 Hz, 2H), 7.29 (d, J = 7.6 Hz, 2H), 7.26 – 7.23 (m, 2H), 7.12 – 7.02 (m, 10H), 6.85 (d, J = 8.1 Hz, 2H), 6.77 (t, J = 7.4 Hz, 2H), 6.61 (t, J = 7.6 Hz, 2H), 2.80 (t, J = 7.5 Hz, 2H), 1.53 – 1.48 (m, 2H).

^{13}C NMR (151 MHz, $CDCl_3$) δ 142.78, 142.39, 141.00, 140.76, 140.36, 138.83, 138.55, 137.77, 136.58, 128.04 (q, J = 269.4 Hz), 127.10, 125.74, 125.52, 124.33, 124.28, 124.15, 123.83, 123.56, 121.72, 121.42, 121.18, 121.08, 120.37, 120.30, 120.23, 119.39, 117.83, 112.32, 109.95, 109.90, 109.67, 109.04, 32.75 (q, J = 29.0 Hz), 22.01.

^{19}F NMR (565 MHz, $CDCl_3$) δ -66.31 (t, J = 10.2 Hz).

HRMS (ESI) (m/z): calcd for $C_{58}H_{36}F_3N_5Na$ ($[M + Na]^+$): 882.2815; found: 882.2811.

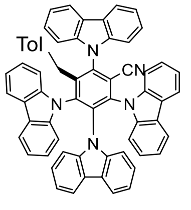

(2r,3r,4r,6r)-2,3,4,6-tetra(9H-carbazol-9-yl)-5-(4-methylbenzyl)benzonitrile (134)

This compound was obtained in 35% (60.0 mg) yield as yellow oil by the general procedure.

^{1}H NMR (600 MHz, $CDCl_3$) δ 8.18 (d, J = 7.6 Hz, 2H), 7.71 – 7.65 (m, 4H), 7.61 (d, J = 7.8 Hz, 2H), 7.46 – 7.37 (m, 4H), 7.28 (d, J = 7.7 Hz, 2H), 7.25 (d, J = 7.6 Hz, 2H), 7.08 – 7.00 (m, 9H), 6.91 (d, J = 8.2 Hz, 2H), 6.75 (t, J = 7.4 Hz, 2H), 6.65 – 6.60 (m,

2H), 6.24 (d, J = 7.7 Hz, 2H), 5.80 (d, J = 7.7 Hz, 2H), 3.71 (s, 2H), 1.83 (s, 3H).
^{13}C NMR (151 MHz, $CDCl_3$) δ 145.75, 141.98, 140.87, 140.12, 138.88, 138.69, 137.85, 136.15, 133.51, 128.39, 127.20, 126.63, 125.43, 125.32, 124.20, 124.08, 123.73, 123.51, 121.15, 121.02, 120.58, 120.16, 120.07, 119.32, 112.60, 110.10, 109.98, 109.85, 109.13, 34.61, 20.49.
HRMS (ESI) (m/z): calcd for $C_{63}H_{41}N_5Na$ ([M + Na]$^+$): 890.3254; found: 890.3241.

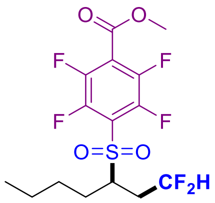

methyl (R)-4-((1,1-difluoroheptan-3-yl)sulfonyl)-2,3,5,6-tetrafluorobenzoate (135)

This compound was obtained in 65% (36.4 mg) yield as yellow oil by the general procedure.
^{1}H NMR (600 MHz, $CDCl_3$) δ 6.15 (tt, J = 56.1, 4.4 Hz, 1H), 4.03 (s, 3H), 3.45 (dt, J = 7.4, 4.1 Hz, 1H), 2.58 – 2.44 (m, 2H), 2.37 – 2.21 (m, 1H), 2.03 – 1.87 (m, 2H), 1.81 – 1.70 (m, 1H), 1.57 – 1.45 (m, 2H), 1.42 – 1.34 (m, 2H), 1.33 – 1.27 (m, 1H), 0.90 (t, J = 7.2 Hz, 4H).
^{13}C NMR (151 MHz, $CDCl_3$) δ 158.61, 145.92 – 145.33 (m), 144.24 – 143.54 (m), 119.83 (t, J = 14.5 Hz), 118.19 (t, J = 16.8 Hz), 114.48 (t, J = 240.5 Hz), 61.12 (t, J = 4.8 Hz), 53.94, 32.18 (t, J = 23.5 Hz), 28.02, 22.21, 13.55.
^{19}F NMR (565 MHz, $CDCl_3$) δ -114.08 – -118.12 (m), -134.03 – -134.36 (m), -135.42 (tt, J = 16.9, 7.6 Hz).
HRMS (ESI) (m/z): calcd for $C_{15}H_{16}F_6NaO_4S$ ([M + Na]$^+$): 429.0566; found: 429.0559.

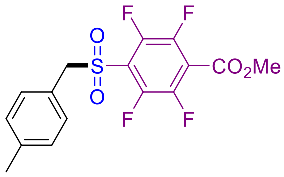

methyl 2,3,5,6-tetrafluoro-4-((4-methylbenzyl)sulfonyl)benzoate (136)

This compound was obtained in 7% (2.3 mg) yield as yellow oil by the general procedure.
^{1}H NMR (600 MHz, $CDCl_3$) δ 7.22 – 7.12 (m, 4H), 4.58 (s, 2H), 4.03 (s, 3H), 2.36 (s, 3H).
^{13}C NMR (151 MHz, $CDCl_3$) δ 158.79, 145.35, 143.61, 139.96, 130.52, 129.91, 122.64, 120.31, 117.56, 63.58, 53.89, 21.25.
^{19}F NMR (565 MHz, $CDCl_3$) δ -134.58 – -134.71 (m), -135.99 – -136.20 (m).
HRMS (ESI) (m/z): calcd for $C_{16}H_{12}F_4NaO_4S$ ([M + Na]$^+$): 399.0285; found: 399.0281.

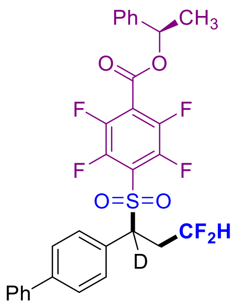

(S)-2,2,2-trifluoro-1-phenylethyl 4-((1-([1,1'-biphenyl]-4-yl)-3,3-difluoropropyl-1-d)sulfonyl)-2,3,5,6-tetra-fluorobenzoate (137)

This compound was obtained in 51% (60.4 mg) yield as yellow oil by the general procedure.

^{1}H NMR (600 MHz, $CDCl_3$) δ 7.59 – 7.55 (m, 2H), 7.54 (d, J = 7.3 Hz, 2H), 7.44 (d, J = 8.4 Hz, 2H), 7.40 – 7.31 (m, 8H), 6.13 (q, J = 6.6 Hz, 1H), 5.82 (tdd, J = 55.8, 5.9, 3.5 Hz, 1H), 4.64 (dd, J = 10.4, 4.6 Hz, 0.03H), 3.04 – 2.93 (m, 1H), 2.89 – 2.75 (m, 1H), 1.66 (d, J = 6.6 Hz, 3H).

^{13}C NMR (151 MHz, $CDCl_3$) δ 157.33, 144.57 (dm, J = 268.7 Hz), 143.33, 139.76, 139.48, 129.70, 128.95, 128.73, 128.61, 128.10, 128.04, 127.10, 126.23, 119.19 (t, J = 14.2 Hz), 118.44 (t, J = 16.8 Hz), 114.44 (t, J = 241.7 Hz), 76.43, 66.99 (t, J = 5.9 Hz), 32.61 (t, J = 23.8 Hz), 22.00.

^{19}F NMR (565 MHz, $CDCl_3$) δ -115.91 – -117.31 (m), -133.88 – -134.08 (m), -135.96 – -136.09 (m).

HRMS (ESI) (m/z): calcd for $C_{30}H_{21}DF_6NaO_4S$ ([M + Na]+), 616.1098; found, 616.1102.

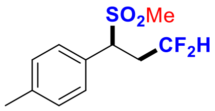

(S)-1-(3,3-difluoro-1-(methylsulfonyl)propyl)-4-methylbenzene (138)

This compound was obtained in 69% (34.2 mg) yield as yellow oil by the general procedure.

^{1}H NMR (600 MHz, $CDCl_3$) δ 7.32 (d, J = 2.0 Hz, 2H), 7.25 (d, J = 7.9 Hz, 2H), 5.76 (tdd, J = 56.1, 5.9, 3.7 Hz, 1H), 4.23 (dd, J = 10.1, 4.9 Hz, 1H), 3.00 – 2.87 (m, 1H), 2.64 (s, 3H), 2.63 – 2.53 (m, 1H), 2.38 (s, 3H).

^{13}C NMR (151 MHz, $CDCl_3$) δ 140.02, 130.23, 128.94, 128.83, 115.03 (t, J = 240.7 Hz), 64.14 (dd, J = 7.1, 4.2 Hz), 38.46, 32.58 (t, J = 23.8 Hz), 21.20.

^{19}F NMR (565 MHz, $CDCl_3$) δ -115.99 – -117.88 (m).

HRMS (ESI) (m/z): calcd for $C_{11}H_{14}F_2NaO_2S$ ([M + Na]$^+$), 271.0575; found, 271.0582.

^1H, ^{13}C, ^{19}F NMR Spectra of New Compounds

^1H NMR (500 MHz, $CDCl_3$) spectrum of 4

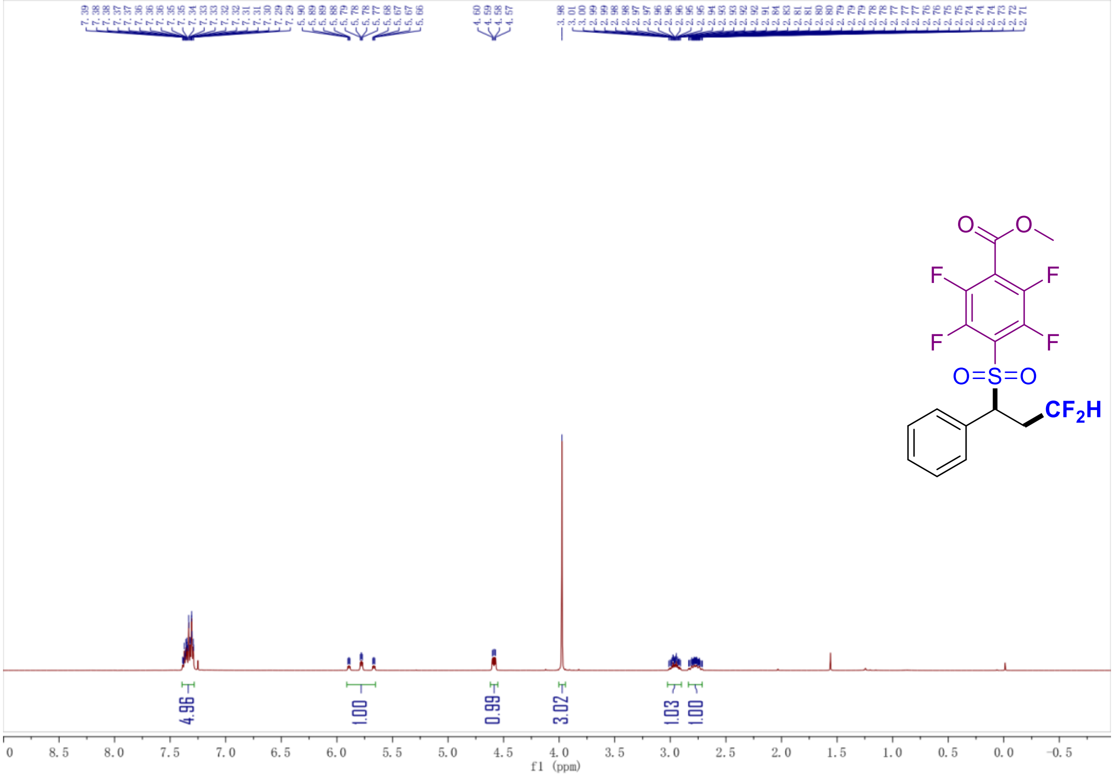

^{13}C NMR (151 MHz, $CDCl_3$) spectrum of 4

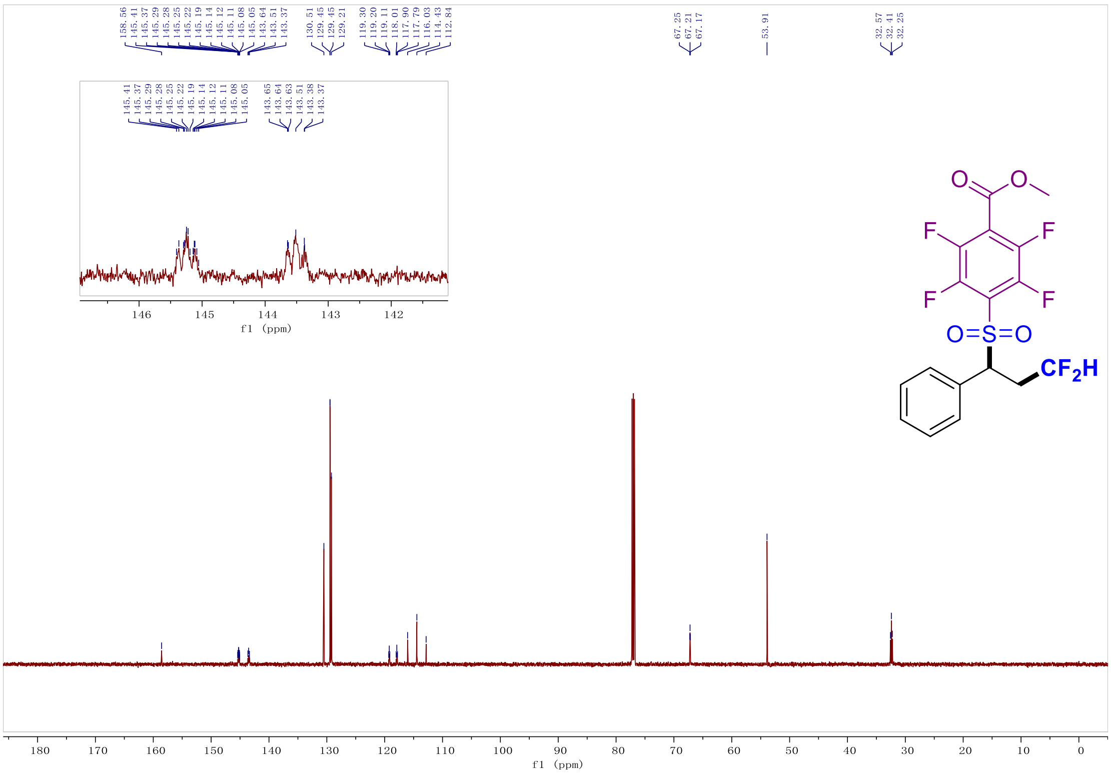

^{19}F NMR (565 MHz, CDCl$_3$) spectrum of 4

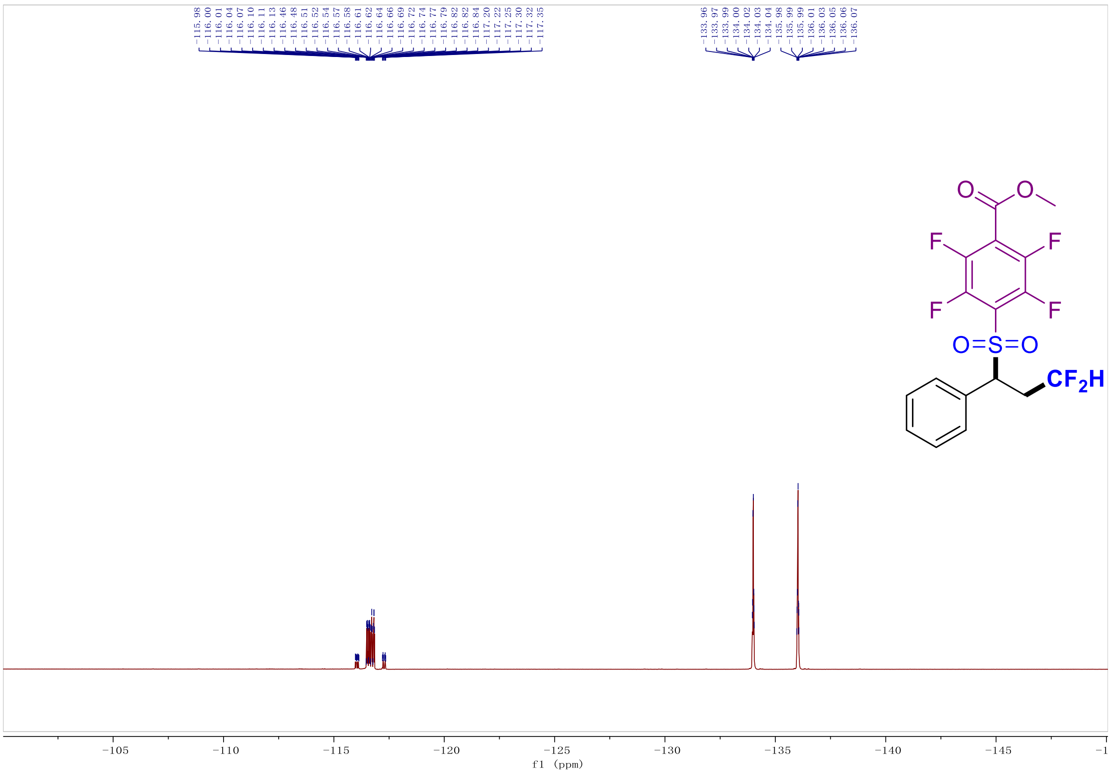

^{1}H NMR (600 MHz, CDCl$_3$) spectrum of 5

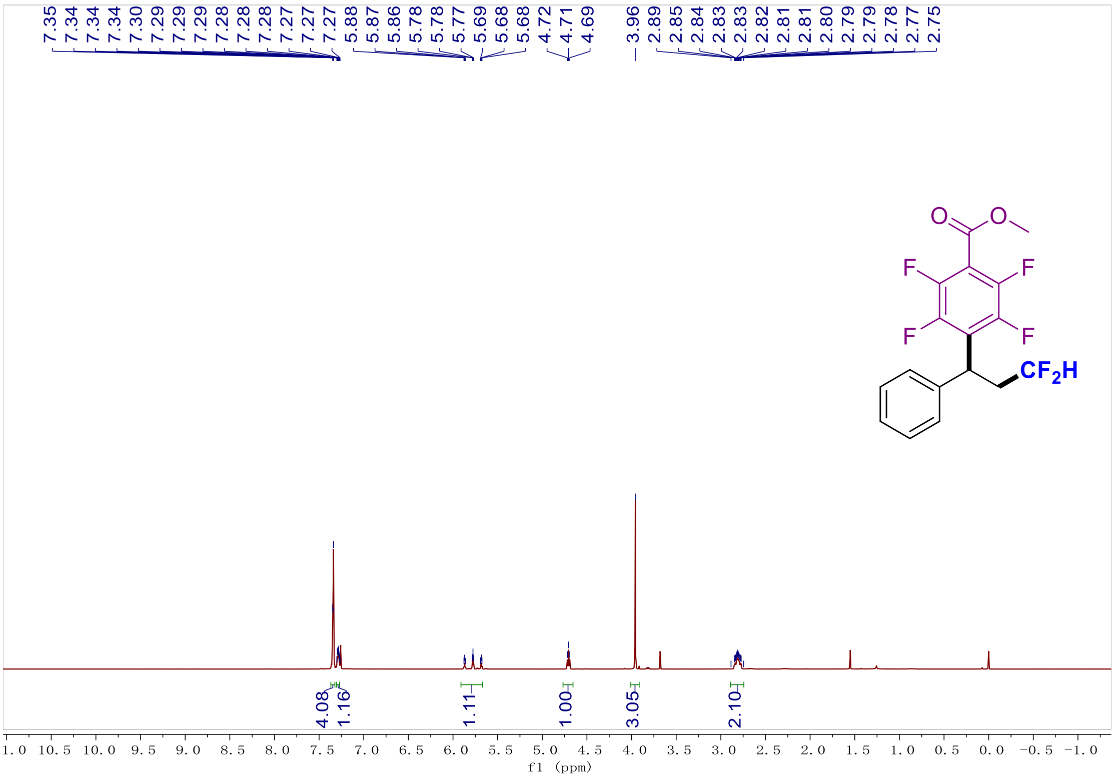

^{13}C NMR (151 MHz, CDCl$_3$) spectrum of 5

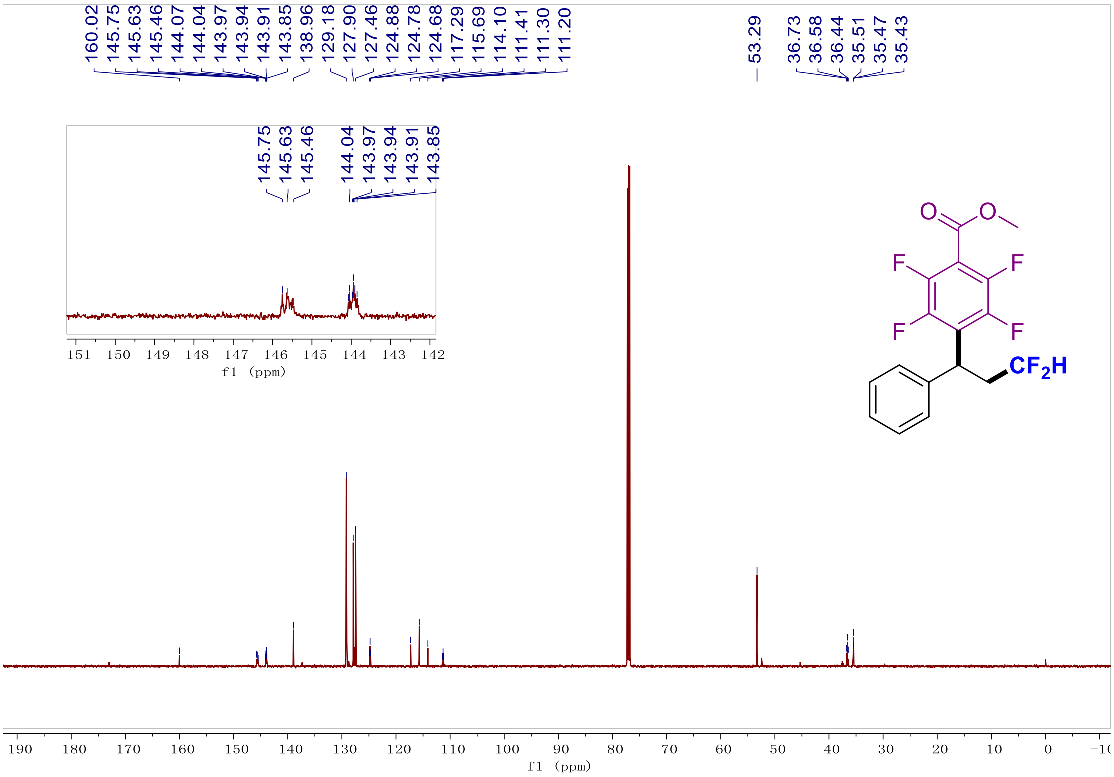

^{19}F NMR (565 MHz, CDCl$_3$) spectrum of 5

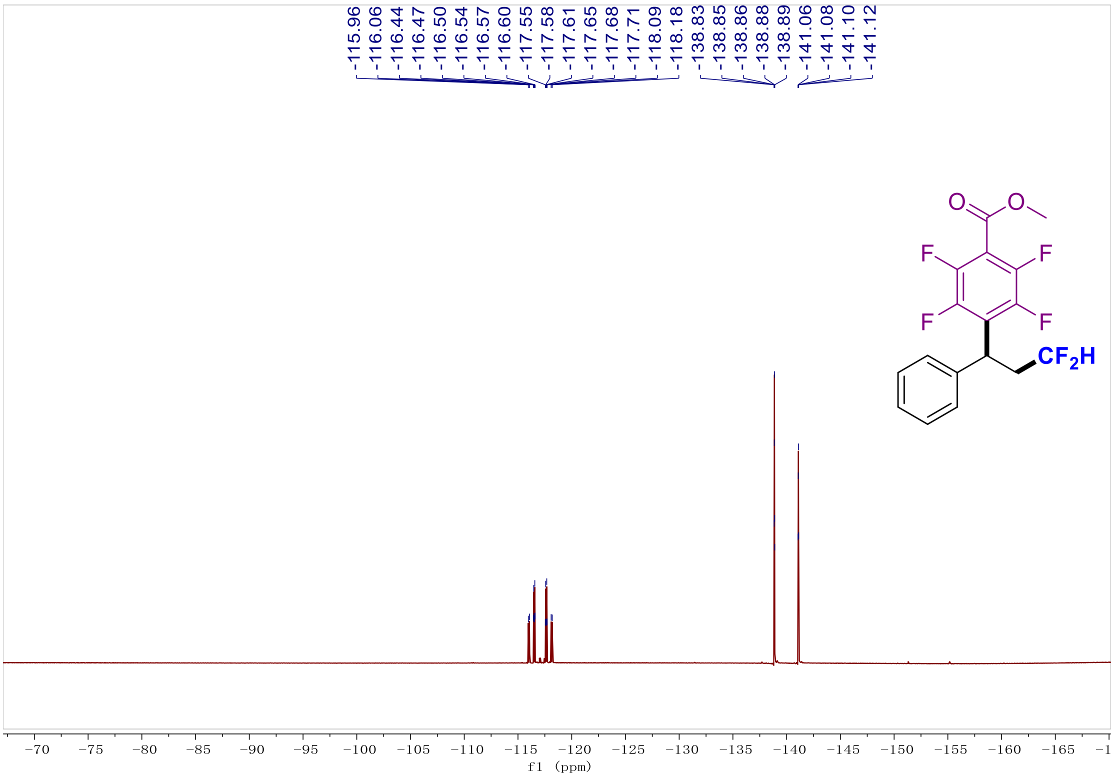

^{1}H NMR (500 MHz, CDCl3) spectrum of 6

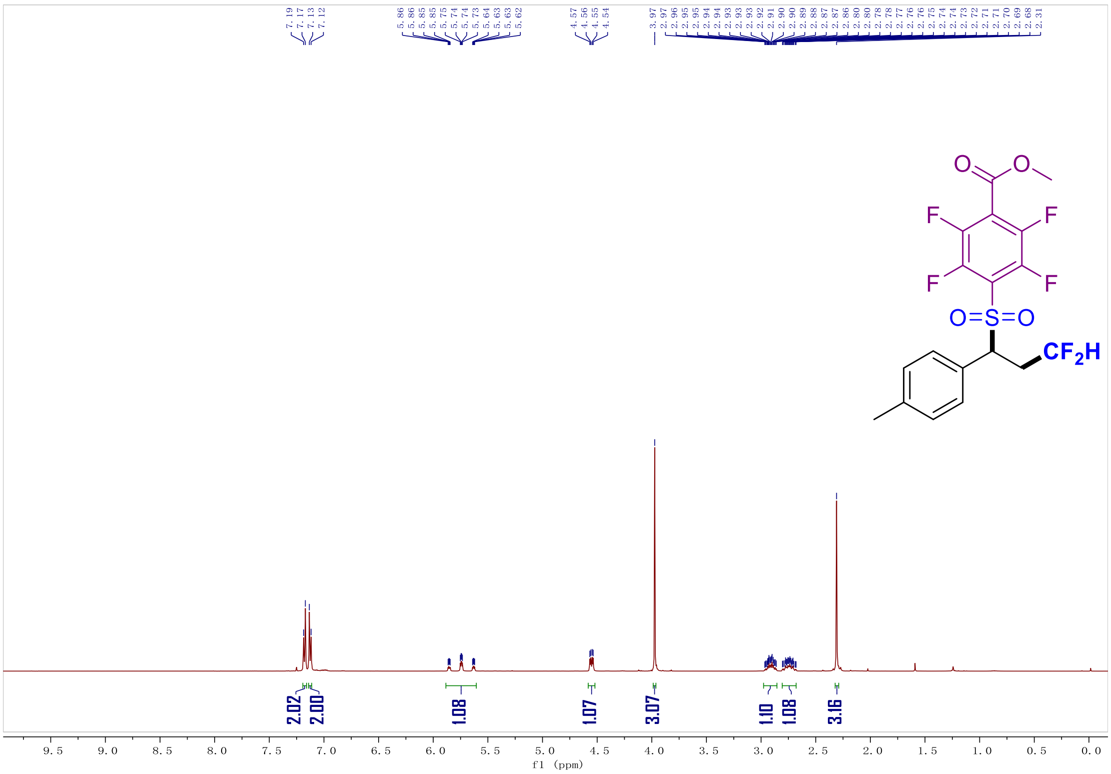

^{13}C NMR (151 MHz, CDCl3) spectrum of 6

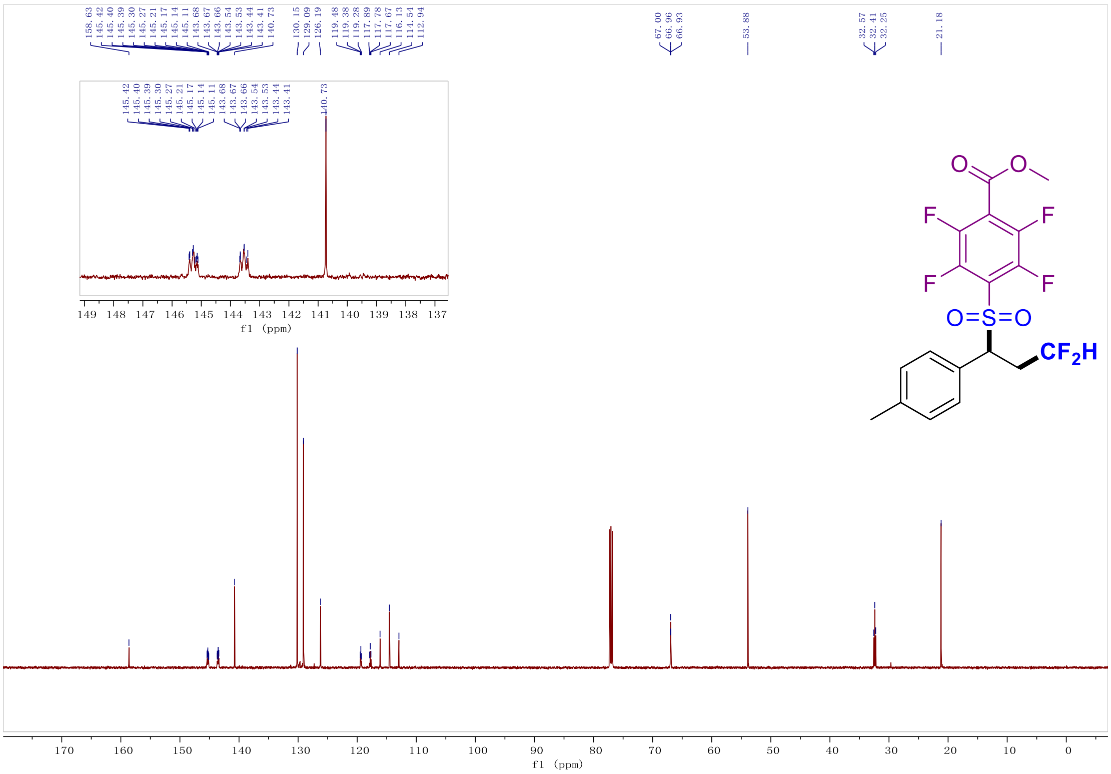

^{19}F NMR (565 MHz, $CDCl_3$) spectrum of 6

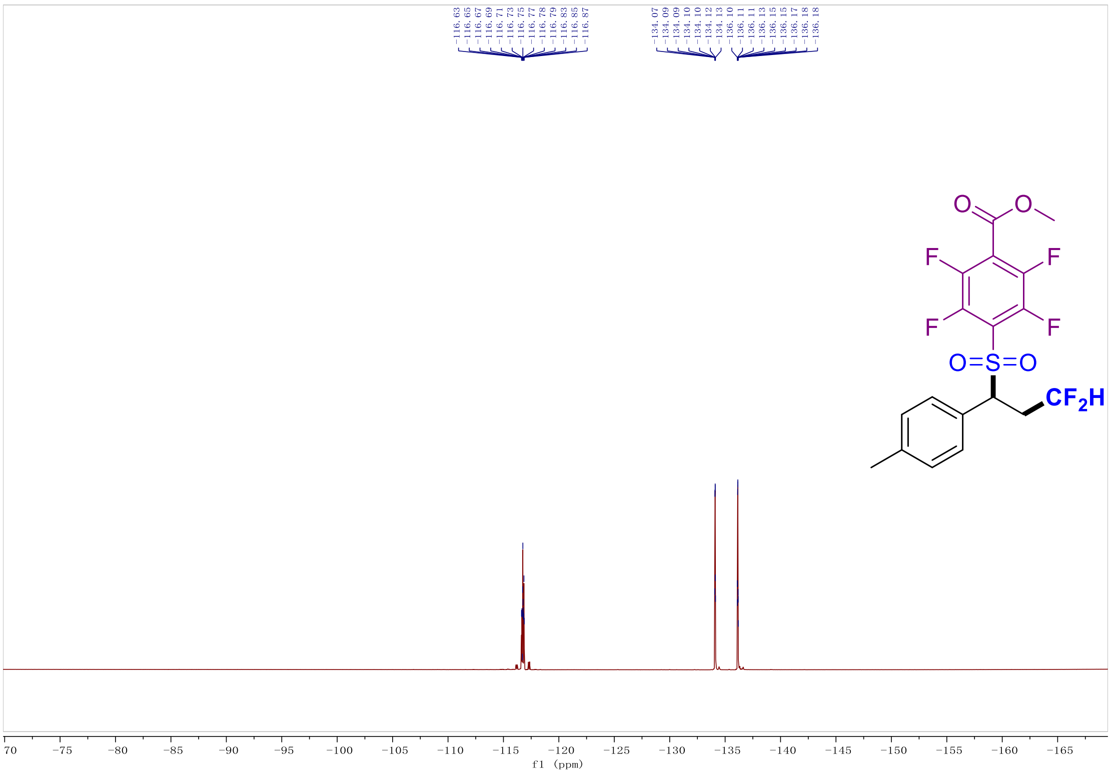

^{1}H NMR (500 MHz, $CDCl_3$) spectrum of 7

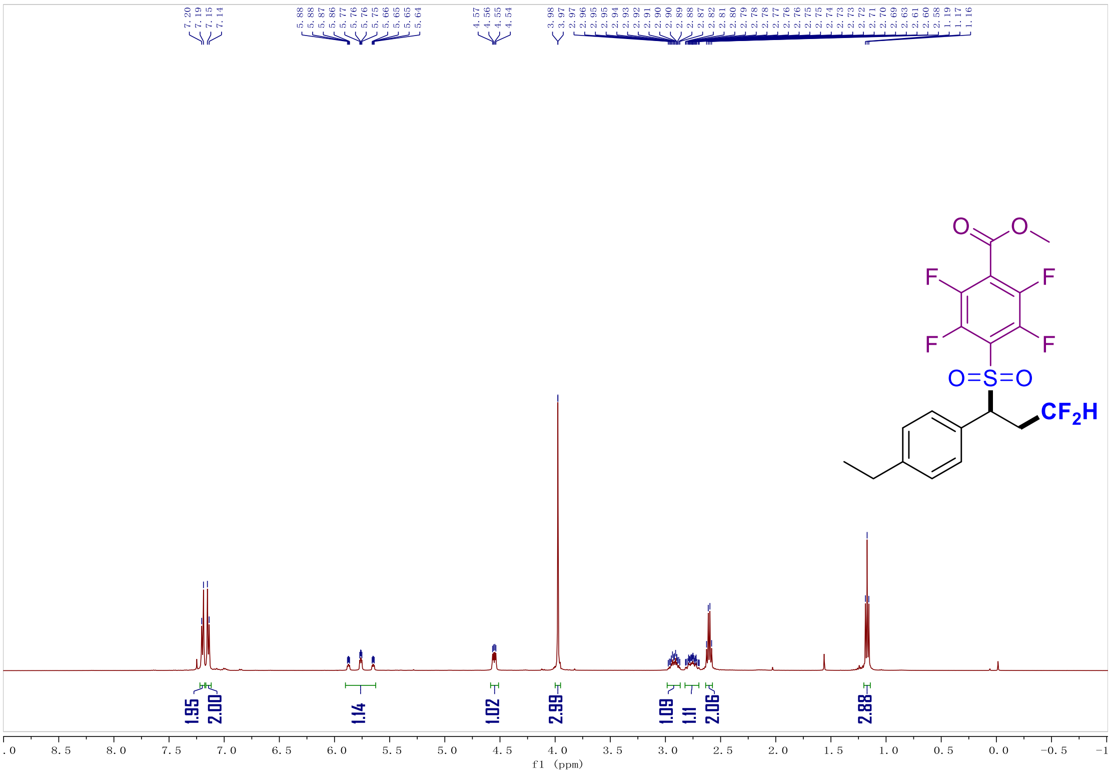

^{13}C NMR (151 MHz, $CDCl_3$) spectrum of 7

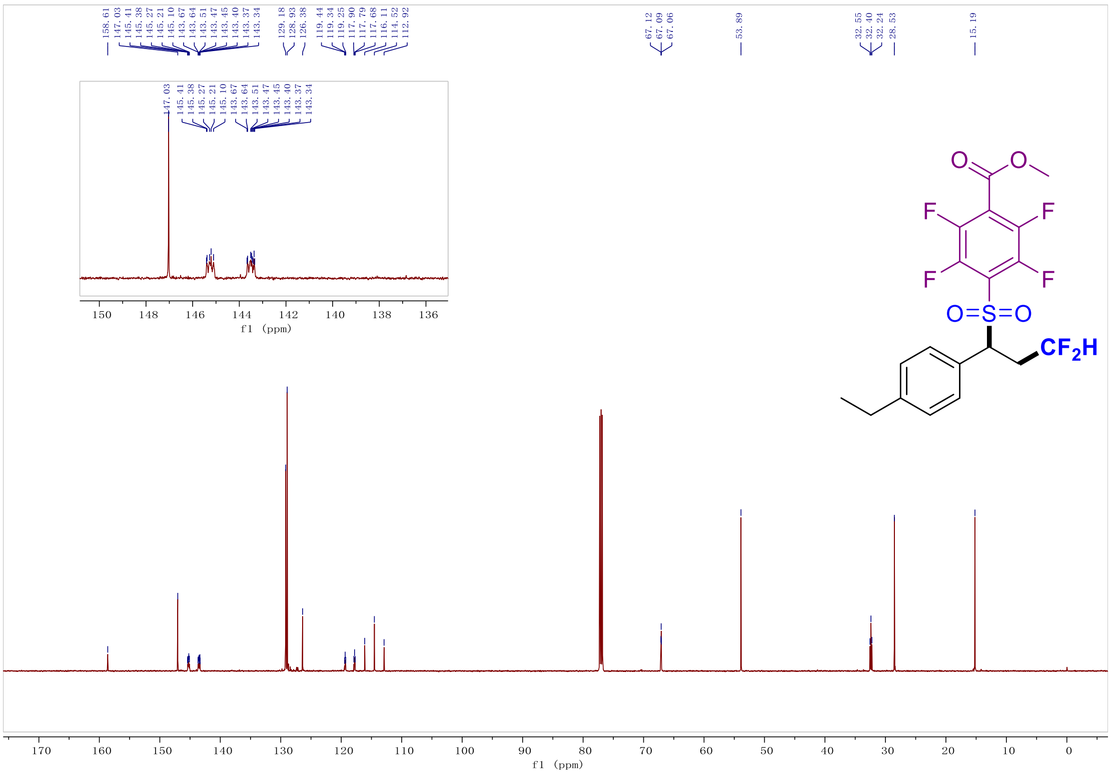

^{19}F NMR (565 MHz, $CDCl_3$) spectrum of 7

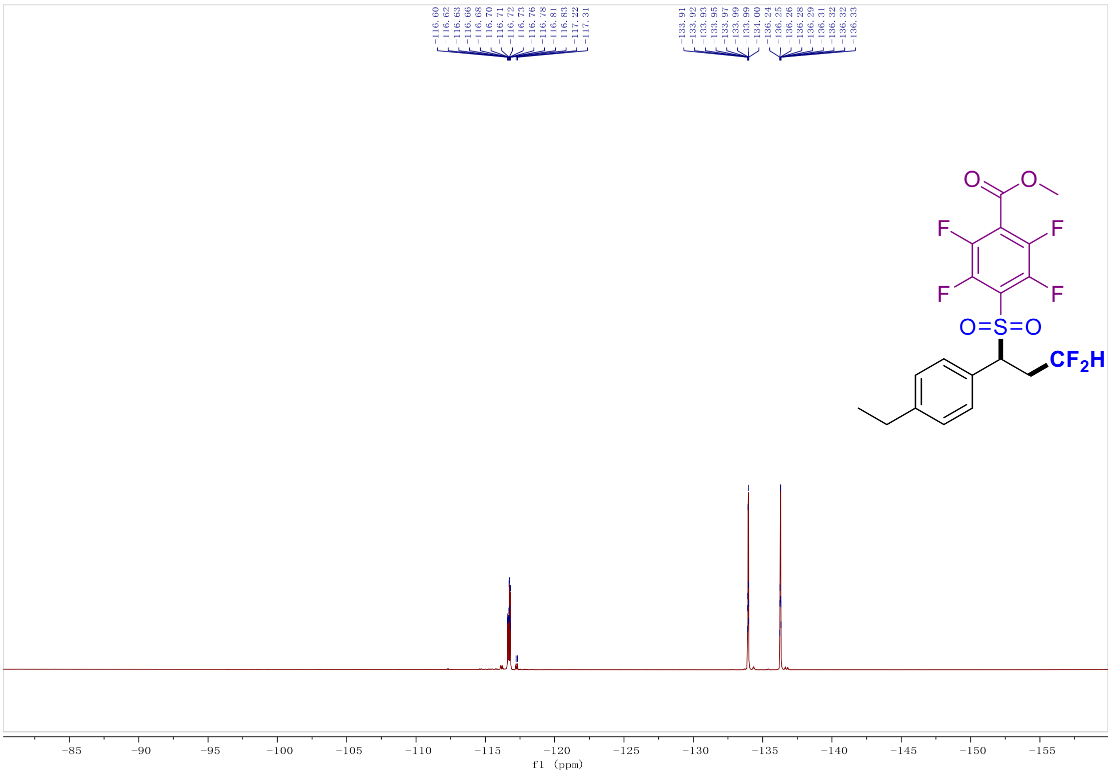

^{1}H NMR (500 MHz, $CDCl_3$) spectrum of 8

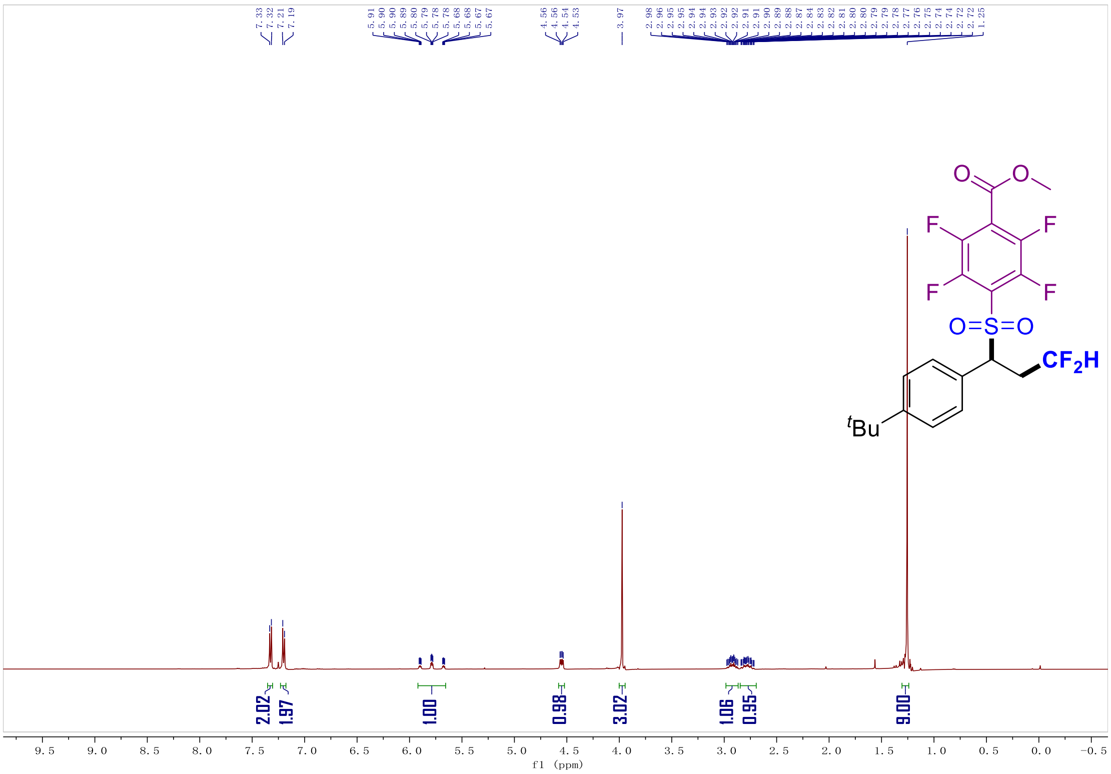

^{13}C NMR (151 MHz, $CDCl_3$) spectrum of 8

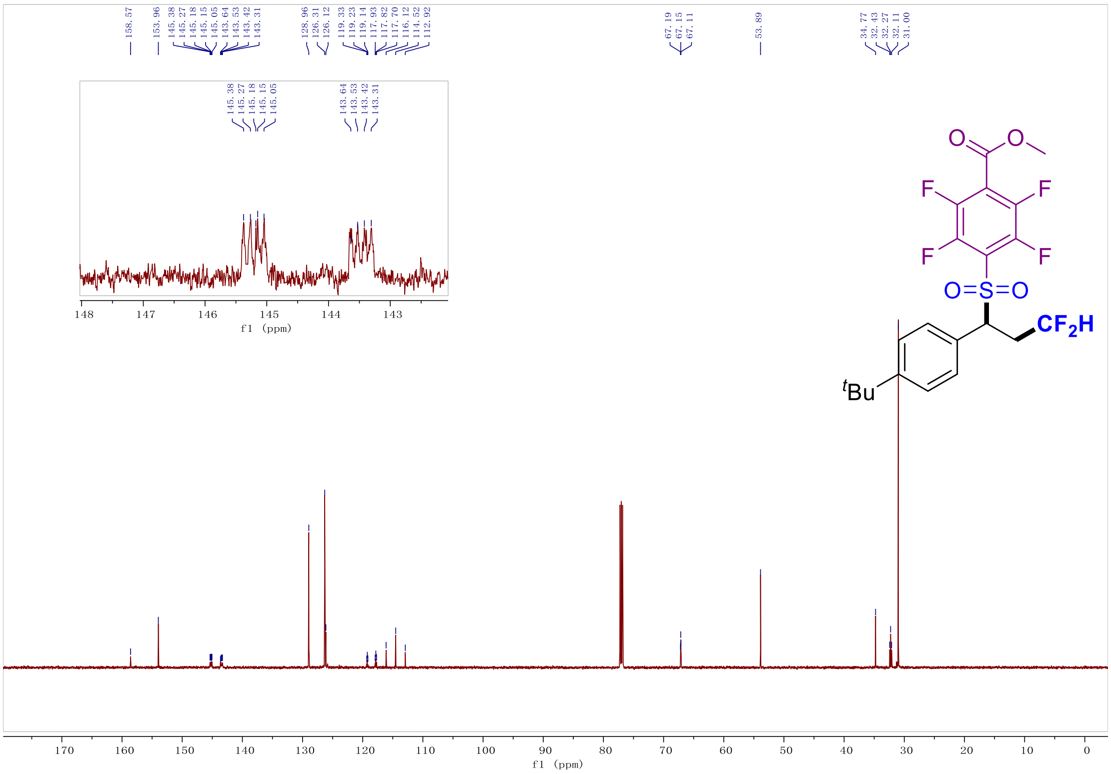

^{19}F NMR (565 MHz, $CDCl_3$) spectrum of 8

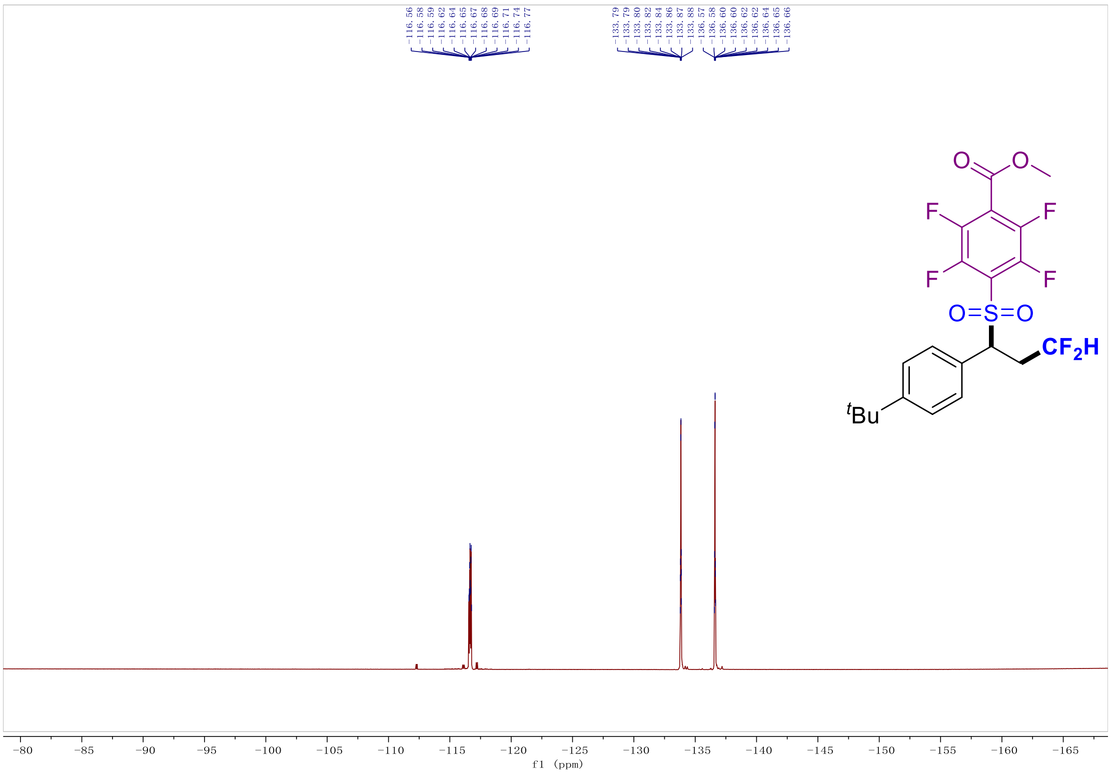

^{1}H NMR (500 MHz, $CDCl_3$) spectrum of 9

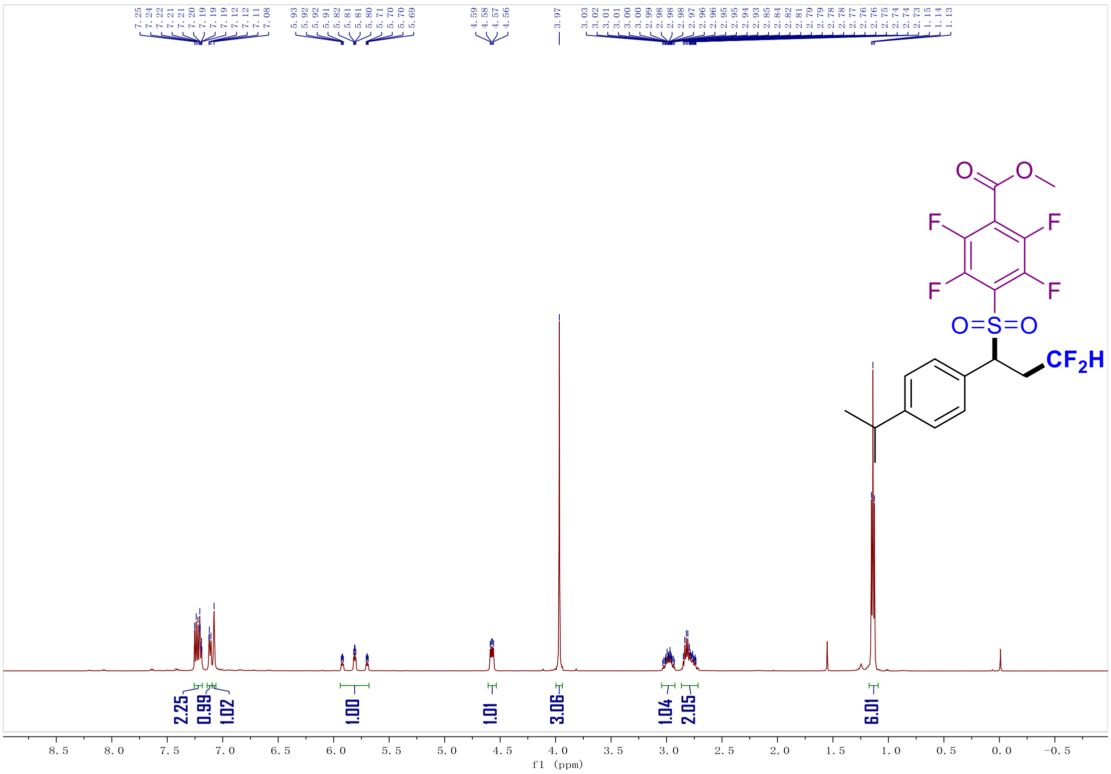

^{13}C NMR (151 MHz, CDCl$_3$) spectrum of 9

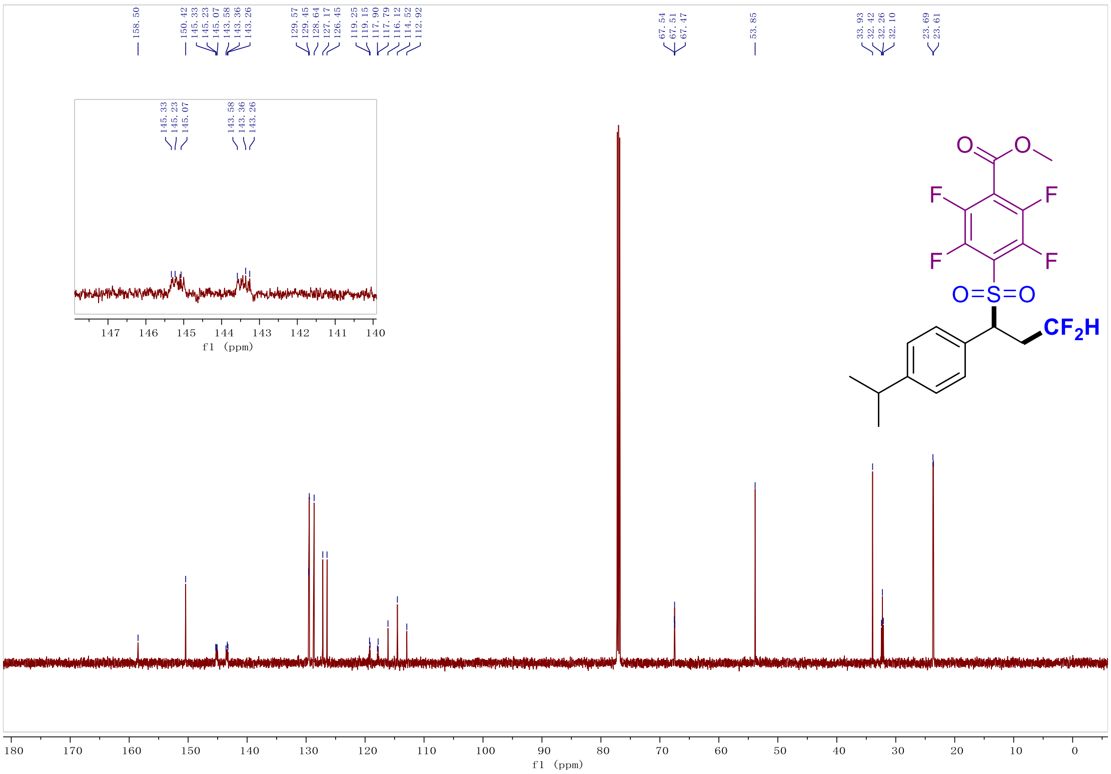

^{19}F NMR (565 MHz, CDCl$_3$) spectrum of 9

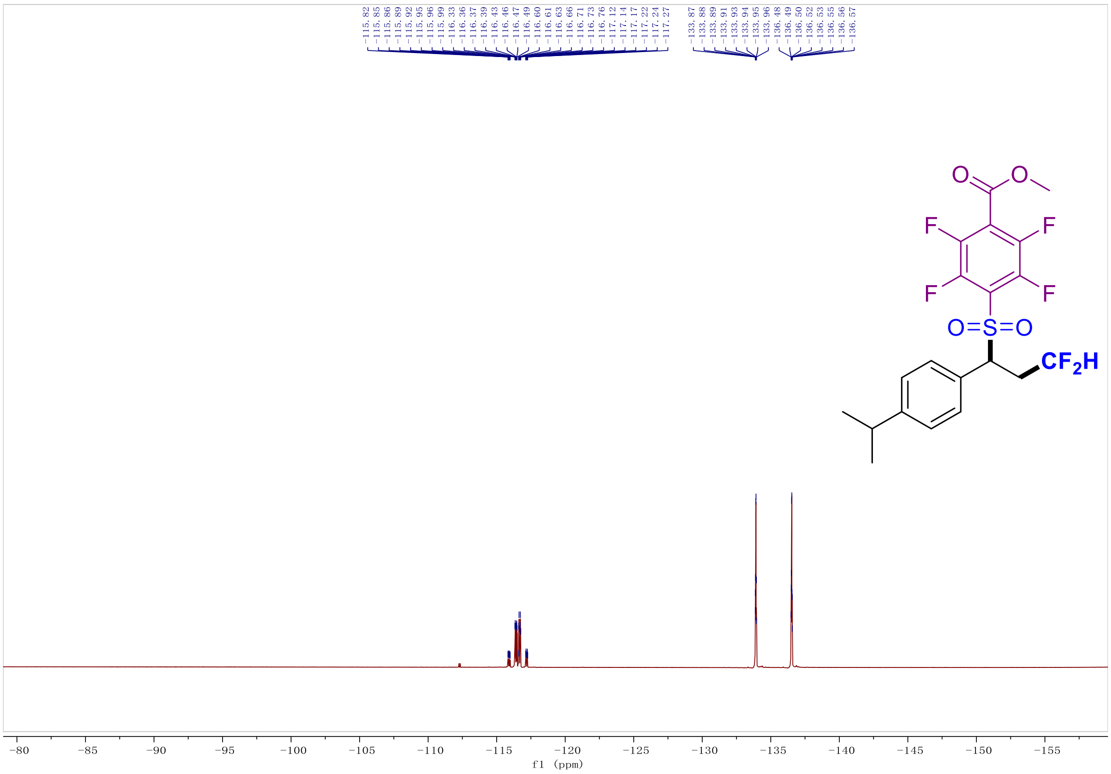

^{1}H NMR (500 MHz, $CDCl_3$) spectrum of 10

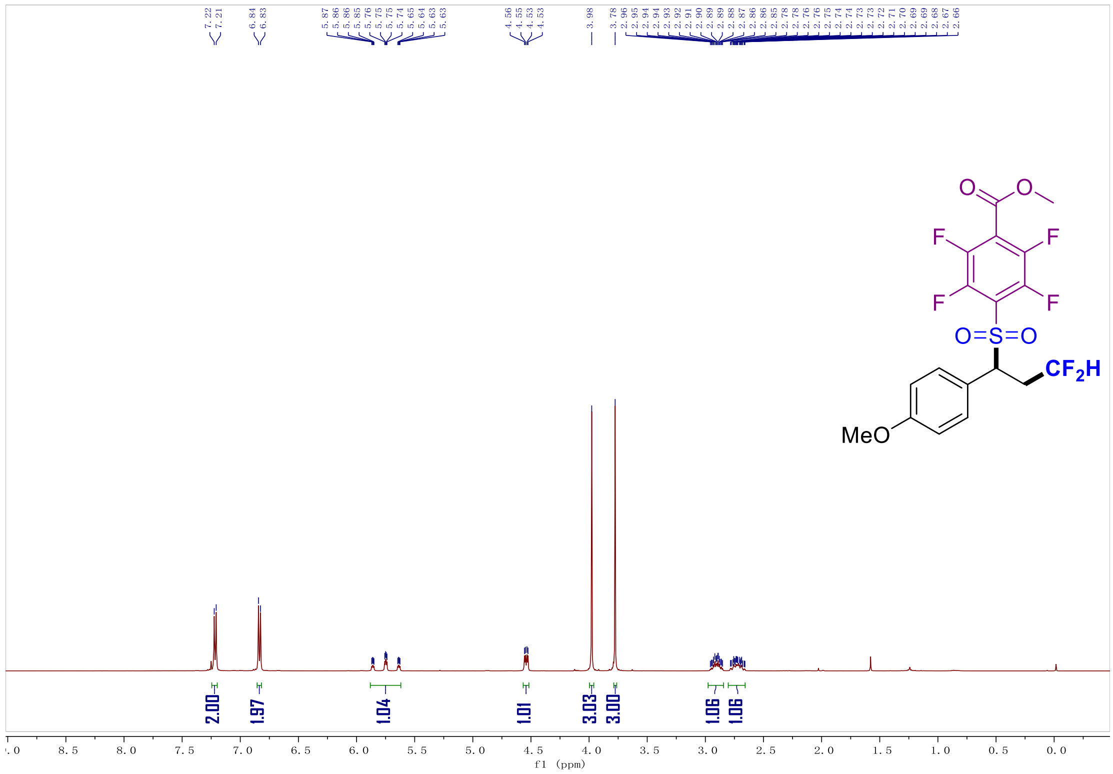

^{13}C NMR (151 MHz, $CDCl_3$) spectrum of 10

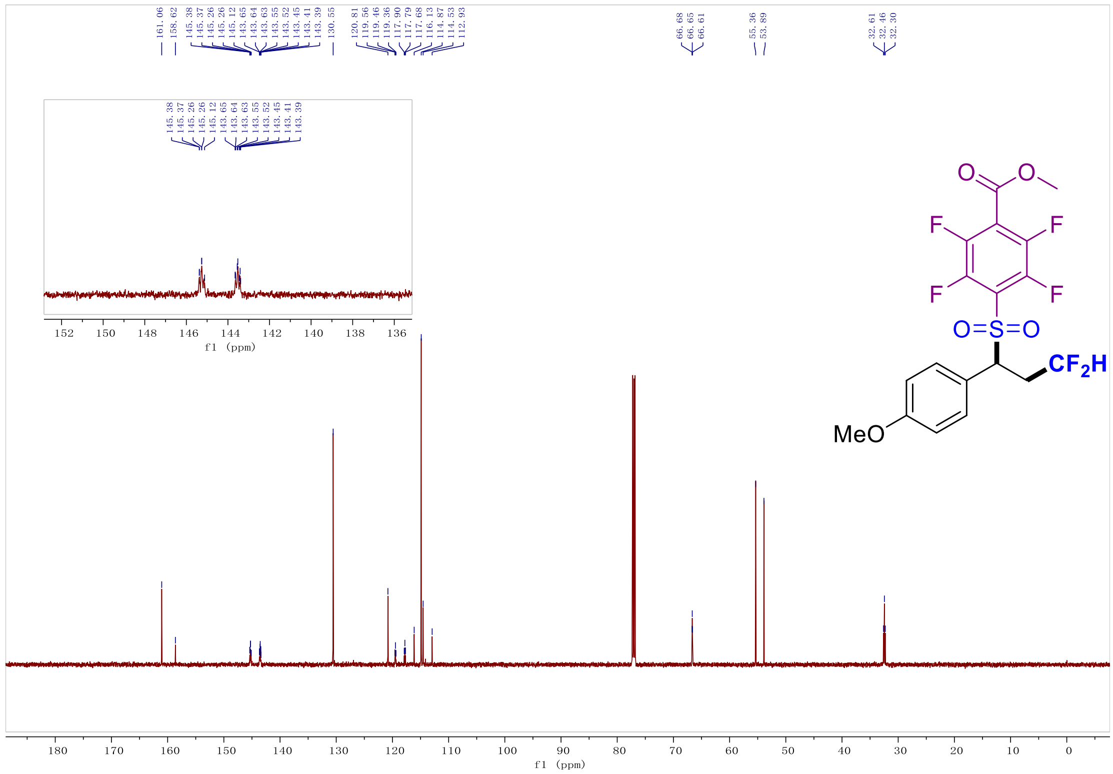

^{19}F NMR (565 MHz, CDCl3) spectrum of 10

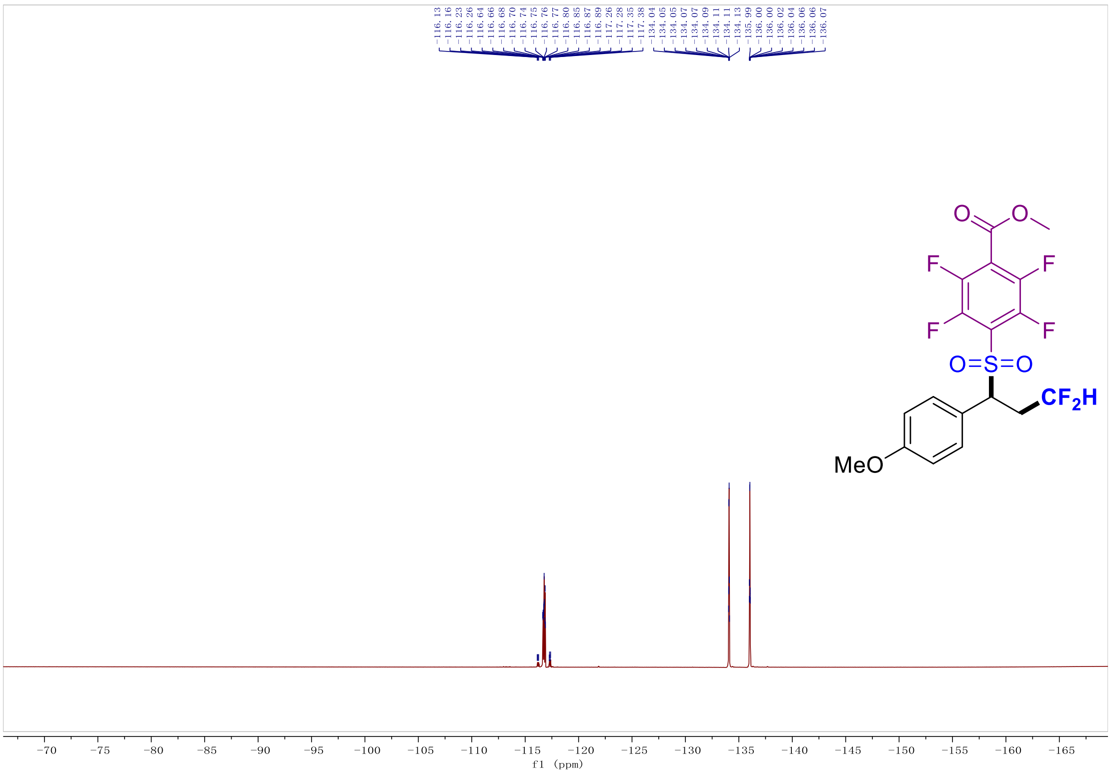

^{1}H NMR (500 MHz, CDCl3) spectrum of 11

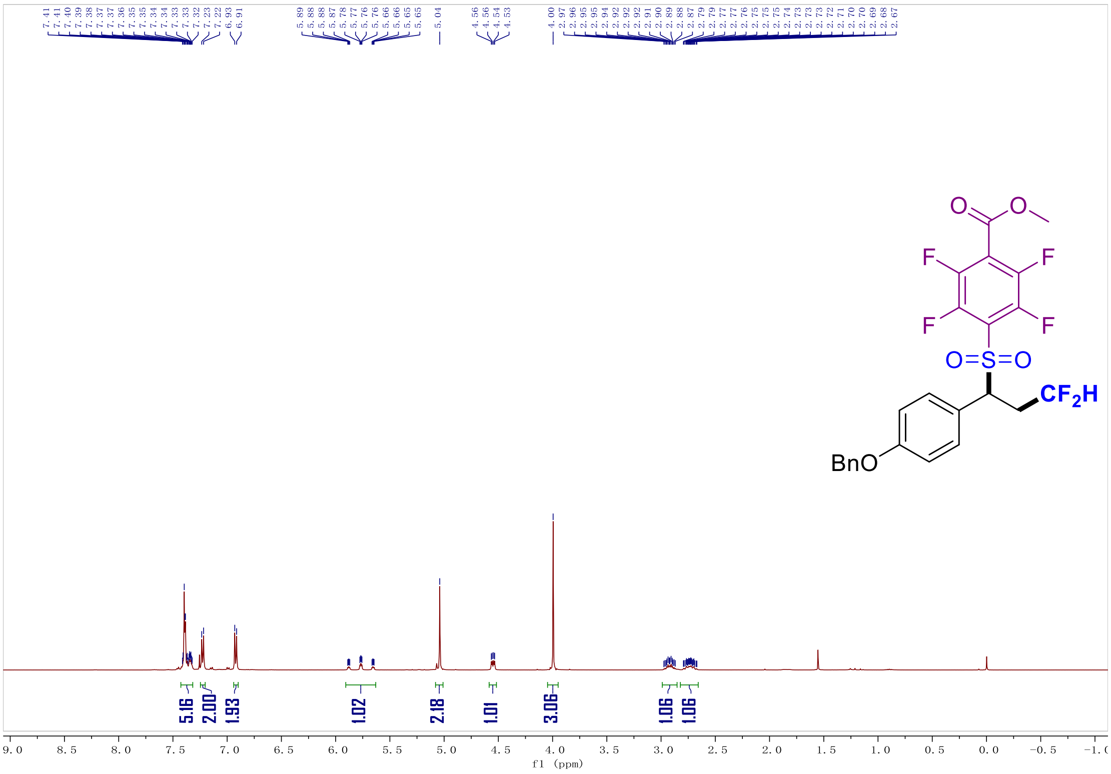

^{13}C NMR (151 MHz, CDCl$_3$) spectrum of 11

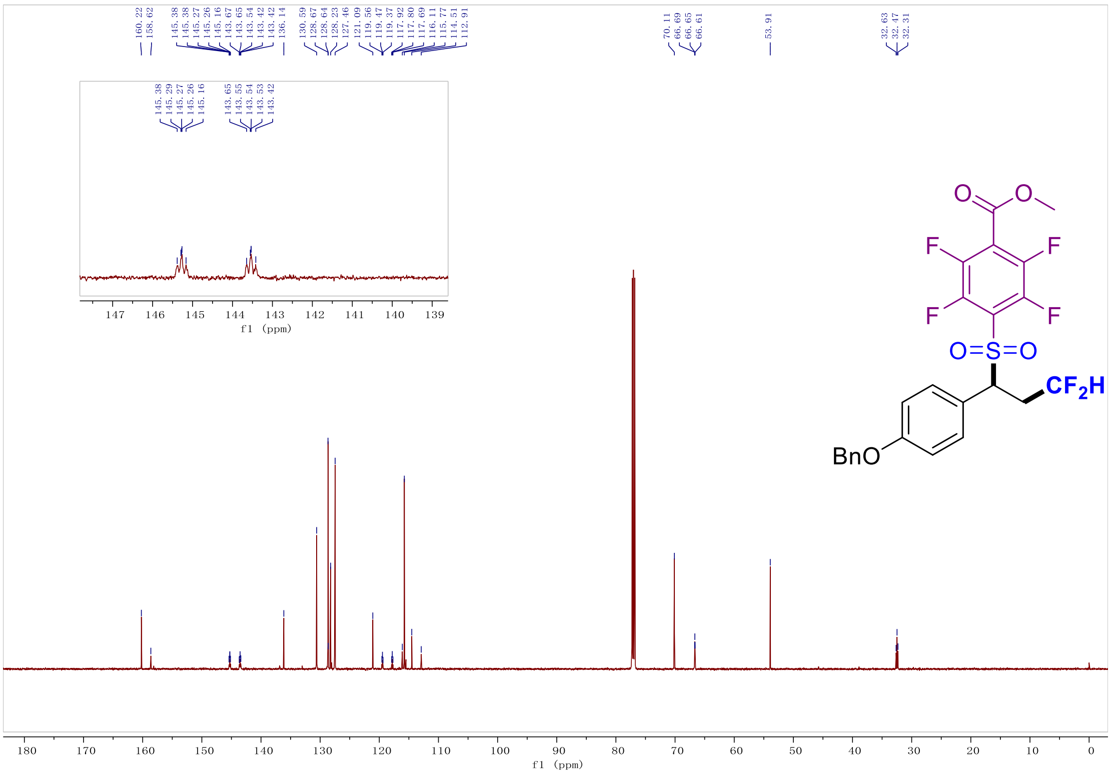

^{19}F NMR (565 MHz, CDCl$_3$) spectrum of 11

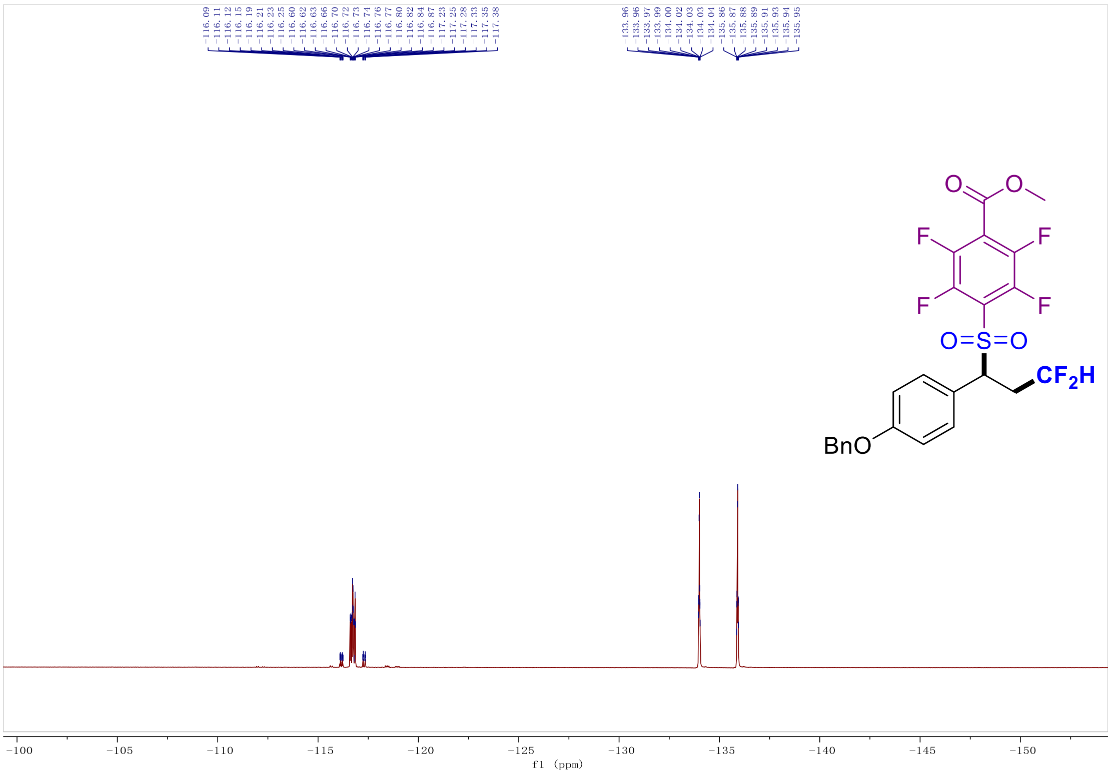

^{1}H NMR (500 MHz, $CDCl_3$) spectrum of 12

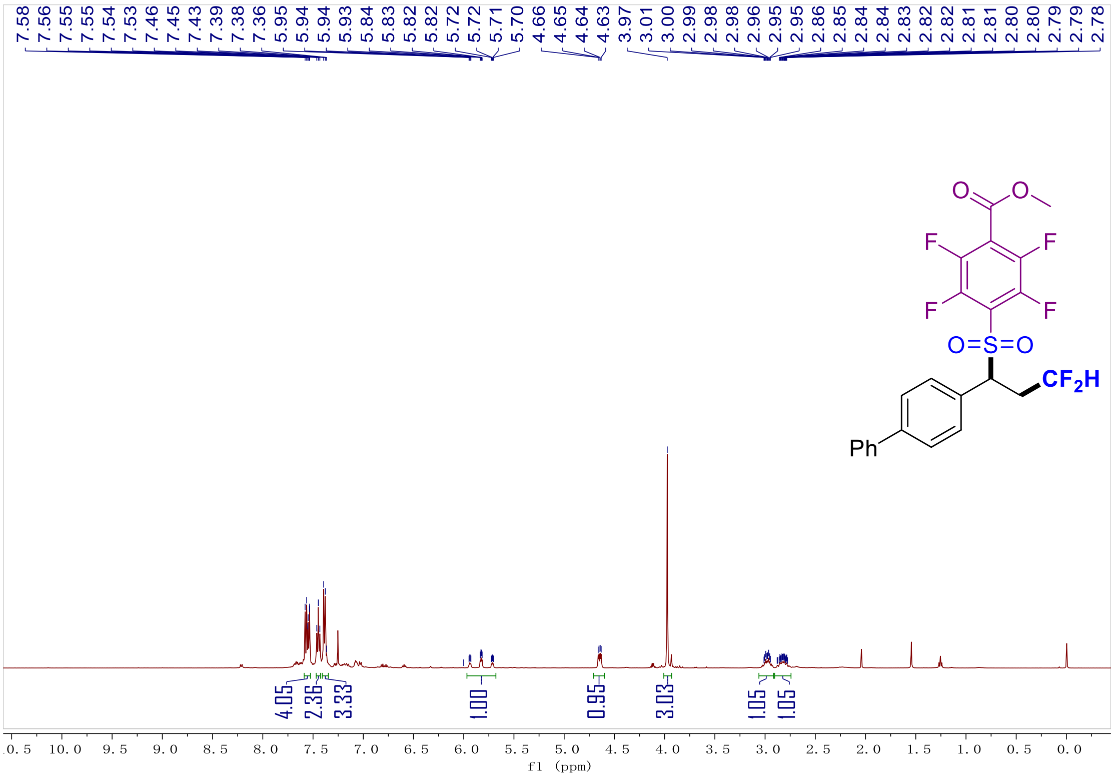

^{13}C NMR (151 MHz, $CDCl_3$) spectrum of 12

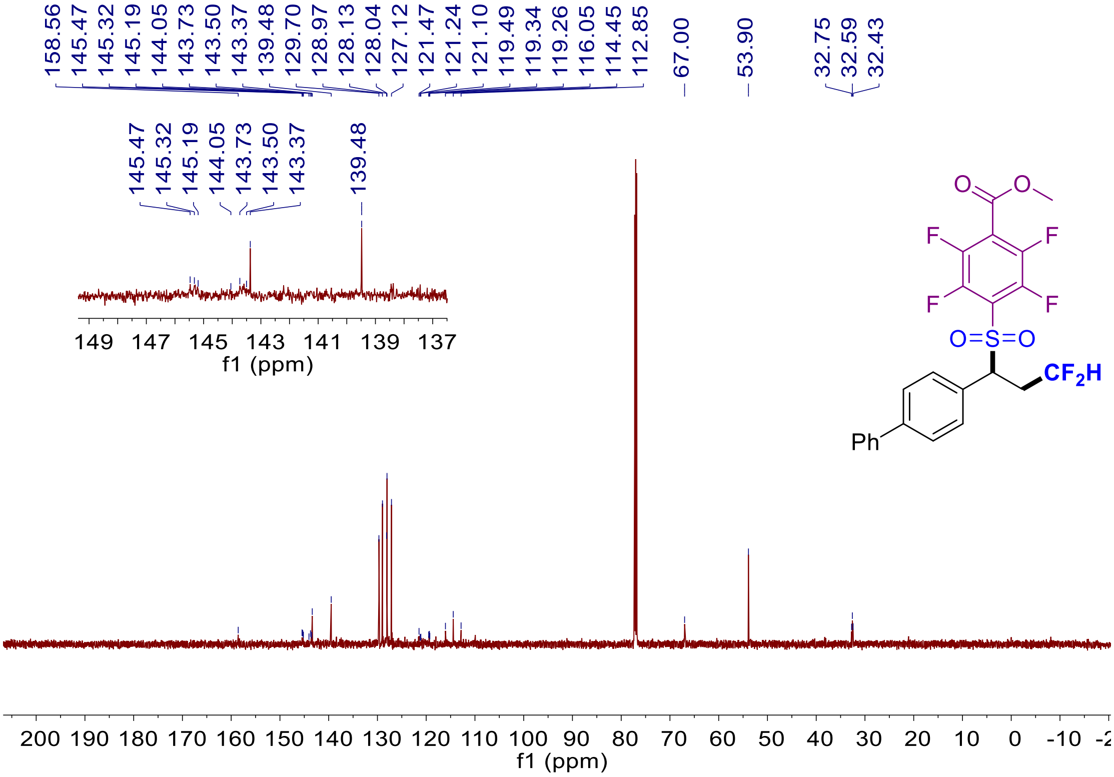

^{19}F NMR (565 MHz, CDCl$_3$) spectrum of 12

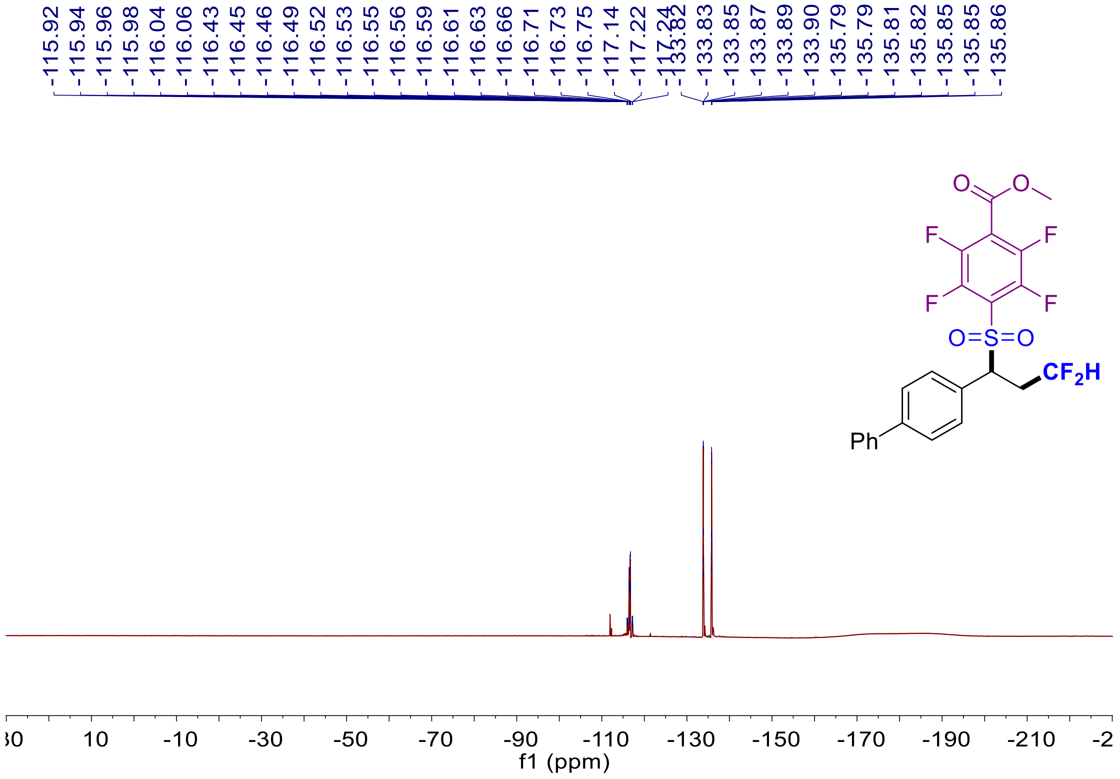

^{1}H NMR (500 MHz, CDCl$_3$) spectrum of 13

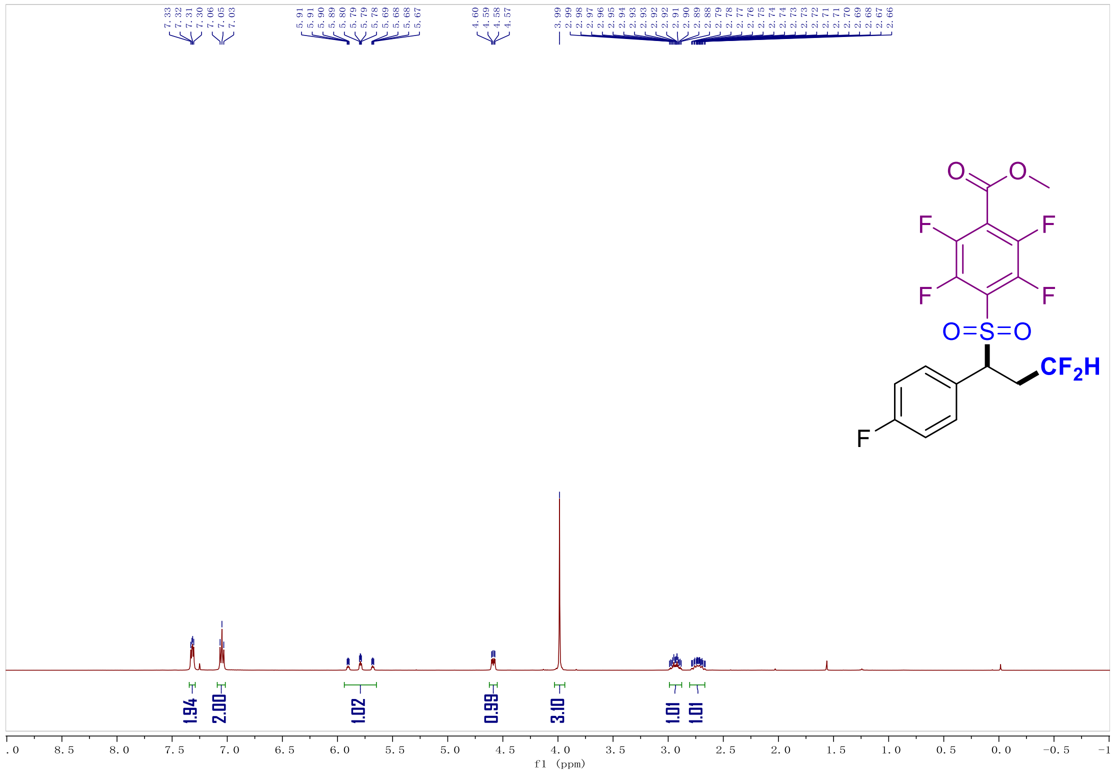

^{13}C NMR (151 MHz, $CDCl_3$) spectrum of 13

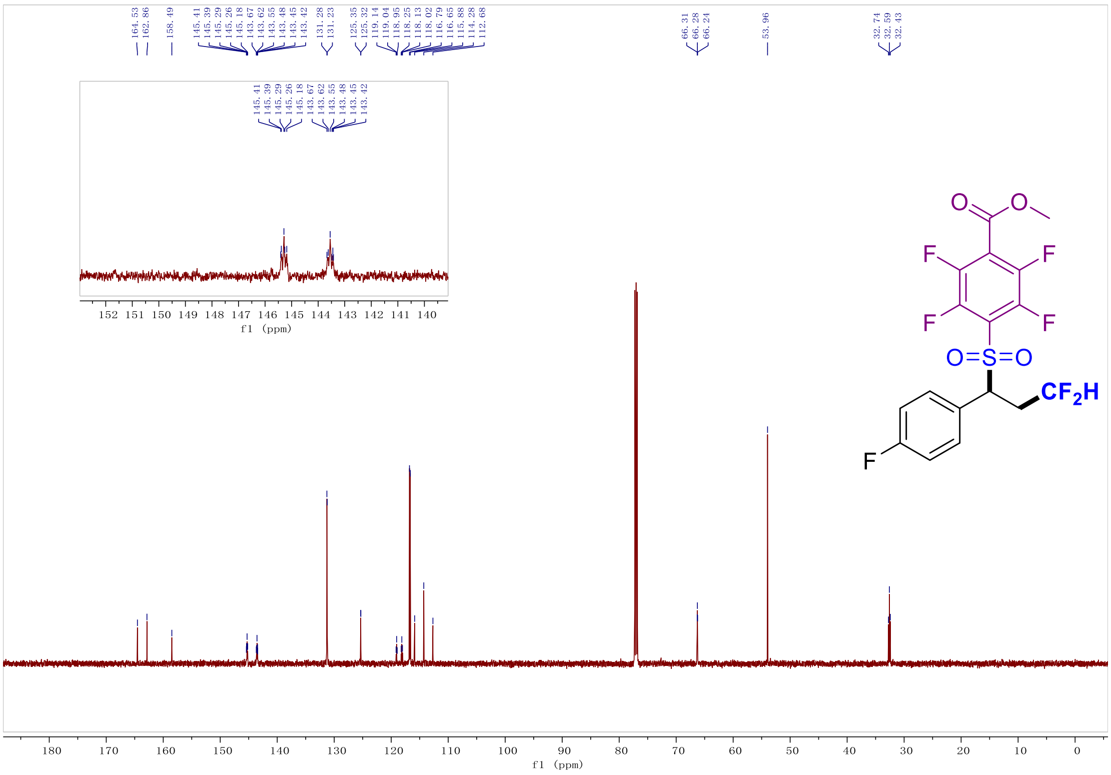

^{19}F NMR (565 MHz, $CDCl_3$) spectrum of 13

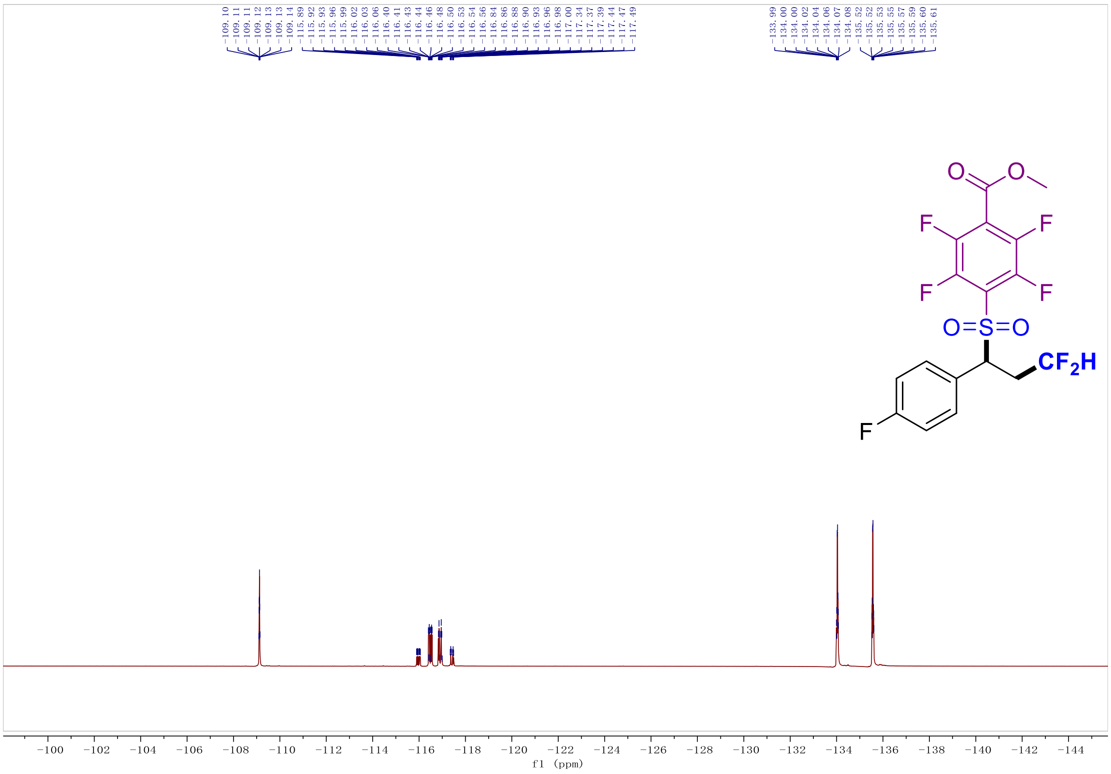

^1H NMR (500 MHz, $CDCl_3$) spectrum of 14

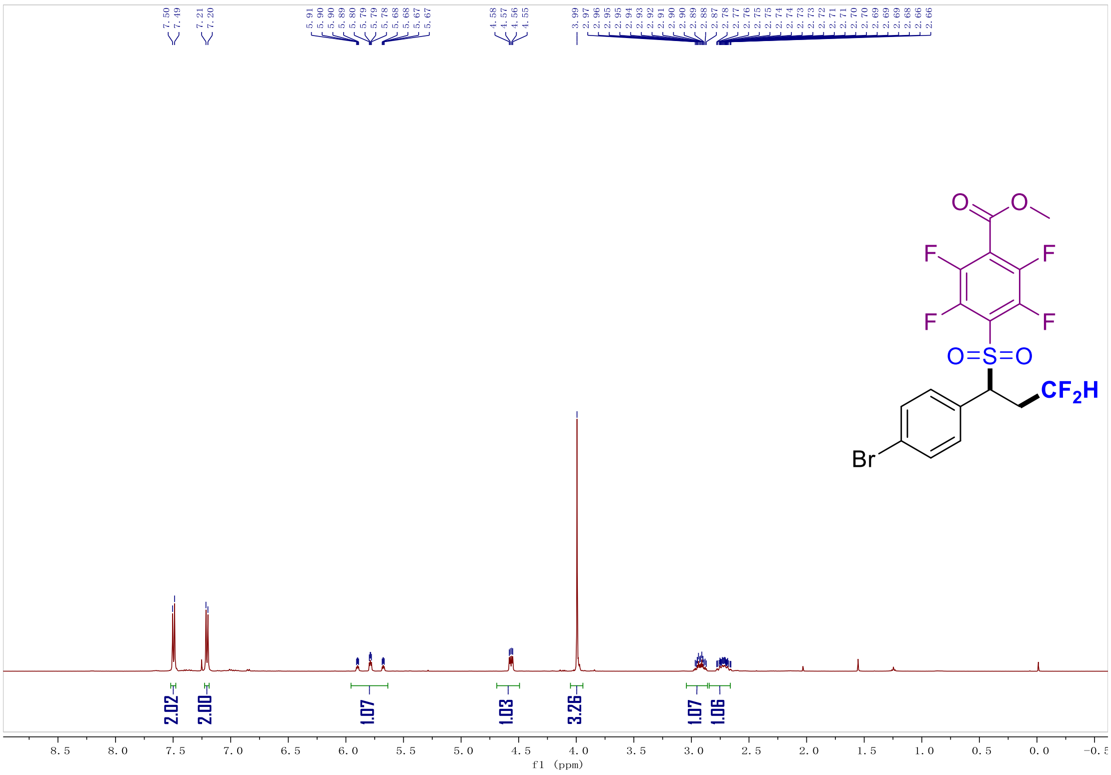

^{13}C NMR (151 MHz, $CDCl_3$) spectrum of 14

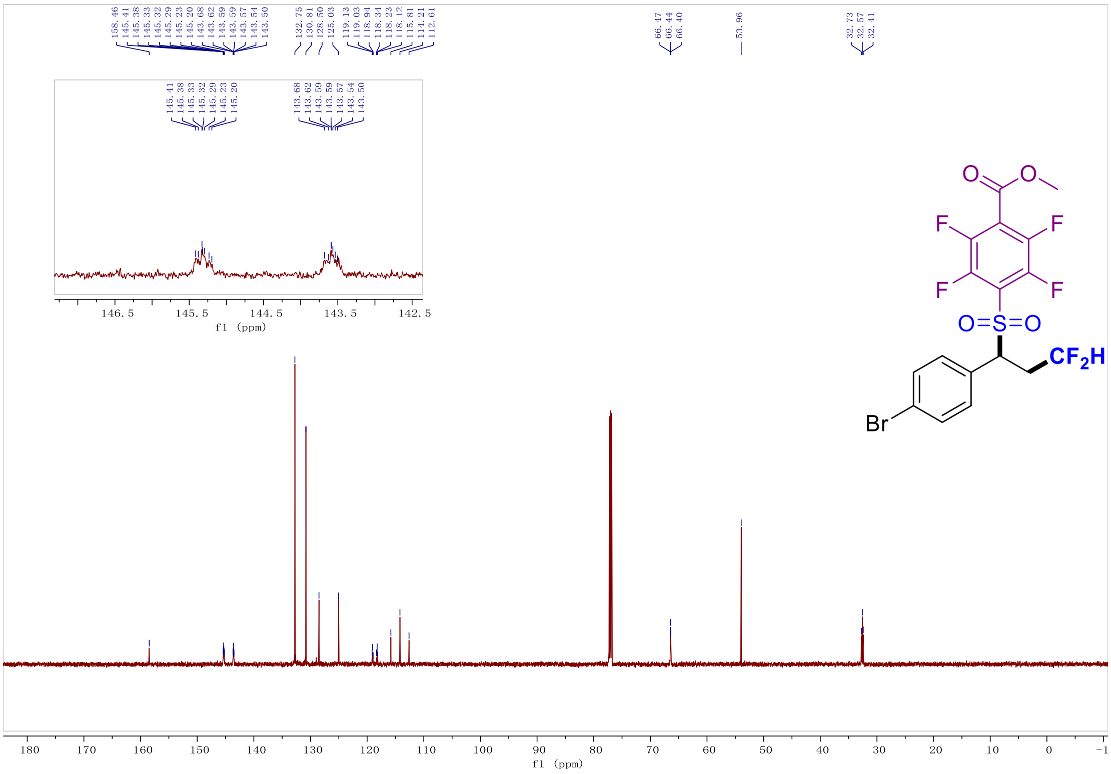

^{19}F NMR (565 MHz, $CDCl_3$) spectrum of 14

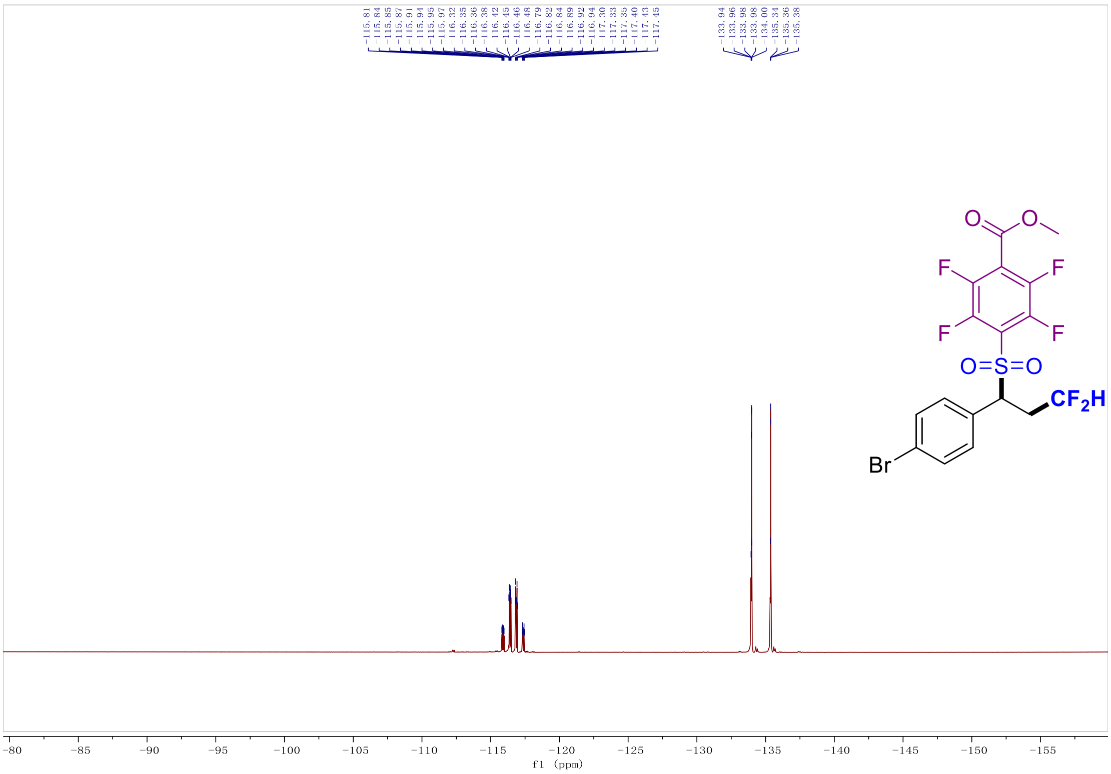

^{1}H NMR (500 MHz, $CDCl_3$) spectrum of 15

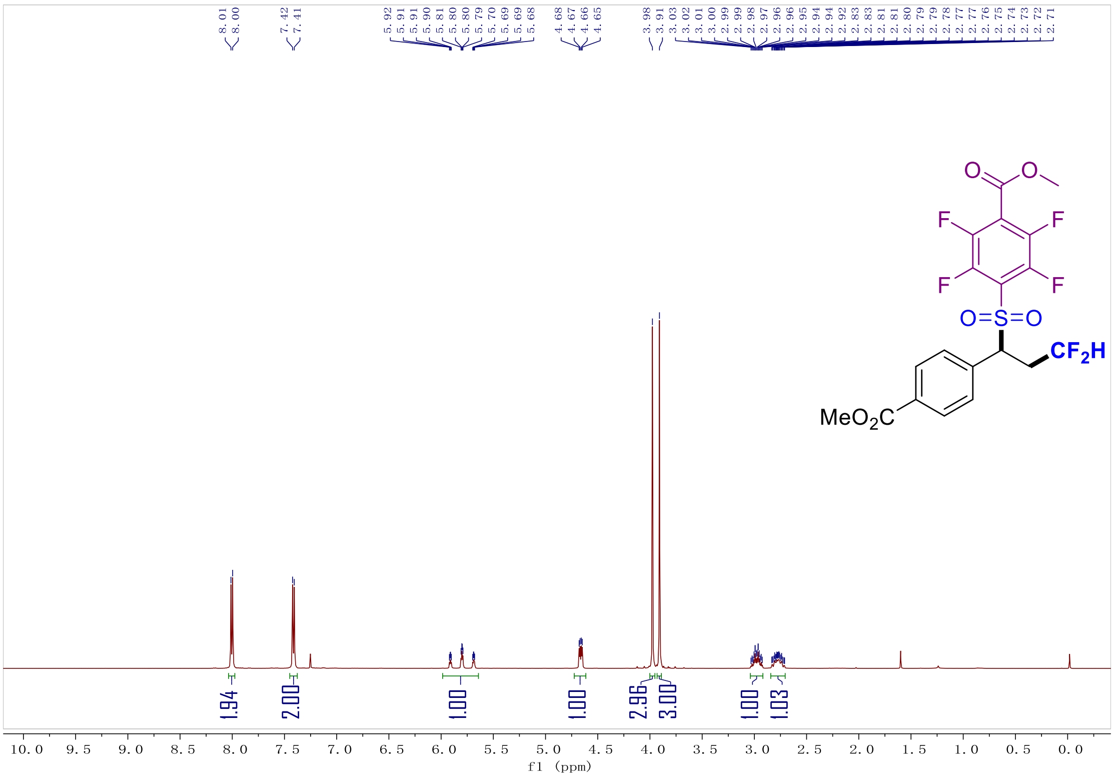

^{13}C NMR (151 MHz, CDCl$_3$) spectrum of 15

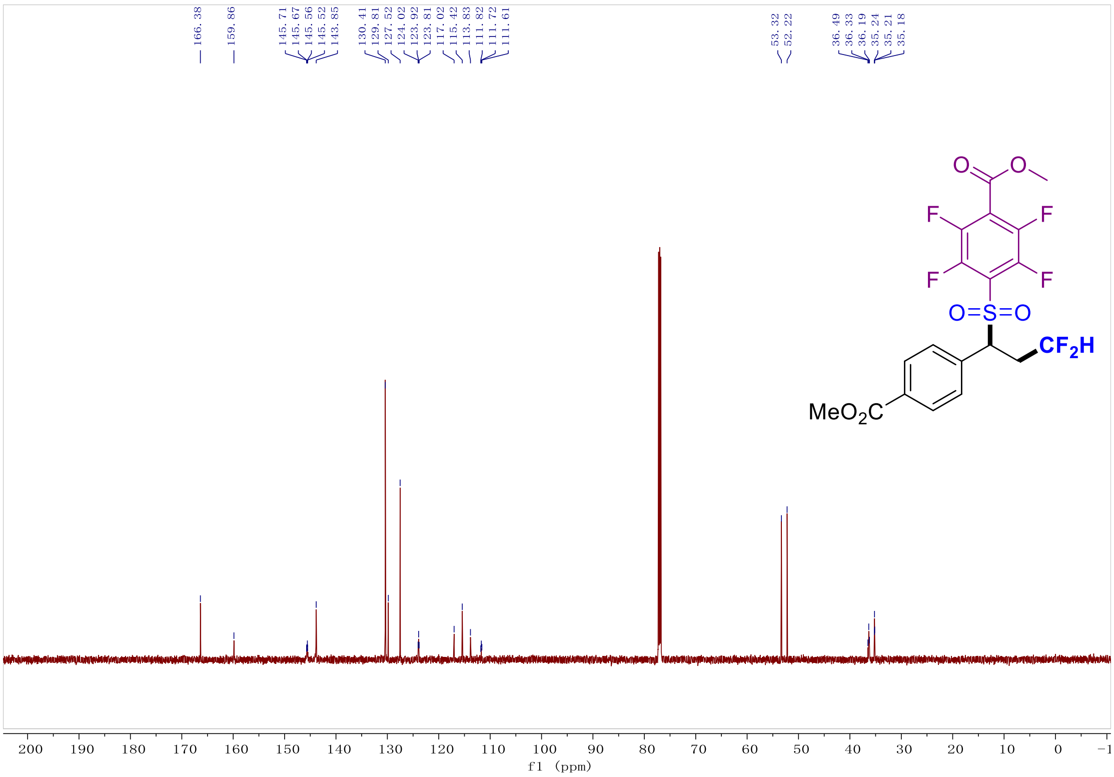

^{19}F NMR (565 MHz, CDCl$_3$) spectrum of 15

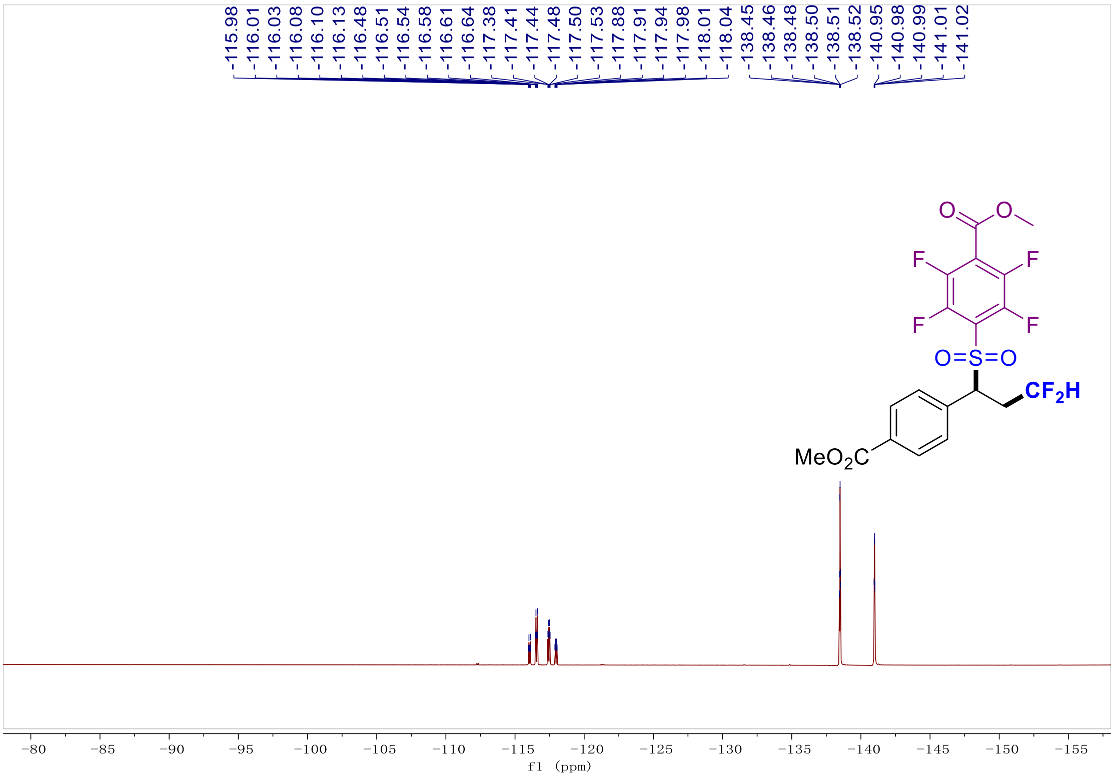

^{1}H NMR (500 MHz, $CDCl_3$) spectrum of 16

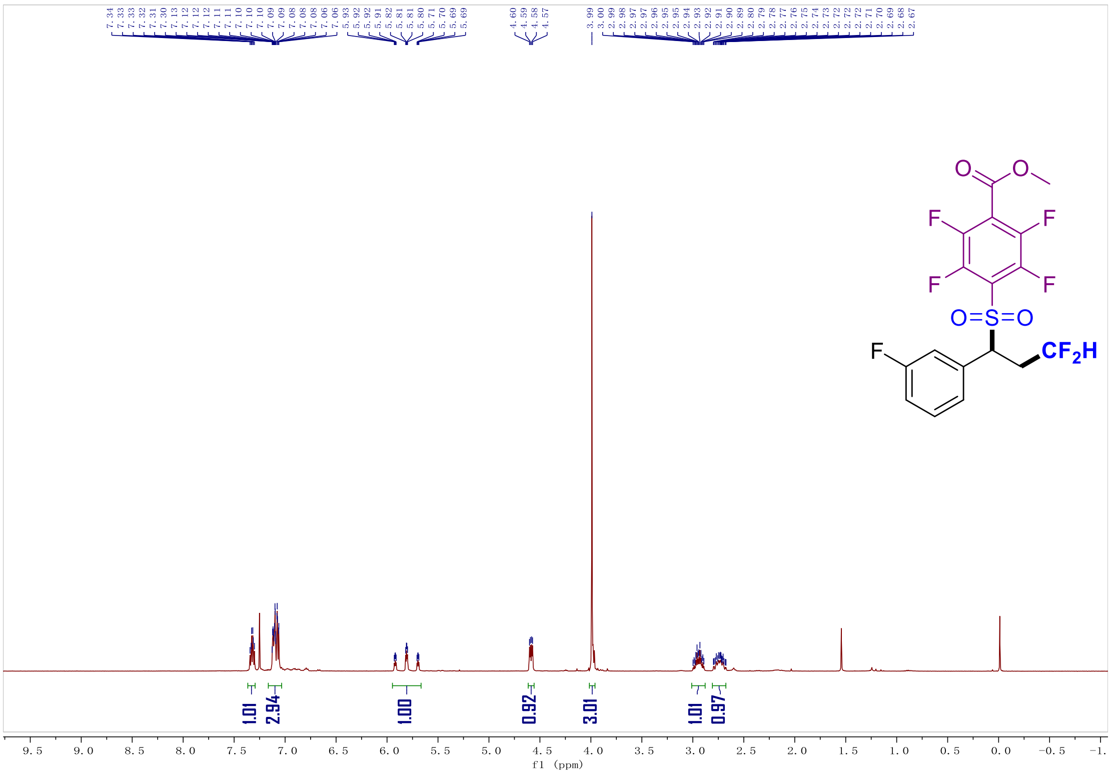

^{13}C NMR (151 MHz, $CDCl_3$) spectrum of 16

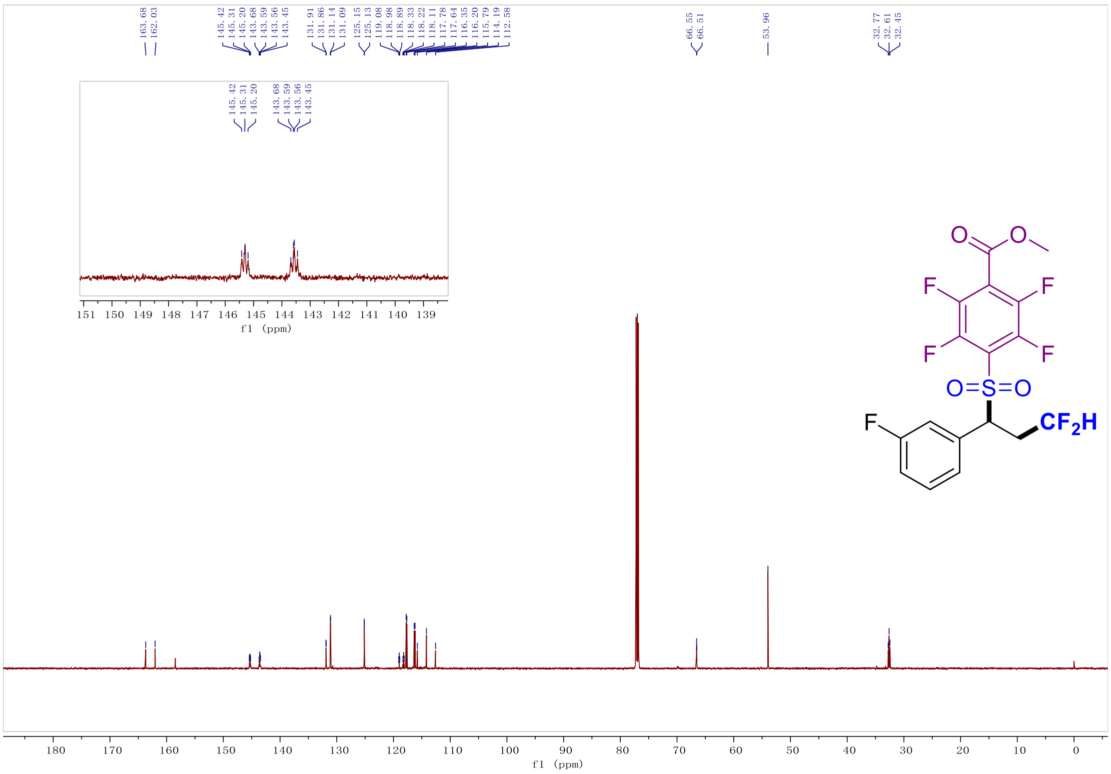

^{19}F NMR (565 MHz, CDCl$_3$) spectrum of 16

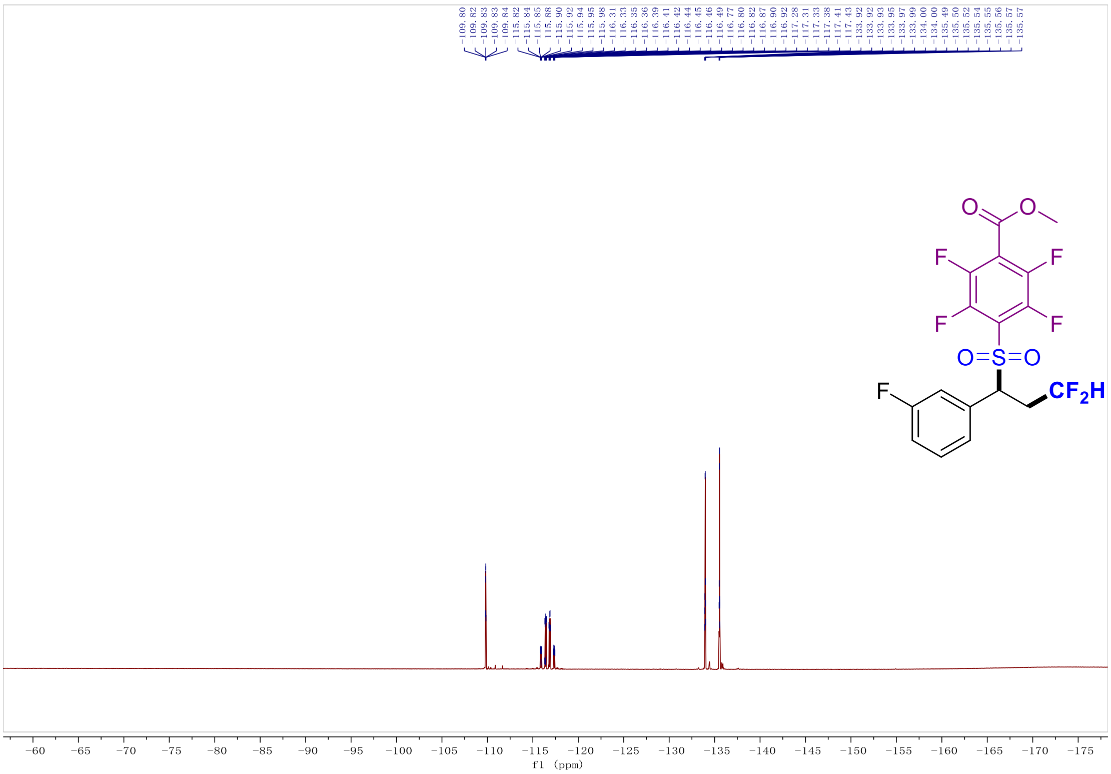

^{1}H NMR (500 MHz, CDCl$_3$) spectrum of 17

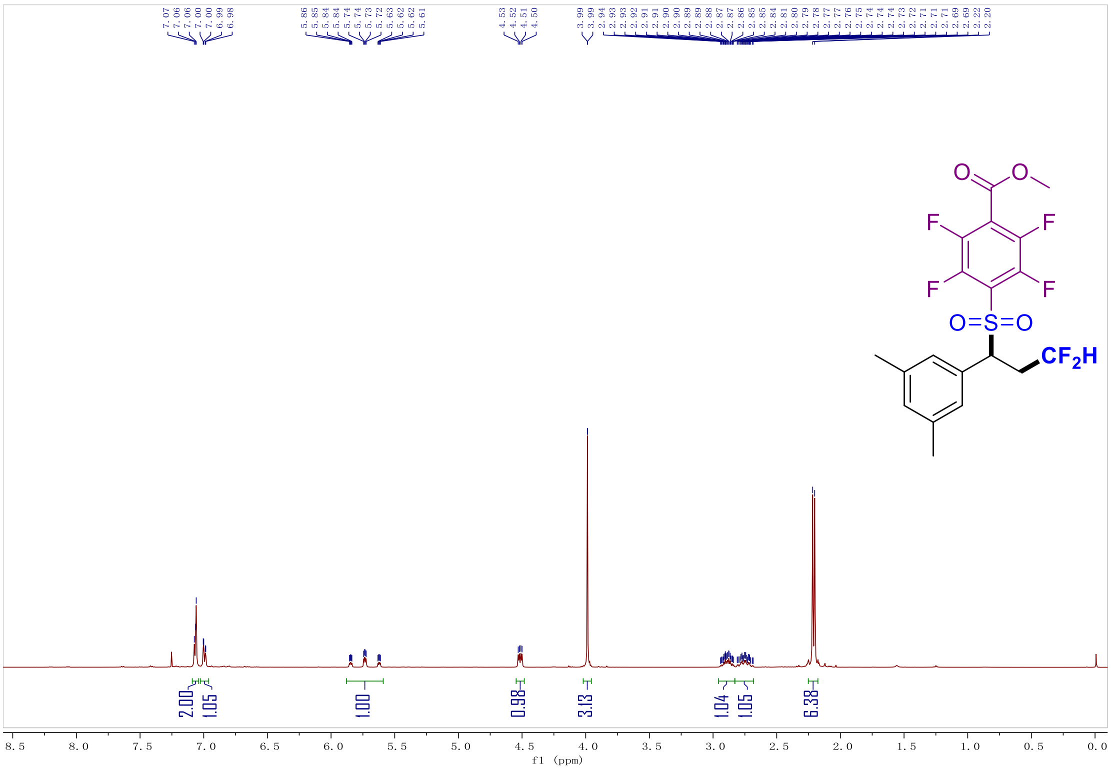

^{13}C NMR (151 MHz, $CDCl_3$) spectrum of 17

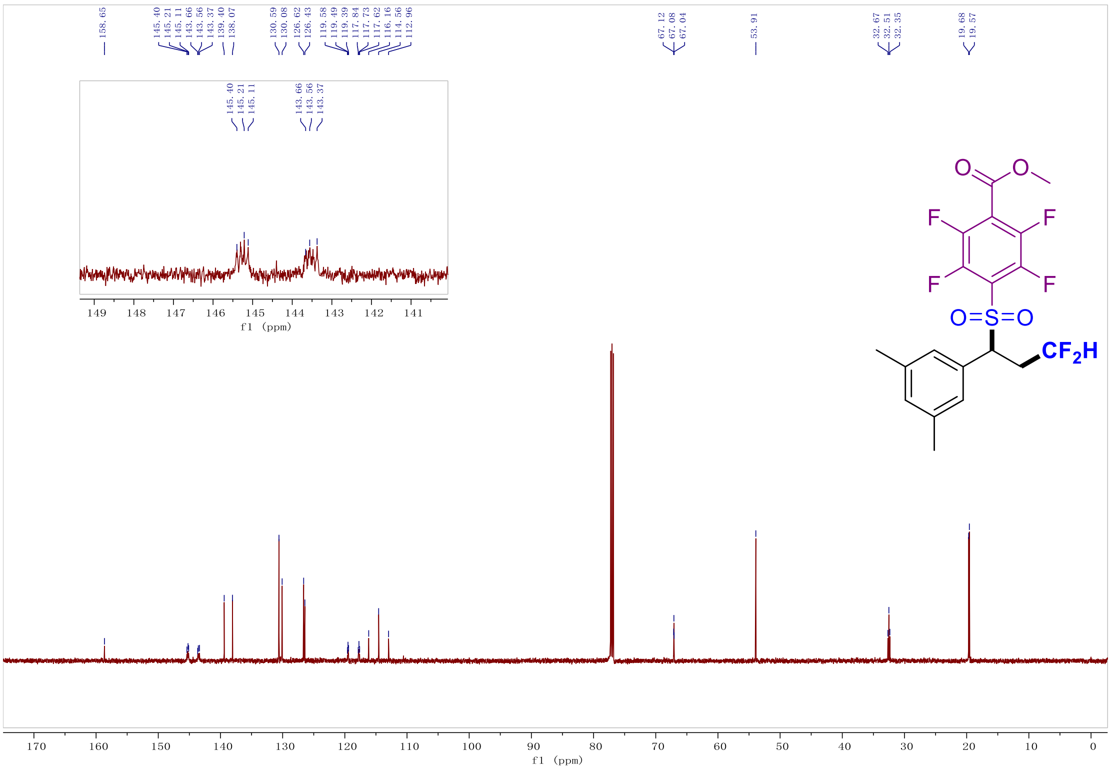

^{19}F NMR (565 MHz, $CDCl_3$) spectrum of 17

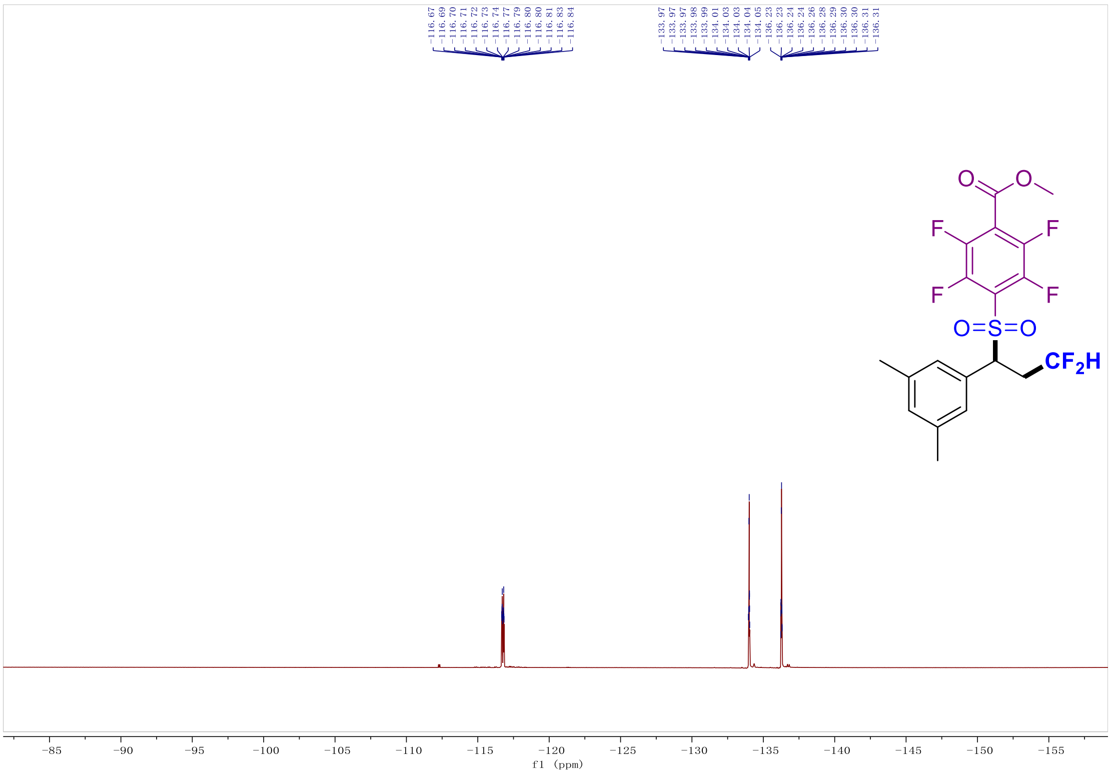

^{1}H NMR (500 MHz, $CDCl_3$) spectrum of 18

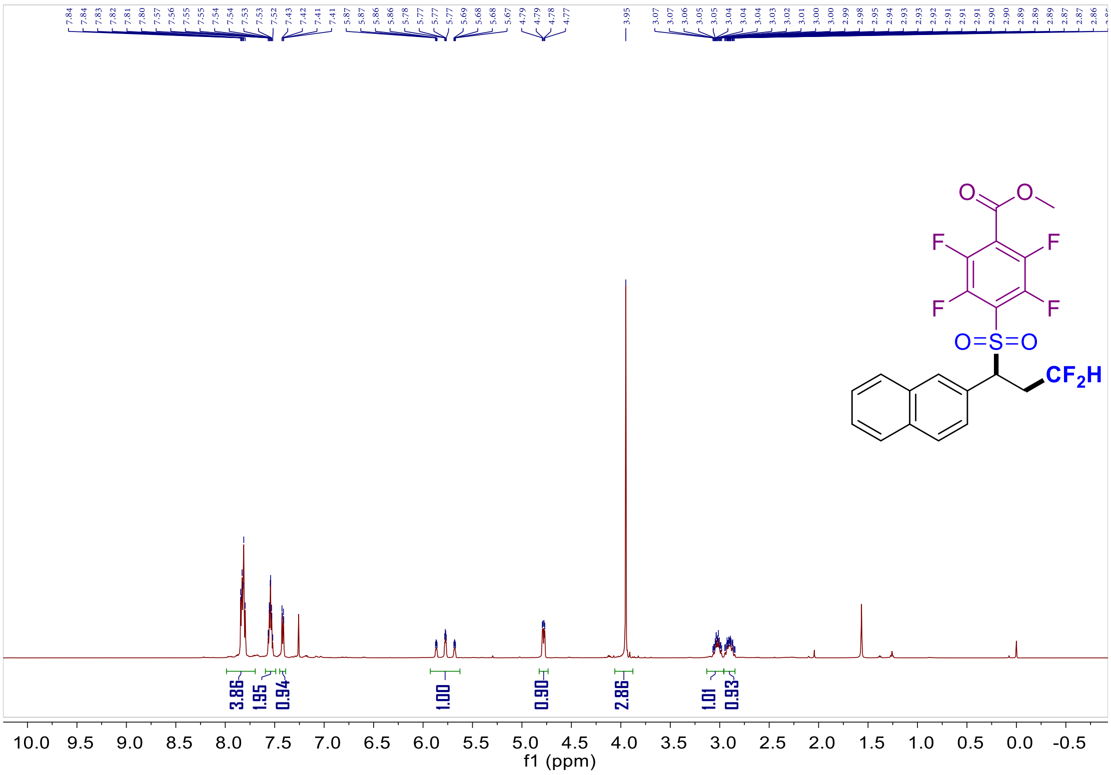

^{13}C NMR (151 MHz, $CDCl_3$) spectrum of 18

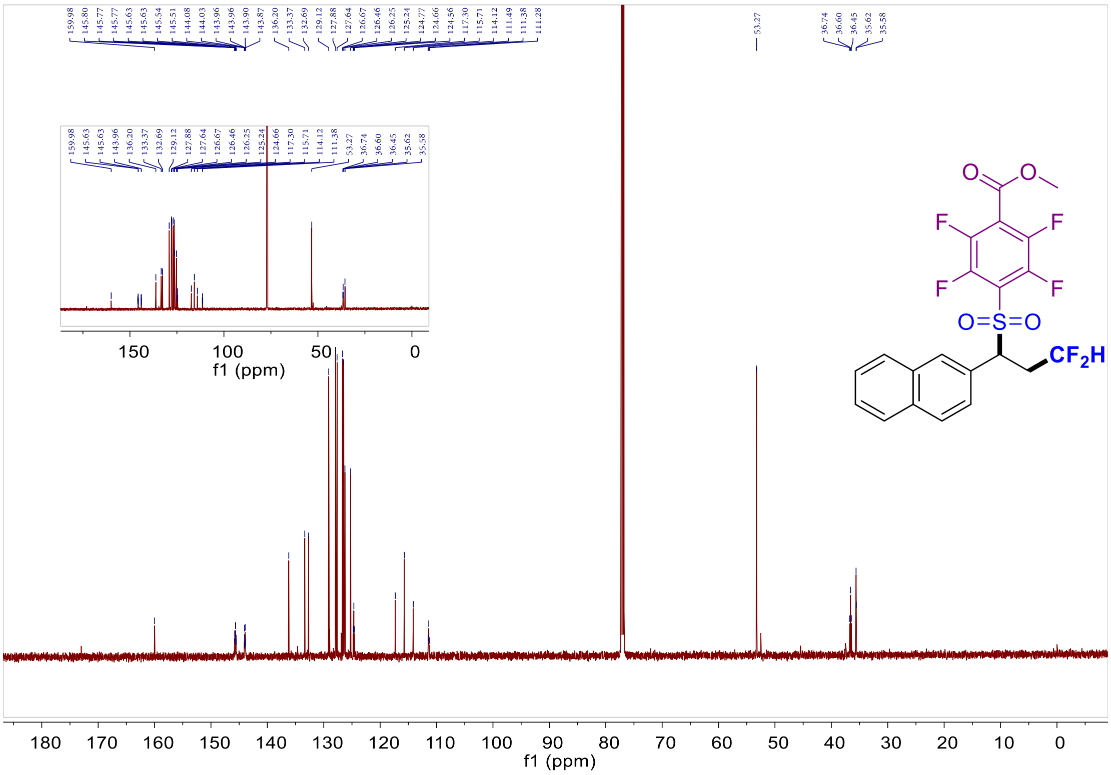

^{19}F NMR (565 MHz, CDCl$_3$) spectrum of 18

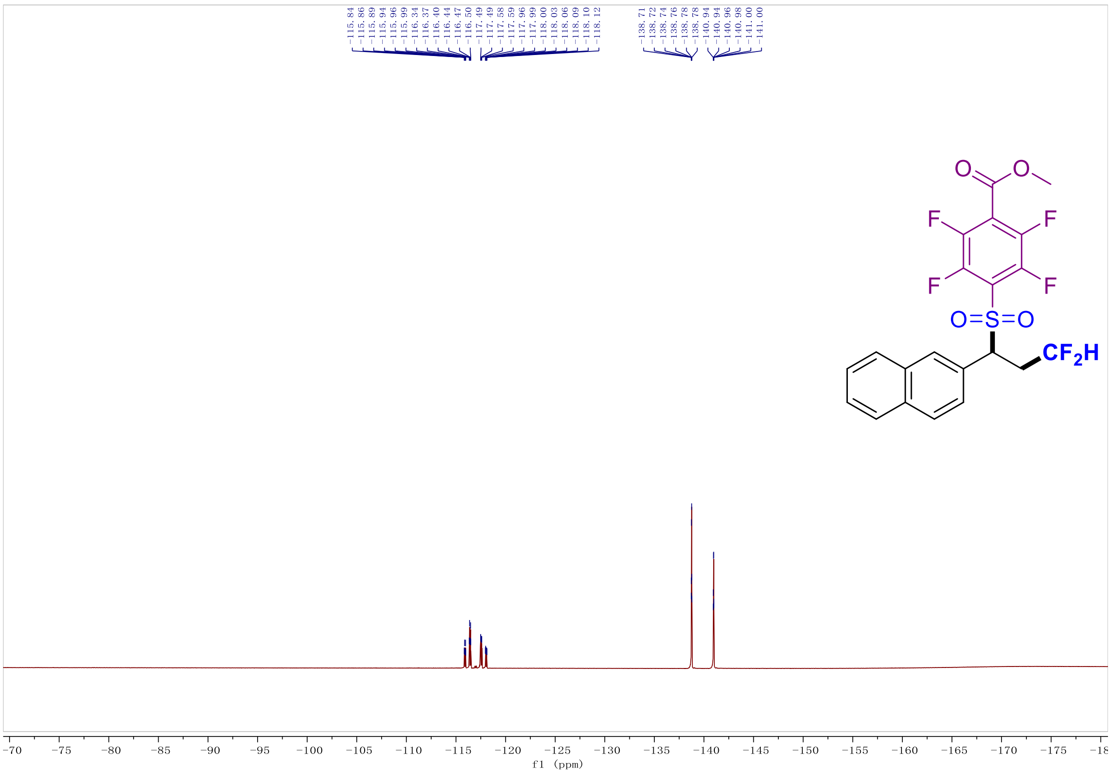

^{1}H NMR (500 MHz, CDCl$_3$) spectrum of 19

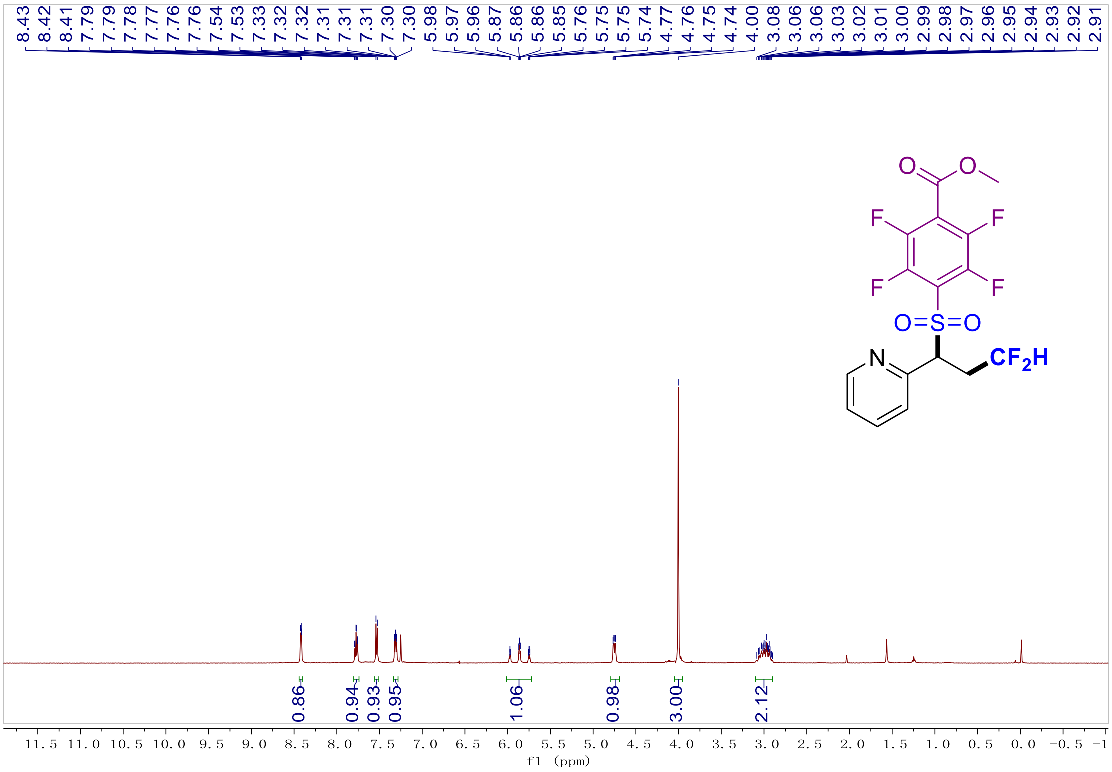

^{13}C NMR (151 MHz, $CDCl_3$) spectrum of 19

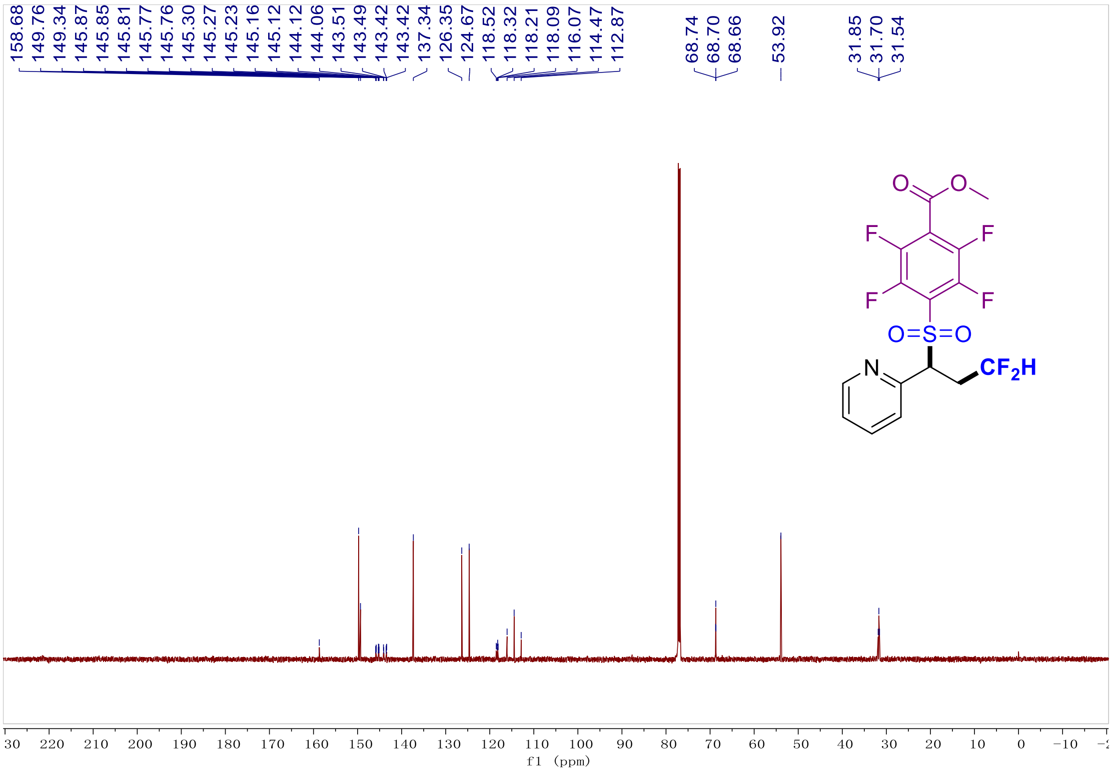

^{19}F NMR (565 MHz, $CDCl_3$) spectrum of 19

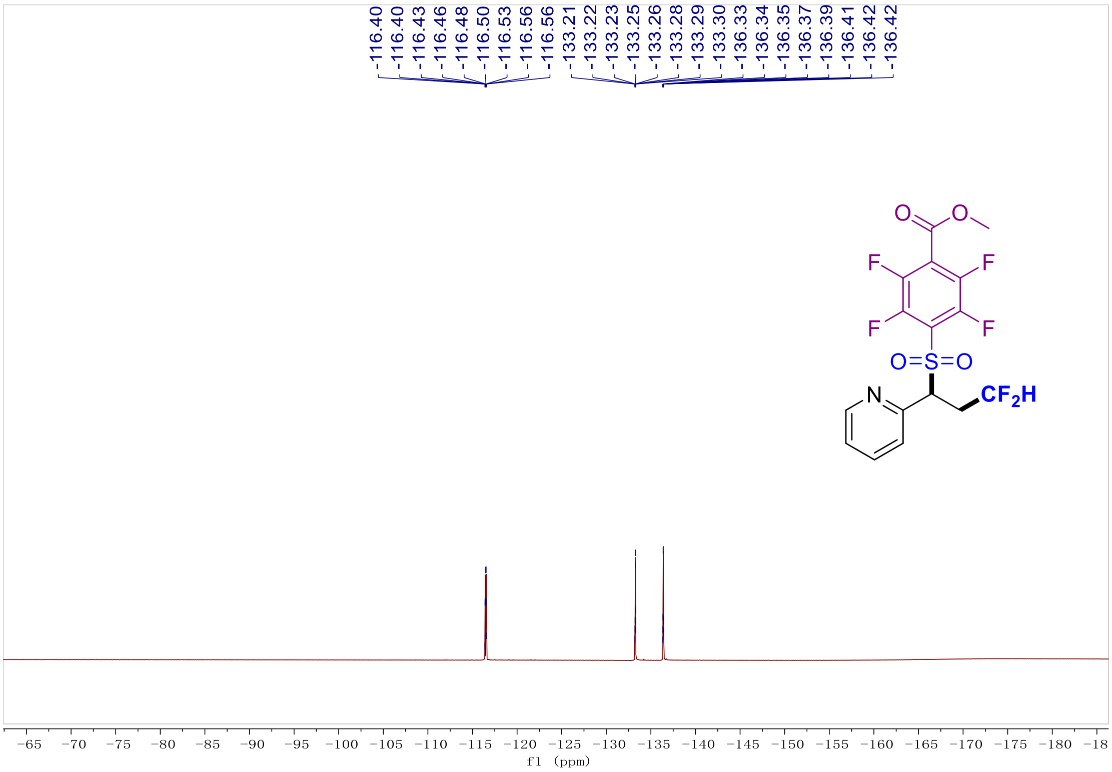

^{1}H NMR (600 MHz, $CDCl_3$) spectrum of 20

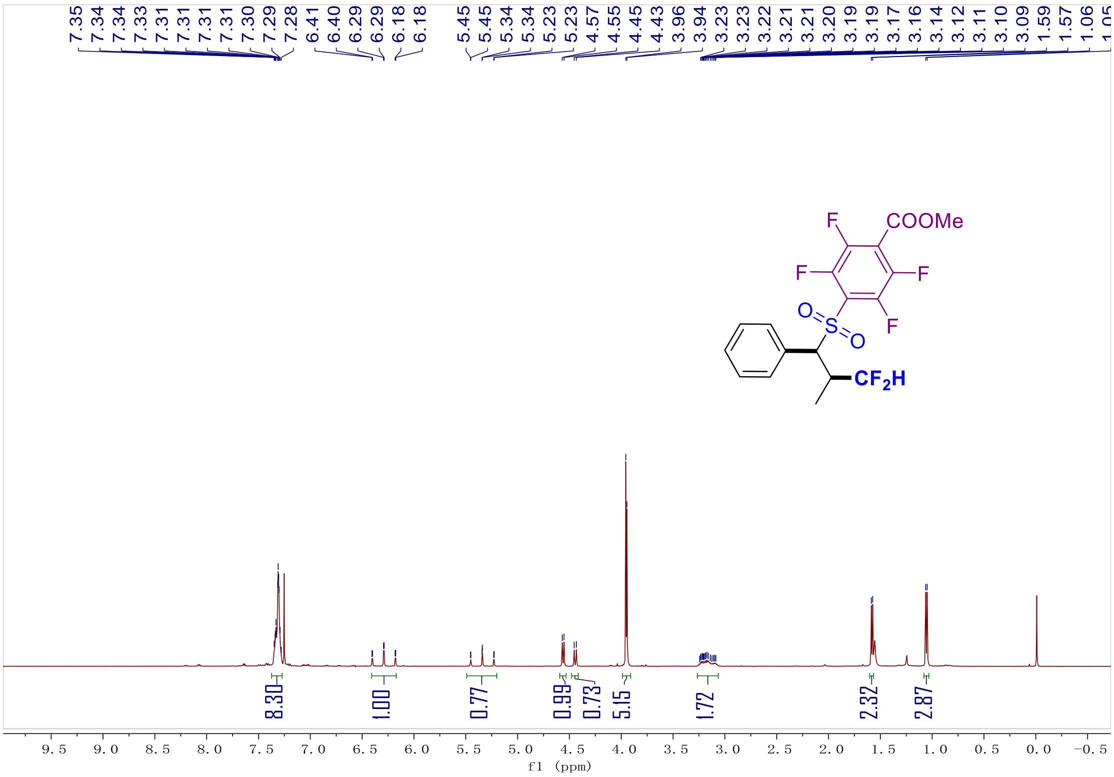

^{13}C NMR (151 MHz, $CDCl_3$) spectrum of 20

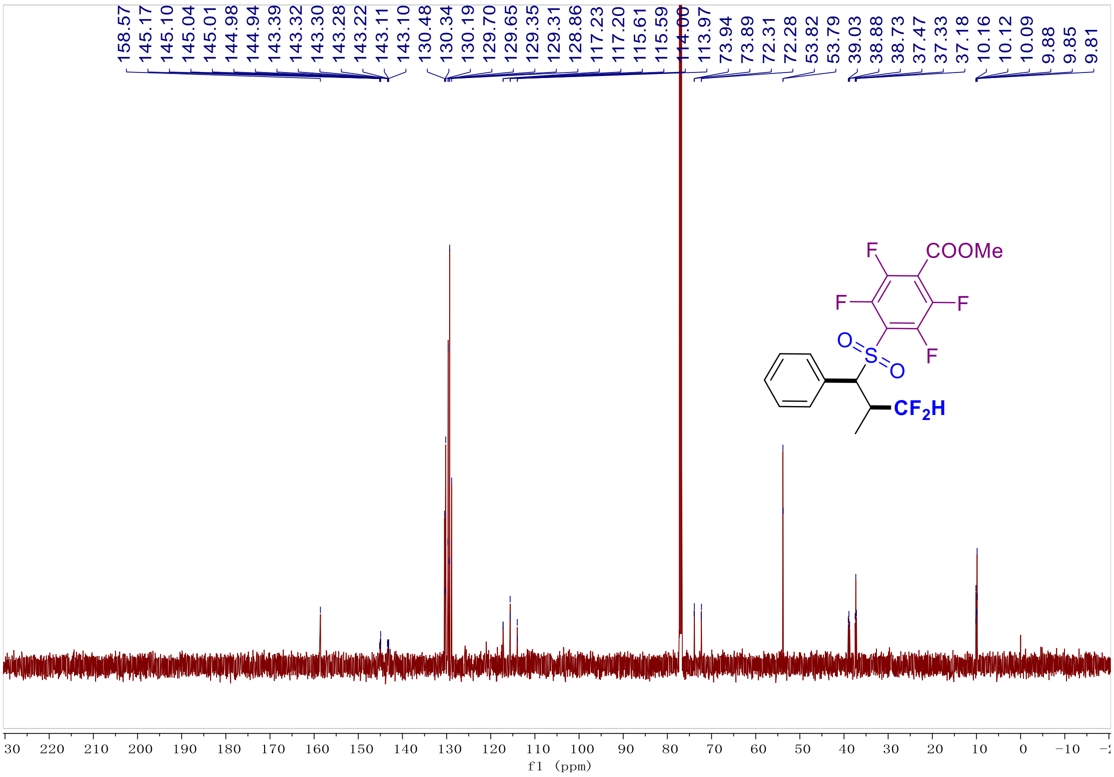

^{19}F NMR (565 MHz, $CDCl_3$) spectrum of 20

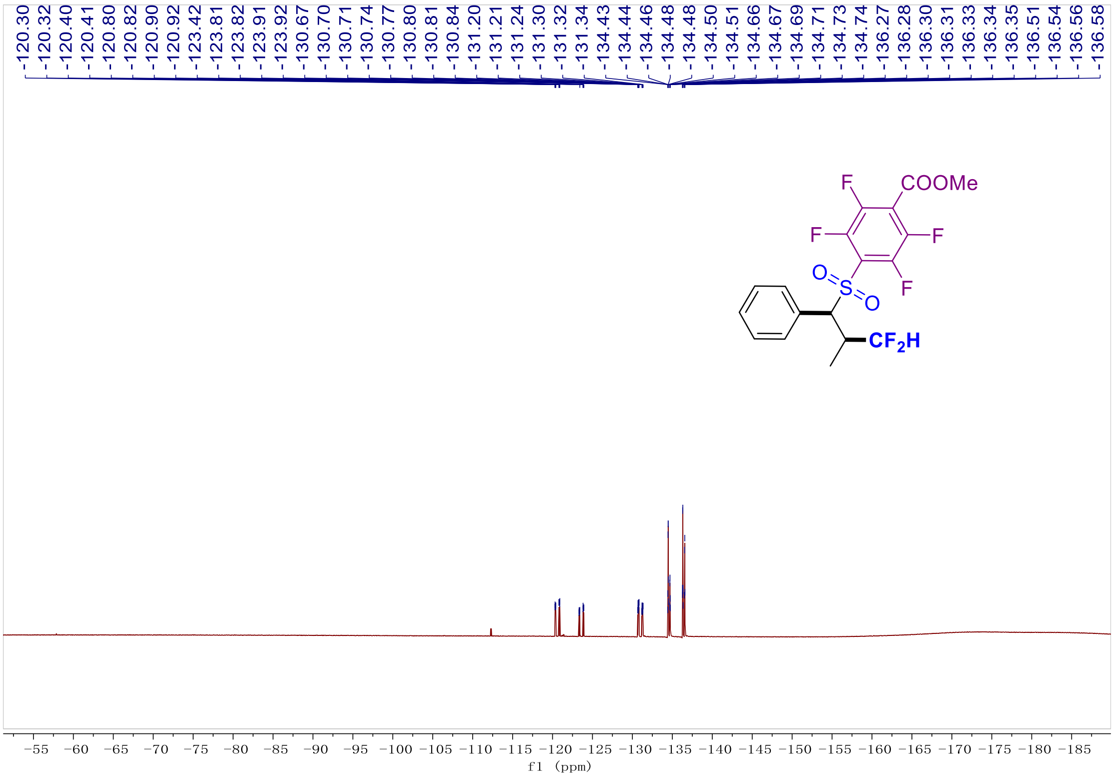

^{1}H NMR (600 MHz, $CDCl_3$) spectrum of 21

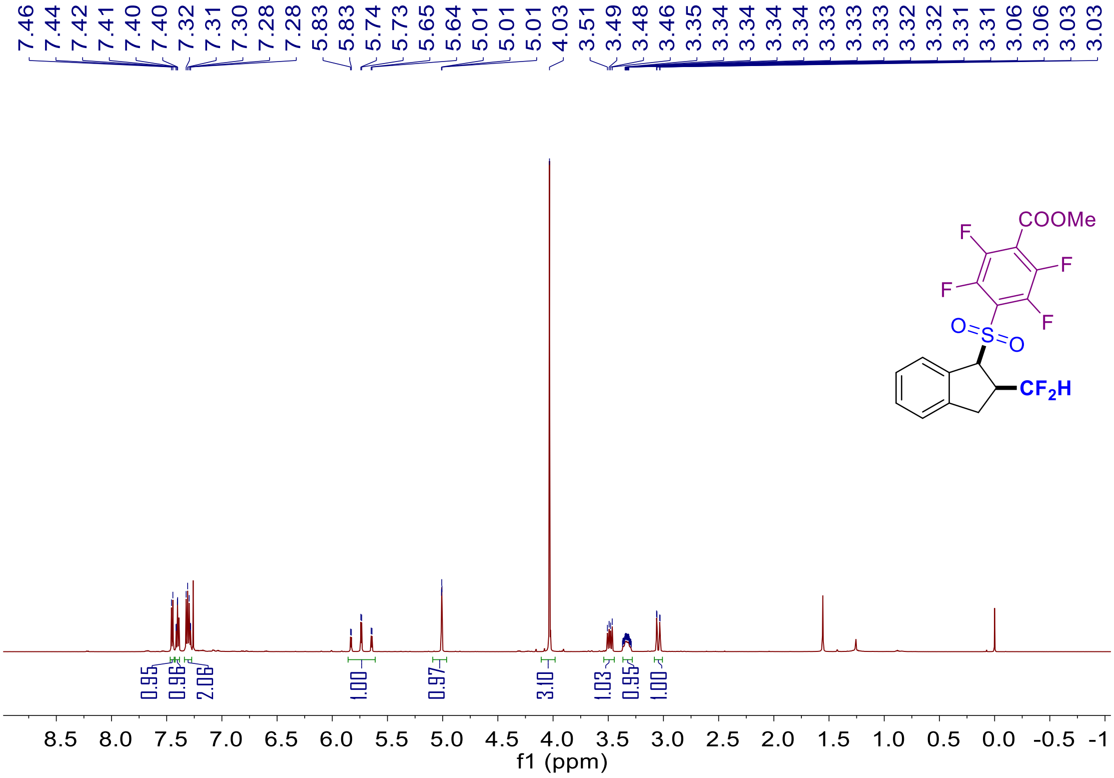

^{13}C NMR (151 MHz, $CDCl_3$) spectrum of 21

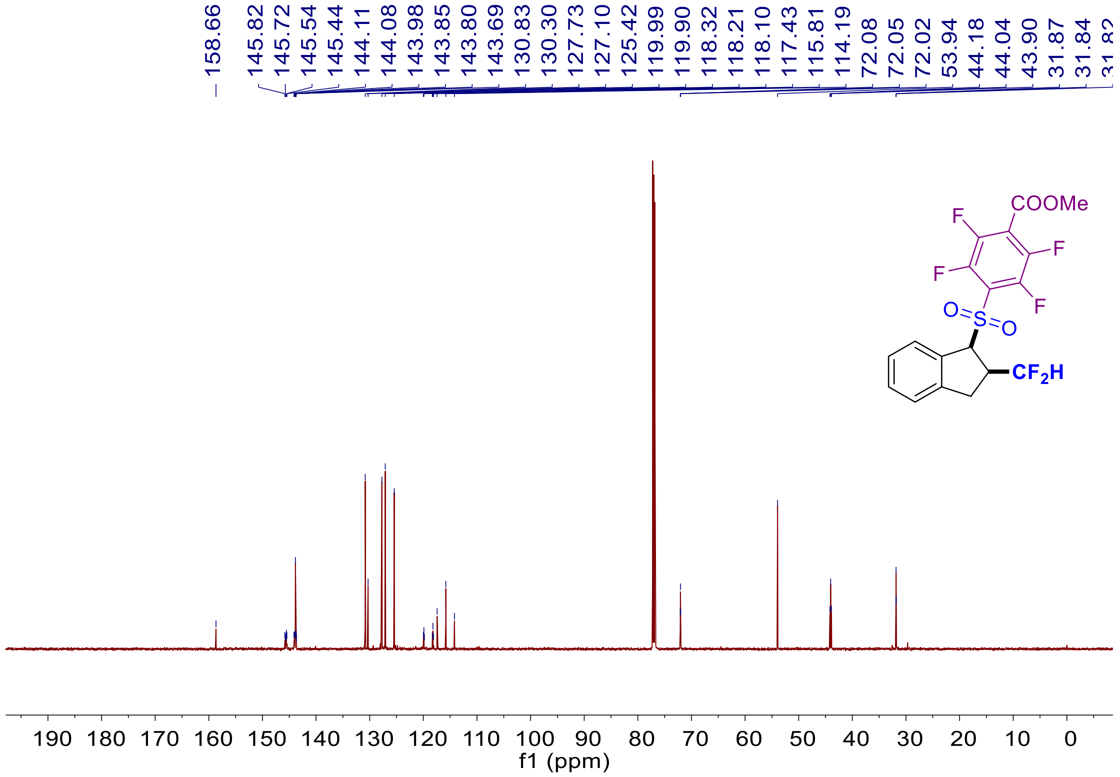

^{19}F NMR (565 MHz, $CDCl_3$) spectrum of 21

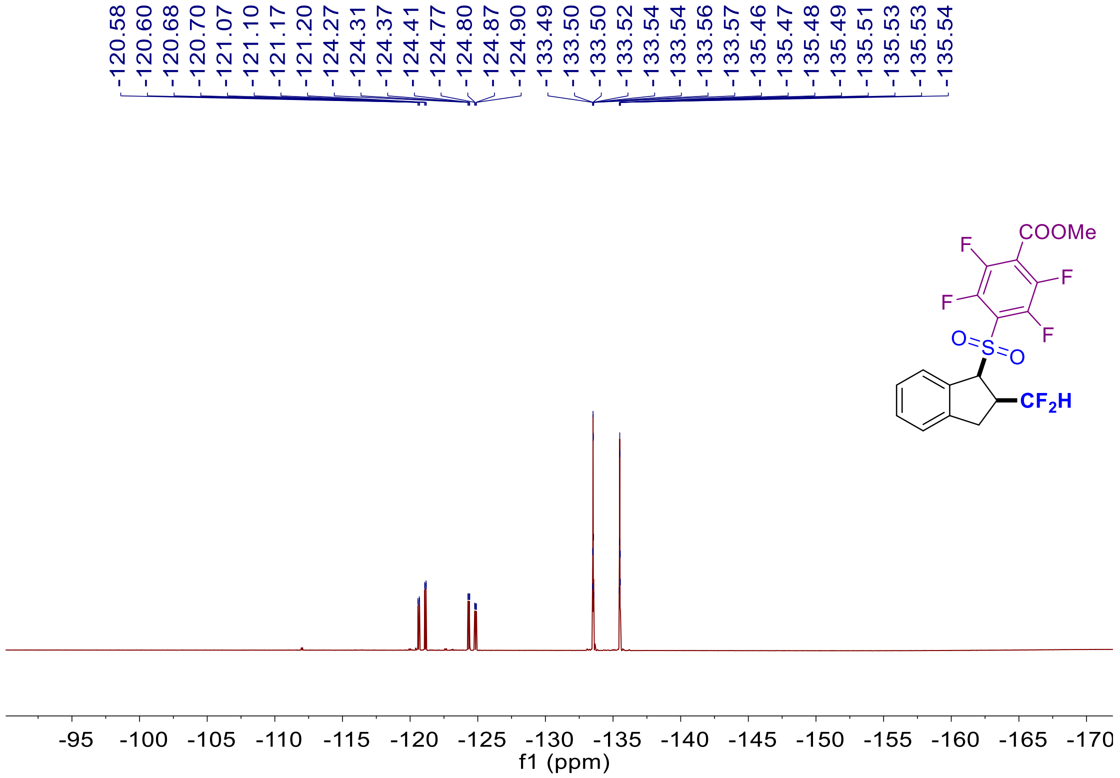

^1H NMR (500 MHz, $CDCl_3$) spectrum of 22

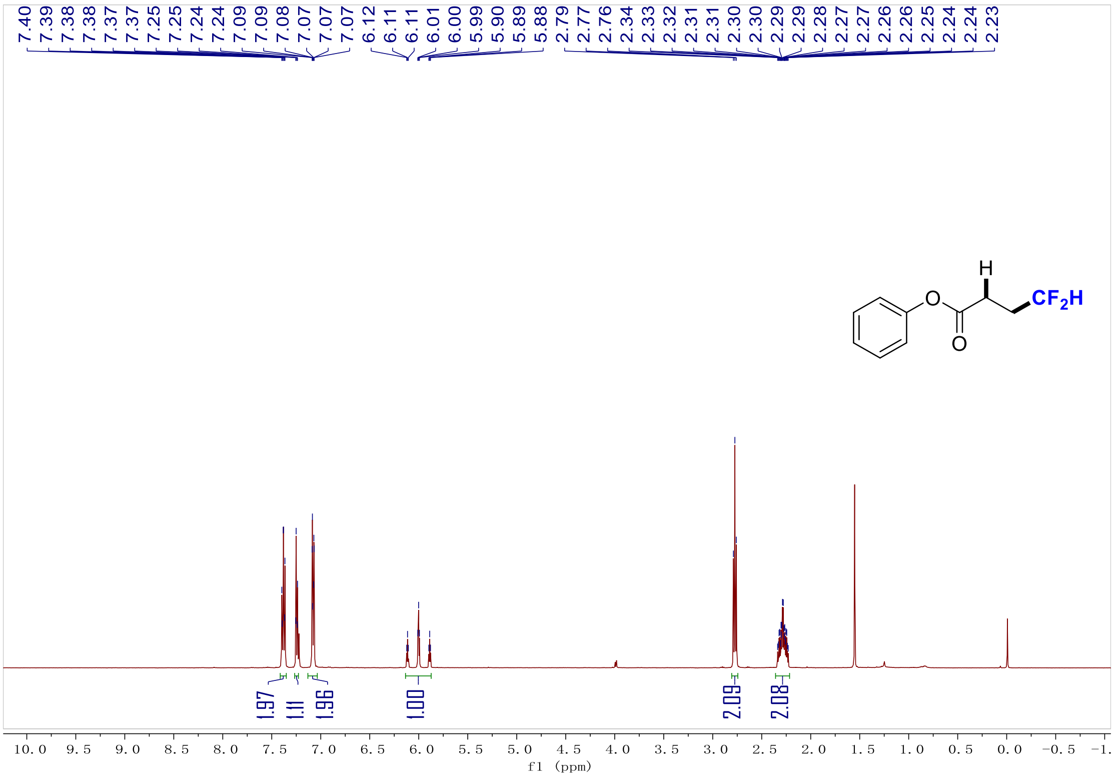

^{13}C NMR (151 MHz, $CDCl_3$) spectrum of 22

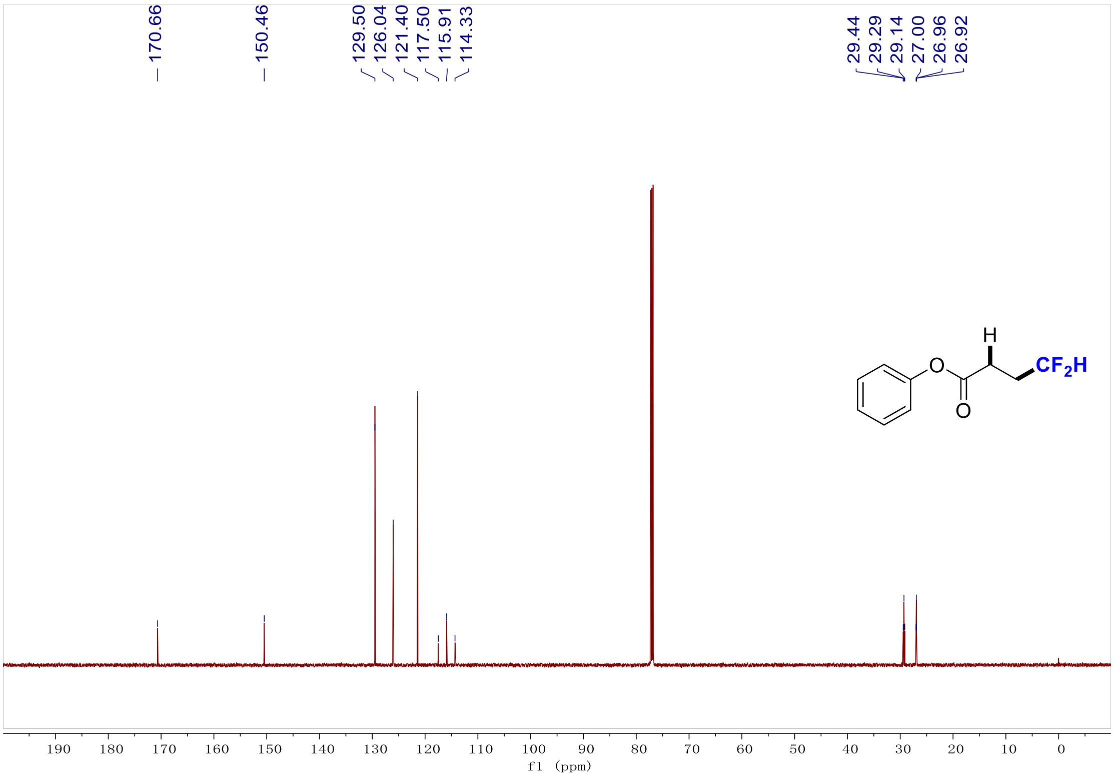

^{19}F NMR (565 MHz, CDCl3) spectrum of 22

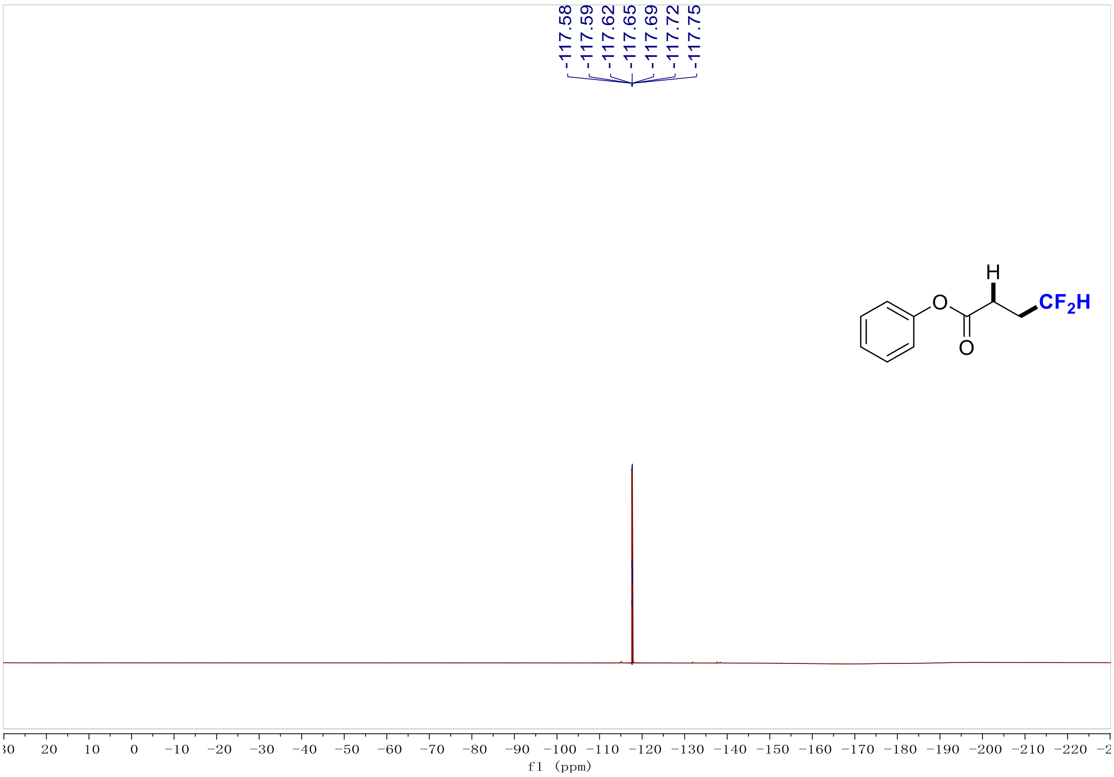

^{1}H NMR (500 MHz, CDCl3) spectrum of 23

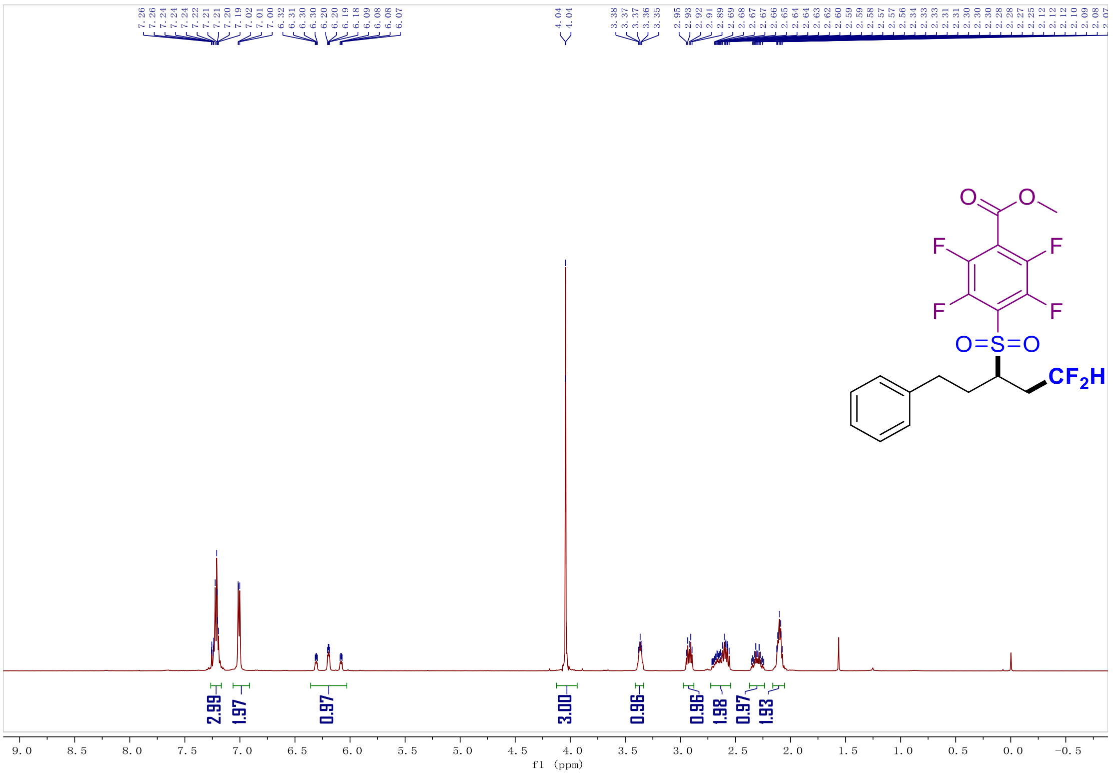

^{13}C NMR (151 MHz, $CDCl_3$) spectrum of 23

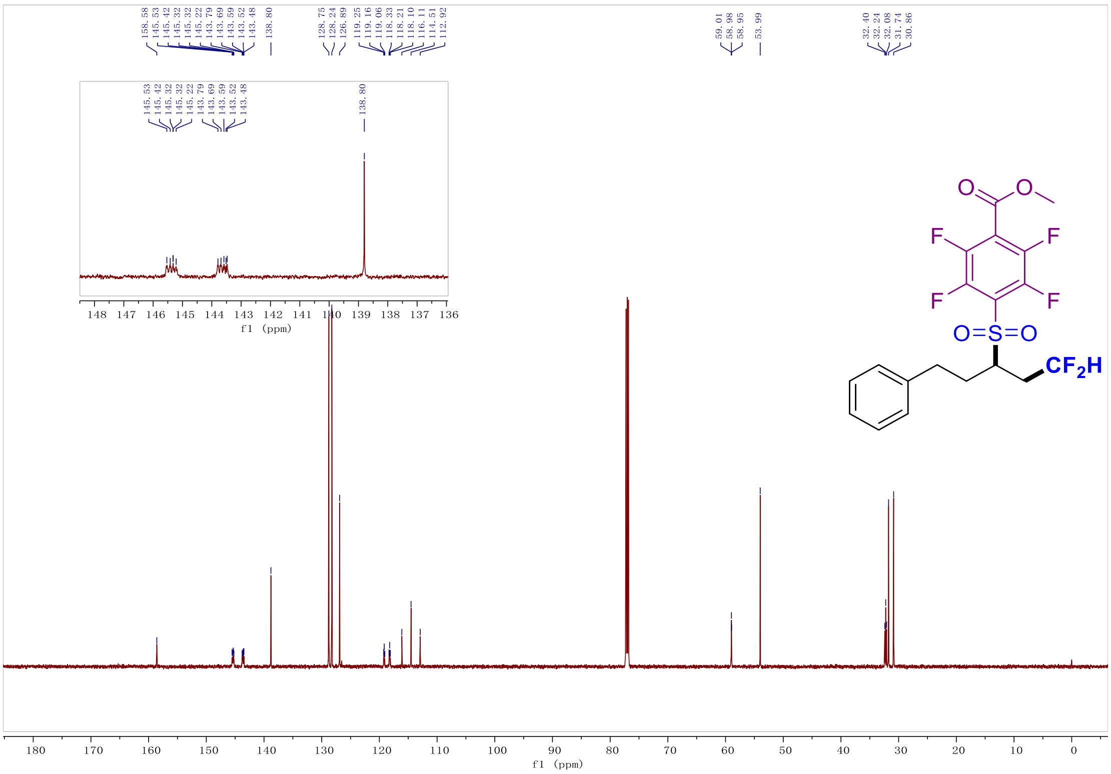

^{19}F NMR (565 MHz, $CDCl_3$) spectrum of 23

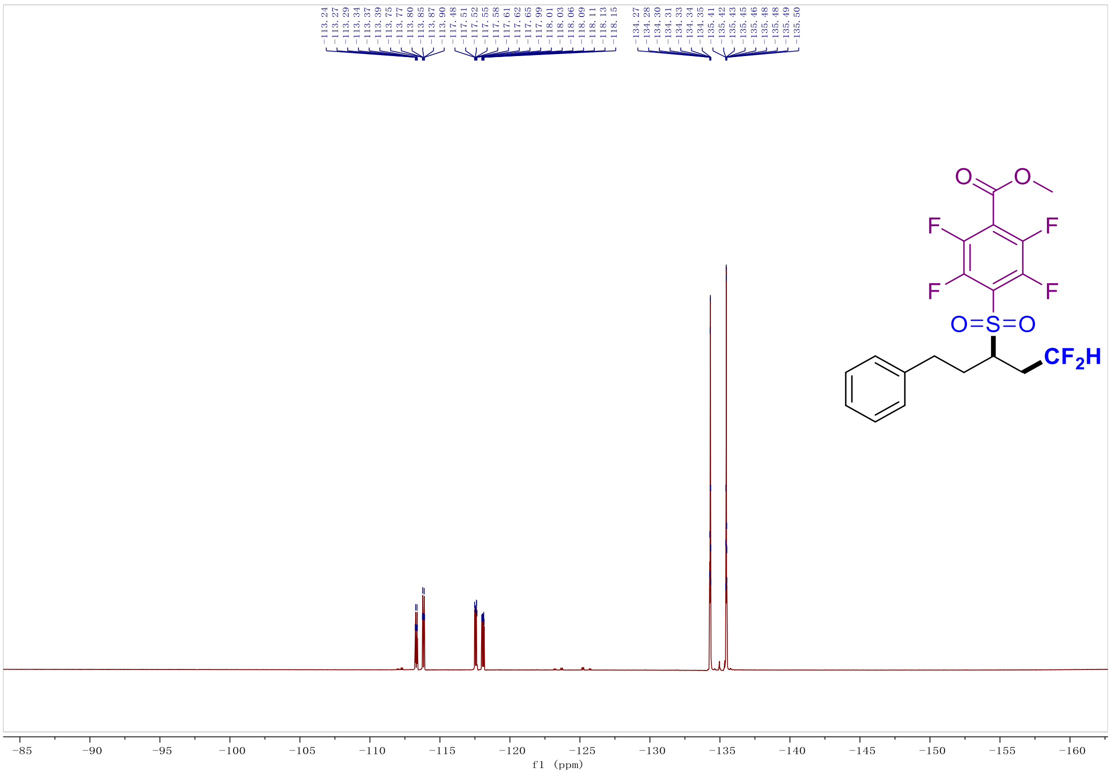

^{1}H NMR (500 MHz, $CDCl_3$) spectrum of 24

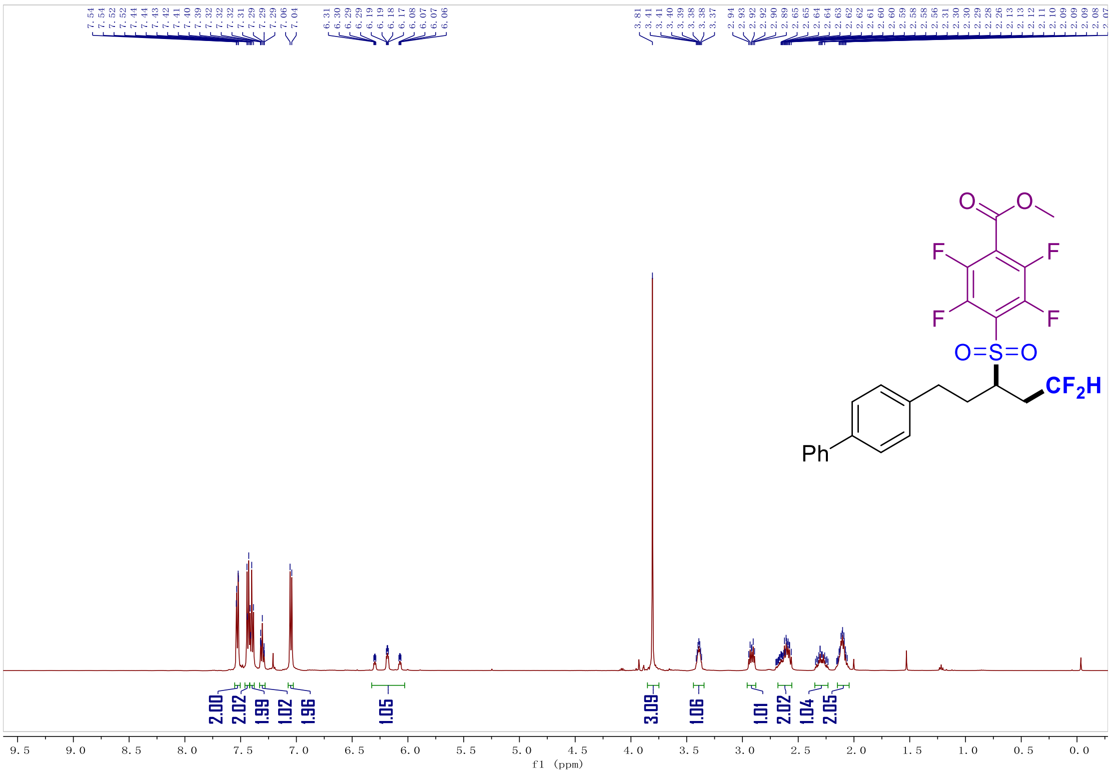

^{13}C NMR (151 MHz, $CDCl_3$) spectrum of 24

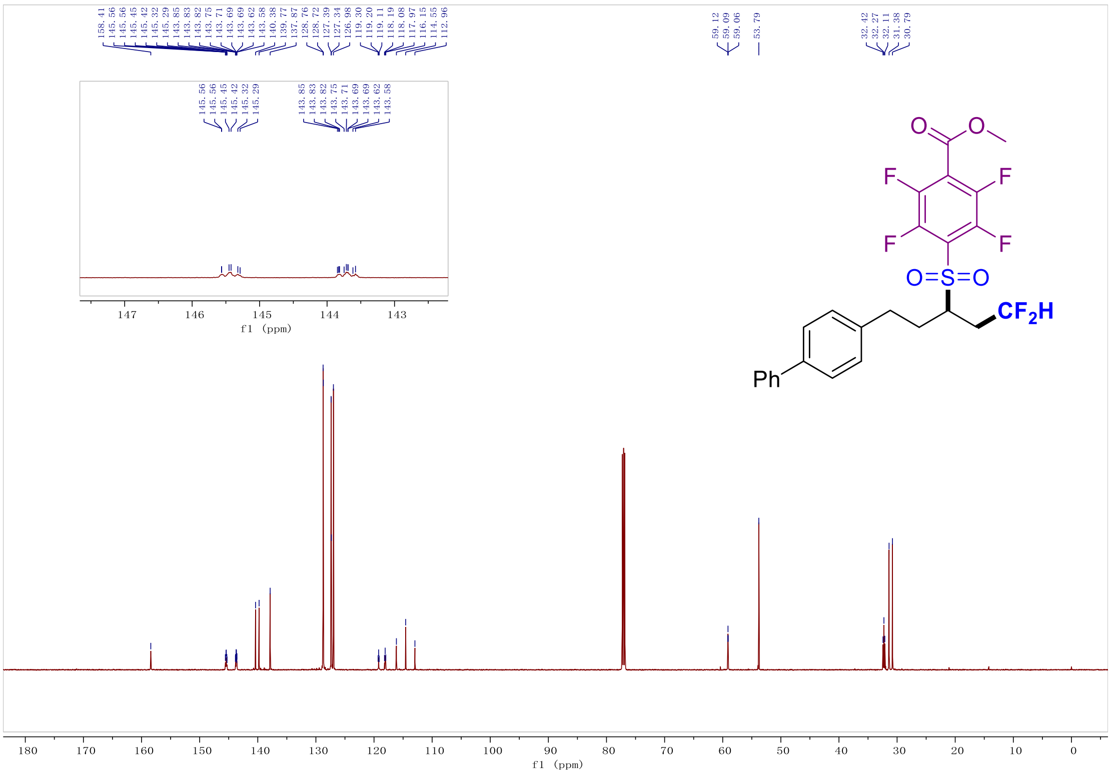

^{19}F NMR (565 MHz, CDCl$_3$) spectrum of 24

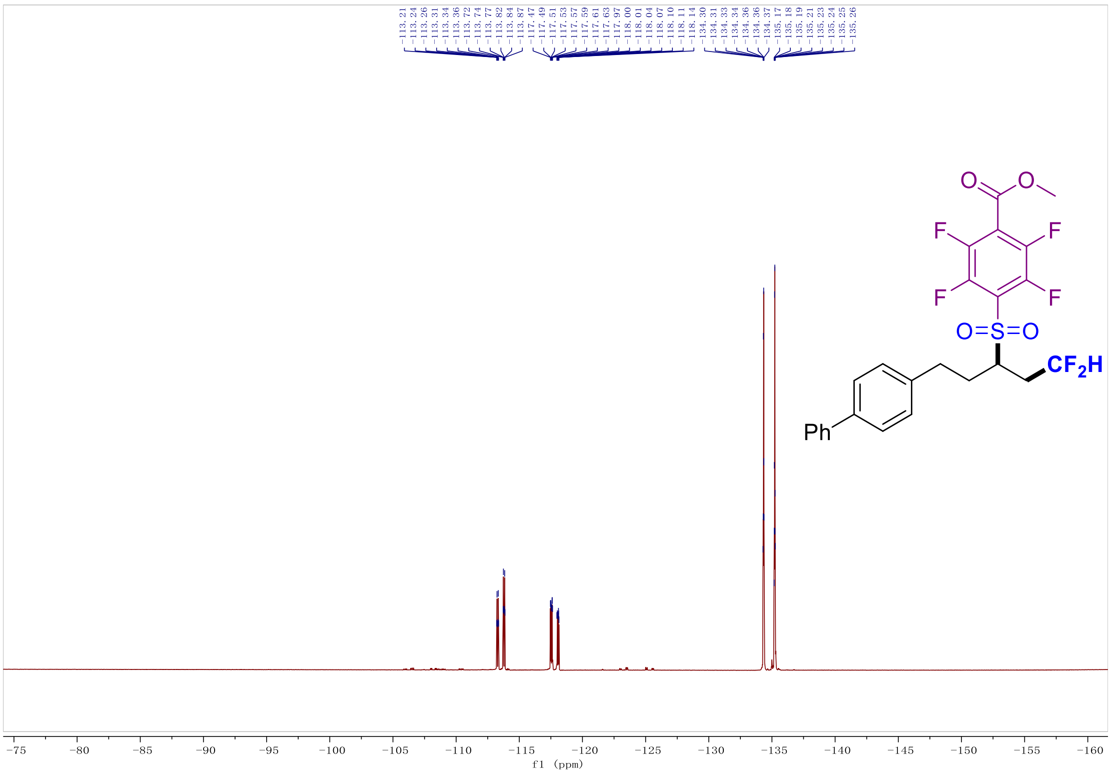

^{1}H NMR (500 MHz, CDCl$_3$) spectrum of 25

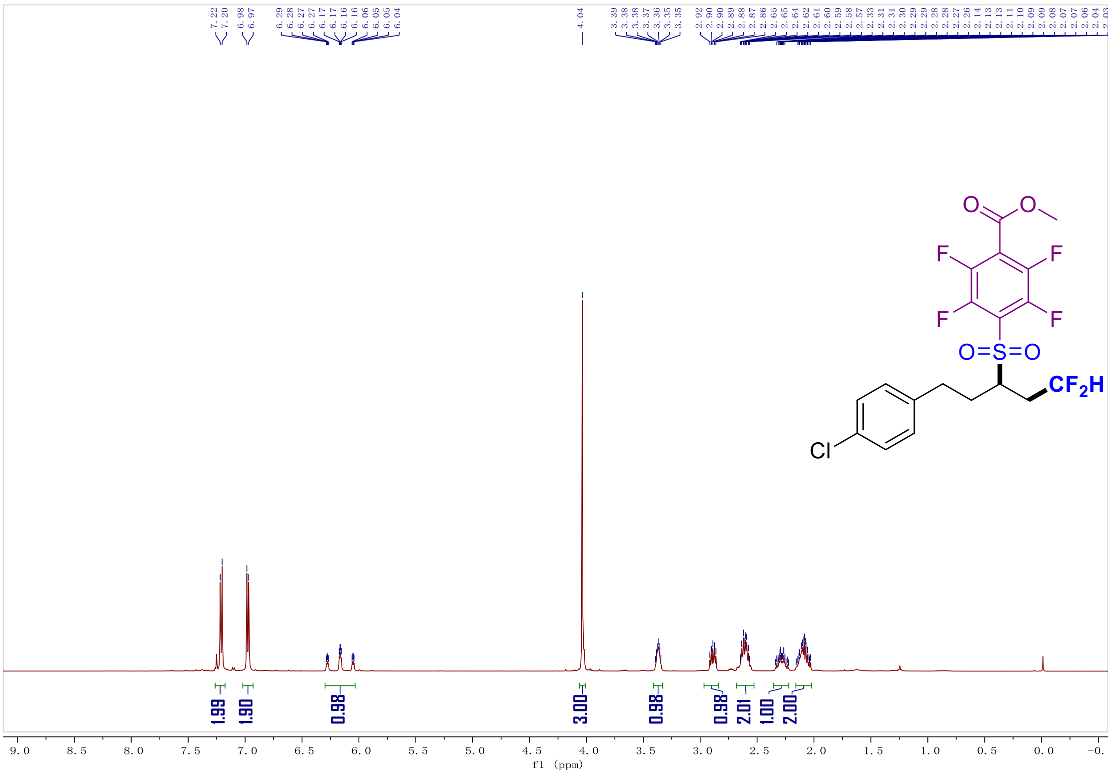

^{13}C NMR (151 MHz, $CDCl_3$) spectrum of 25

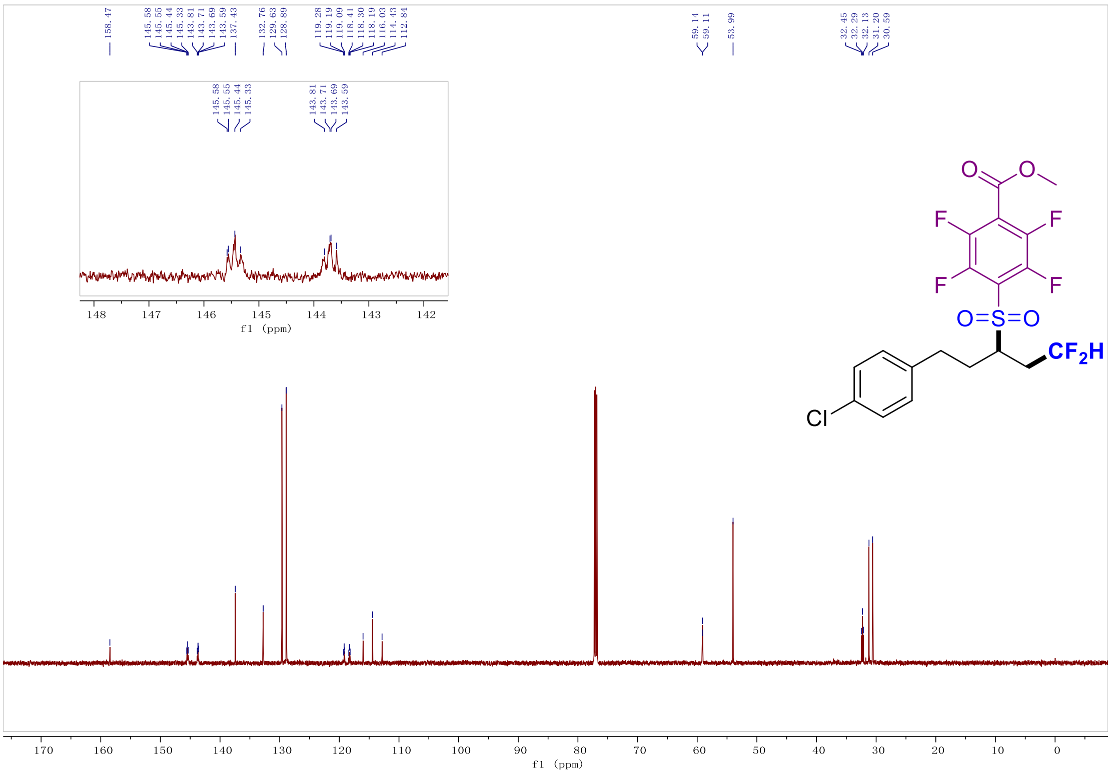

^{19}F NMR (565 MHz, $CDCl_3$) spectrum of 25

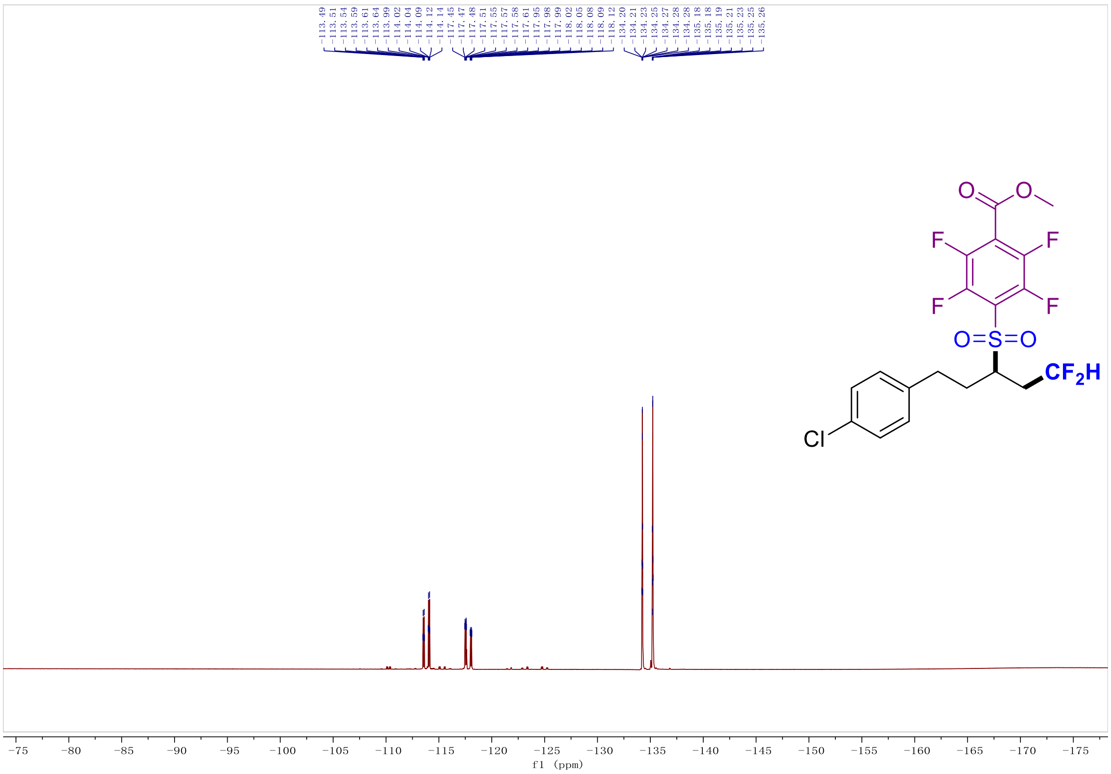

^{1}H NMR (600 MHz, $CDCl_3$) spectrum of 26

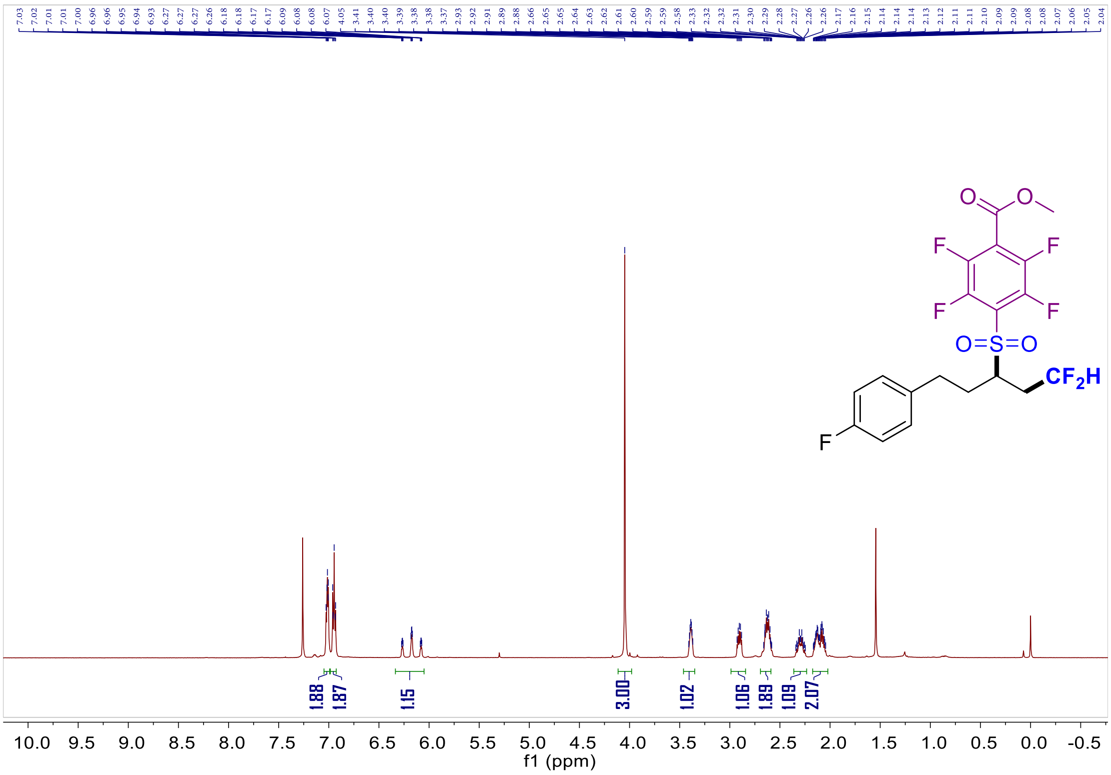

^{13}C NMR (151 MHz, $CDCl_3$) spectrum of 26

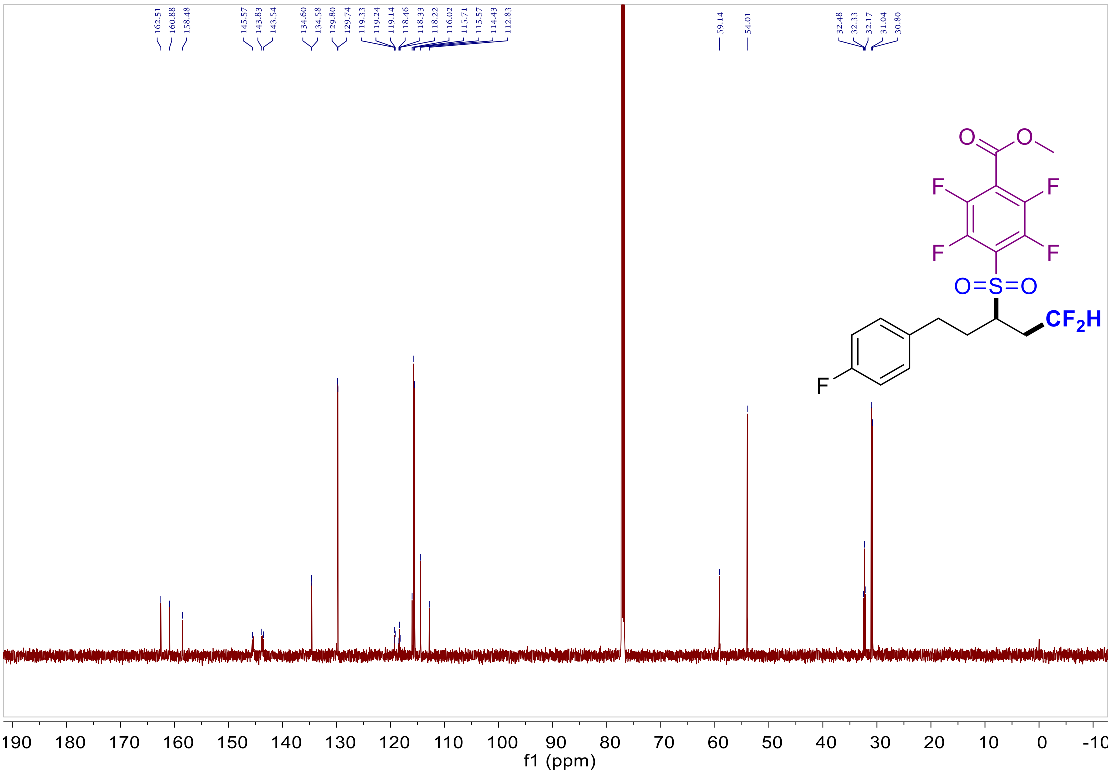

^{19}F NMR (565 MHz, CDCl$_3$) spectrum of 26

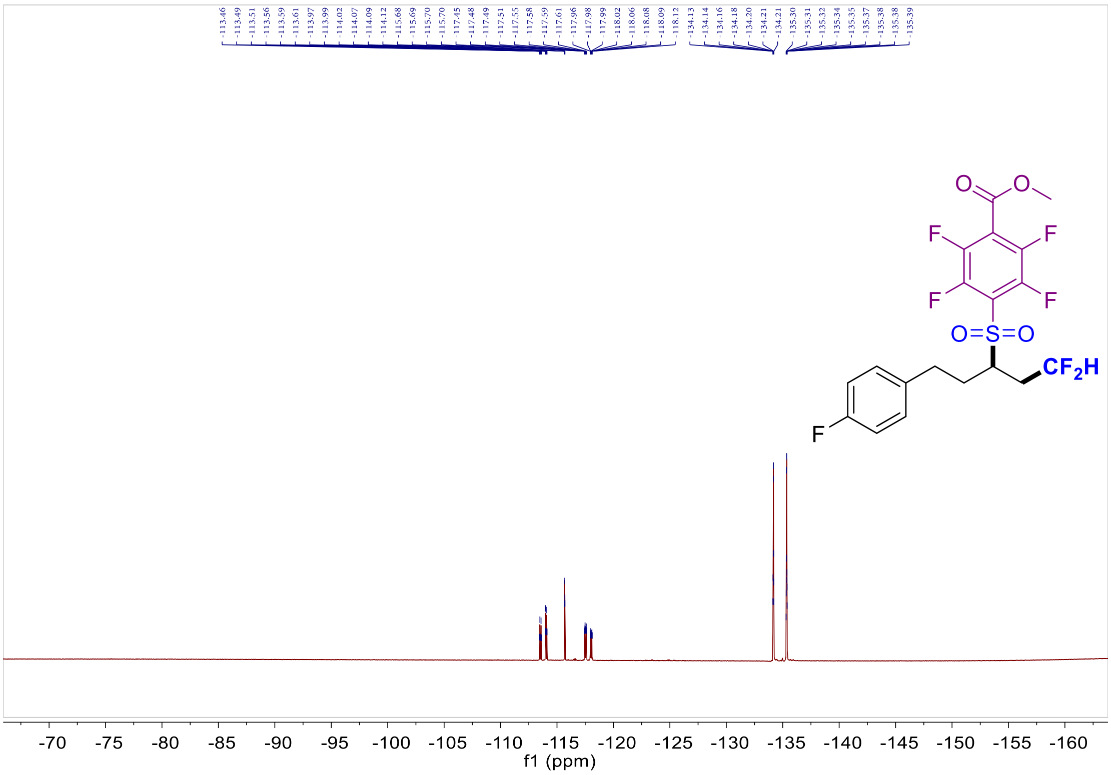

^{1}H NMR (500 MHz, CDCl$_3$) spectrum of 27

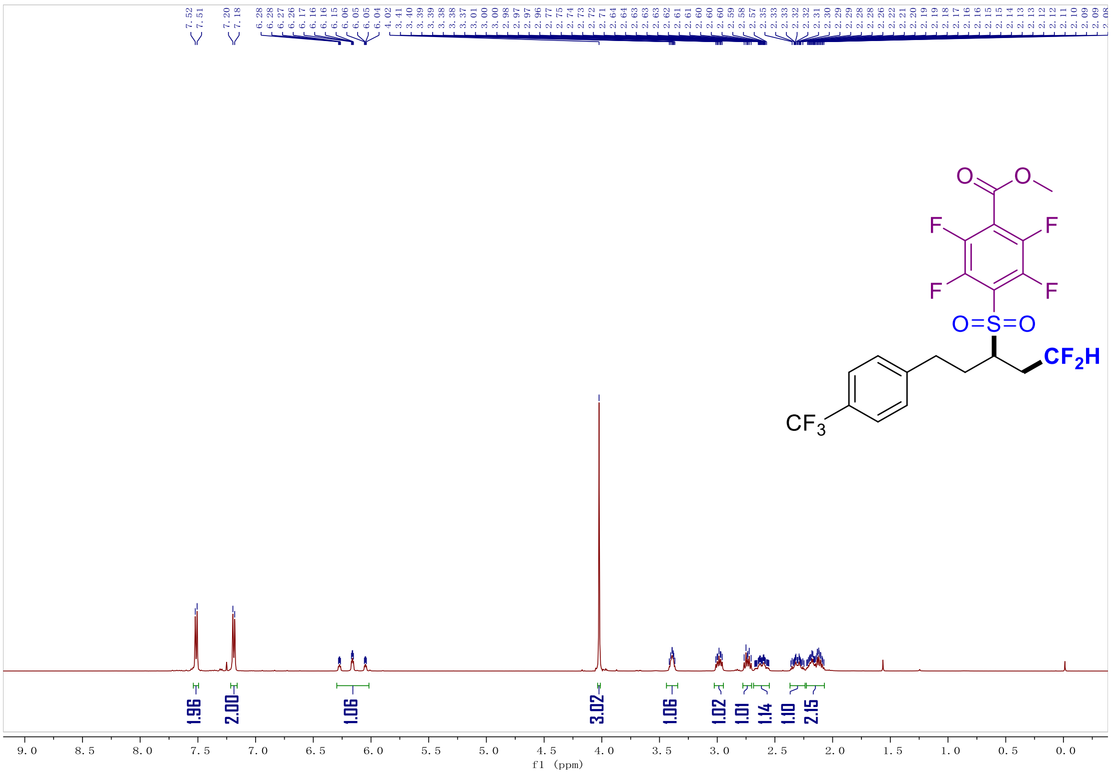

^{13}C NMR (151 MHz, CDCl$_3$) spectrum of 27

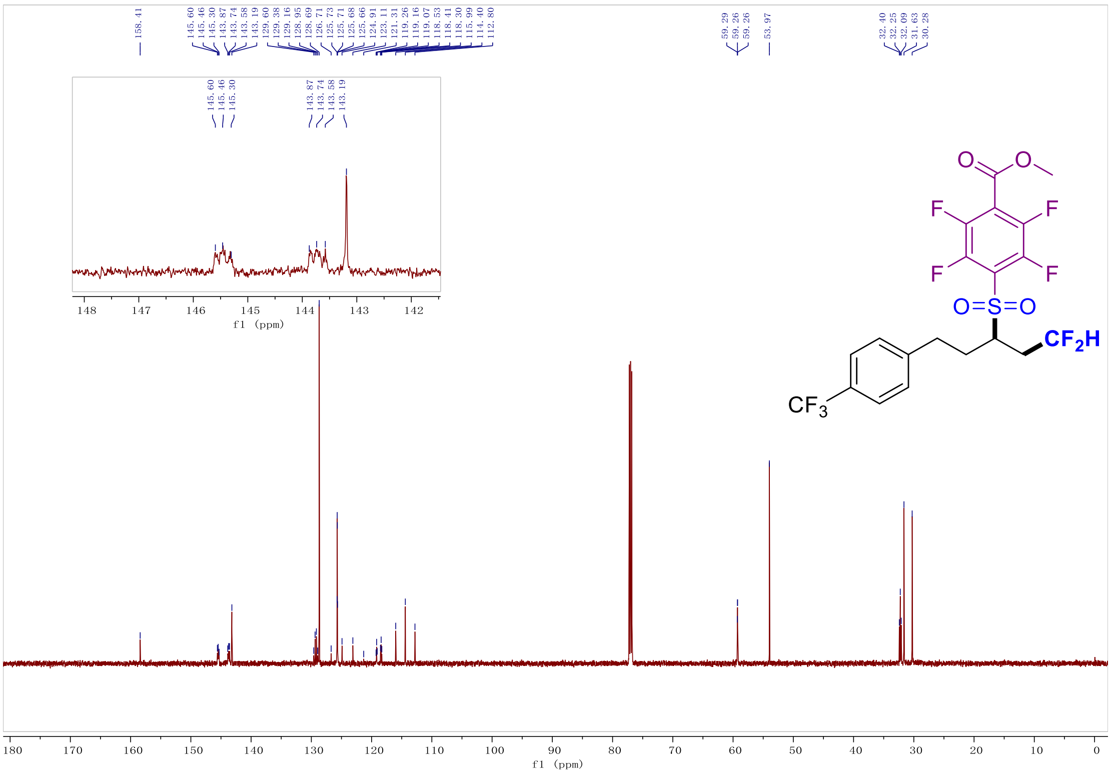

^{19}F NMR (565 MHz, CDCl$_3$) spectrum of 27

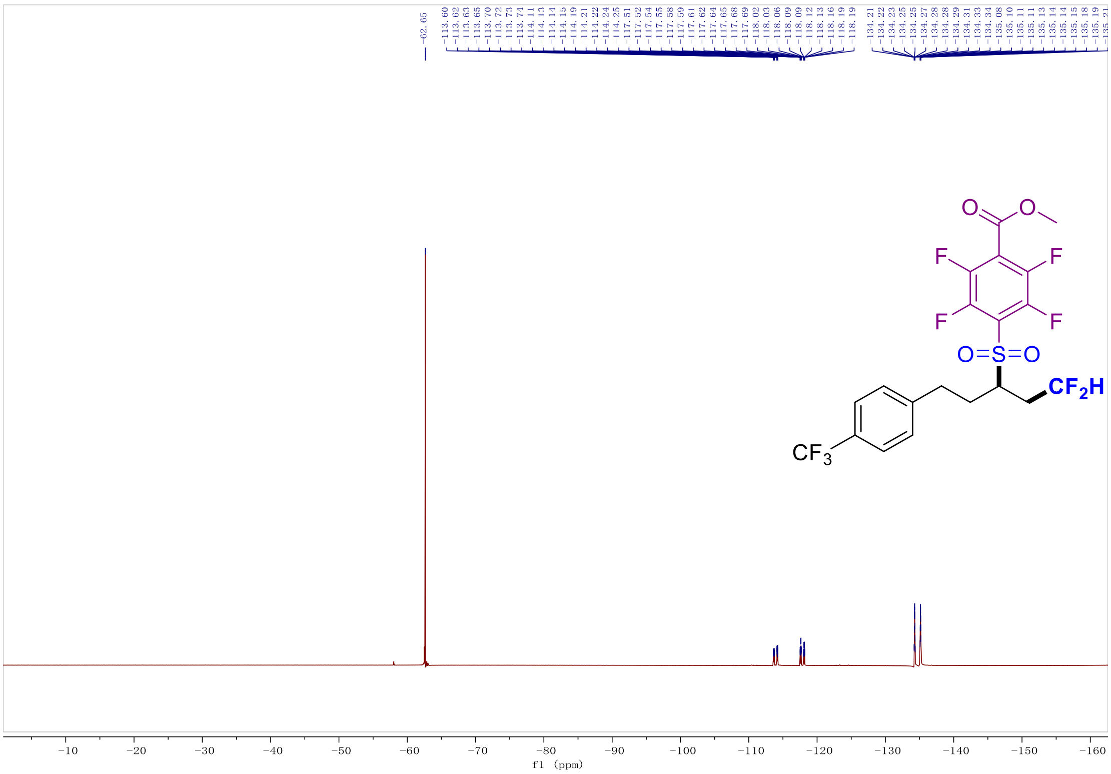

^{1}H NMR (500 MHz, $CDCl_3$) spectrum of 28

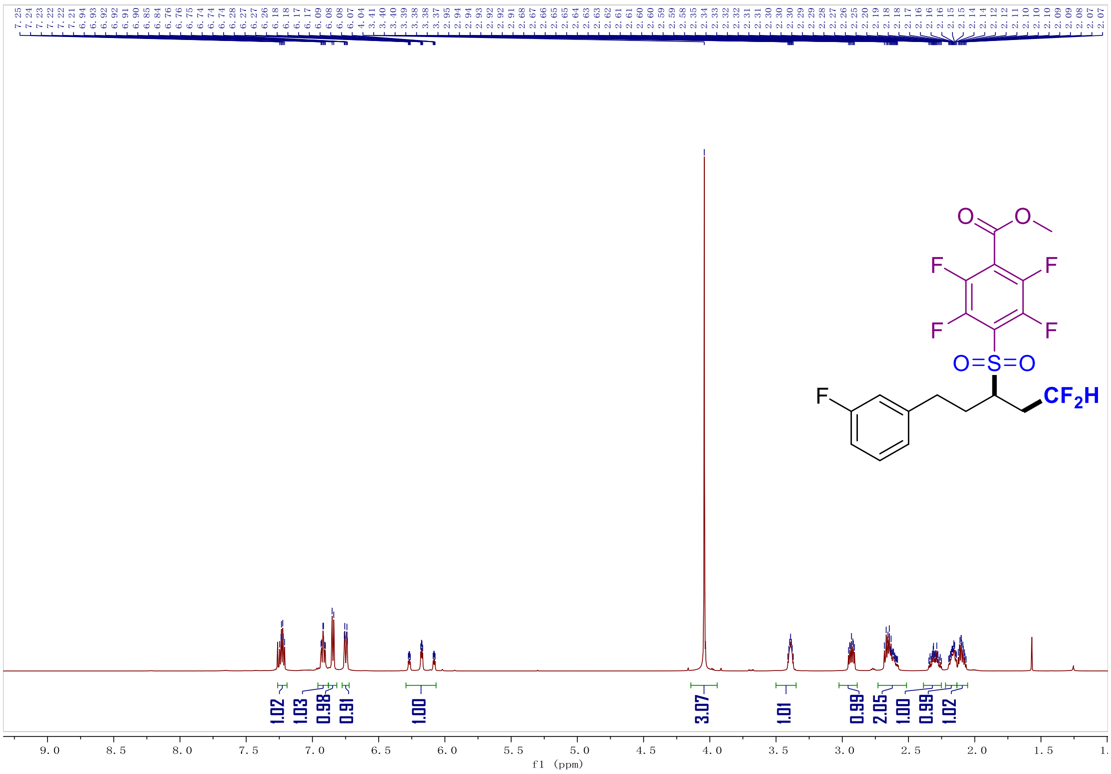

^{13}C NMR (151 MHz, $CDCl_3$) spectrum of 28

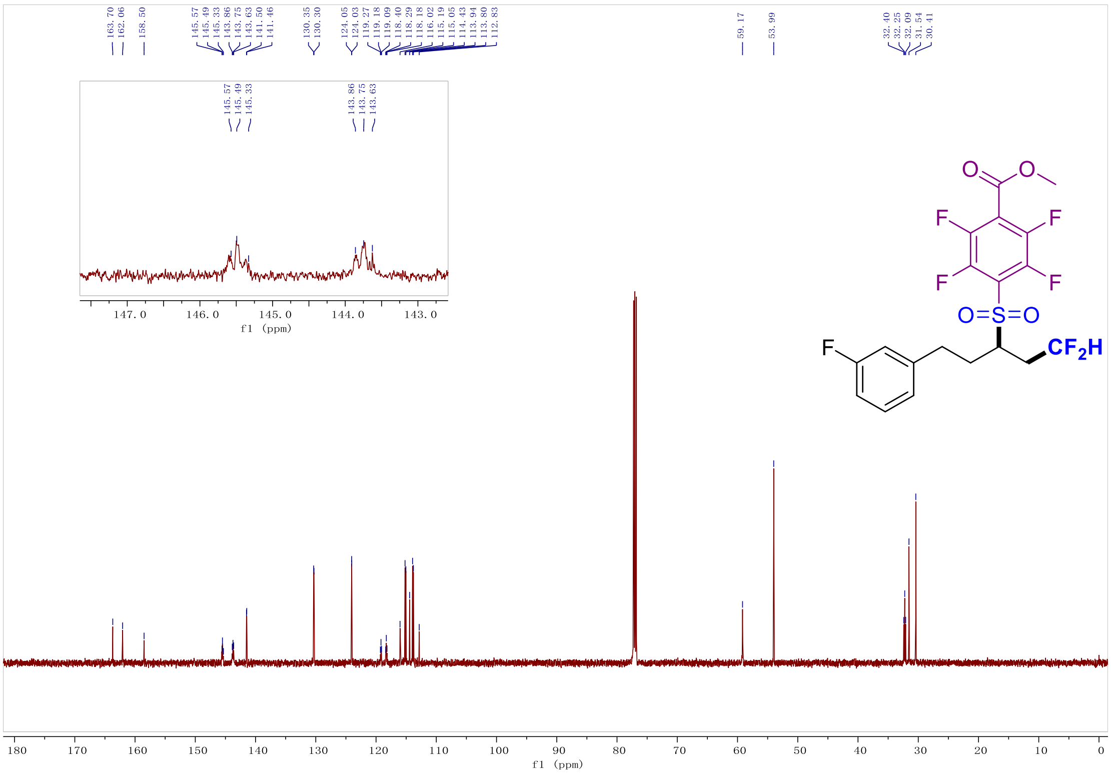

^{19}F NMR (565 MHz, CDCl$_3$) spectrum of 28

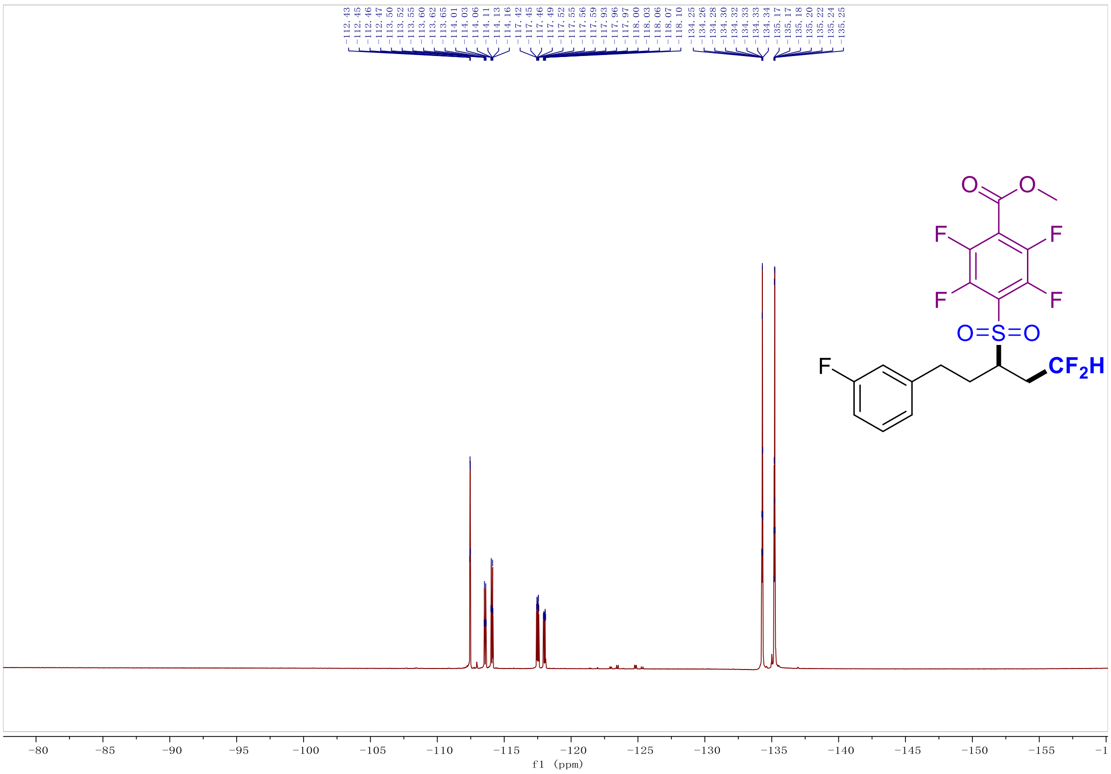

^{1}H NMR (500 MHz, CDCl$_3$) spectrum of 29

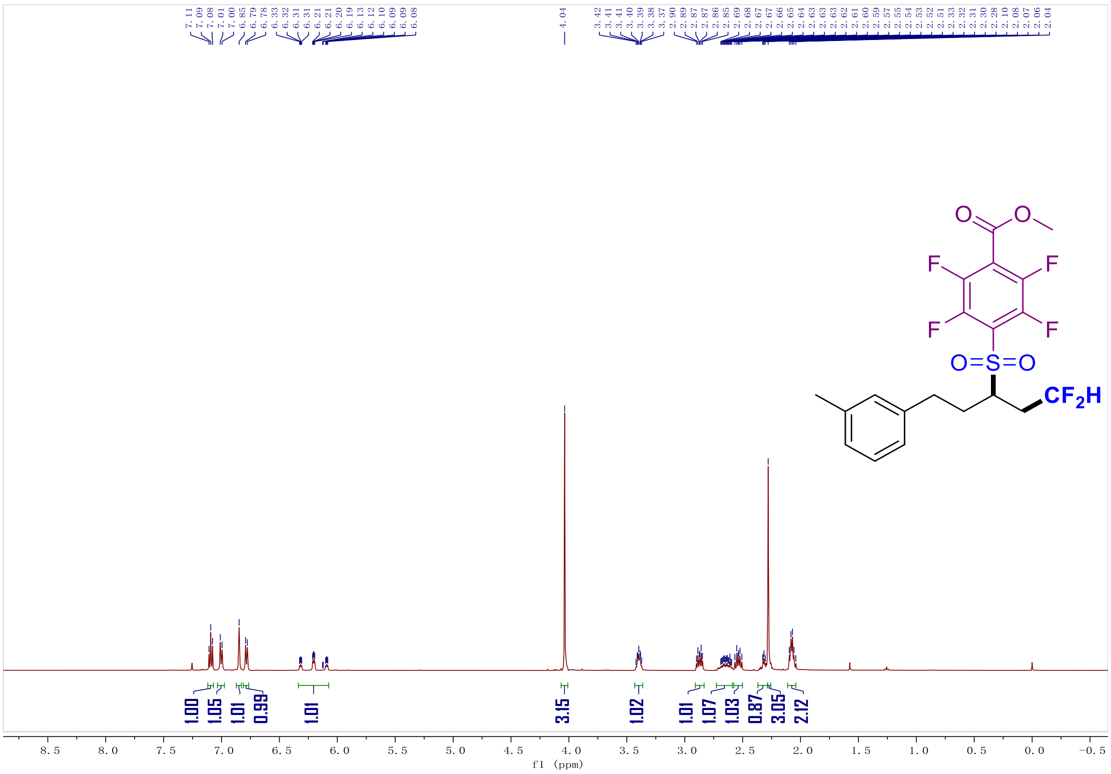

^{13}C NMR (151 MHz, $CDCl_3$) spectrum of 29

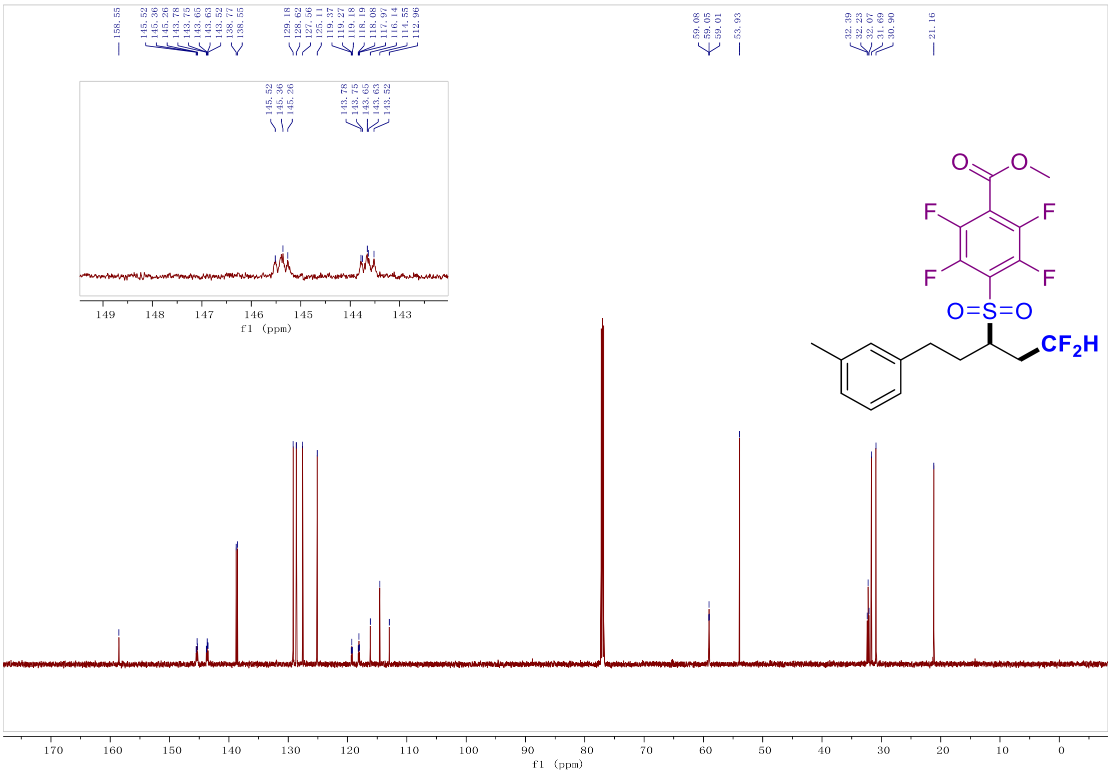

^{19}F NMR (565 MHz, $CDCl_3$) spectrum of 29

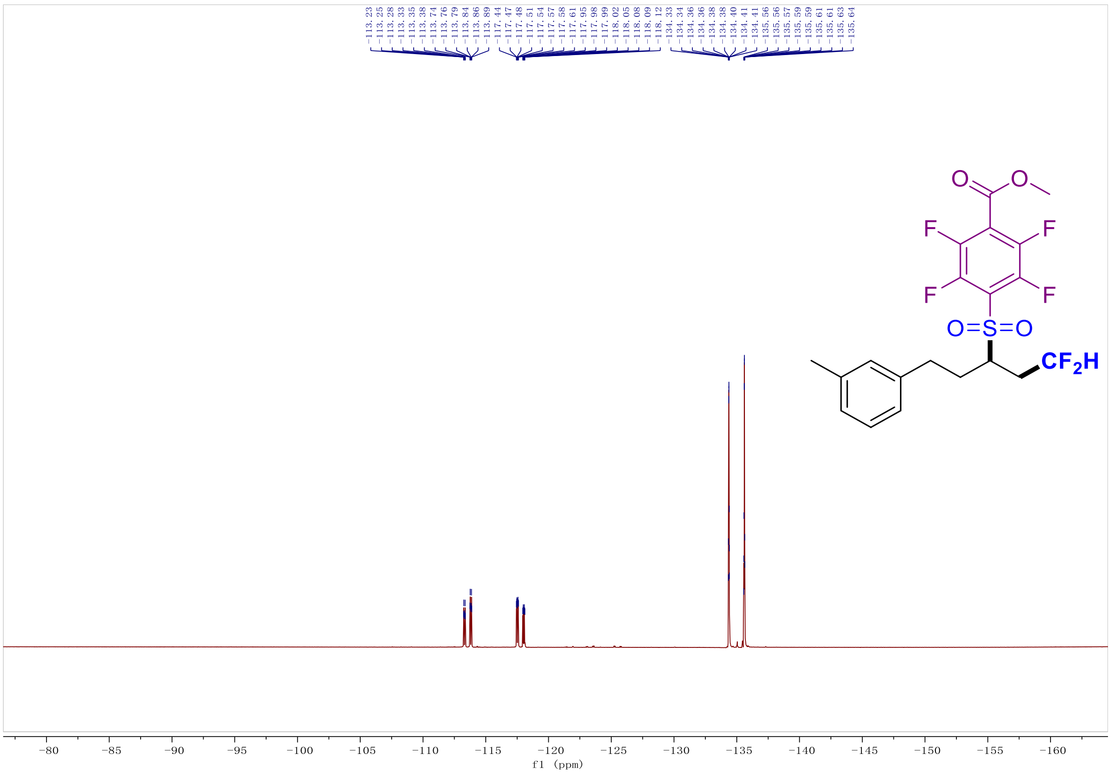

^{1}H NMR (500 MHz, $CDCl_3$) spectrum of 30

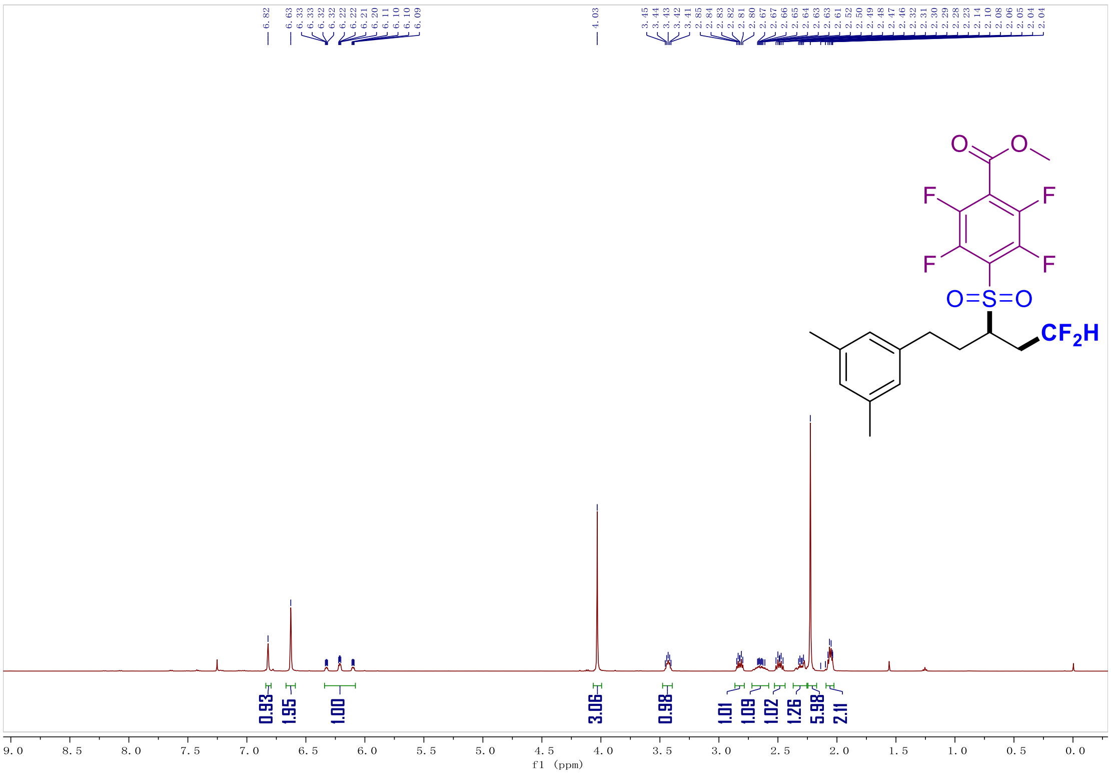

^{13}C NMR (151 MHz, $CDCl_3$) spectrum of 30

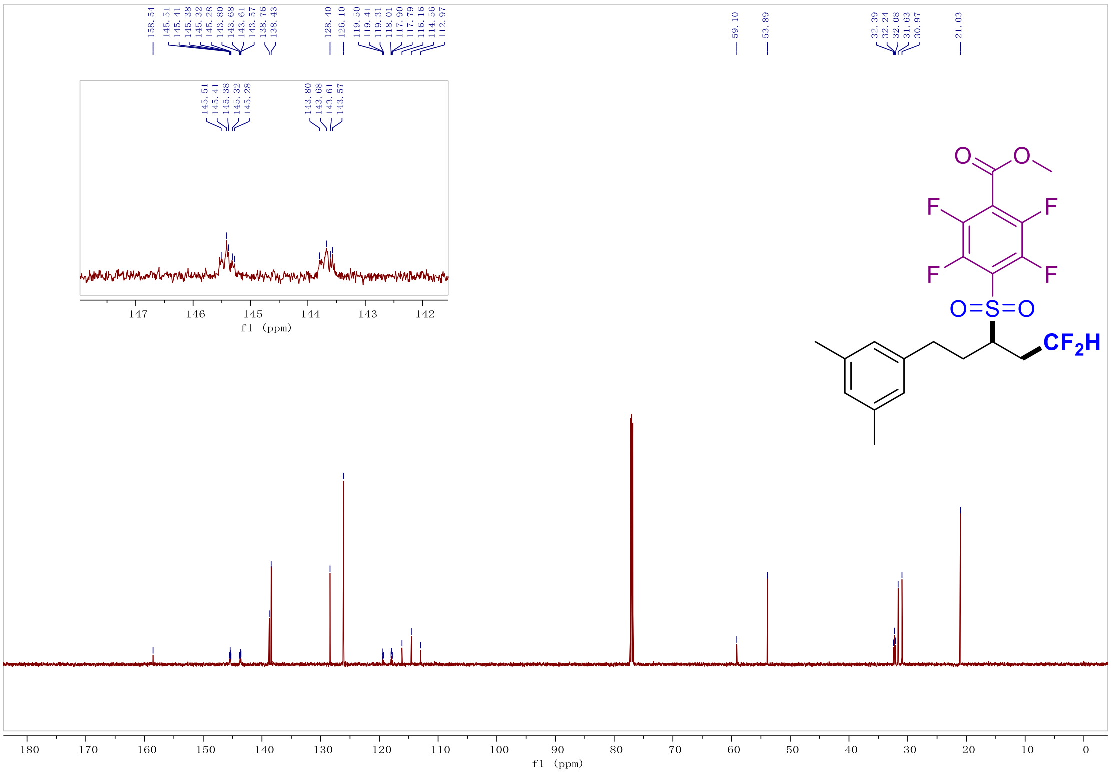

^{19}F NMR (565 MHz, CDCl3) spectrum of 30

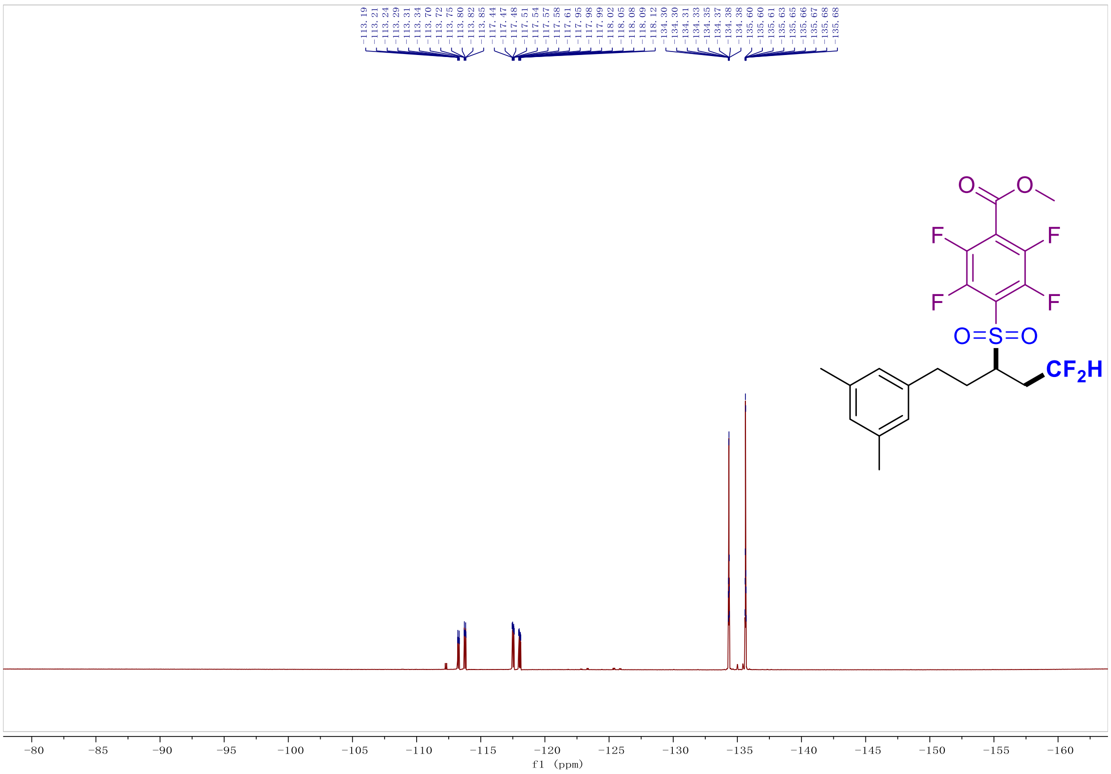

^{1}H NMR (500 MHz, CDCl3) spectrum of 31

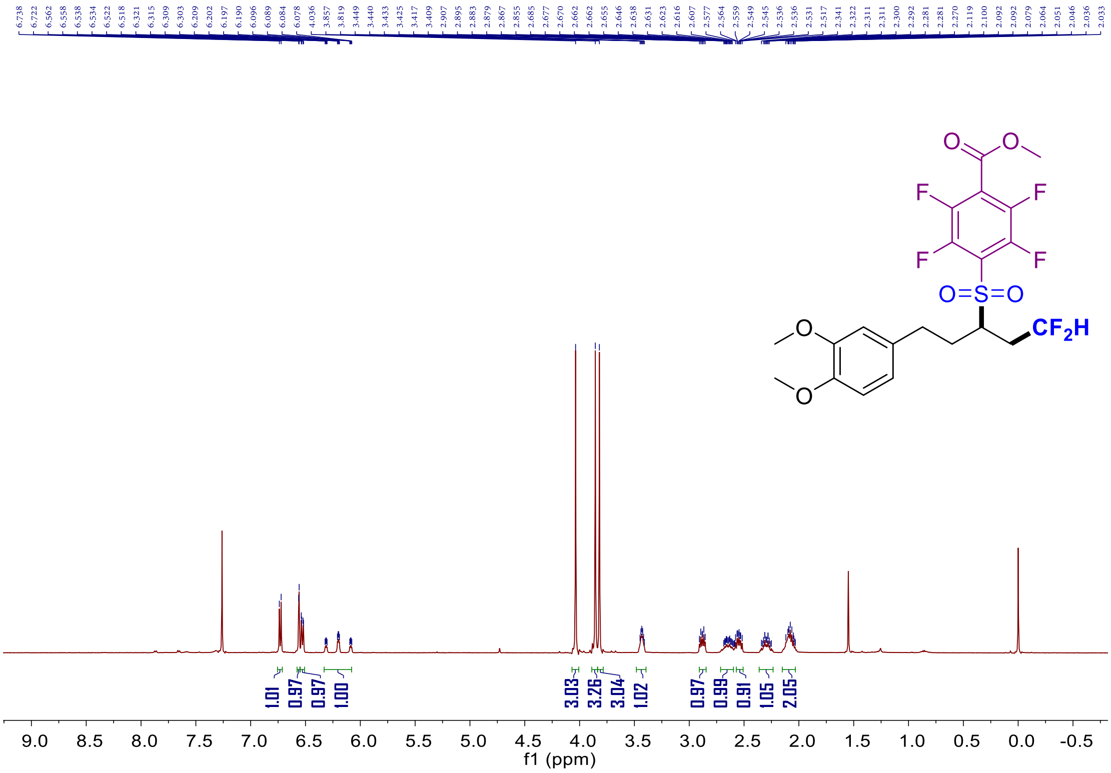

^{13}C NMR (151 MHz, $CDCl_3$) spectrum of 31

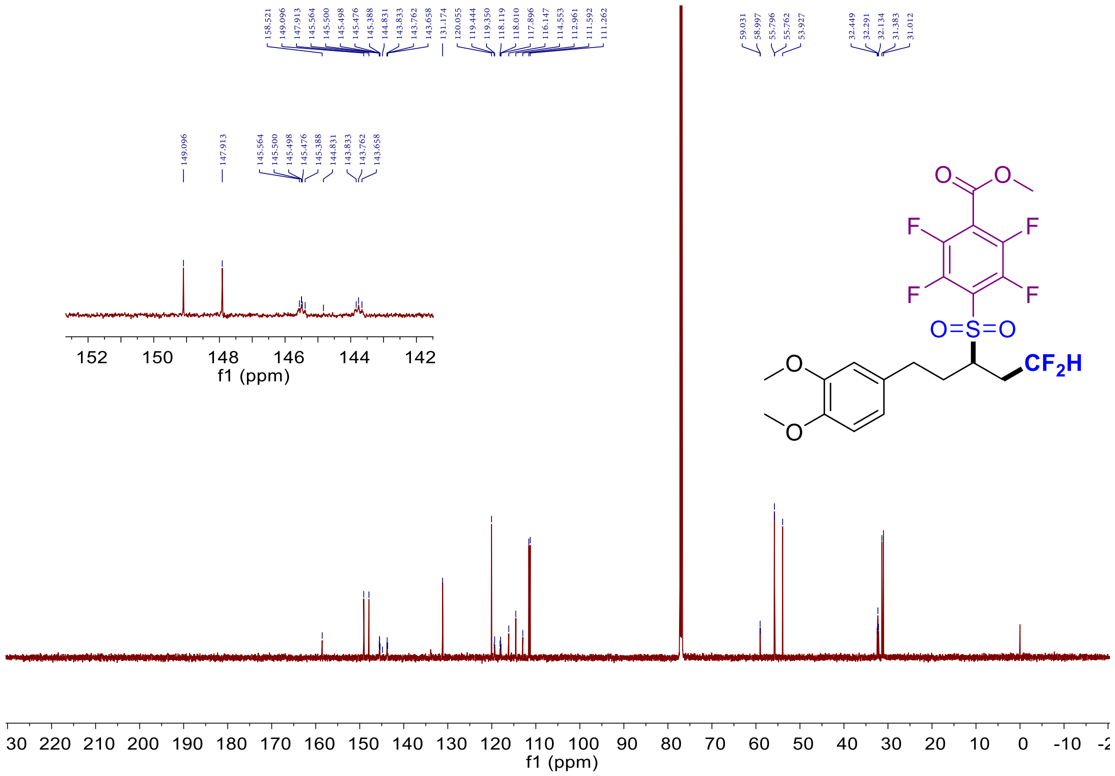

^{19}F NMR (565 MHz, $CDCl_3$) spectrum of 31

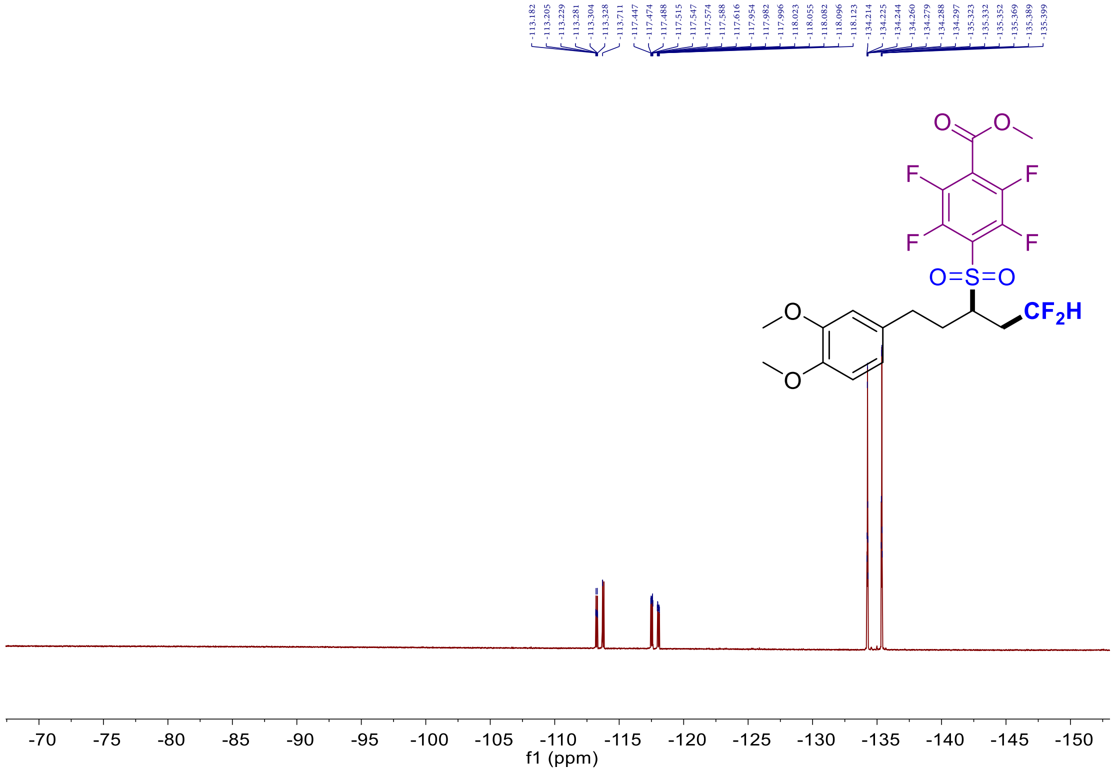

^{1}H NMR (500 MHz, CDCl$_3$) spectrum of 32

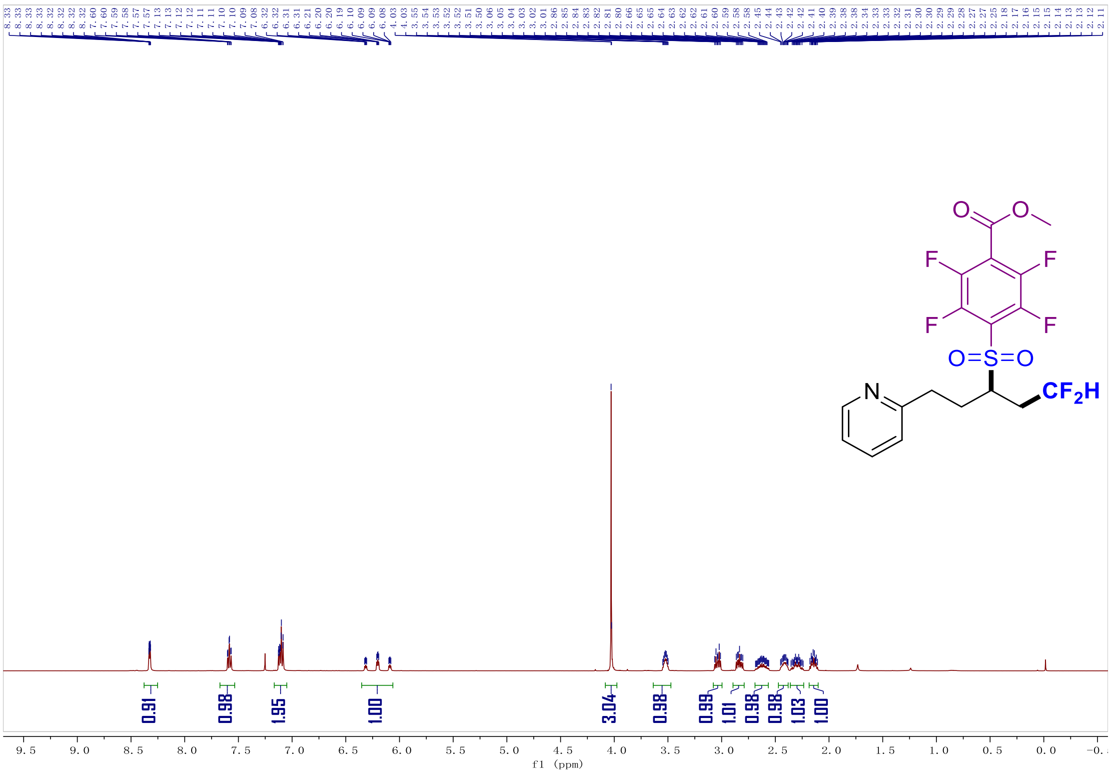

^{13}C NMR (151 MHz, CDCl$_3$) spectrum of 32

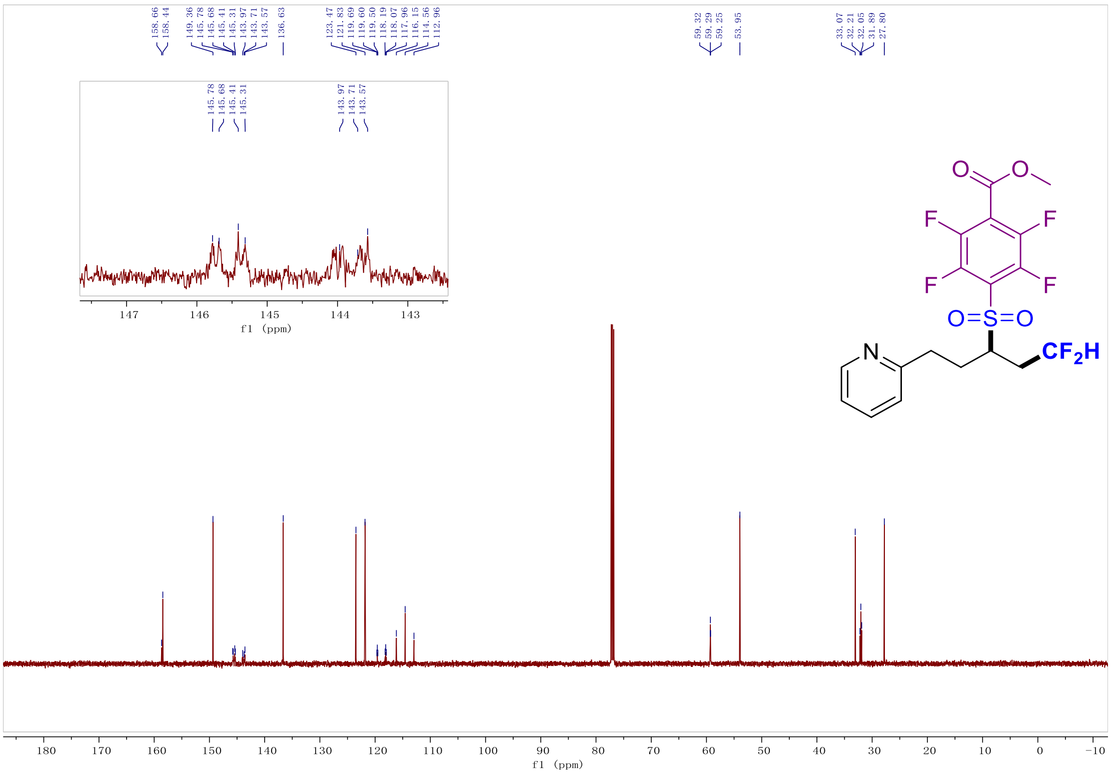

^{19}F NMR (565 MHz, $CDCl_3$) spectrum of 32

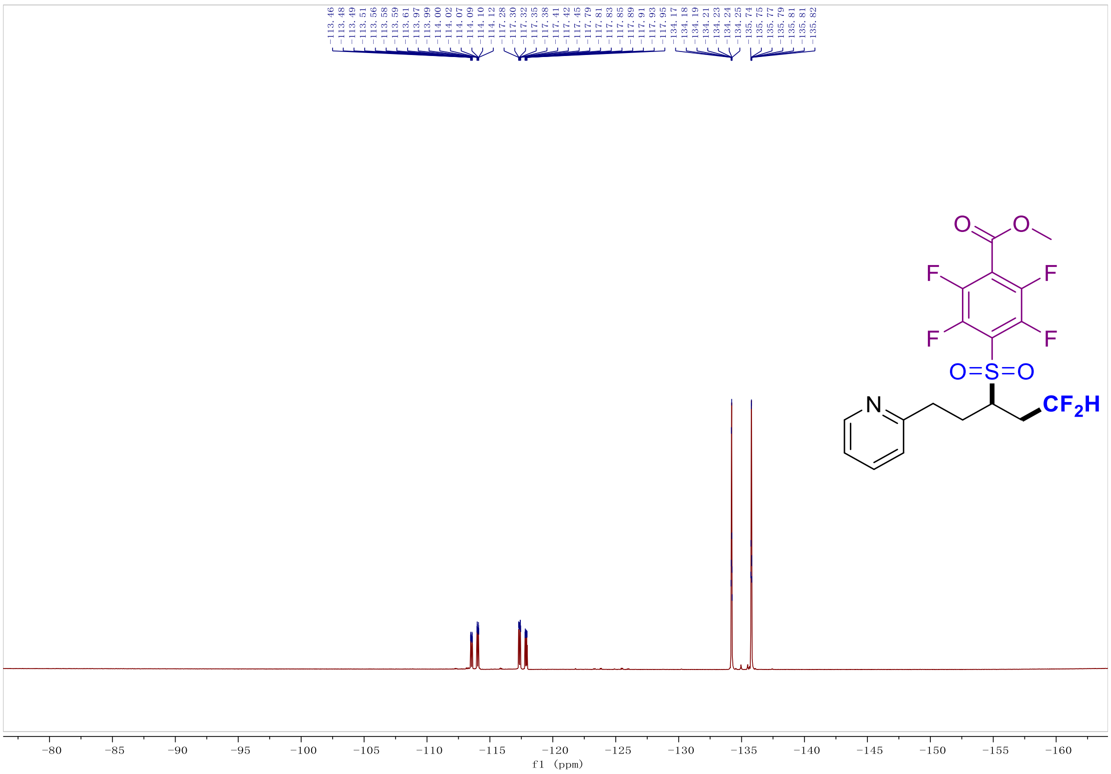

^{1}H NMR (500 MHz, $CDCl_3$) spectrum of 33

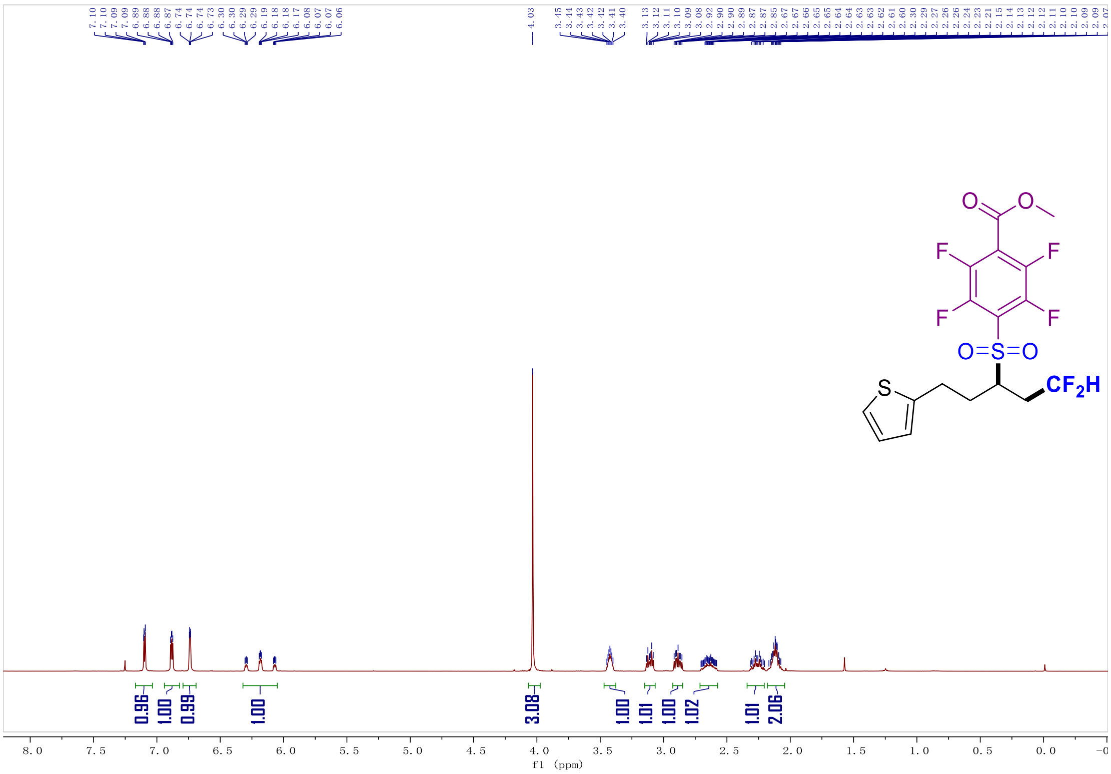

^{13}C NMR (151 MHz, $CDCl_3$) spectrum of 33

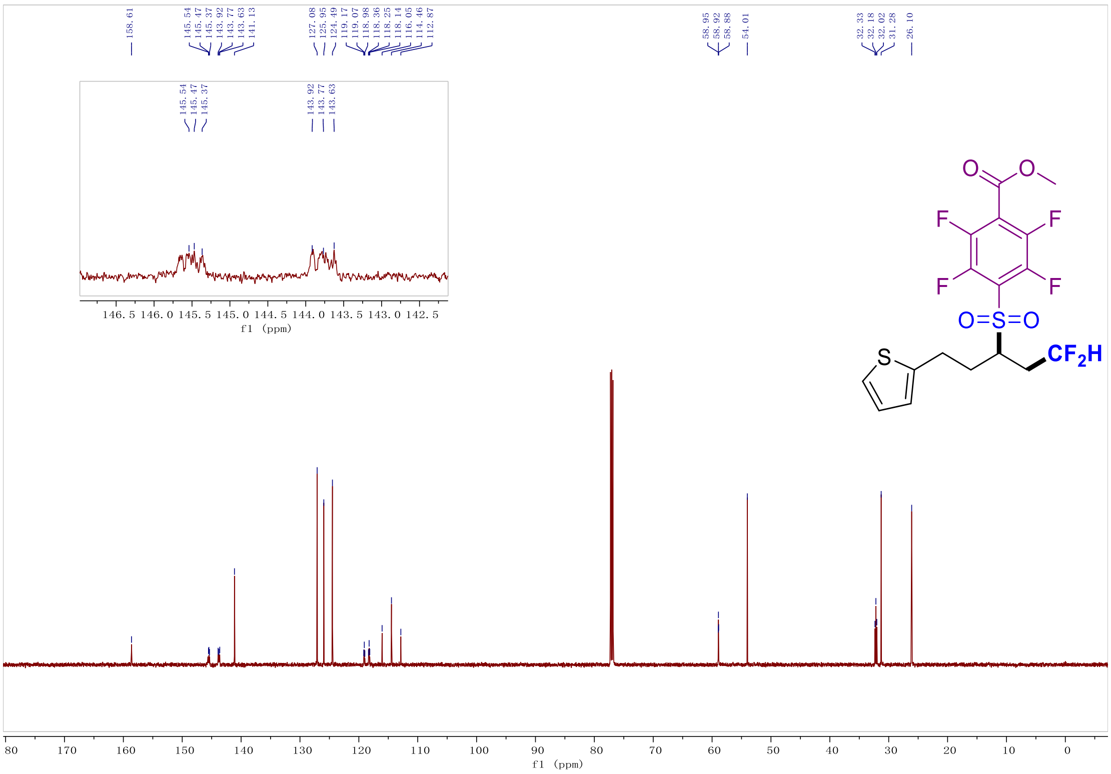

^{19}F NMR (565 MHz, $CDCl_3$) spectrum of 33

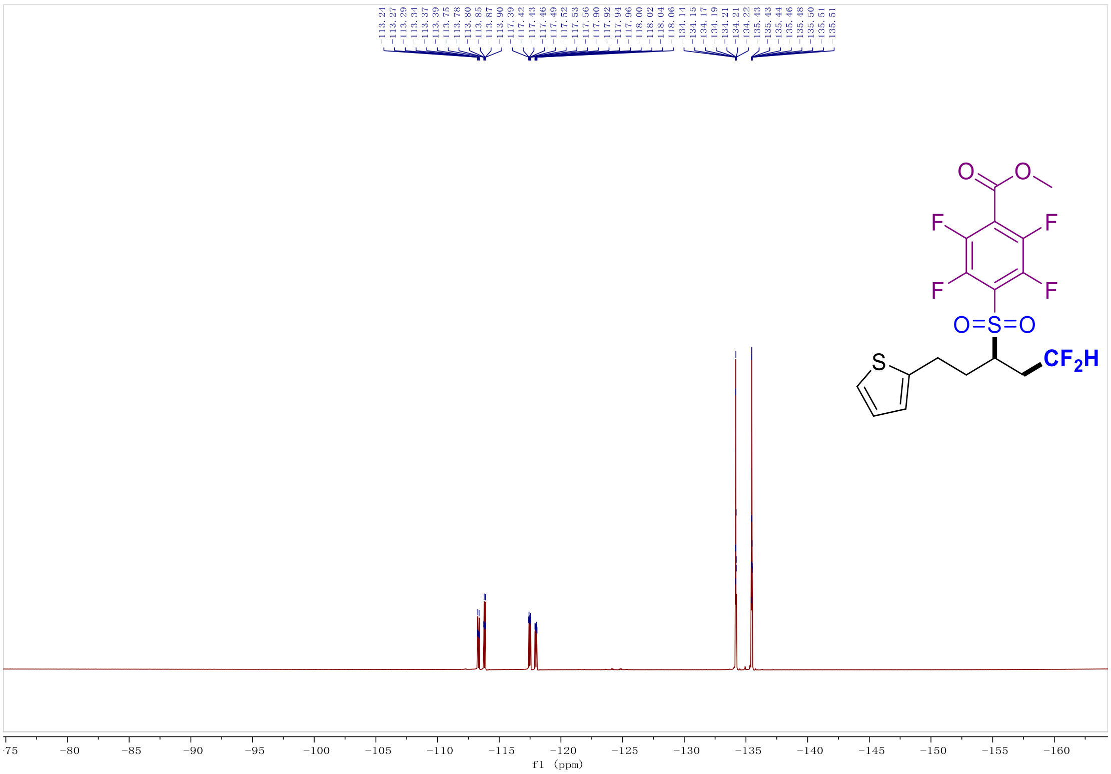

^{1}H NMR (500 MHz, CDCl$_3$) spectrum of 34

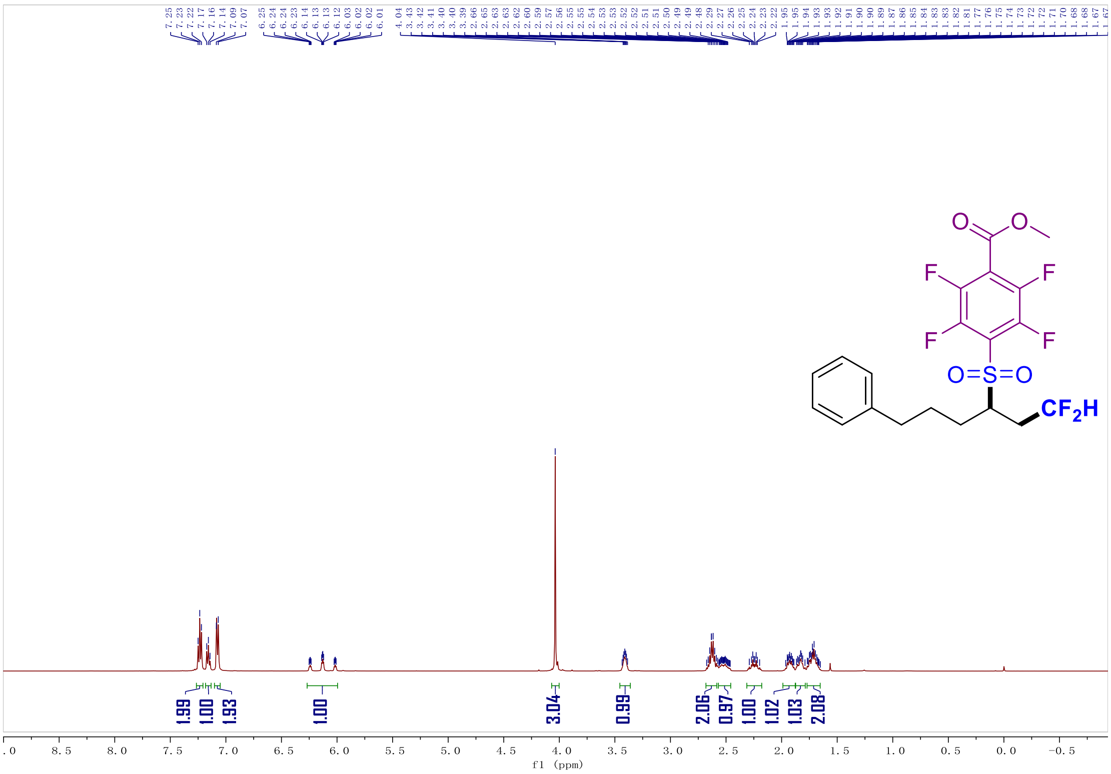

^{13}C NMR (151 MHz, CDCl$_3$) spectrum of 34

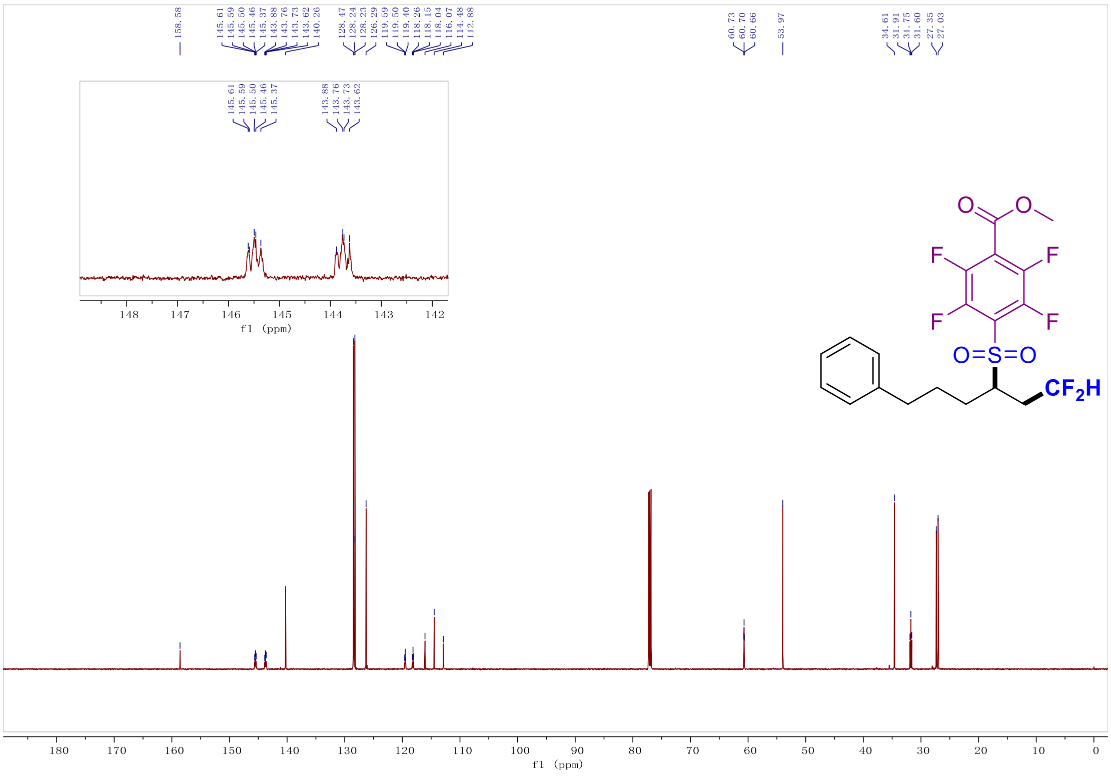

^{19}F NMR (565 MHz, $CDCl_3$) spectrum of 34

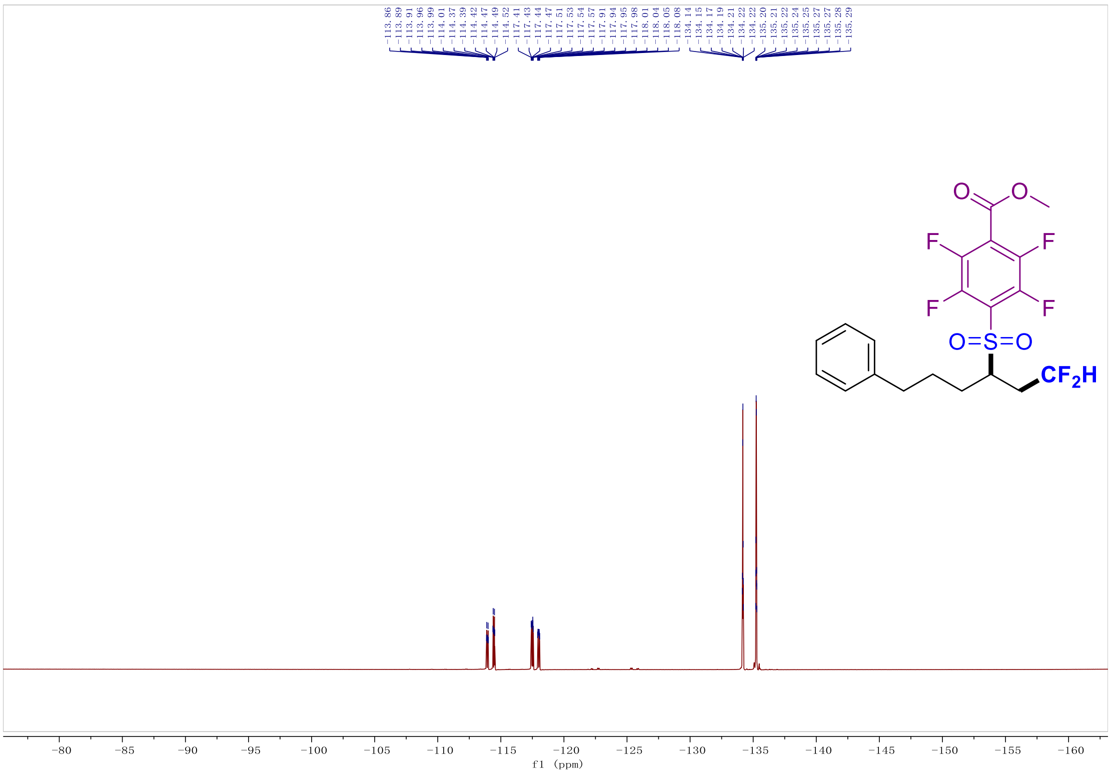

^{1}H NMR (500 MHz, $CDCl_3$) spectrum of 35

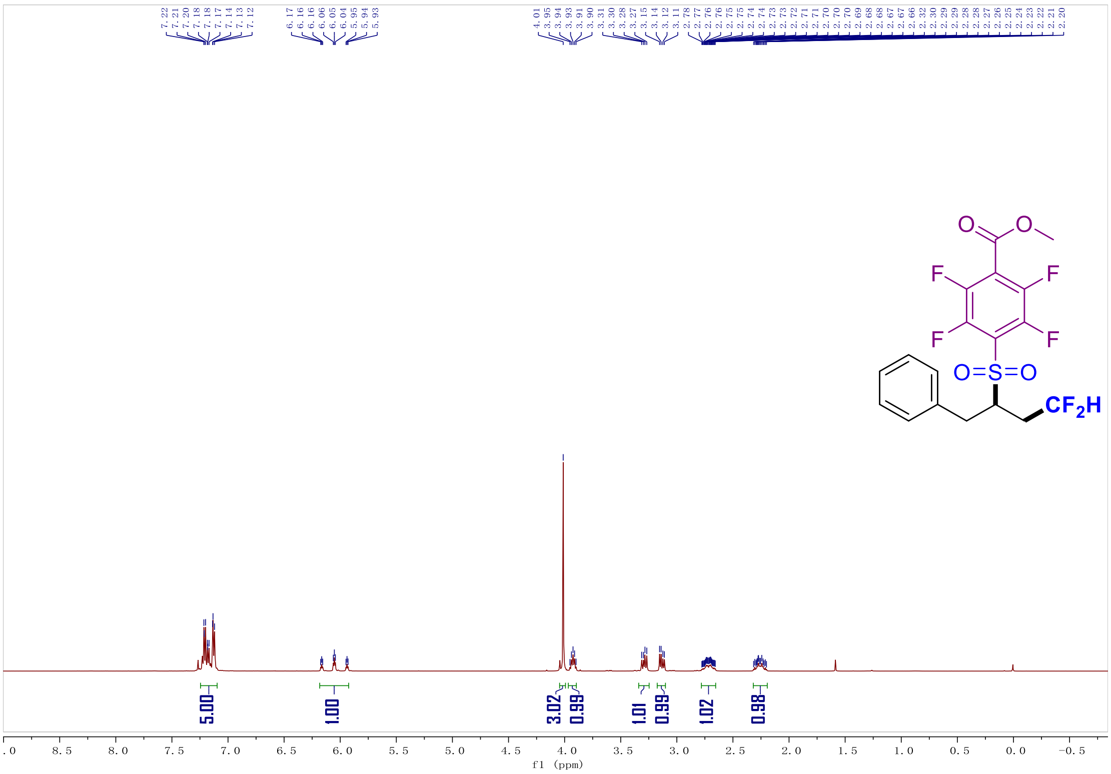

^{13}C NMR (151 MHz, CDCl$_3$) spectrum of 35

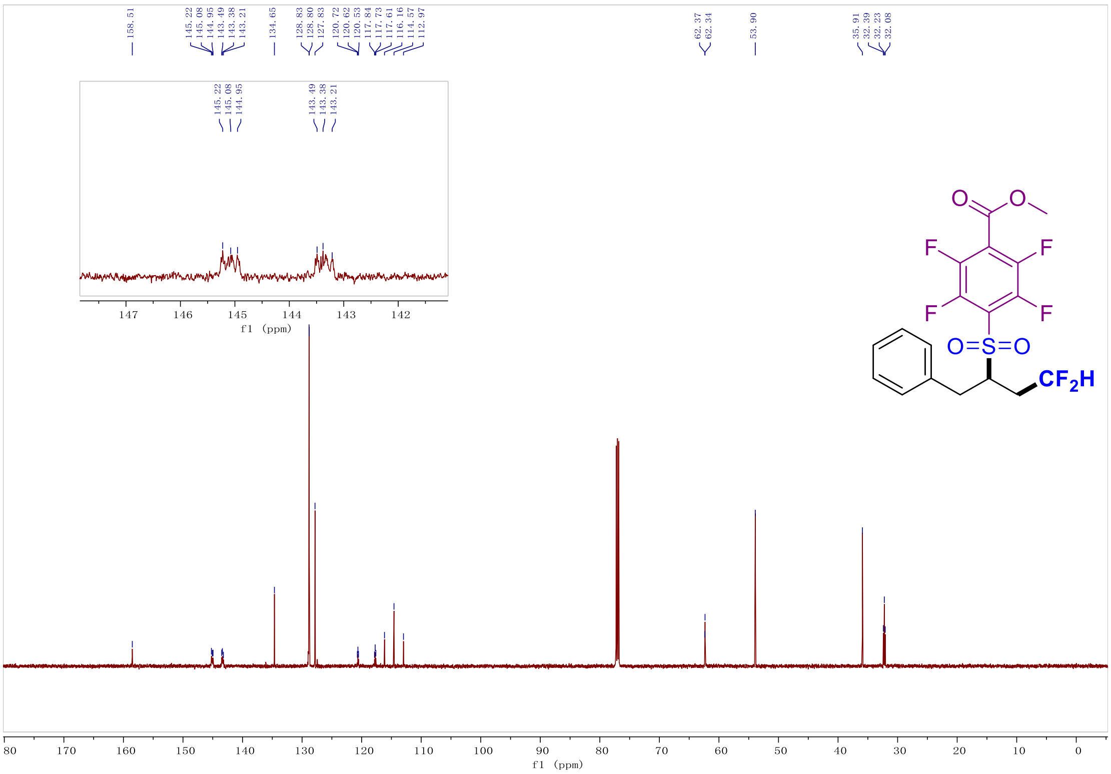

^{19}F NMR (565 MHz, CDCl$_3$) spectrum of 35

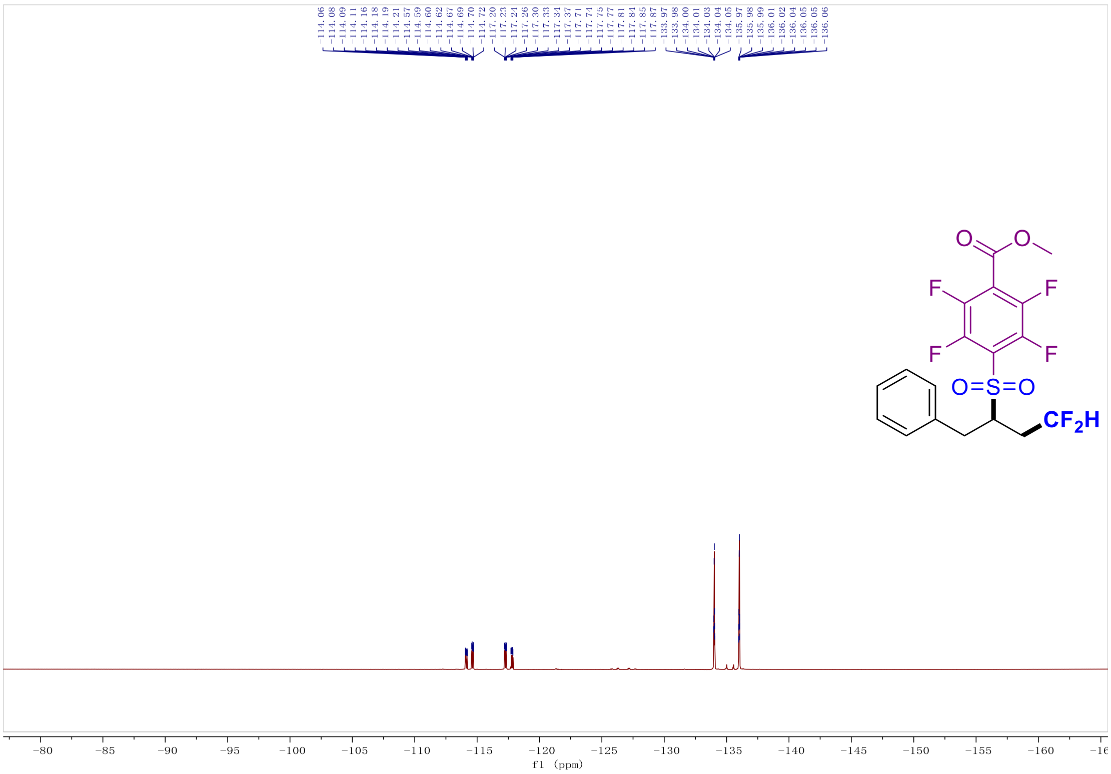

^{1}H NMR (500 MHz, $CDCl_3$) spectrum of 36

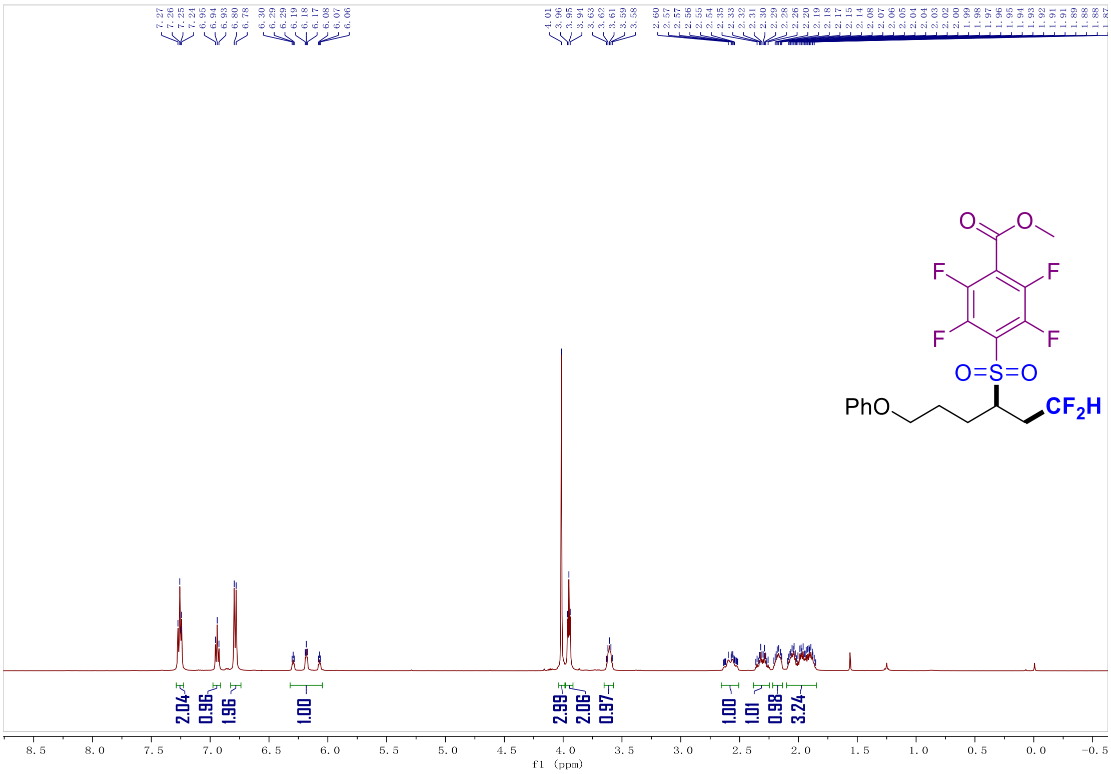

^{13}C NMR (151 MHz, $CDCl_3$) spectrum of 36

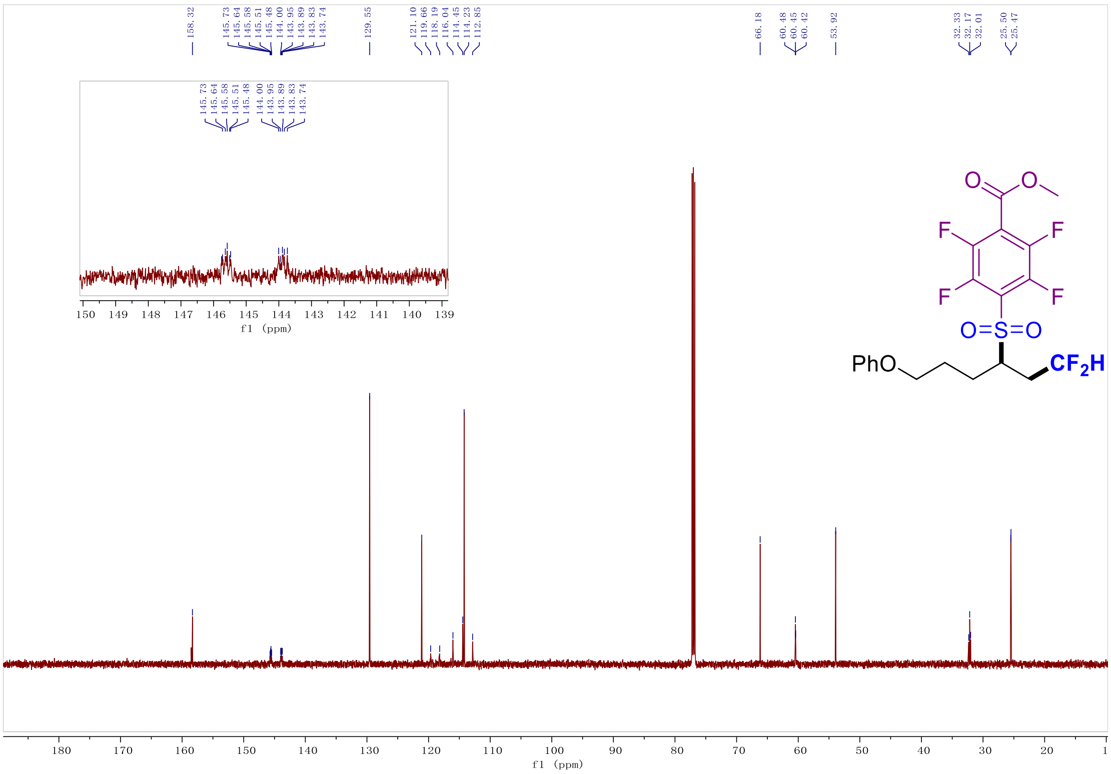

^{19}F NMR (565 MHz, $CDCl_3$) spectrum of 36

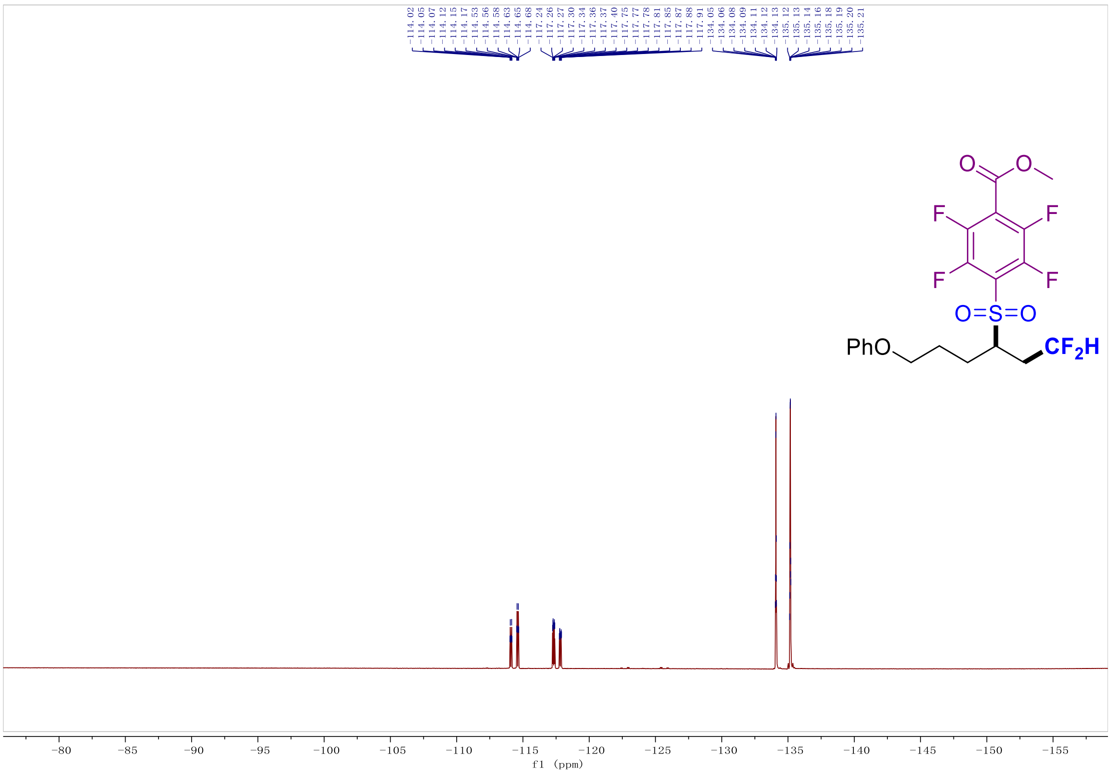

^{1}H NMR (500 MHz, $CDCl_3$) spectrum of 37

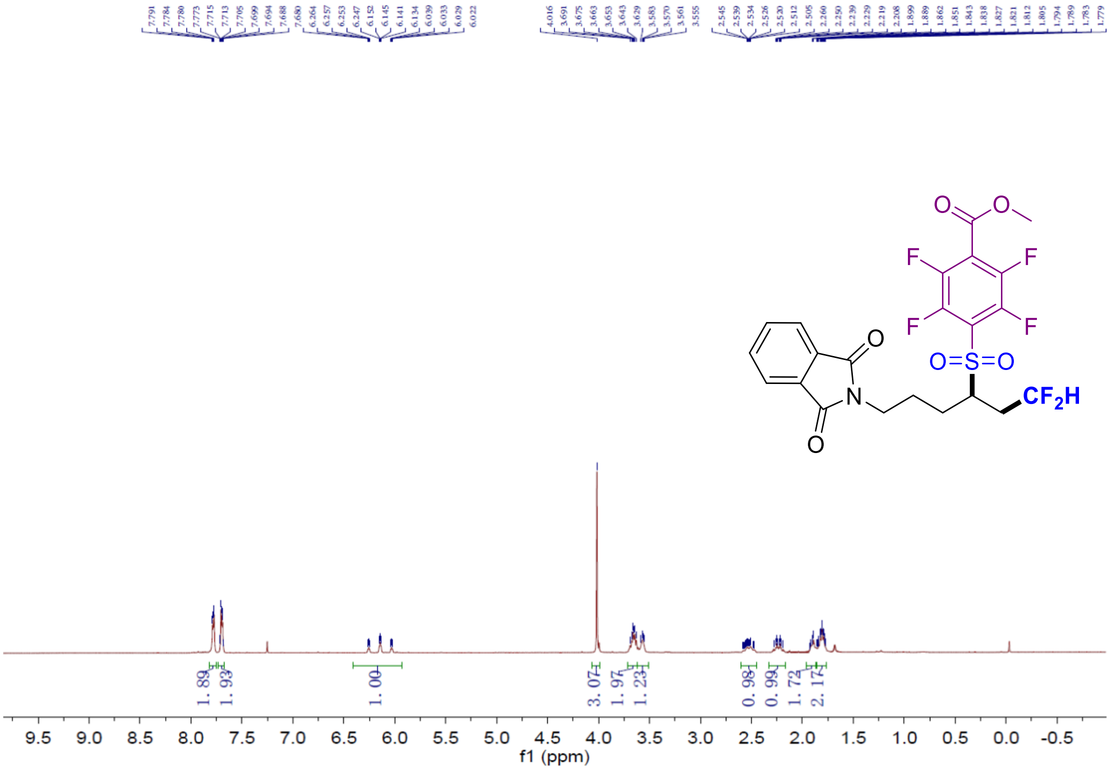

^{13}C NMR (151 MHz, $CDCl_3$) spectrum of 37

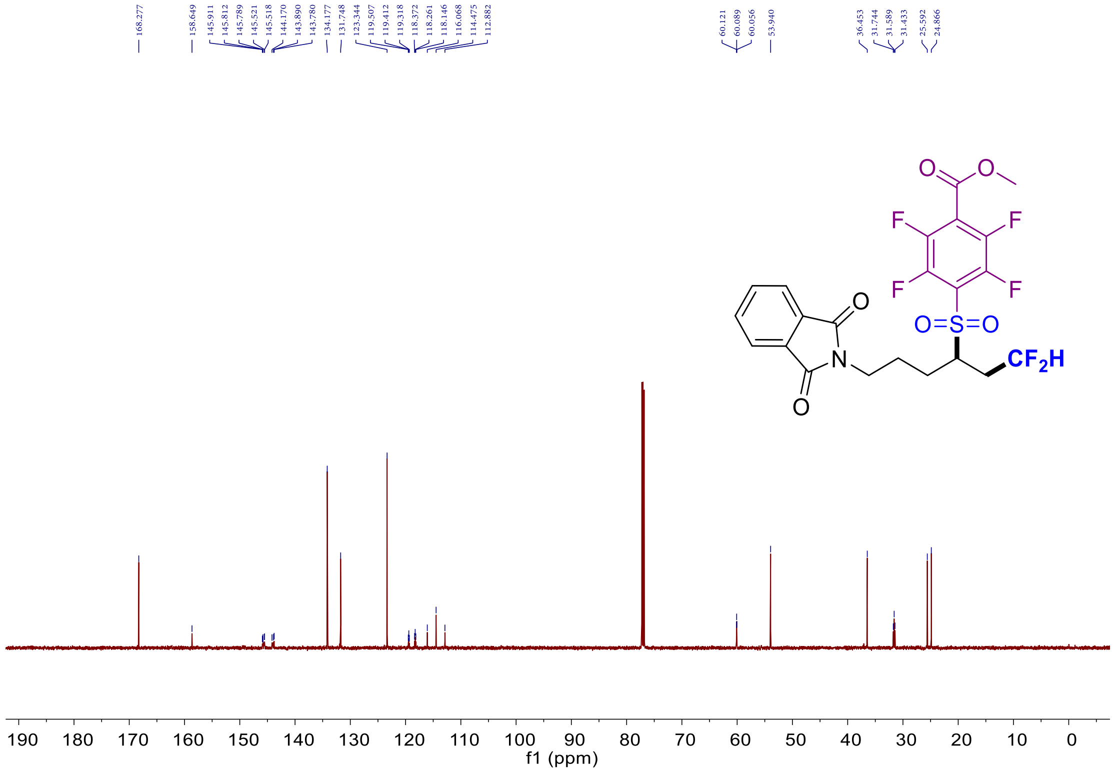

^{19}F NMR (565 MHz, $CDCl_3$) spectrum of 37

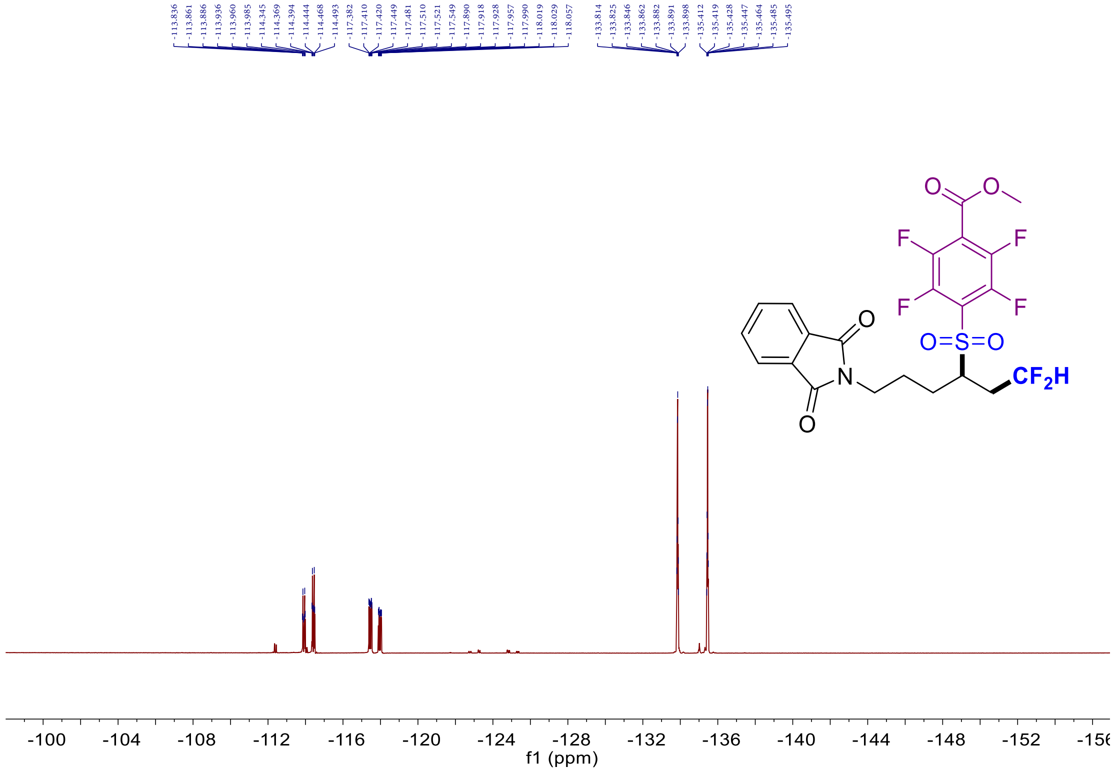

^{1}H NMR (500 MHz, $CDCl_3$) spectrum of 38

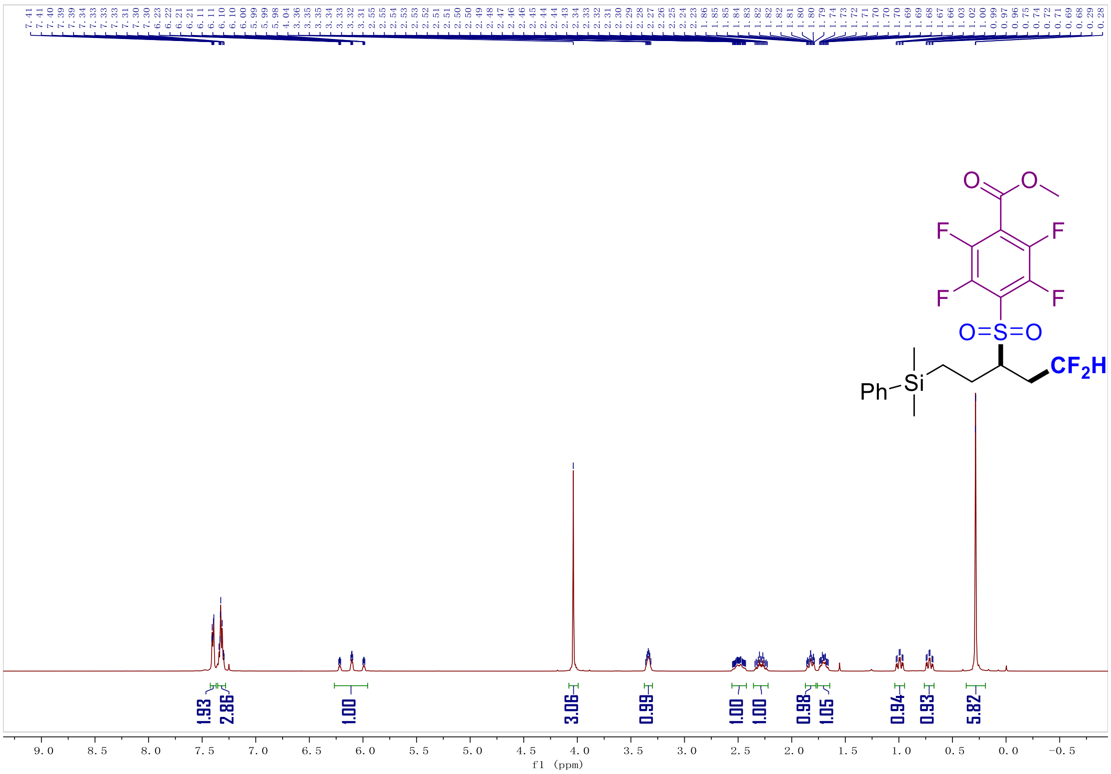

^{13}C NMR (151 MHz, $CDCl_3$) spectrum of 38

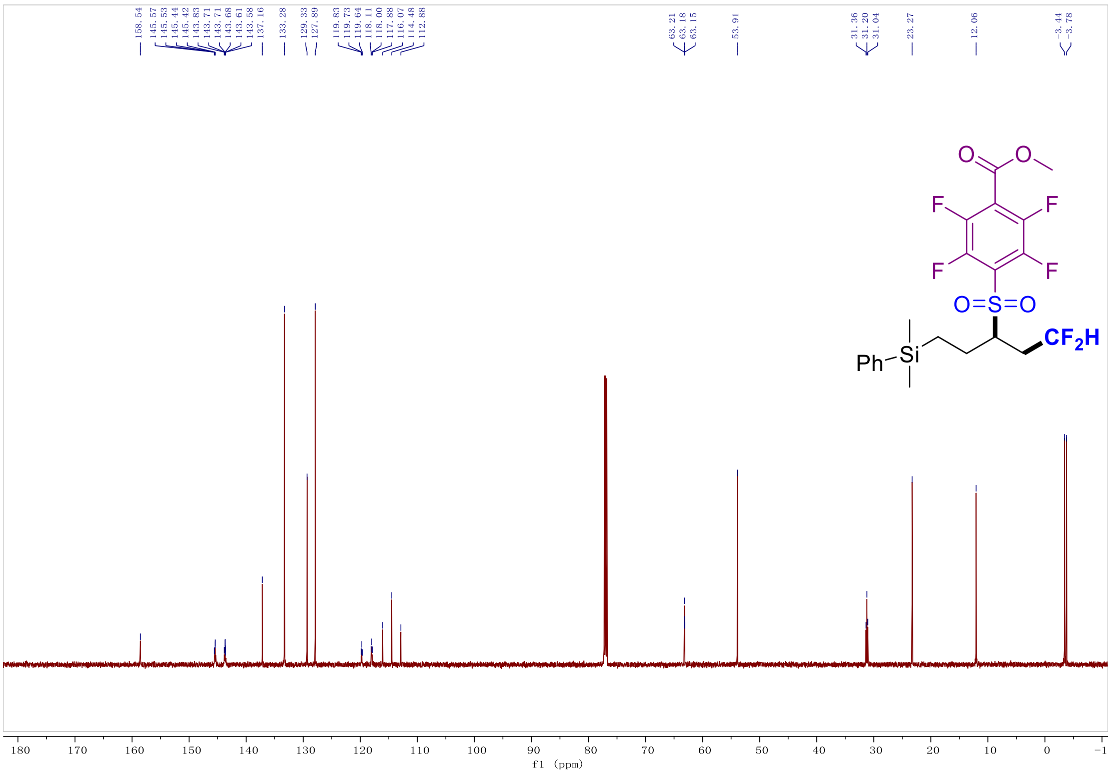

^{19}F NMR (565 MHz, $CDCl_3$) spectrum of 38

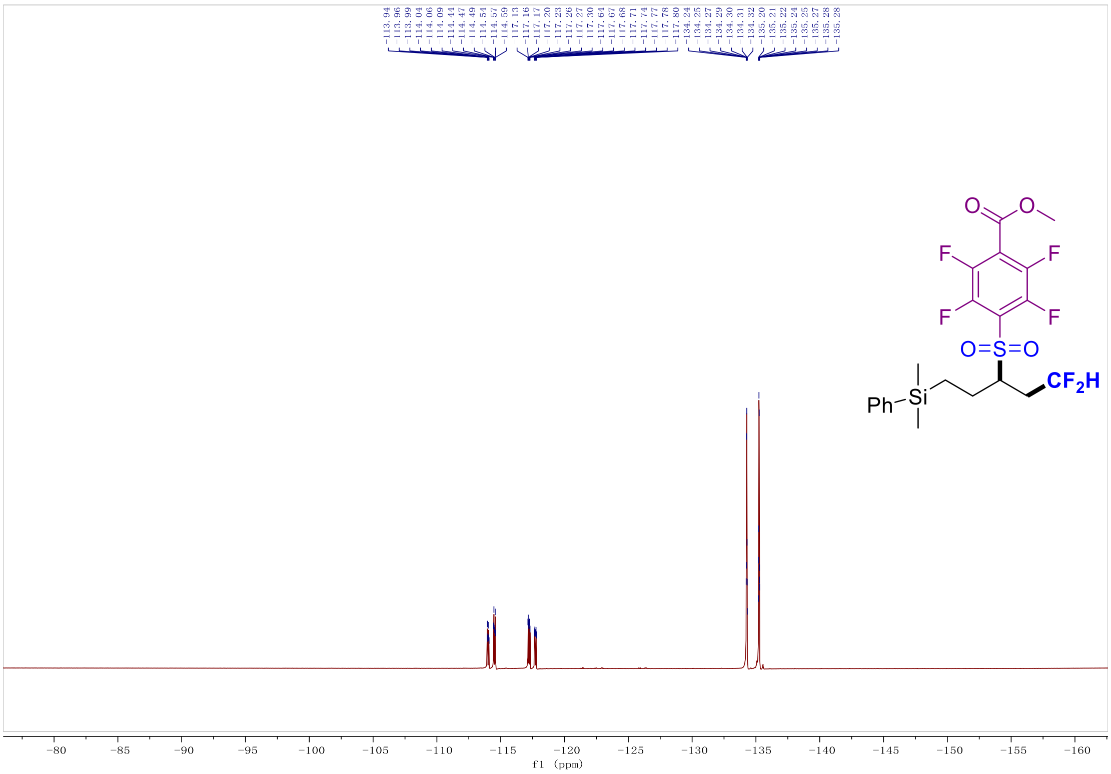

^{1}H NMR (500 MHz, $CDCl_3$) spectrum of 39

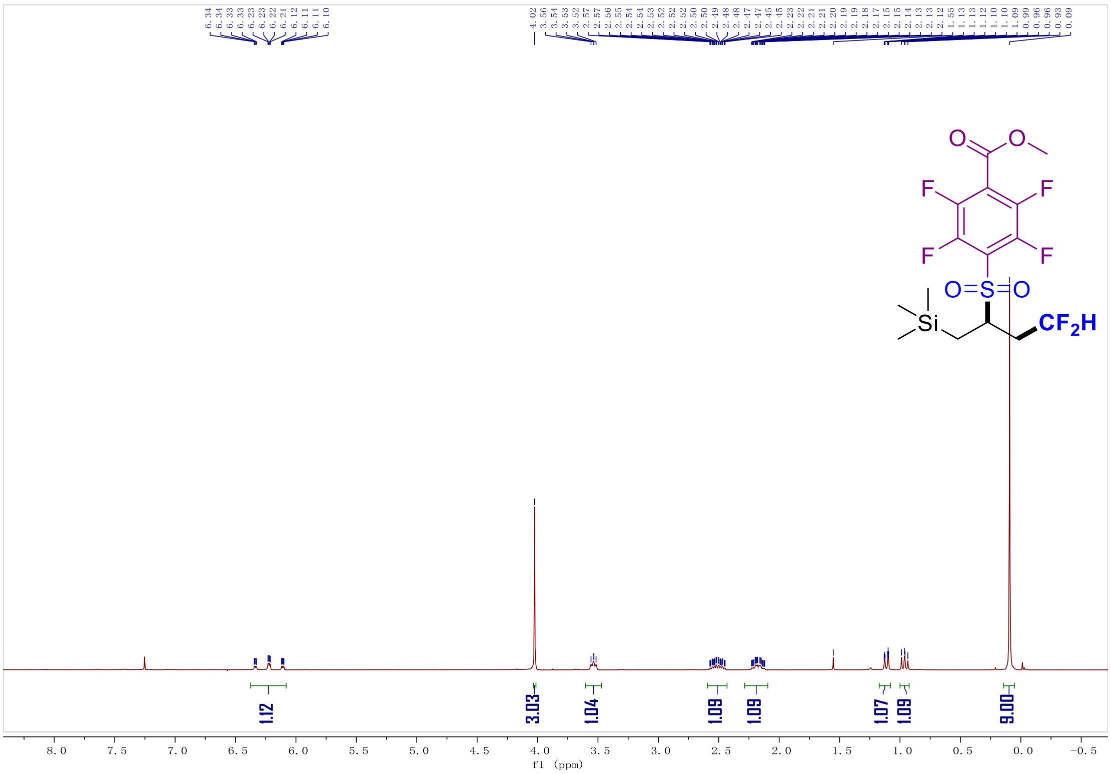

^{13}C NMR (151 MHz, $CDCl_3$) spectrum of 39

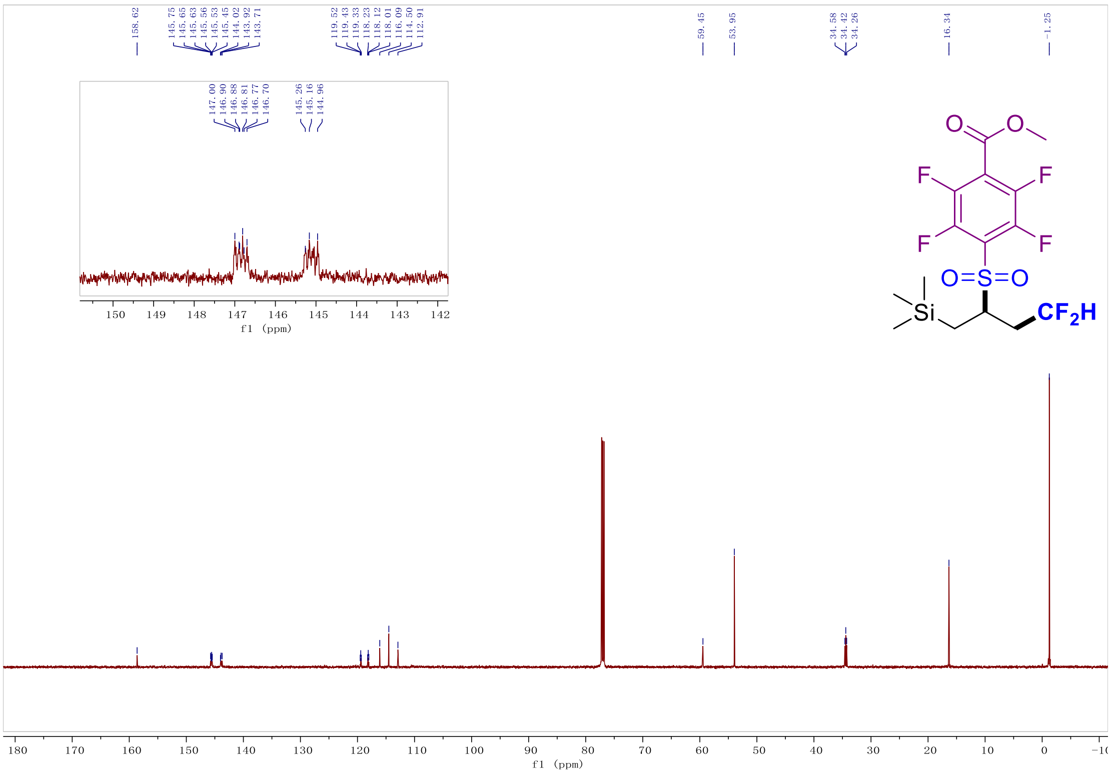

^{19}F NMR (565 MHz, $CDCl_3$) spectrum of 39

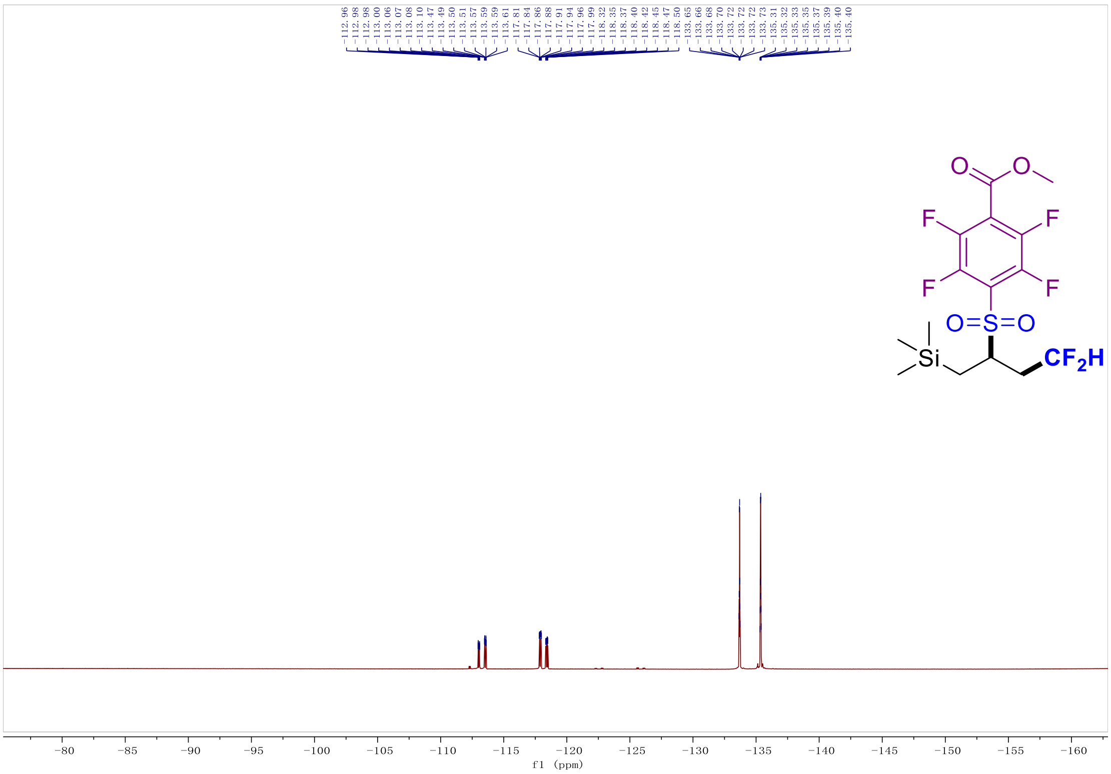

^{1}H NMR (500 MHz, $CDCl_3$) spectrum of 40

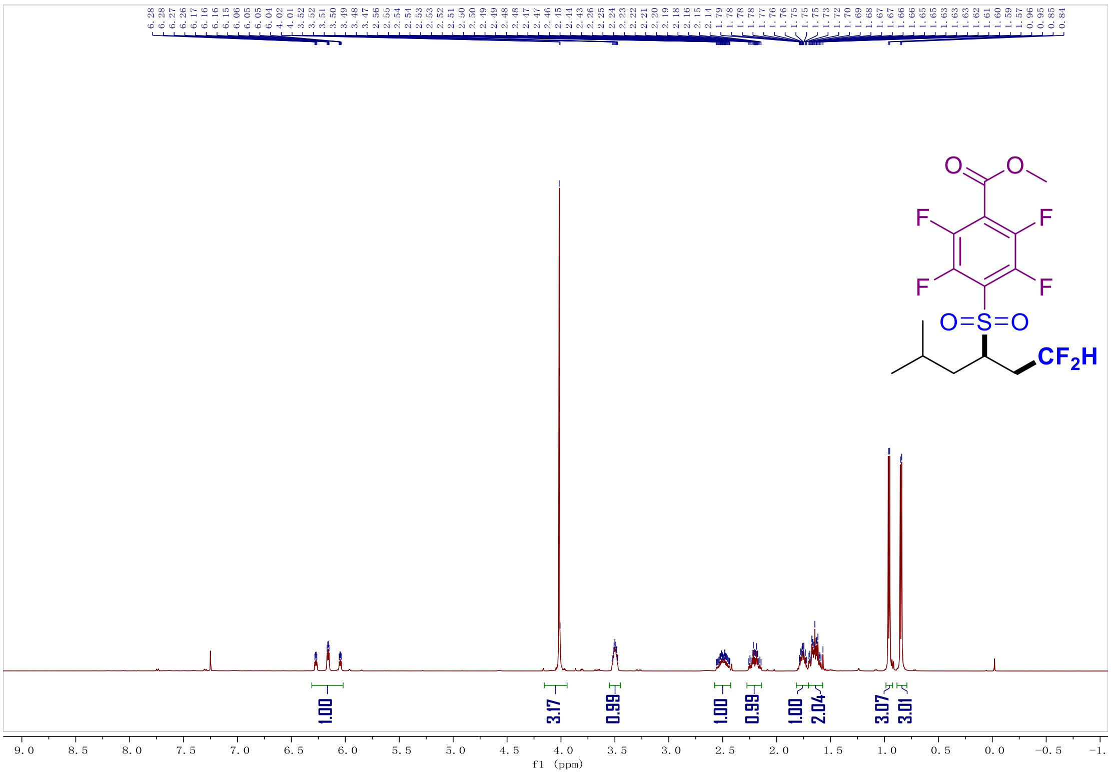

^{13}C NMR (151 MHz, $CDCl_3$) spectrum of 40

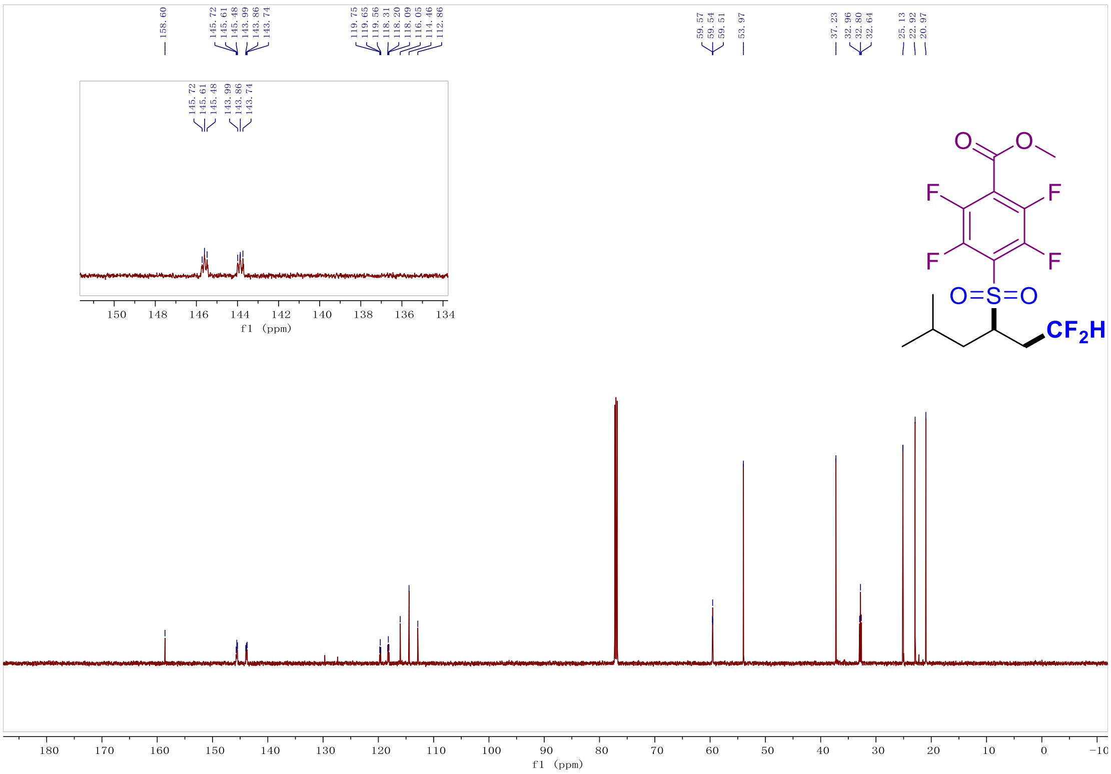

^{19}F NMR (565 MHz, CDCl3) spectrum of 40

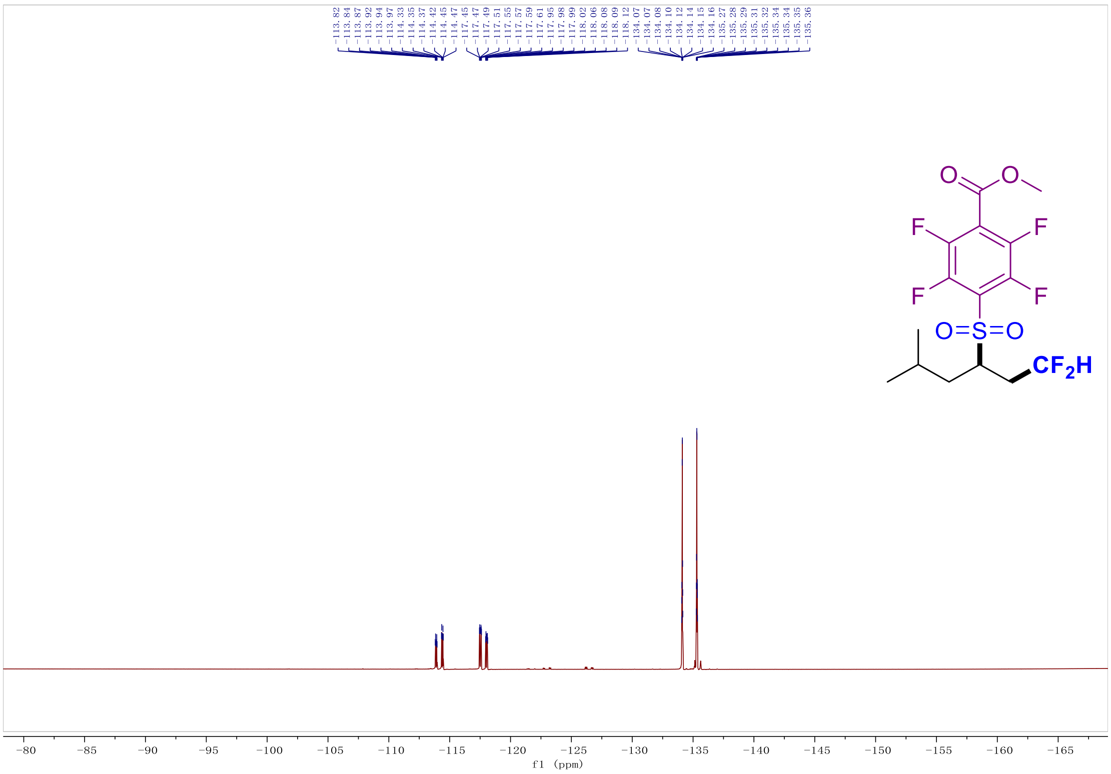

^{1}H NMR (500 MHz, CDCl3) spectrum of 41

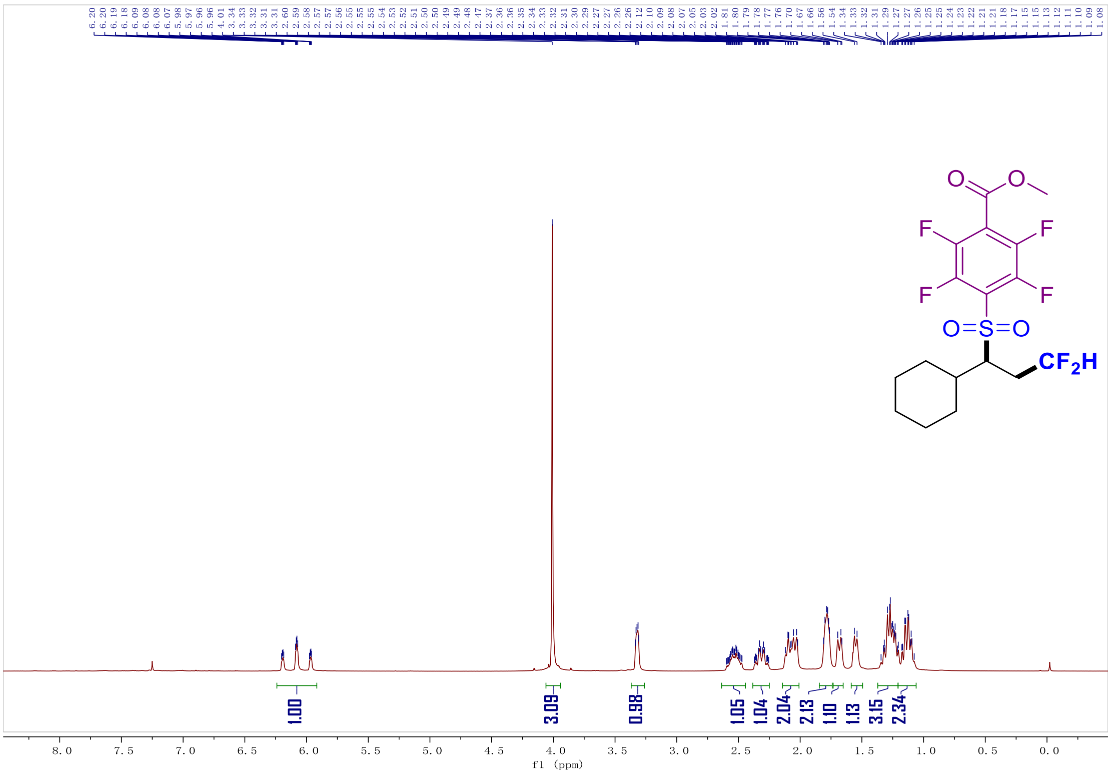

^{13}C NMR (151 MHz, $CDCl_3$) spectrum of 41

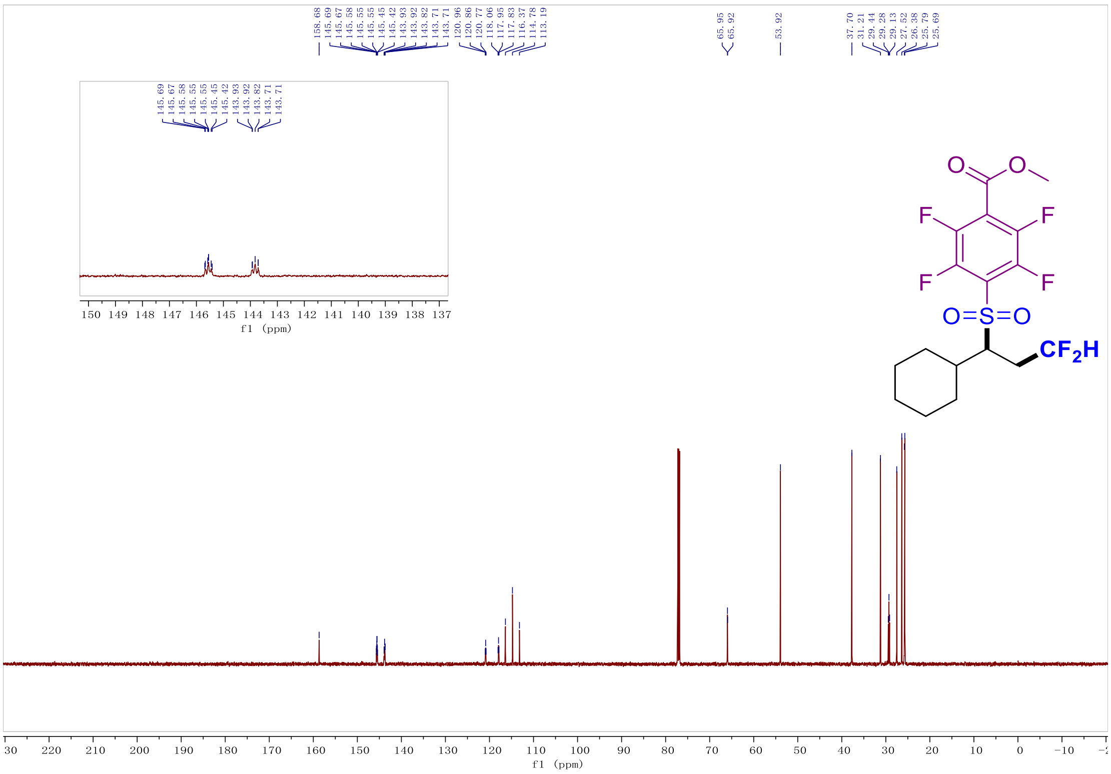

^{19}F NMR (565 MHz, $CDCl_3$) spectrum of 41

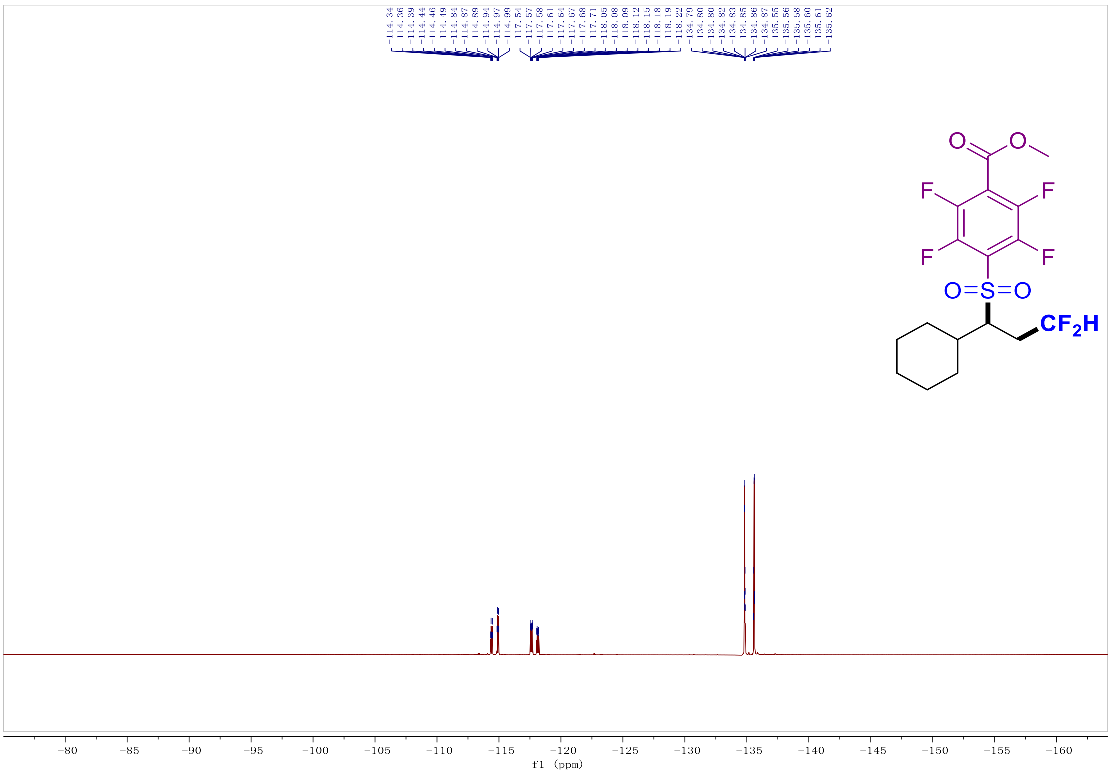

^{1}H NMR (500 MHz, CDCl$_3$) spectrum of 42

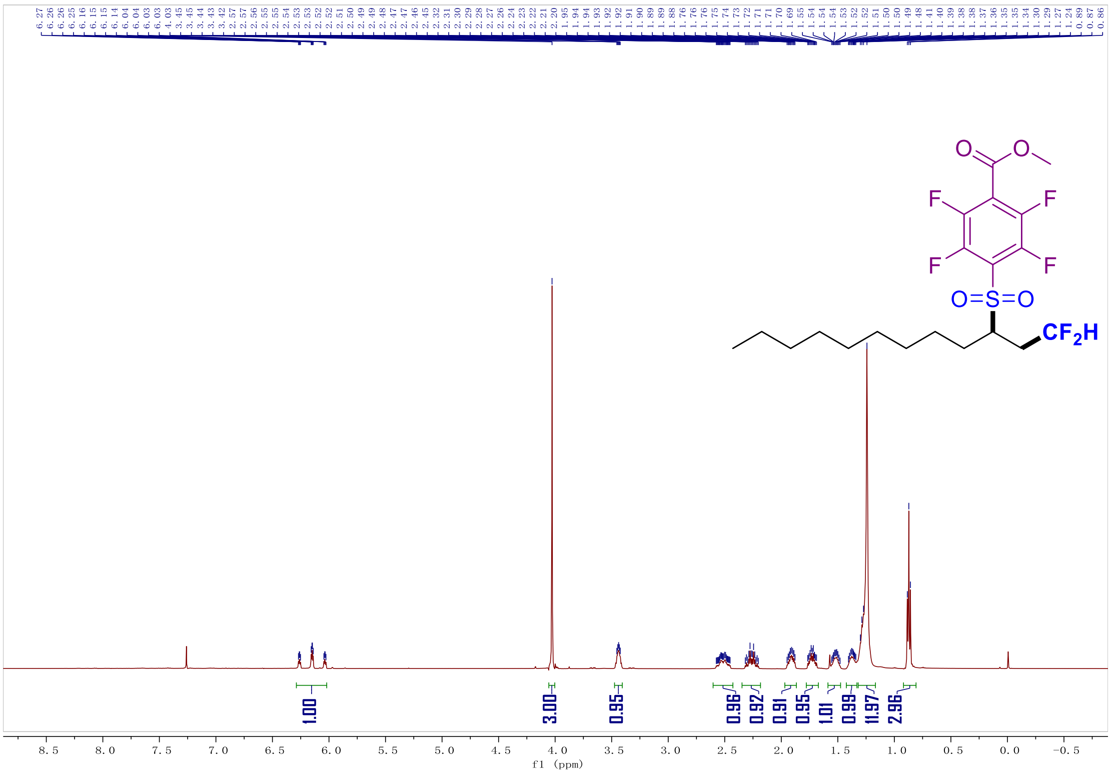

^{13}C NMR (151 MHz, CDCl$_3$) spectrum of 42

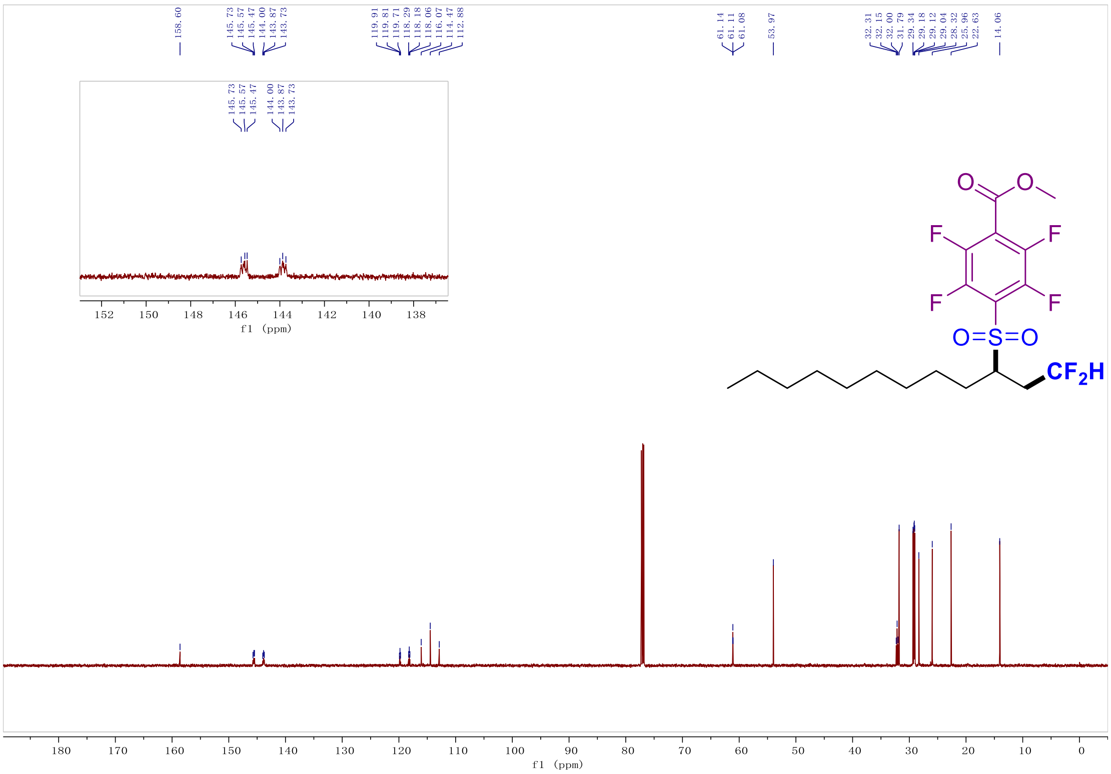

^{19}F NMR (565 MHz, CDCl$_3$) spectrum of 42

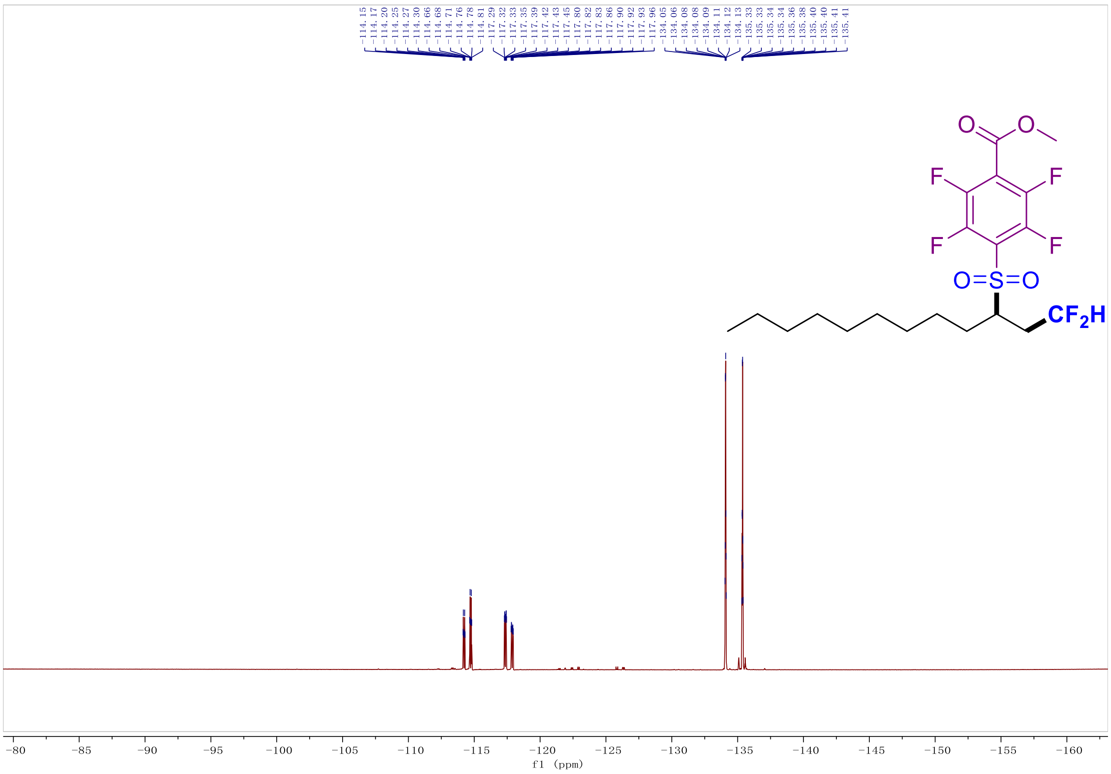

^{1}H NMR (500 MHz, CDCl$_3$) spectrum of 43

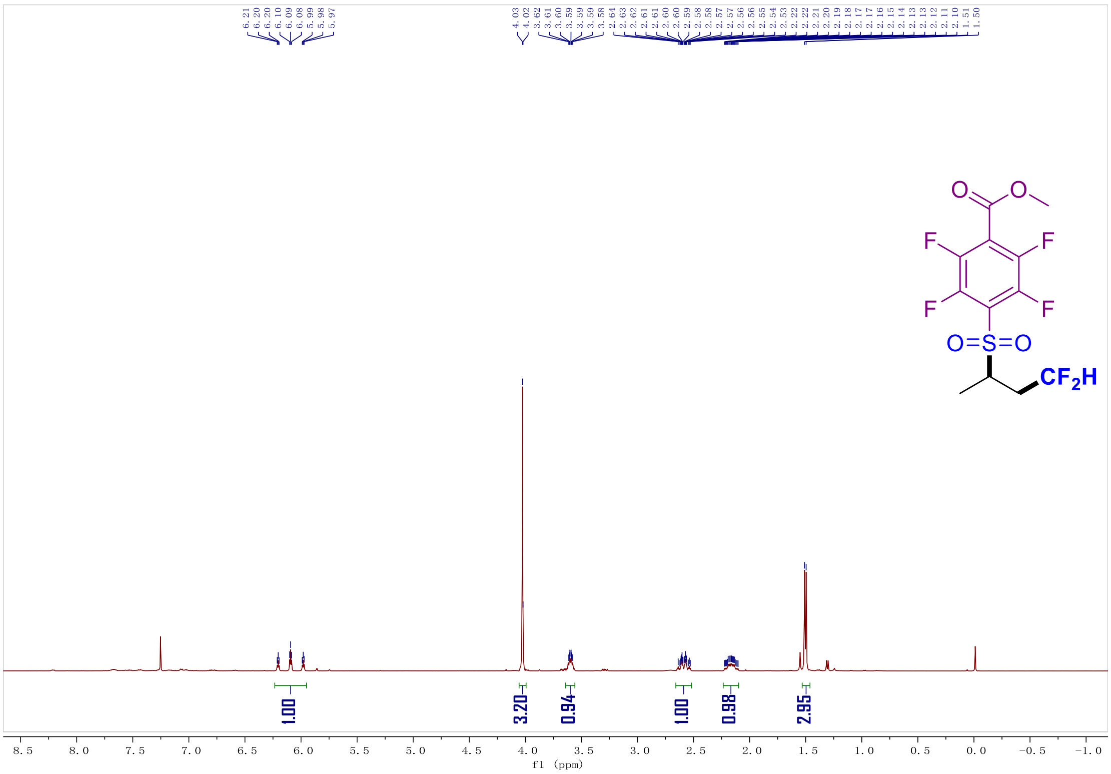

^{13}C NMR (151 MHz, CDCl$_3$) spectrum of 43

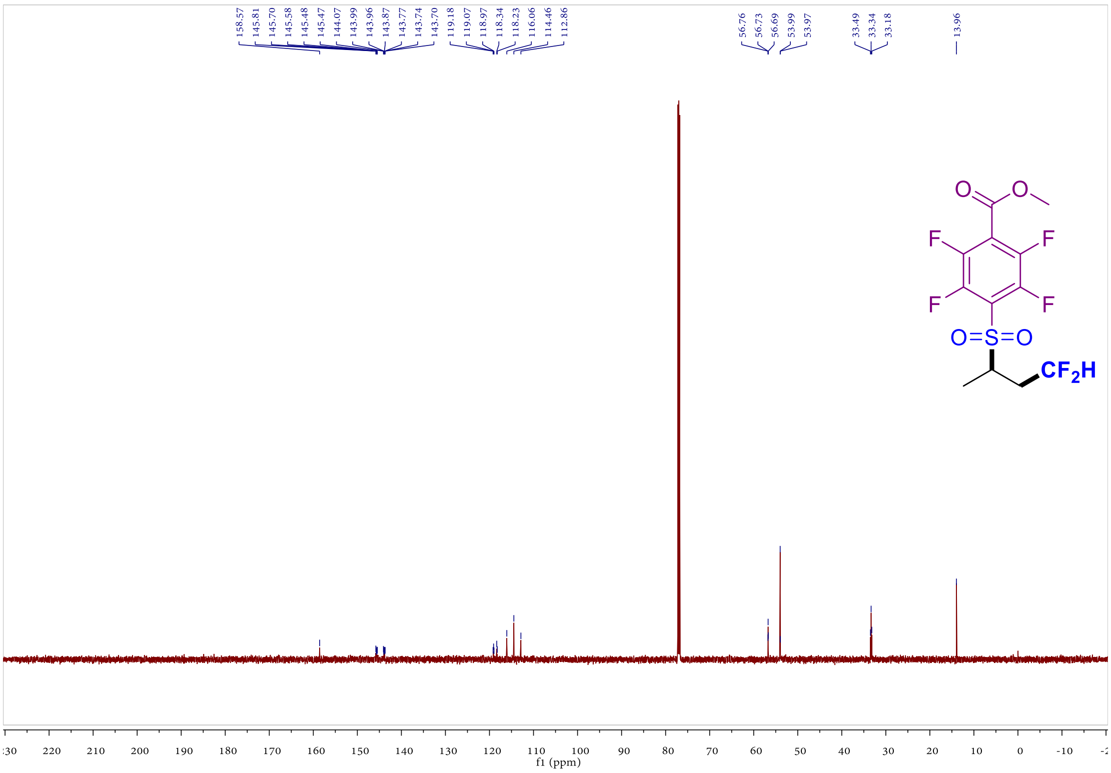

^{19}F NMR (565 MHz, CDCl$_3$) spectrum of 43

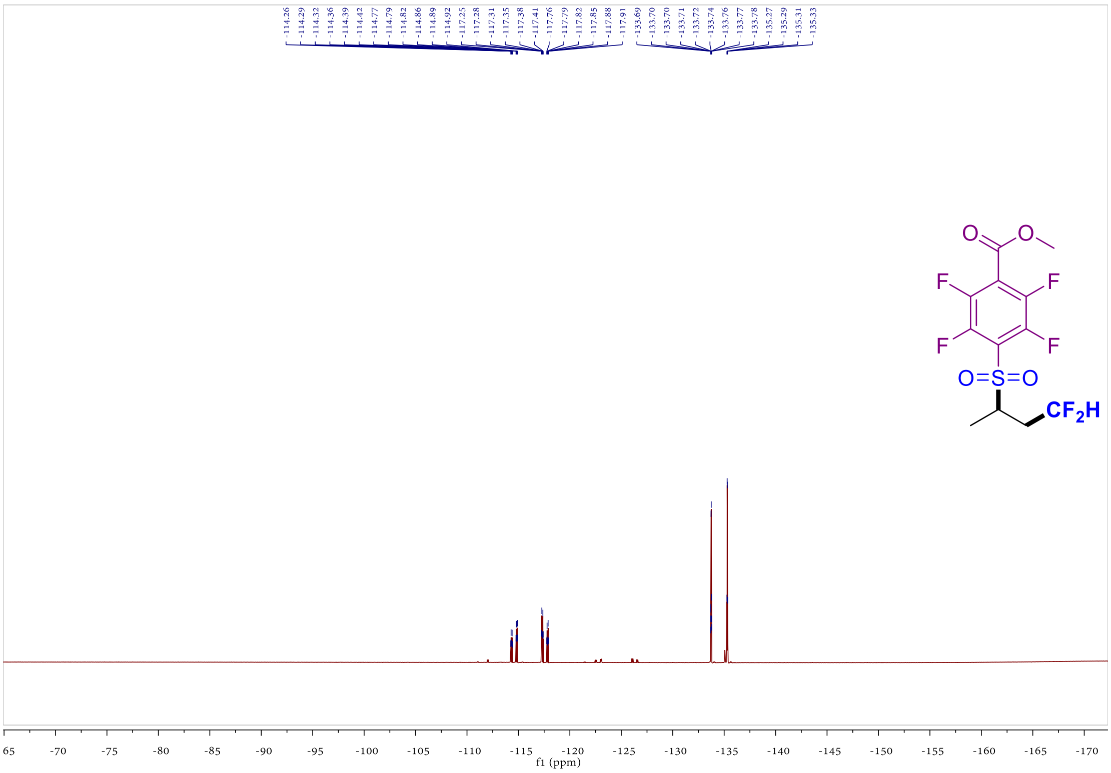

^{1}H NMR (500 MHz, $CDCl_3$) spectrum of 44

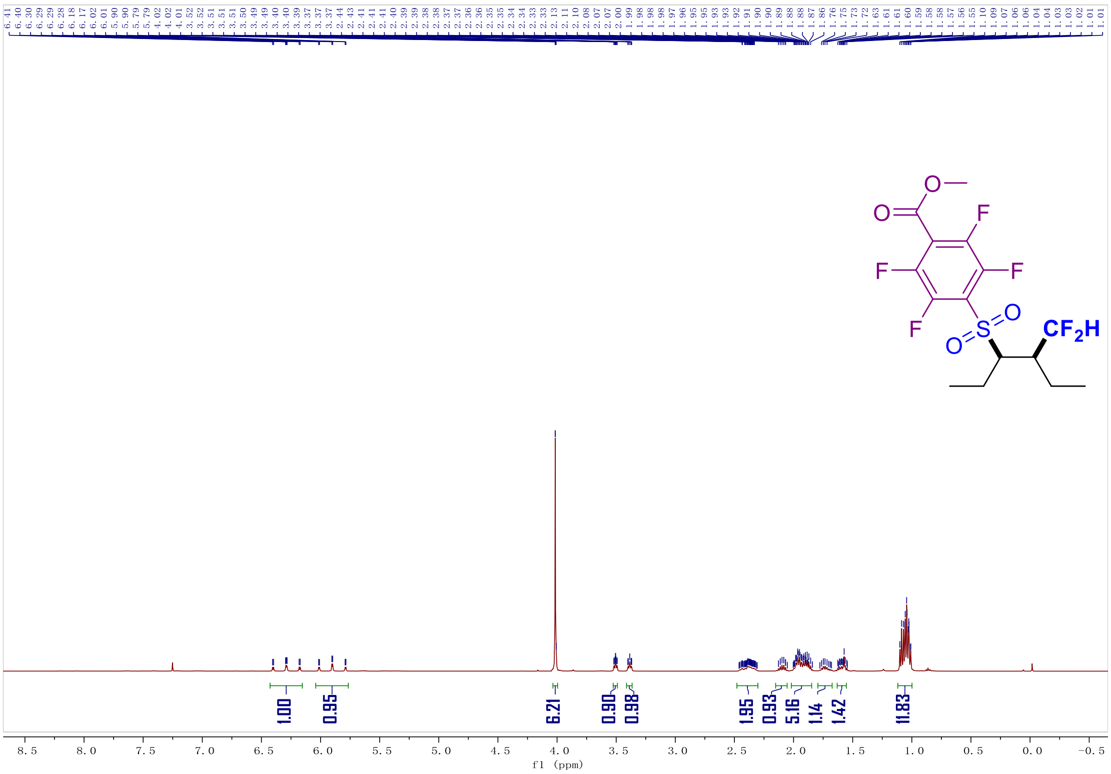

^{13}C NMR (151 MHz, $CDCl_3$) spectrum of 44

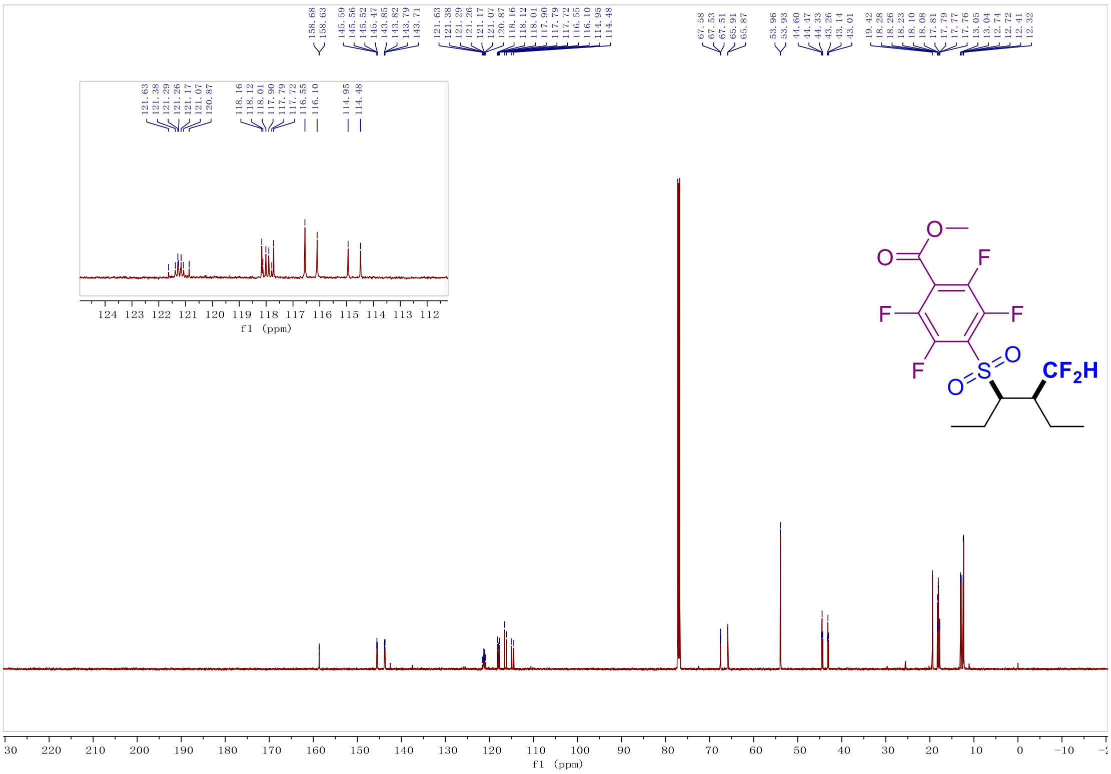

^{19}F NMR (151 MHz, CDCl$_3$) spectrum of 44

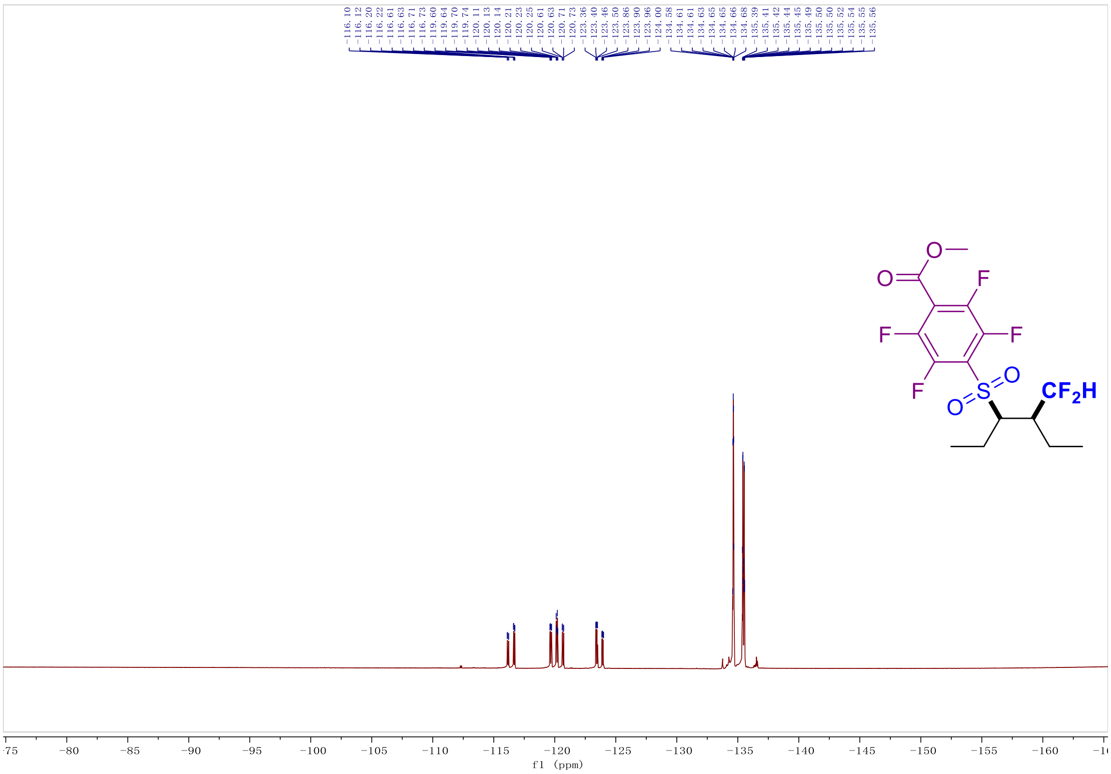

^{1}H NMR (500 MHz, CDCl$_3$) spectrum of 45

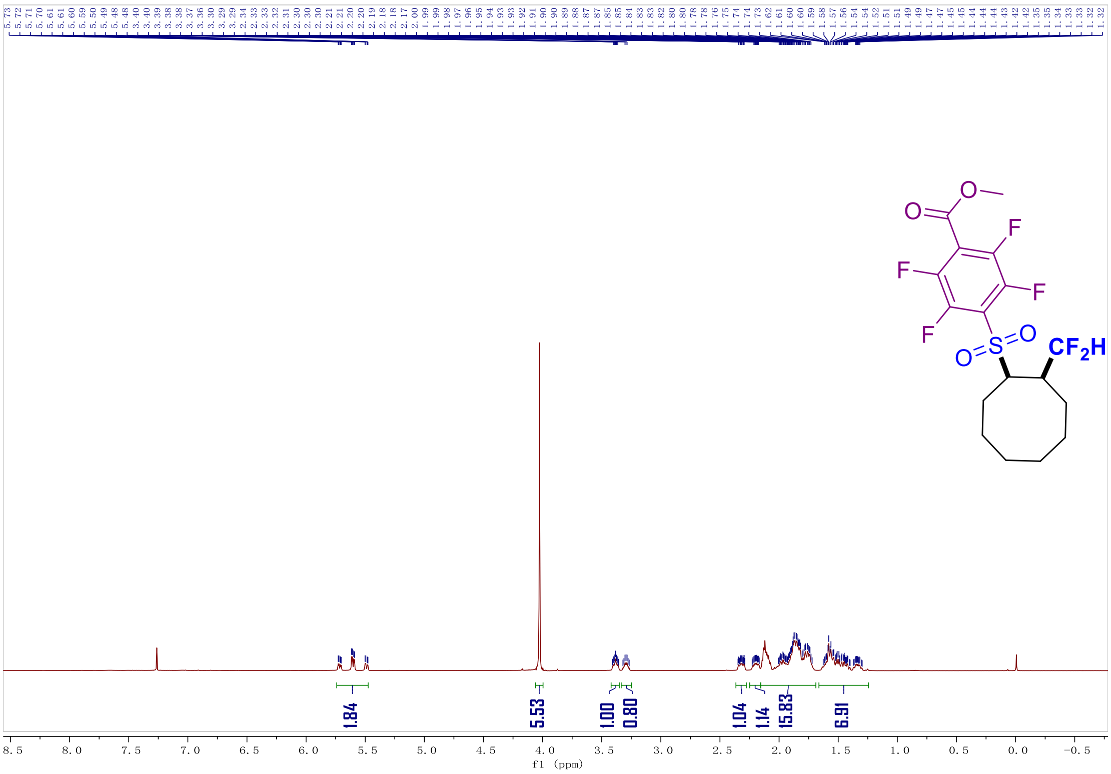

^{13}C NMR (151 MHz, $CDCl_3$) spectrum of 45

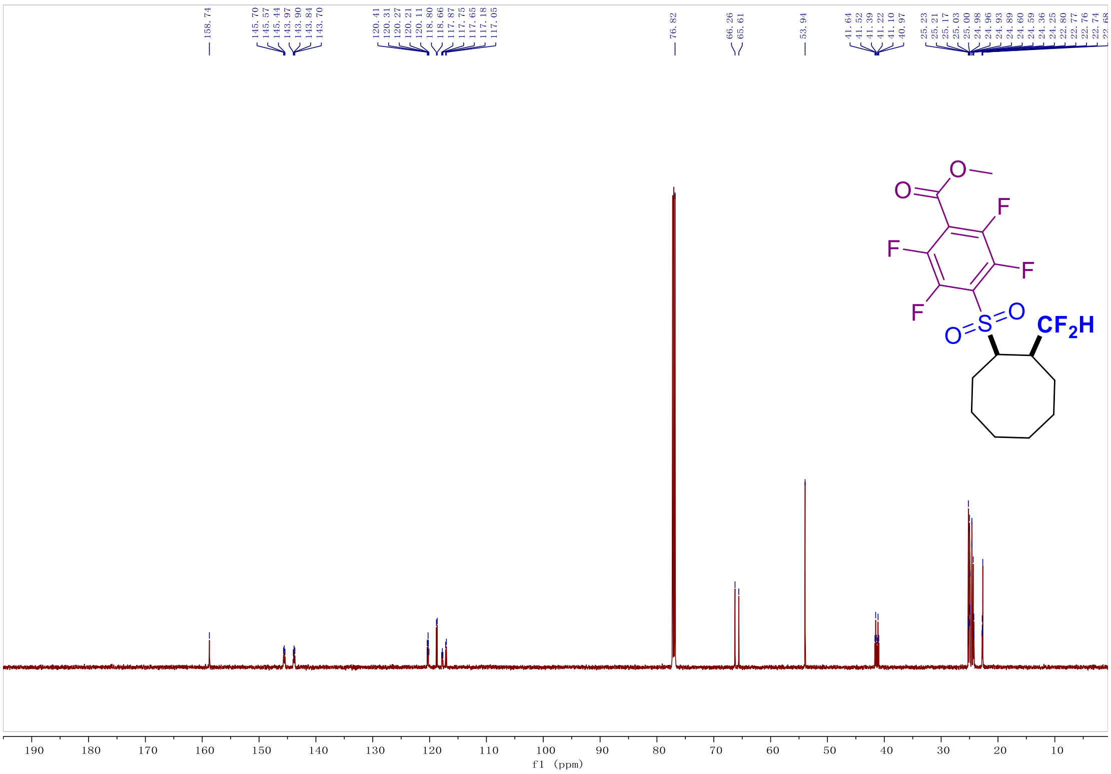

^{19}F NMR (565 MHz, $CDCl_3$) spectrum of 45

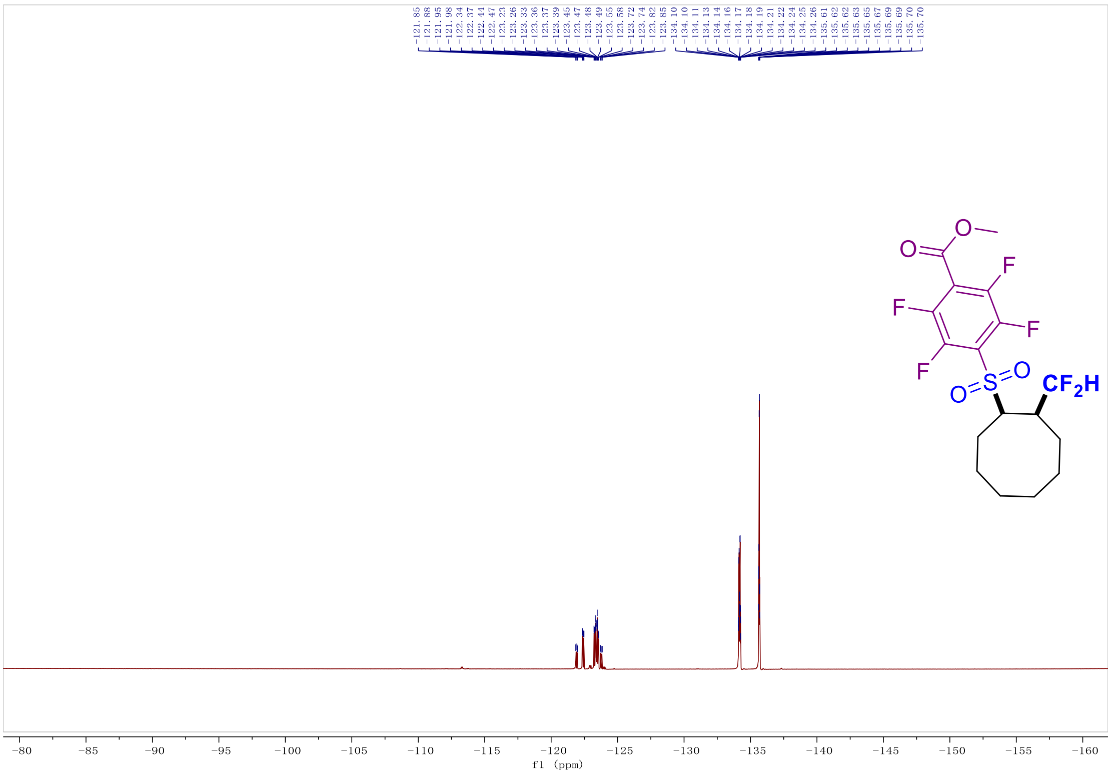

^{1}H NMR (600 MHz, CDCl₃) spectrum of 46

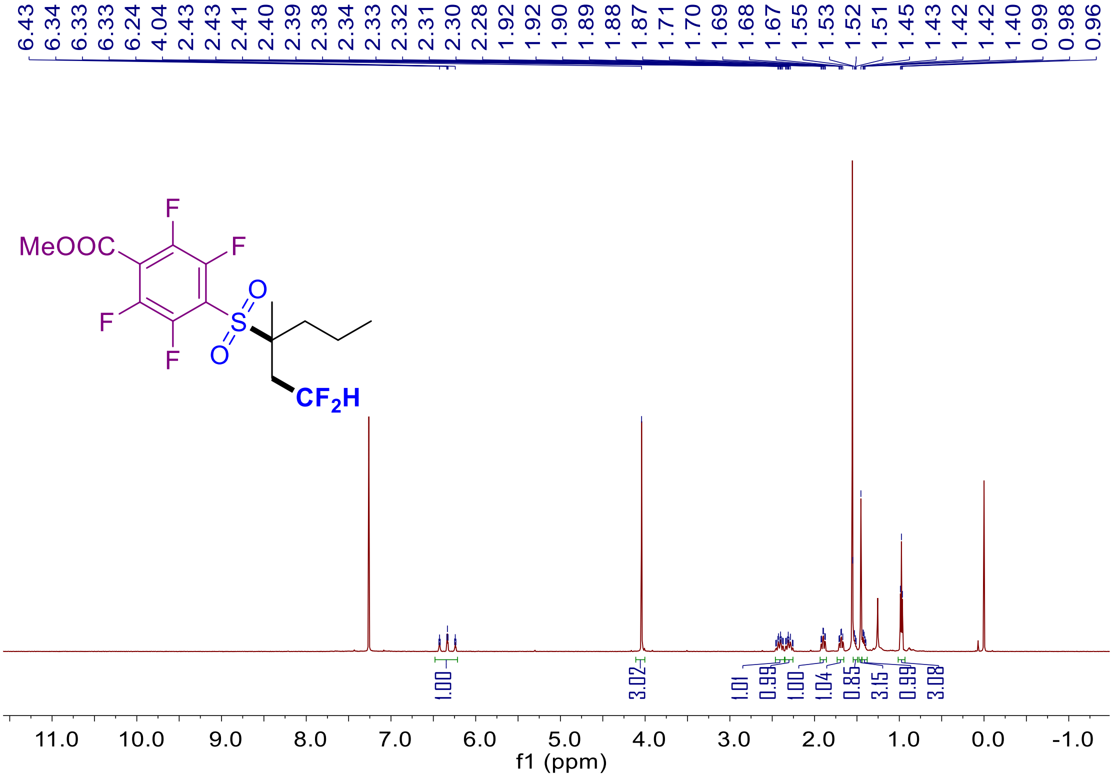

^{13}C NMR (151 MHz, CDCl₃) spectrum of 46

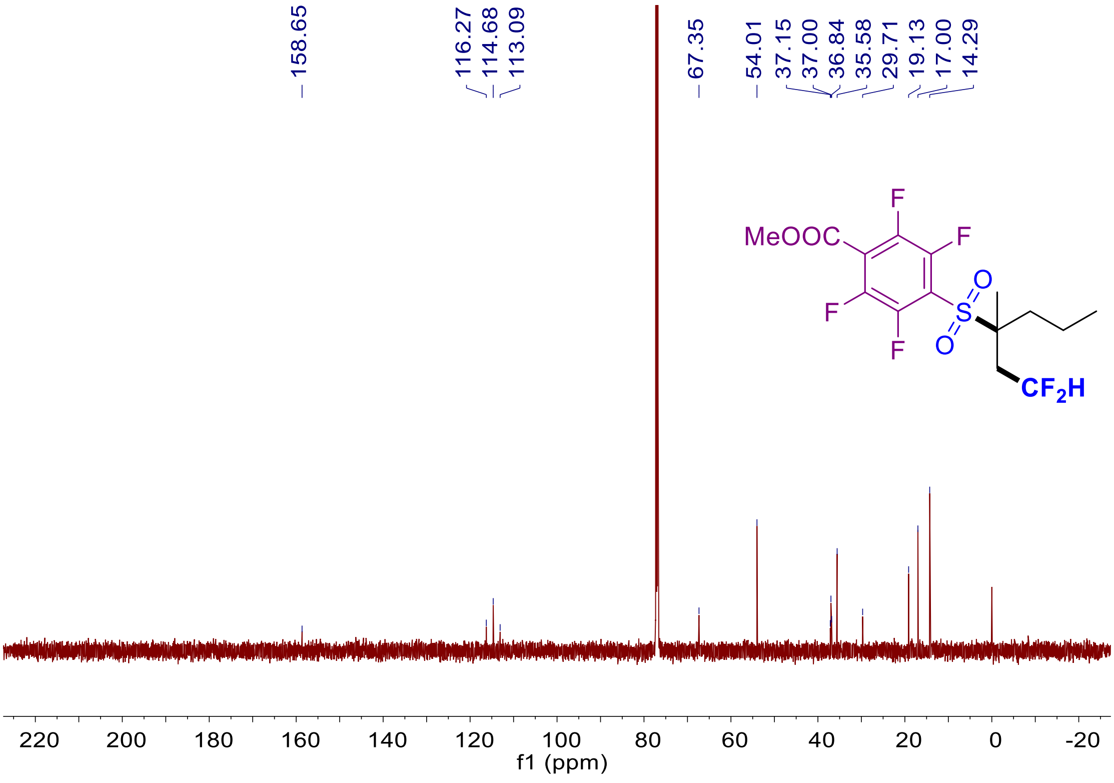

^{19}F NMR (565 MHz, CDCl₃) spectrum of 46

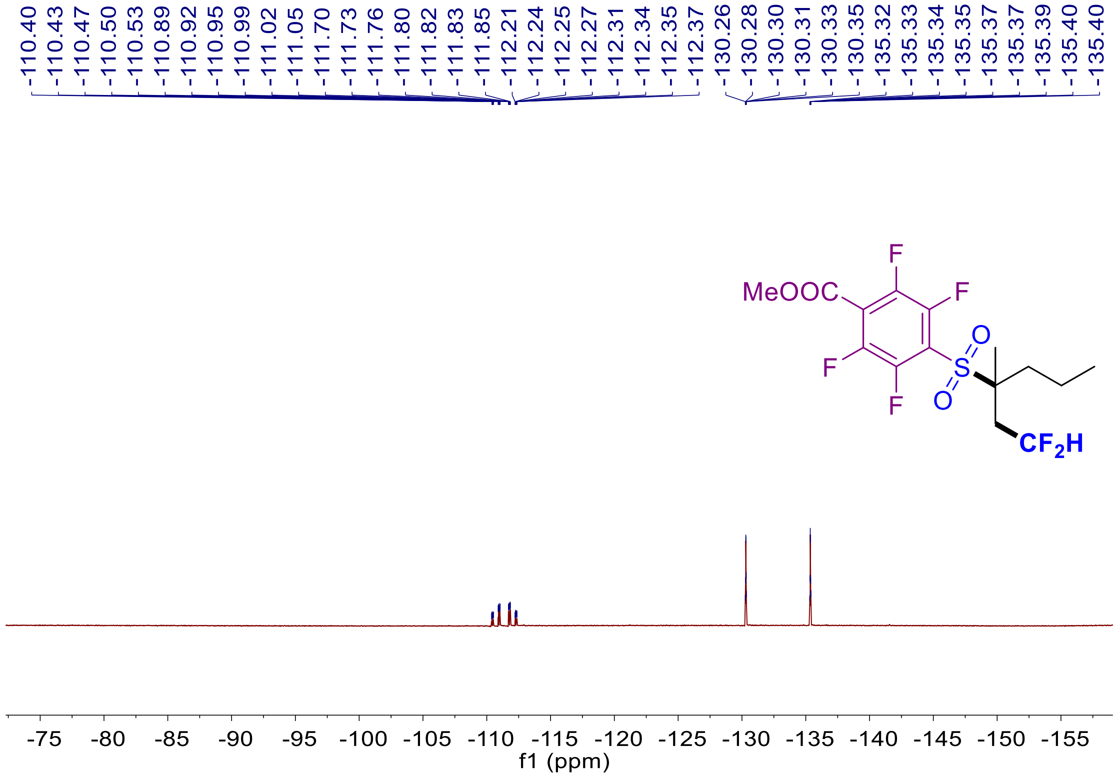

^{1}H NMR (600 MHz, CDCl₃) spectrum of 47

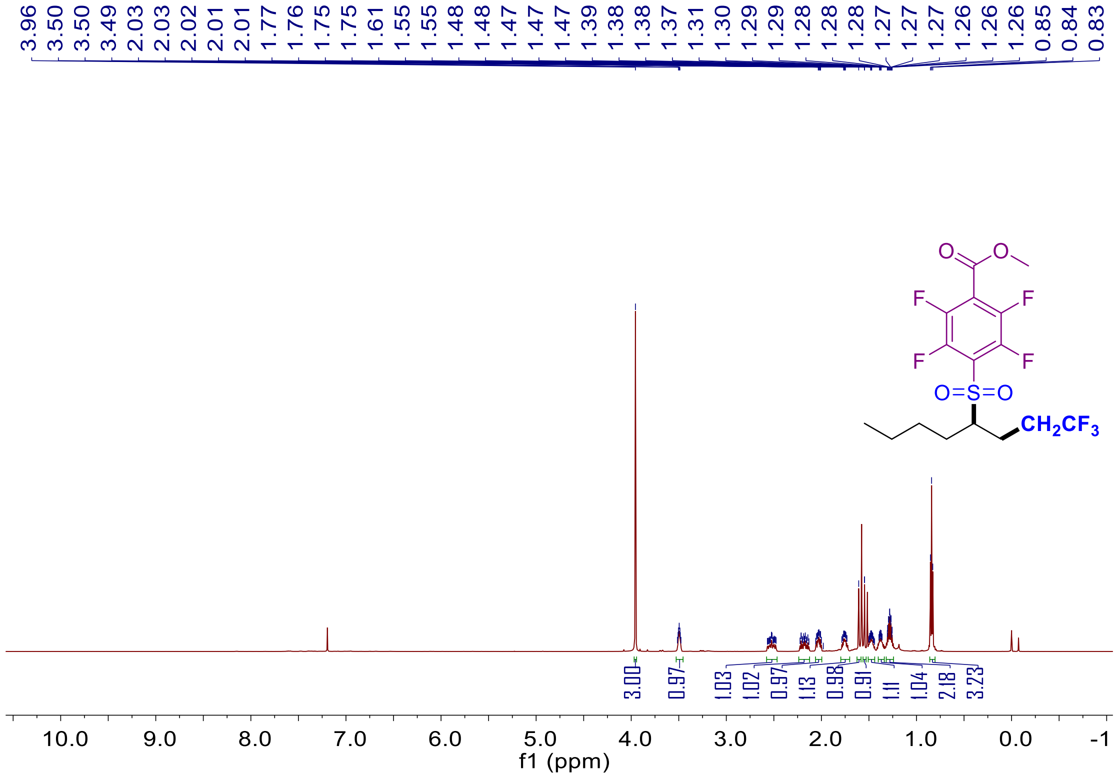

^{13}C NMR (151 MHz, $CDCl_3$) spectrum of 47

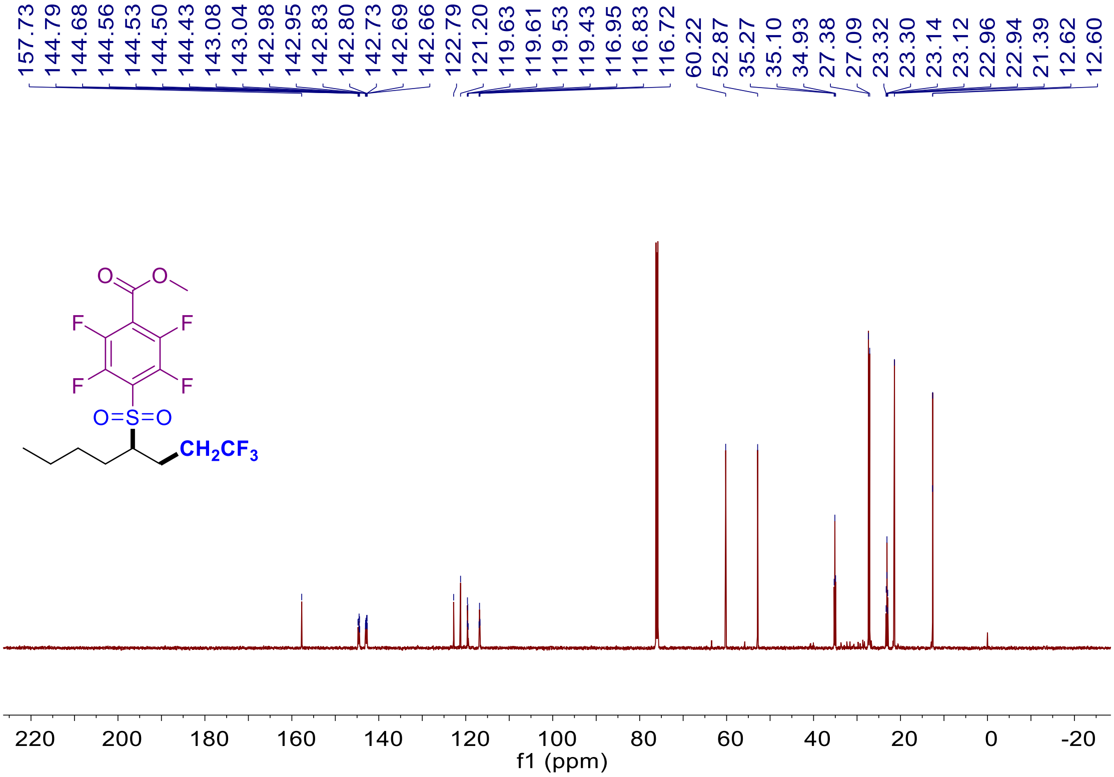

^{19}F NMR (565 MHz, $CDCl_3$) spectrum of 47

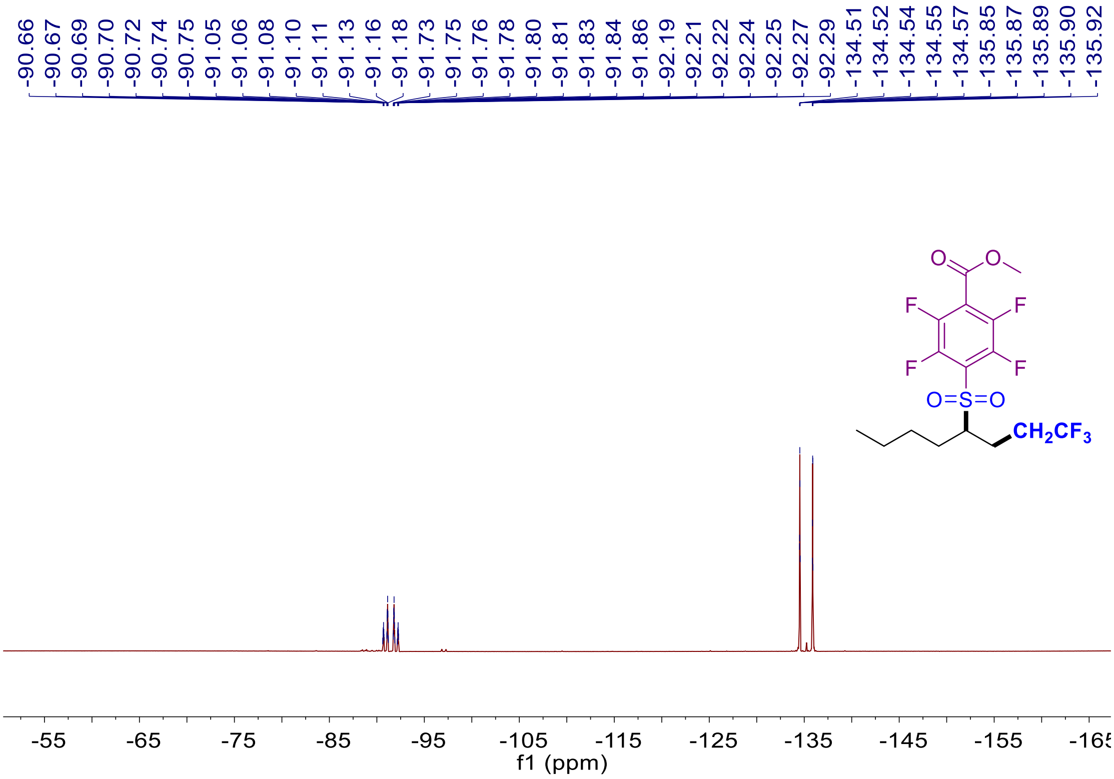

^{1}H NMR (600 MHz, CDCl₃) spectrum of 48

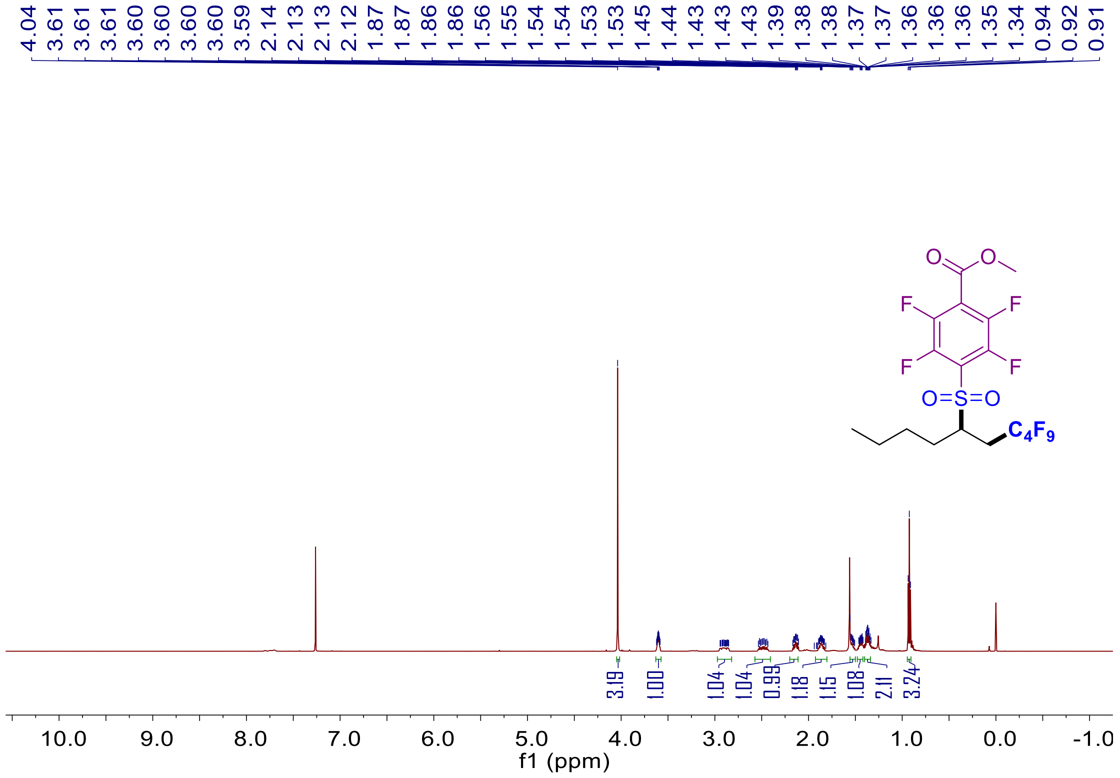

^{13}C NMR (151 MHz, CDCl₃) spectrum of 48

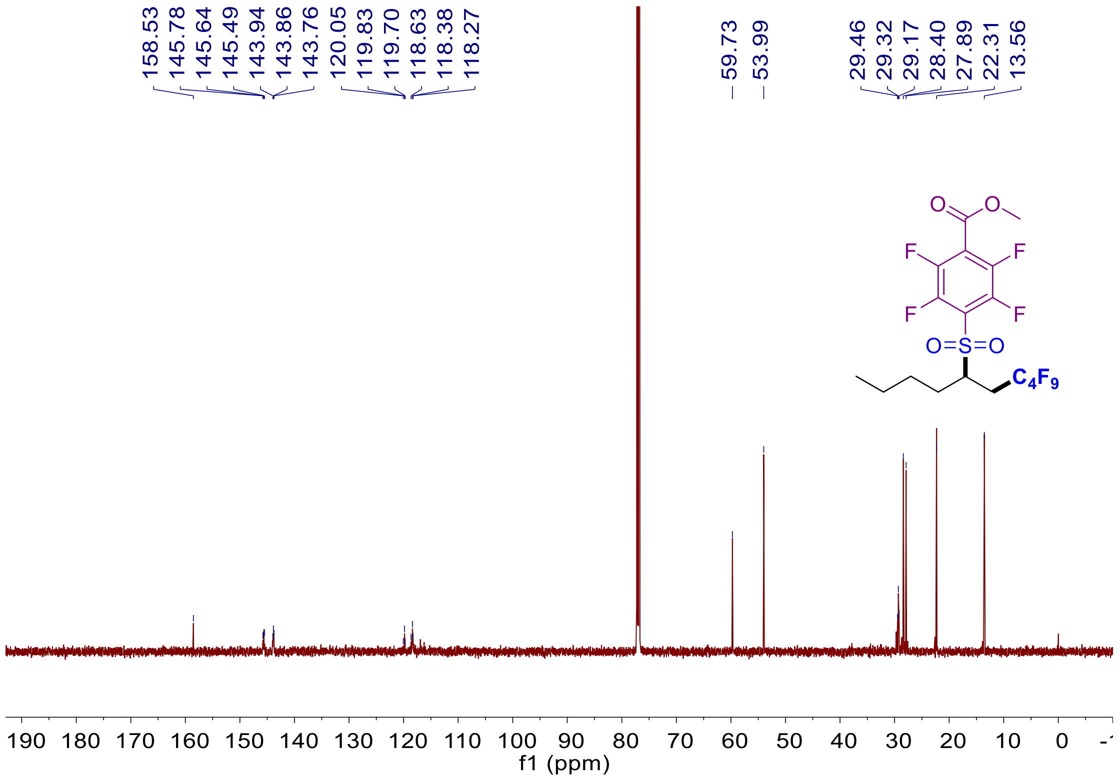

^{19}F NMR (565 MHz, $CDCl_3$) spectrum of 48

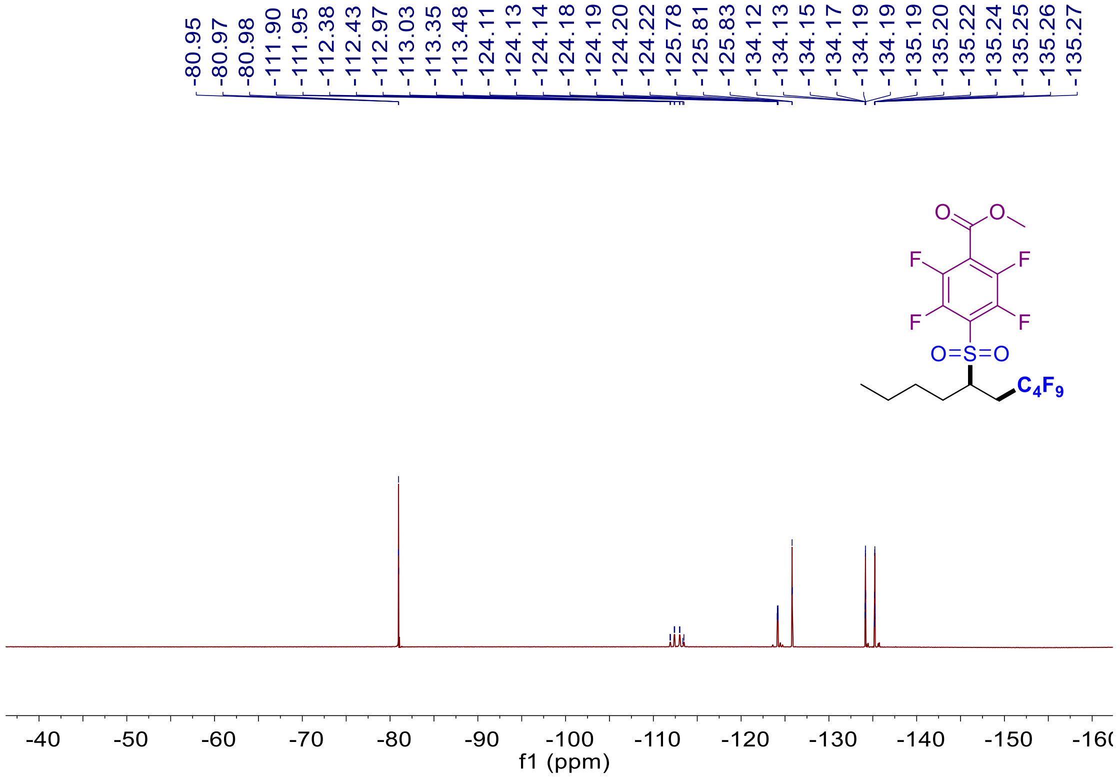

^{1}H NMR (600 MHz, $CDCl_3$) spectrum of 49

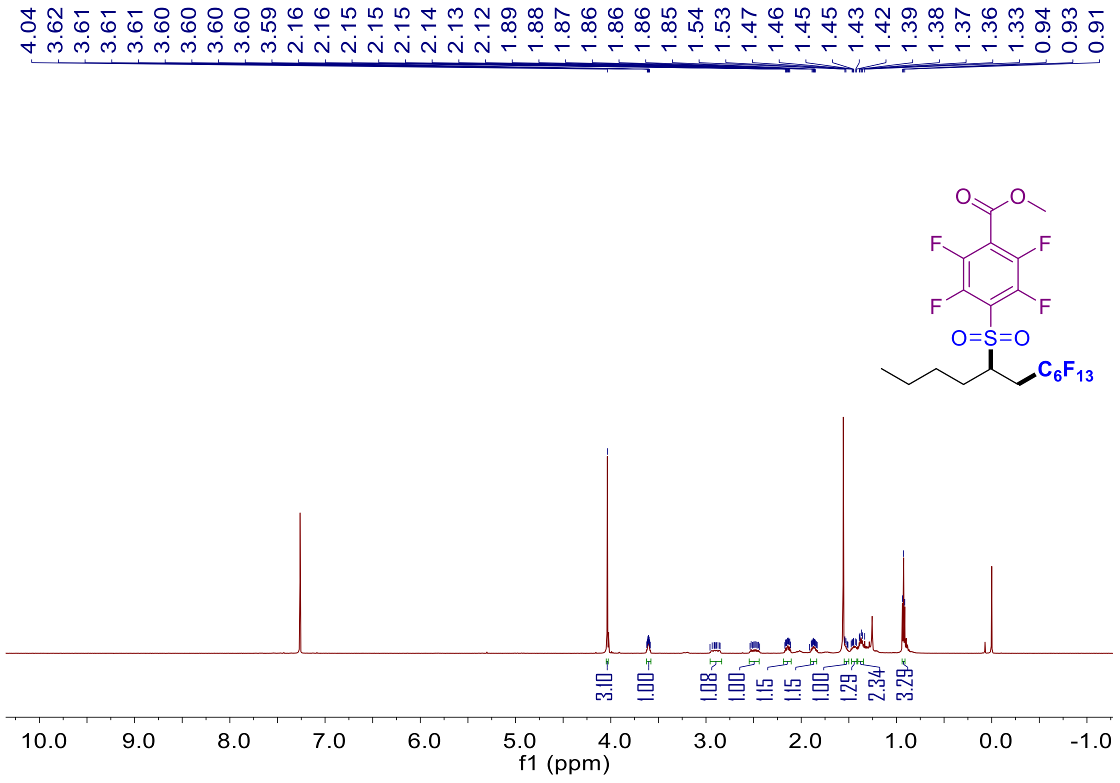

^{13}C NMR (151 MHz, $CDCl_3$) spectrum of 49

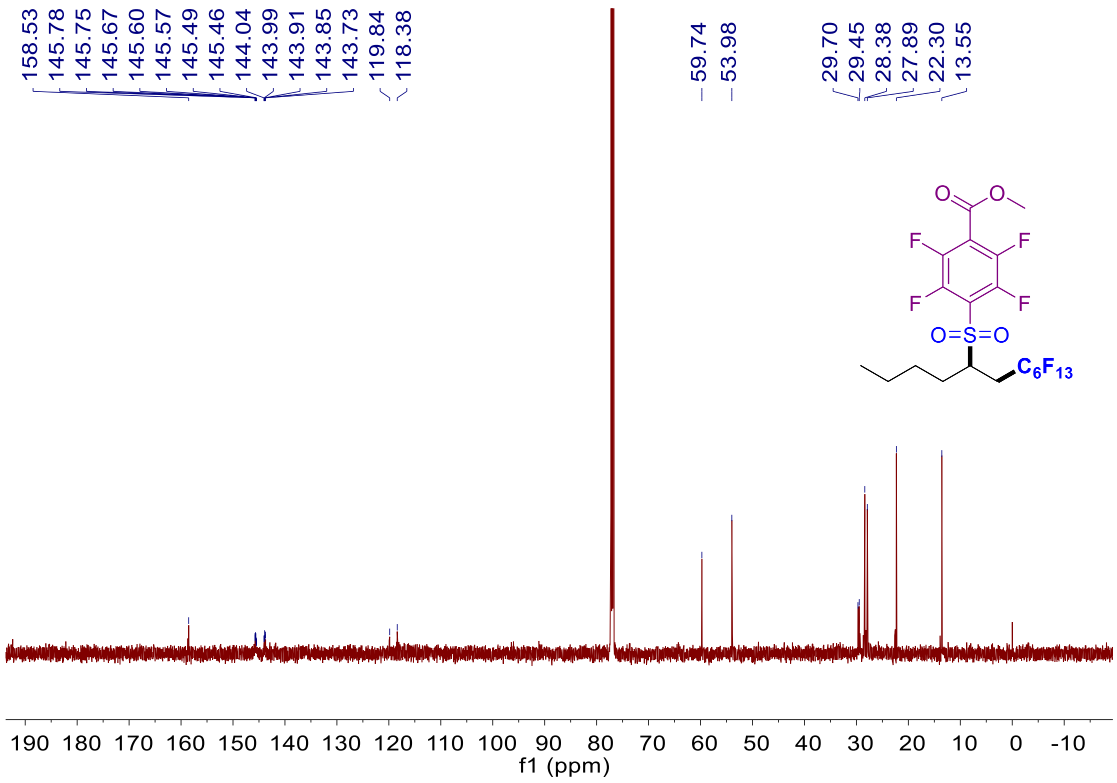

^{19}F NMR (565 MHz, $CDCl_3$) spectrum of 49

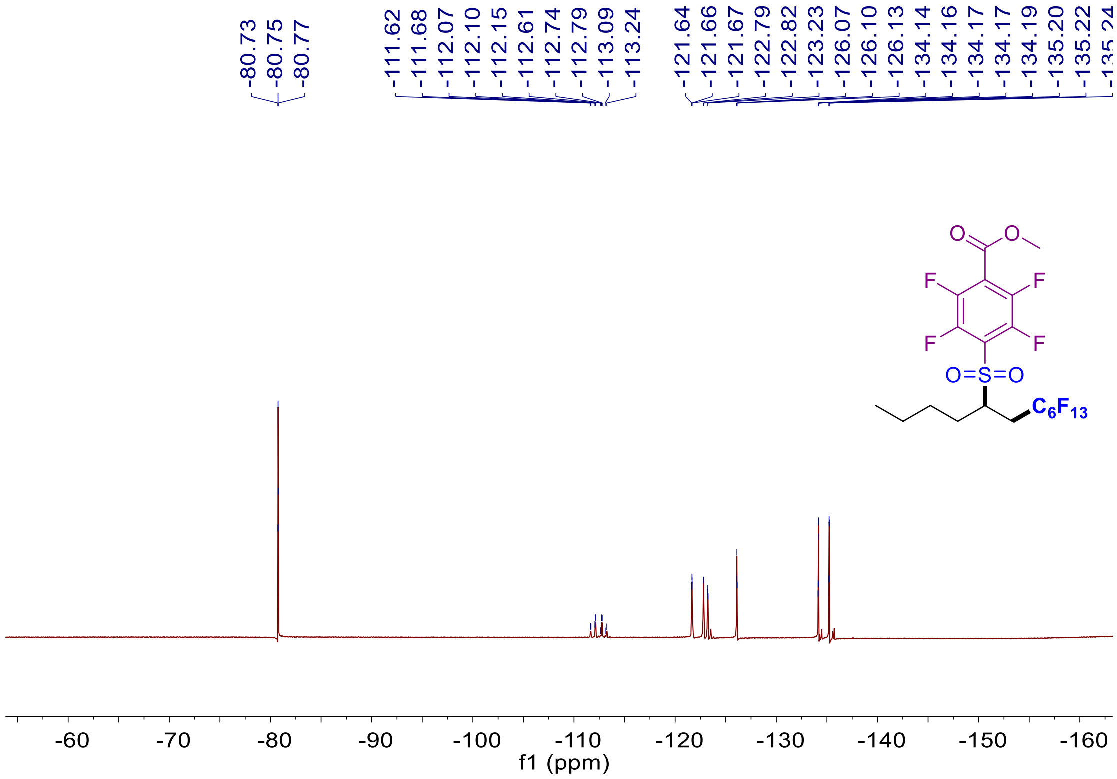

^{1}H NMR (500 MHz, $CDCl_3$) spectrum of 50

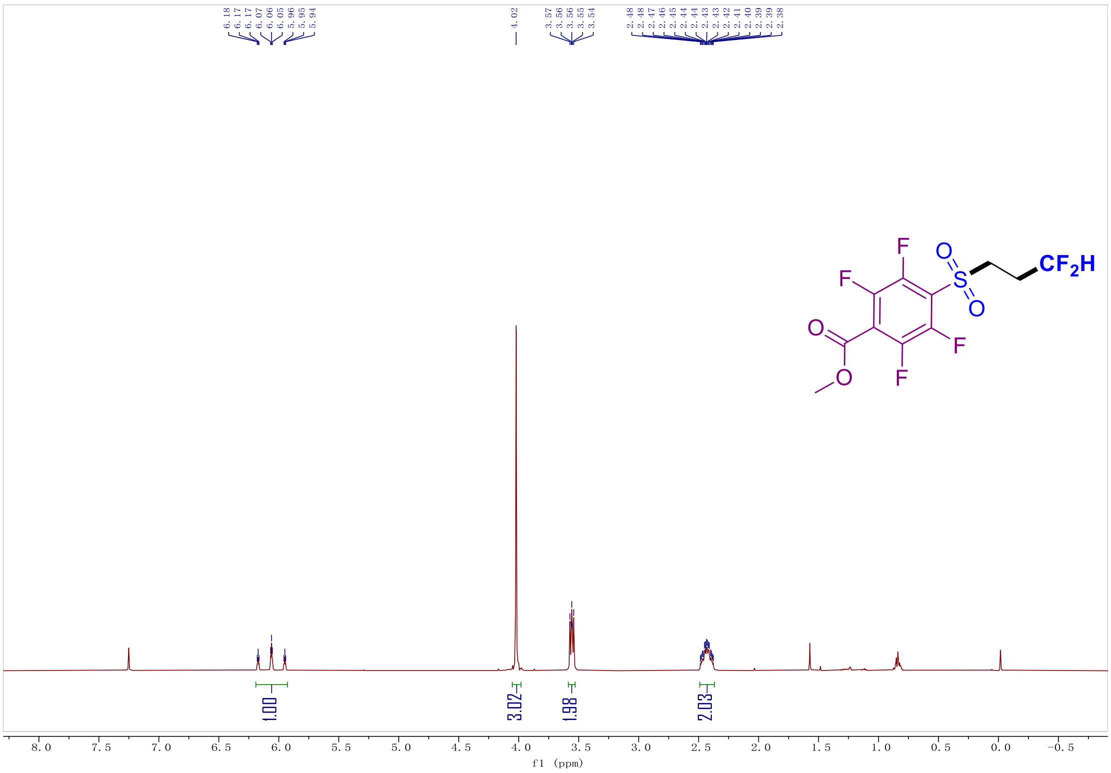

^{13}C NMR (151 MHz, $CDCl_3$) spectrum of 50

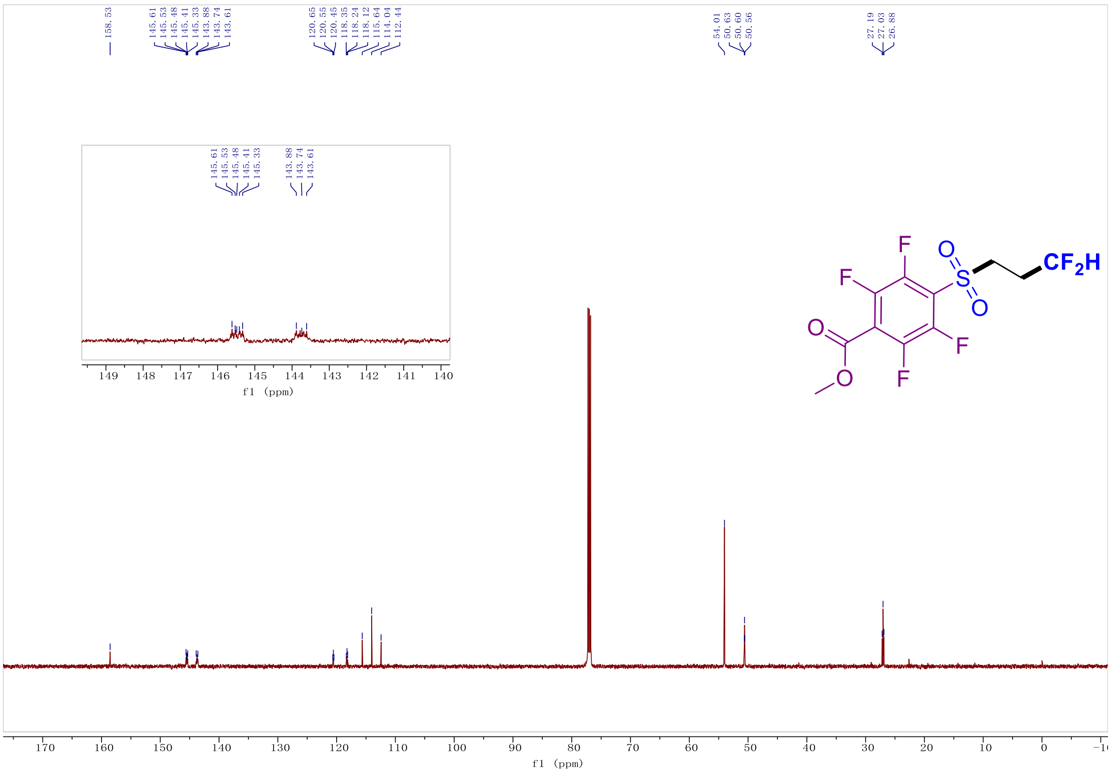

^{19}F NMR (565 MHz, $CDCl_3$) spectrum of 50

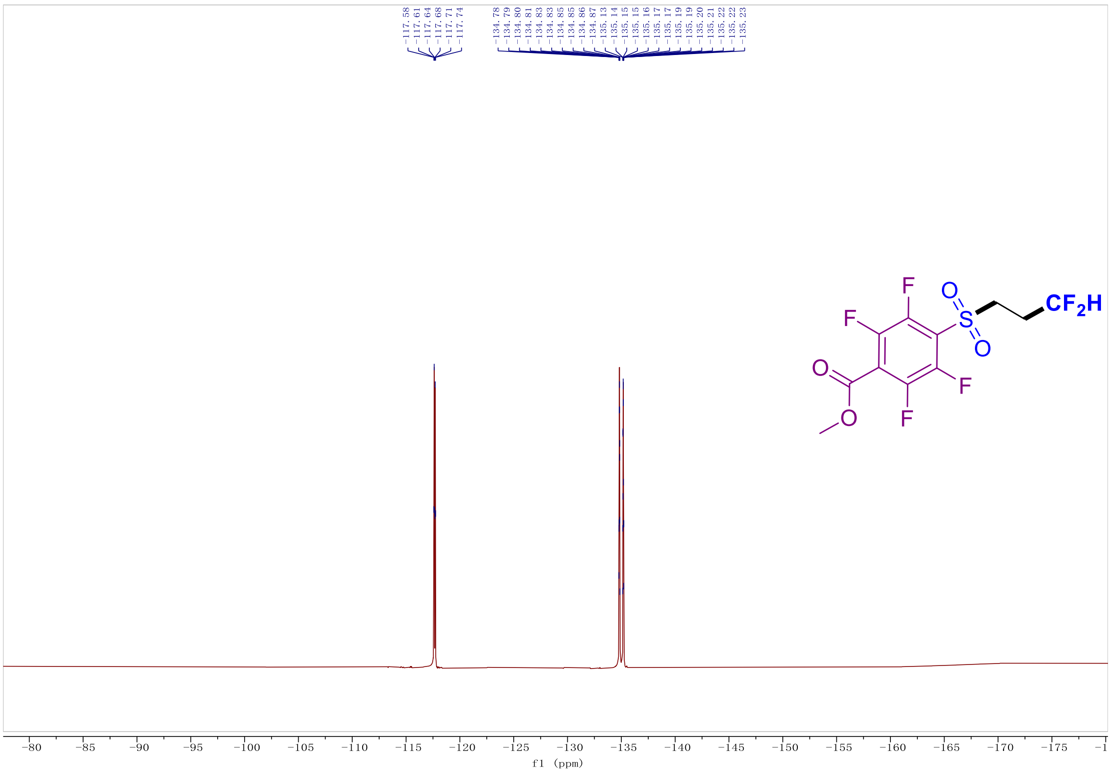

^{1}H NMR (500 MHz, $CDCl_3$) spectrum of 51

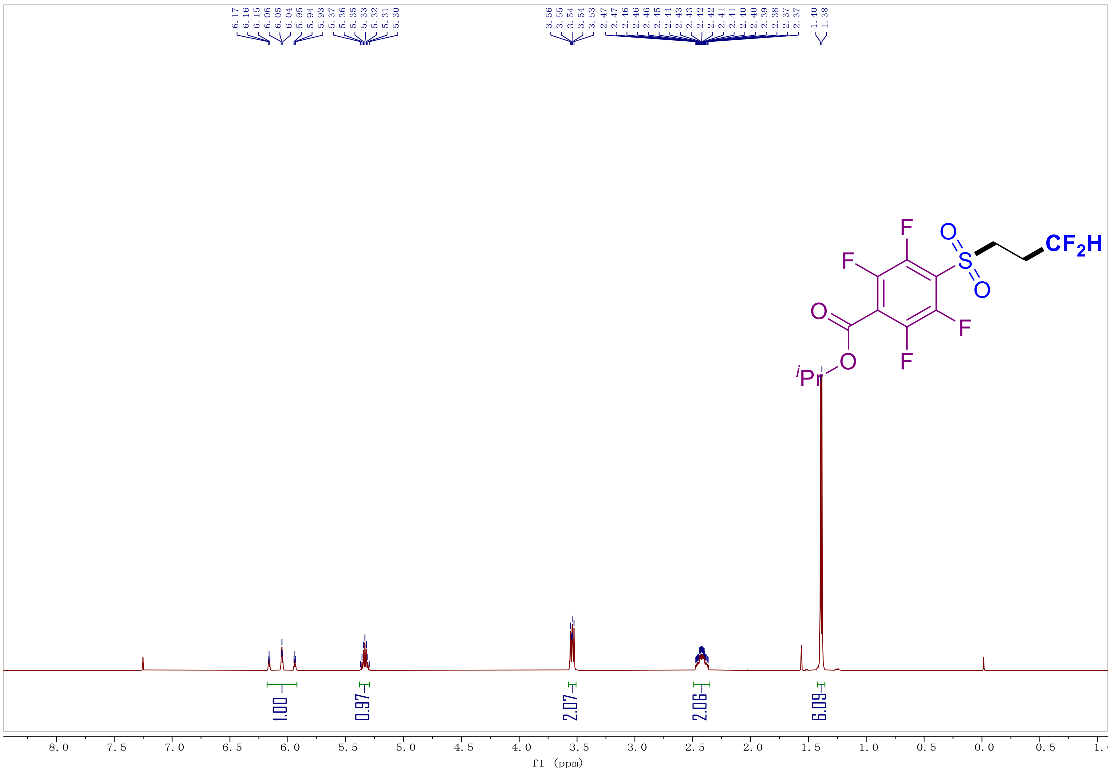

^{13}C NMR (151 MHz, $CDCl_3$) spectrum of 51

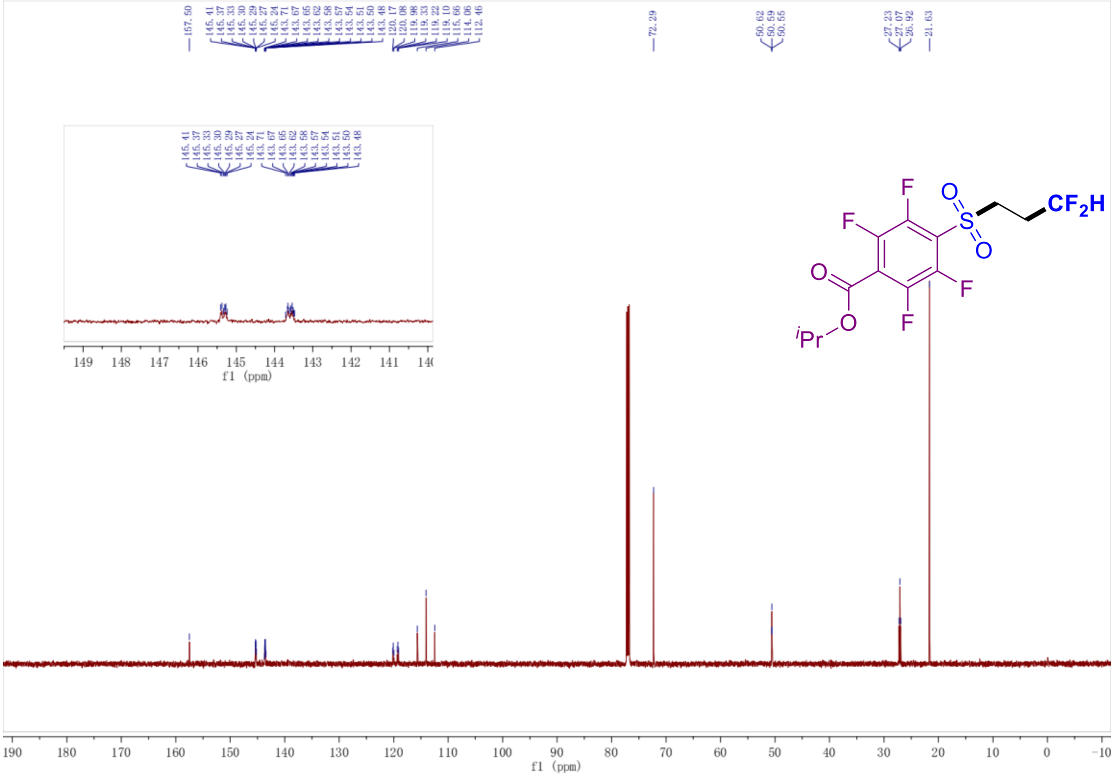

^{19}F NMR (565 MHz, $CDCl_3$) spectrum of 51

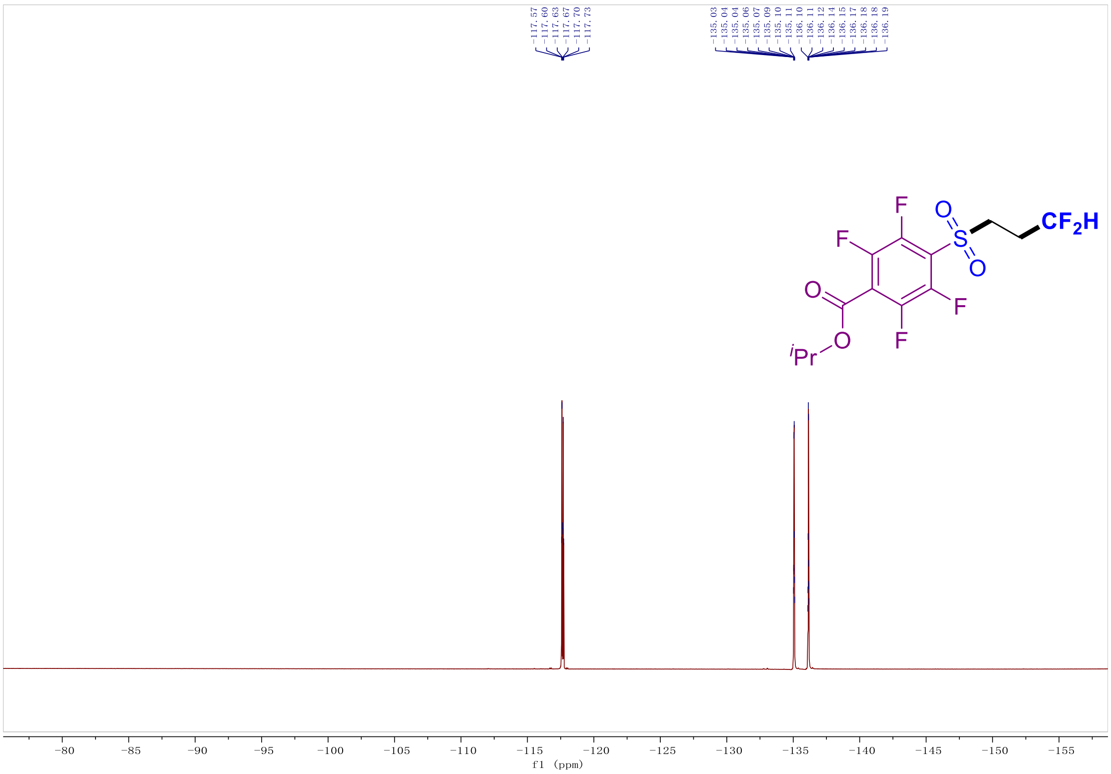

^{1}H NMR (500 MHz, CDCl$_3$) spectrum of 52

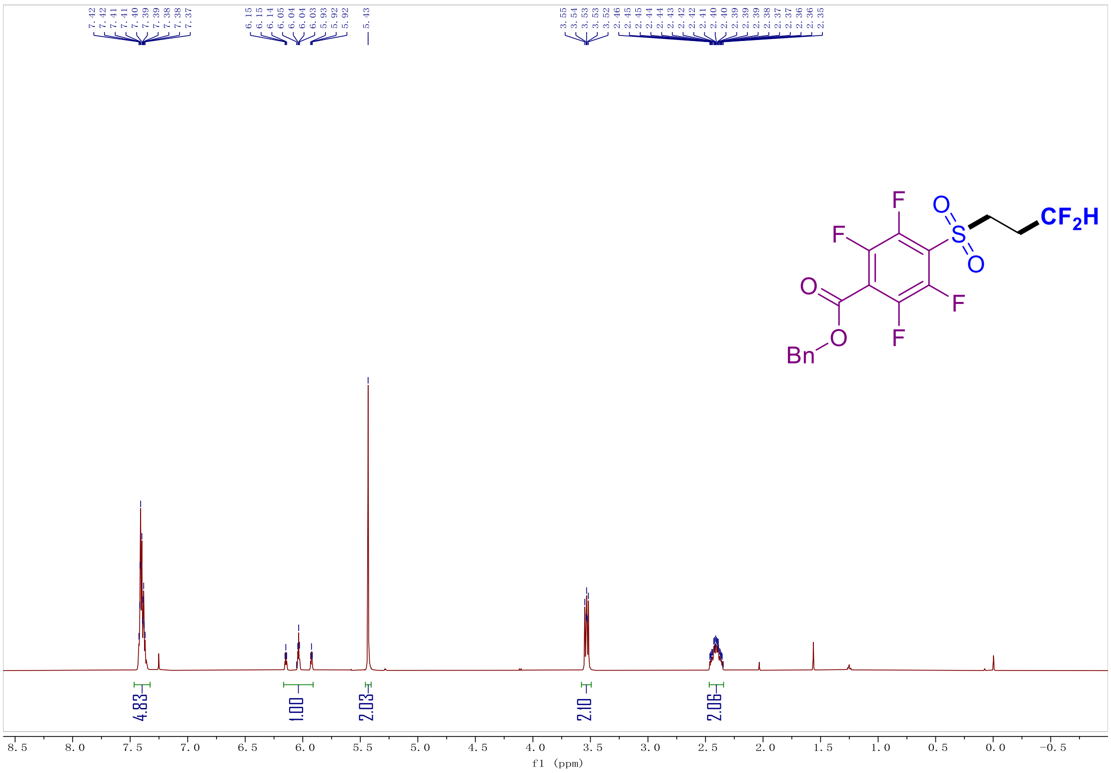

^{13}C NMR (151 MHz, CDCl$_3$) spectrum of 52

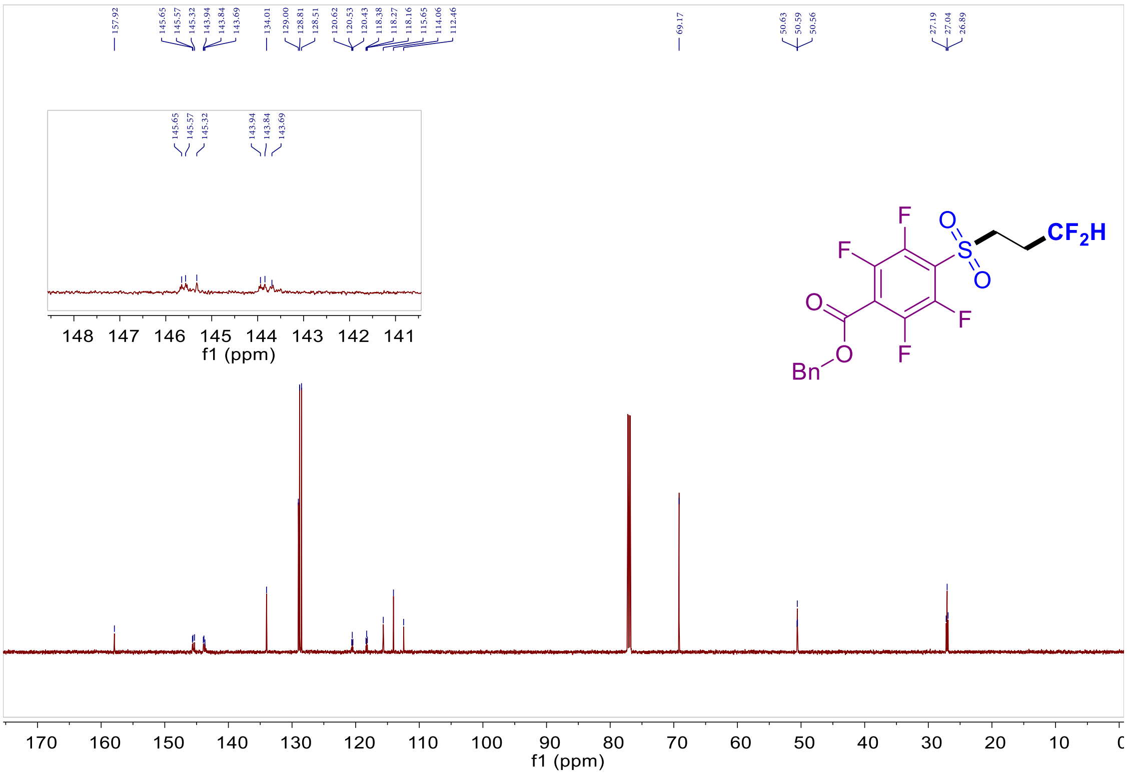

^{19}F NMR (565 MHz, CDCl$_3$) spectrum of 52

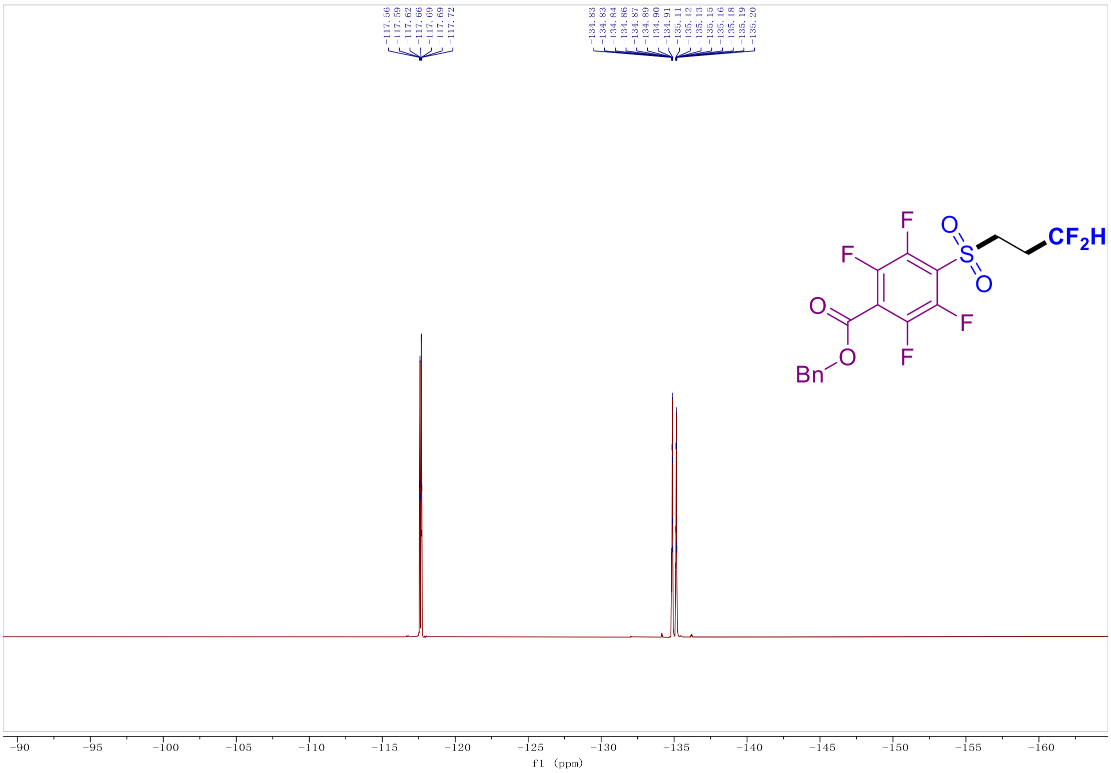

^{1}H NMR (500 MHz, CDCl$_3$) spectrum of 53

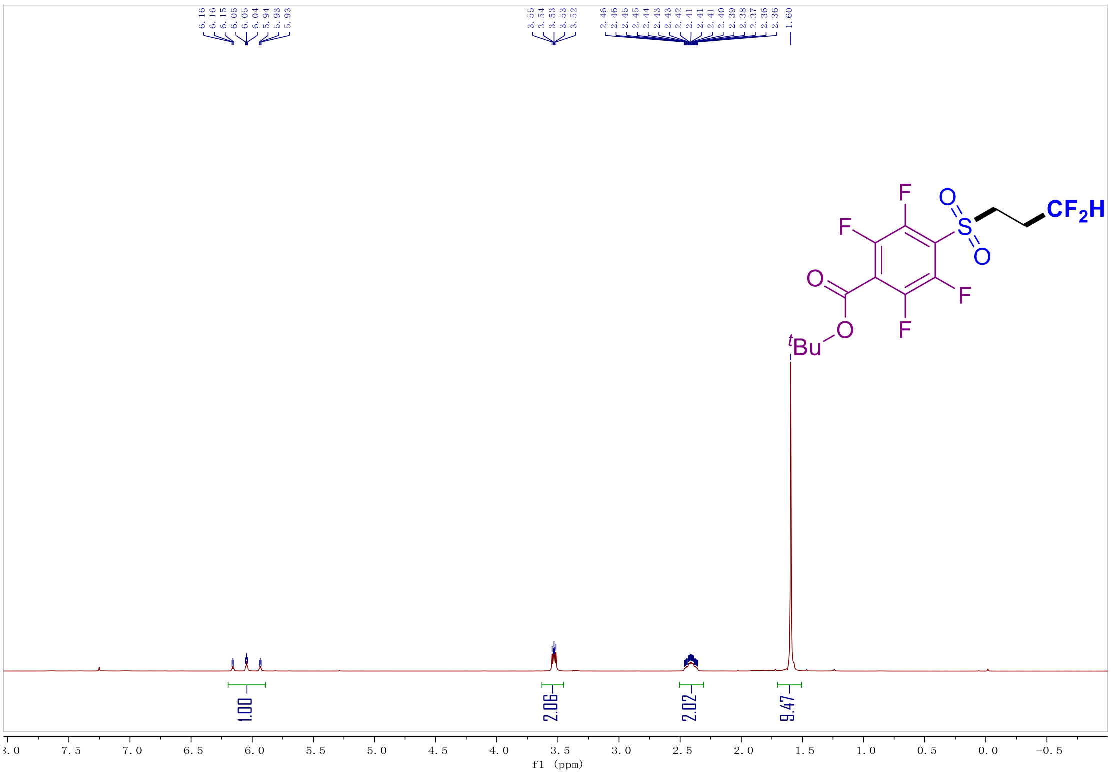

^{13}C NMR (151 MHz, CDCl$_3$) spectrum of 53

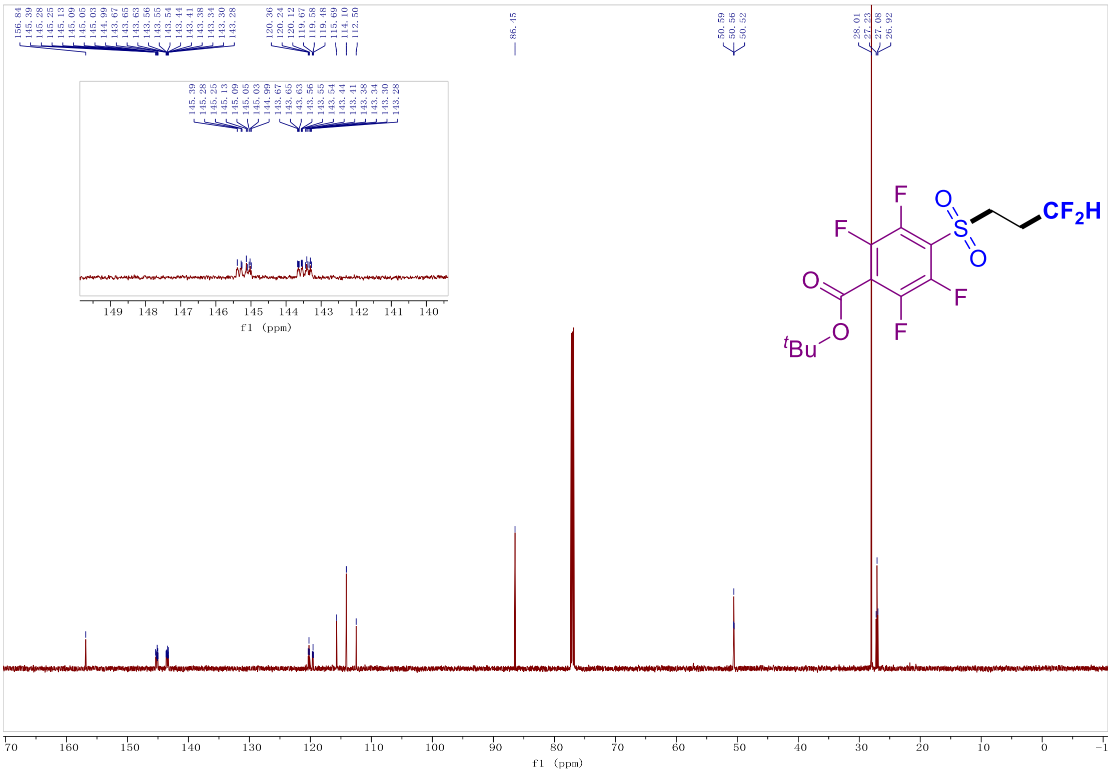

^{19}F NMR (565 MHz, CDCl$_3$) spectrum of 53

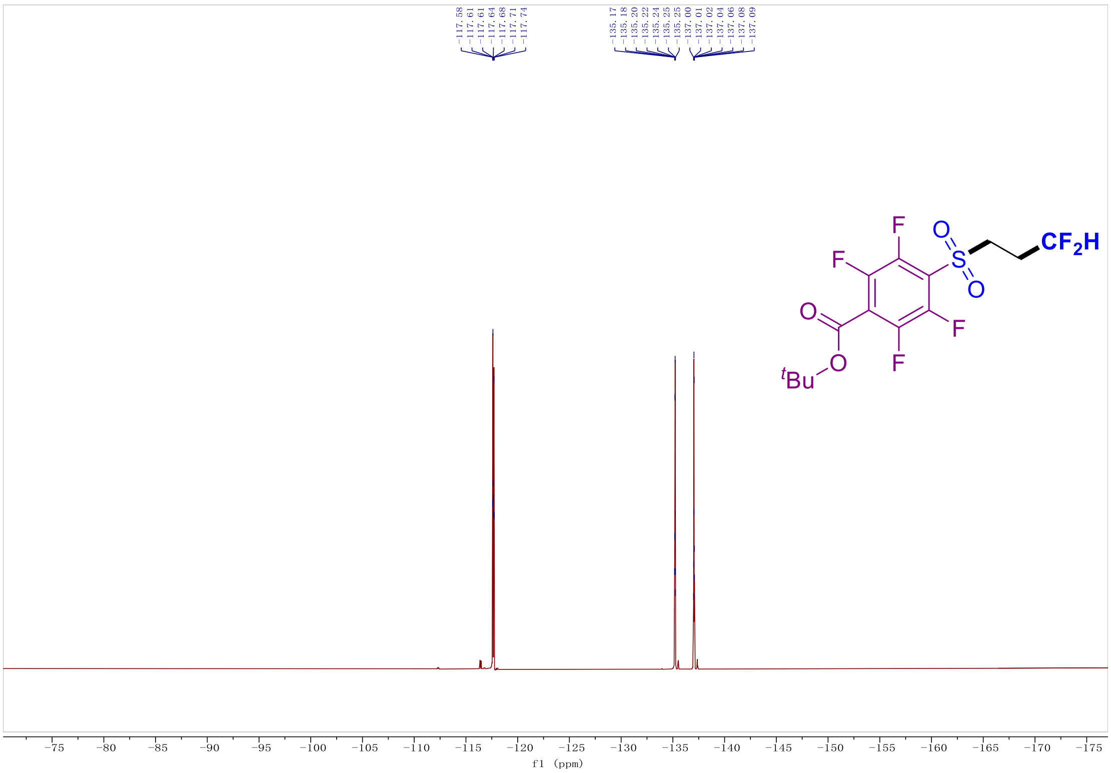

^{1}H NMR (500 MHz, $CDCl_3$) spectrum of 54

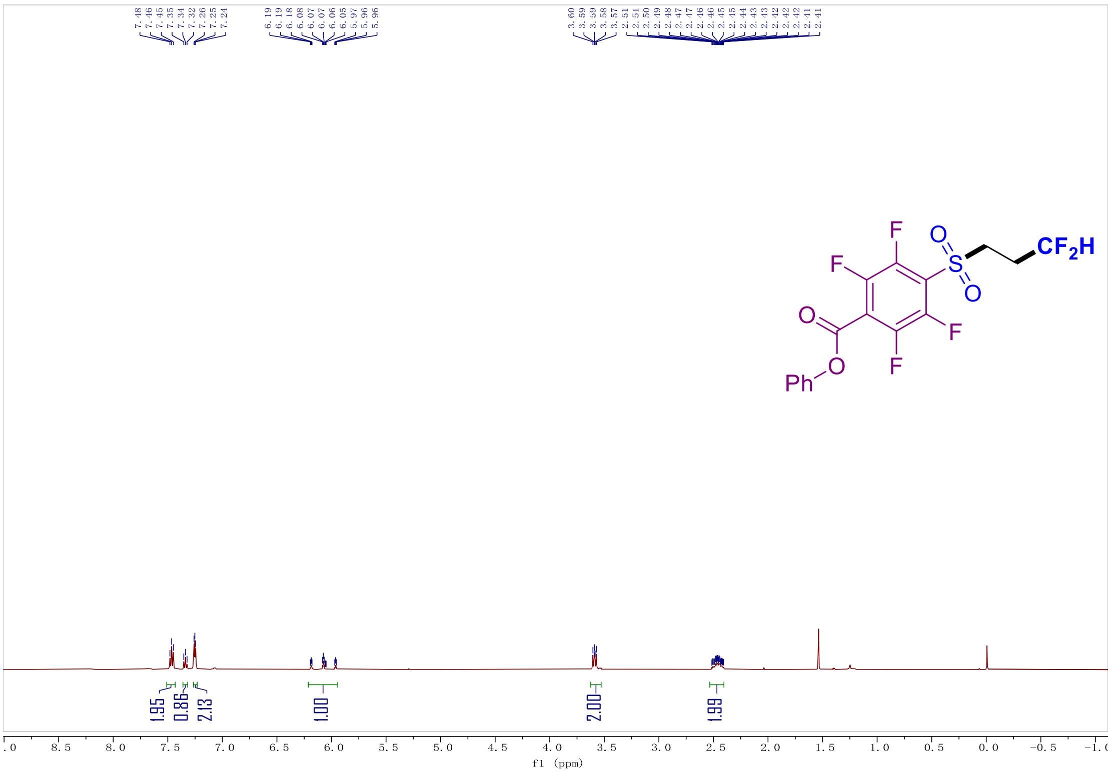

^{13}C NMR (151 MHz, $CDCl_3$) spectrum of 54

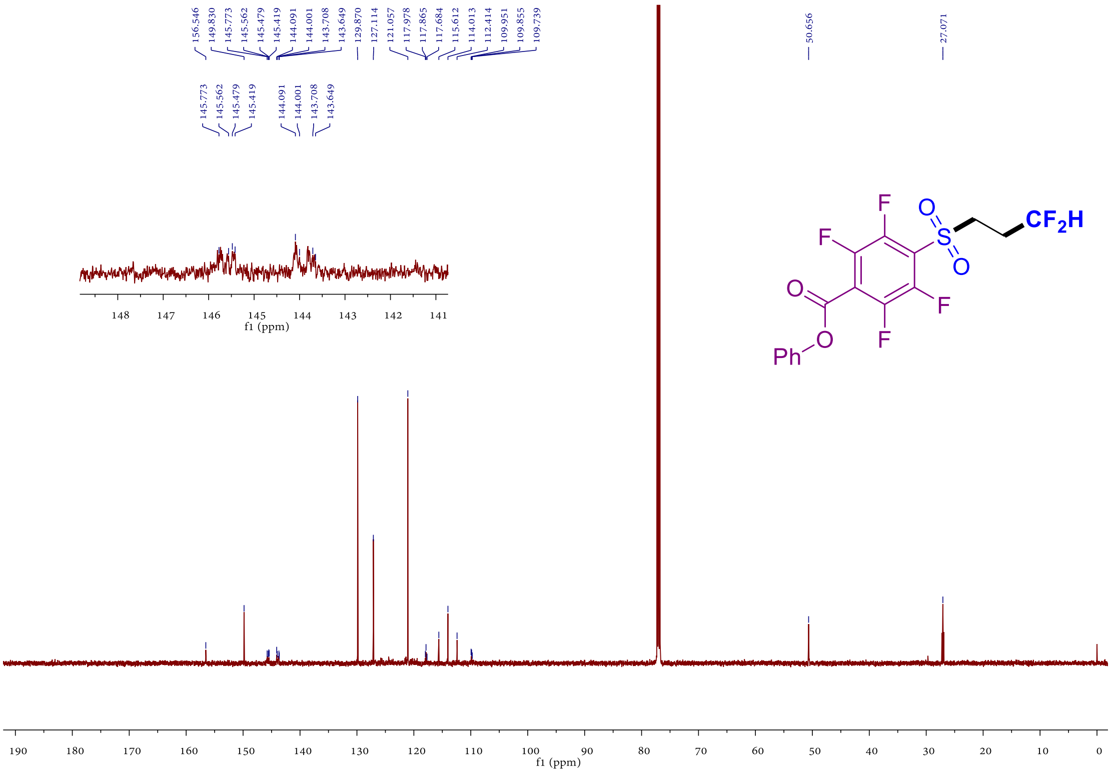

^{19}F NMR (565 MHz, CDCl$_3$) spectrum of 54

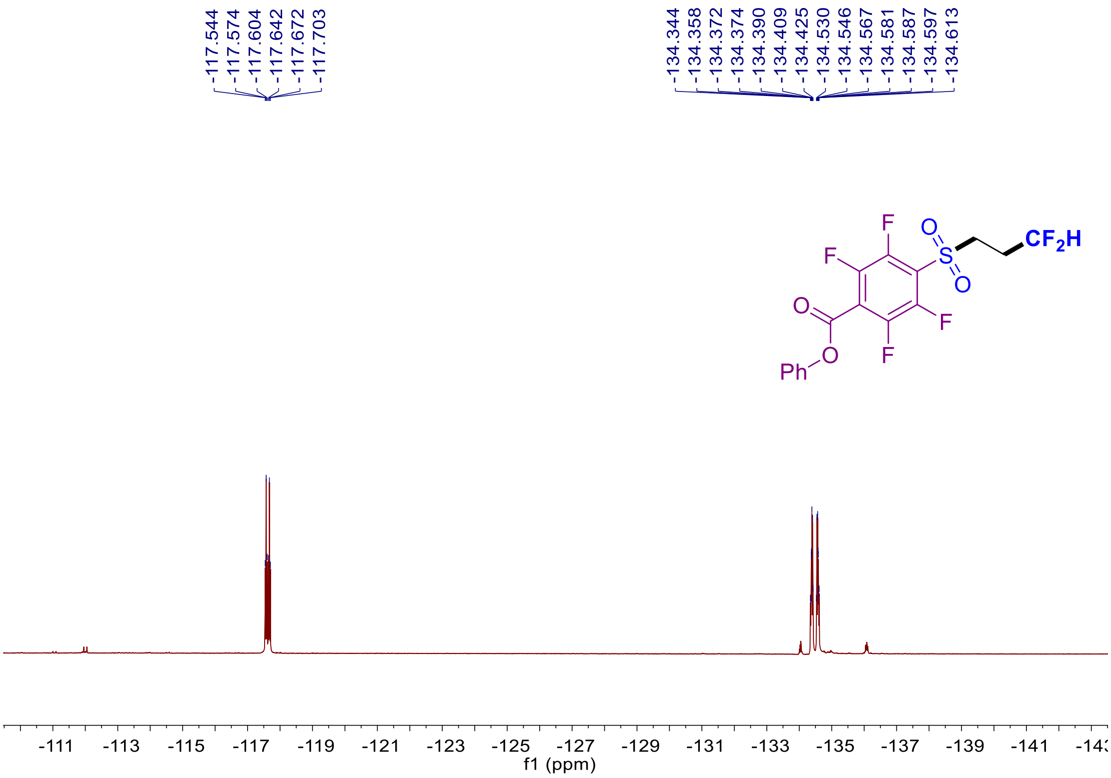

^{1}H NMR (500 MHz, CDCl$_3$) spectrum of 55

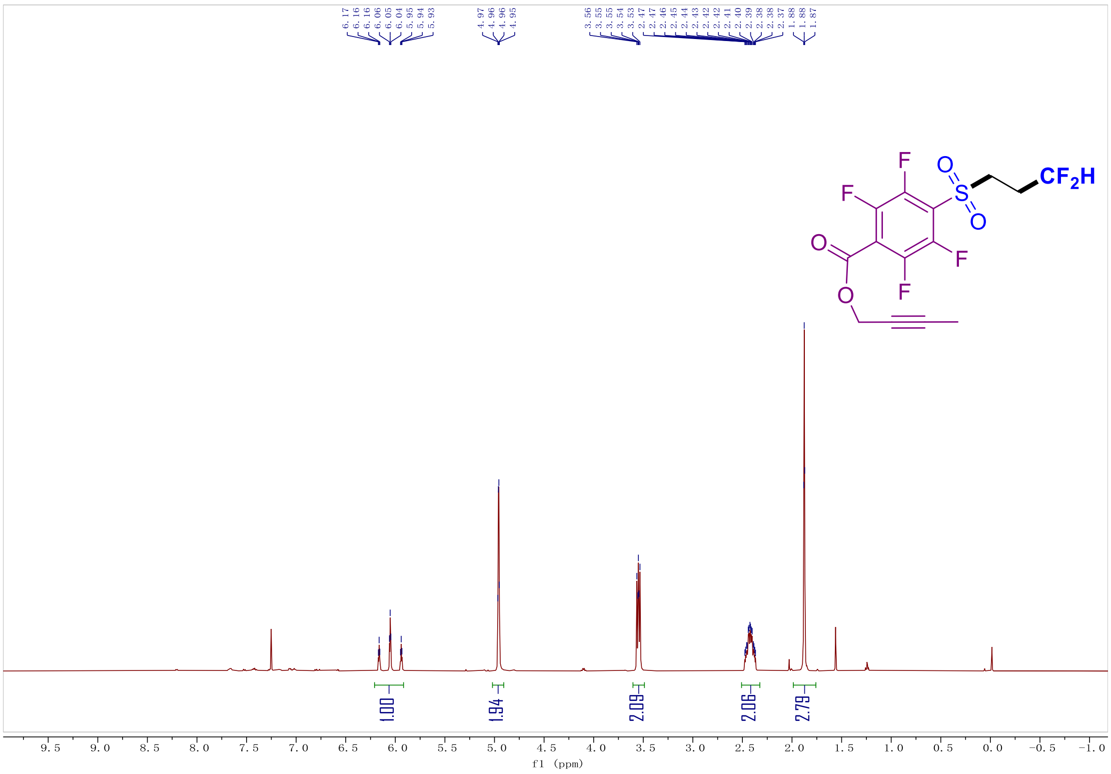

^{13}C NMR (151 MHz, $CDCl_3$) spectrum of 55

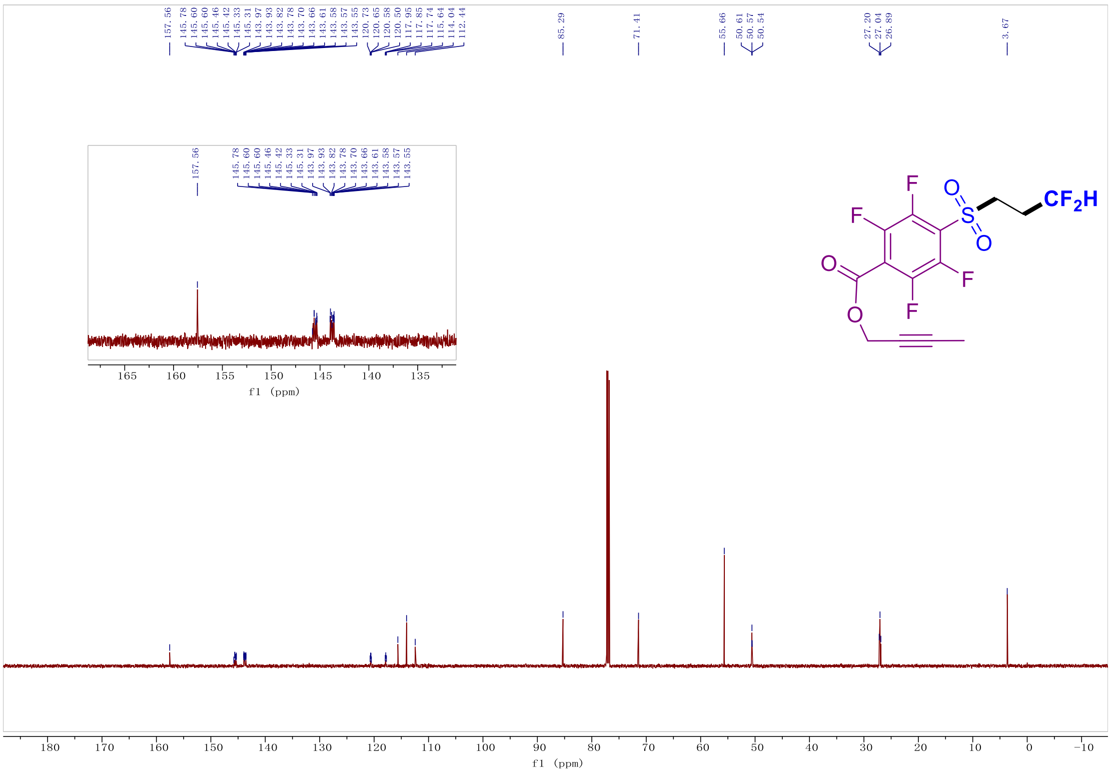

^{19}F NMR (565 MHz, $CDCl_3$) spectrum of 55

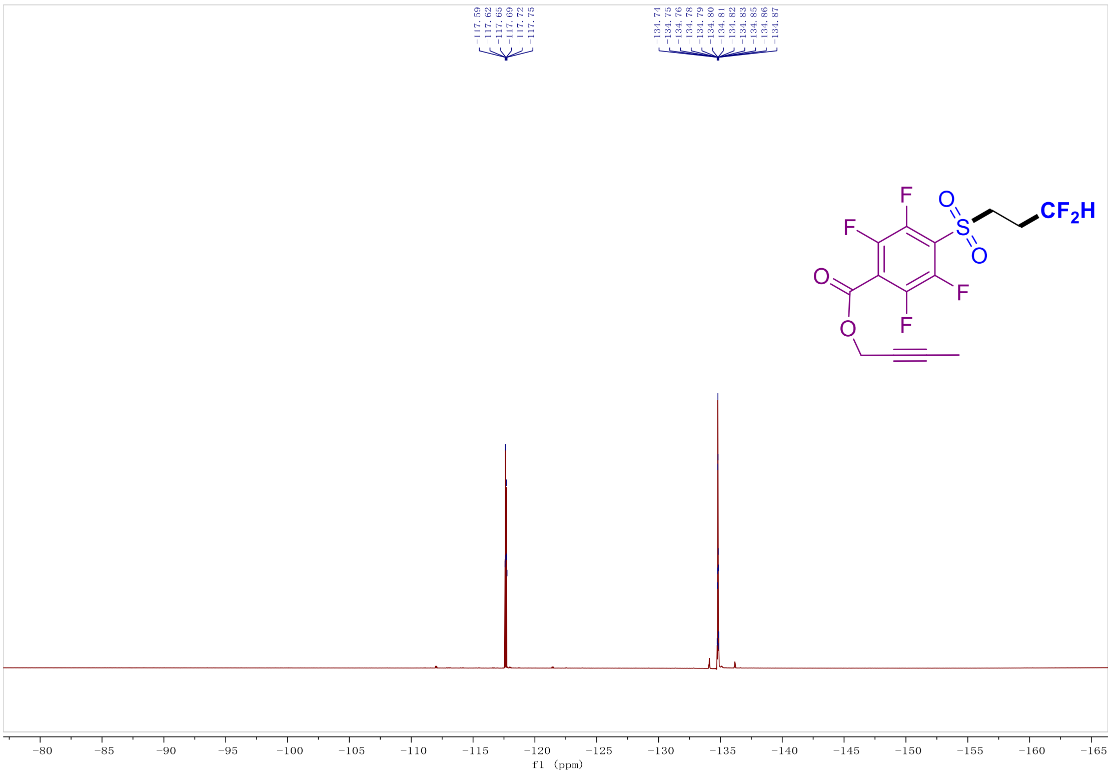

^{1}H NMR (500 MHz, CDCl₃) spectrum of 56

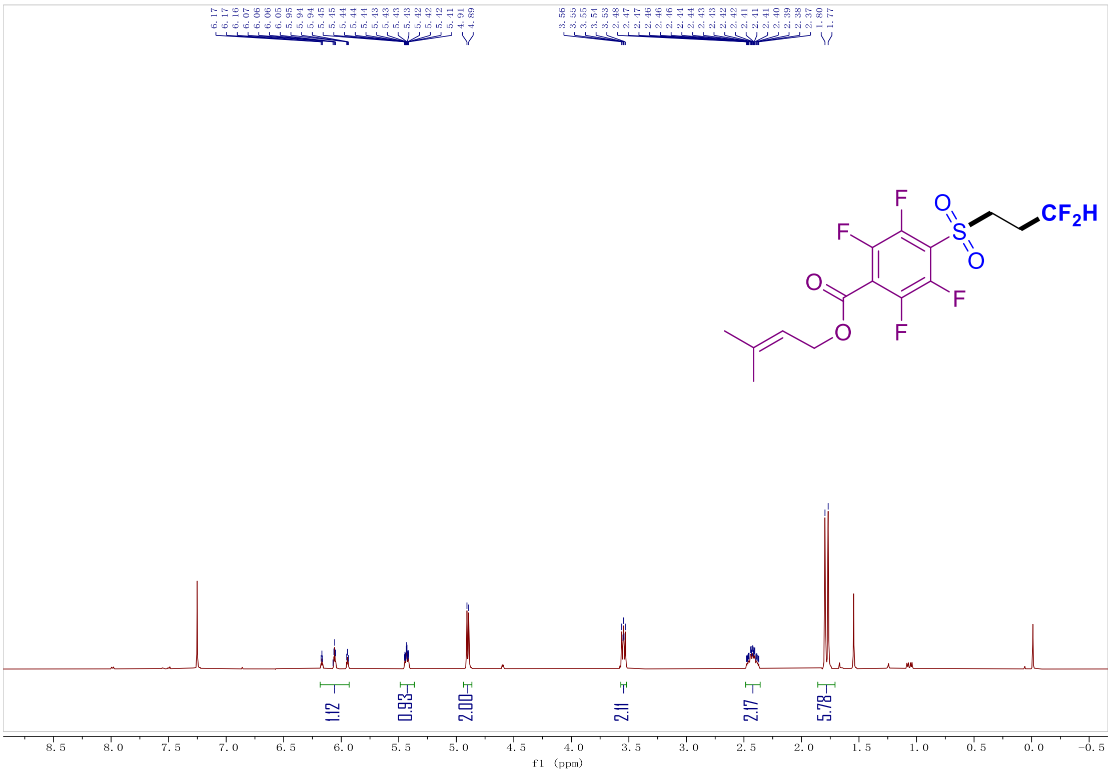

^{13}C NMR (151 MHz, CDCl₃) spectrum of 56

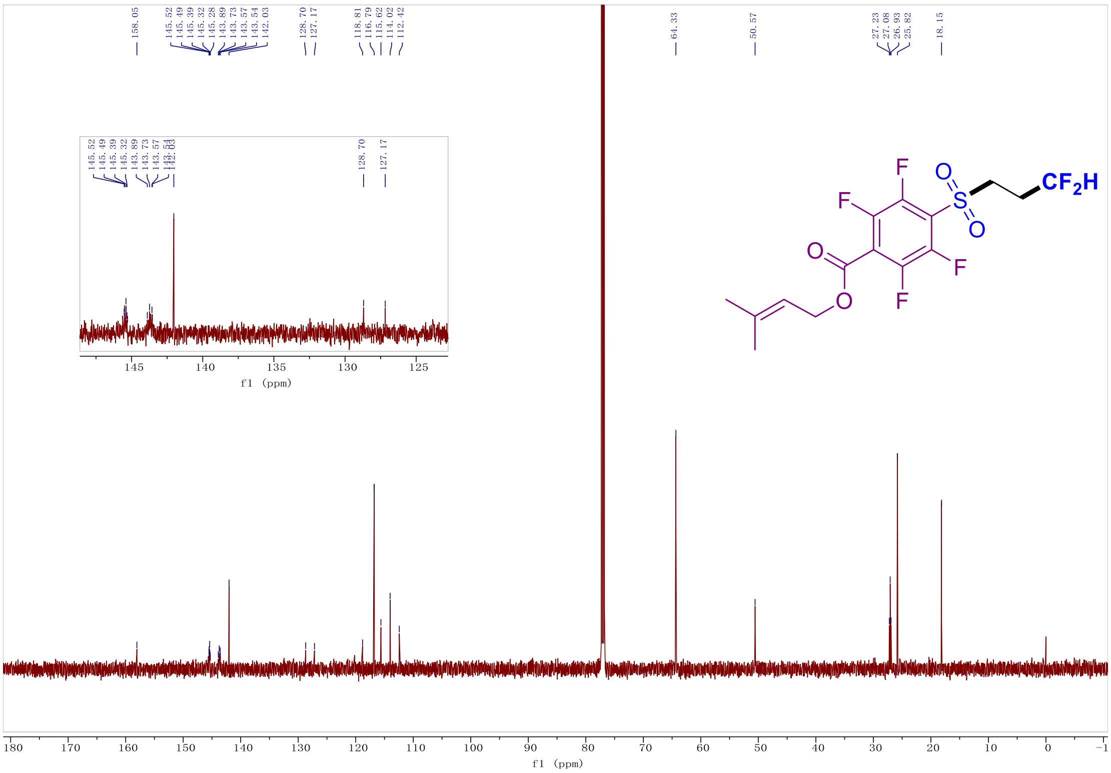

^{19}F NMR (565 MHz, $CDCl_3$) spectrum of 56

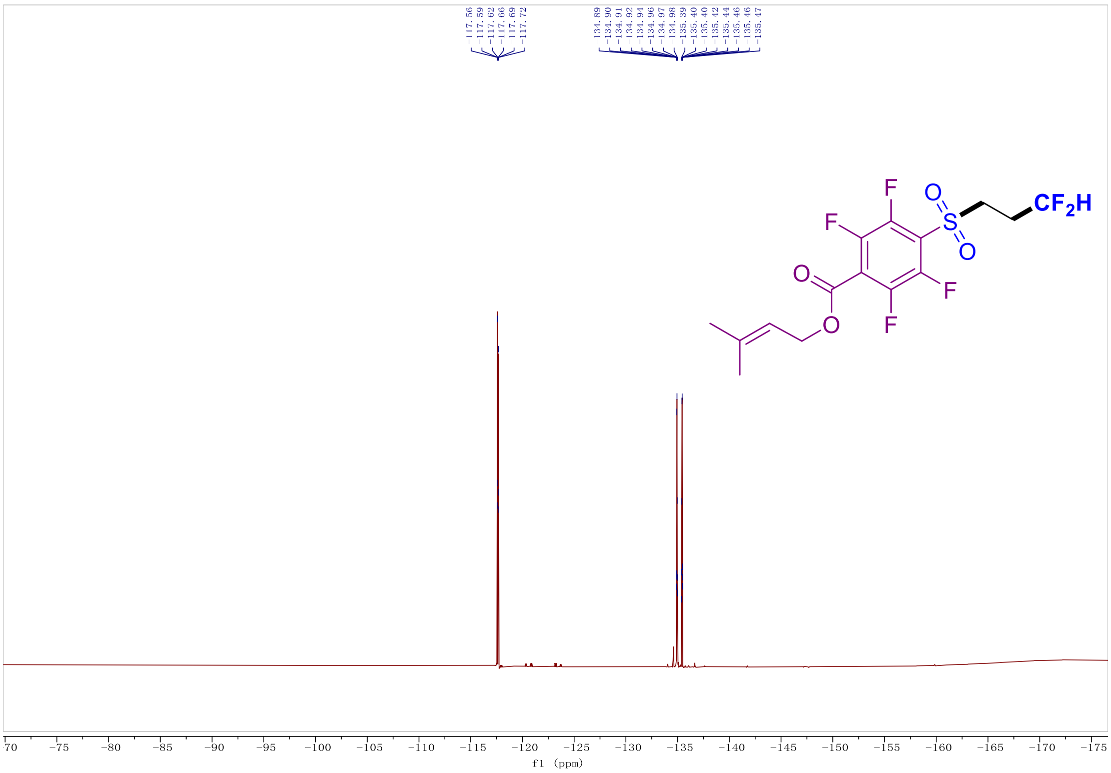

^{1}H NMR (500 MHz, $CDCl_3$) spectrum of 57

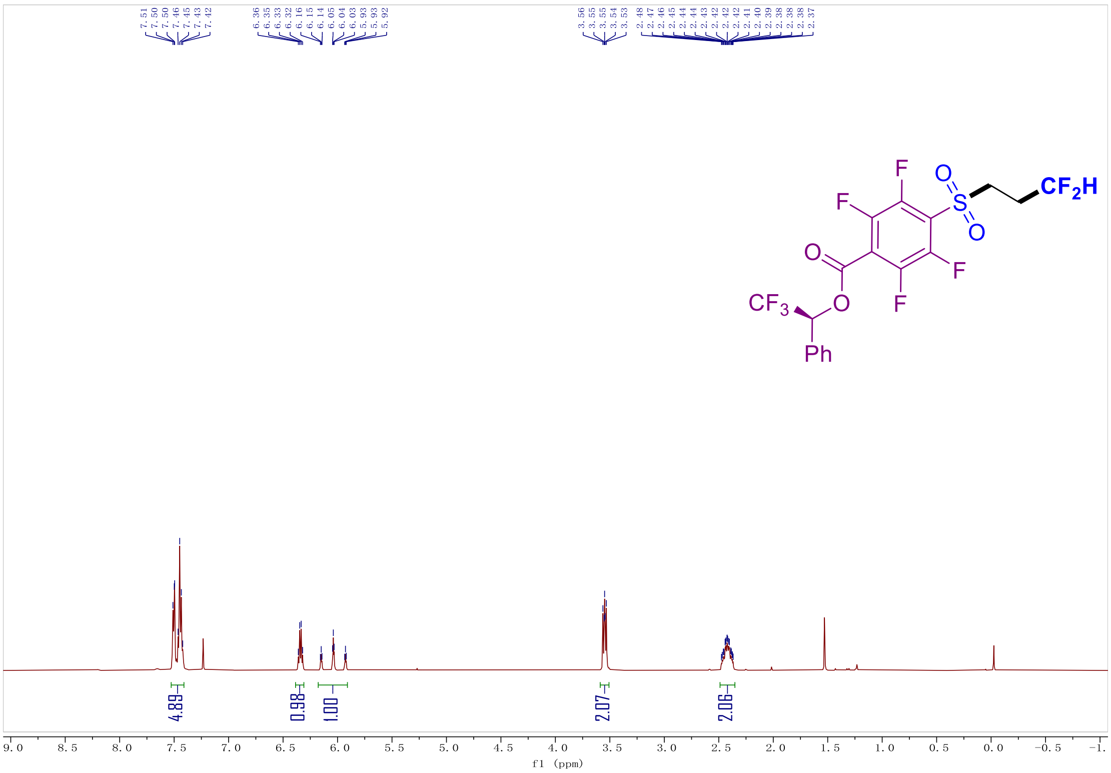

^{13}C NMR (151 MHz, $CDCl_3$) spectrum of 57

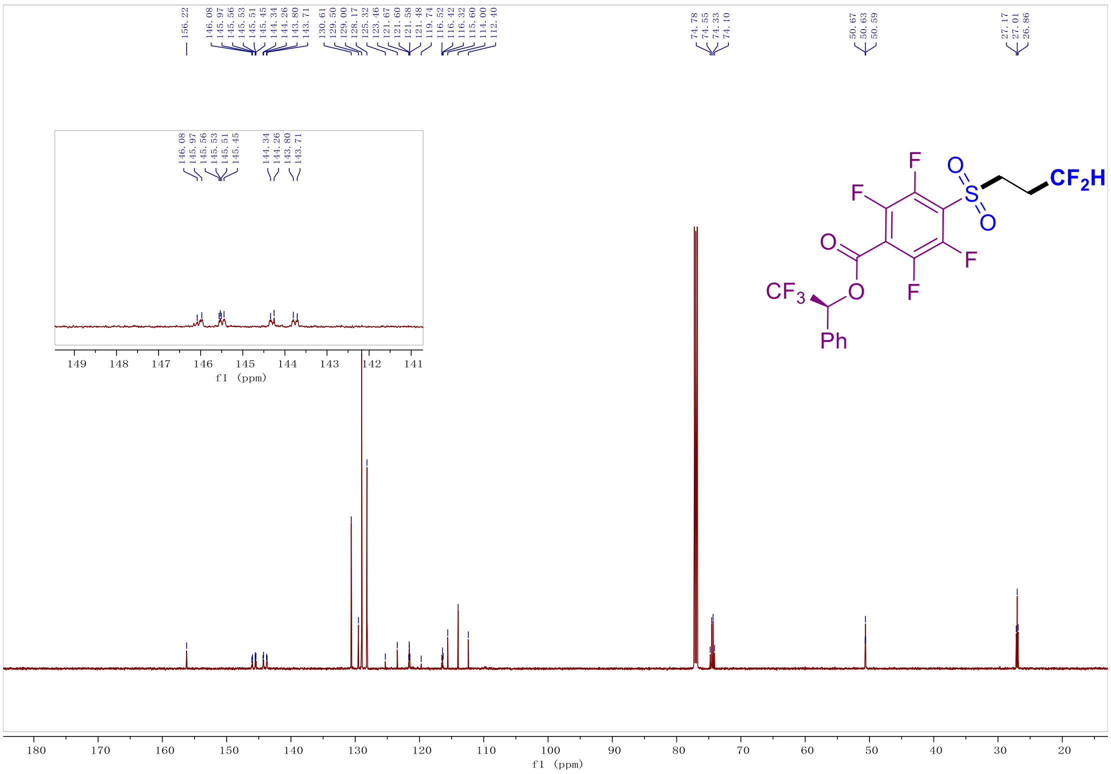

^{19}F NMR (565 MHz, $CDCl_3$) spectrum of 57

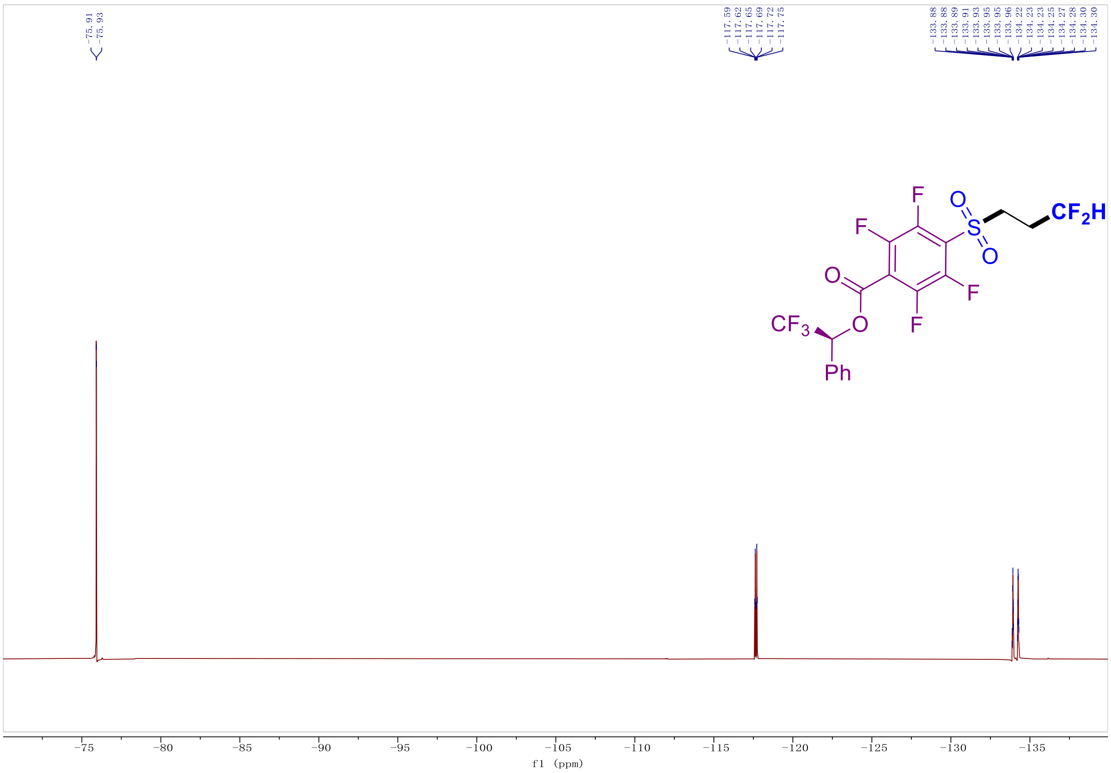

^{1}H NMR (500 MHz, CDCl$_3$) spectrum of 58

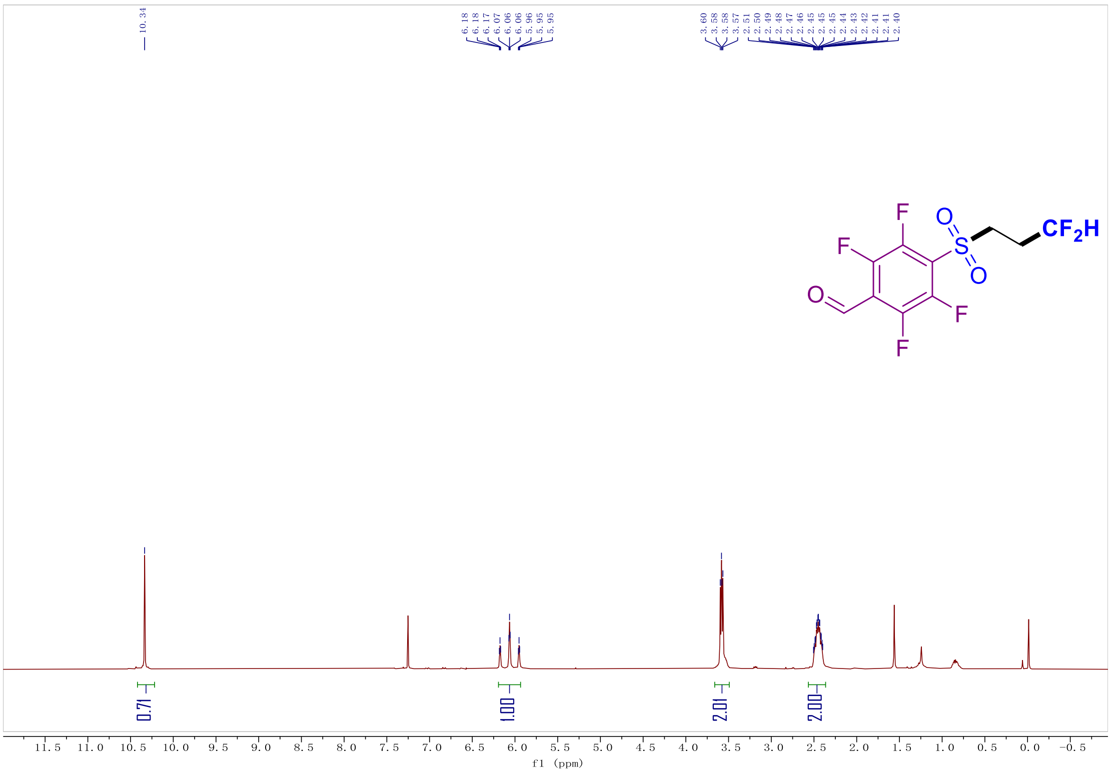

^{13}C NMR (151 MHz, CDCl$_3$) spectrum of 58

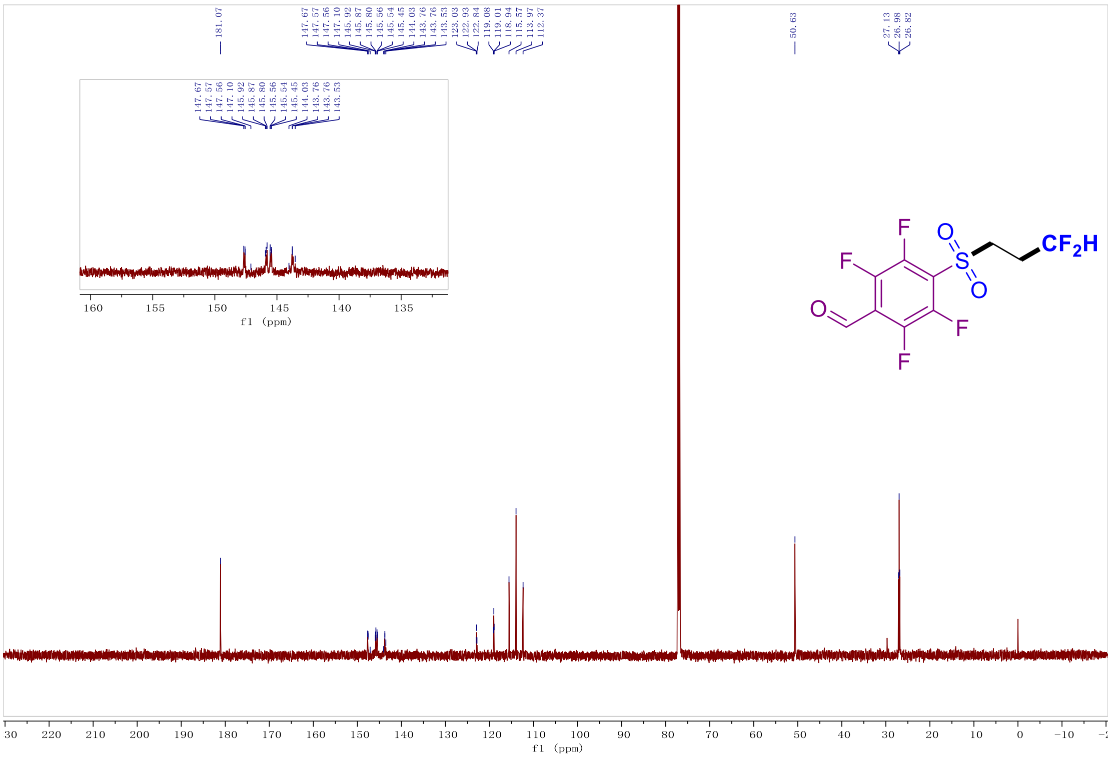

^{19}F NMR (565 MHz, CDCl$_3$) spectrum of 58

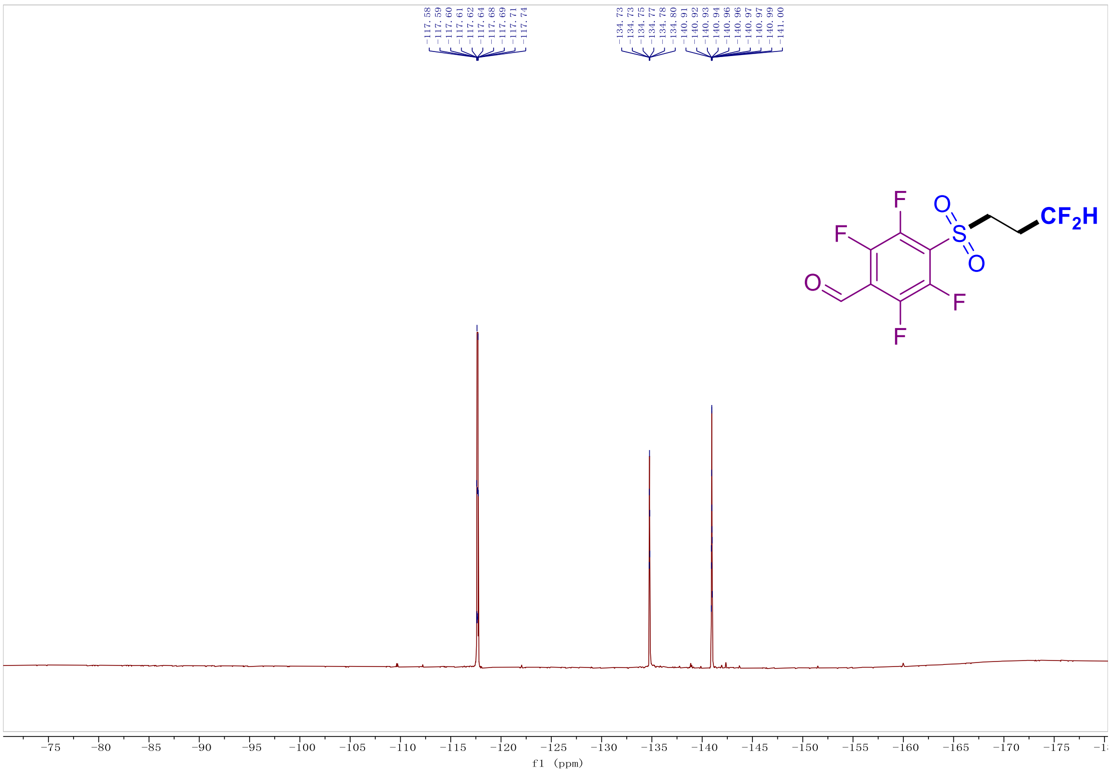

^{1}H NMR (500 MHz, CDCl$_3$) spectrum of 59

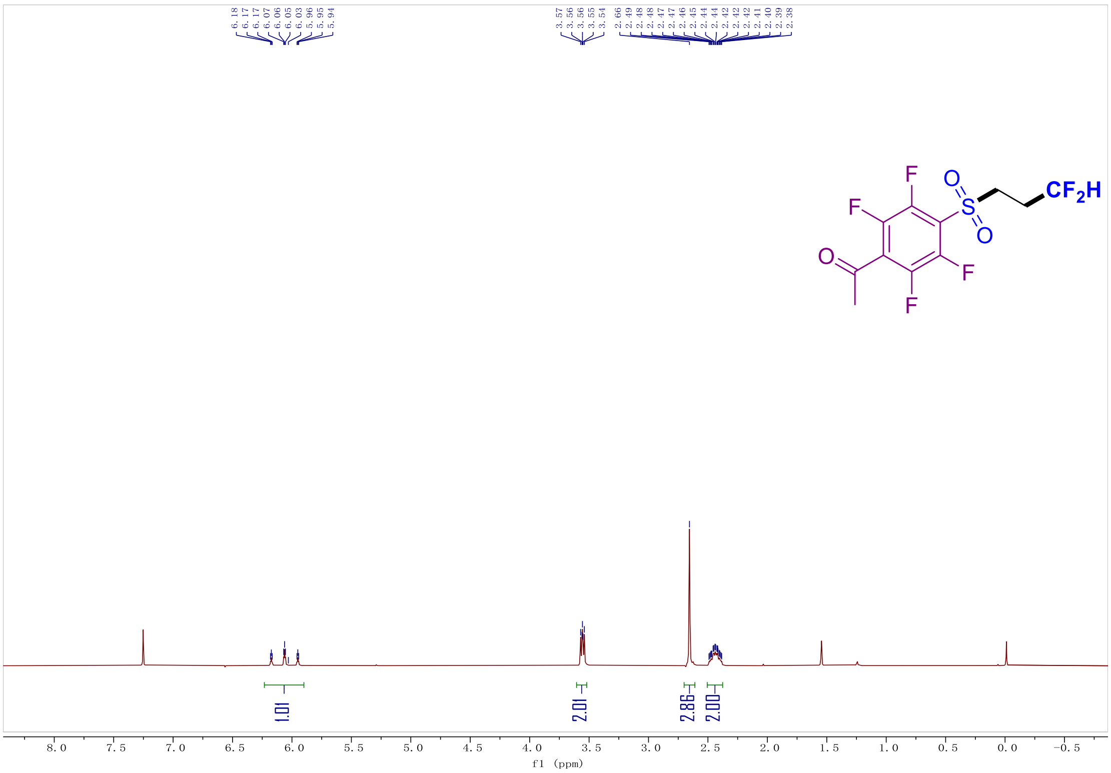

^{13}C NMR (151 MHz, CDCl$_3$) spectrum of 59

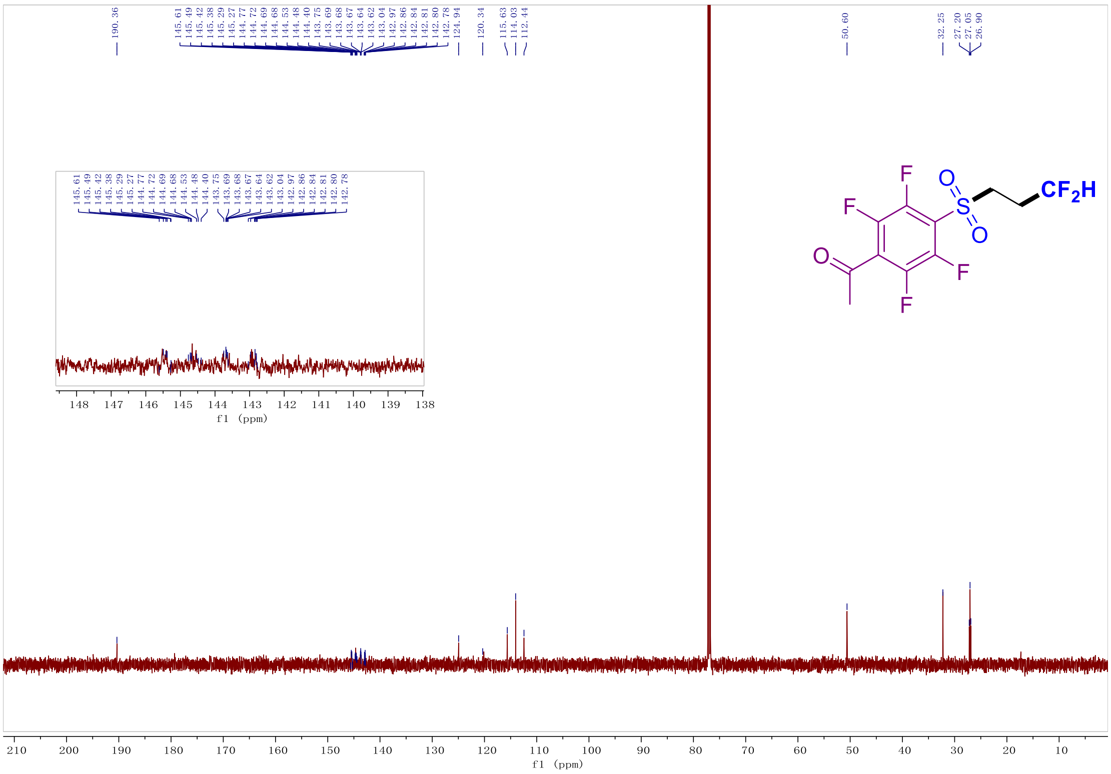

^{19}F NMR (565 MHz, CDCl$_3$) spectrum of 59

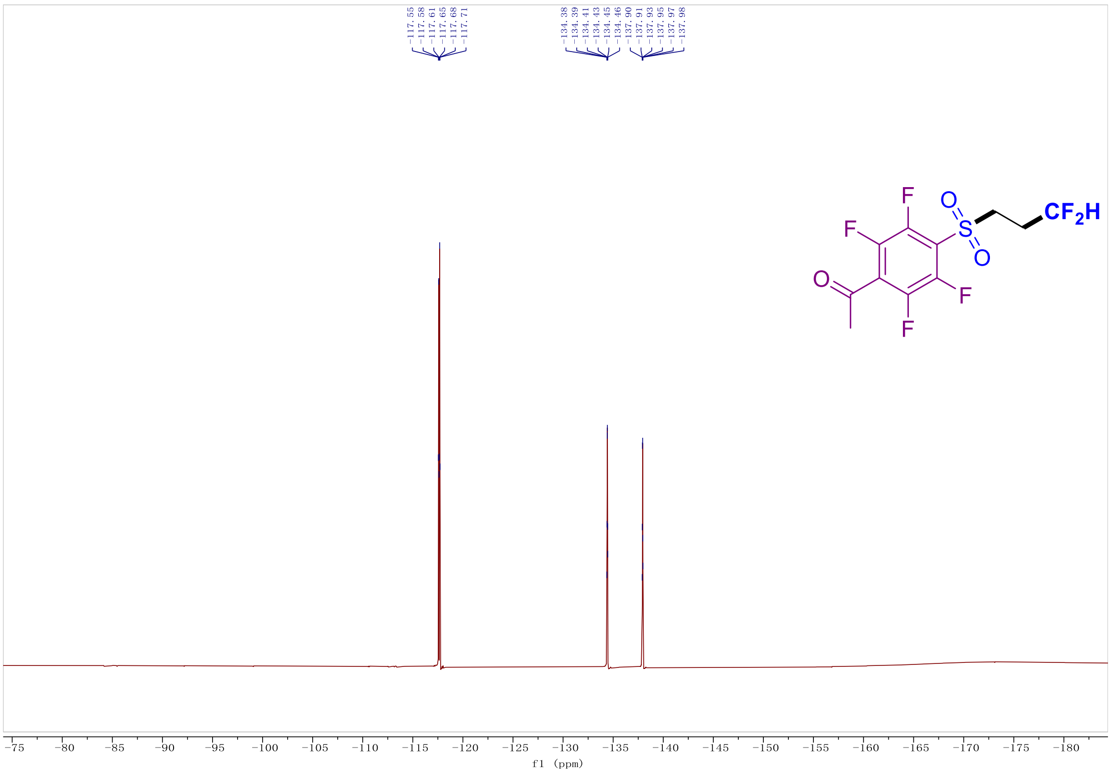

^{1}H NMR (500 MHz, CDCl3) spectrum of 60

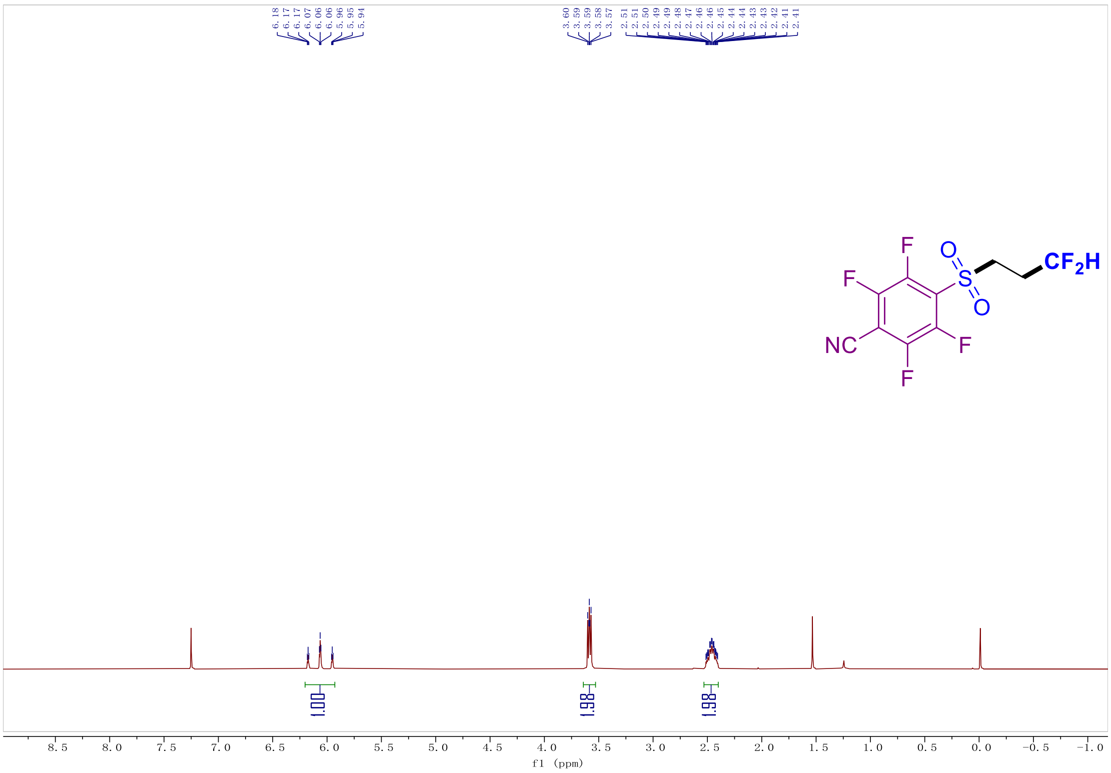

^{13}C NMR (151 MHz, CDCl3) spectrum of 60

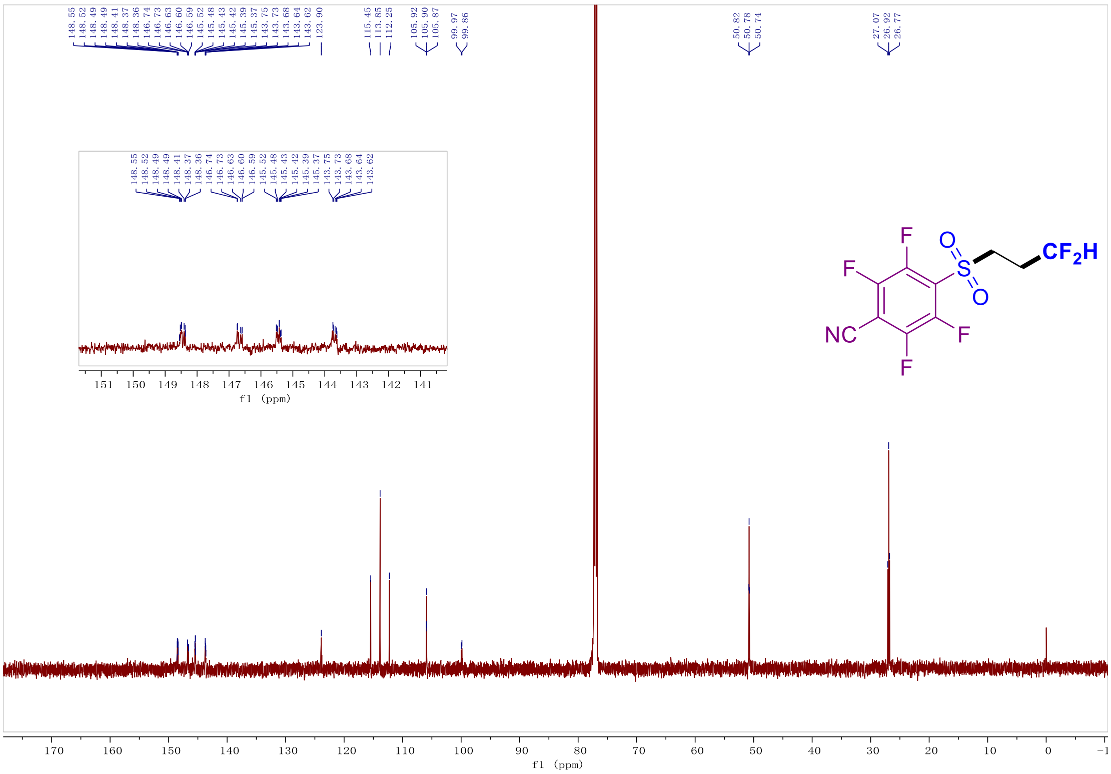

^{19}F NMR (565 MHz, $CDCl_3$) spectrum of 60

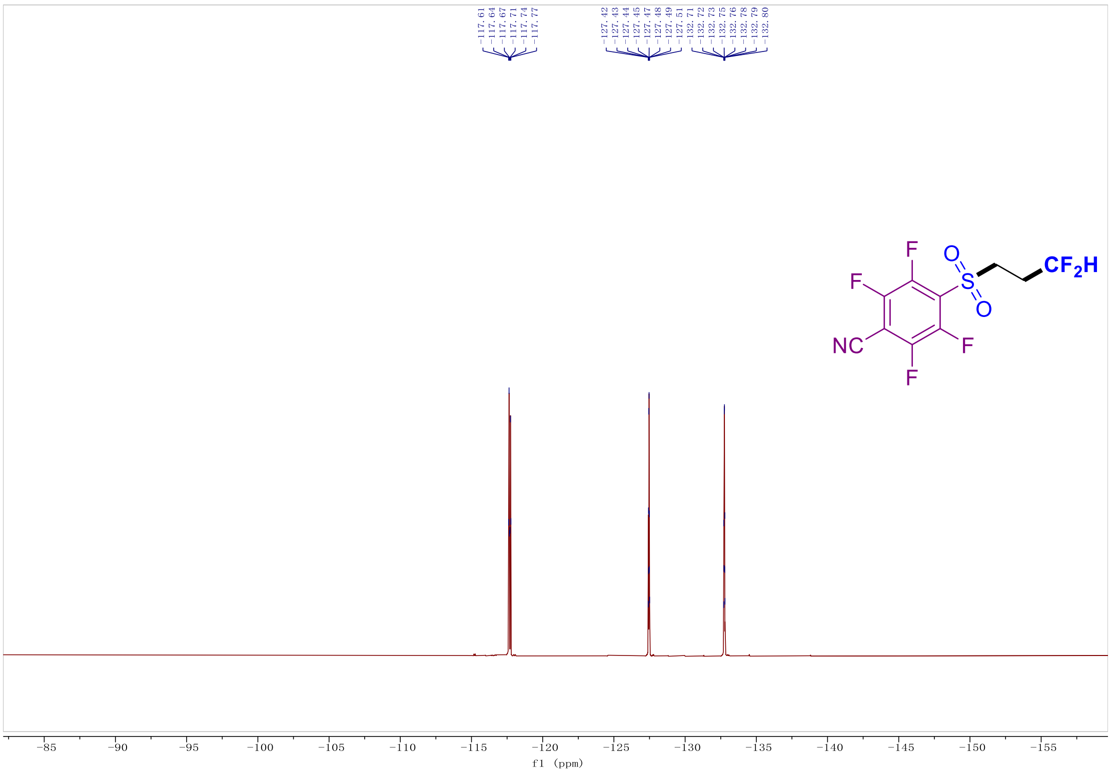

^{1}H NMR (500 MHz, $CDCl_3$) spectrum of 61

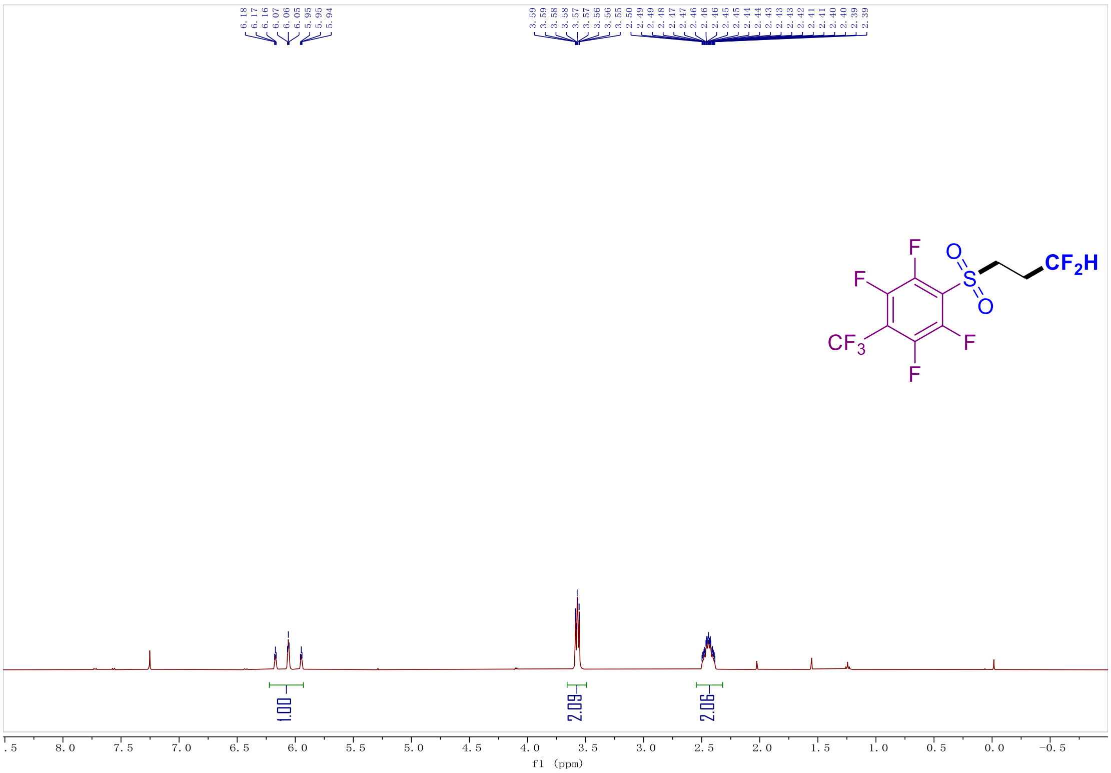

^{13}C NMR (151 MHz, $CDCl_3$) spectrum of 61

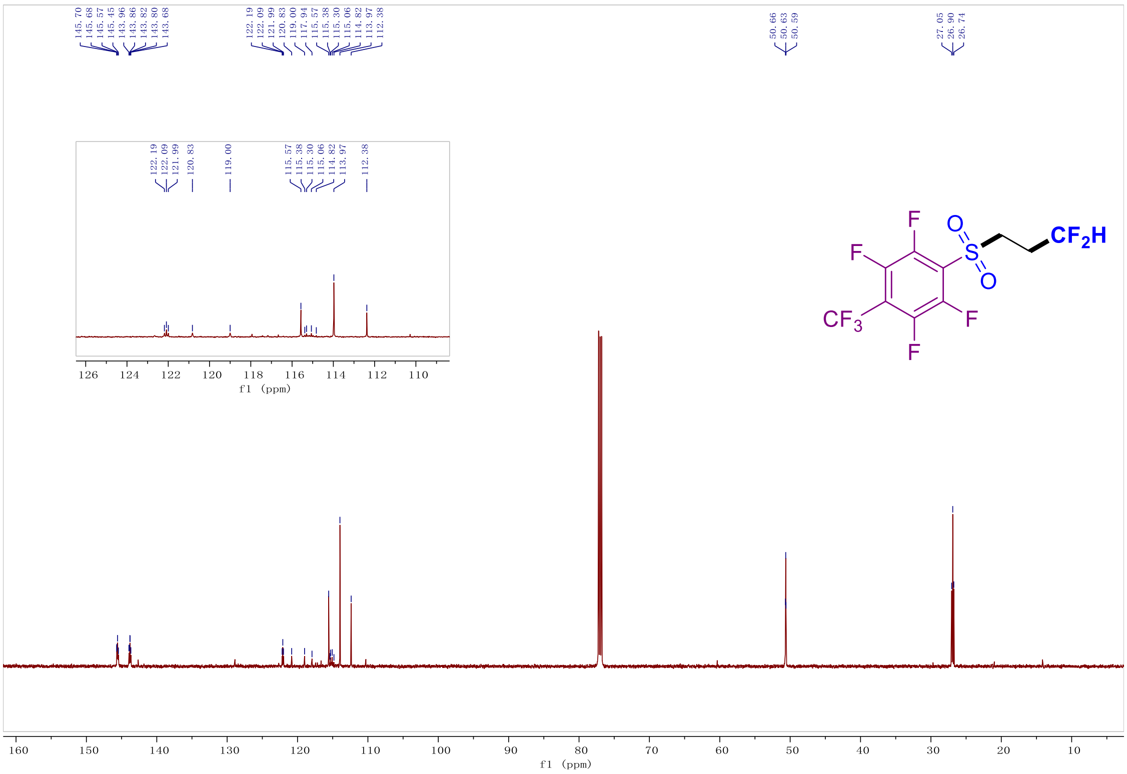

^{19}F NMR (565 MHz, $CDCl_3$) spectrum of 61

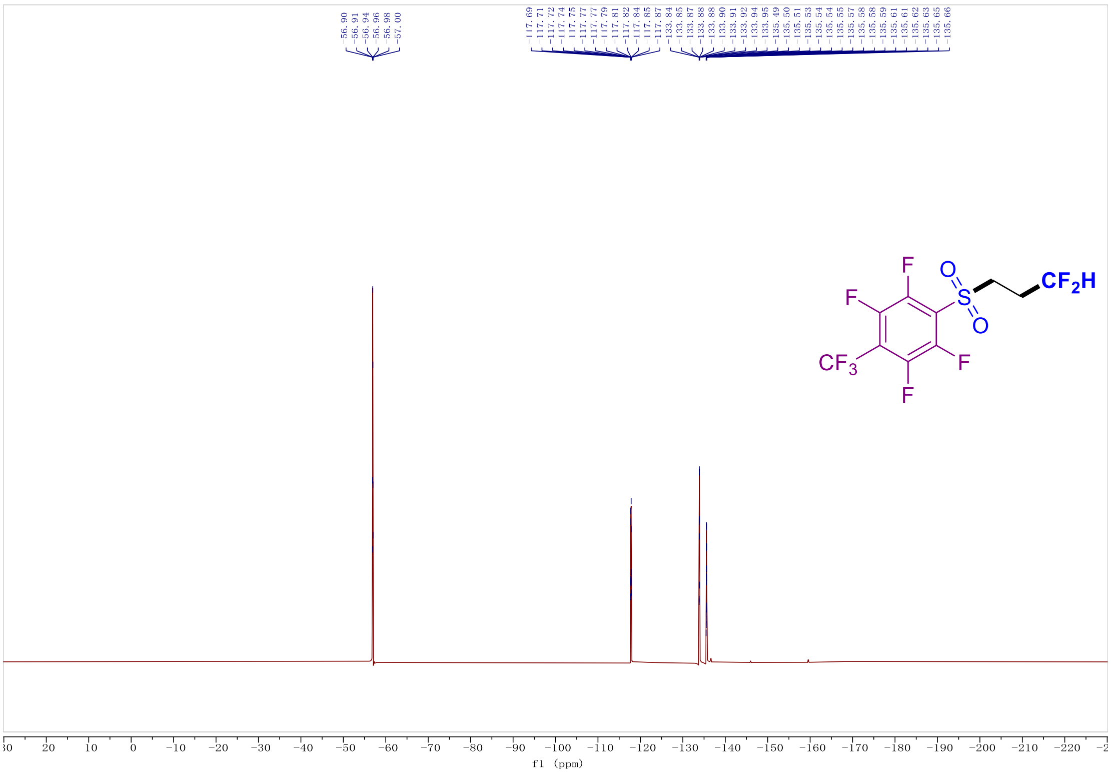

^{1}H NMR (500 MHz, CDCl$_3$) spectrum of 62

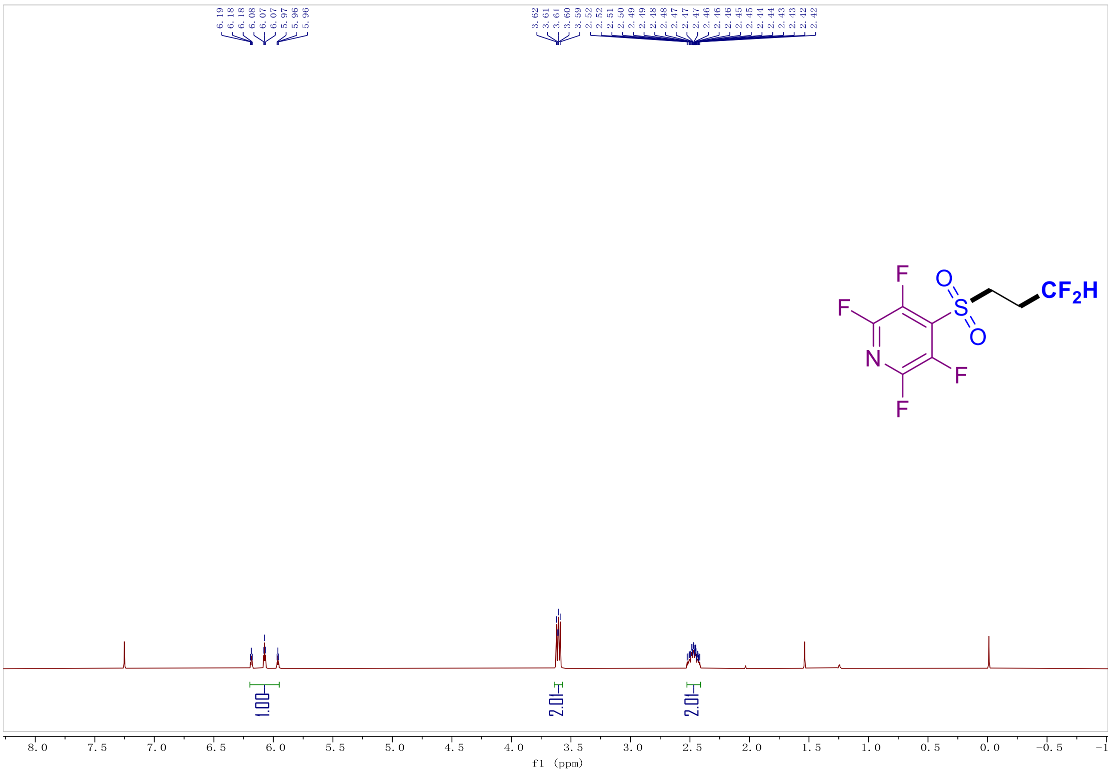

^{13}C NMR (151 MHz, CDCl$_3$) spectrum of 62

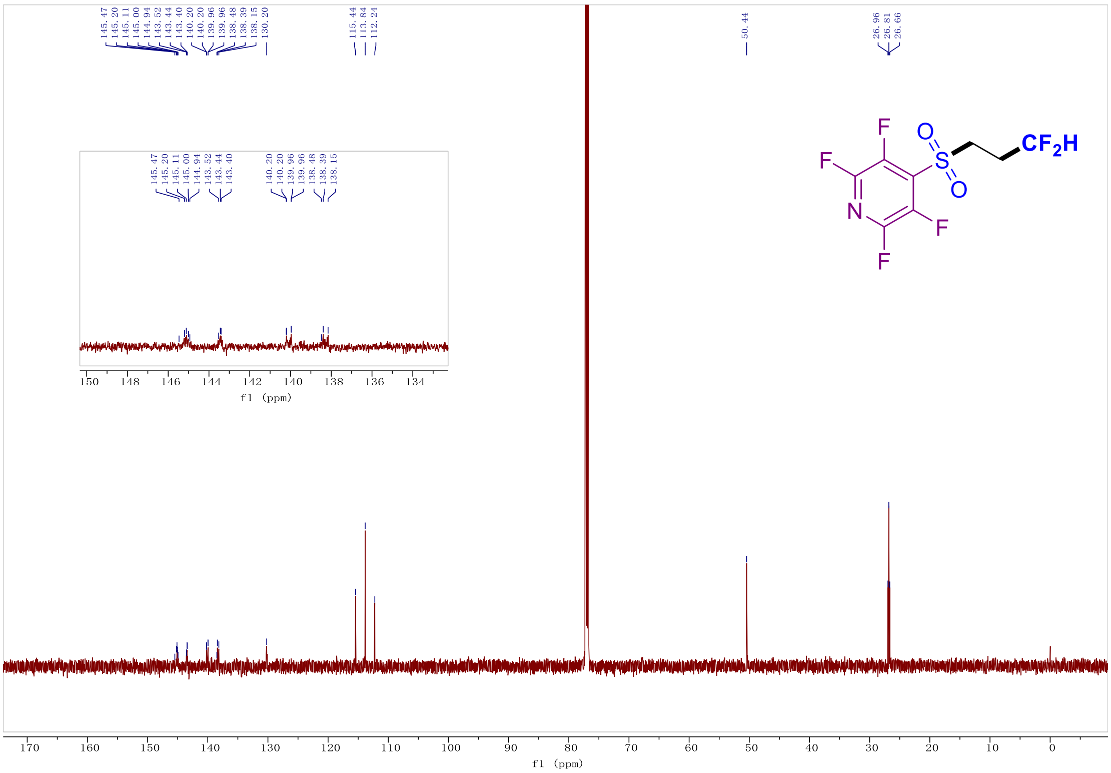

^{19}F NMR (565 MHz, $CDCl_3$) spectrum of 62

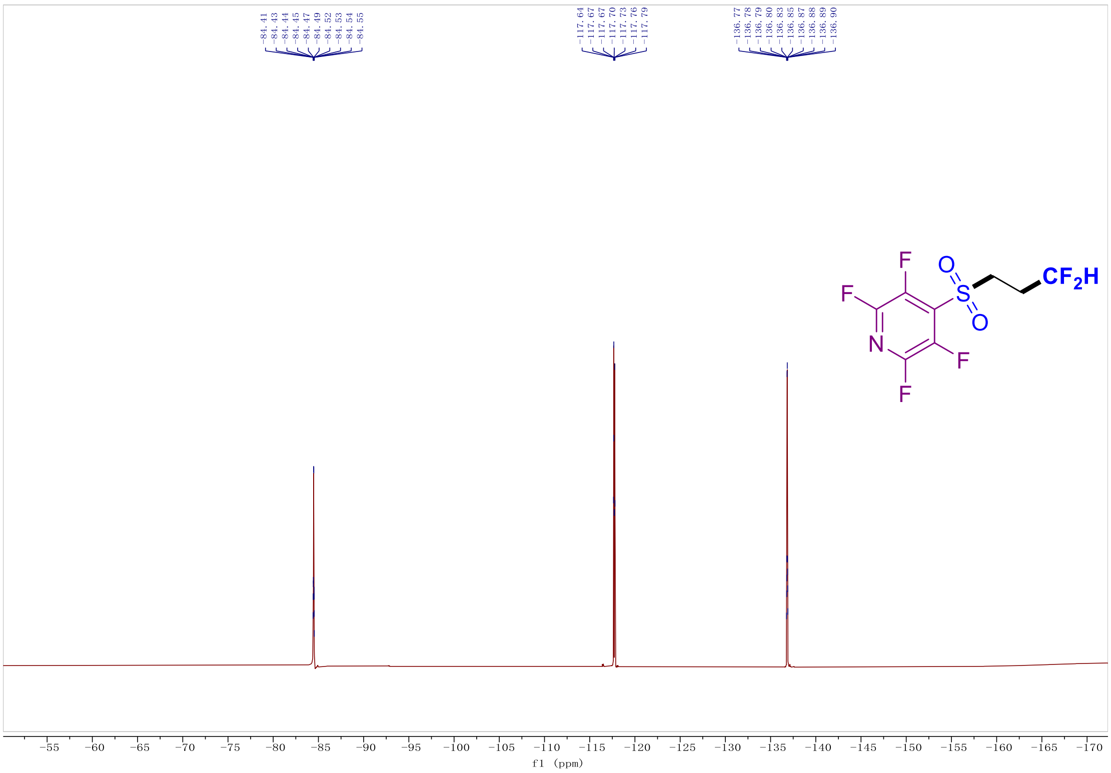

^{1}H NMR (500 MHz, $CDCl_3$) spectrum of 63

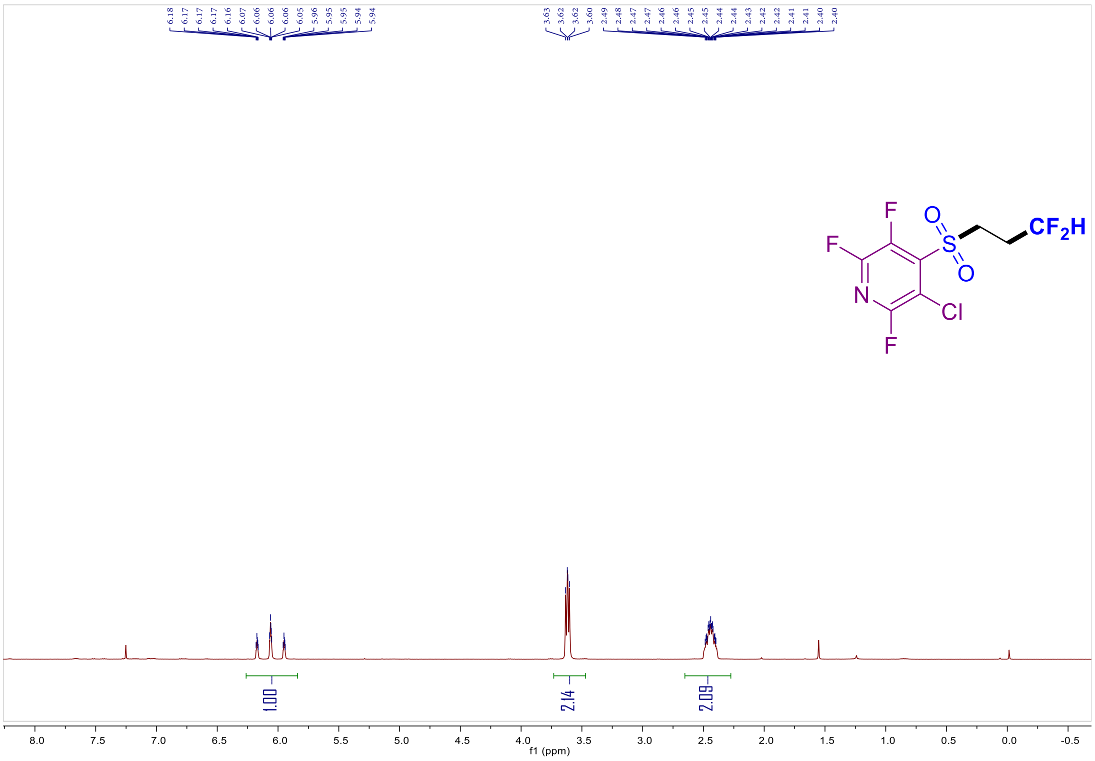

^{13}C NMR (151 MHz, $CDCl_3$) spectrum of 63

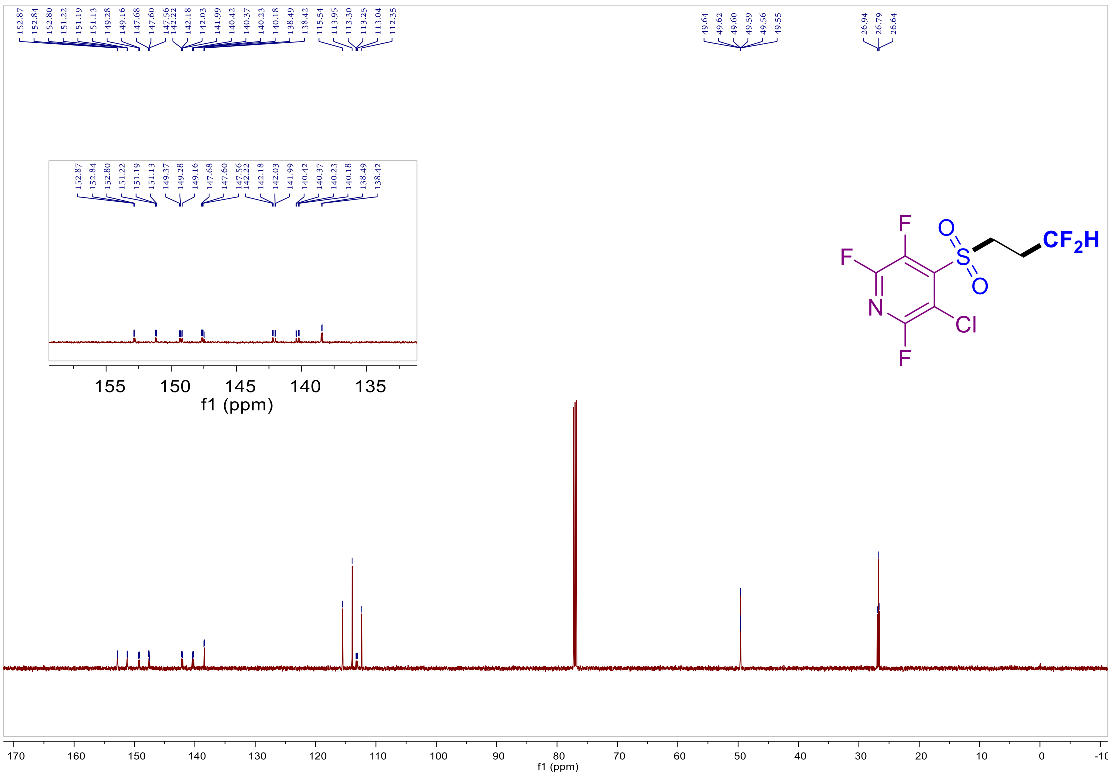

^{19}F NMR (565 MHz, $CDCl_3$) spectrum of 63

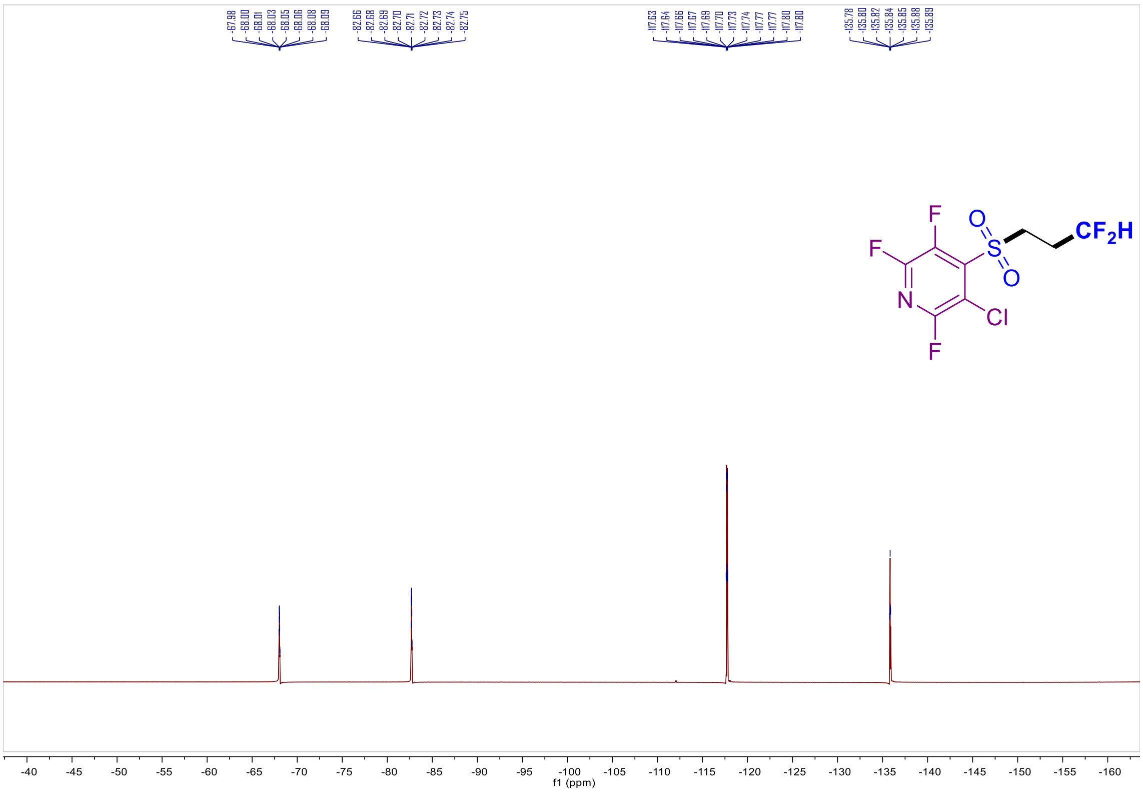

^{1}H NMR (500 MHz, $CDCl_3$) spectrum of 64

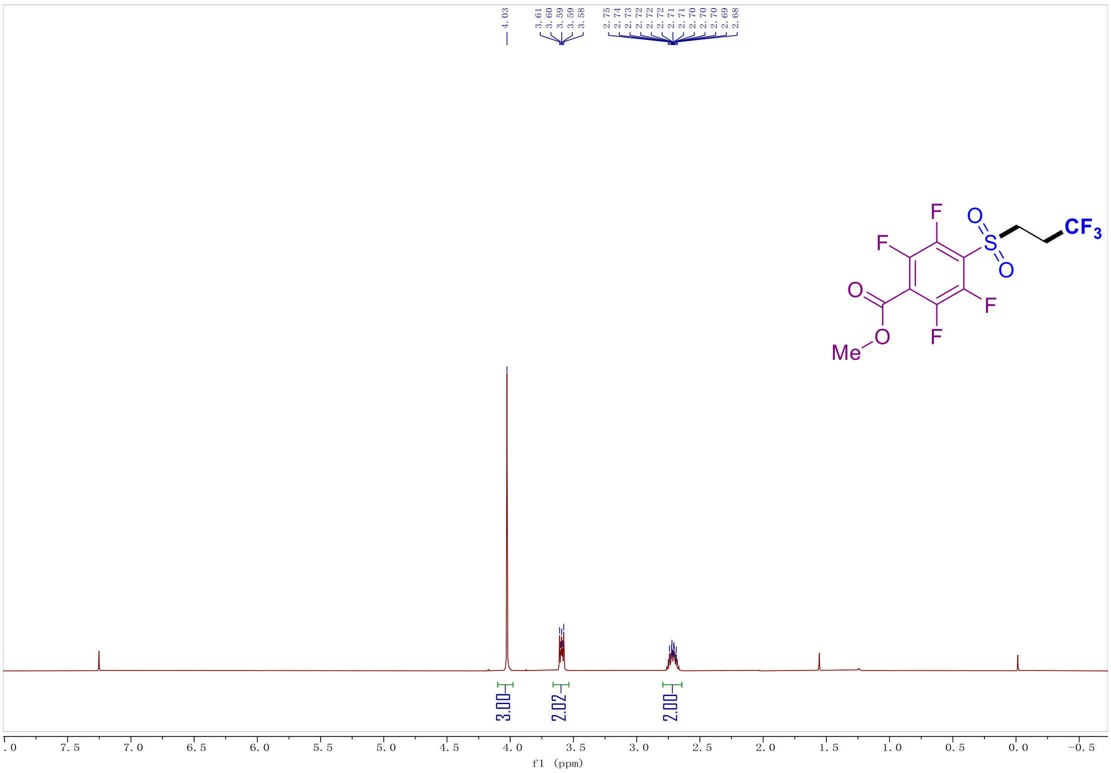

^{13}C NMR (151 MHz, $CDCl_3$) spectrum of 64

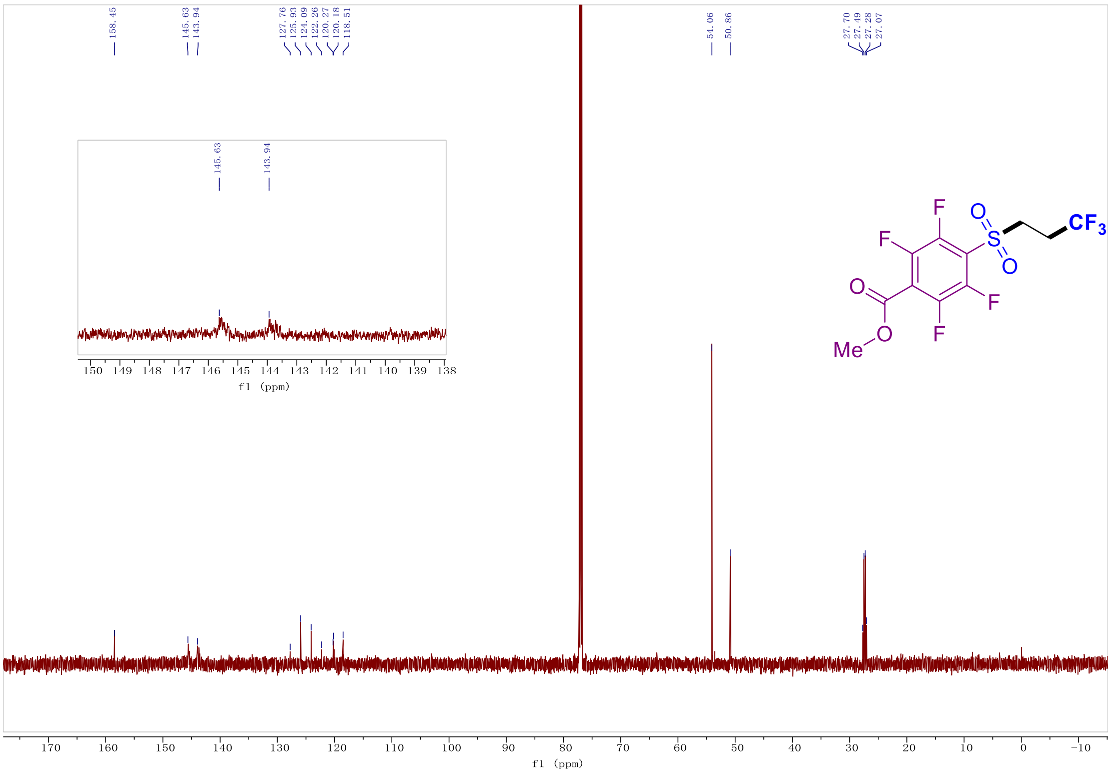

^{19}F NMR (565 MHz, CDCl$_3$) spectrum of 64

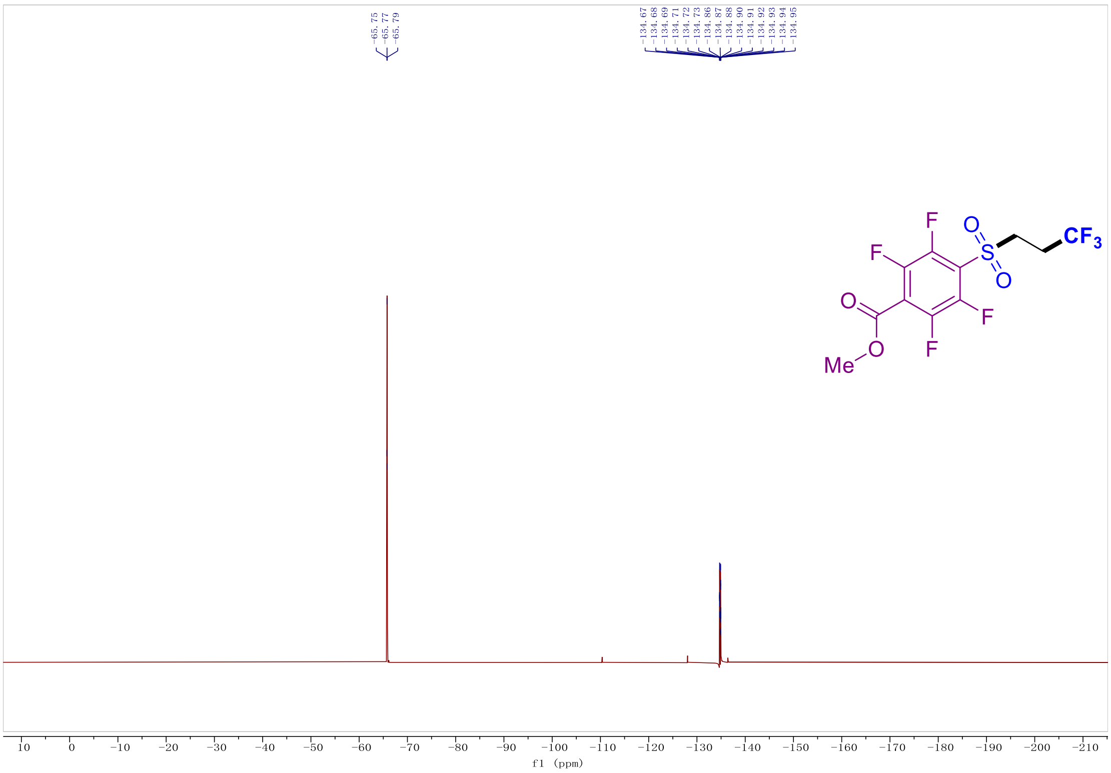

^{1}H NMR (500 MHz, CDCl$_3$) spectrum of 65

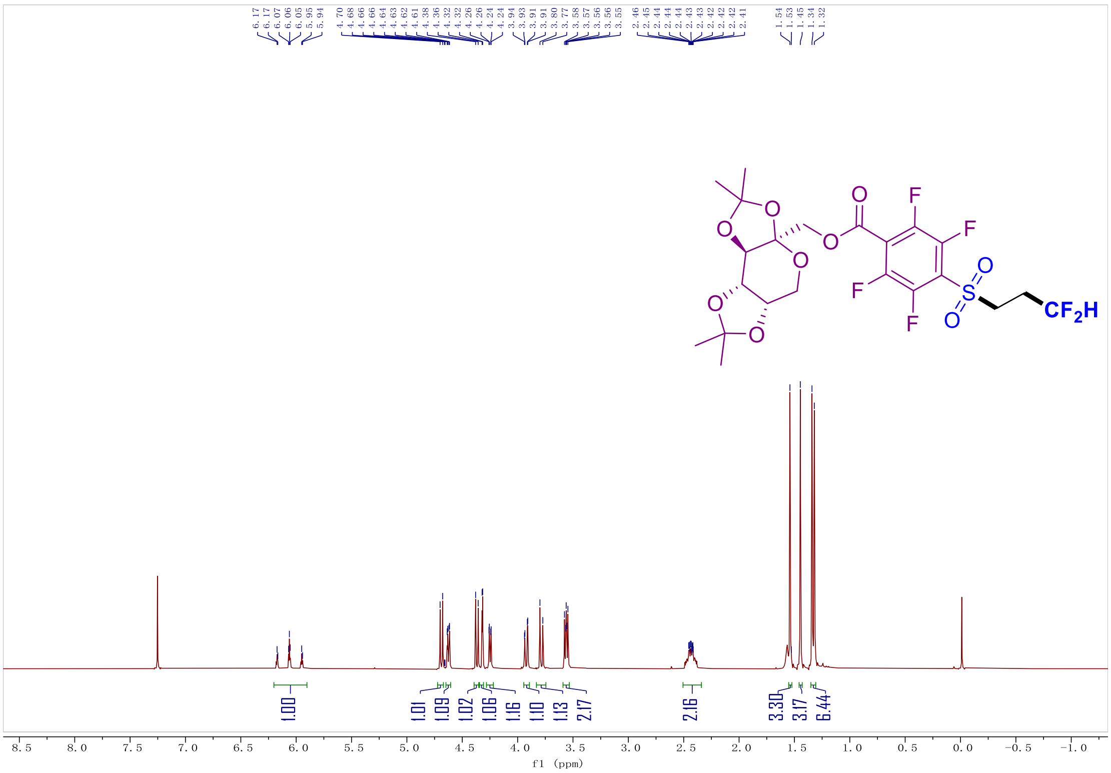

^{13}C NMR (151 MHz, $CDCl_3$) spectrum of 65

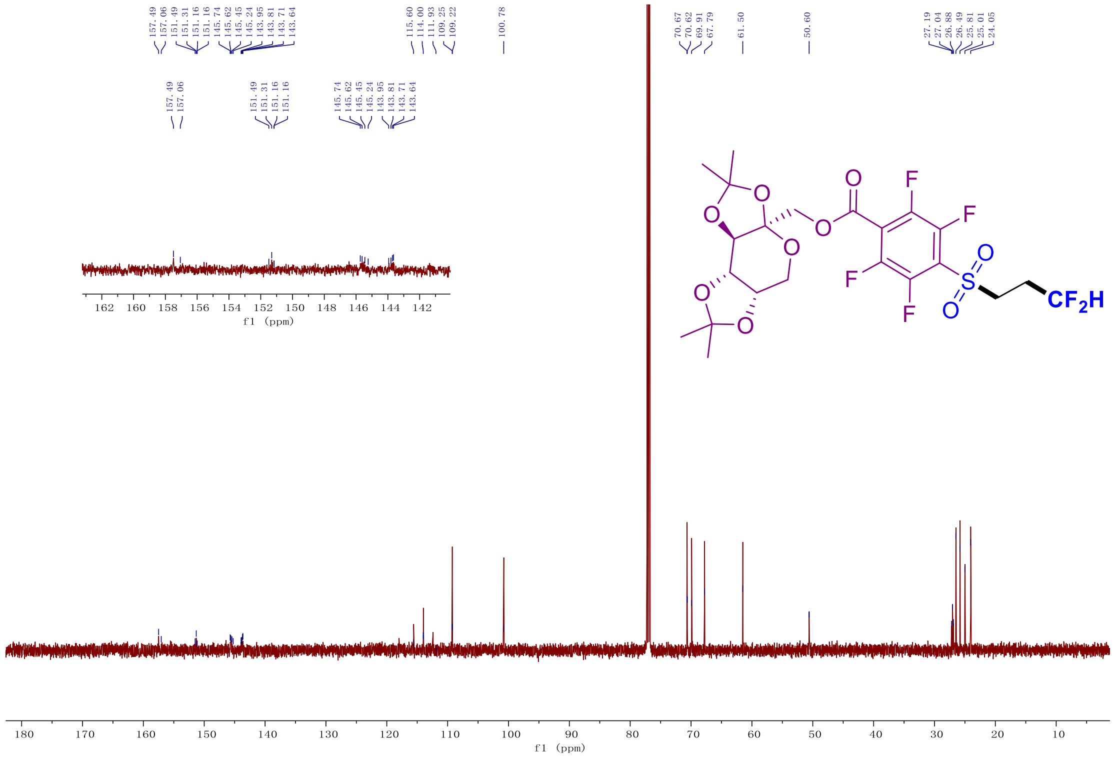

^{19}F NMR (565 MHz, $CDCl_3$) spectrum of 65

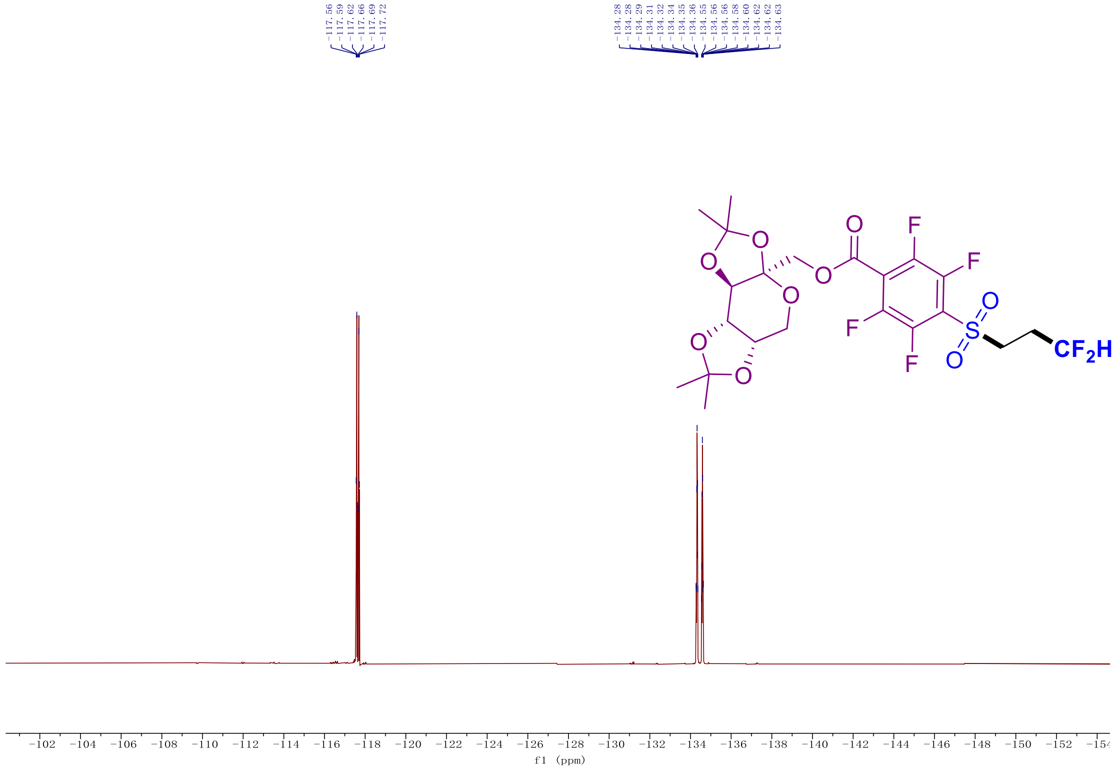

^{1}H NMR (500 MHz, CDCl3) spectrum of 66

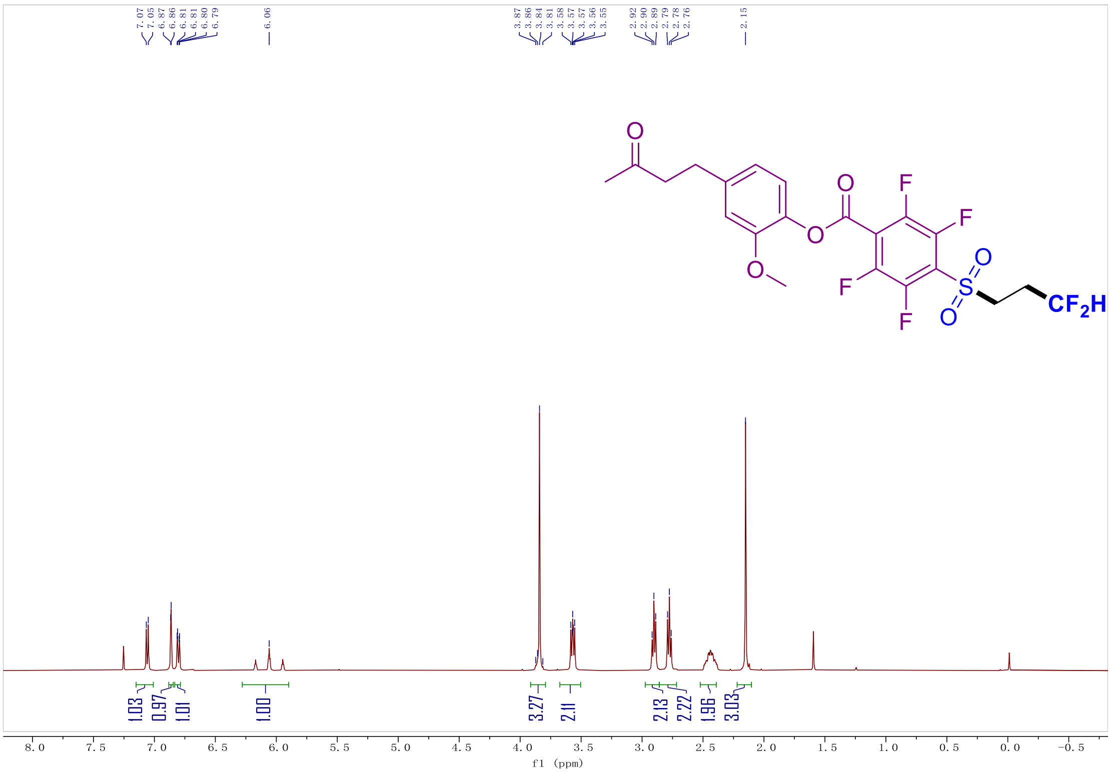

^{13}C NMR (151 MHz, CDCl3) spectrum of 66

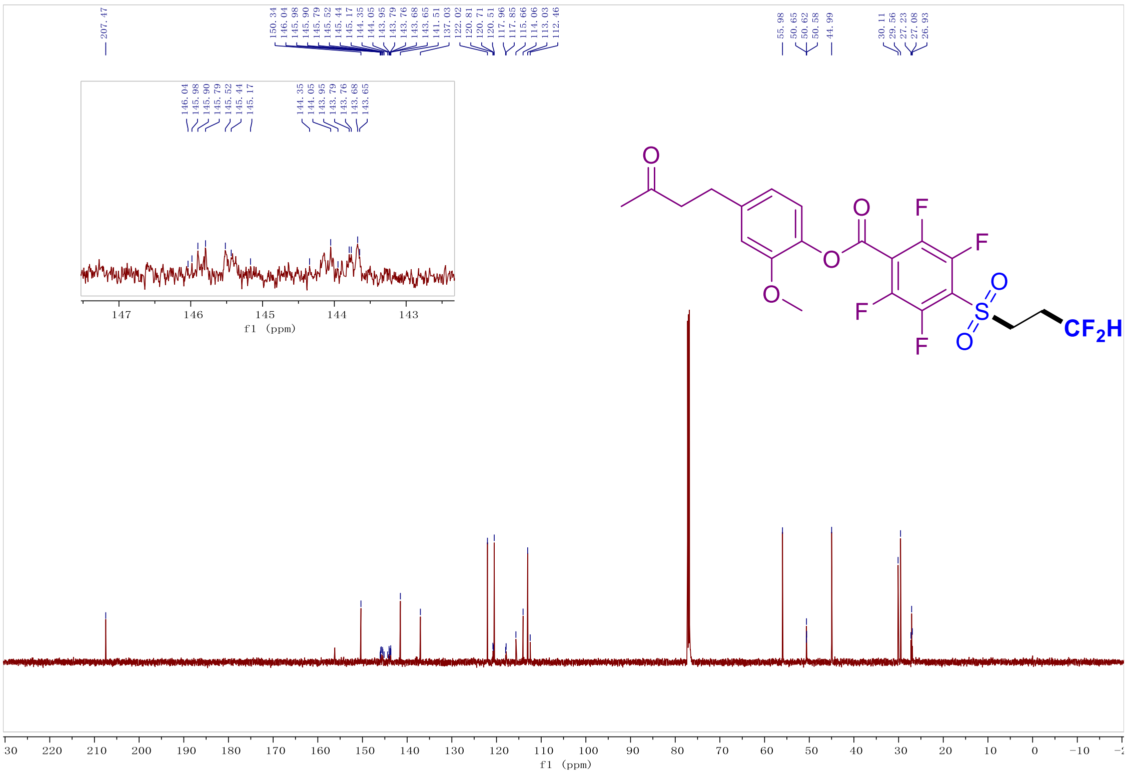

^{19}F NMR (565 MHz, $CDCl_3$) spectrum of 66

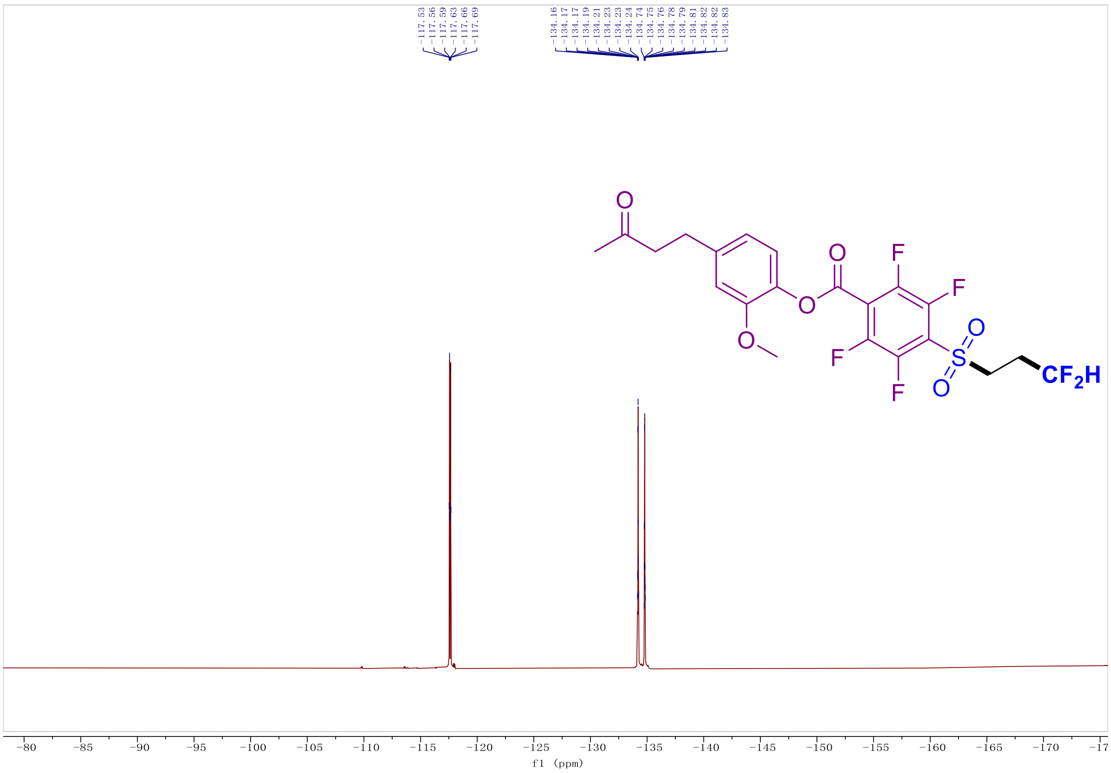

^{1}H NMR (500 MHz, $CDCl_3$) spectrum of 67

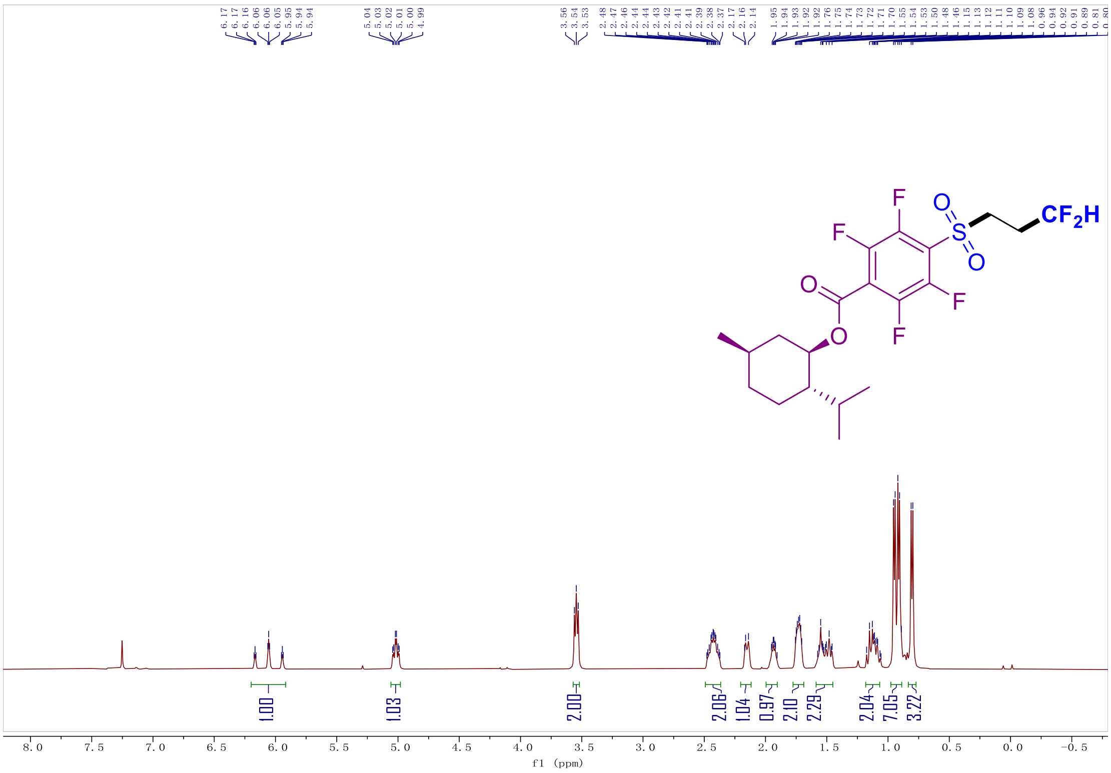

^{13}C NMR (151 MHz, $CDCl_3$) spectrum of 67

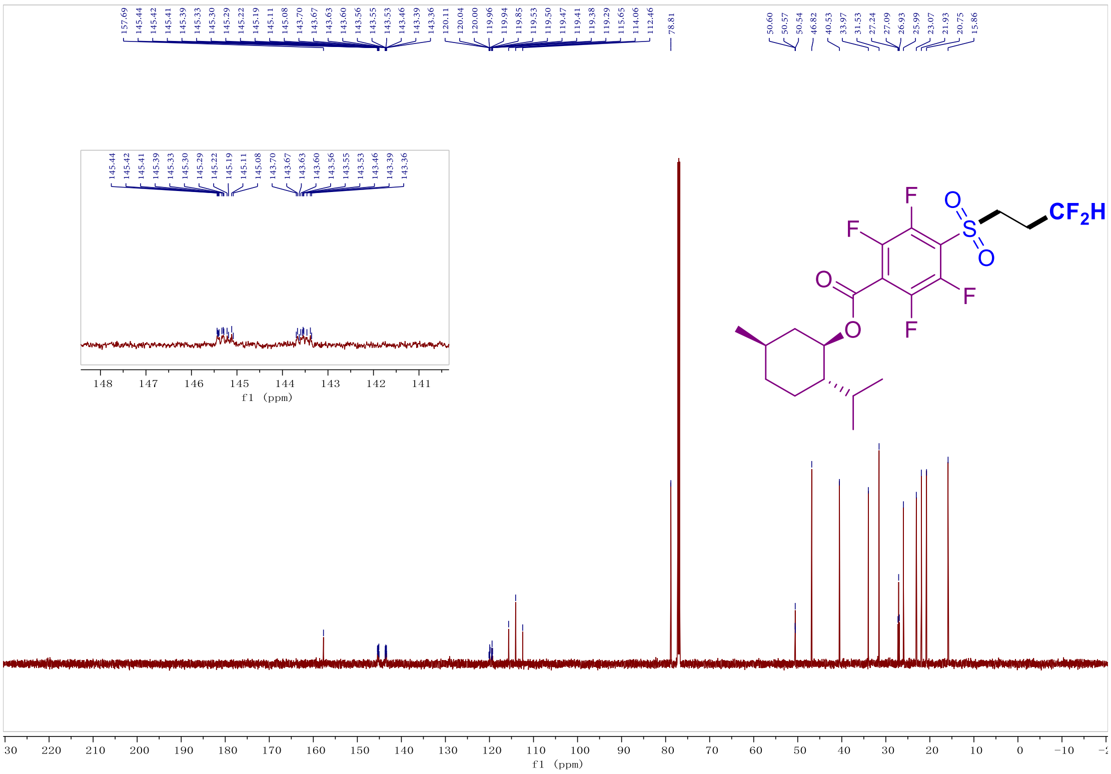

^{19}F NMR (565 MHz, $CDCl_3$) spectrum of 67

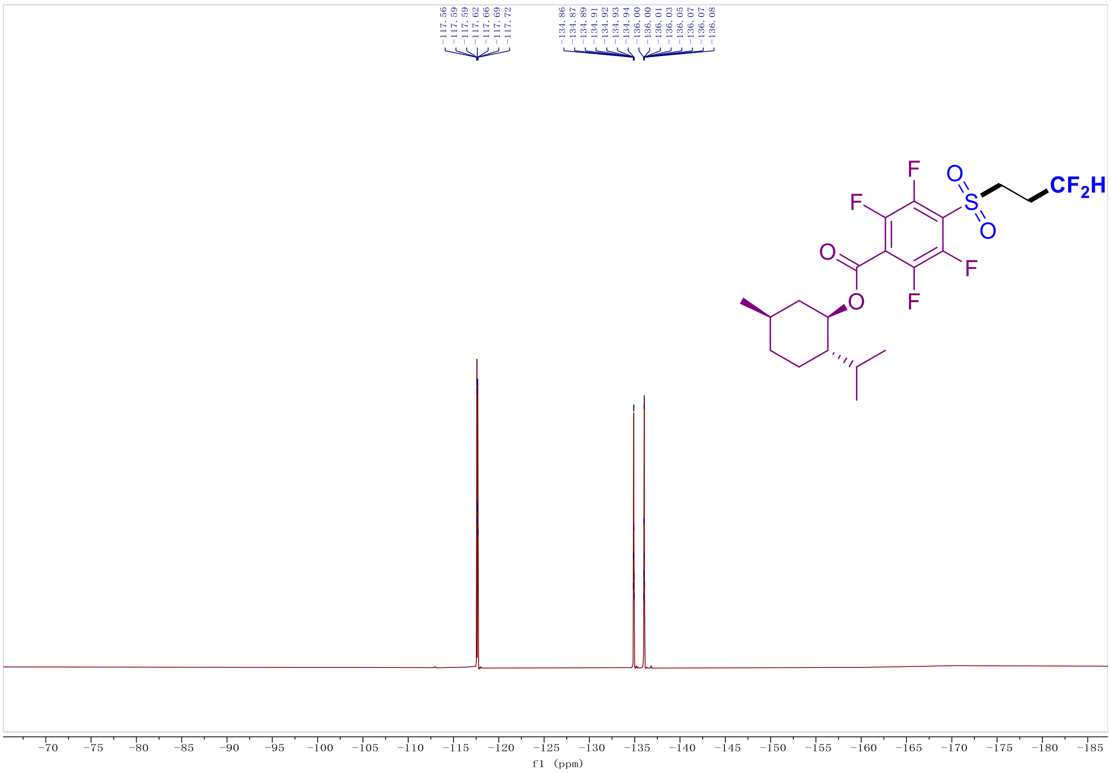

^{1}HNMR (500 MHz, $CDCl_3$) spectrum of 68

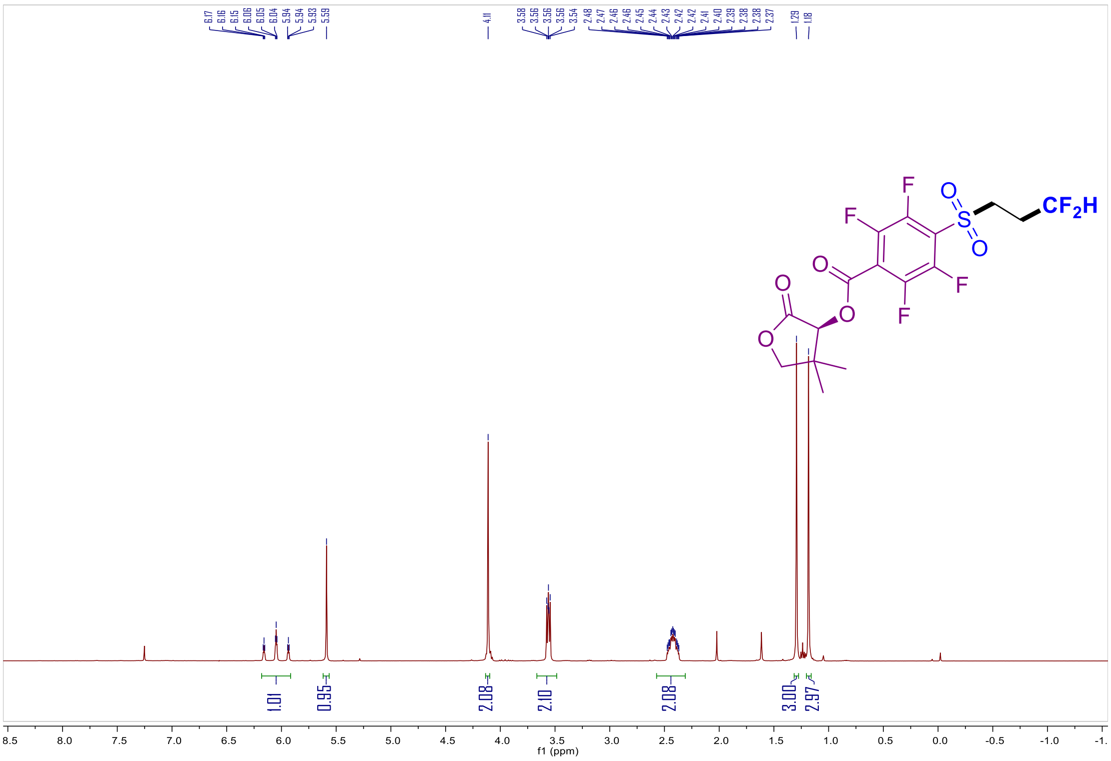

^{13}C NMR (151 MHz, $CDCl_3$) spectrum of 68

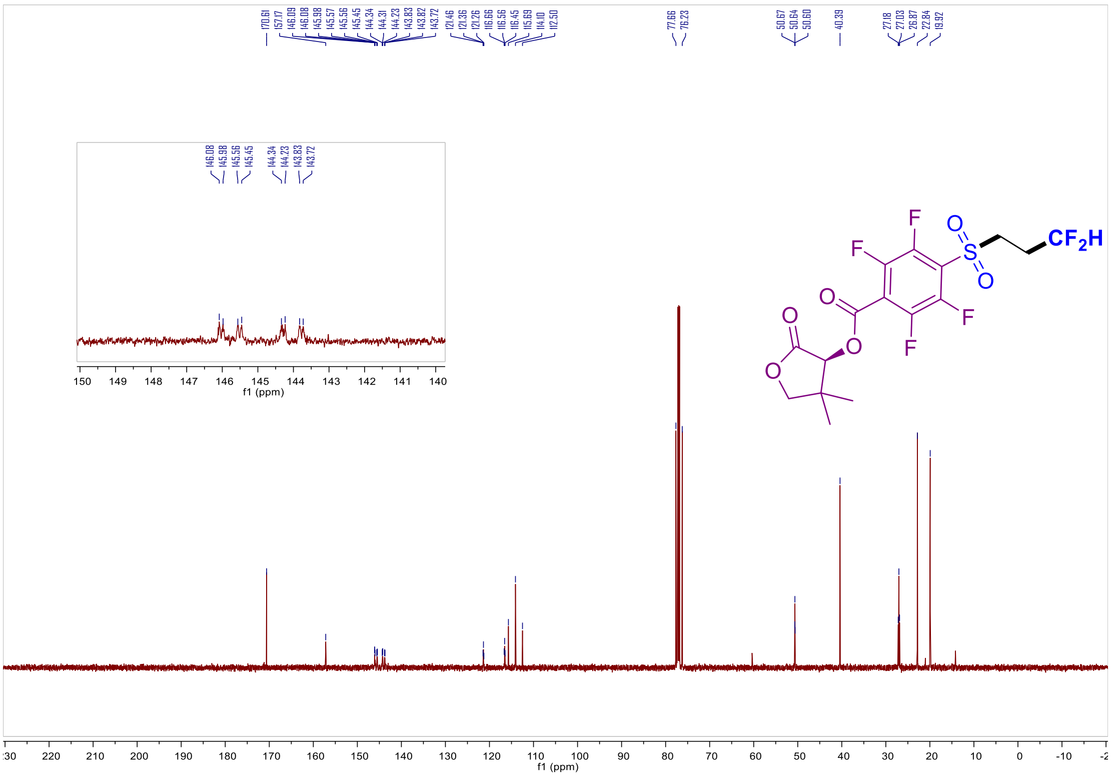

^{19}F NMR (565 MHz, CDCl₃) spectrum of 68

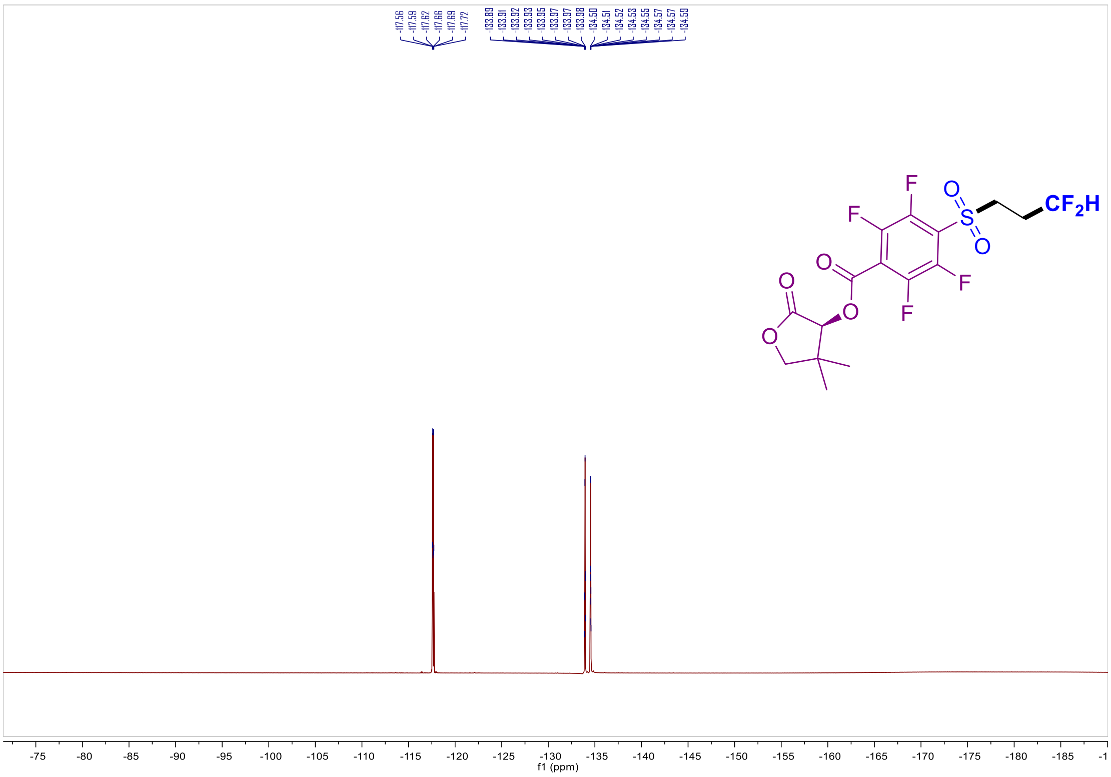

^{1}H NMR (500 MHz, CDCl₃) spectrum of 69

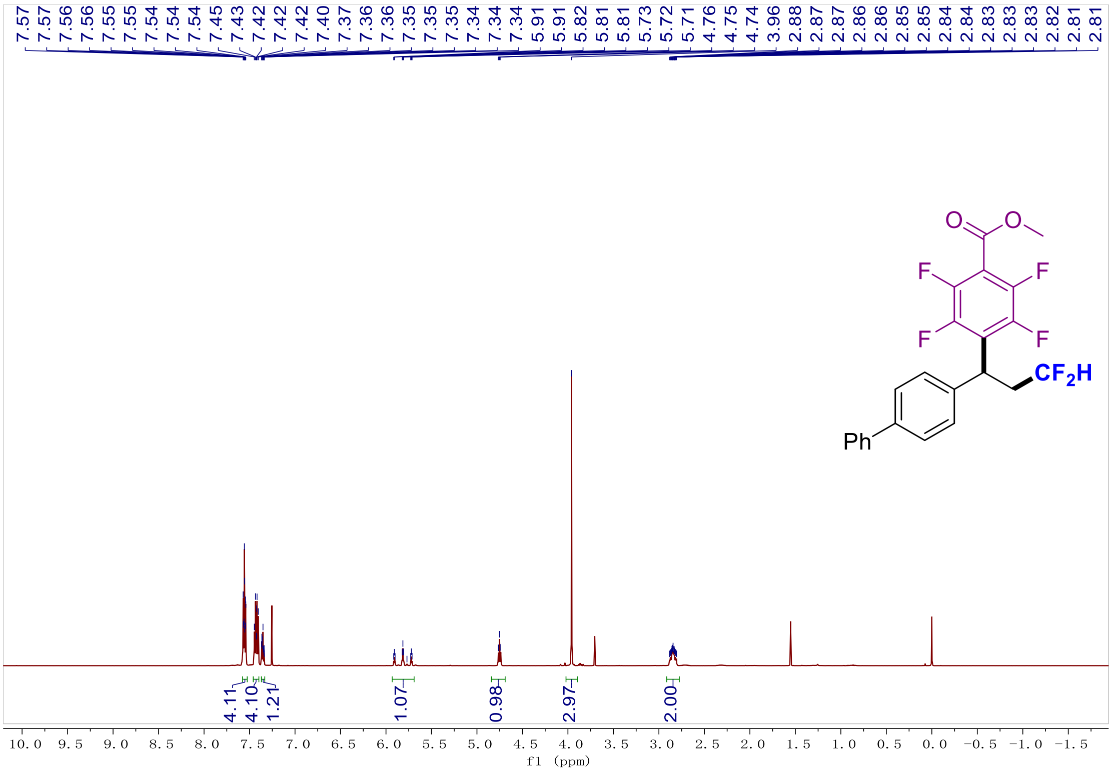

^{13}C NMR (151 MHz, CDCl$_3$) spectrum of 69

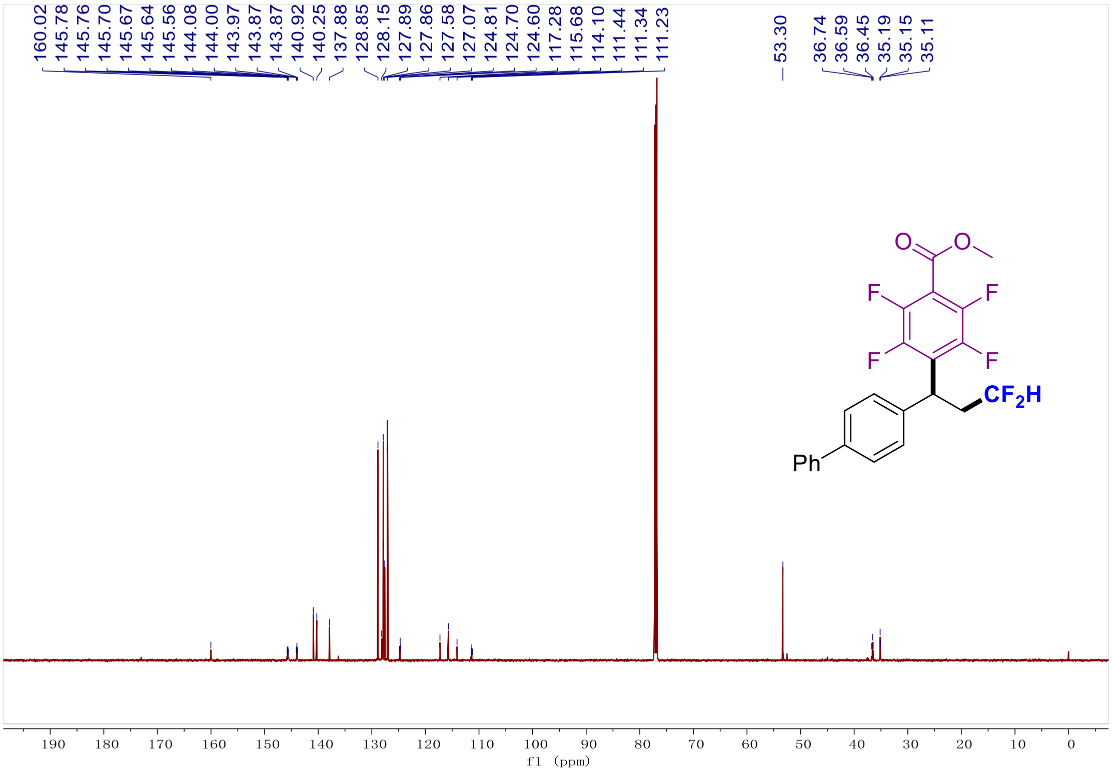

^{19}F NMR (565 MHz, CDCl$_3$) spectrum of 69

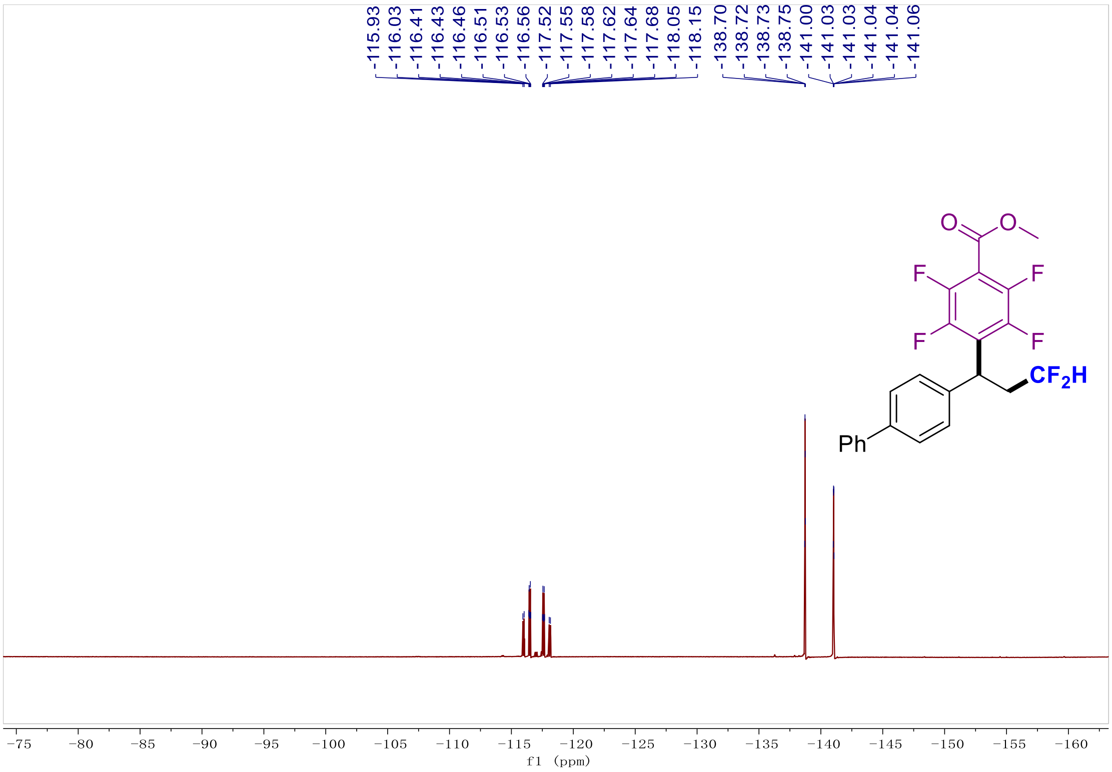

^{1}H NMR (500 MHz, CDCl$_3$) spectrum of 70

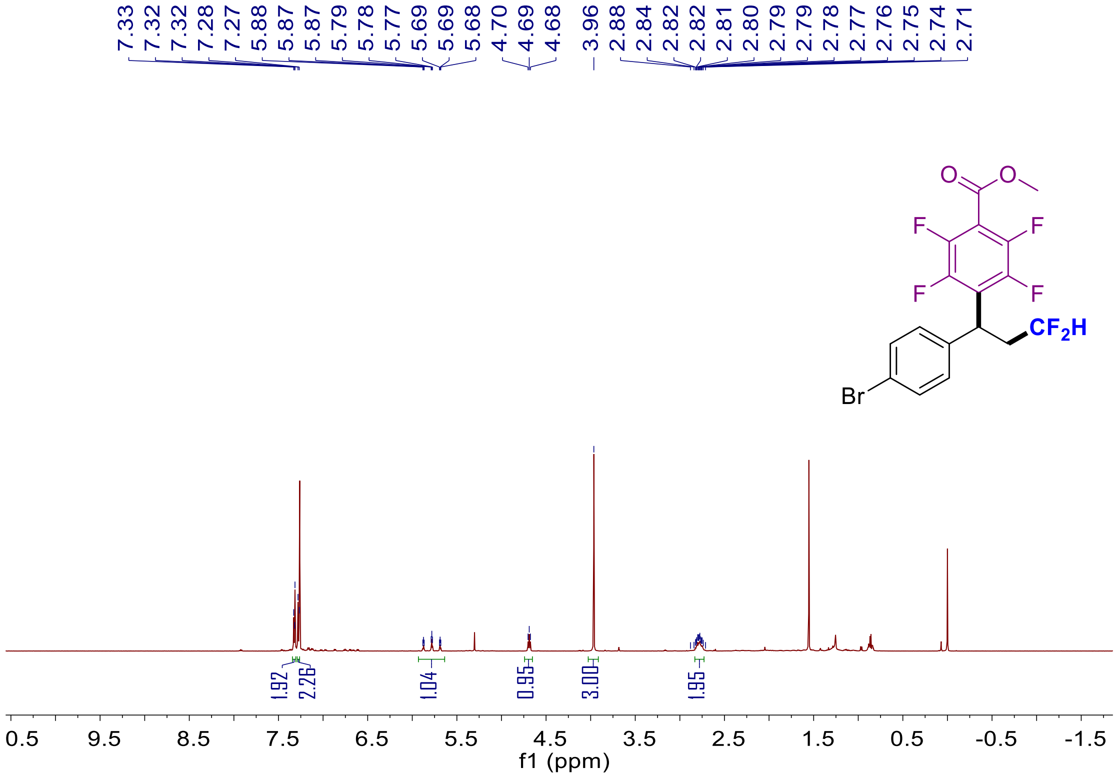

^{13}C NMR (151 MHz, CDCl$_3$) spectrum of 70

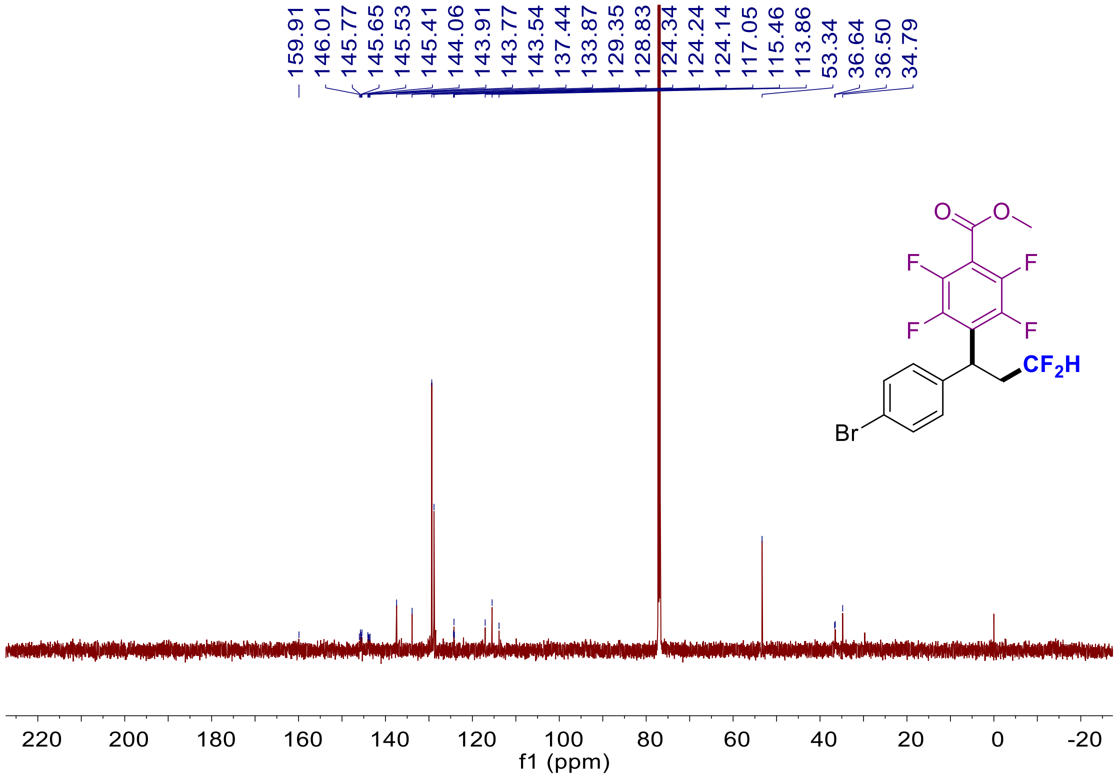

^{19}F NMR (565 MHz, CDCl$_3$) spectrum of 70

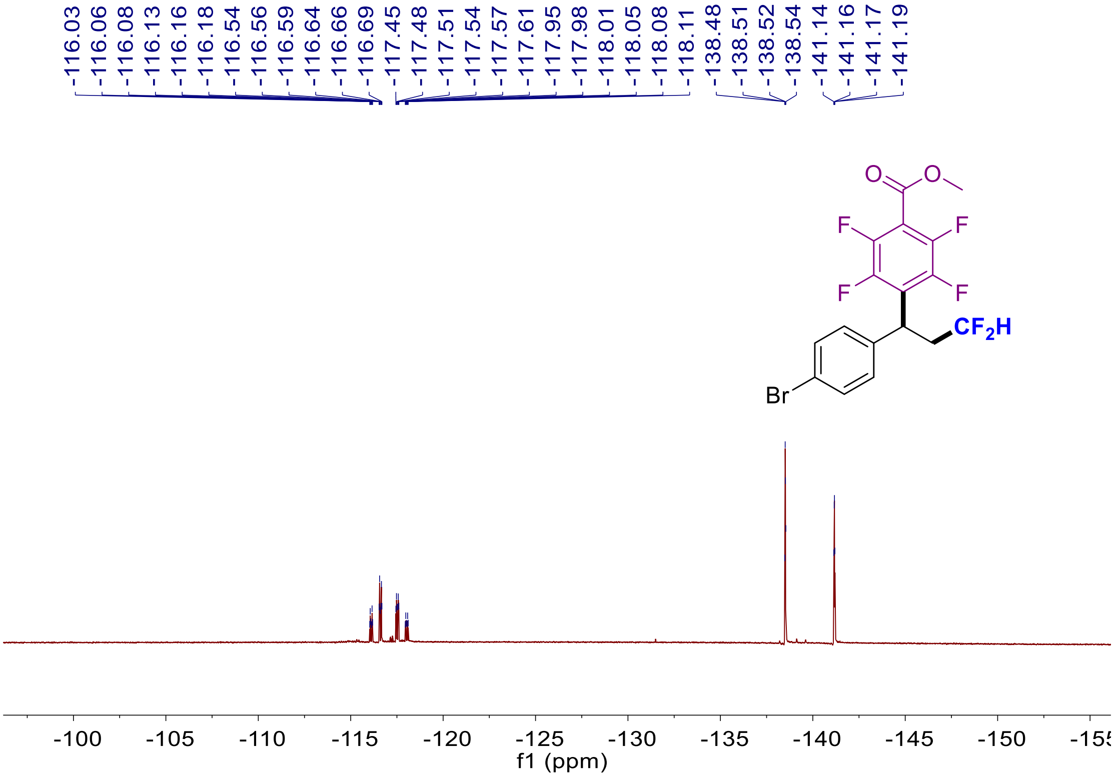

^{1}H NMR (500 MHz, CDCl$_3$) spectrum of 71

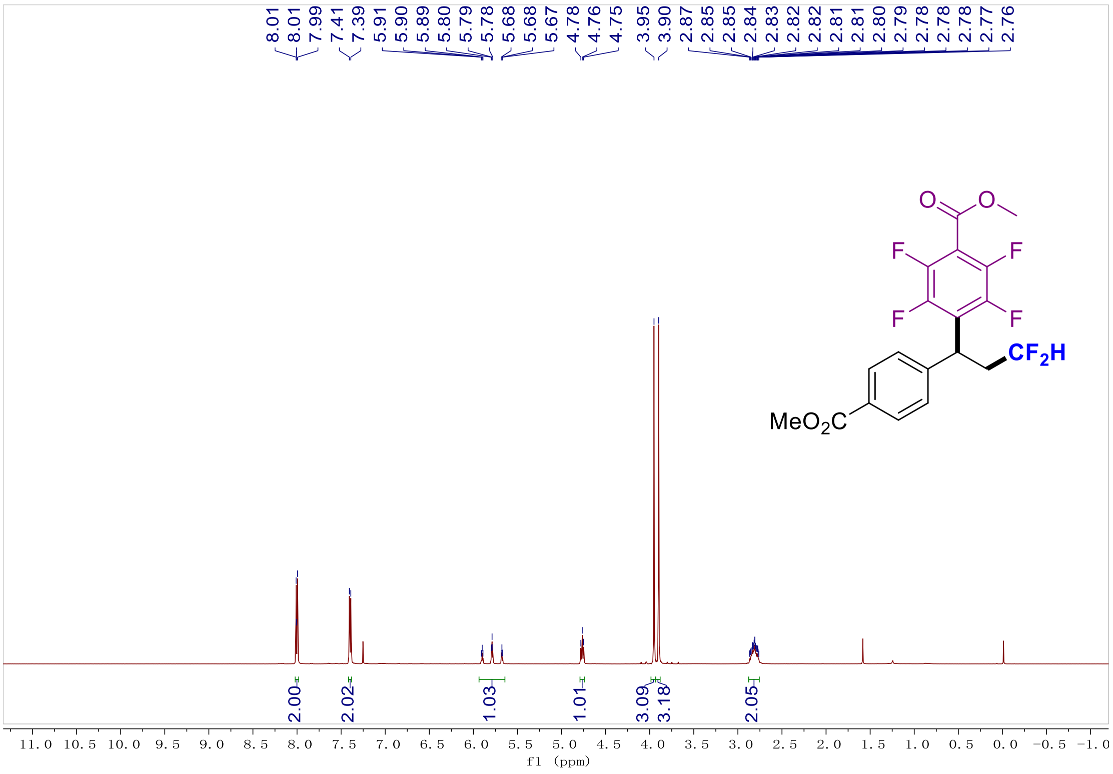

^{13}C NMR (151 MHz, $CDCl_3$) spectrum of 71

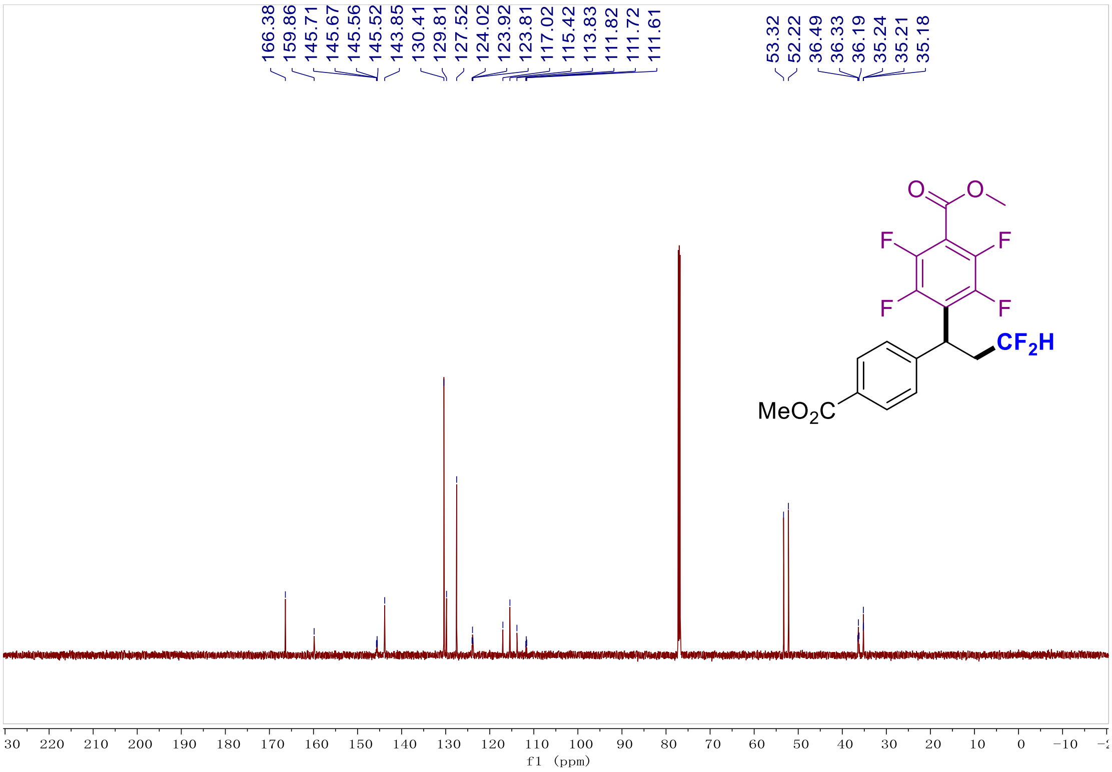

^{19}F NMR (565 MHz, CDCl3) spectrum of 71

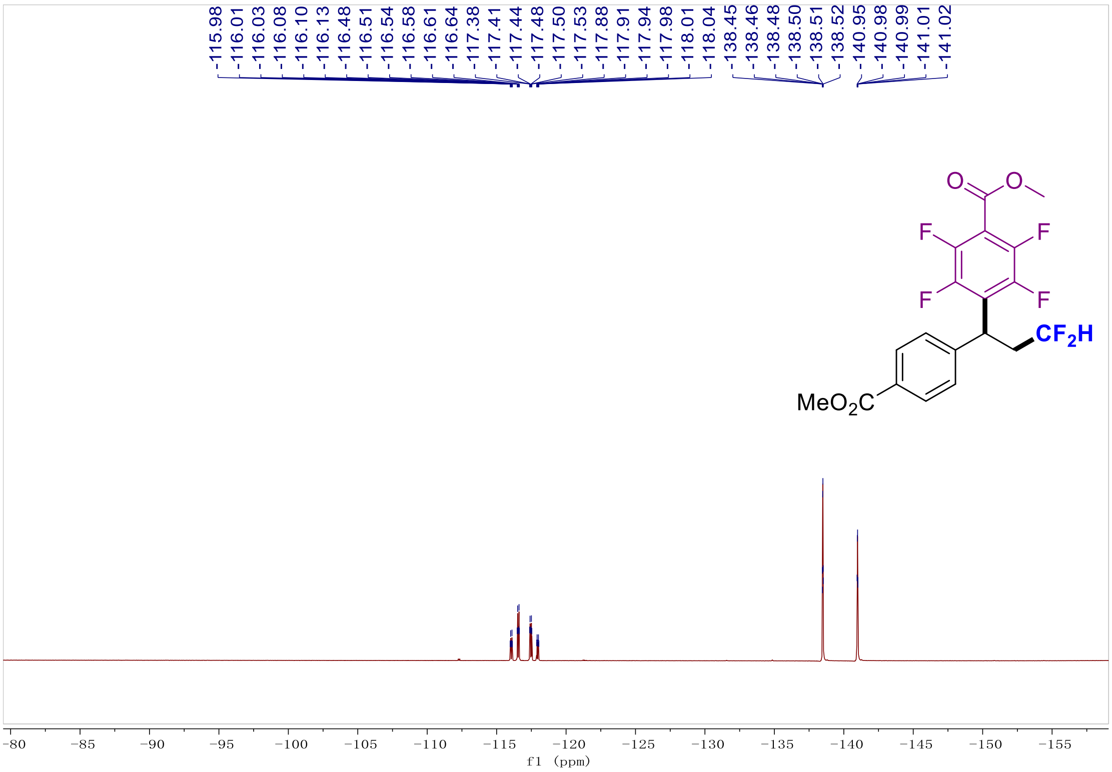

^{1}H NMR (500 MHz, CDCl3) spectrum of 72

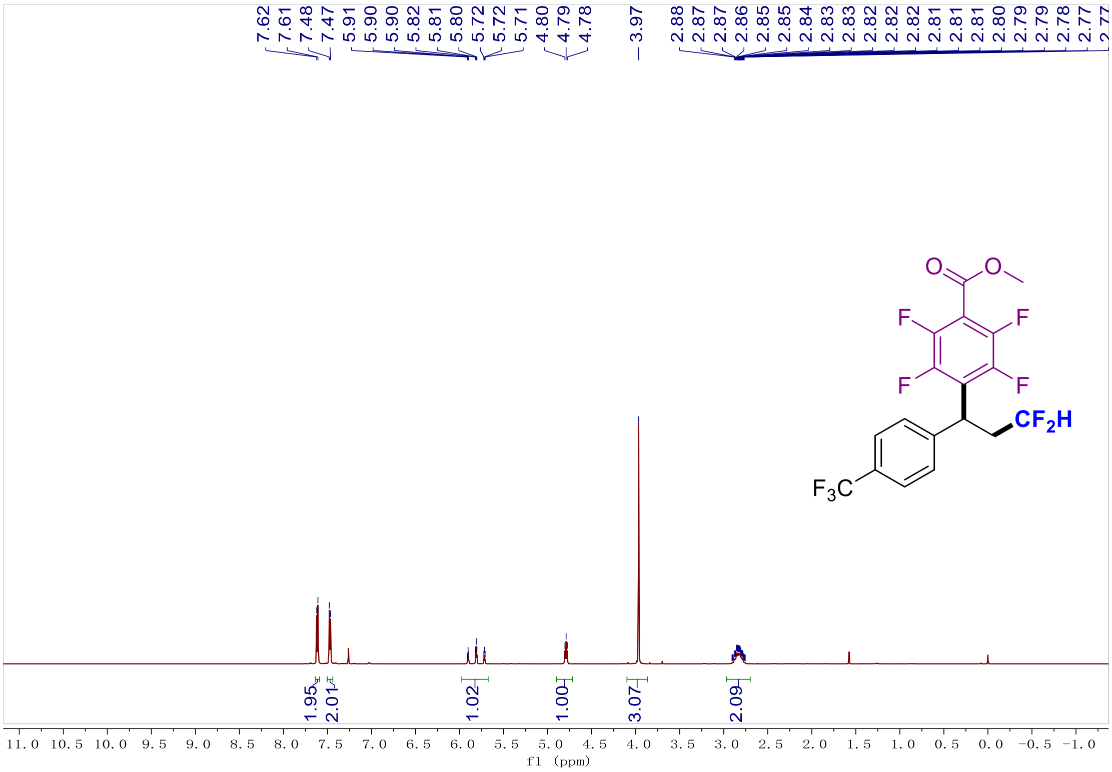

^{13}C NMR (151 MHz, CDCl3) spectrum of 72

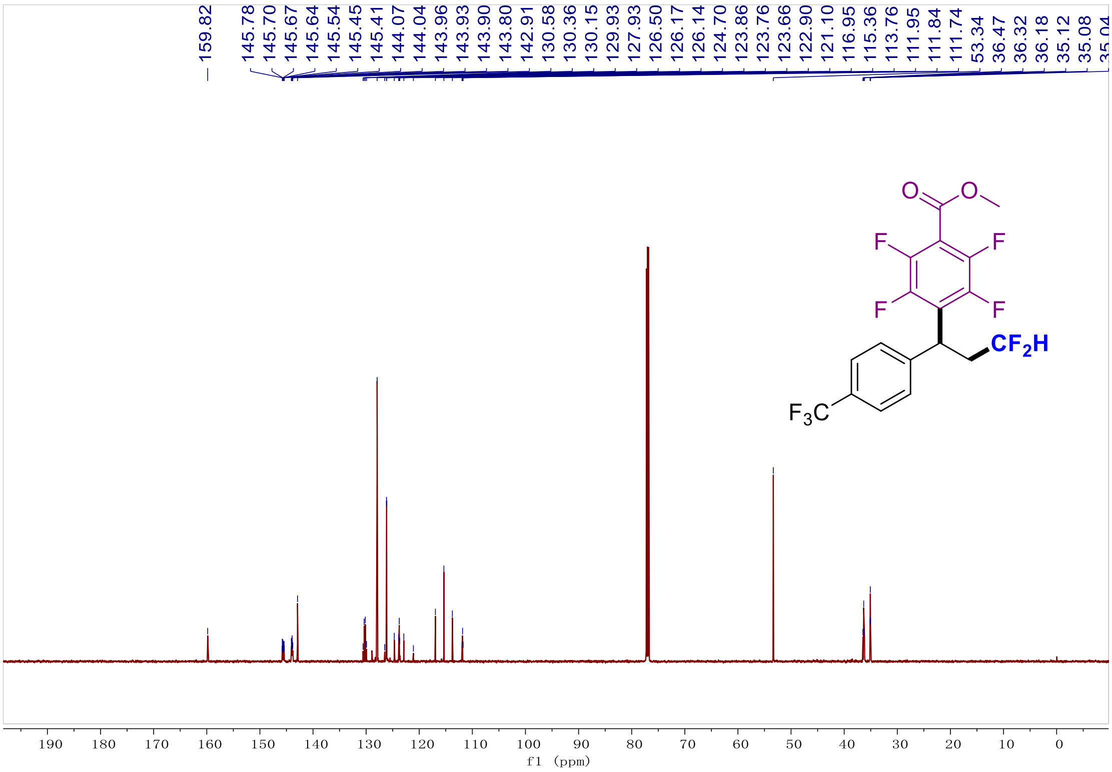

^{19}F NMR (565 MHz, CDCl3) spectrum of 72

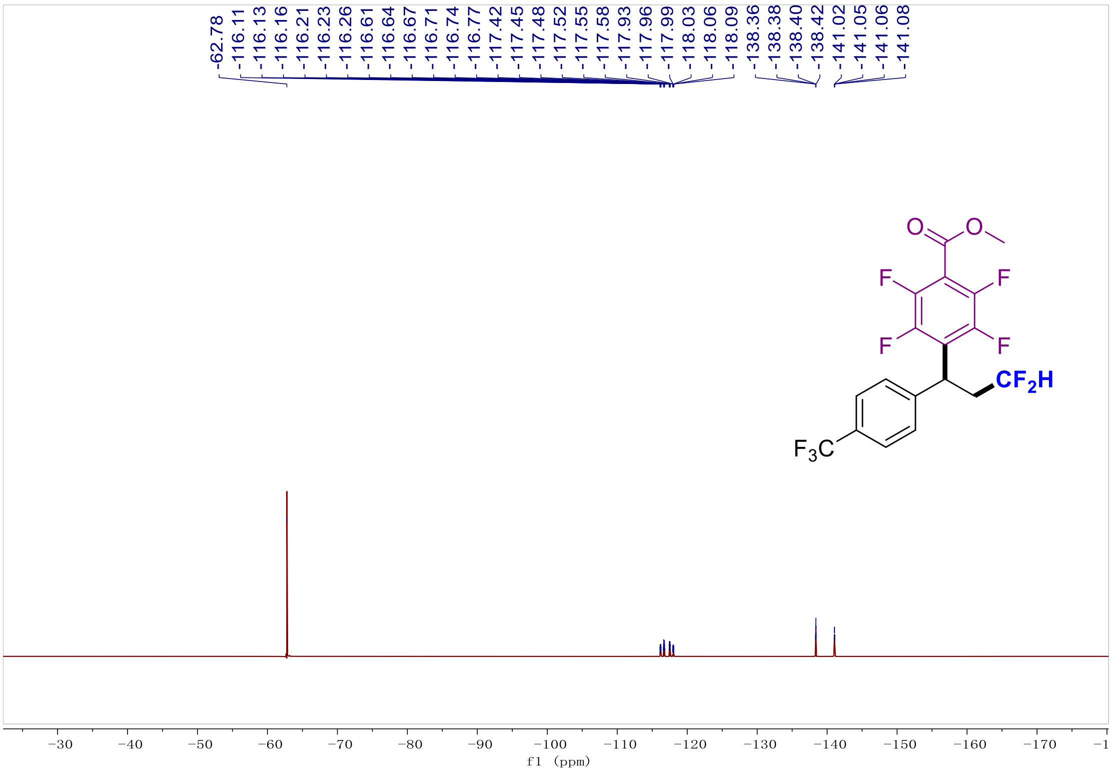

^{1}H NMR (600 MHz, CDCl3) spectrum of 73

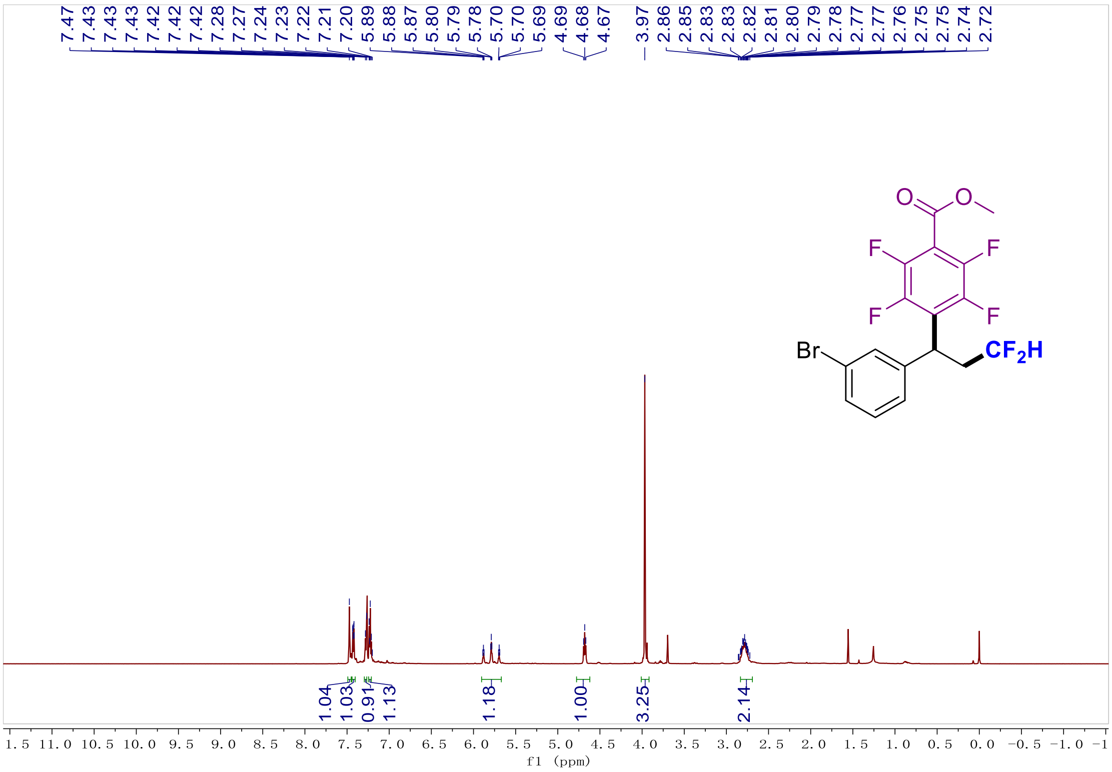

^{13}C NMR (151 MHz, CDCl$_3$) spectrum of 73

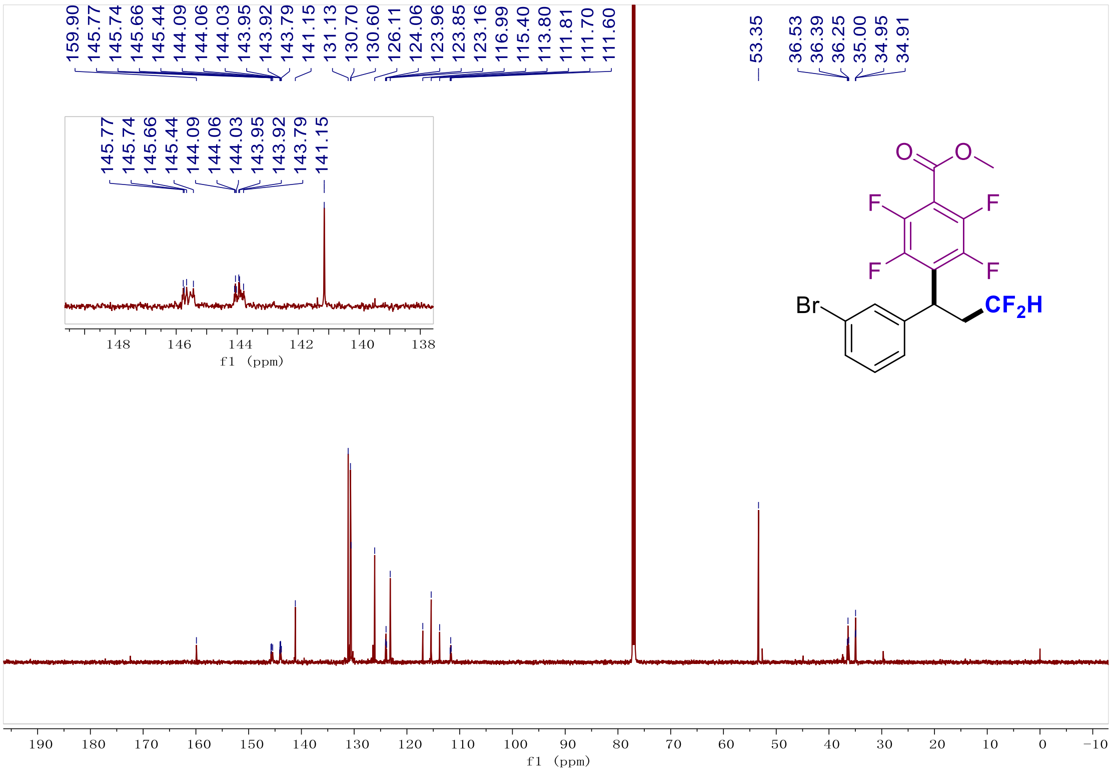

^{19}F NMR (565 MHz, CDCl$_3$) spectrum of 73

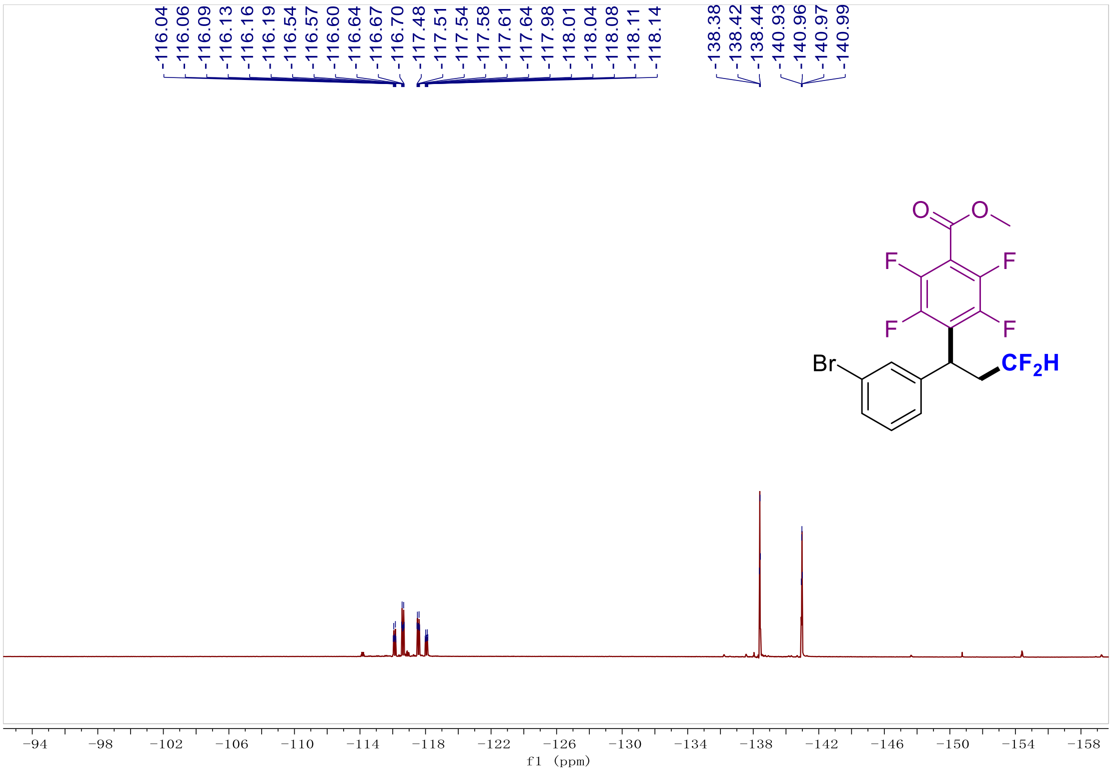

^{1}H NMR (600 MHz, $CDCl_3$) spectrum of 74

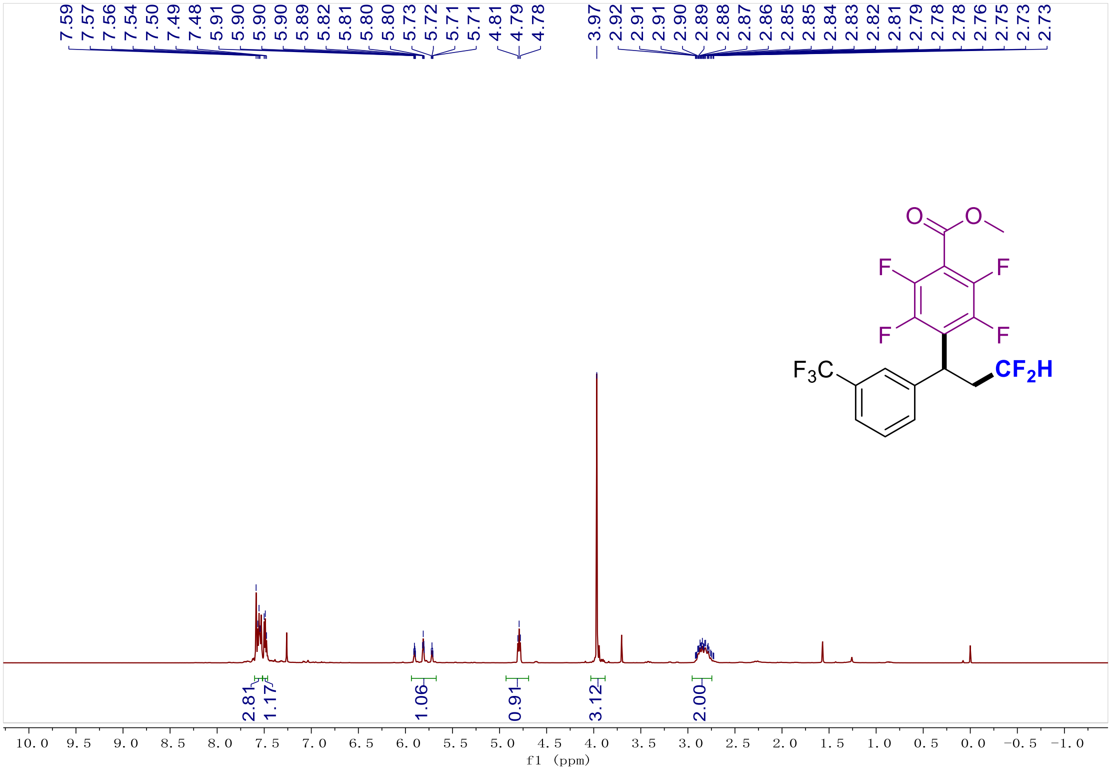

^{13}C NMR (151 MHz, $CDCl_3$) spectrum of 74

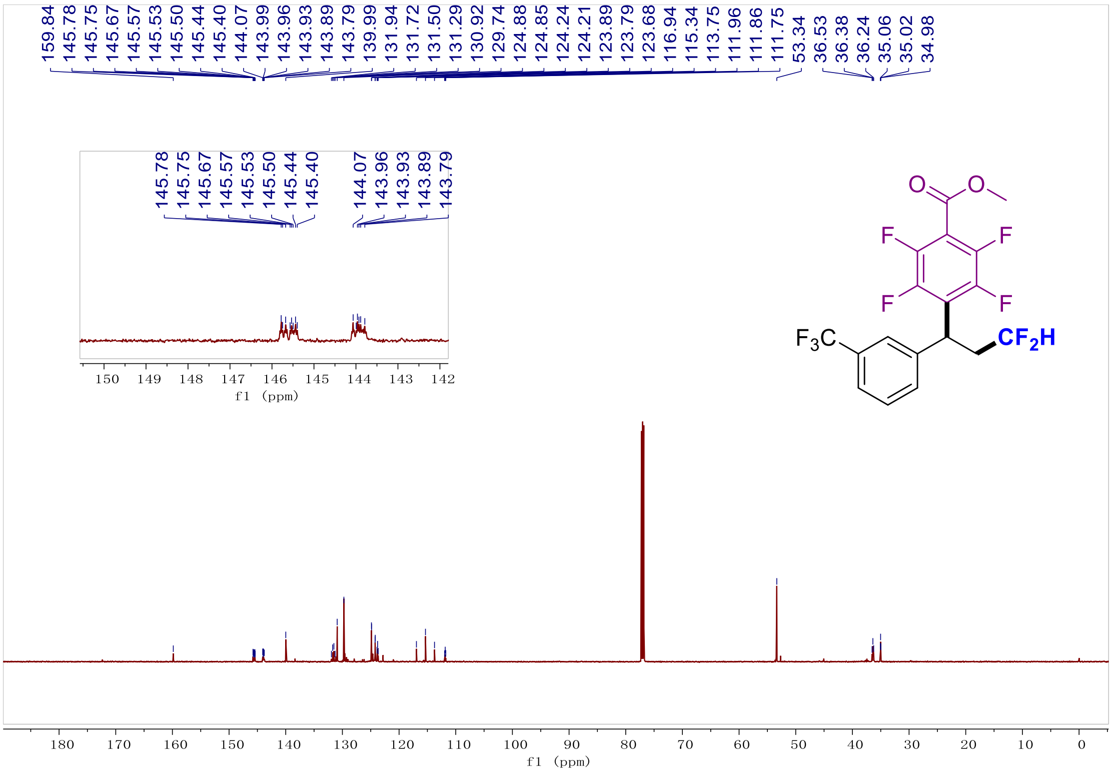

^{19}F NMR (565 MHz, $CDCl_3$) spectrum of 74

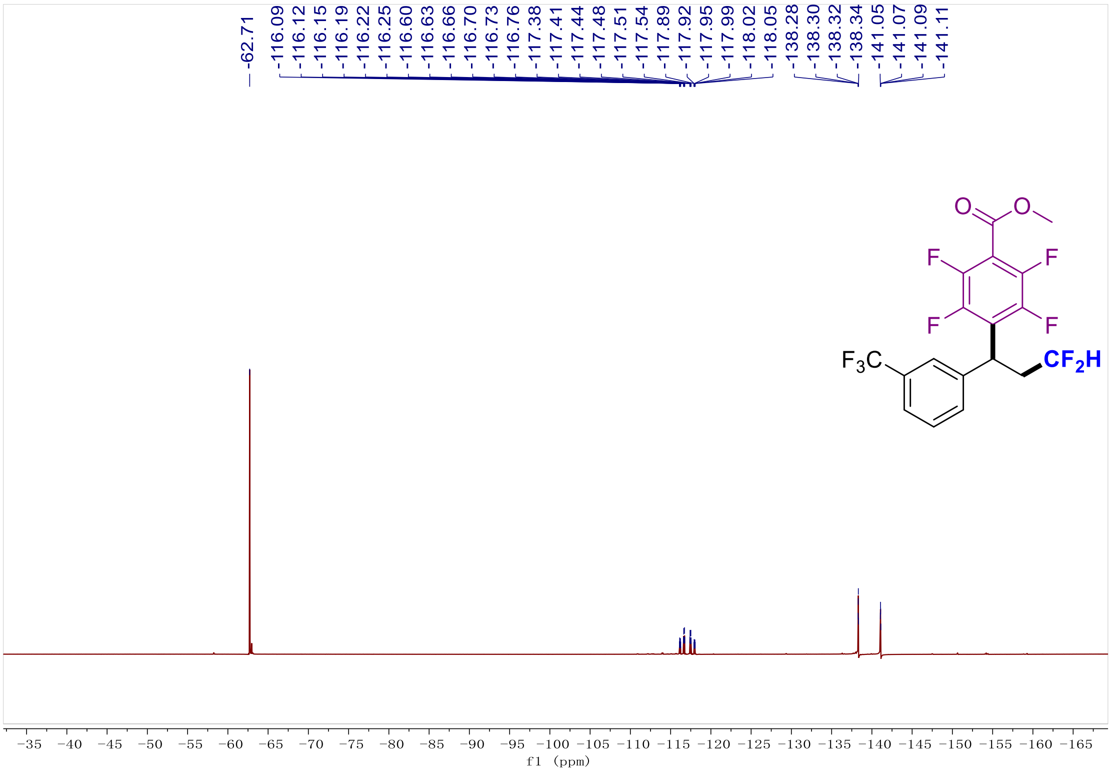

^{1}H NMR (600 MHz, $CDCl_3$) spectrum of 75

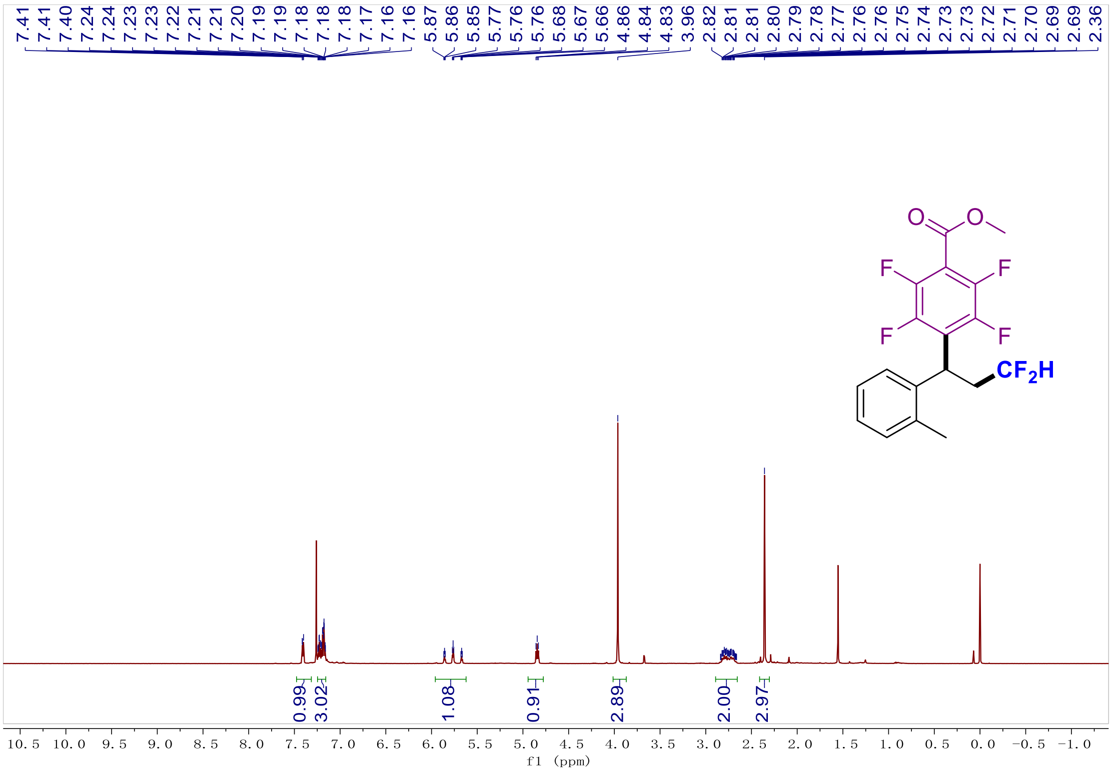

^{13}C NMR (151 MHz, $CDCl_3$) spectrum of 75

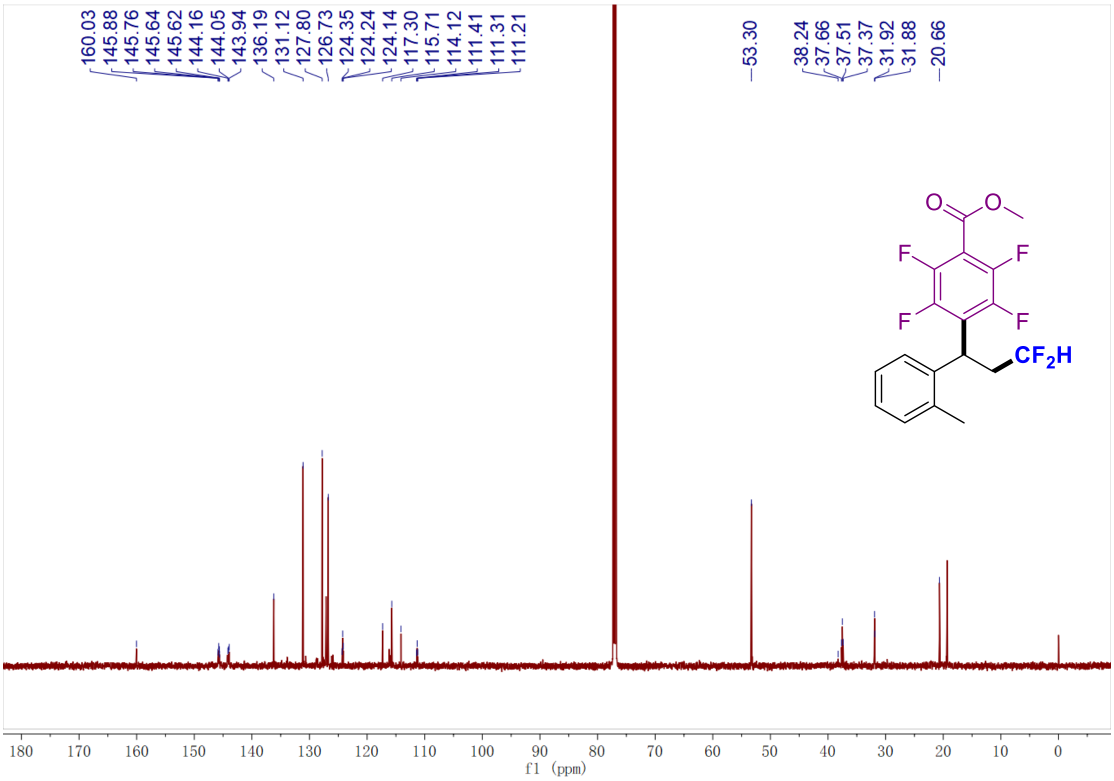

^{19}F NMR (565 MHz, $CDCl_3$) spectrum of 75

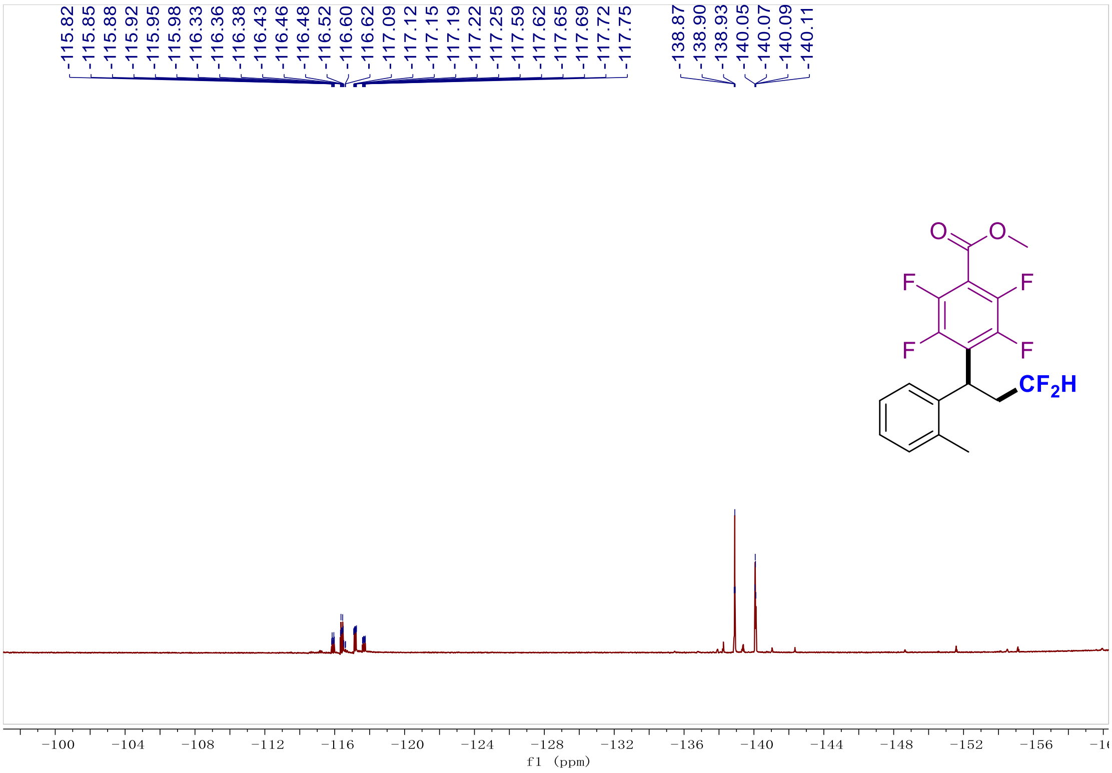

^{1}H NMR (600 MHz, $CDCl_3$) spectrum of 76

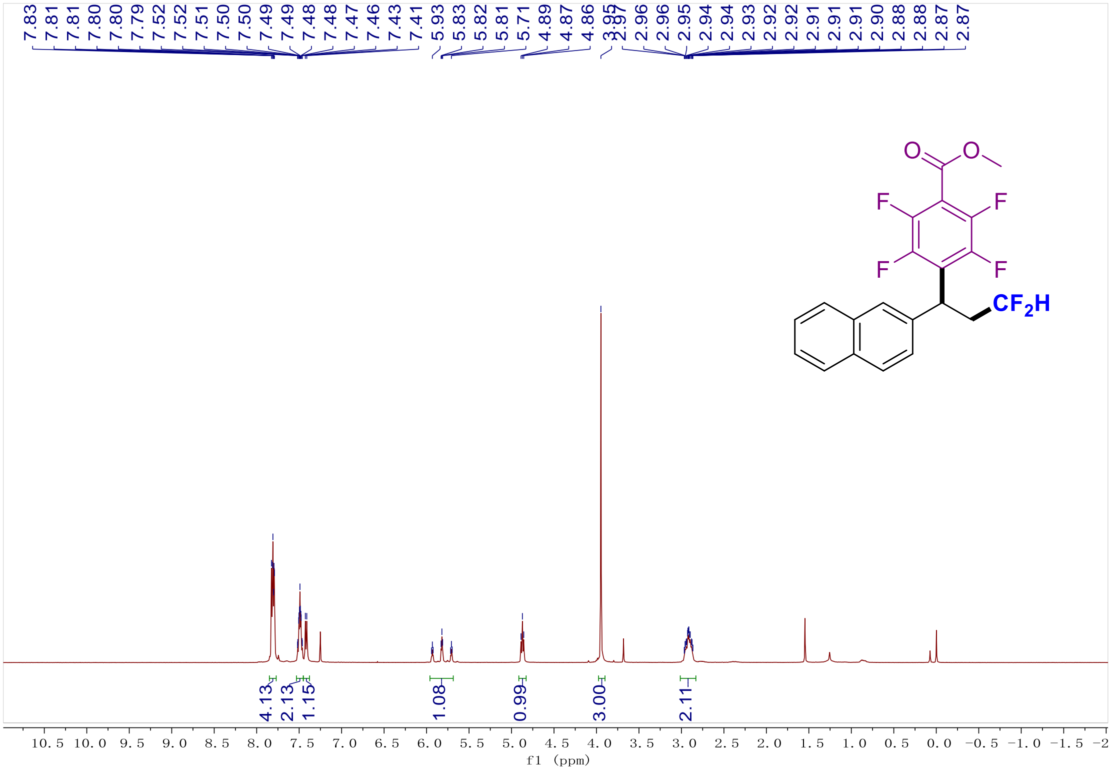

^{13}C NMR (151 MHz, $CDCl_3$) spectrum of 76

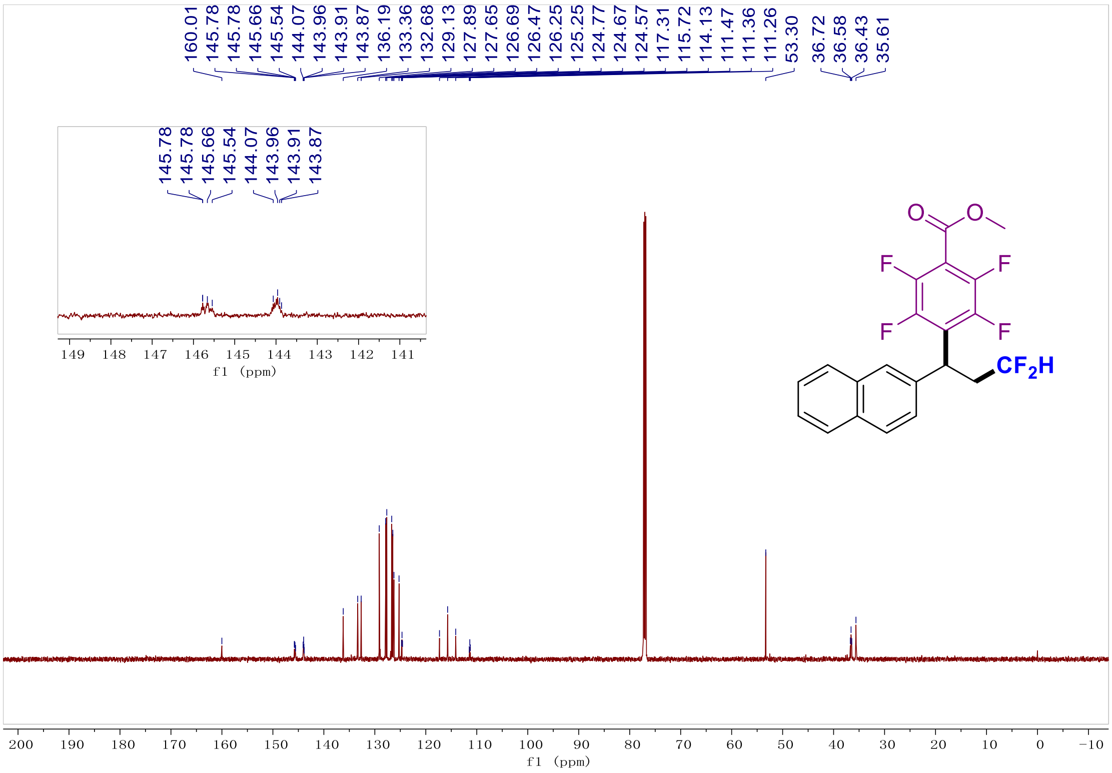

^{19}F NMR (565 MHz, $CDCl_3$) spectrum of 76

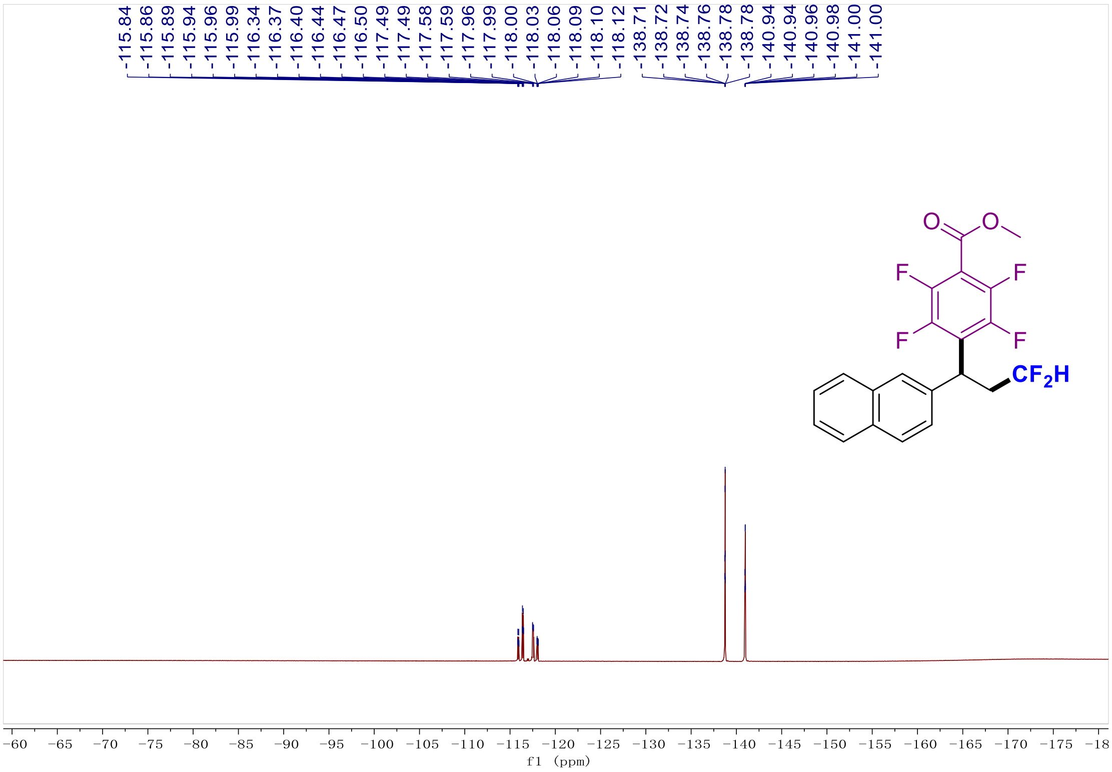

^{1}H NMR (600 MHz, $CDCl_3$) spectrum of 77

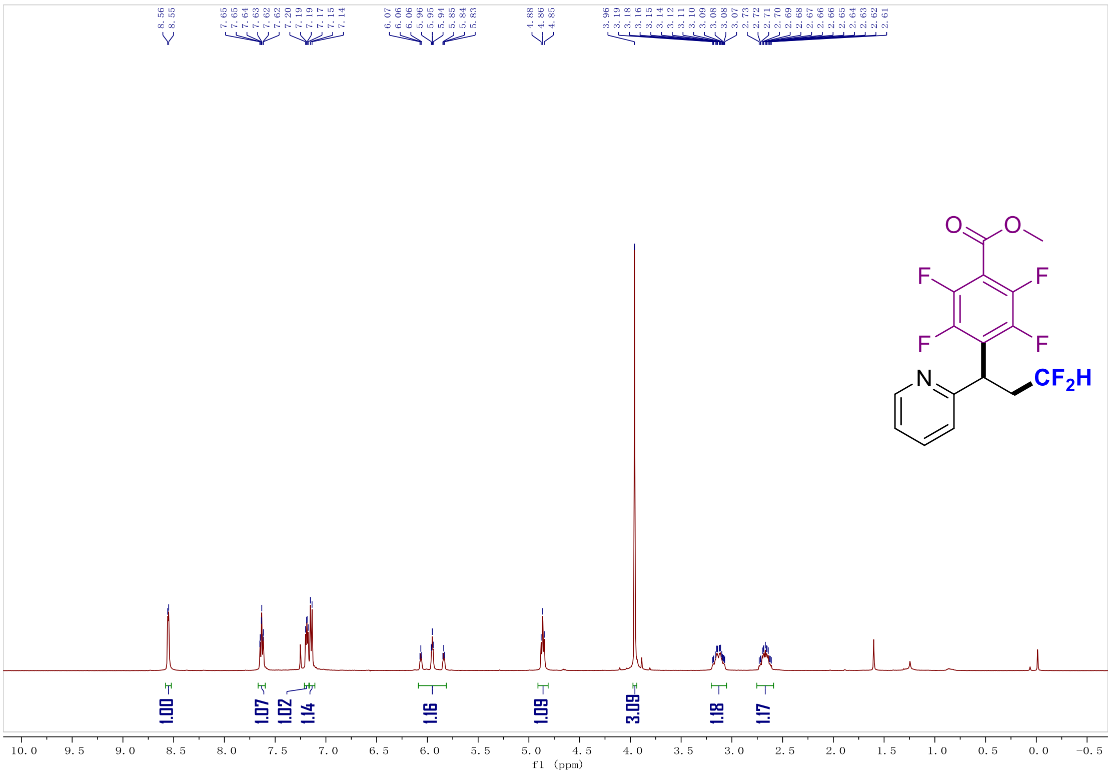

^{13}C NMR (151 MHz, CDCl$_3$) spectrum of 77

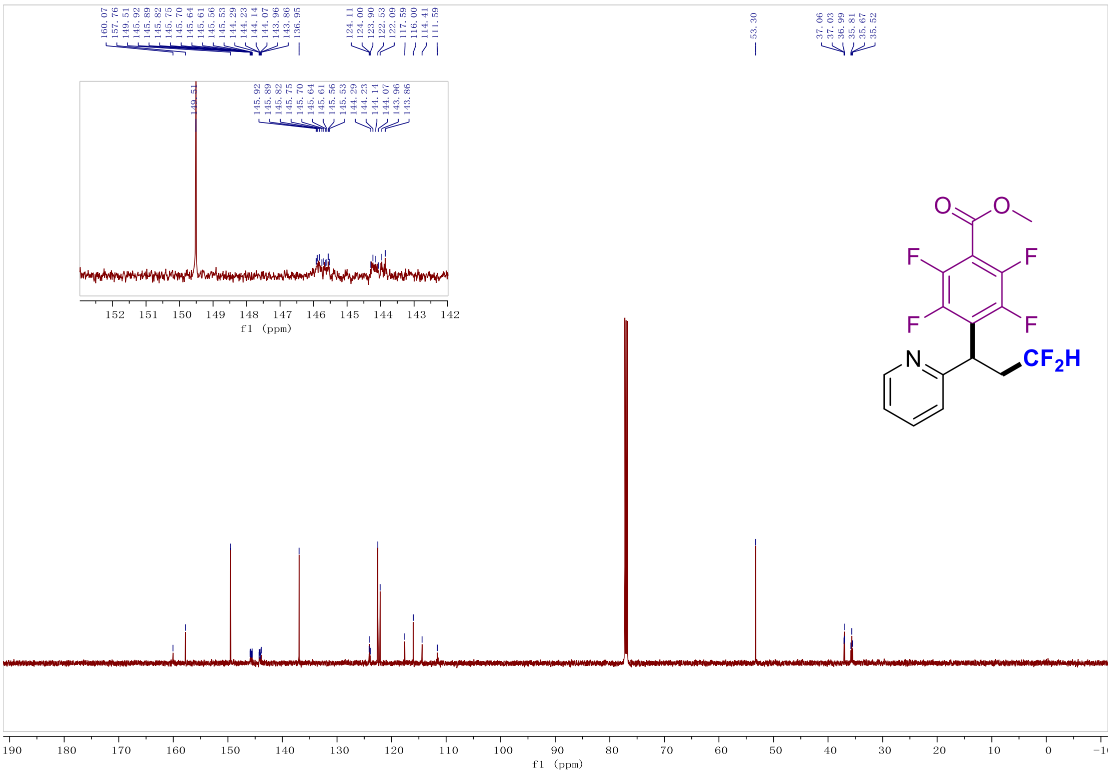

^{19}F NMR (565 MHz, CDCl$_3$) spectrum of 77

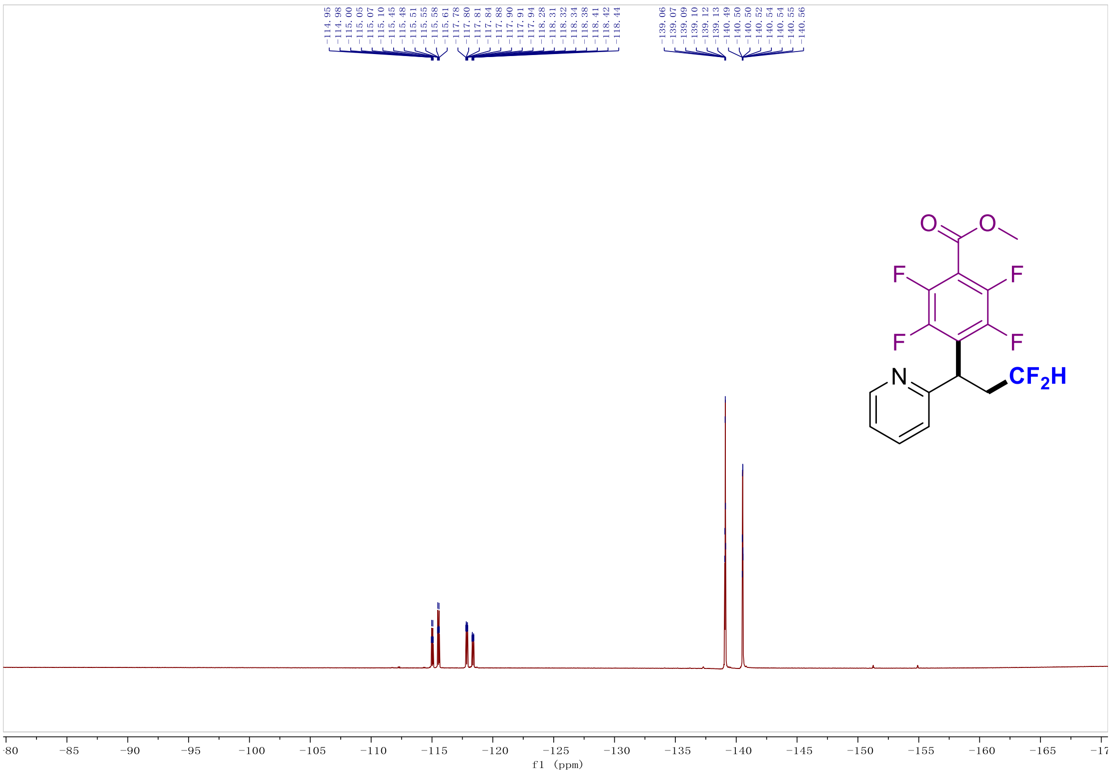

^{1}H NMR (600 MHz, CDCl3) spectrum of 78

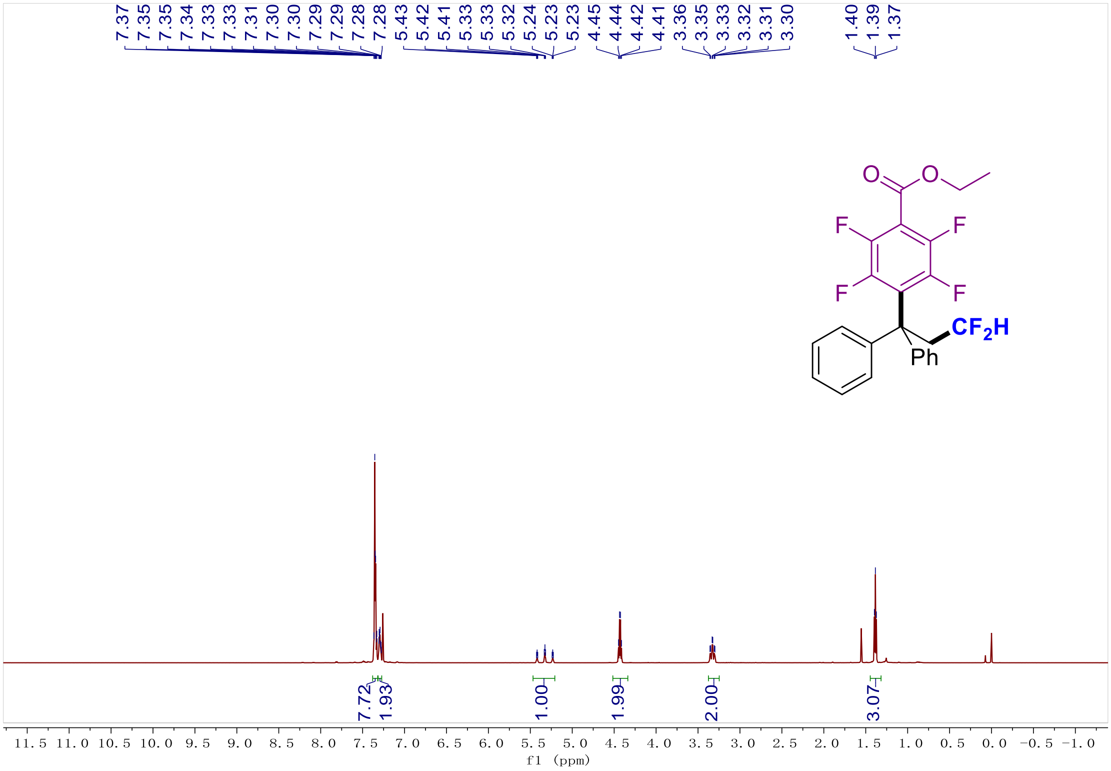

^{13}C NMR (151 MHz, CDCl3) spectrum of 78

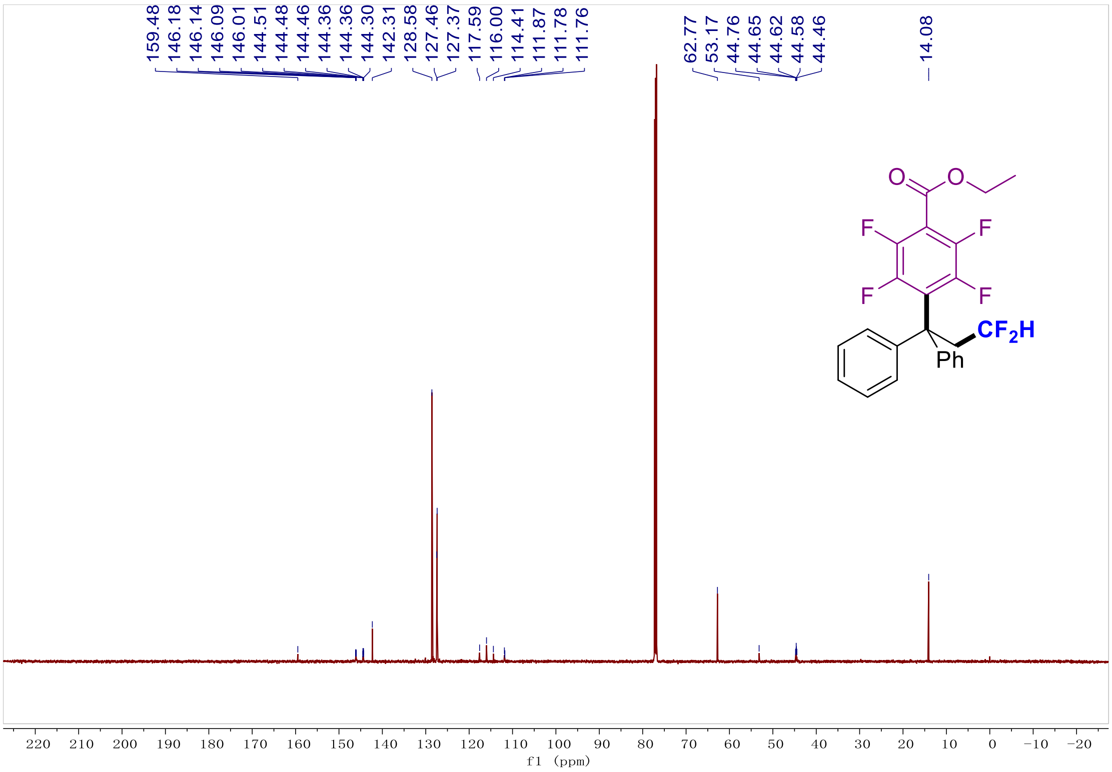

^{19}F NMR (565 MHz, $CDCl_3$) spectrum of 78

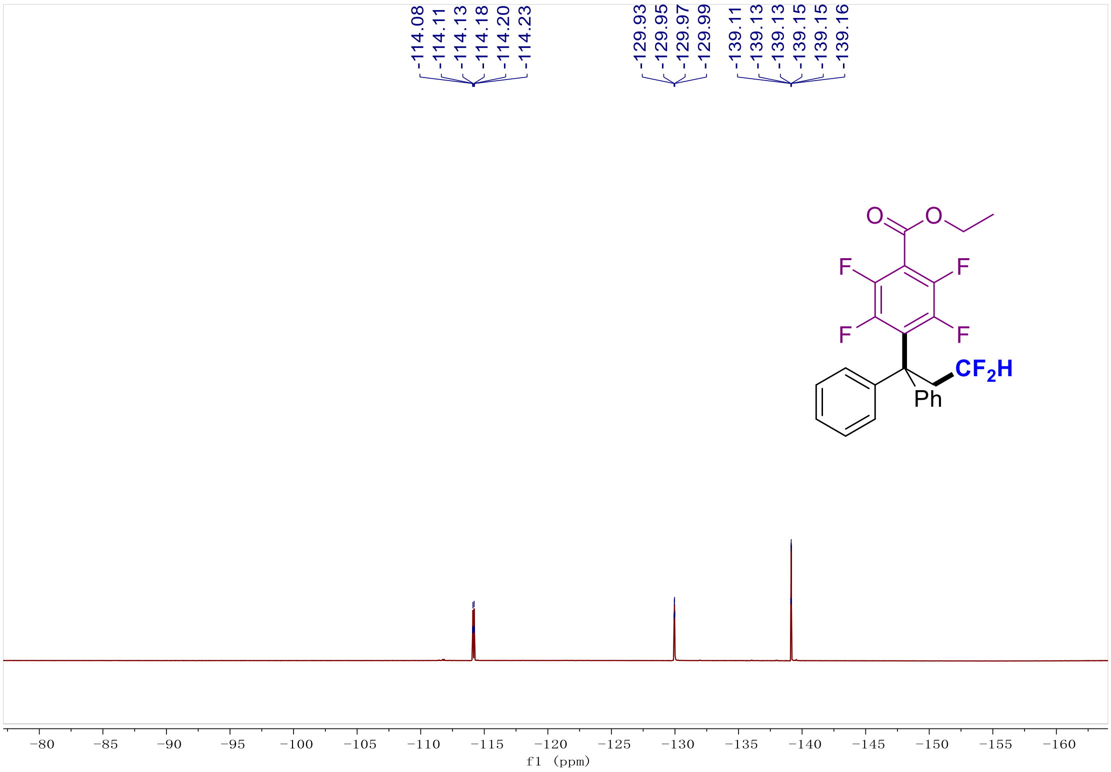

^{1}H NMR (600 MHz, $CDCl_3$) spectrum of 79

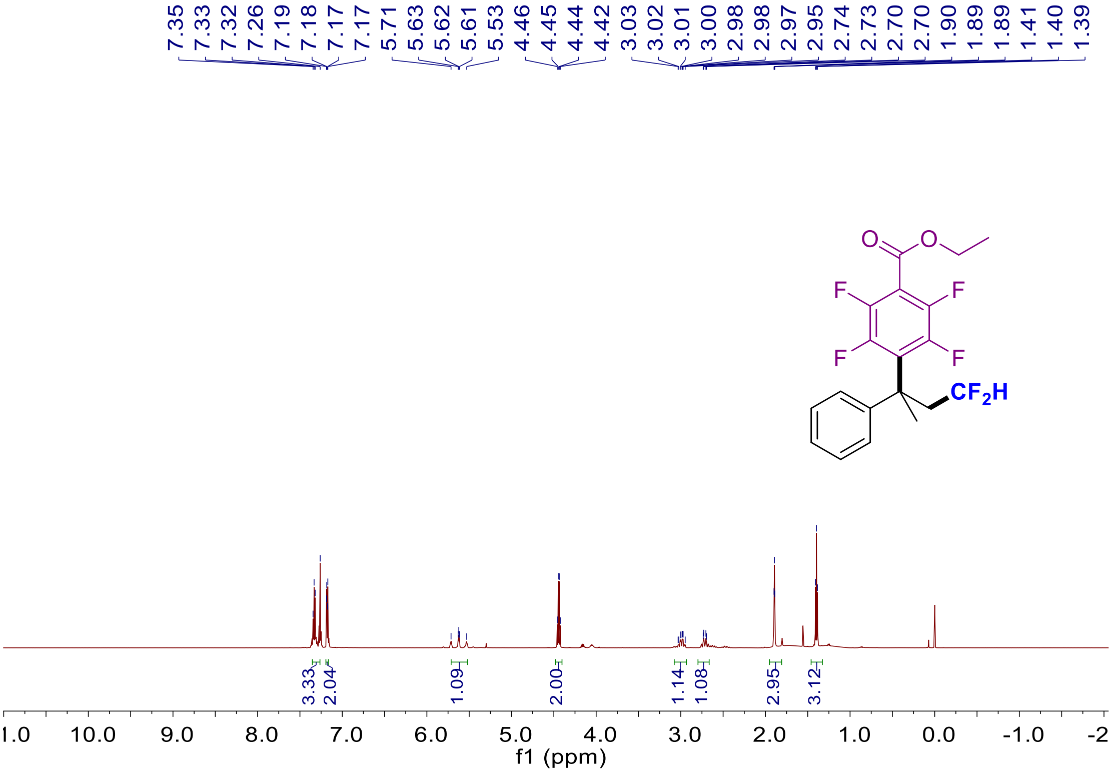

^{13}C NMR (151 MHz, $CDCl_3$) spectrum of 79

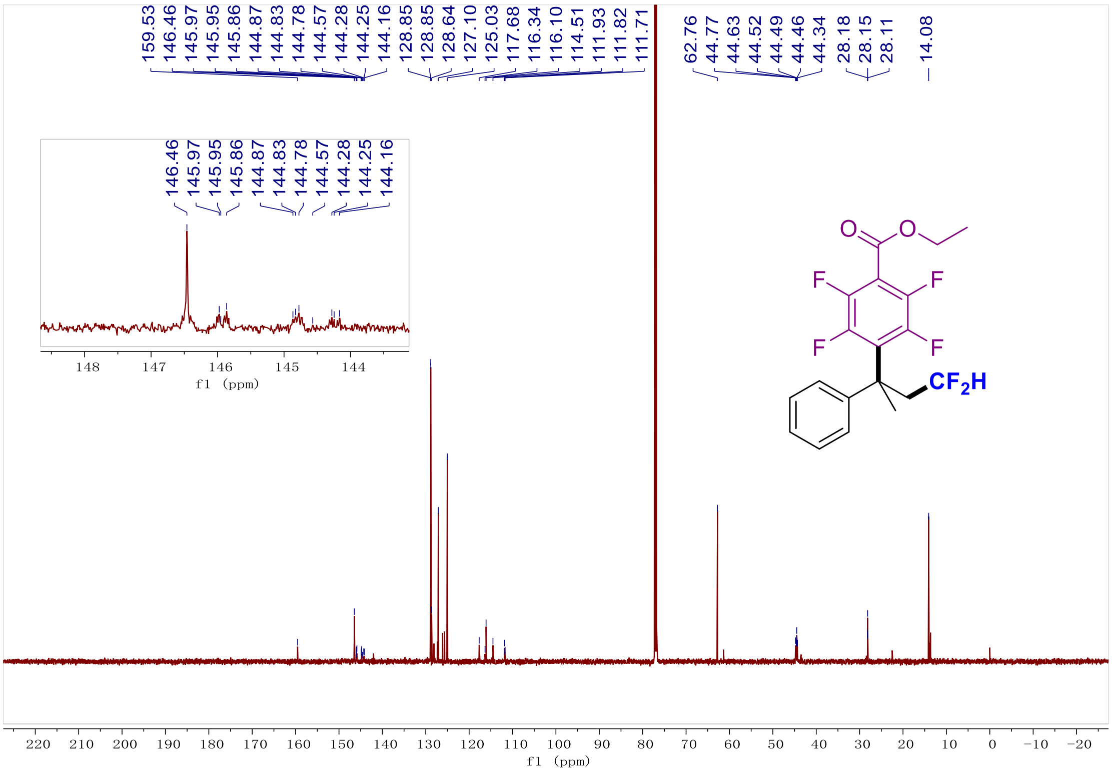

^{19}F NMR (565 MHz, $CDCl_3$) spectrum of 79

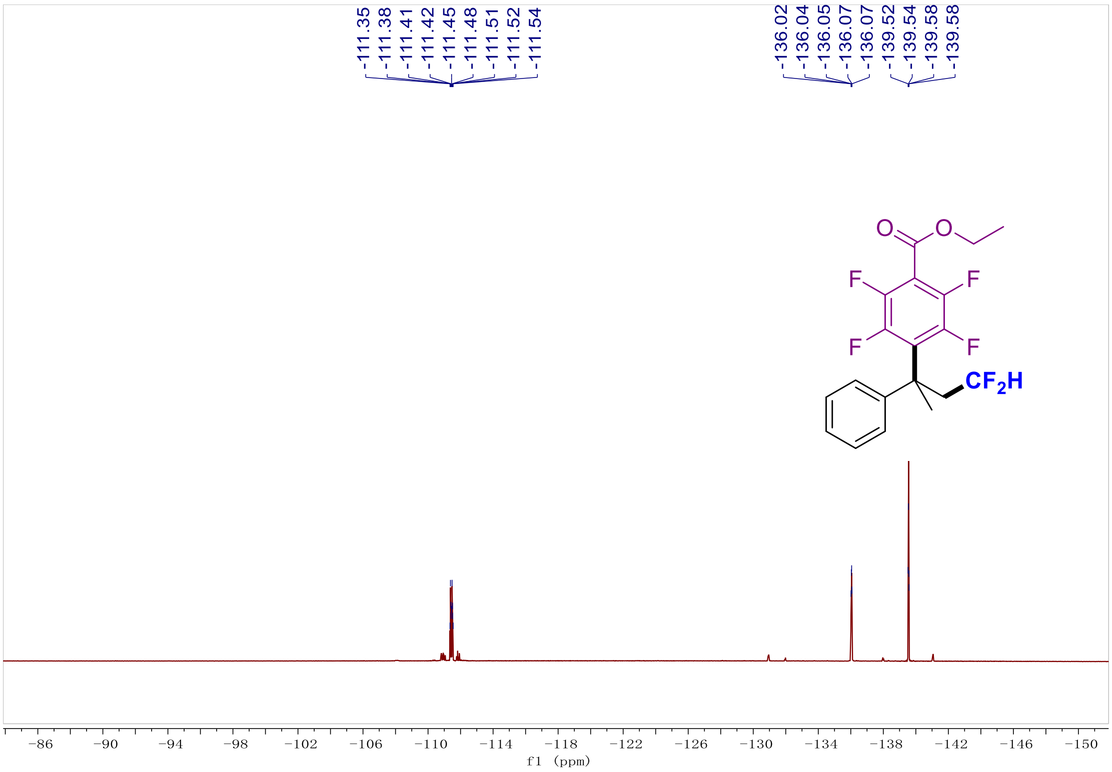

^{1}H NMR (600 MHz, $CDCl_3$) spectrum of 80

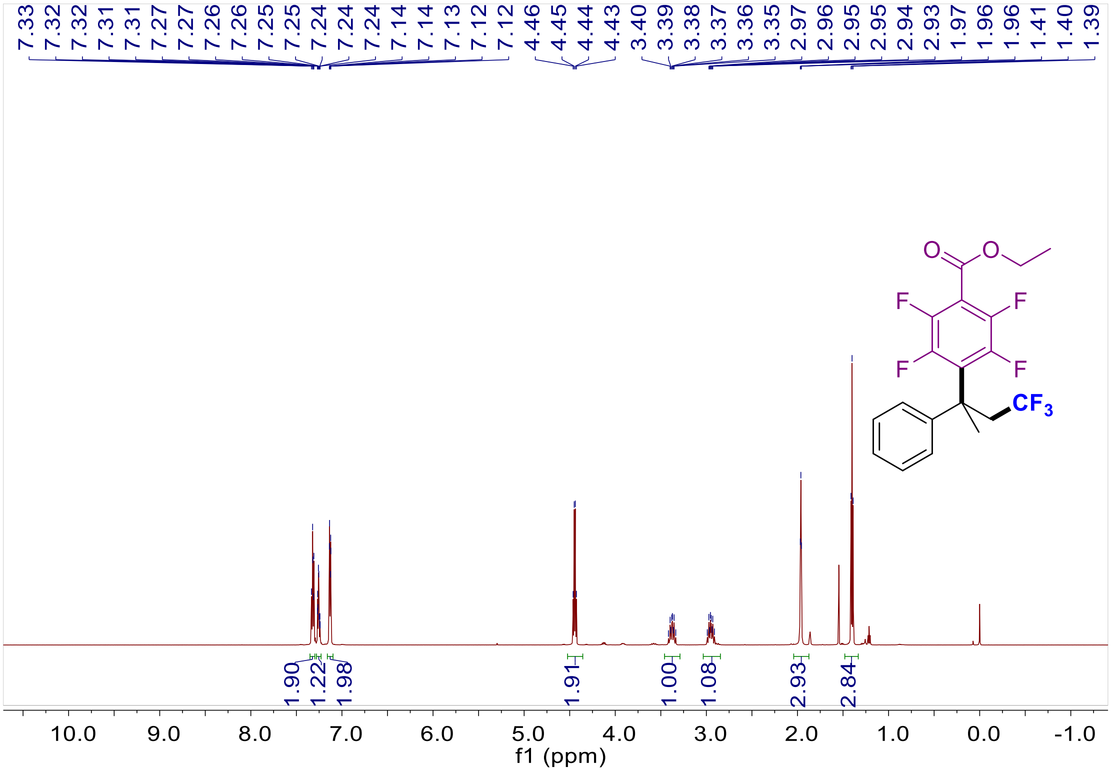

^{13}C NMR (151 MHz, $CDCl_3$) spectrum of 80

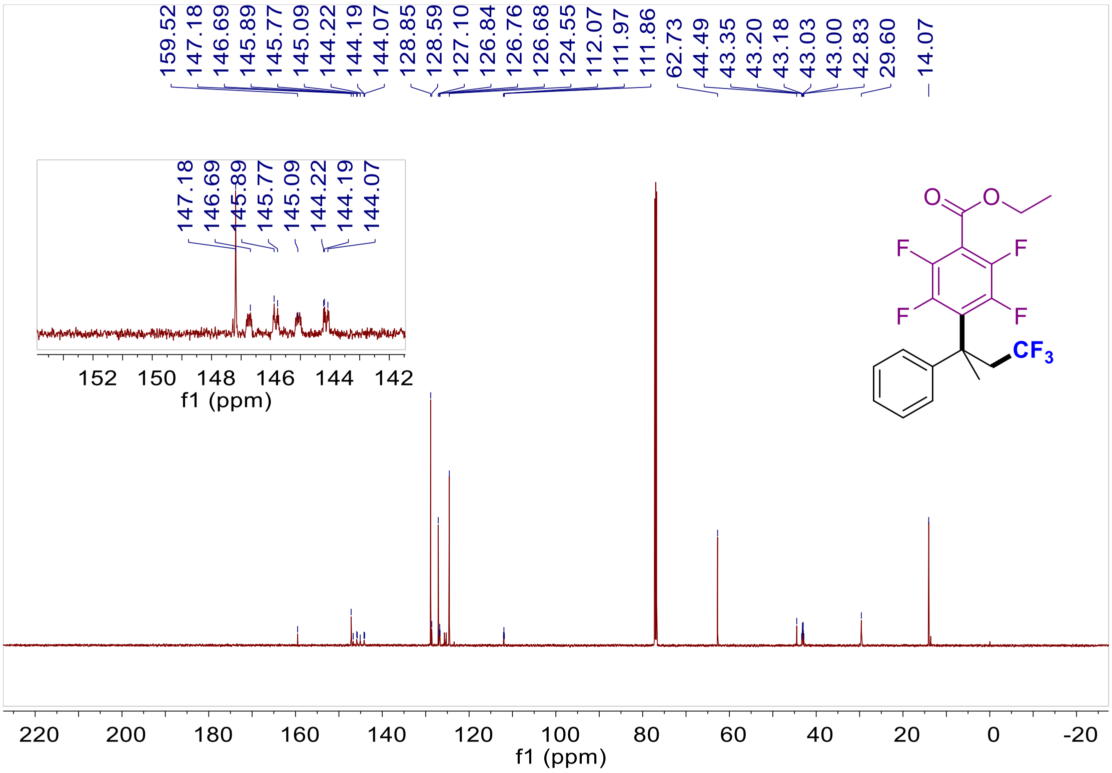

^{19}F NMR (565 MHz, CDCl$_3$) spectrum of 80

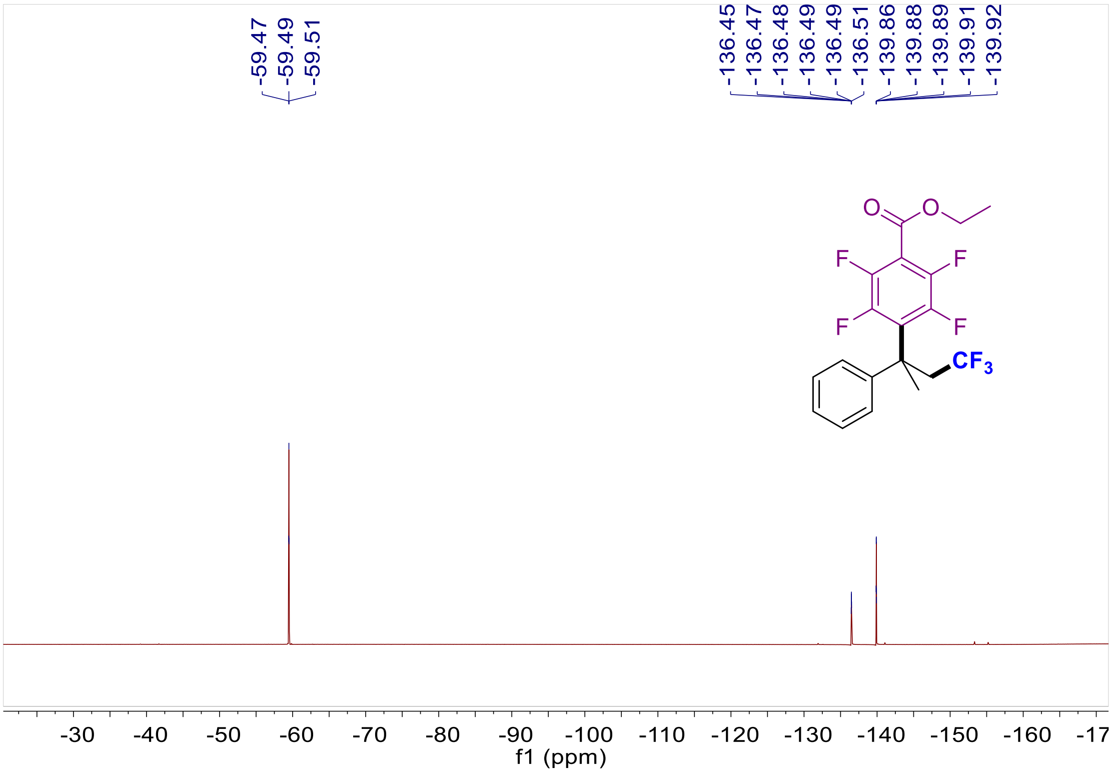

^{1}H NMR (600 MHz, CDCl$_3$) spectrum of 81

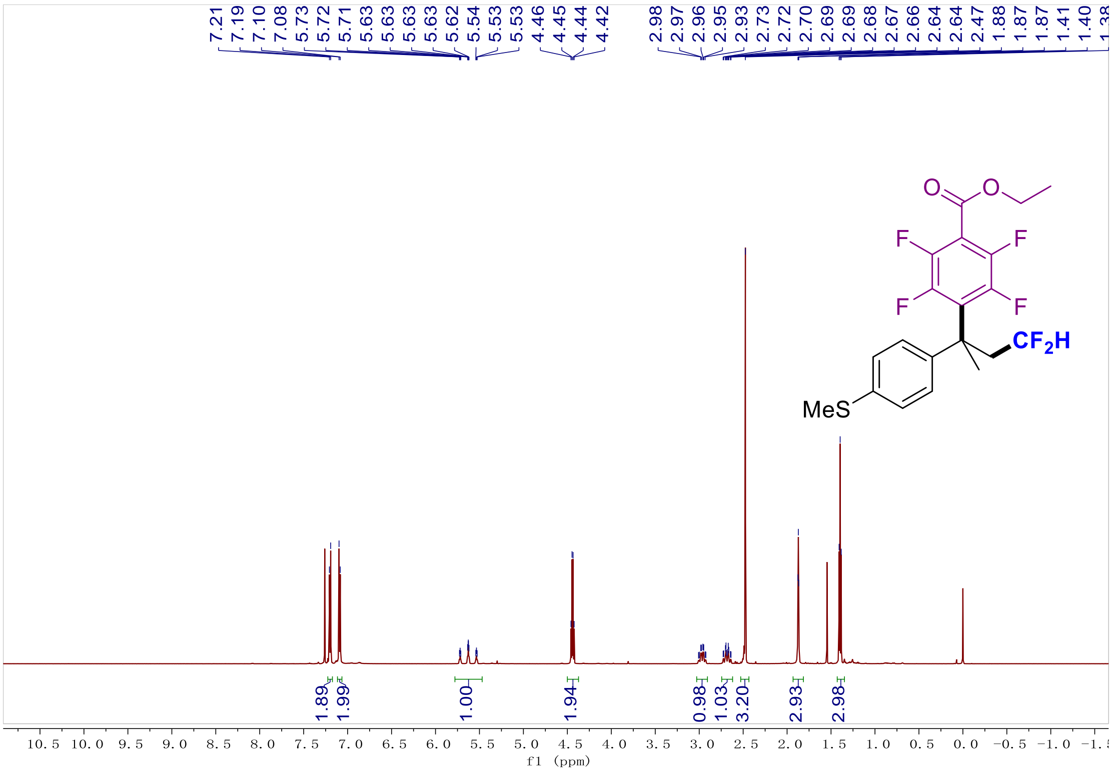

^{13}C NMR (151 MHz, $CDCl_3$) spectrum of 81

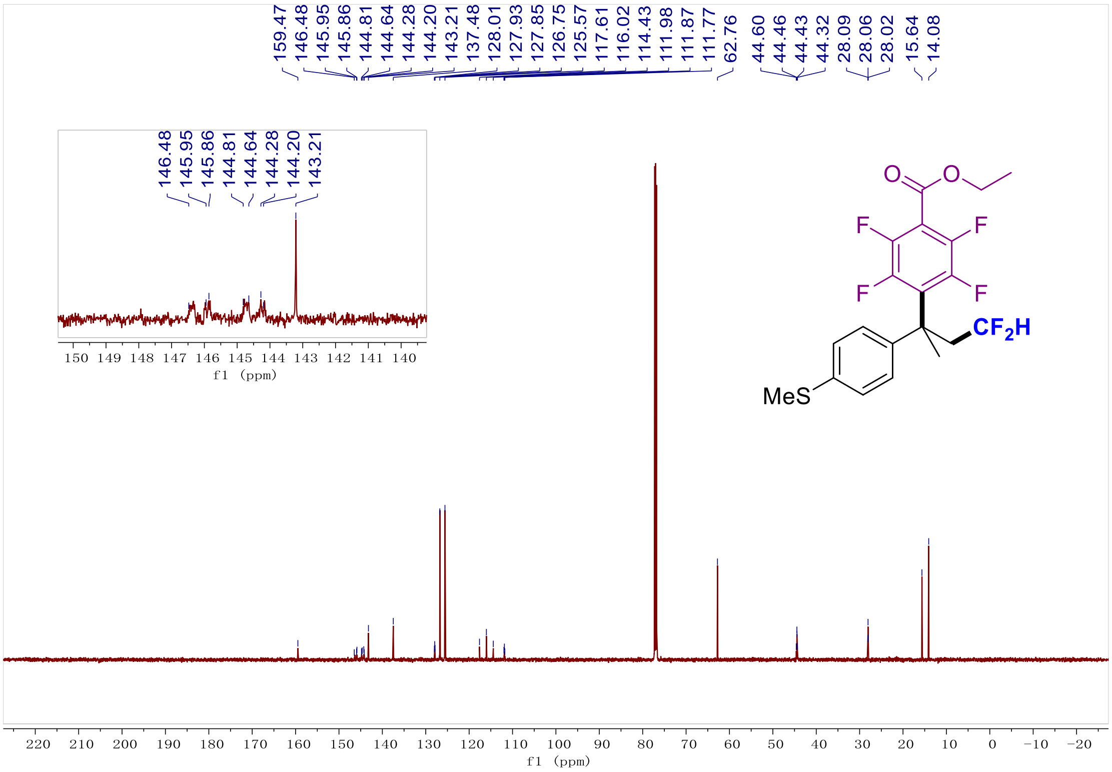

^{19}F NMR (565 MHz, $CDCl_3$) spectrum of 81

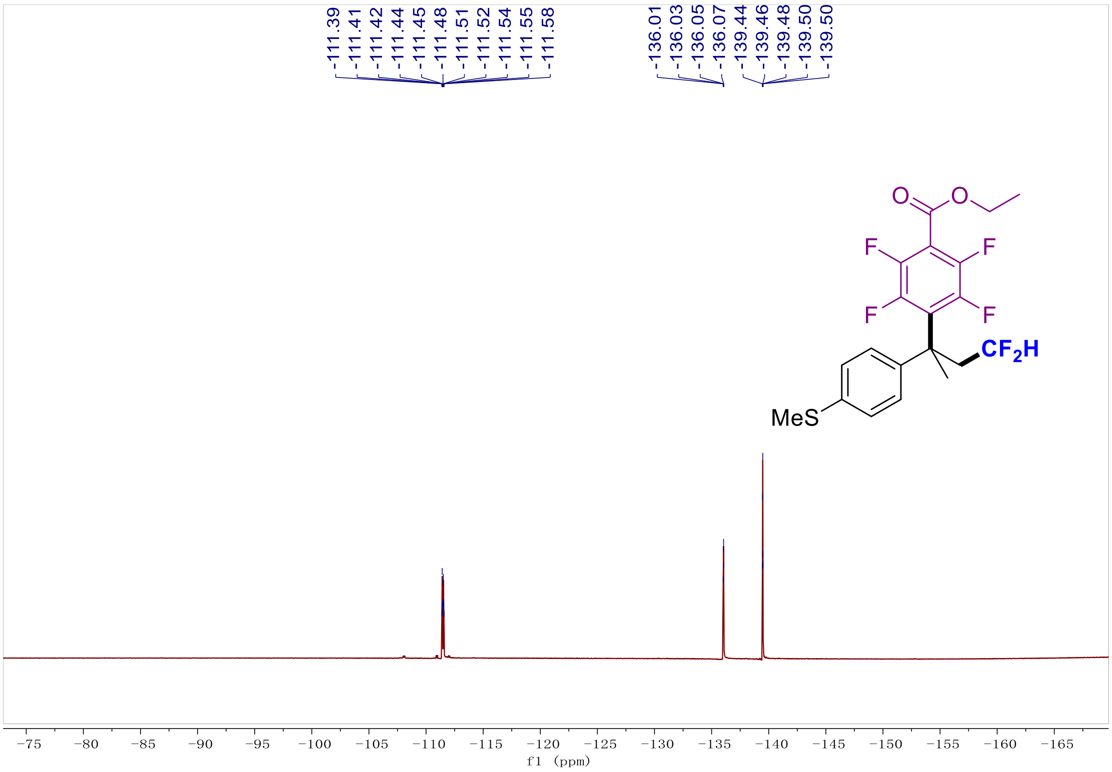

^{1}H NMR (600 MHz, CDCl3) spectrum of 82

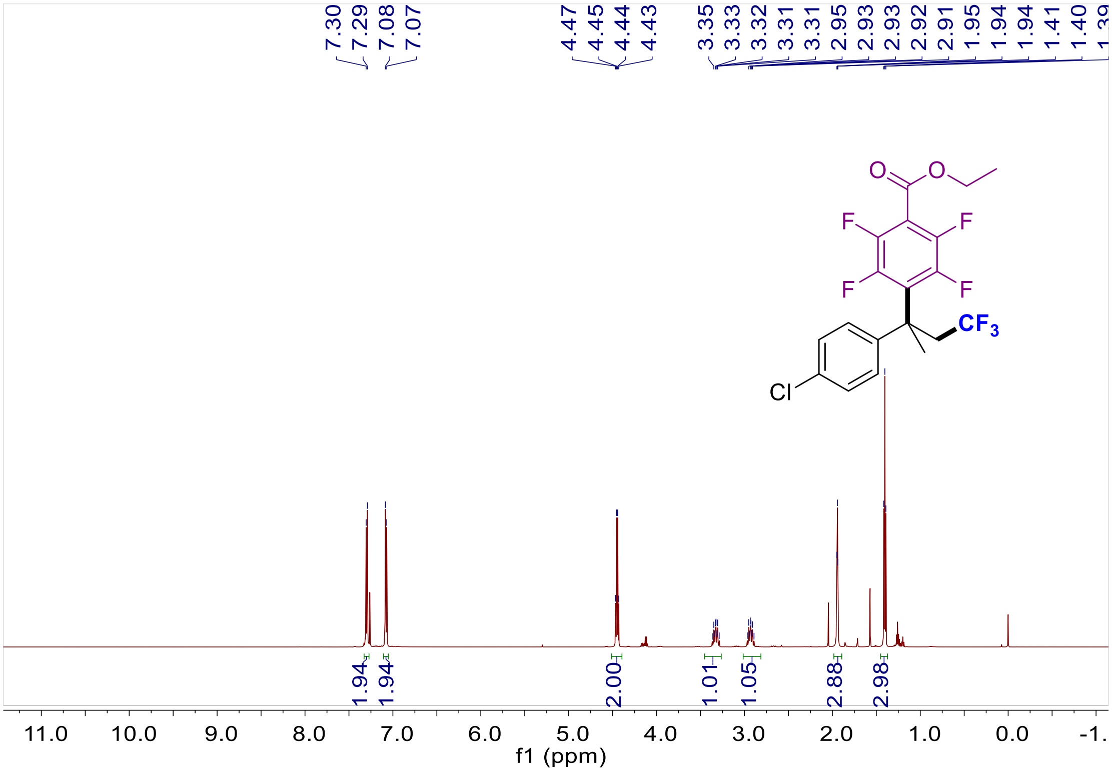

^{13}C NMR (151 MHz, CDCl3) spectrum of 82

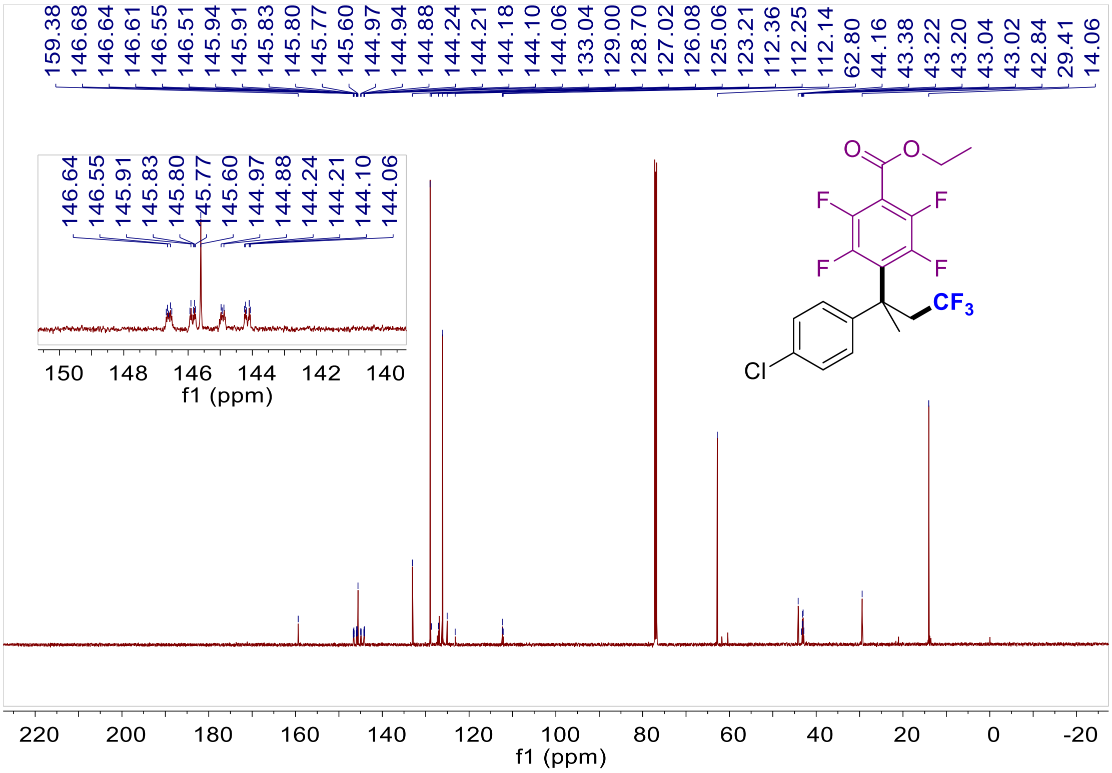

^{19}F NMR (565 MHz, $CDCl_3$) spectrum of 82

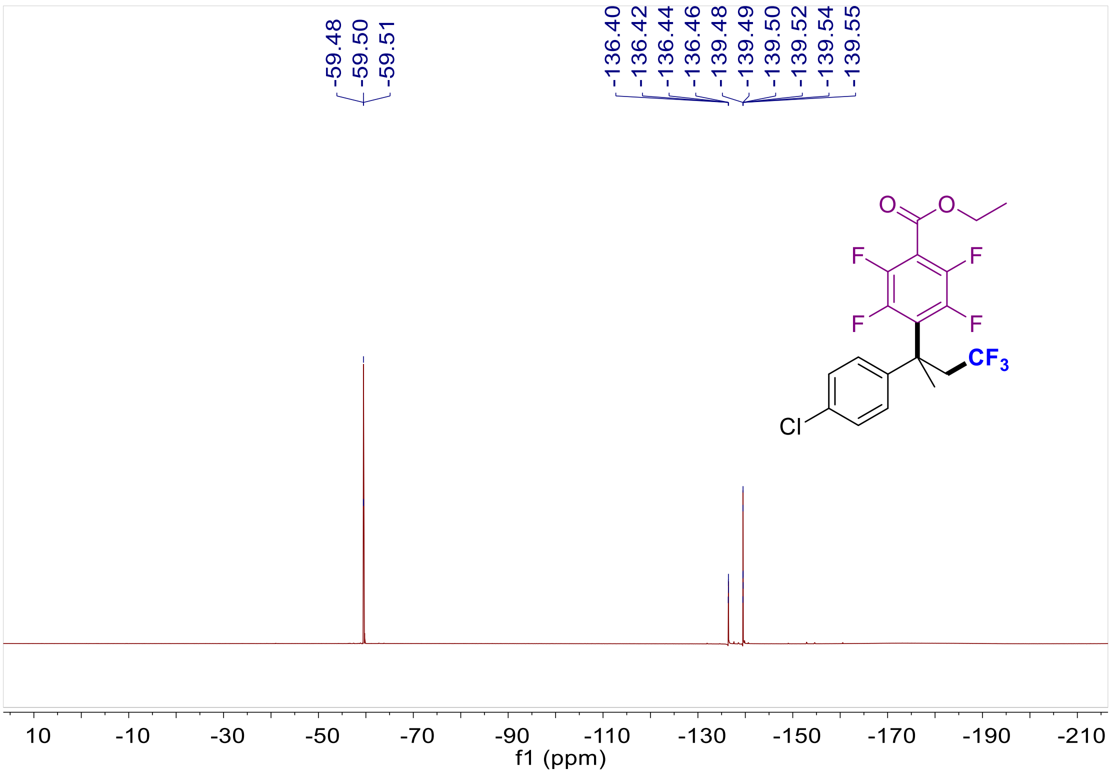

^{1}H NMR (600 MHz, $CDCl_3$) spectrum of 83

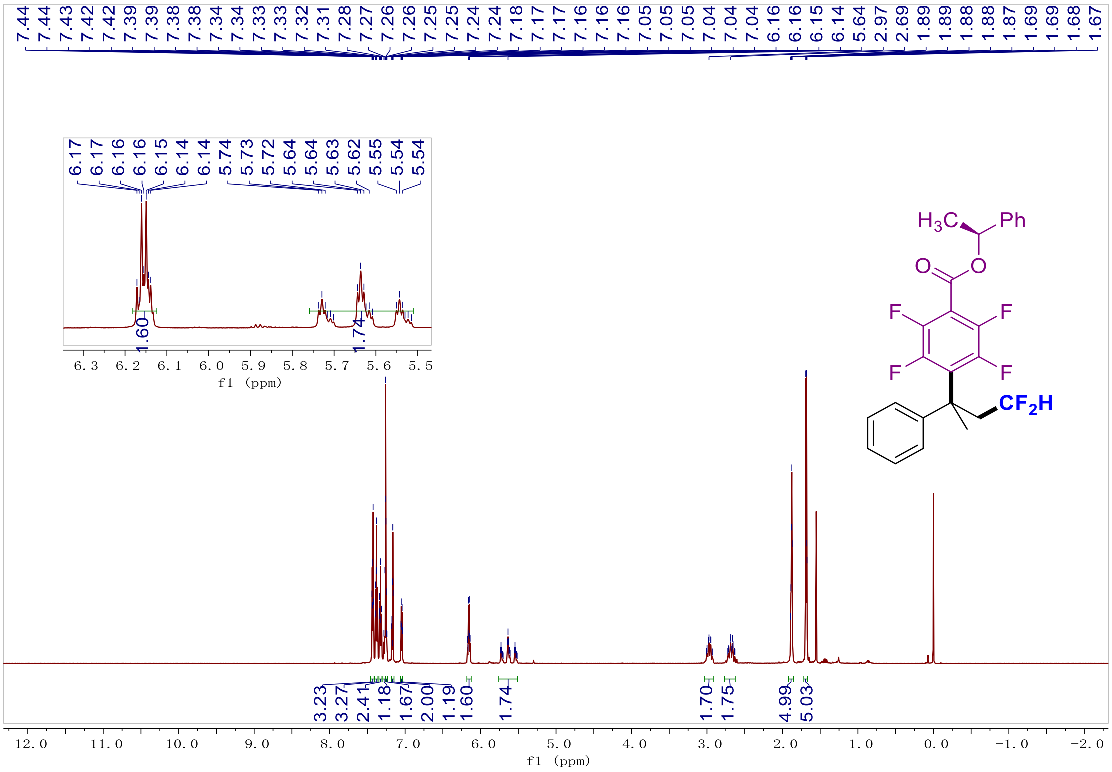

^{13}C NMR (151 MHz, CDCl$_3$) spectrum of 83

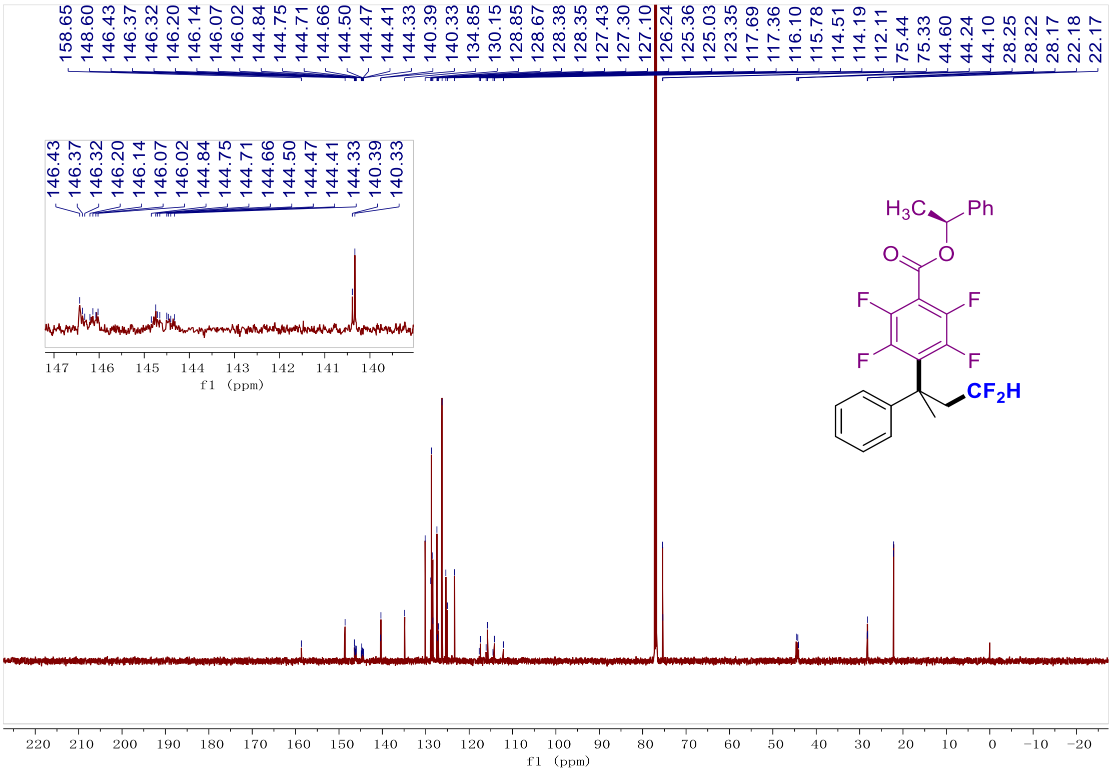

^{19}F NMR (565 MHz, CDCl$_3$) spectrum of 83

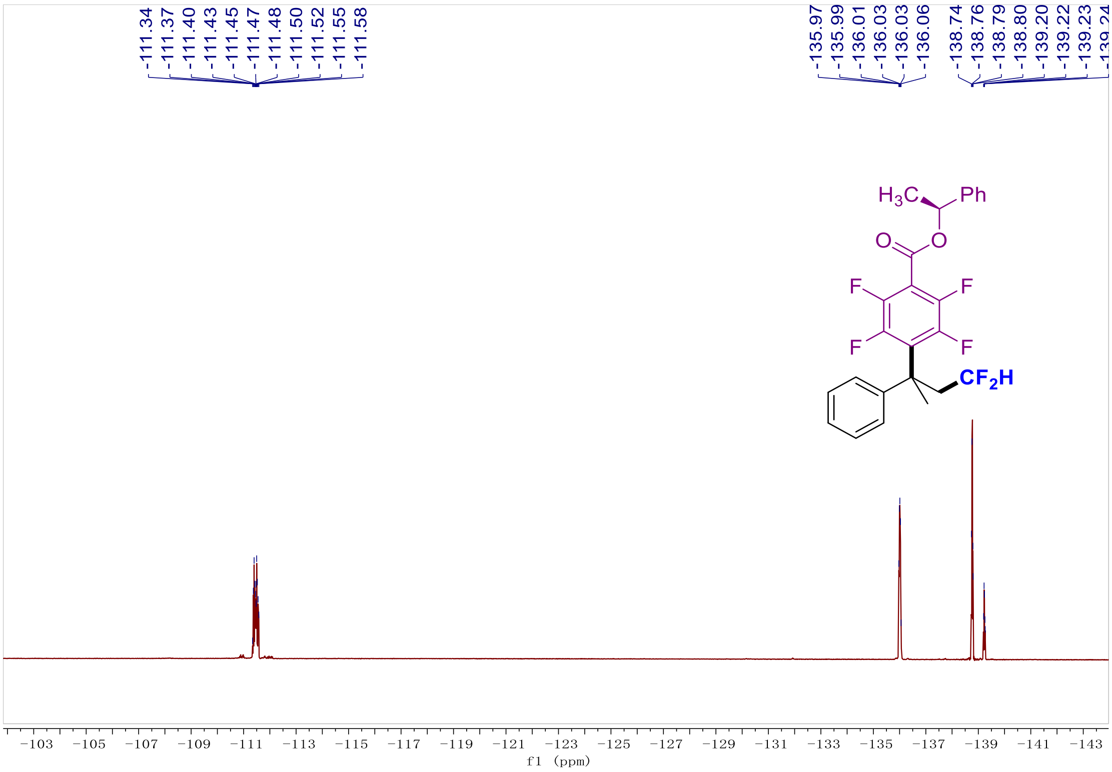

^{1}H NMR (600 MHz, $CDCl_3$) spectrum of 84

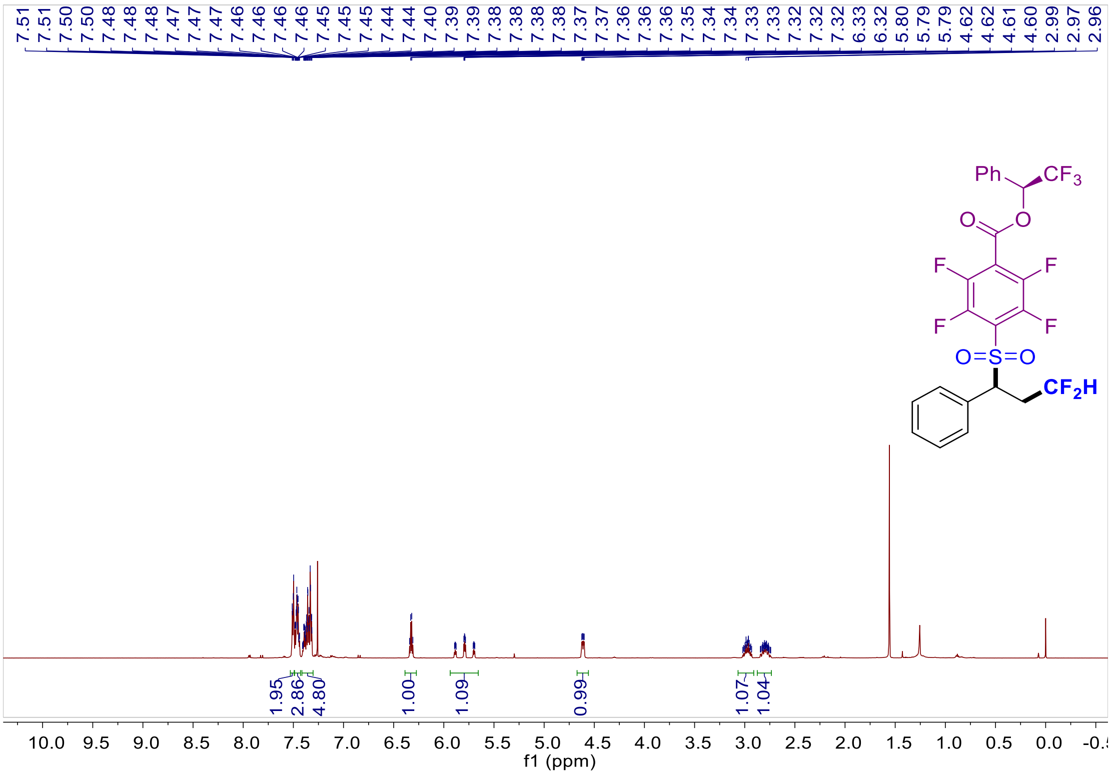

^{13}C NMR (151 MHz, $CDCl_3$) spectrum of 84

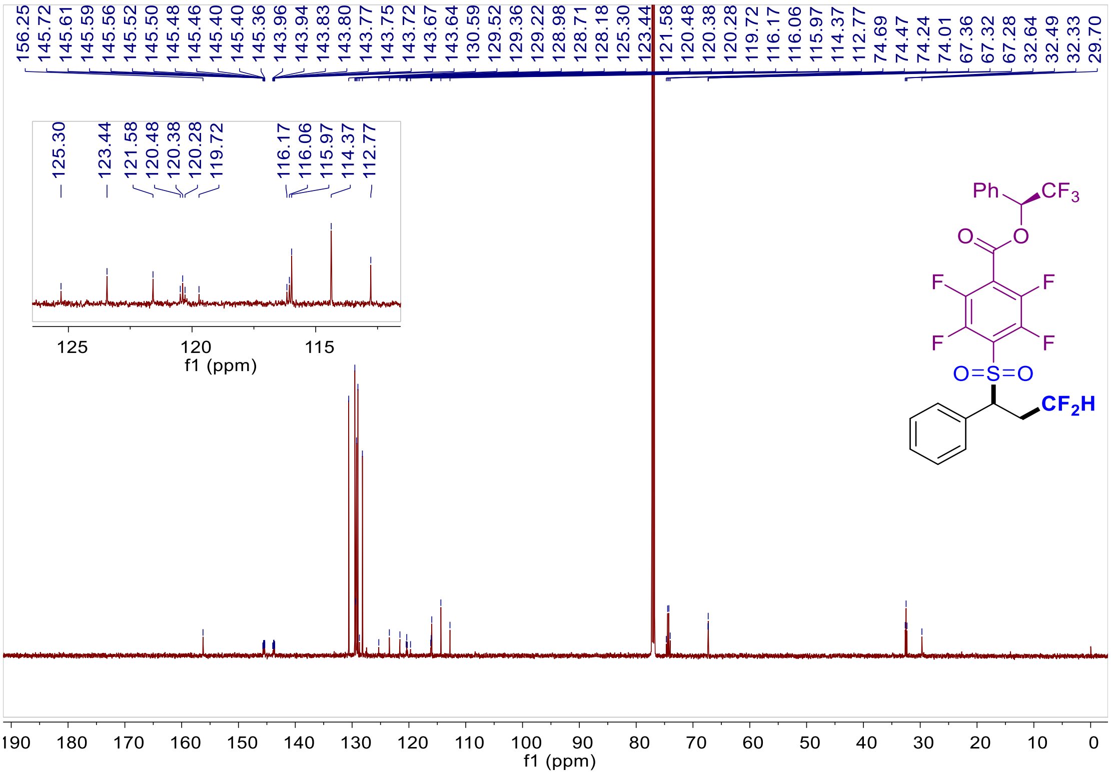

^{19}F NMR (565 MHz, CDCl$_3$) spectrum of 84

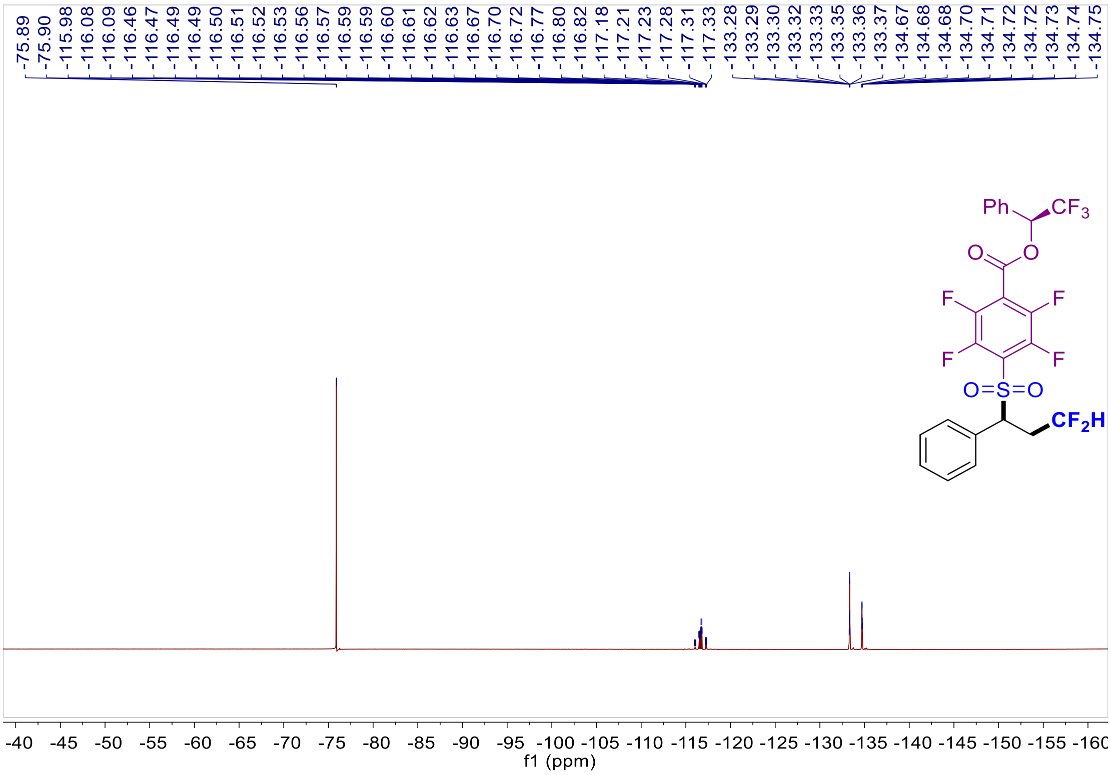

^{1}H NMR (600 MHz, CDCl$_3$) spectrum of 85

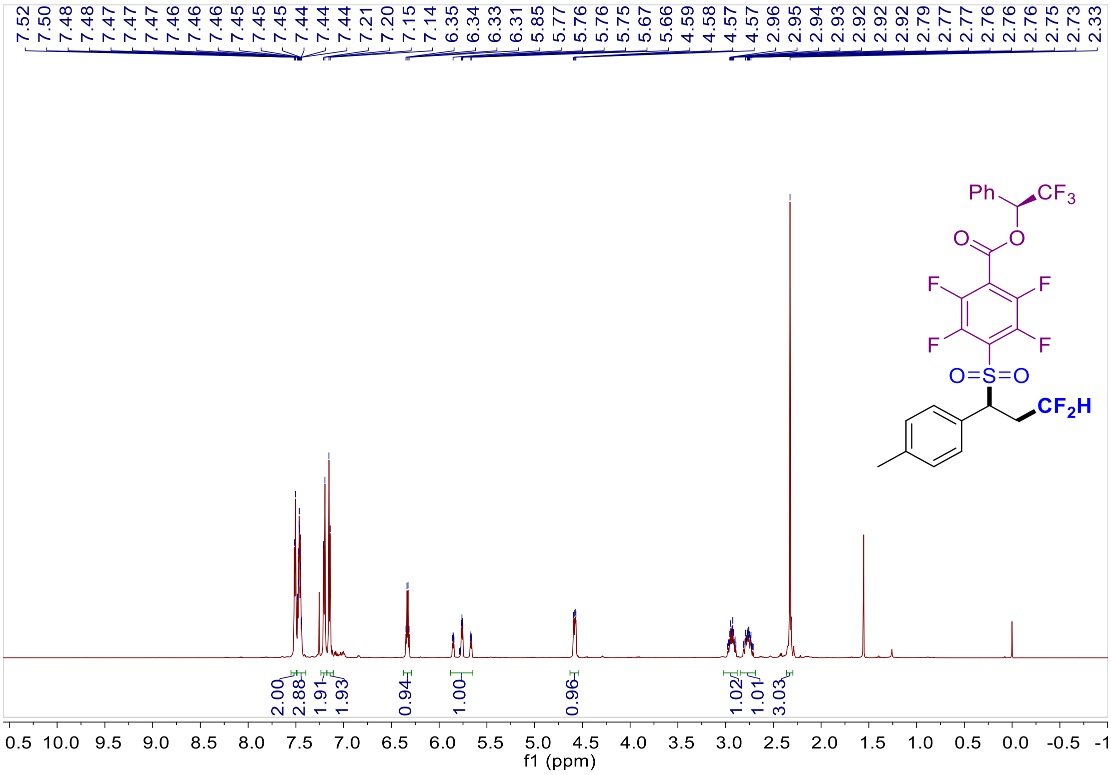

^{13}C NMR (151 MHz, CDCl$_3$) spectrum of 85

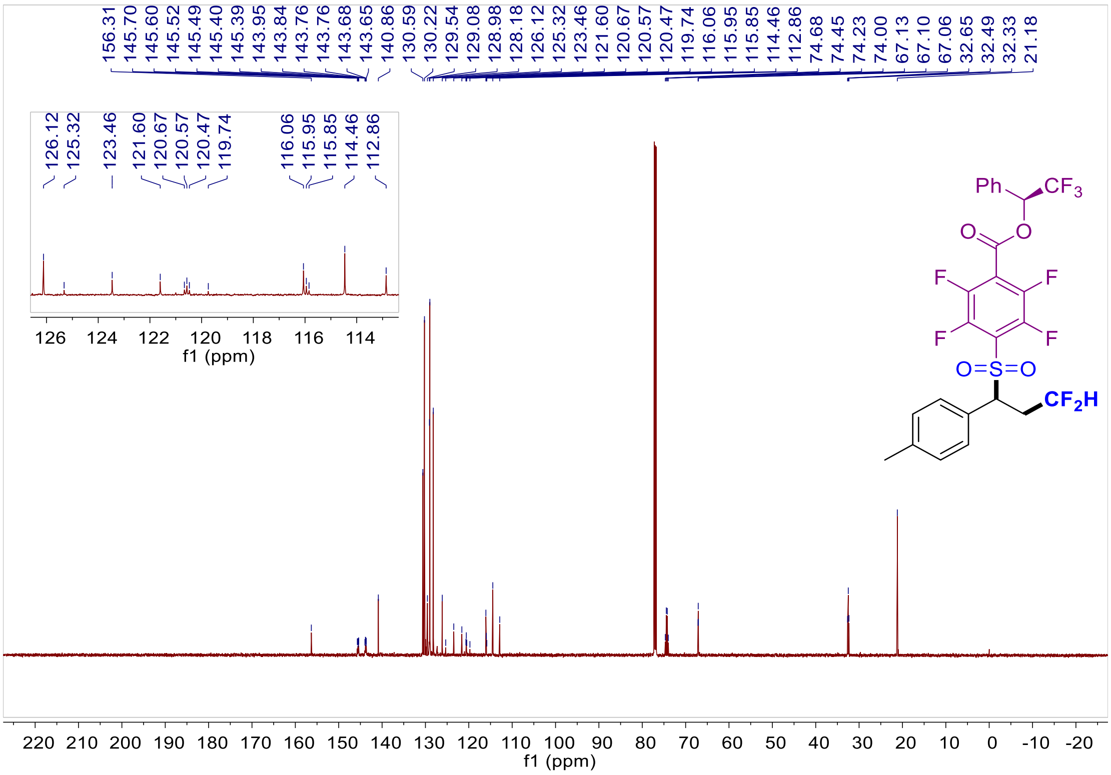

^{19}F NMR (565 MHz, CDCl$_3$) spectrum of 85

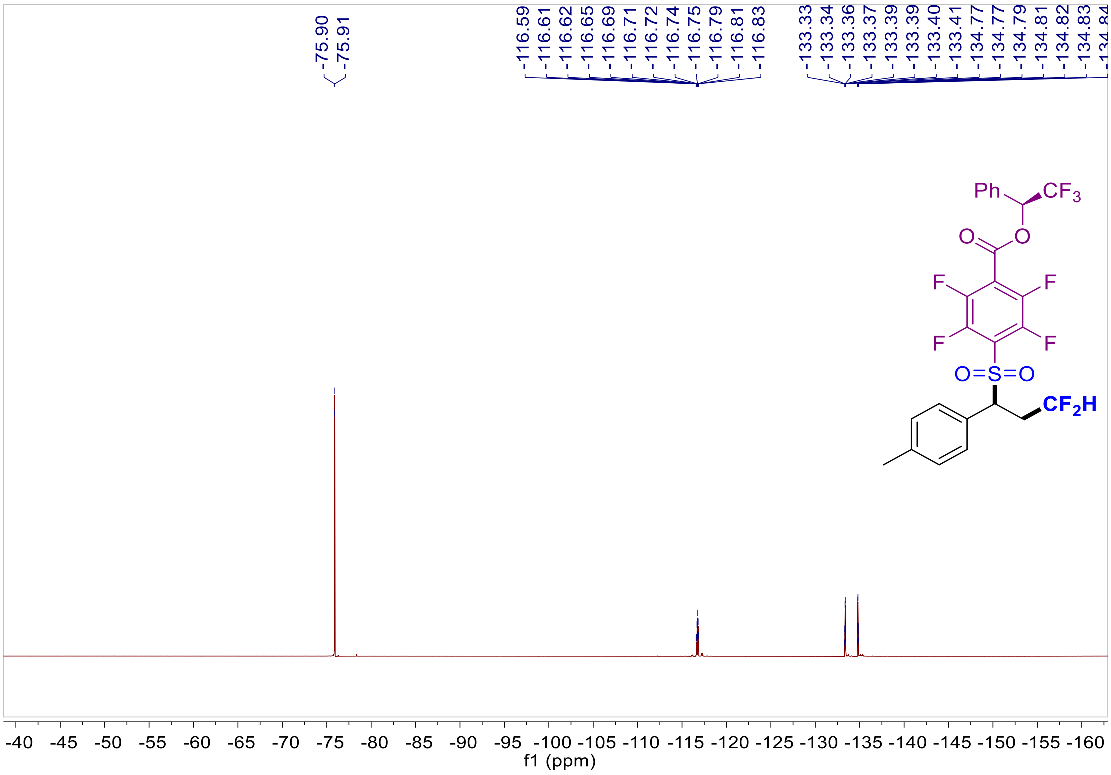

^{1}H NMR (600 MHz, CDCl3) spectrum of 86

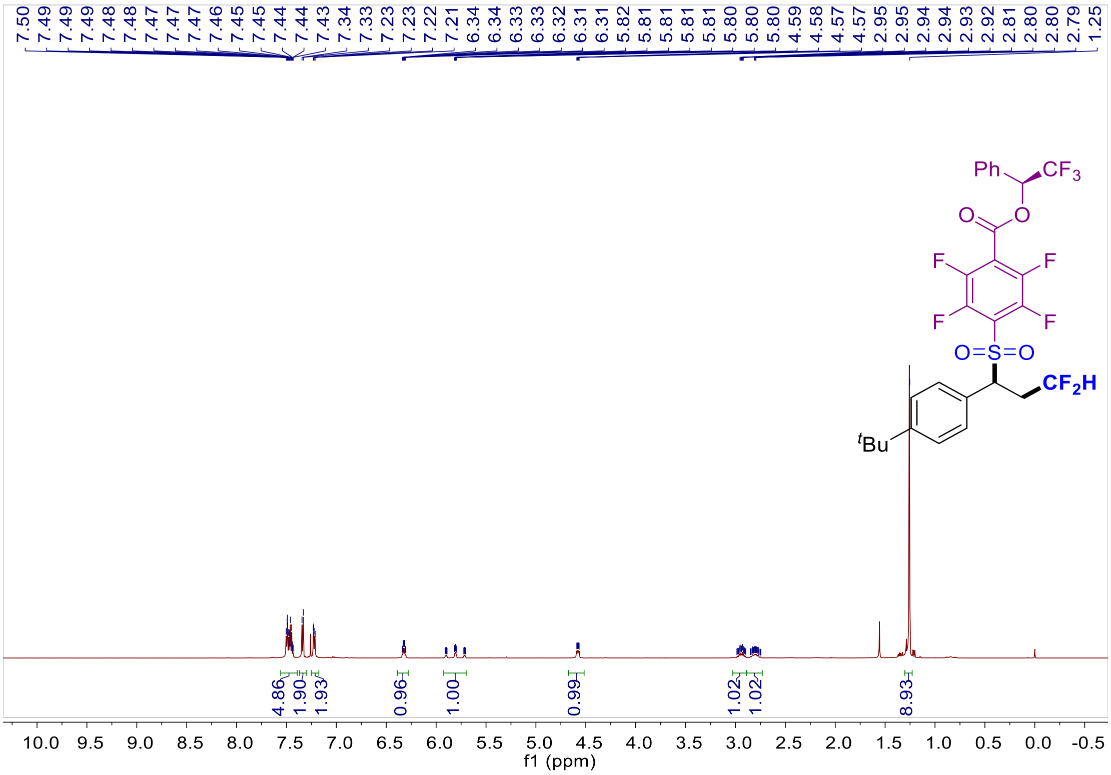

^{13}C NMR (151 MHz, CDCl3) spectrum of 86

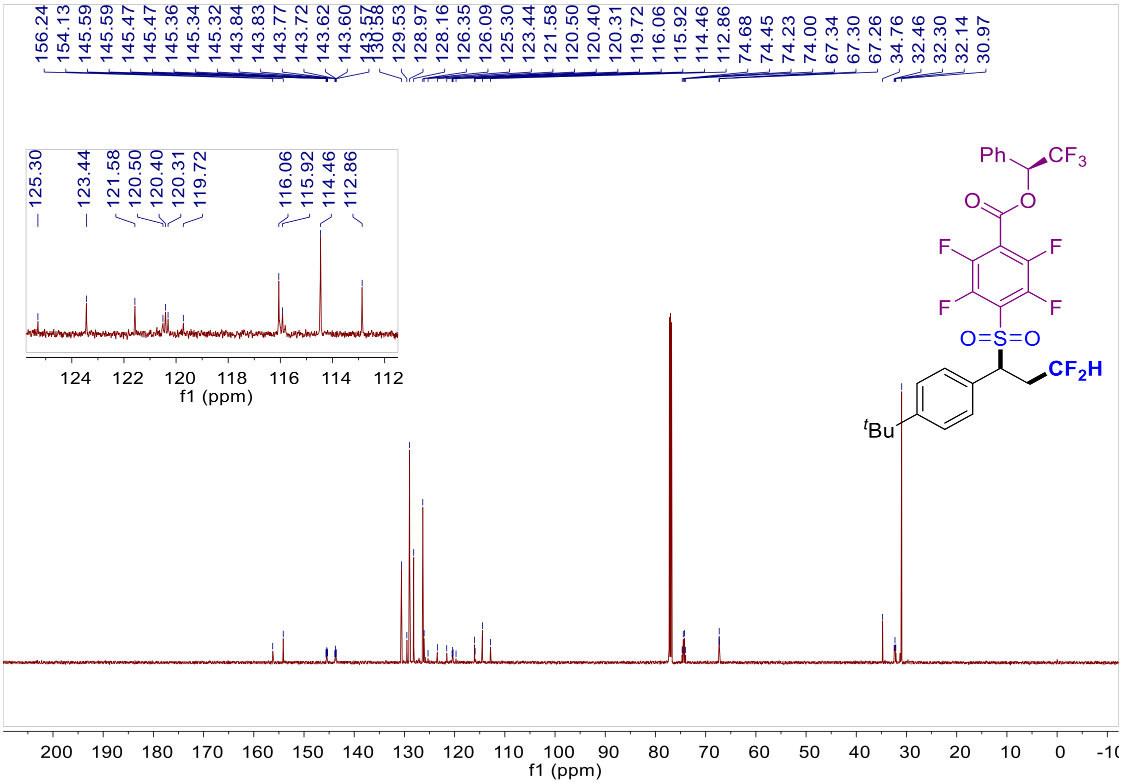

^{19}F NMR (565 MHz, CDCl$_3$) spectrum of 86

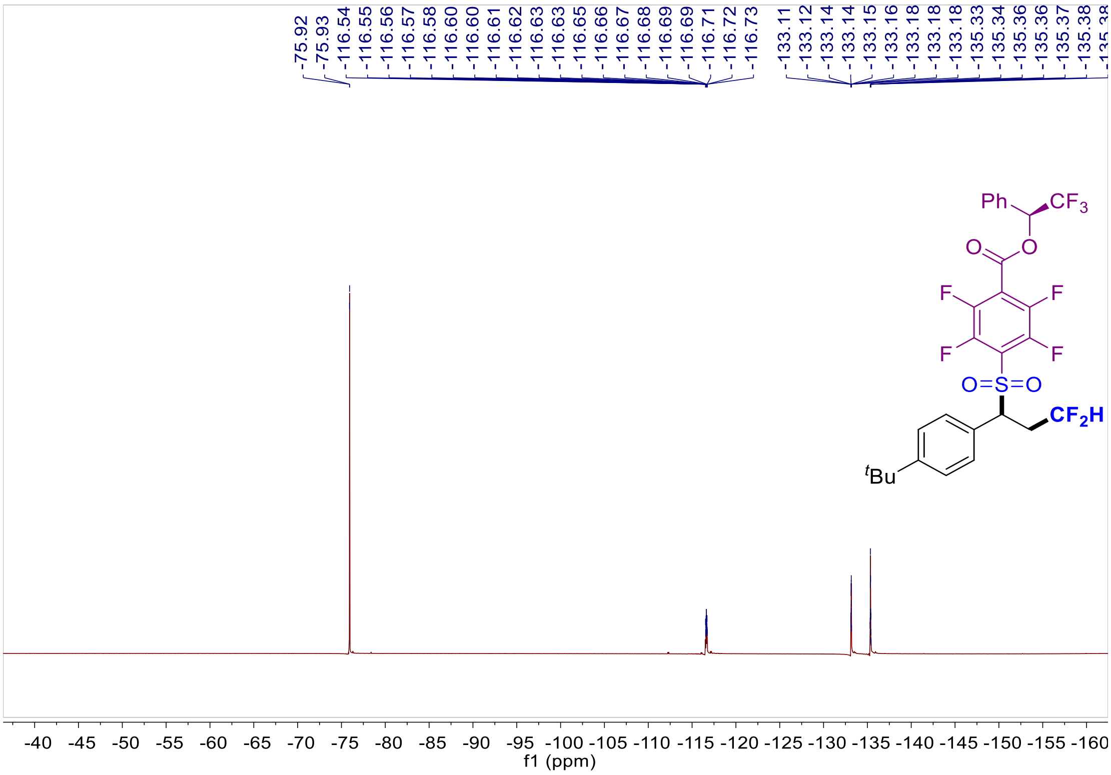

^{1}H NMR (600 MHz, CDCl$_3$) spectrum of 87

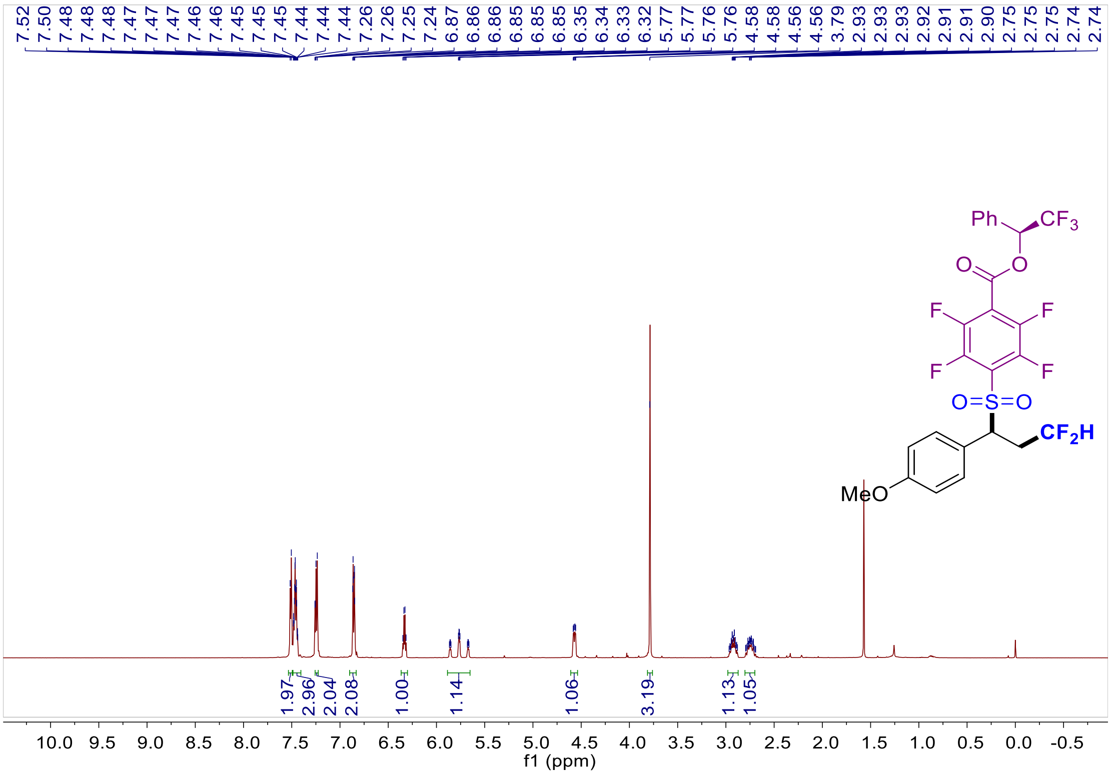

^{13}C NMR (151 MHz, $CDCl_3$) spectrum of 87

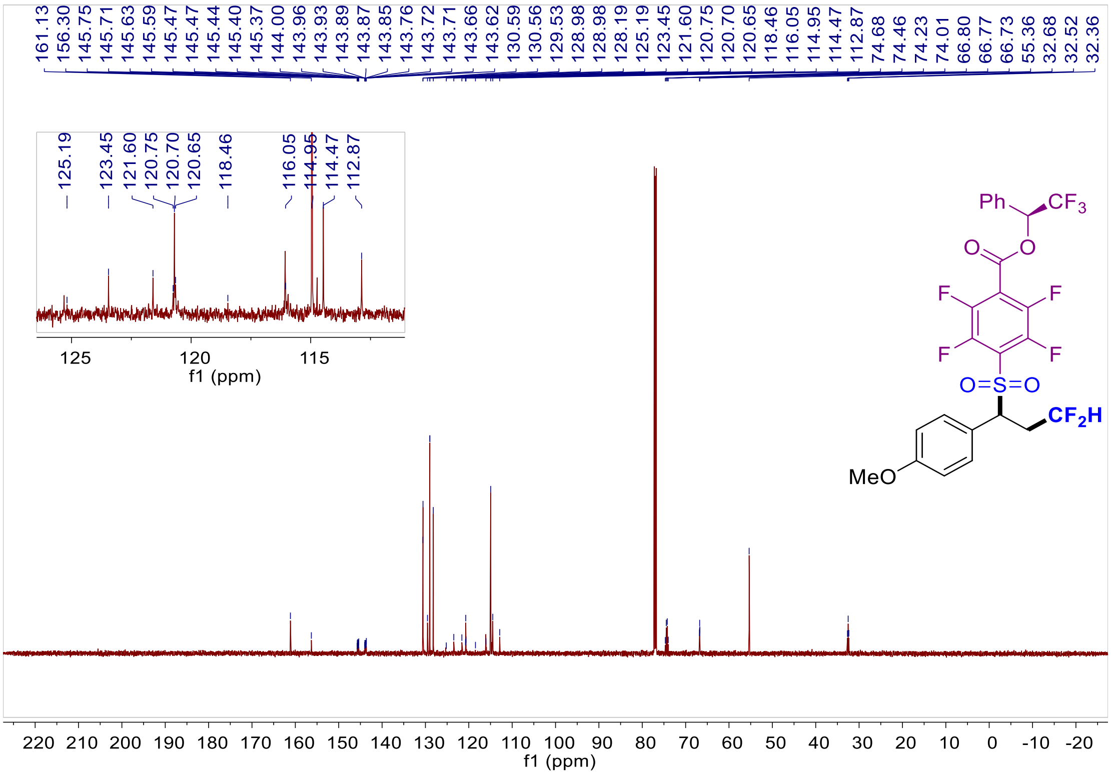

^{19}F NMR (565 MHz, $CDCl_3$) spectrum of 87

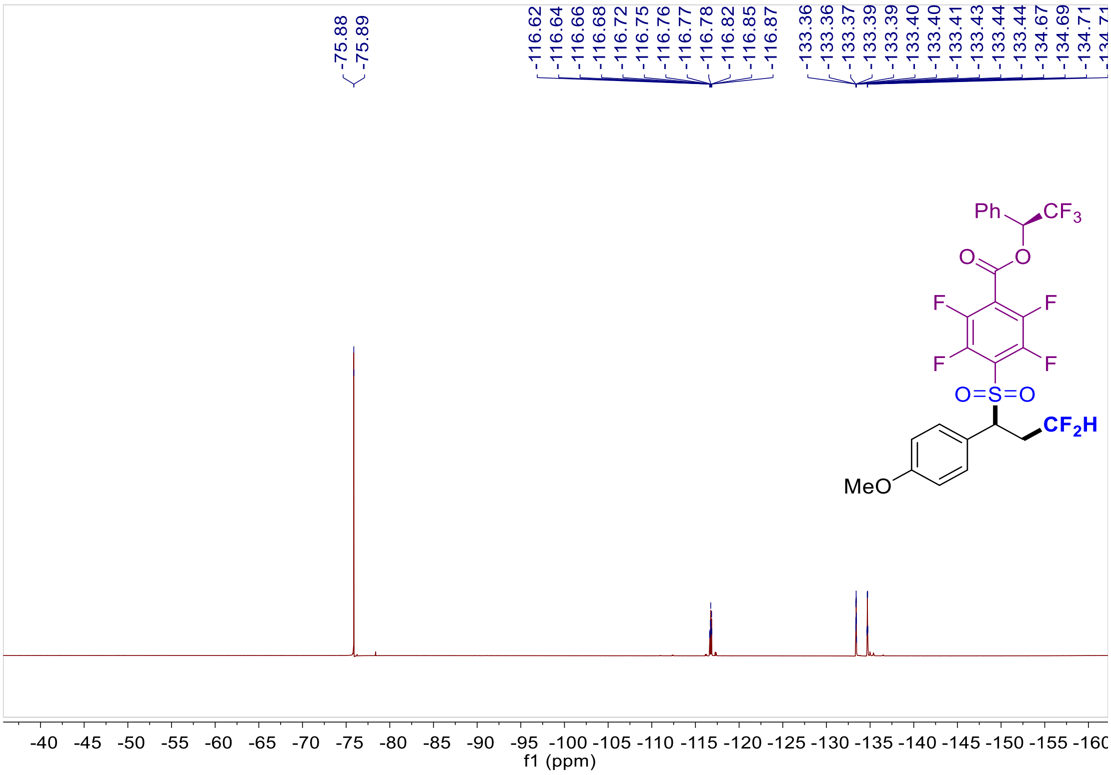

^{1}H NMR (600 MHz, CDCl$_3$) spectrum of 88

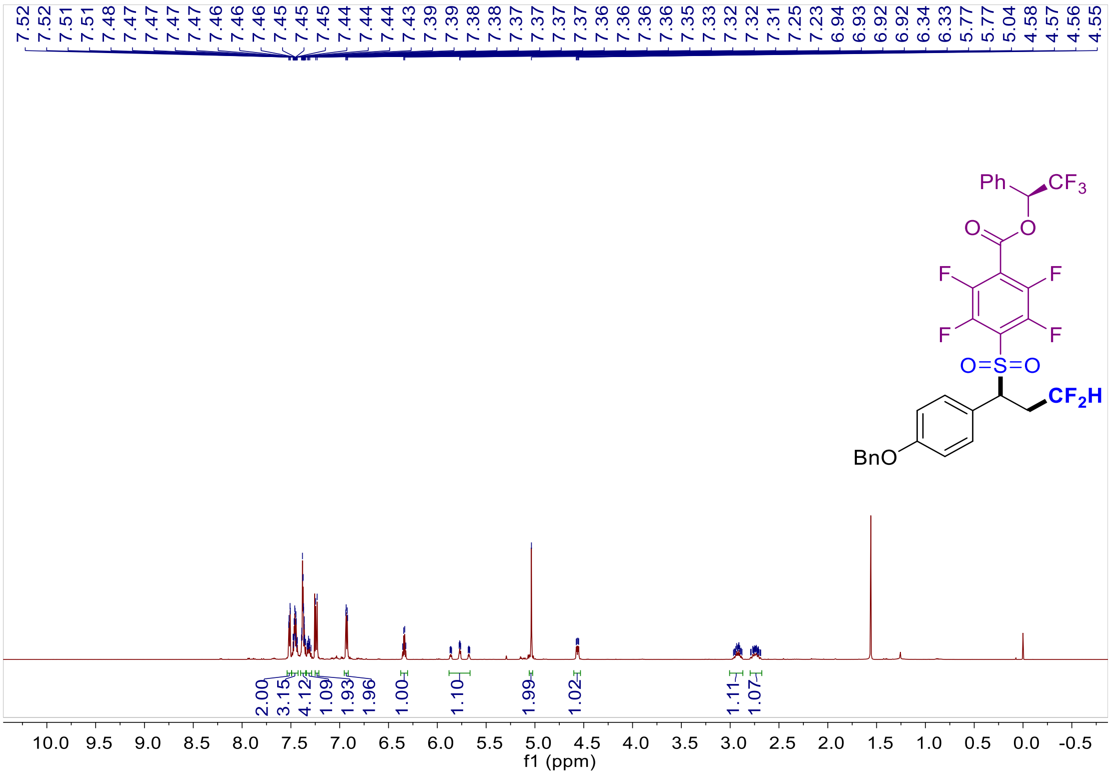

^{13}C NMR (151 MHz, CDCl$_3$) spectrum of 88

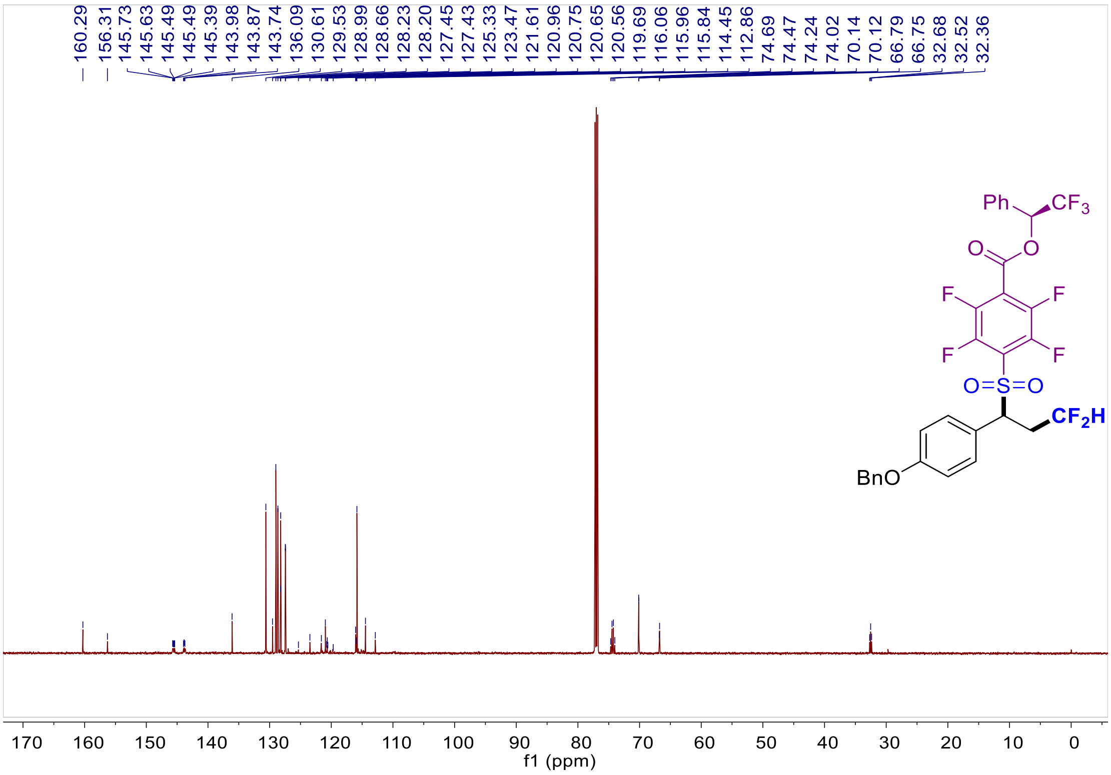

^{19}F NMR (565 MHz, CDCl$_3$) spectrum of 88

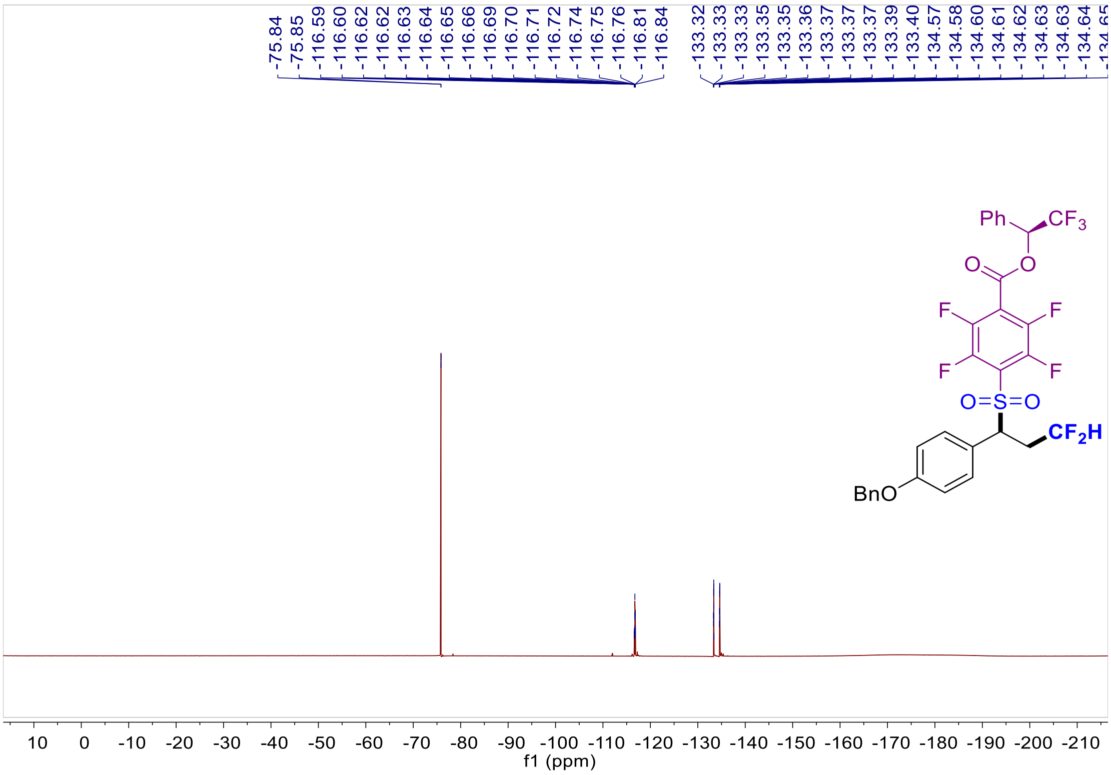

^{1}H NMR (600 MHz, CDCl$_3$) spectrum of 89

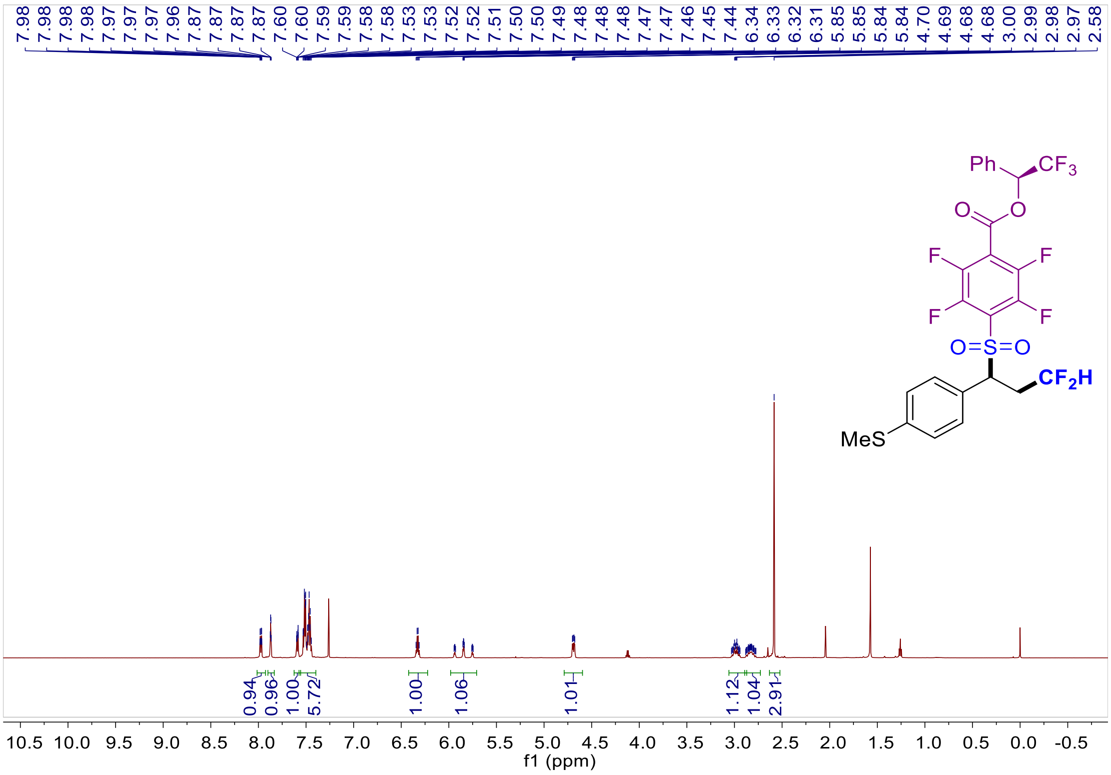

^{13}C NMR (151 MHz, CDCl$_3$) spectrum of 89

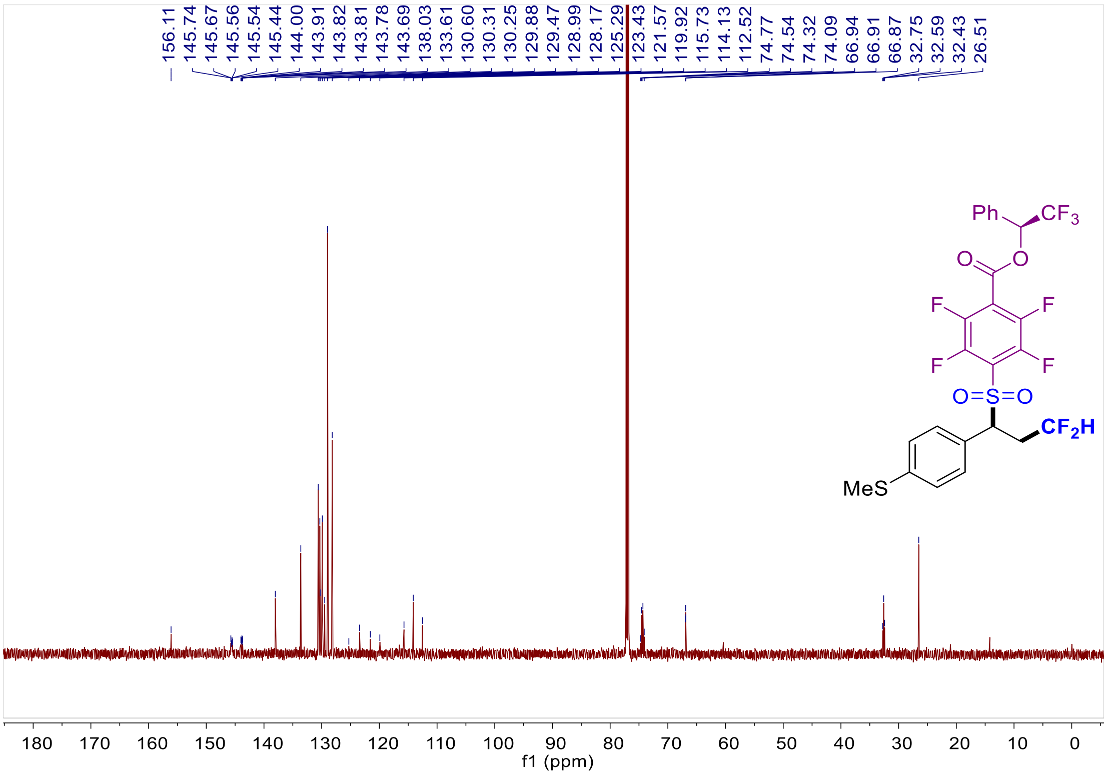

^{19}F NMR (565 MHz, CDCl$_3$) spectrum of 89

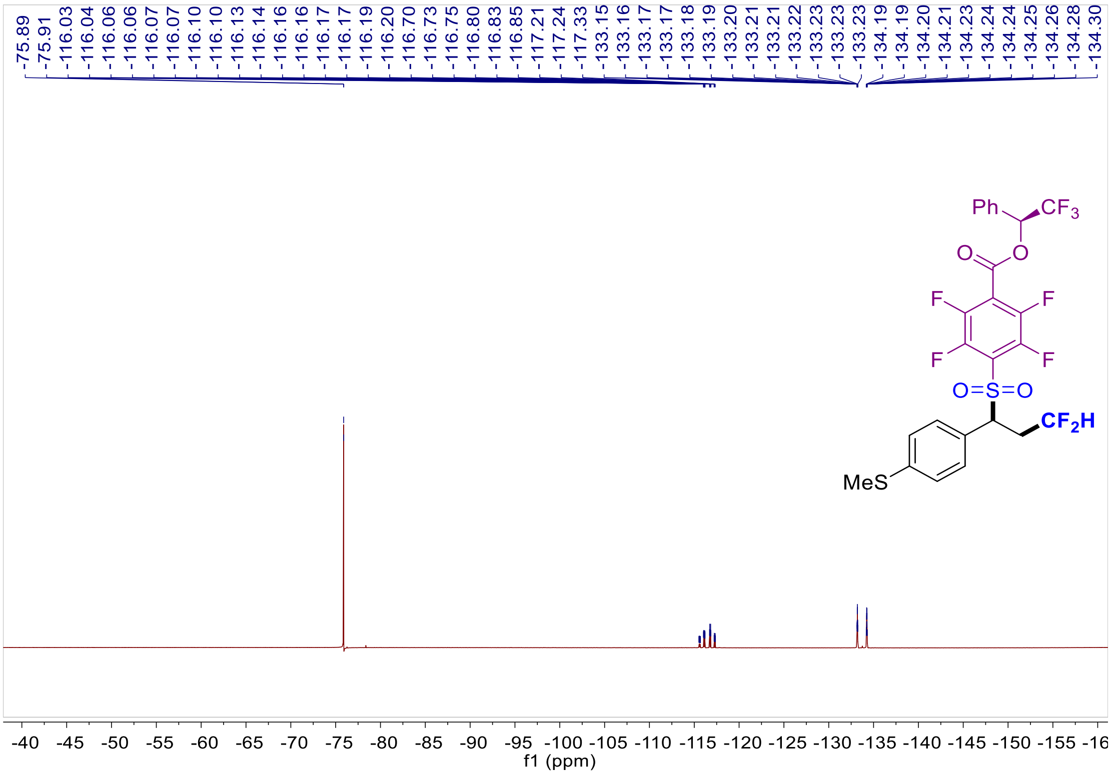

^{1}H NMR (600 MHz, CDCl₃) spectrum of 90

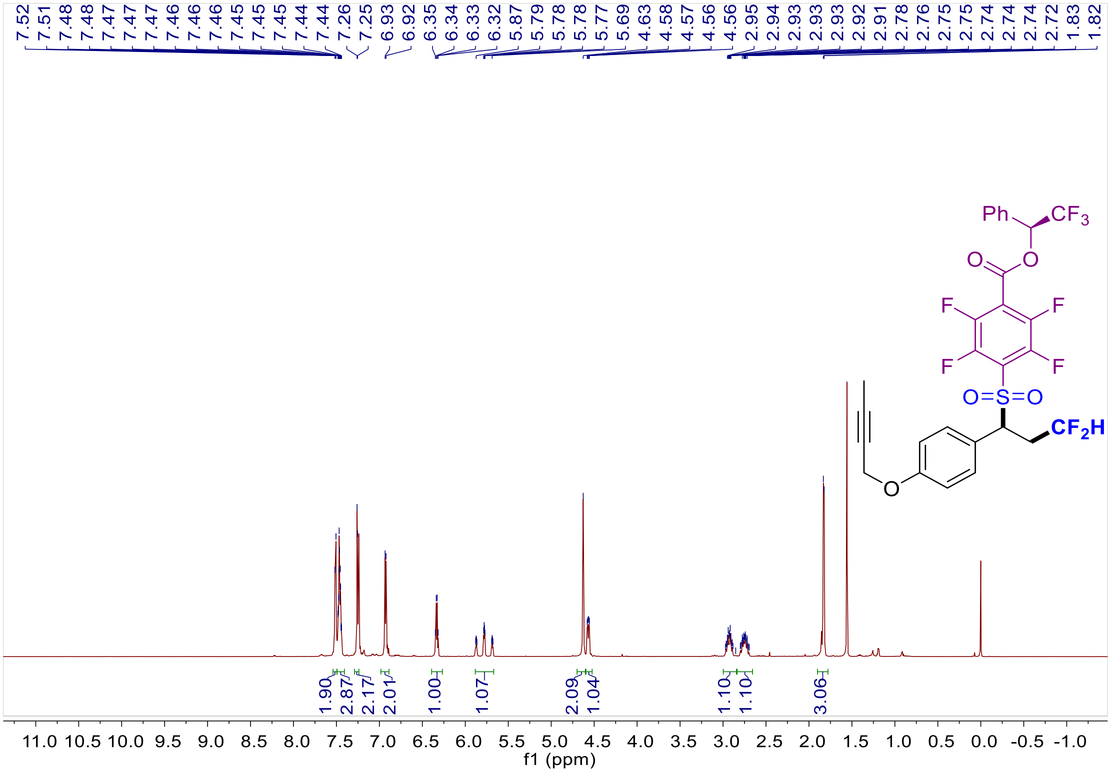

^{13}C NMR (151 MHz, CDCl₃) spectrum of 90

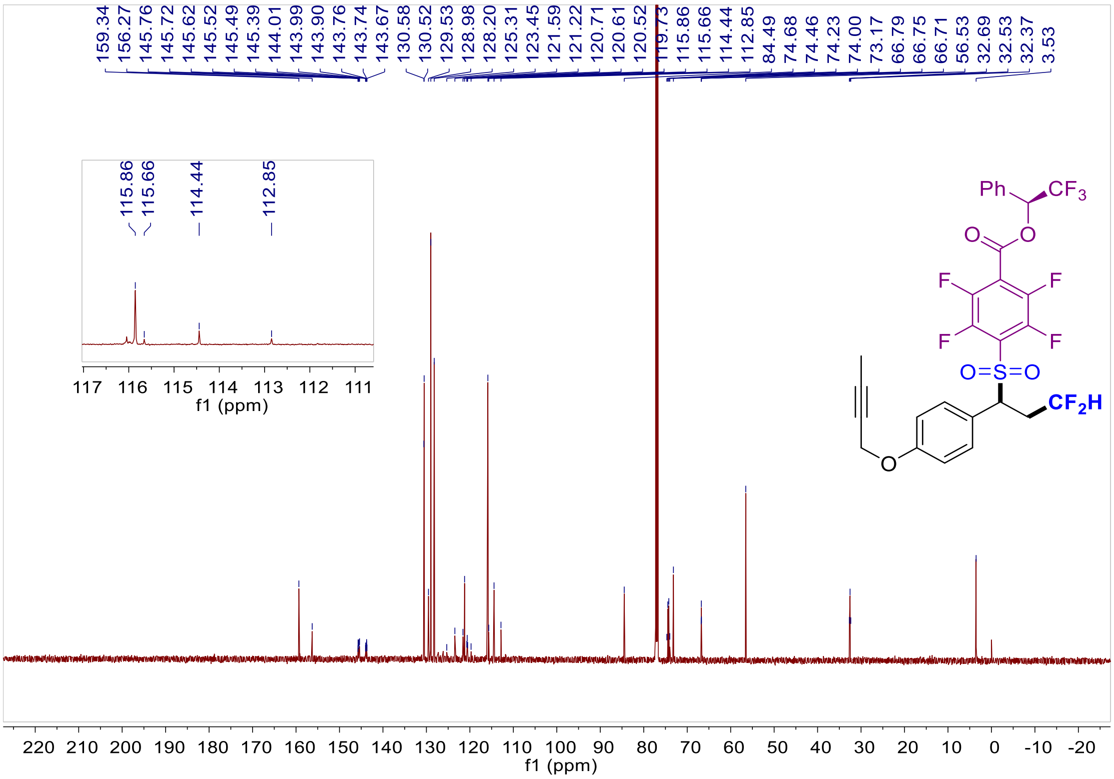

^{19}F NMR (565 MHz, CDCl$_3$) spectrum of 90

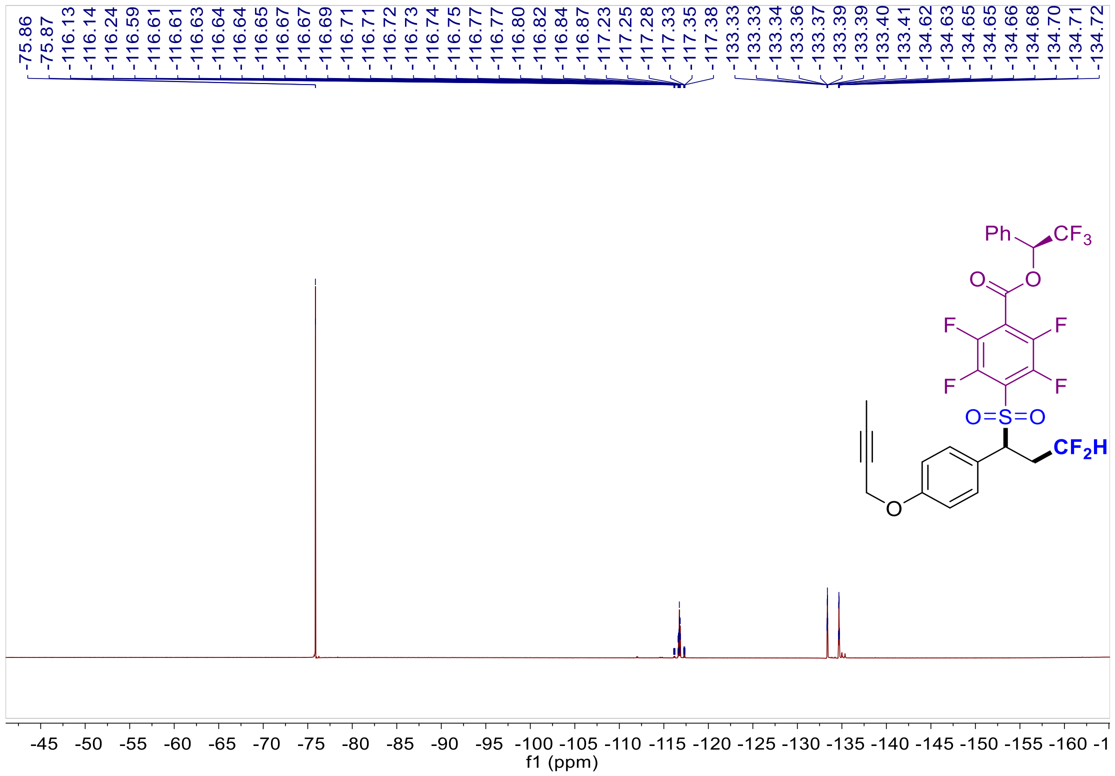

^{1}H NMR (600 MHz, CDCl$_3$) spectrum of 91

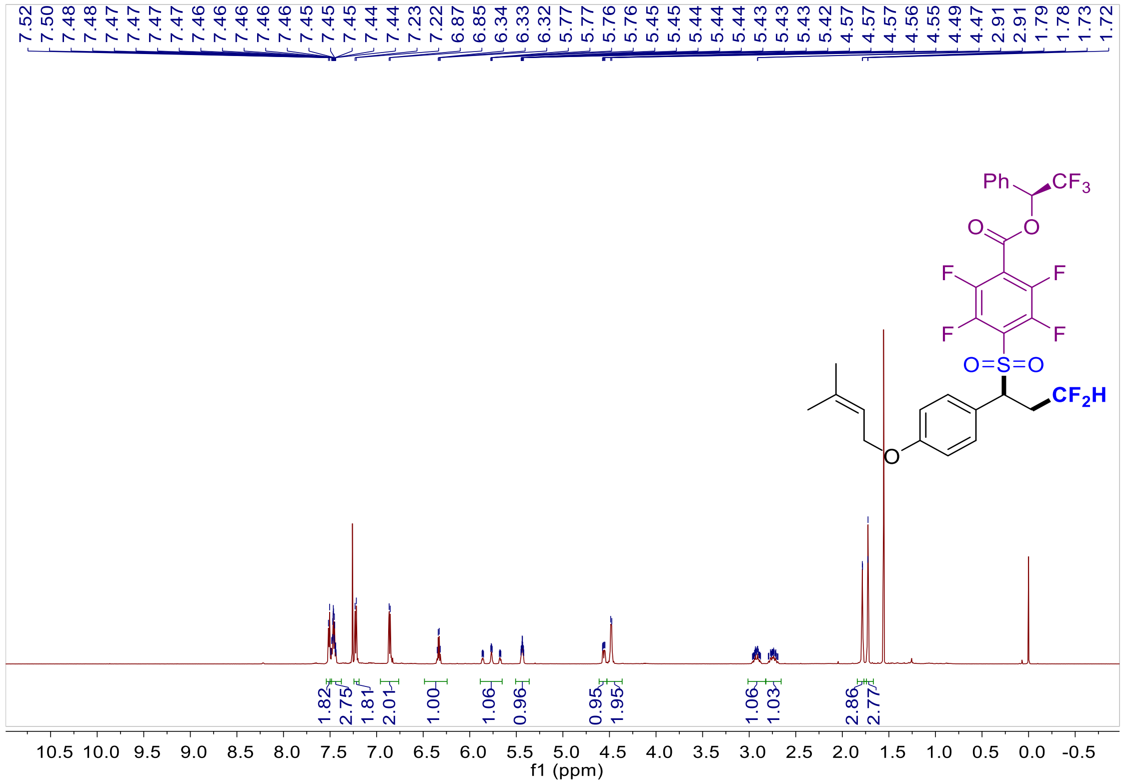

^{13}C NMR (151 MHz, $CDCl_3$) spectrum of 91

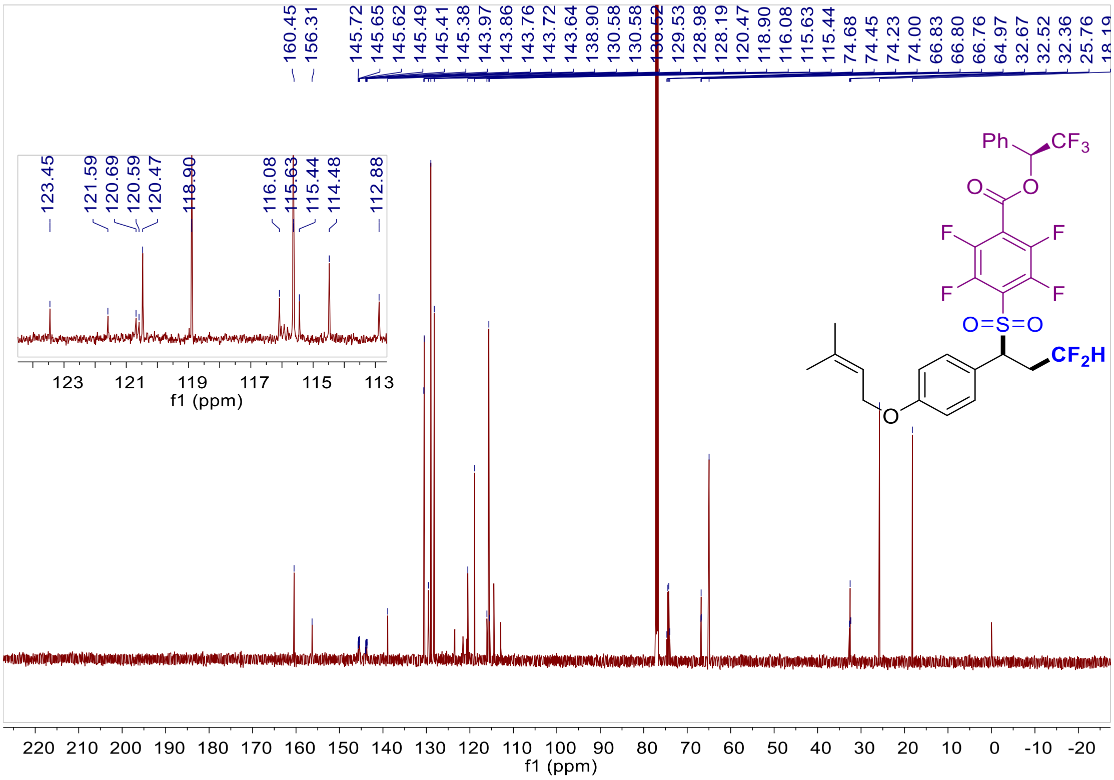

^{19}F NMR (565 MHz, $CDCl_3$) spectrum of 91

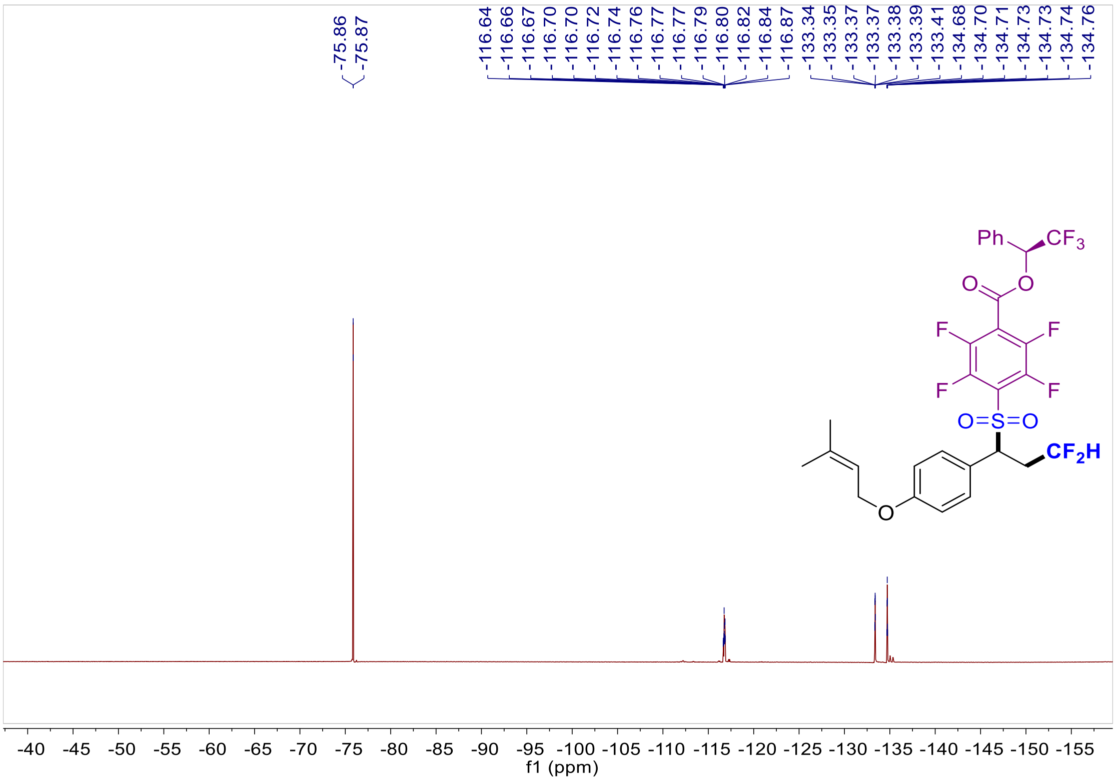

^{1}H NMR (600 MHz, $CDCl_3$) spectrum of 92

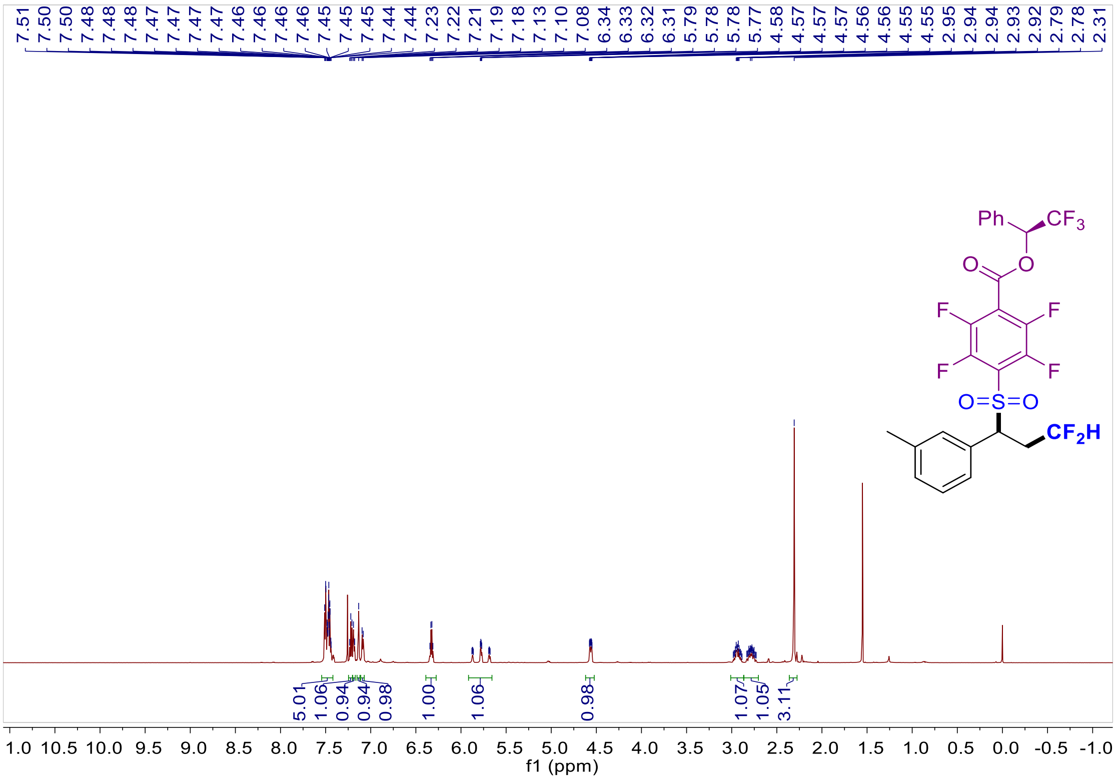

^{13}C NMR (151 MHz, $CDCl_3$) spectrum of 92

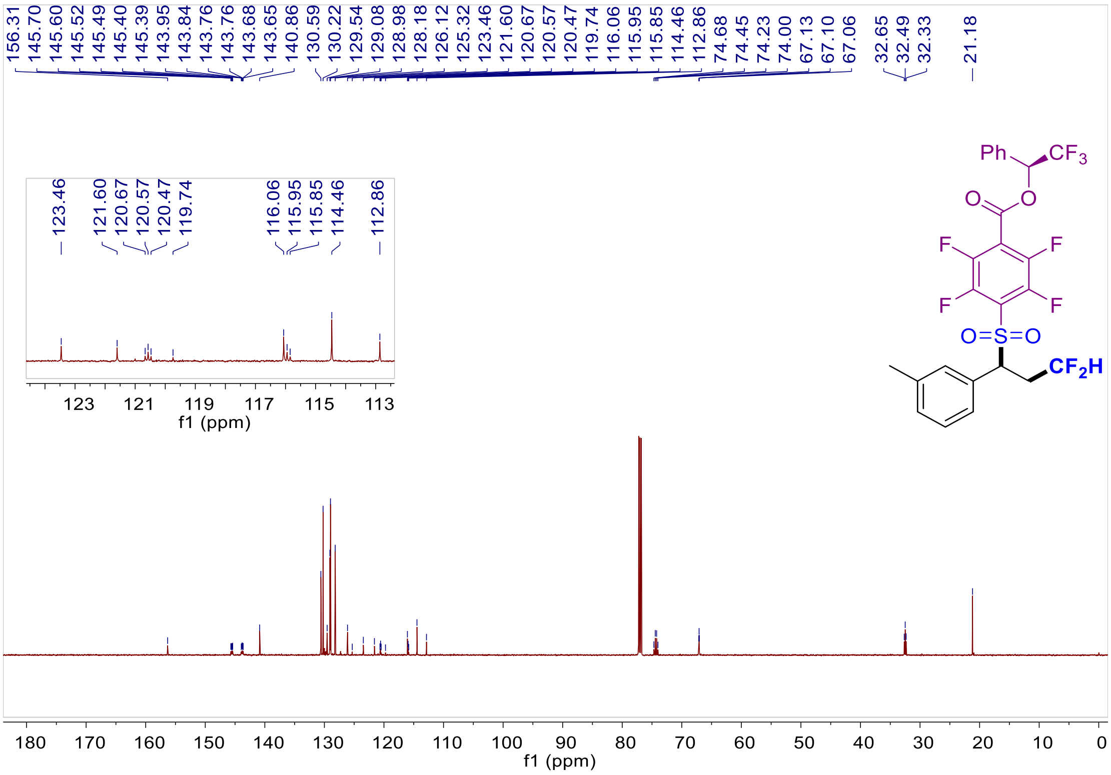

^{19}F NMR (565 MHz, CDCl3) spectrum of 92

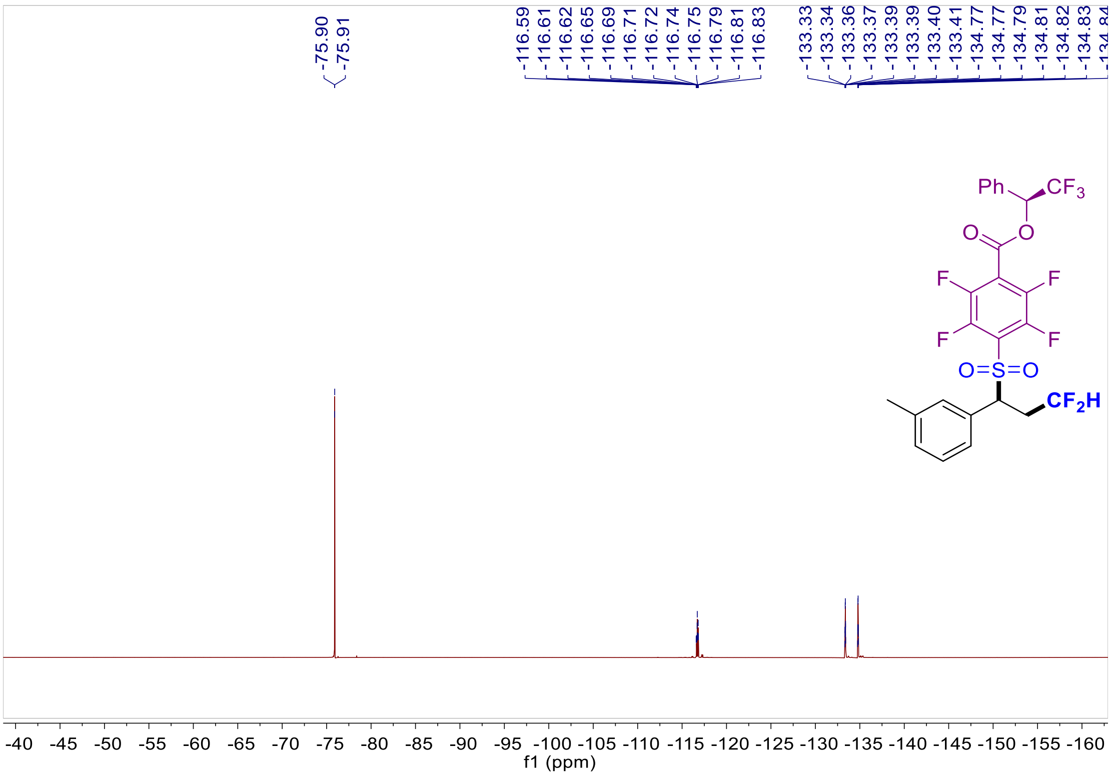

^{1}H NMR (600 MHz, CDCl3) spectrum of 93

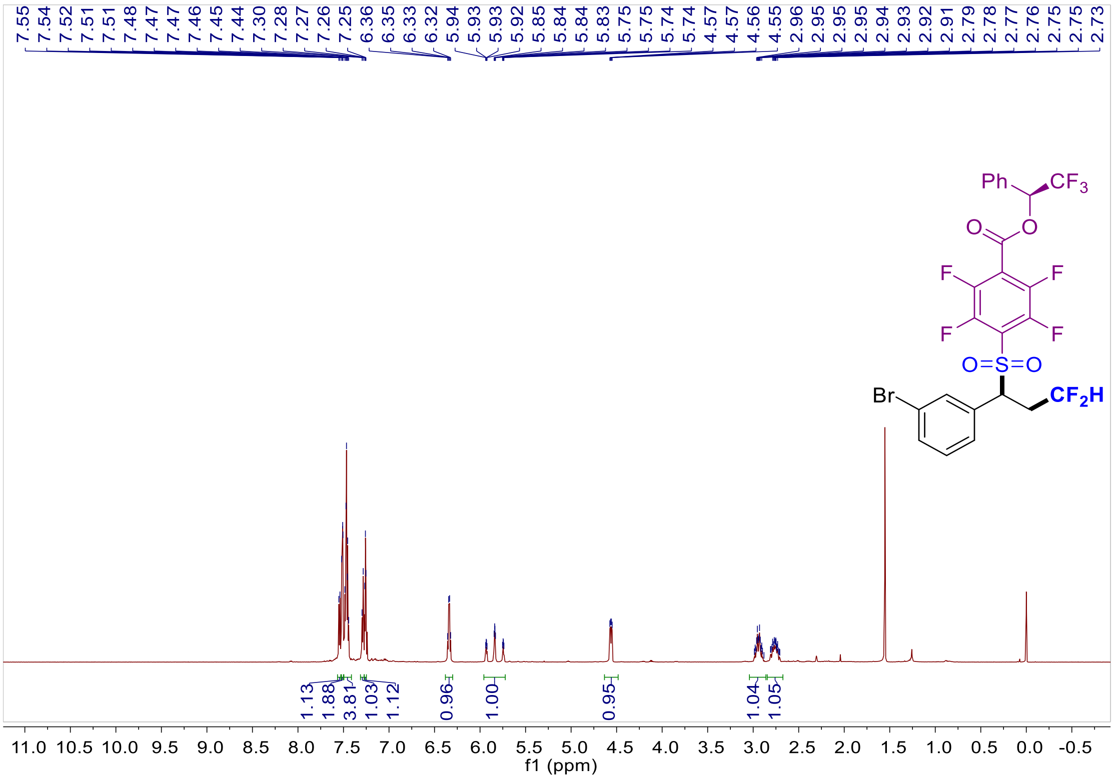

^{13}C NMR (151 MHz, CDCl$_3$) spectrum of 93

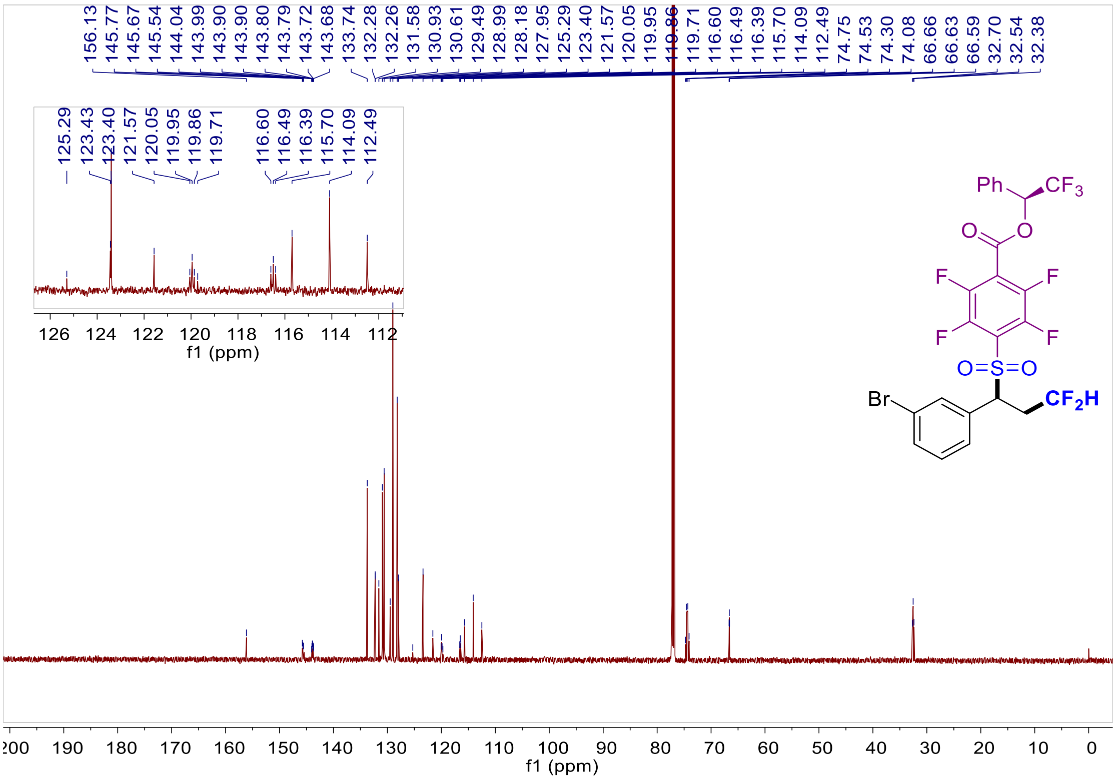

^{19}F NMR (565 MHz, CDCl$_3$) spectrum of 93

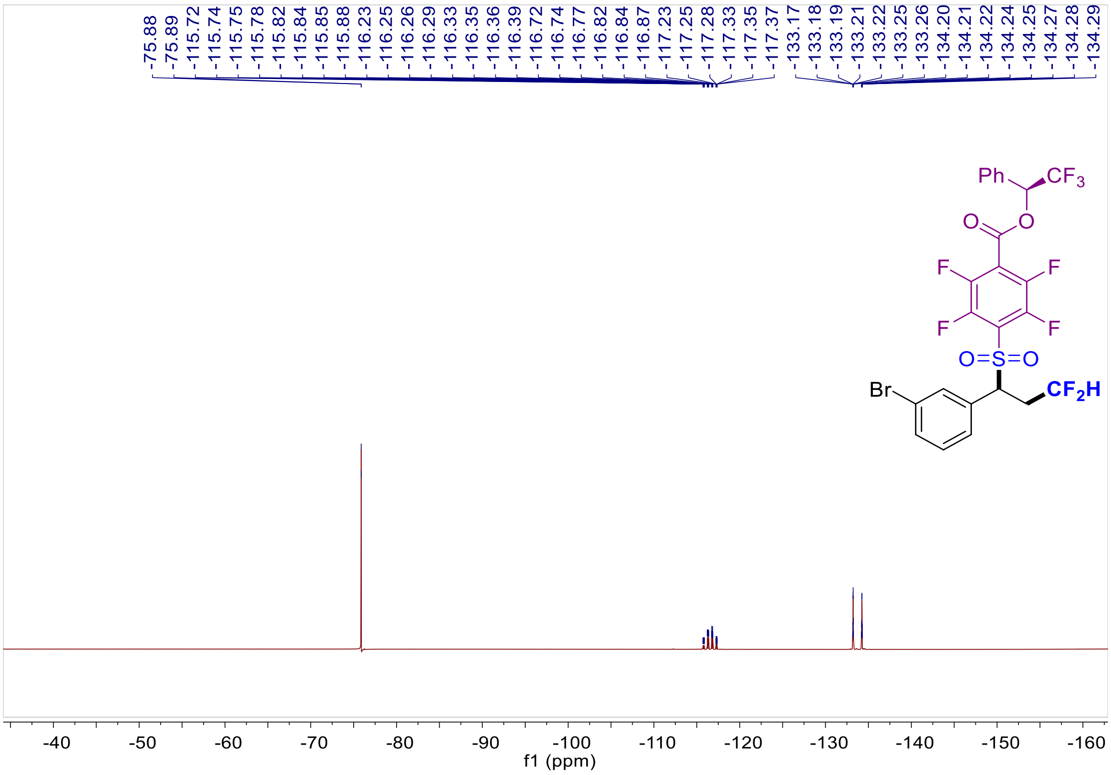

^{1}H NMR (600 MHz, $CDCl_3$) spectrum of 94

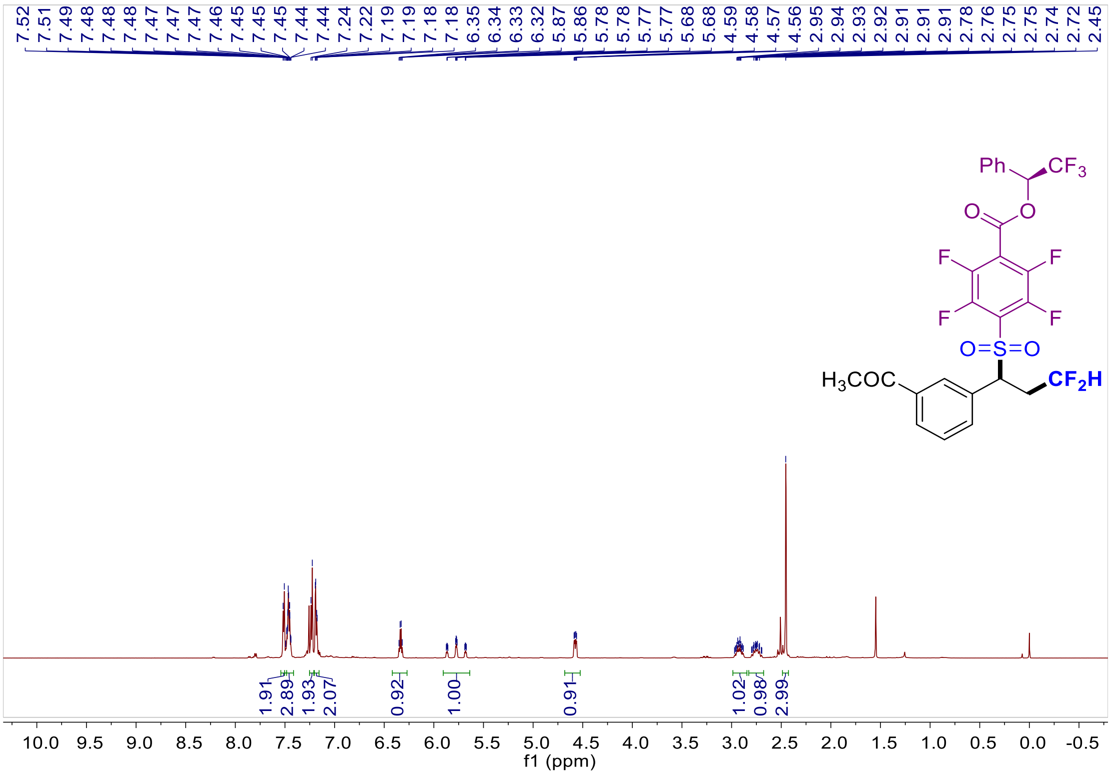

^{13}C NMR (151 MHz, $CDCl_3$) spectrum of 94

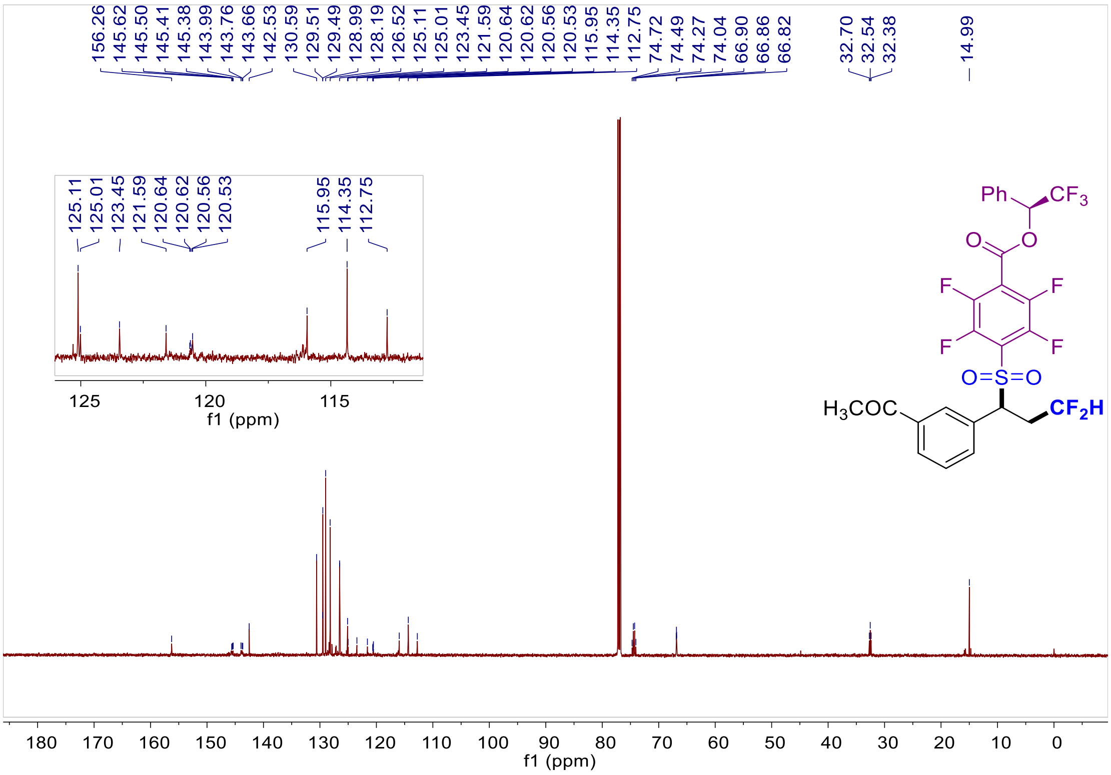

^{19}F NMR (565 MHz, CDCl3) spectrum of 94

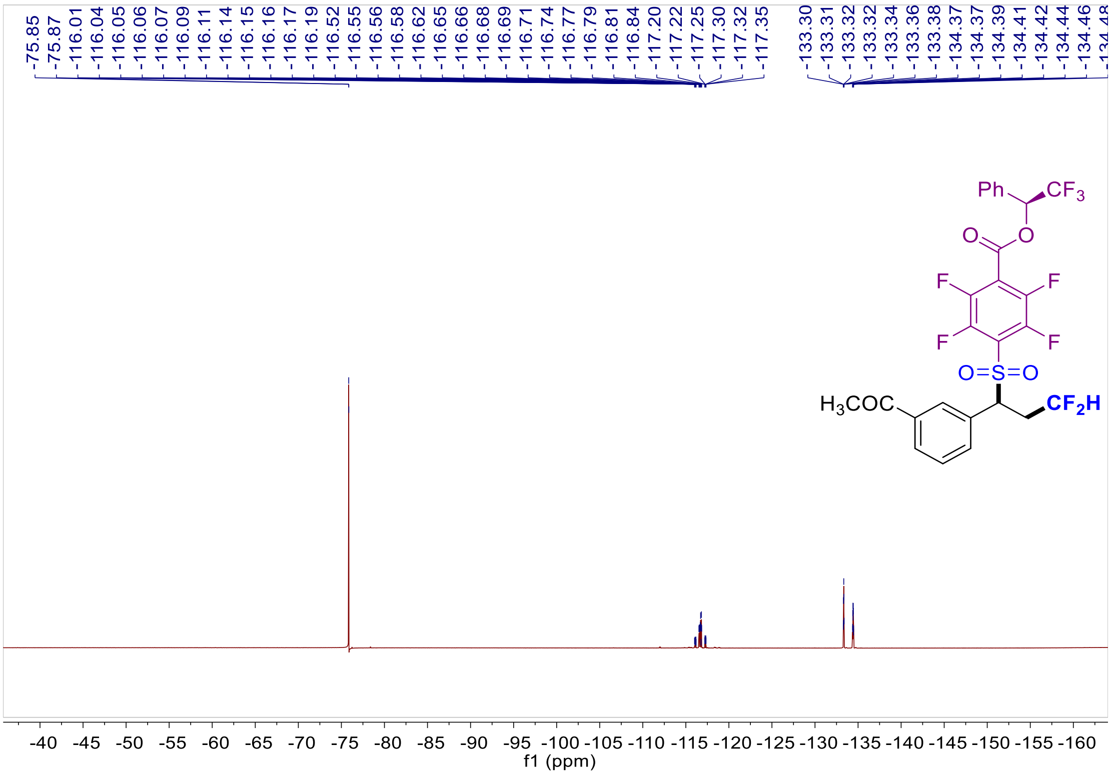

^{1}H NMR (600 MHz, CDCl3) spectrum of 95

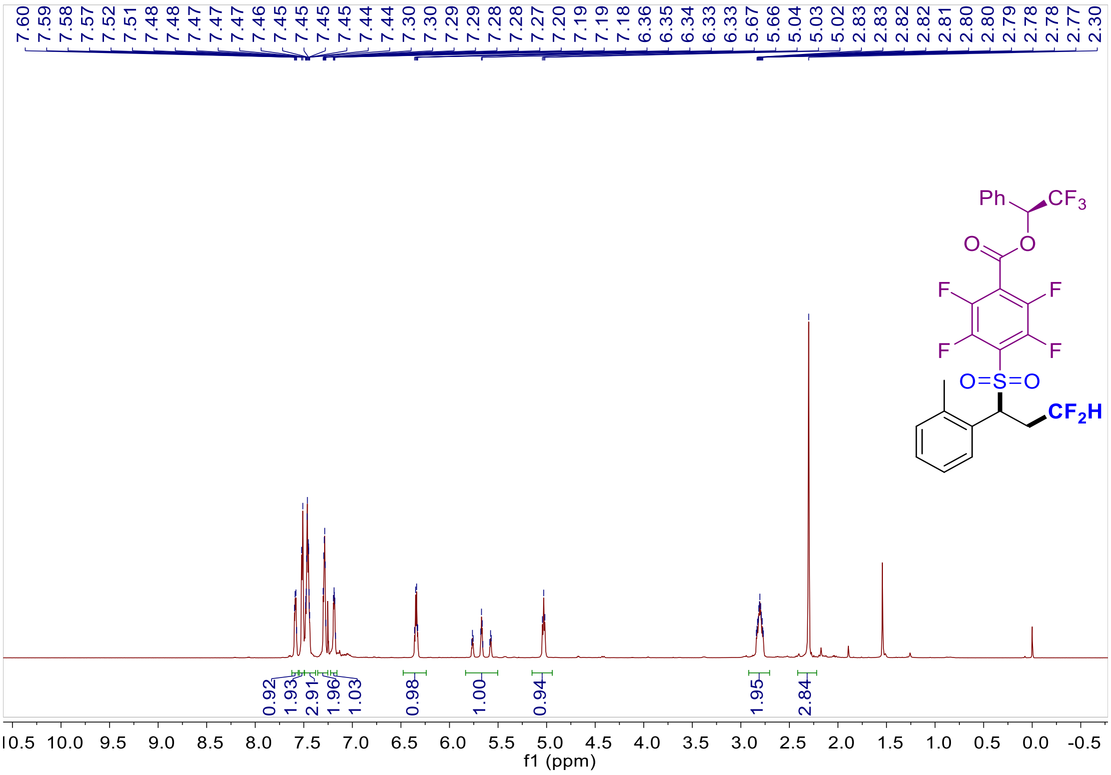

^{13}C NMR (151 MHz, $CDCl_3$) spectrum of 95

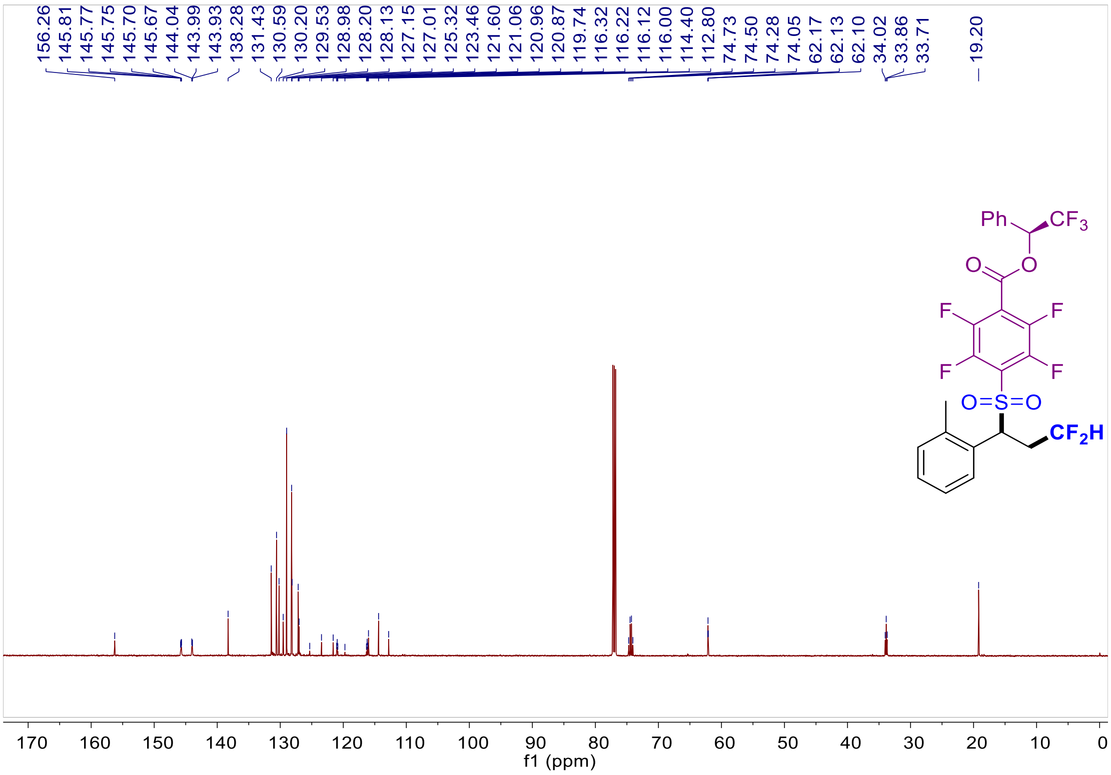

^{19}F NMR (565 MHz, $CDCl_3$) spectrum of 95

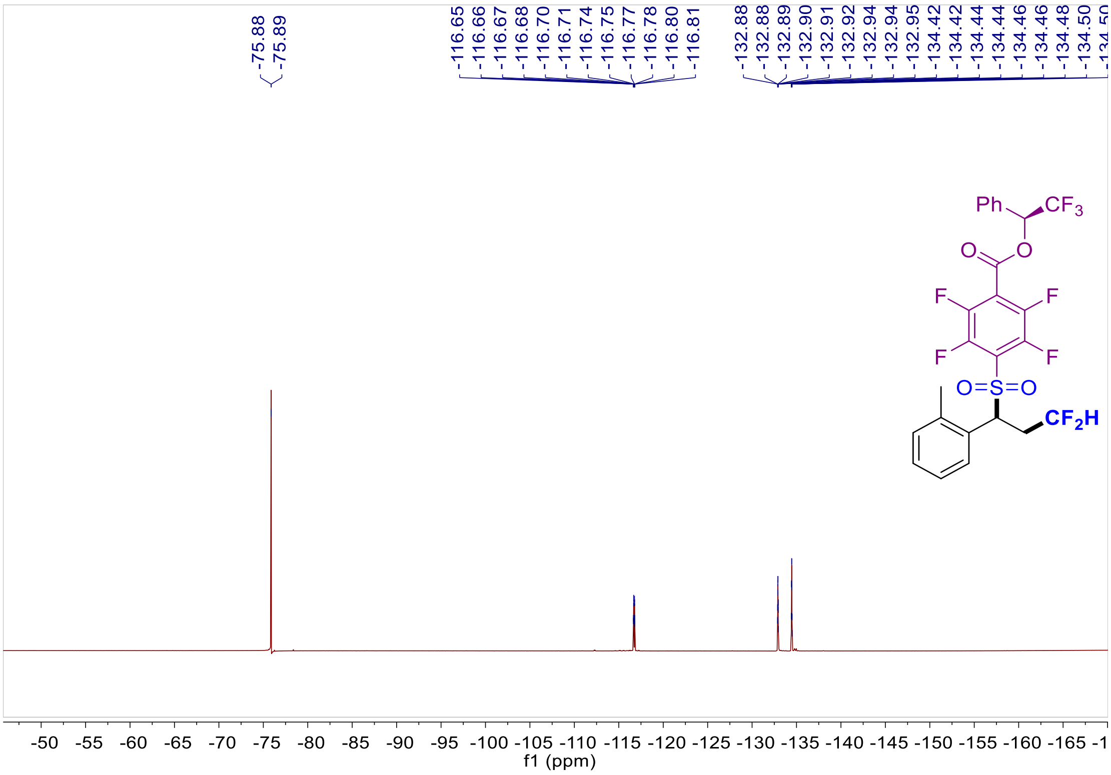

^{1}H NMR (600 MHz, CDCl3) spectrum of 96

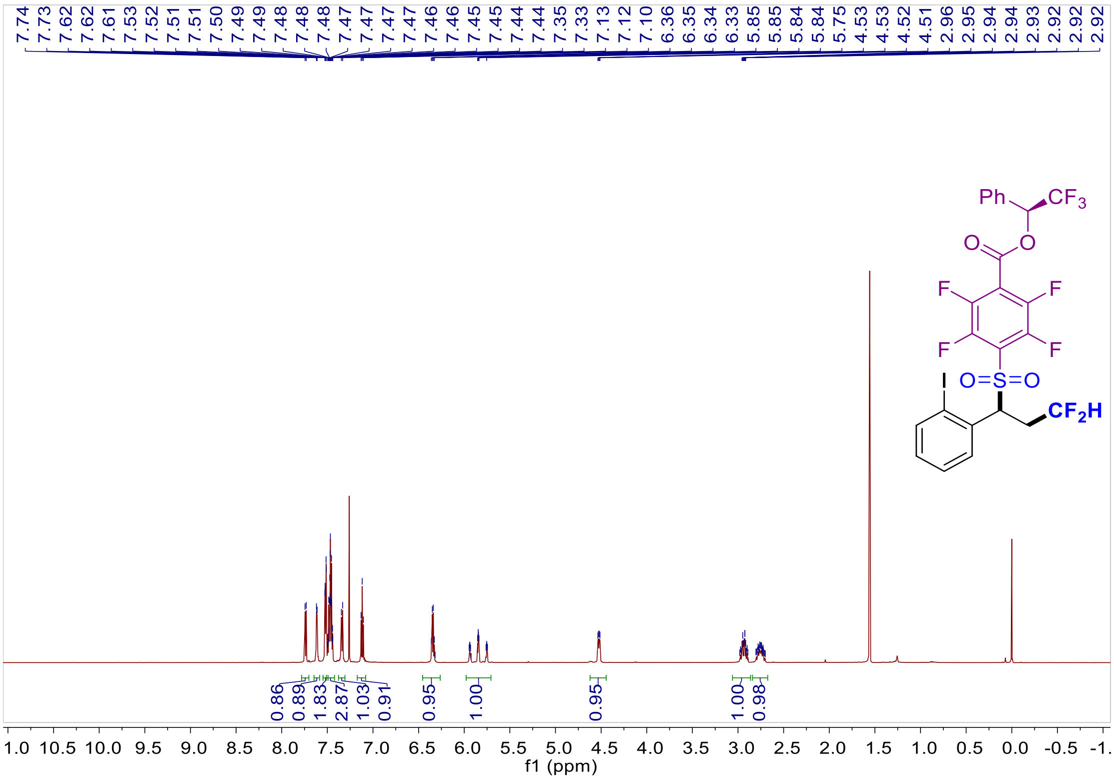

^{13}C NMR (151 MHz, CDCl3) spectrum of 96

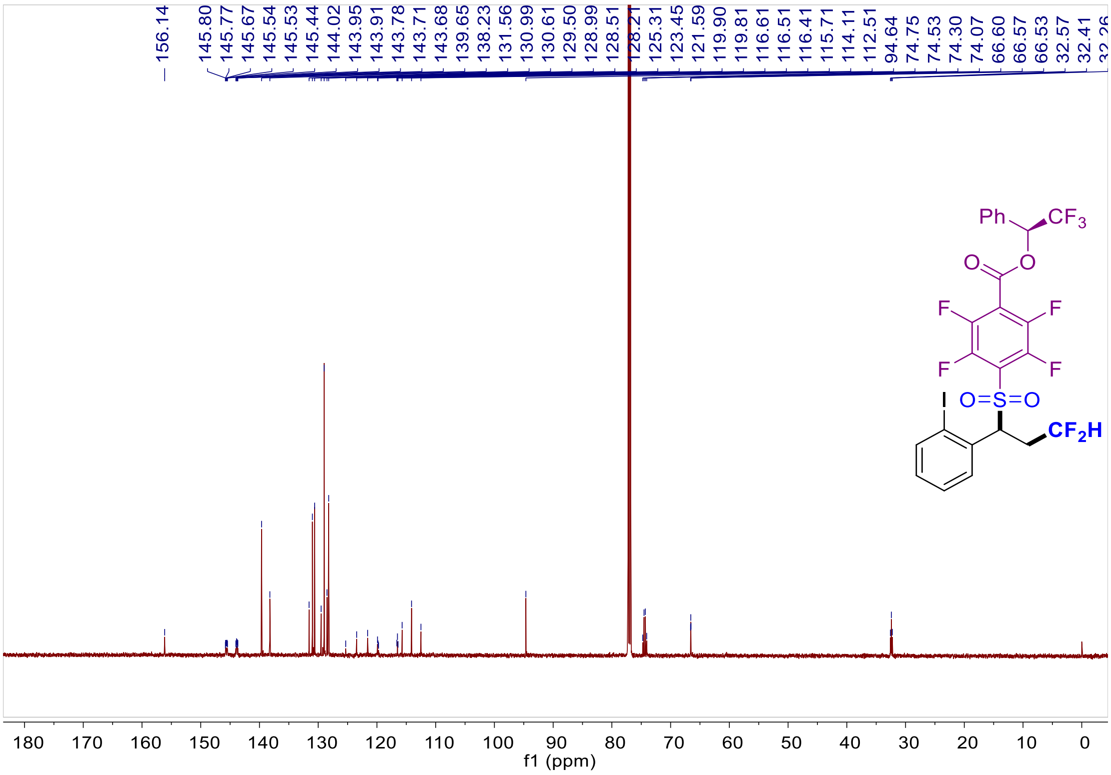

^{19}F NMR (565 MHz, $CDCl_3$) spectrum of 96

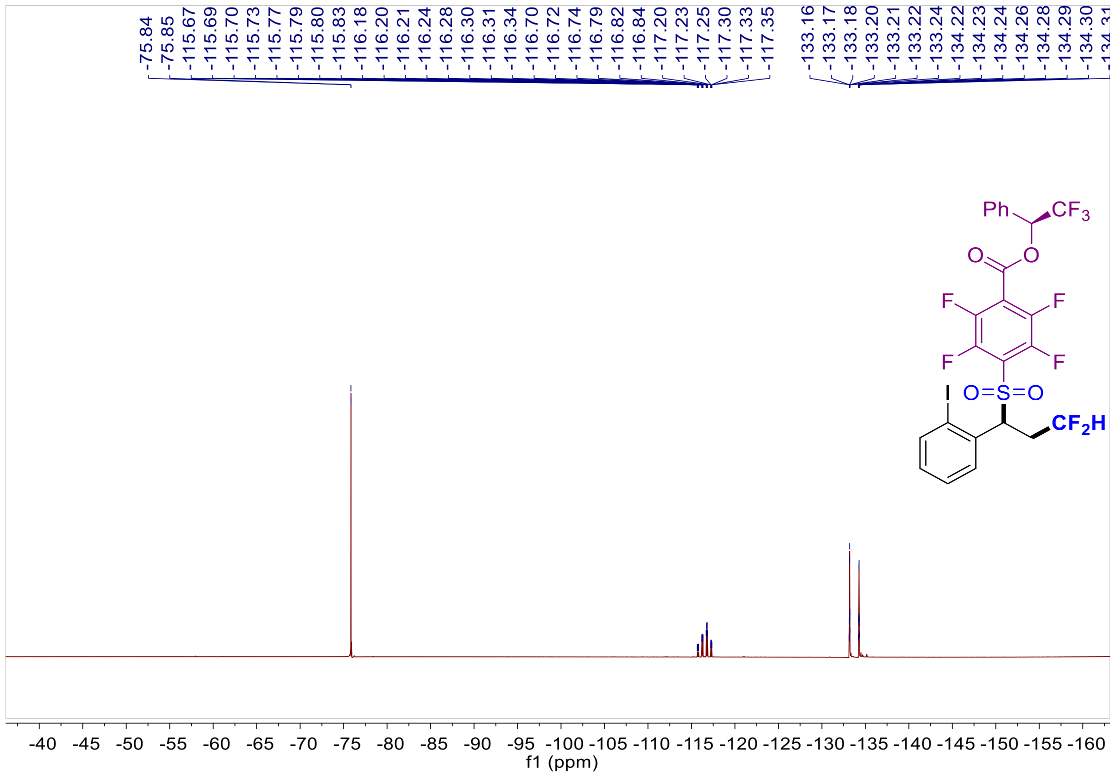

^{1}H NMR (600 MHz, $CDCl_3$) spectrum of 97

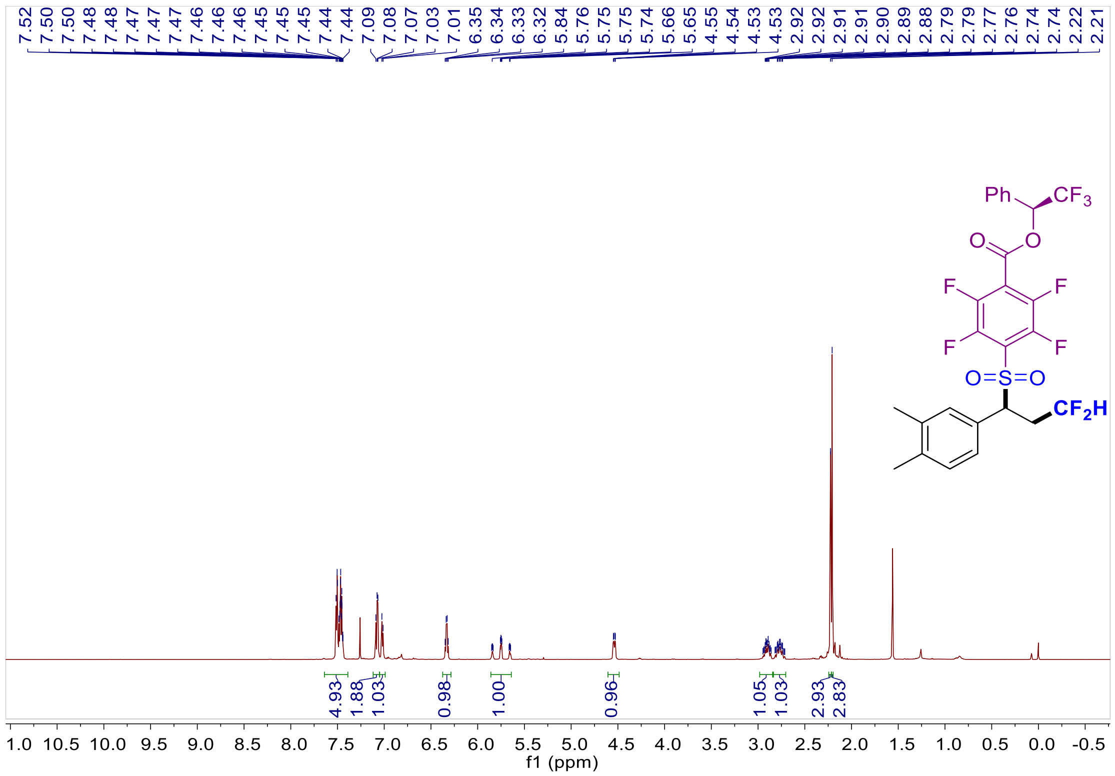

^{13}C NMR (151 MHz, $CDCl_3$) spectrum of 97

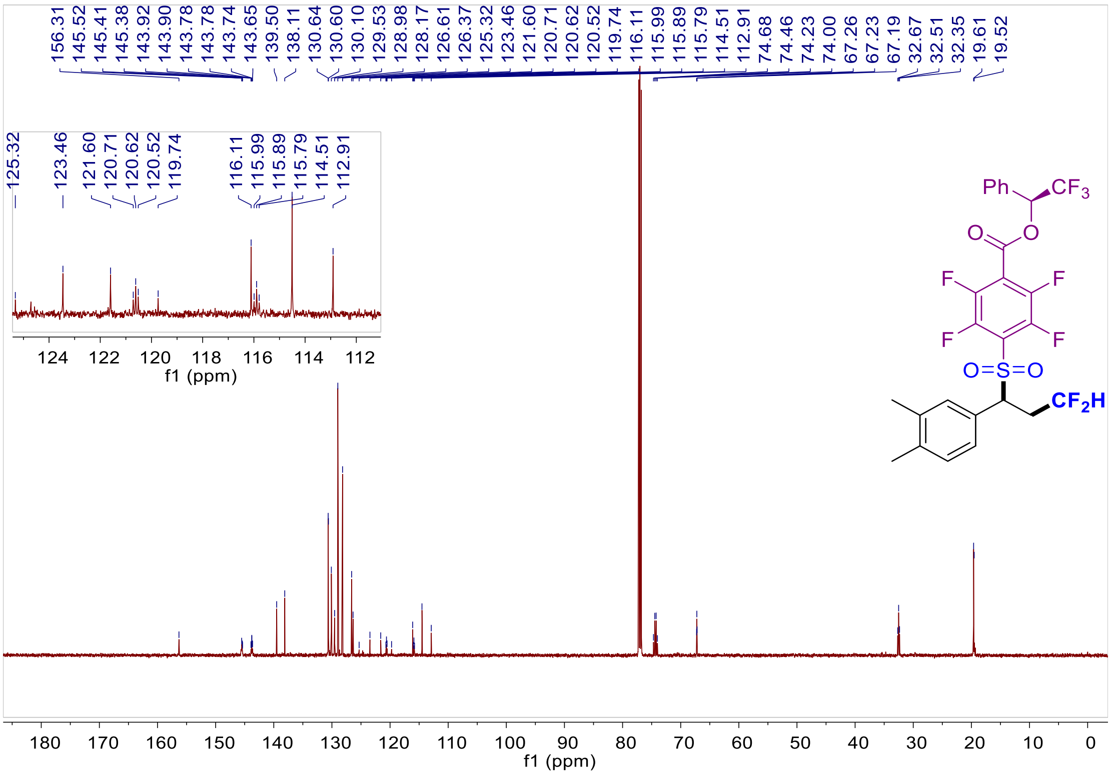

^{19}F NMR (565 MHz, $CDCl_3$) spectrum of 97

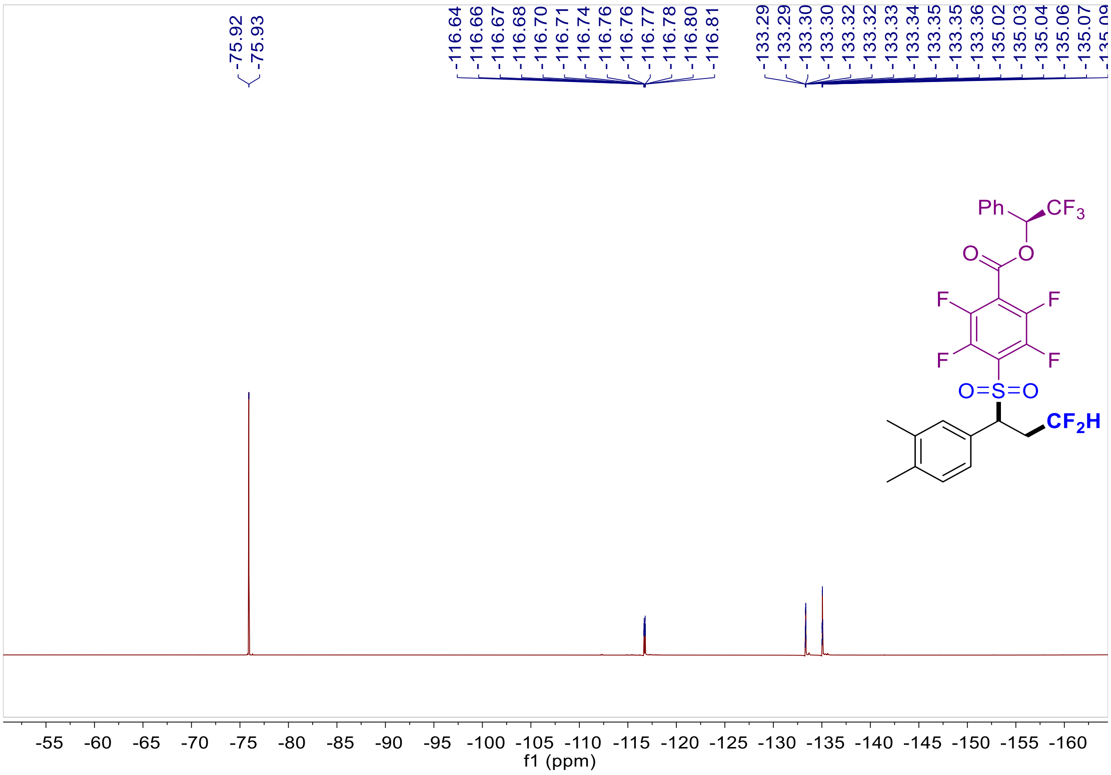

^{1}H NMR (600 MHz, CDCl$_3$) spectrum of 98

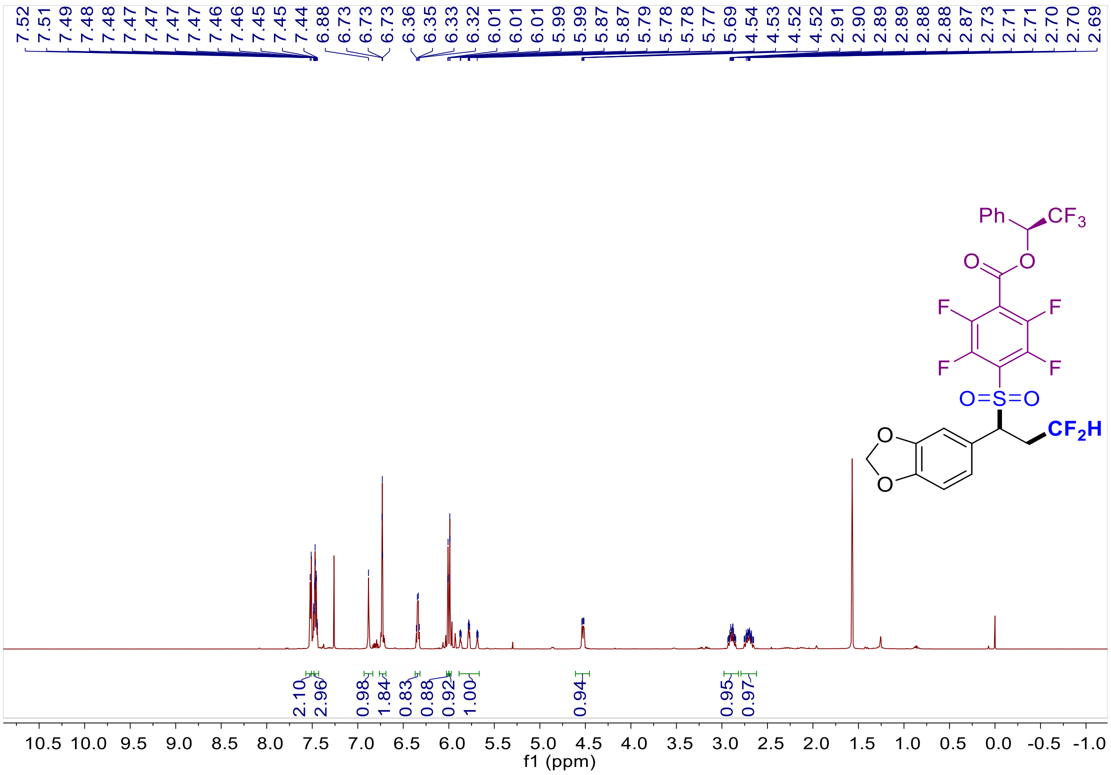

^{13}C NMR (151 MHz, CDCl$_3$) spectrum of 98

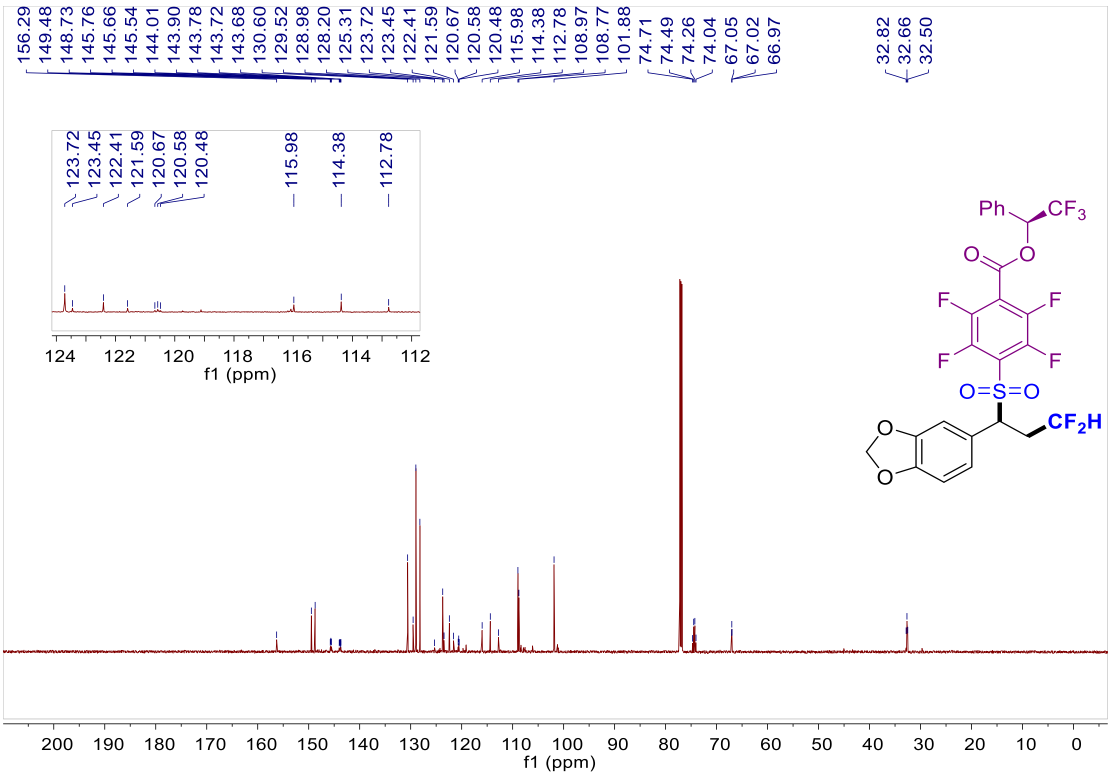

^{19}F NMR (565 MHz, CDCl$_3$) spectrum of 98

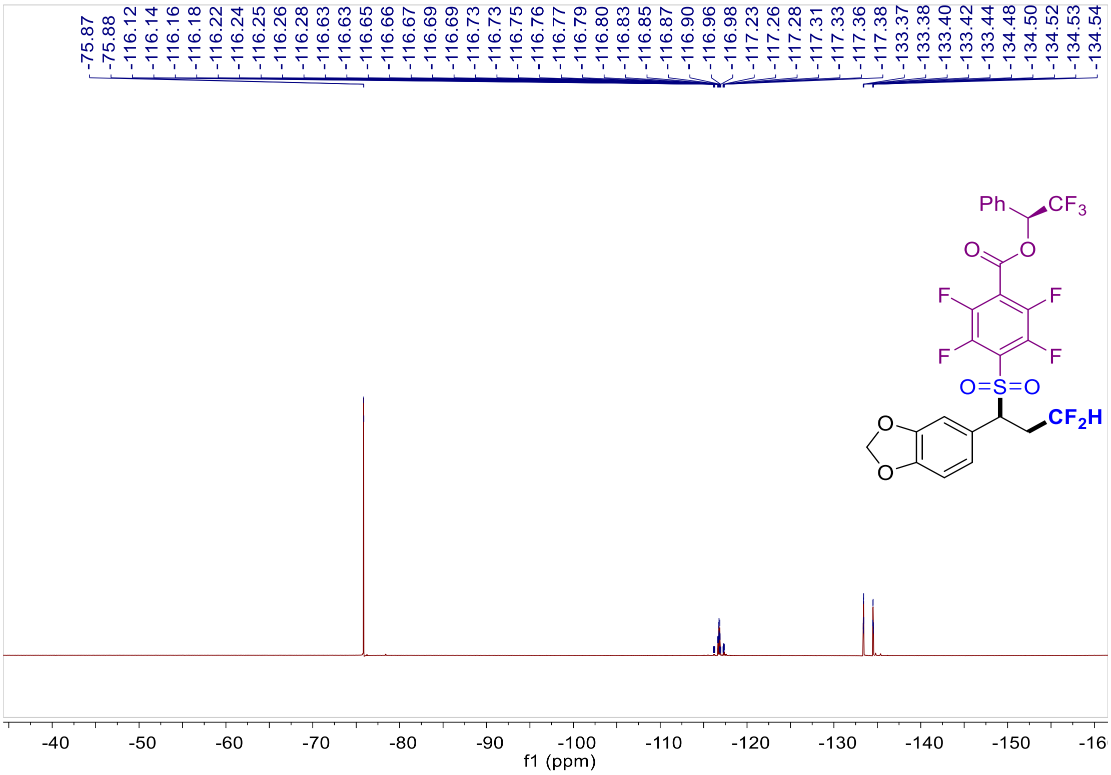

^{1}H NMR (600 MHz, CDCl$_3$) spectrum of 99

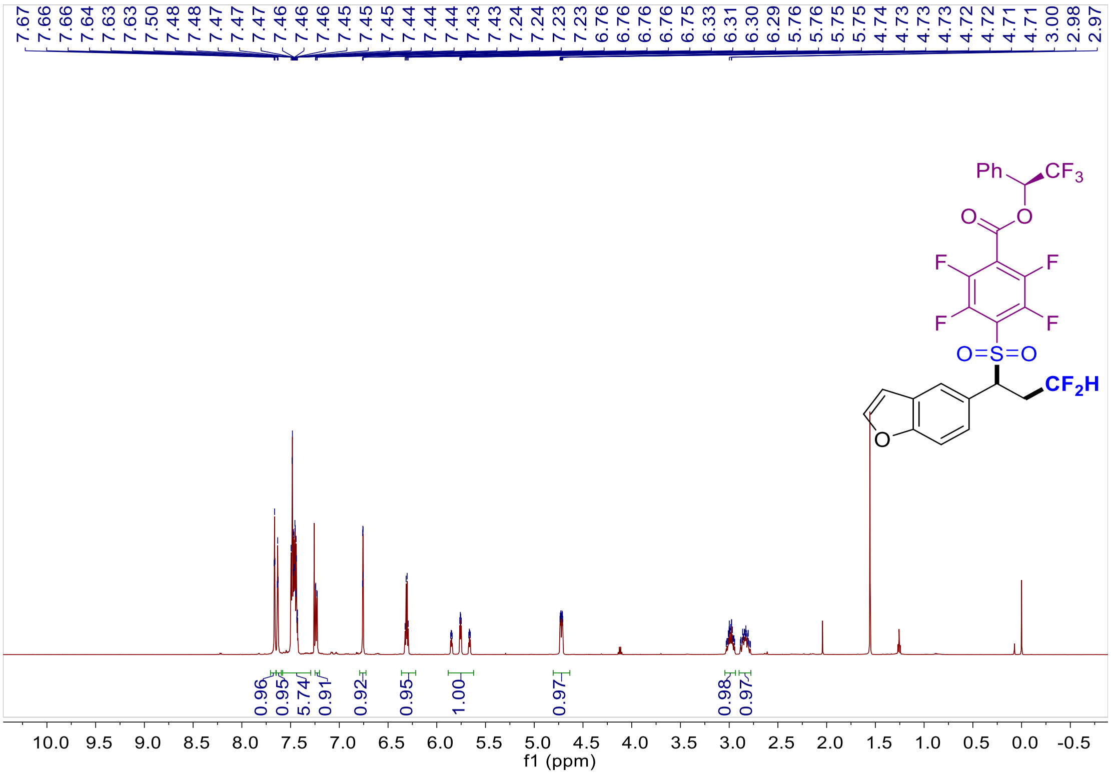

^{13}C NMR (151 MHz, CDCl$_3$) spectrum of 99

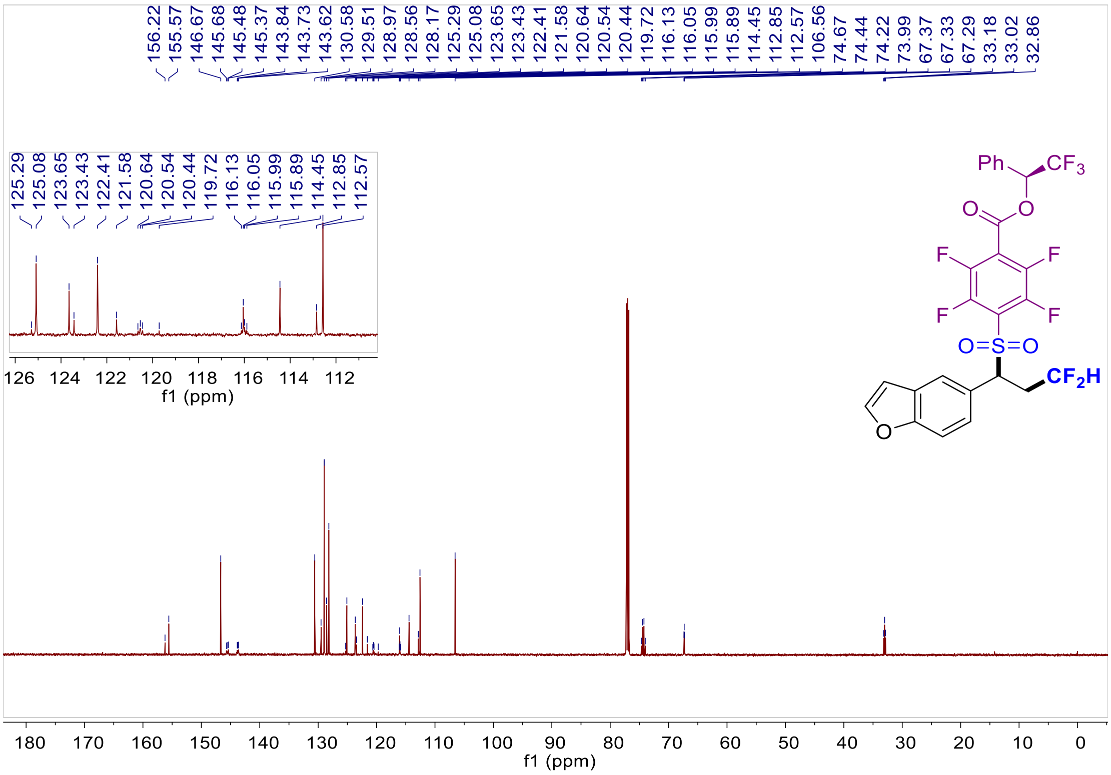

^{19}F NMR (565 MHz, CDCl$_3$) spectrum of 99

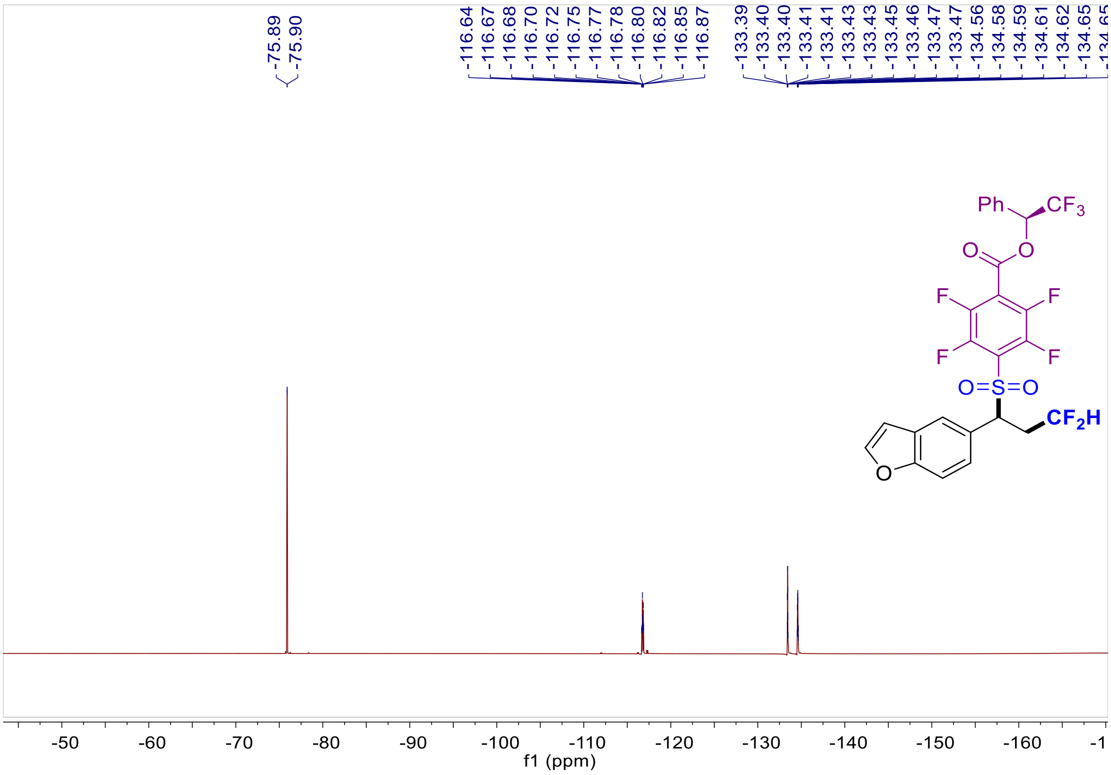

^{1}H NMR (600 MHz, CDCl$_3$) spectrum of 100

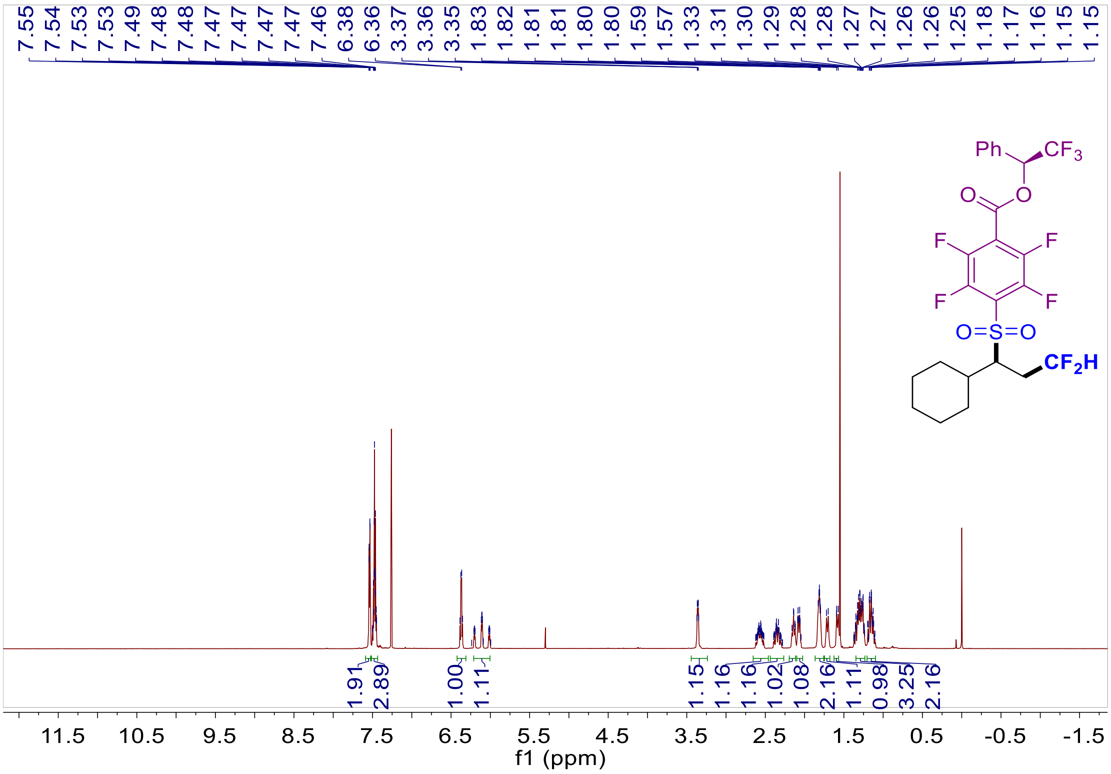

^{13}C NMR (151 MHz, CDCl$_3$) spectrum of 100

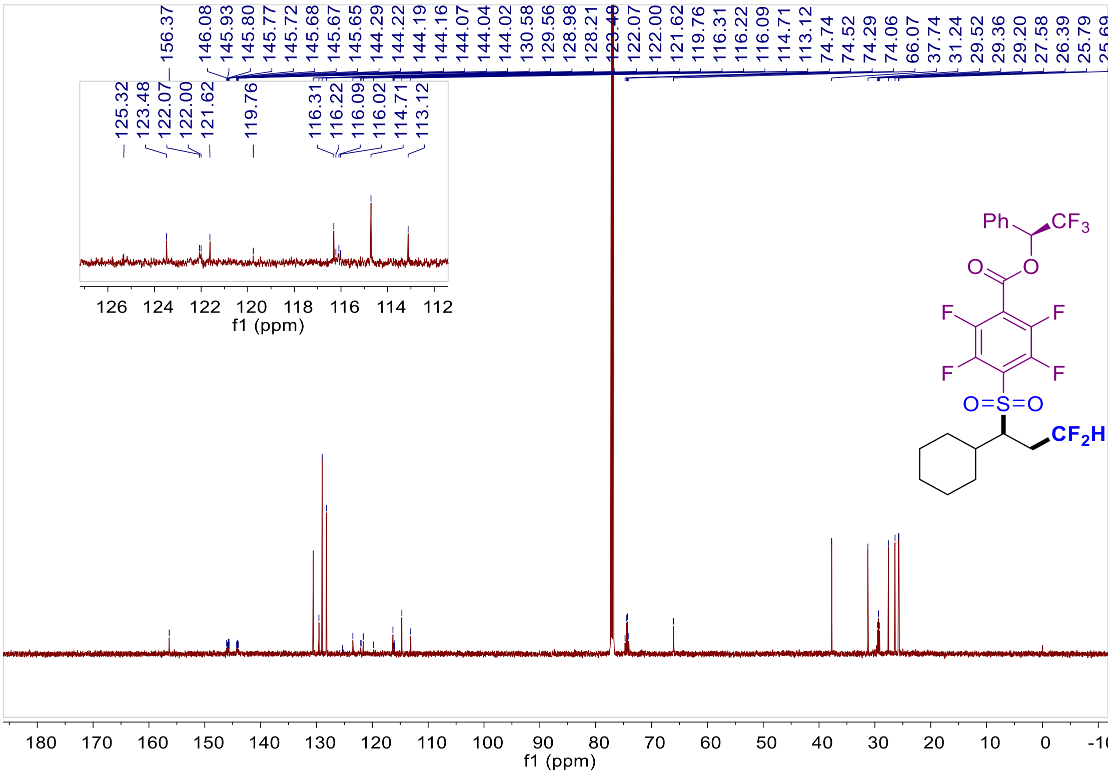

^{19}F NMR (565 MHz, CDCl$_3$) spectrum of 100

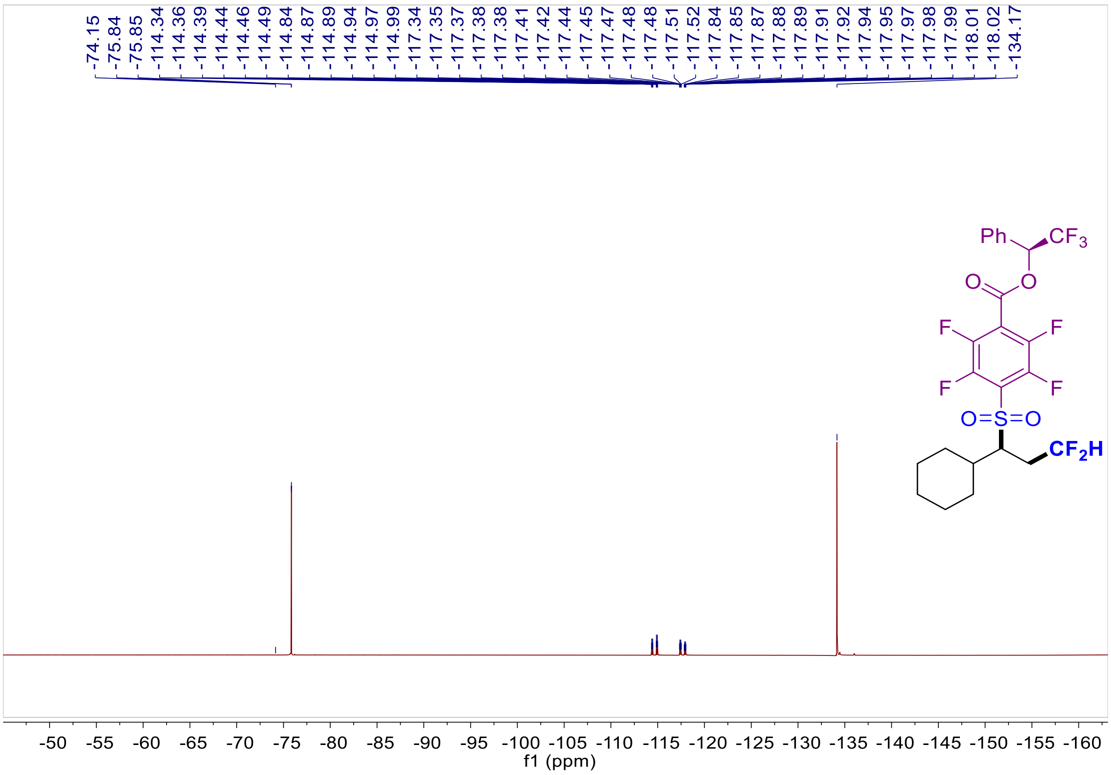

^{1}H NMR (600 MHz, CDCl$_3$) spectrum of 101

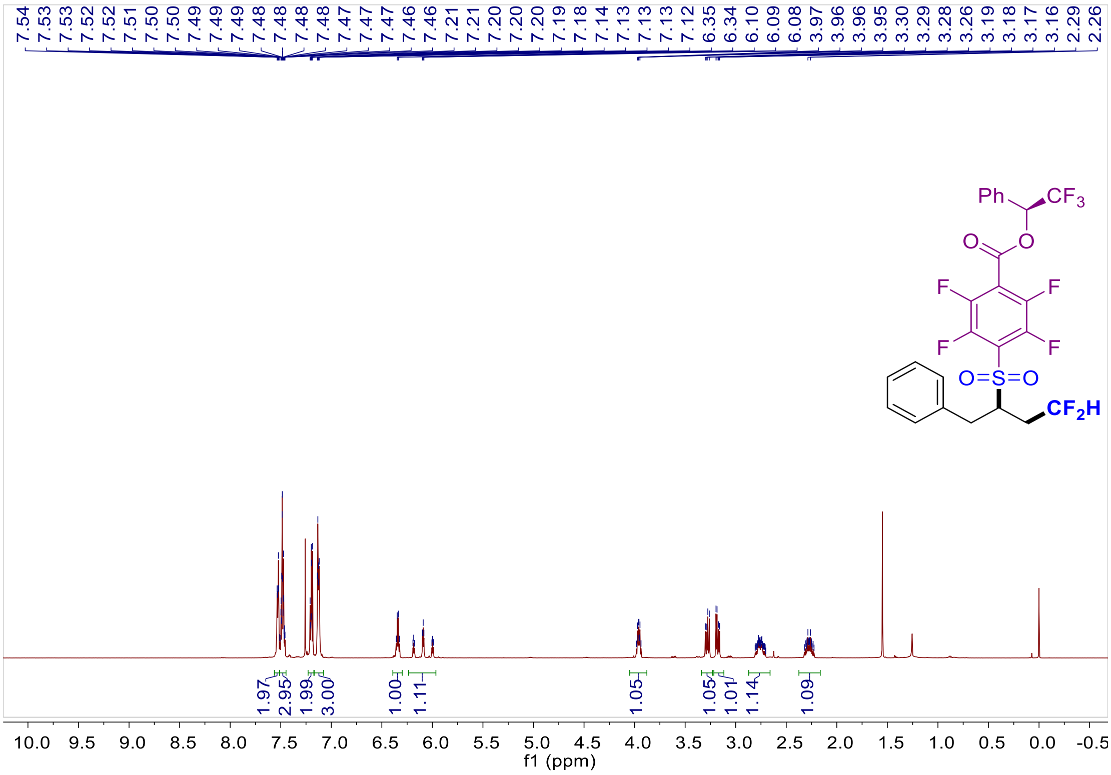

^{13}C NMR (151 MHz, $CDCl_3$) spectrum of 101

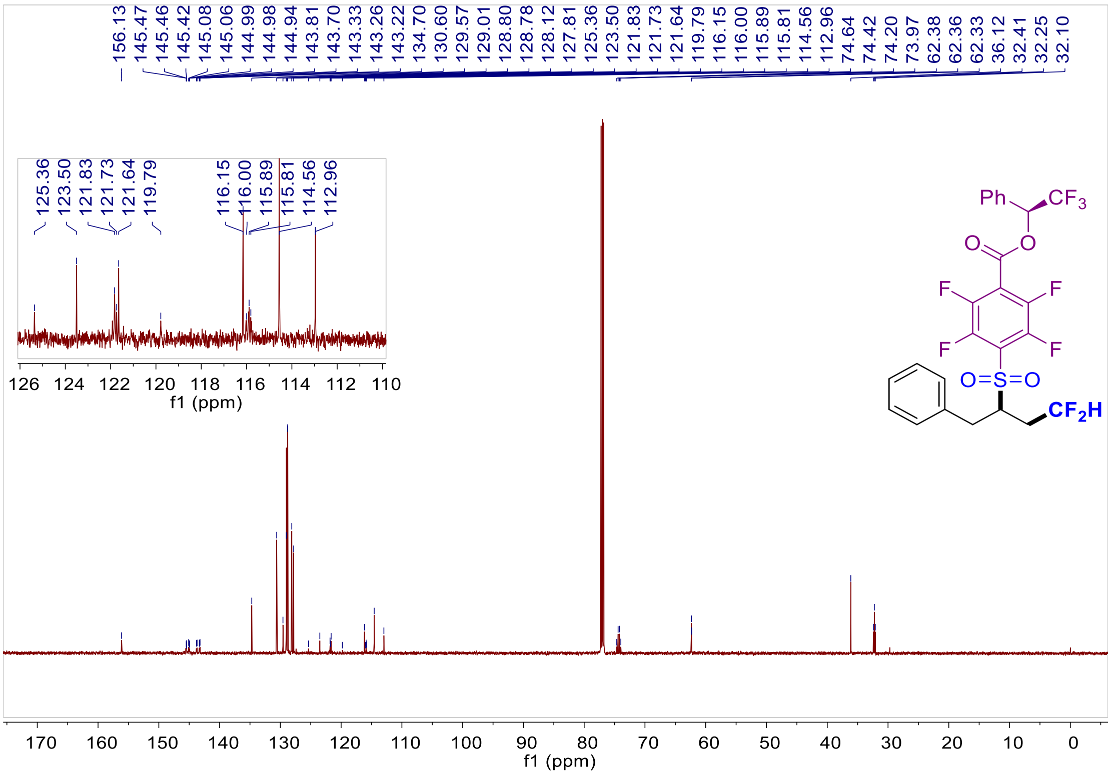

^{19}F NMR (565 MHz, $CDCl_3$) spectrum of 101

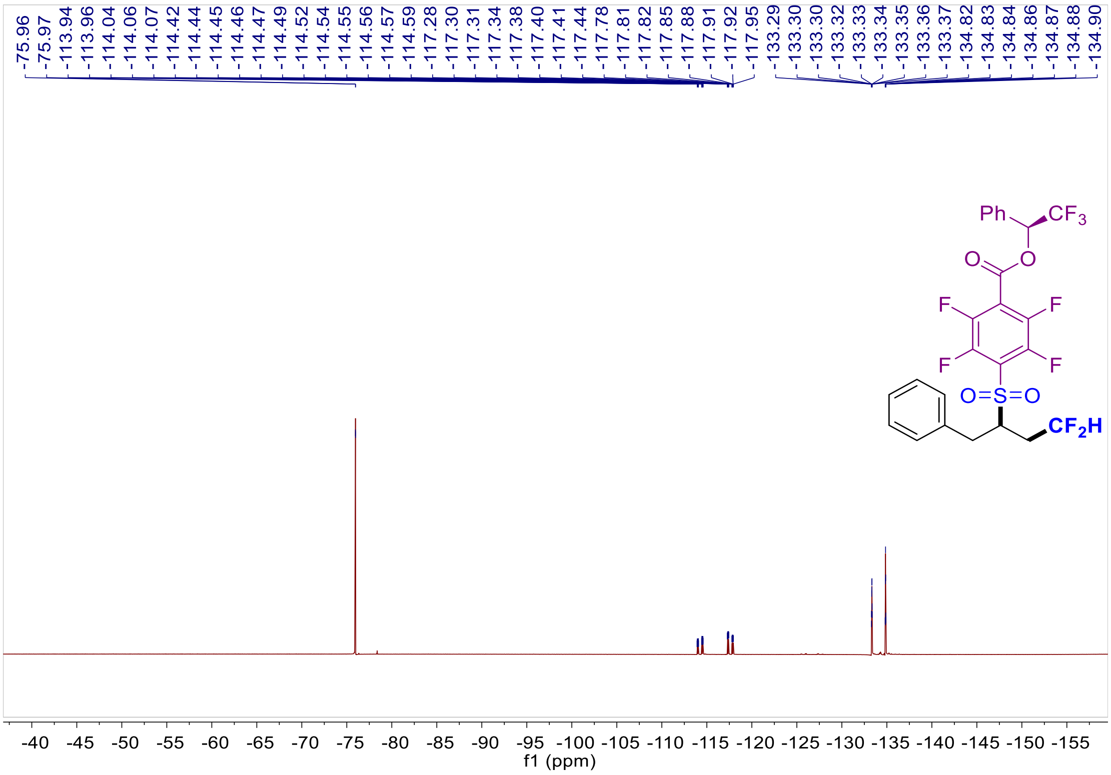

^{1}H NMR (600 MHz, $CDCl_3$) spectrum of 102

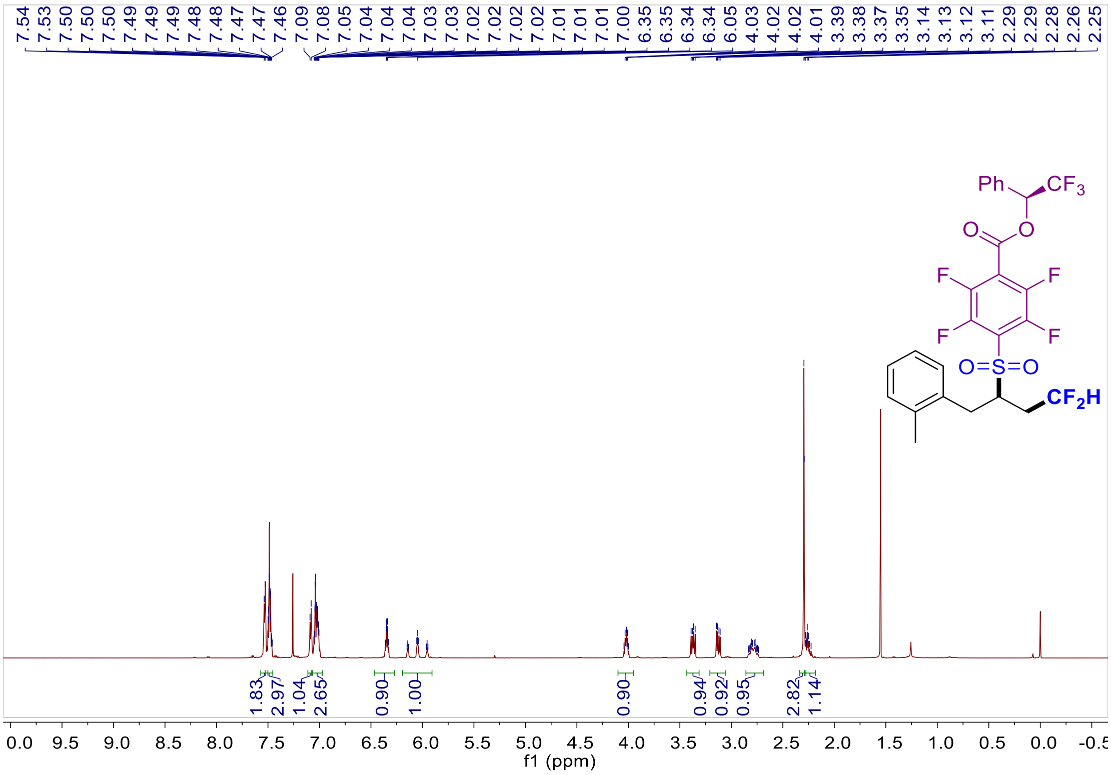

^{13}C NMR (151 MHz, $CDCl_3$) spectrum of 102

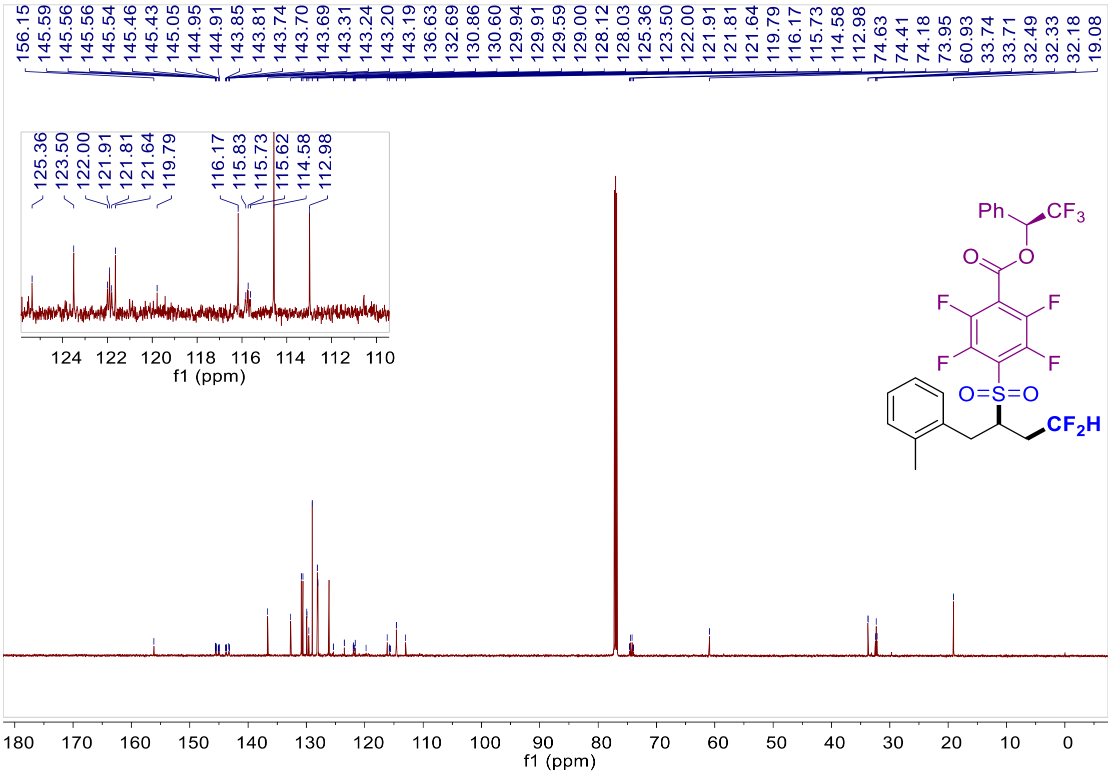

^{19}F NMR (565 MHz, CDCl₃) spectrum of 102

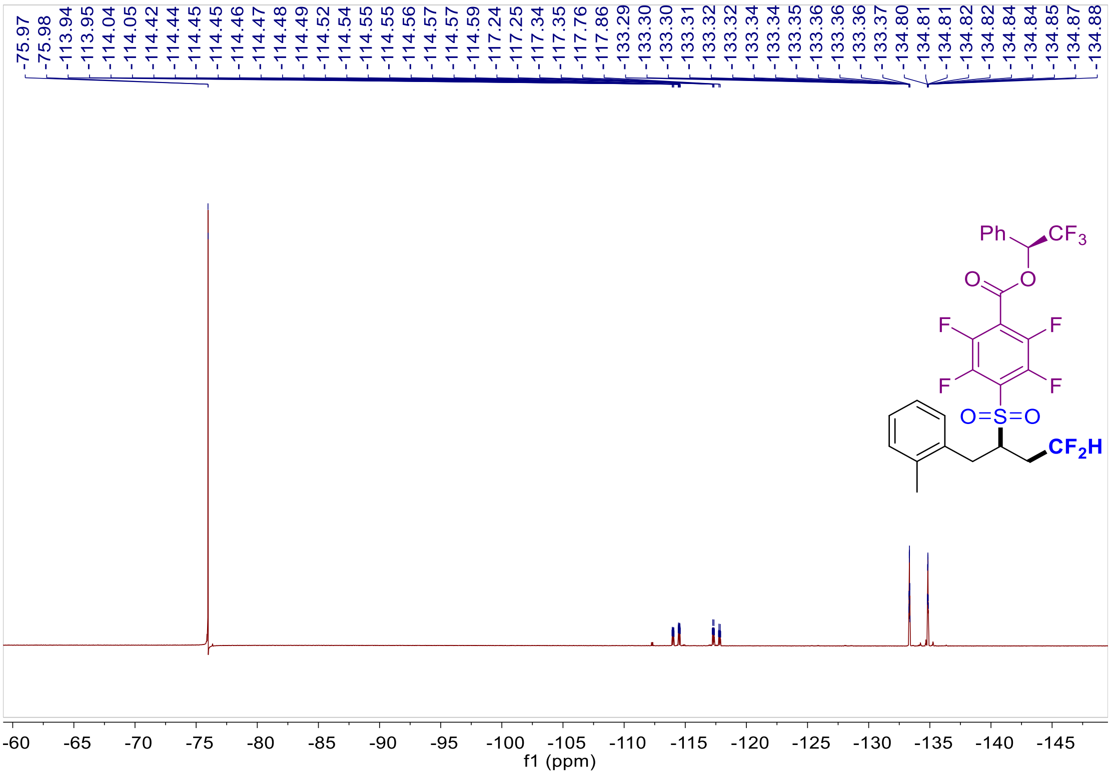

^{1}H NMR (600 MHz, CDCl₃) spectrum of 103

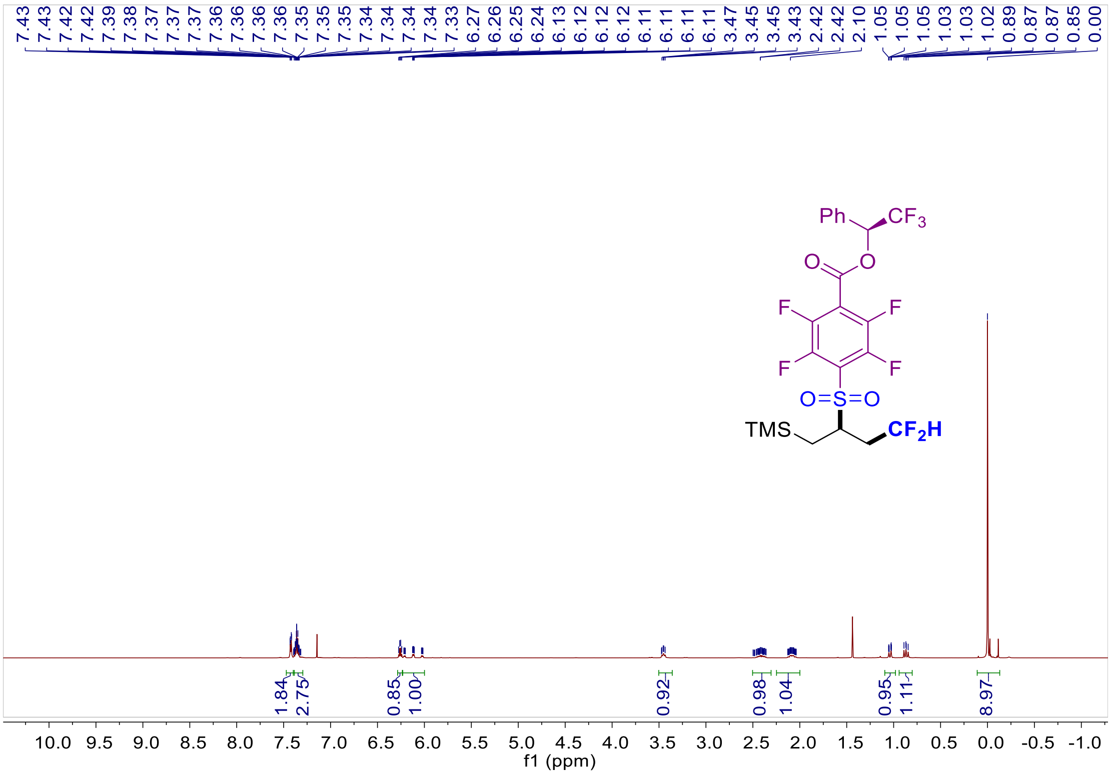

^{13}C NMR (151 MHz, CDCl$_3$) spectrum of 103

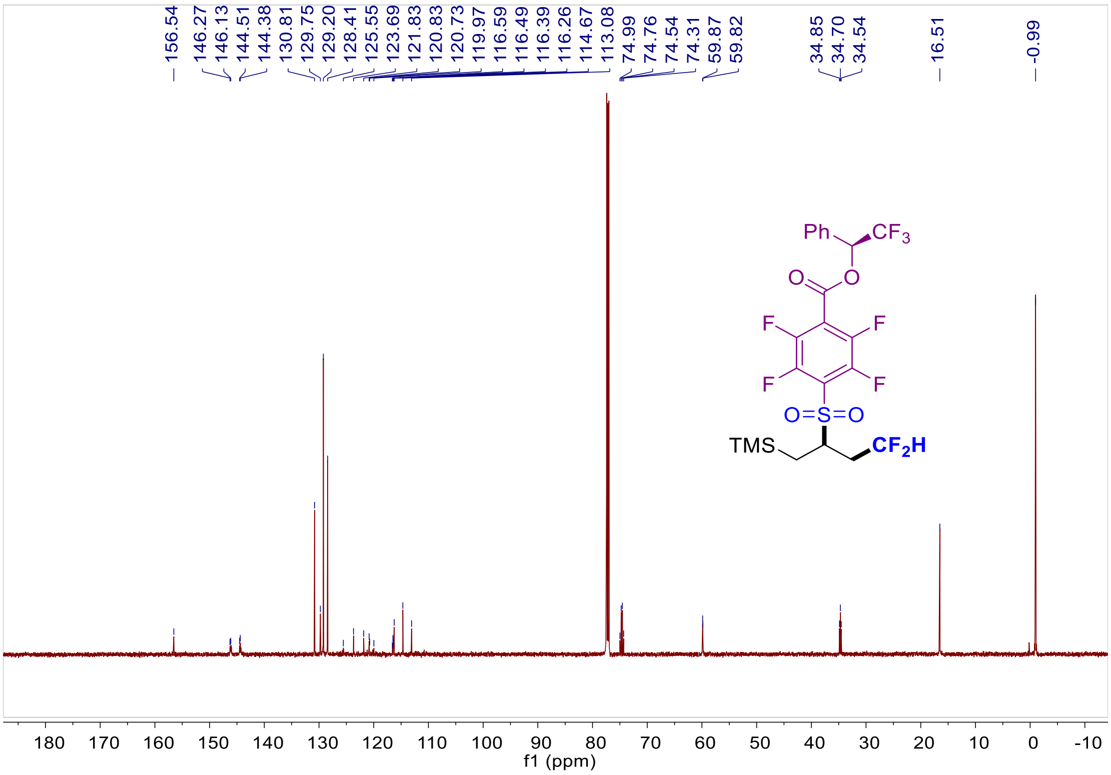

^{19}F NMR (565 MHz, CDCl$_3$) spectrum of 103

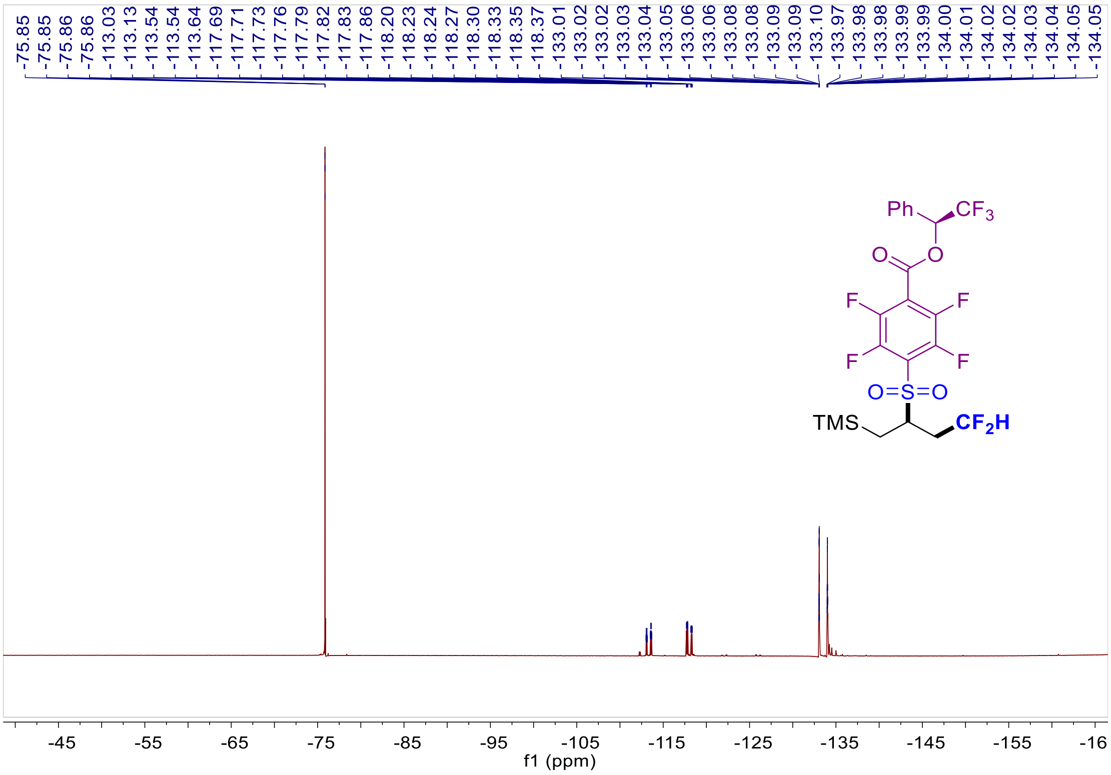

^{1}H NMR (600 MHz, CDCl$_3$) spectrum of 104

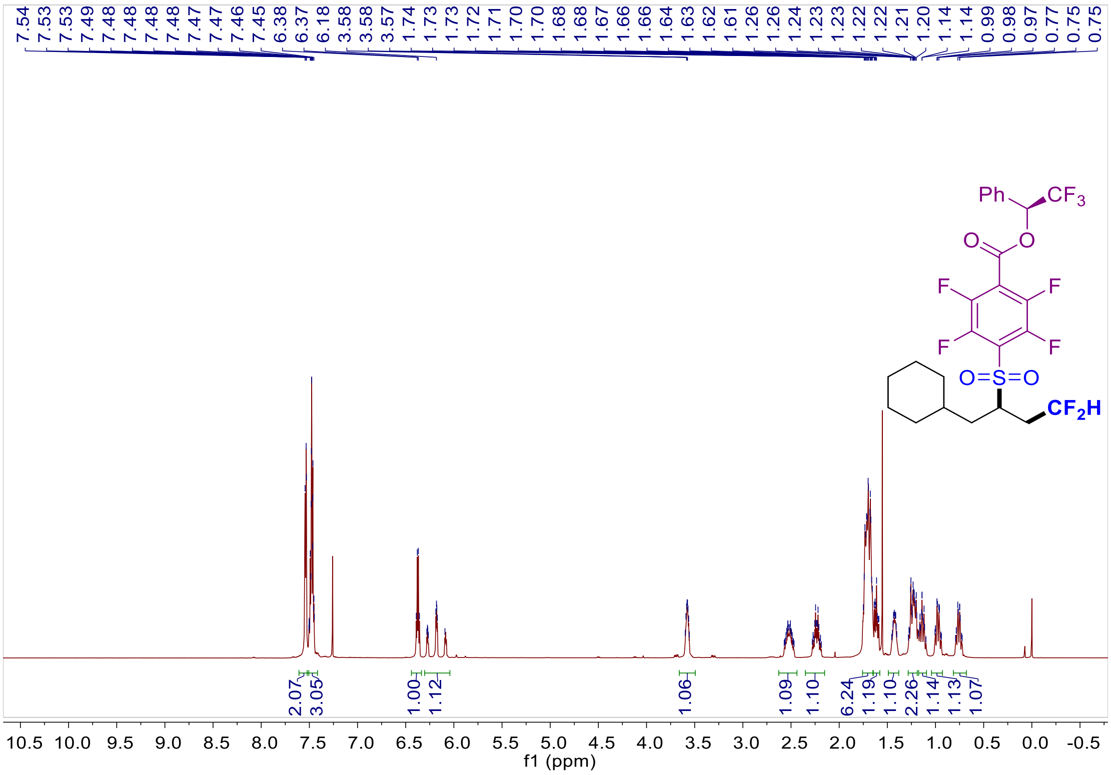

^{13}C NMR (151 MHz, CDCl$_3$) spectrum of 104

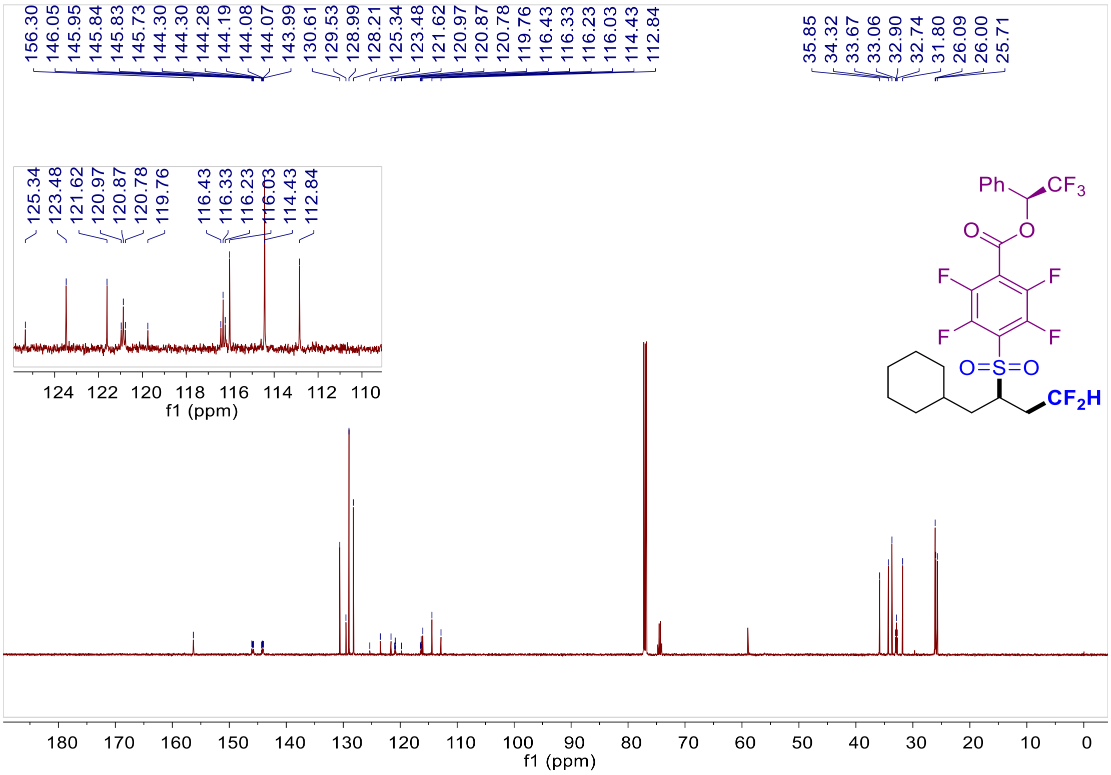

^{19}F NMR (565 MHz, CDCl$_3$) spectrum of 104

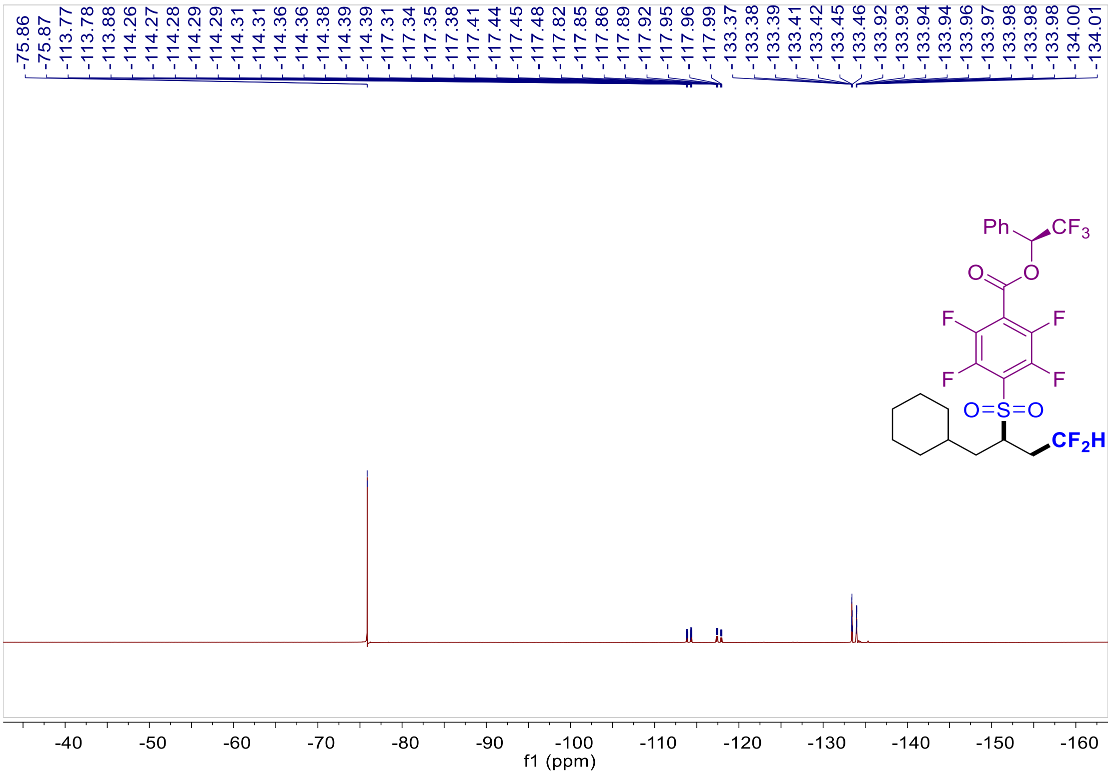

^{1}H NMR (600 MHz, CDCl$_3$) spectrum of 105

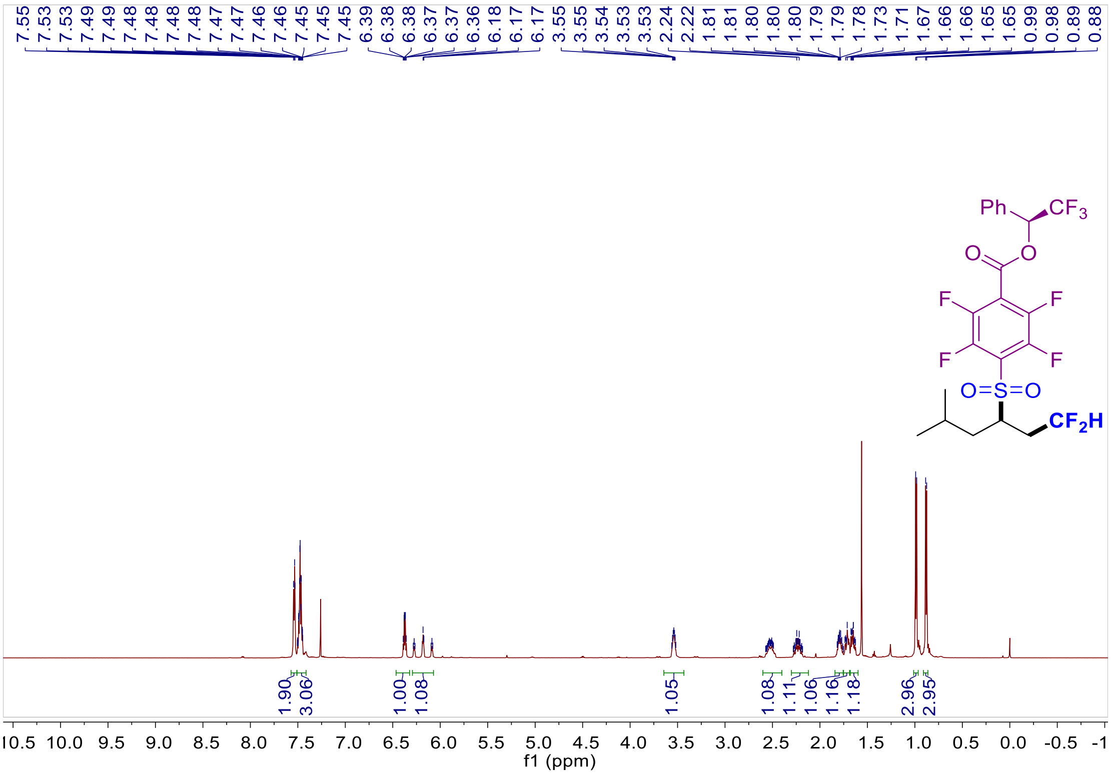

^{13}C NMR (151 MHz, $CDCl_3$) spectrum of 105

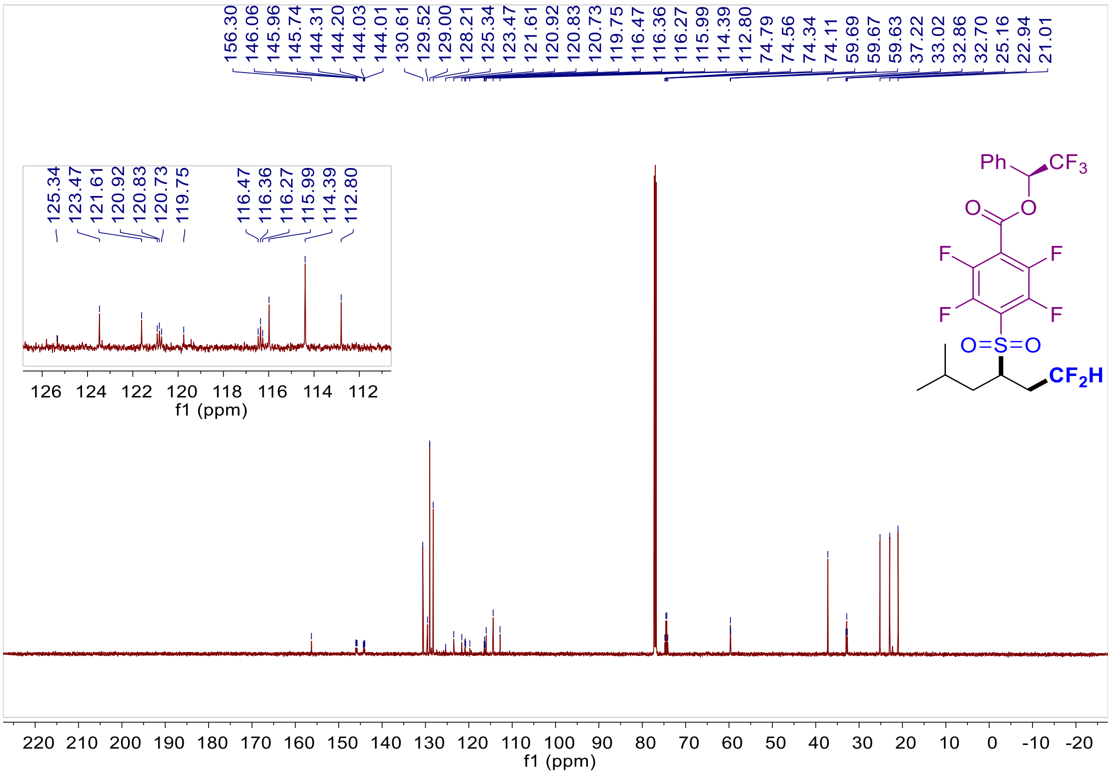

^{19}F NMR (565 MHz, $CDCl_3$) spectrum of 105

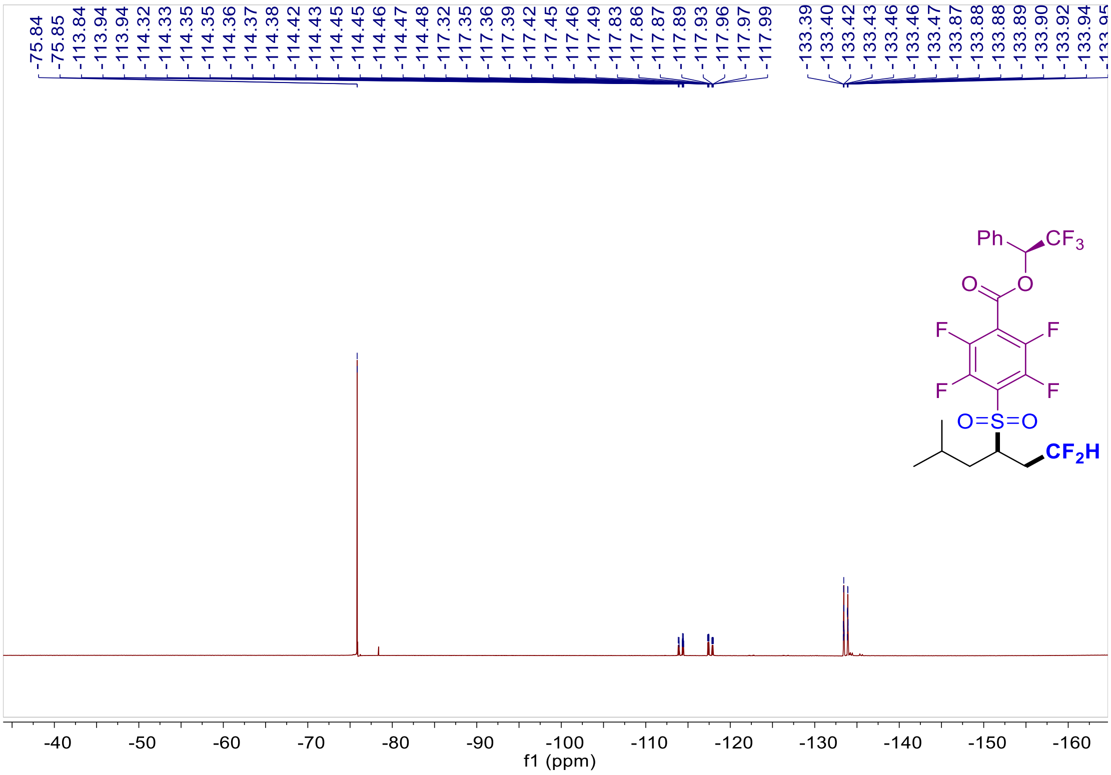

^{1}H NMR (600 MHz, $CDCl_3$) spectrum of 106

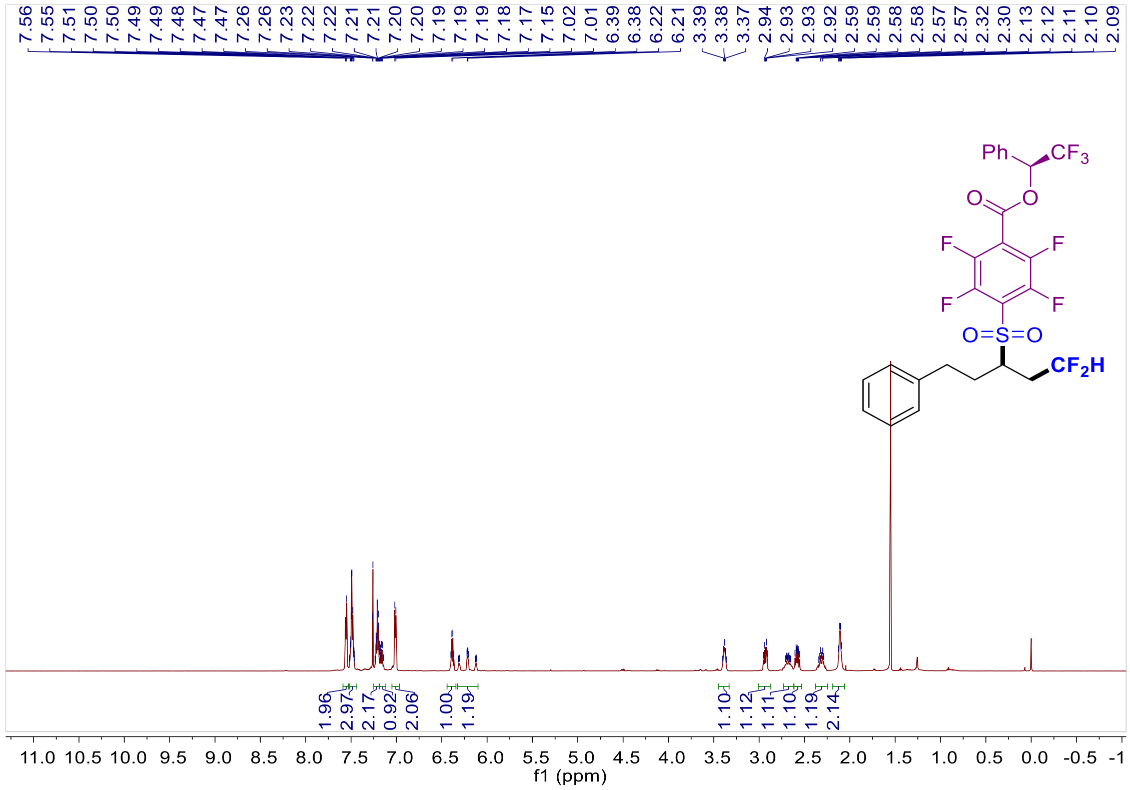

^{13}C NMR (151 MHz, $CDCl_3$) spectrum of 106

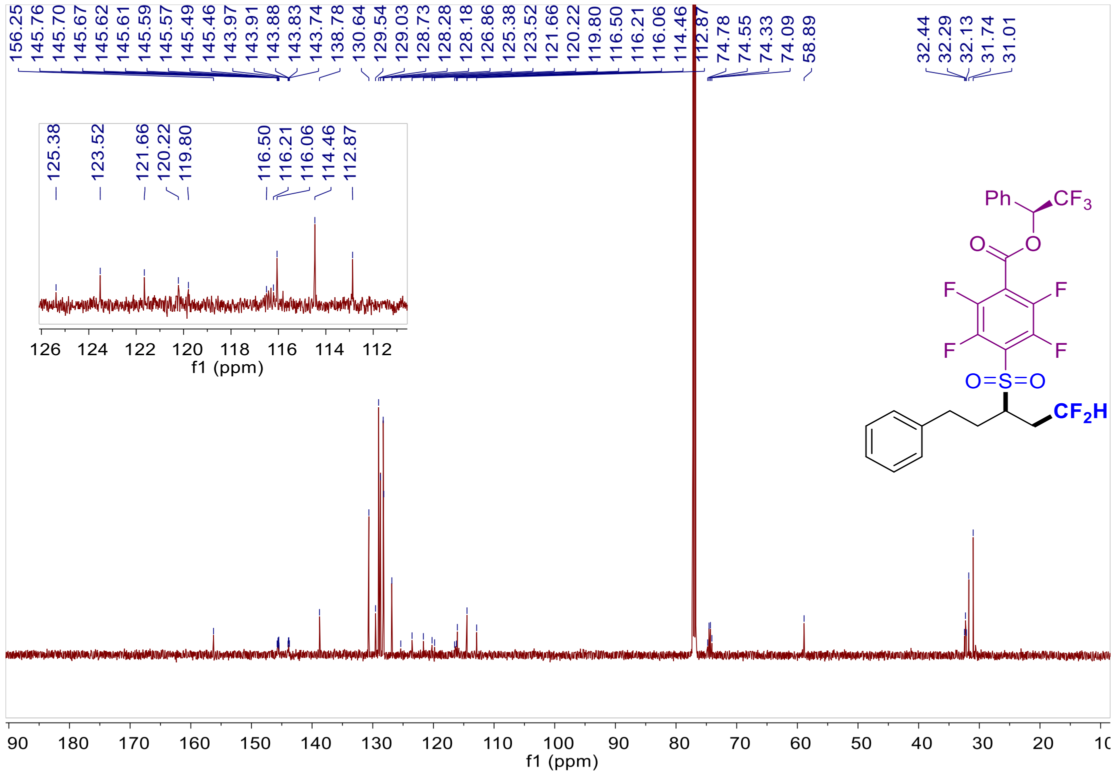

^{19}F NMR (565 MHz, $CDCl_3$) spectrum of 106

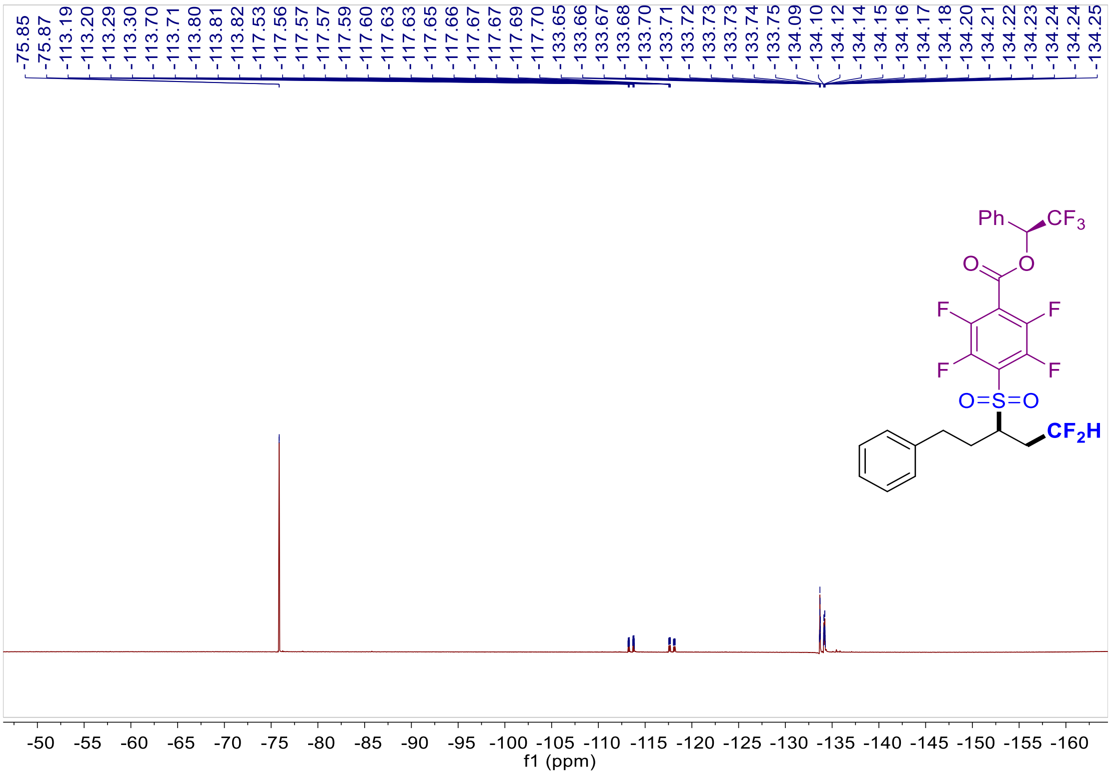

^1H NMR (600 MHz, $CDCl_3$) spectrum of 107

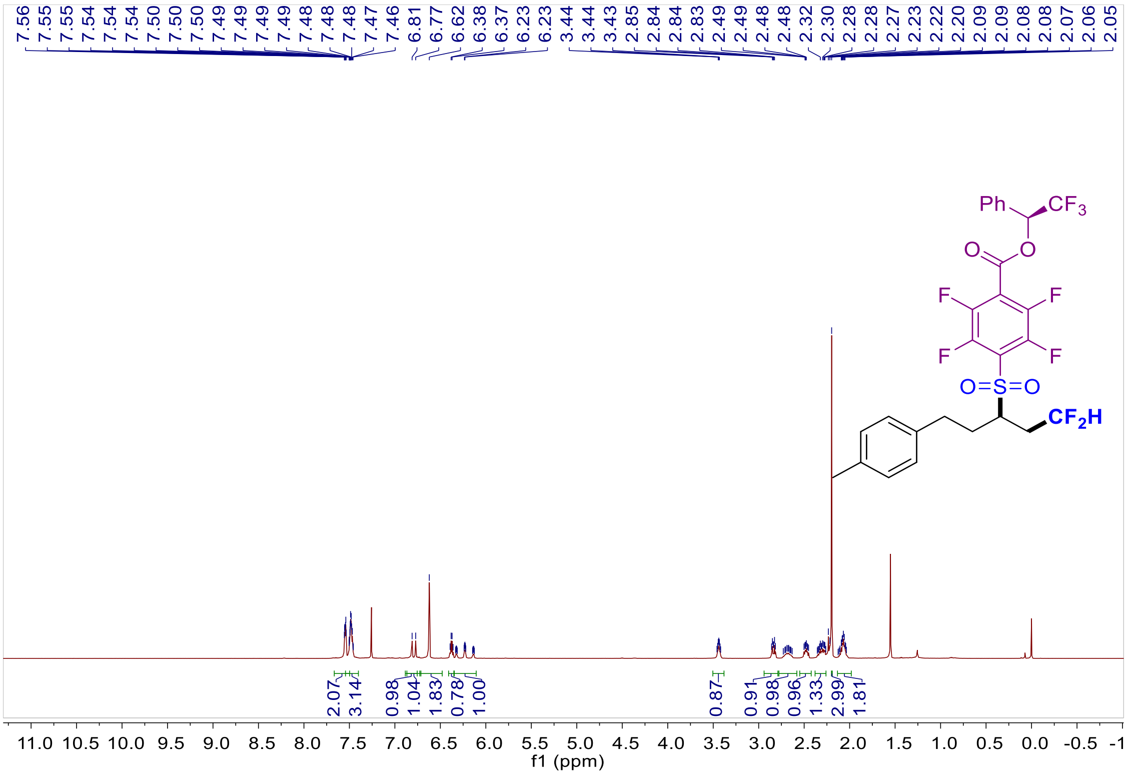

^{13}C NMR (151 MHz, $CDCl_3$) spectrum of 107

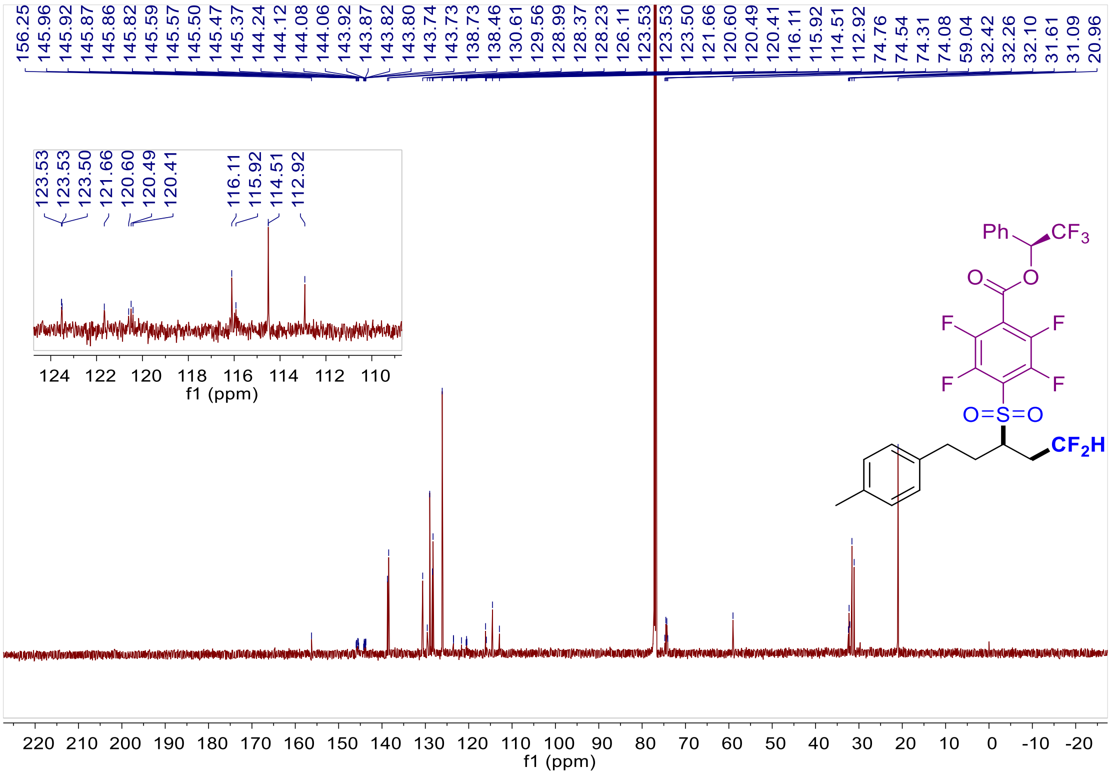

^{19}F NMR (565 MHz, $CDCl_3$) spectrum of 107

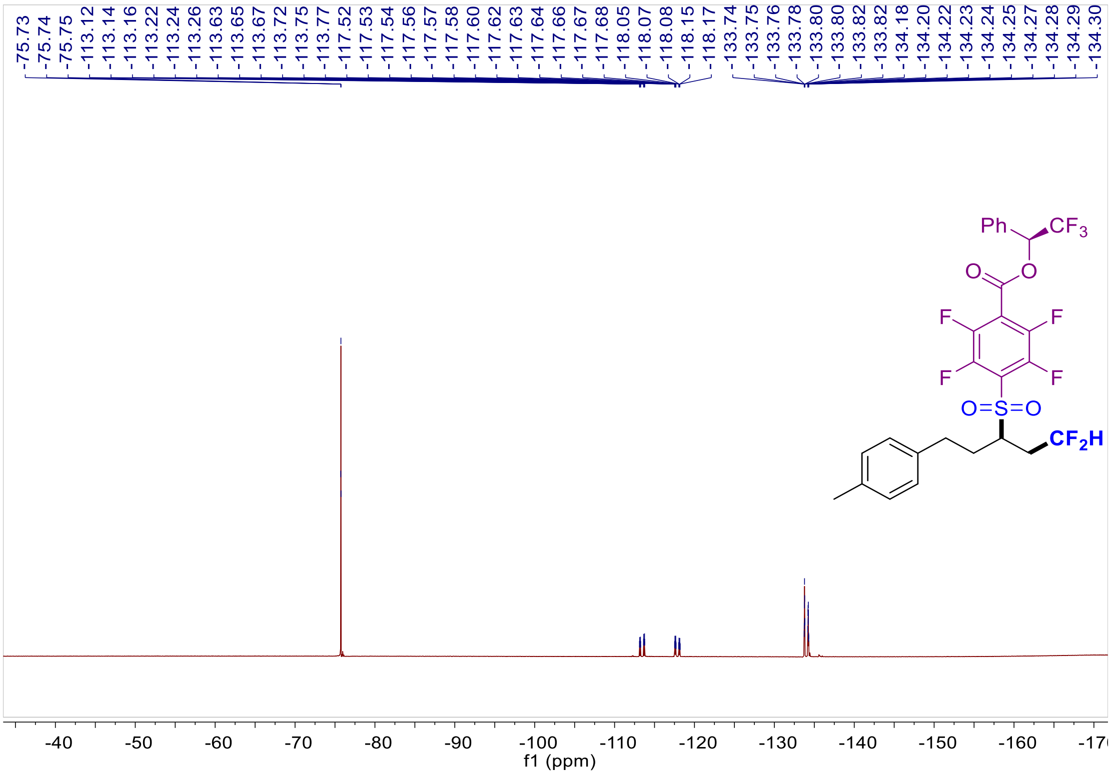

^{1}H NMR (600 MHz, $CDCl_3$) spectrum of 108

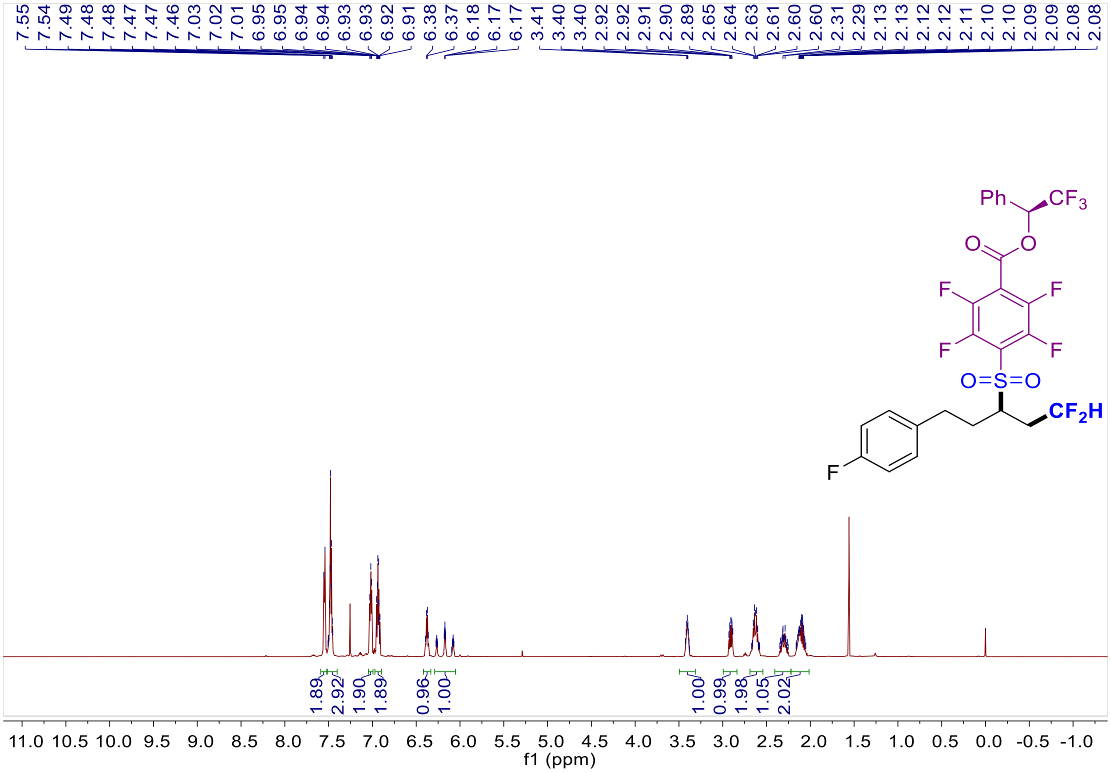

^{13}C NMR (151 MHz, $CDCl_3$) spectrum of 108

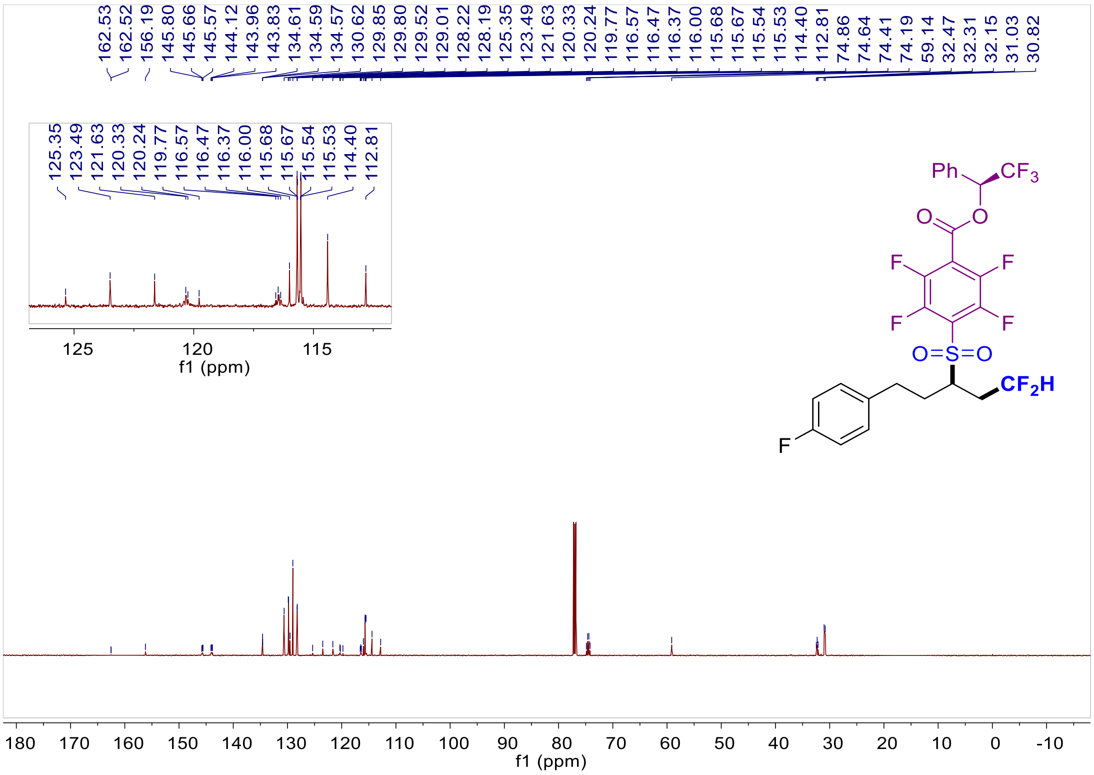

^{19}F NMR (565 MHz, CDCl3) spectrum of 108

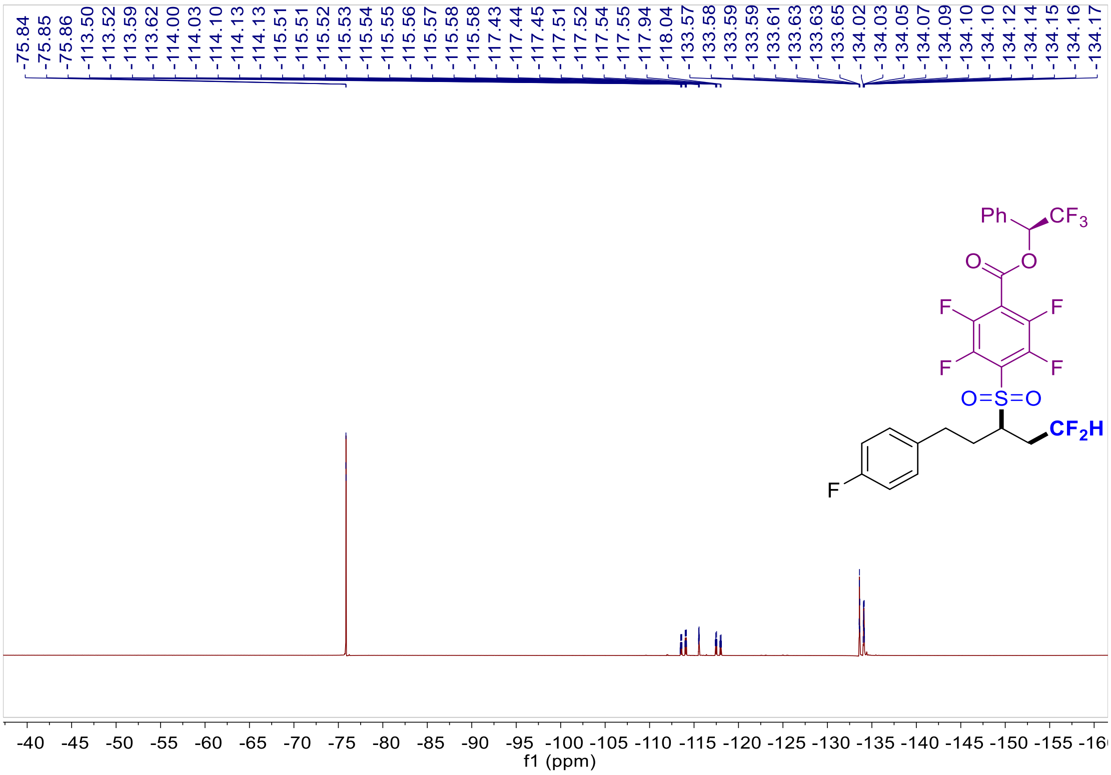

^{1}H NMR (600 MHz, CDCl3) spectrum of 109

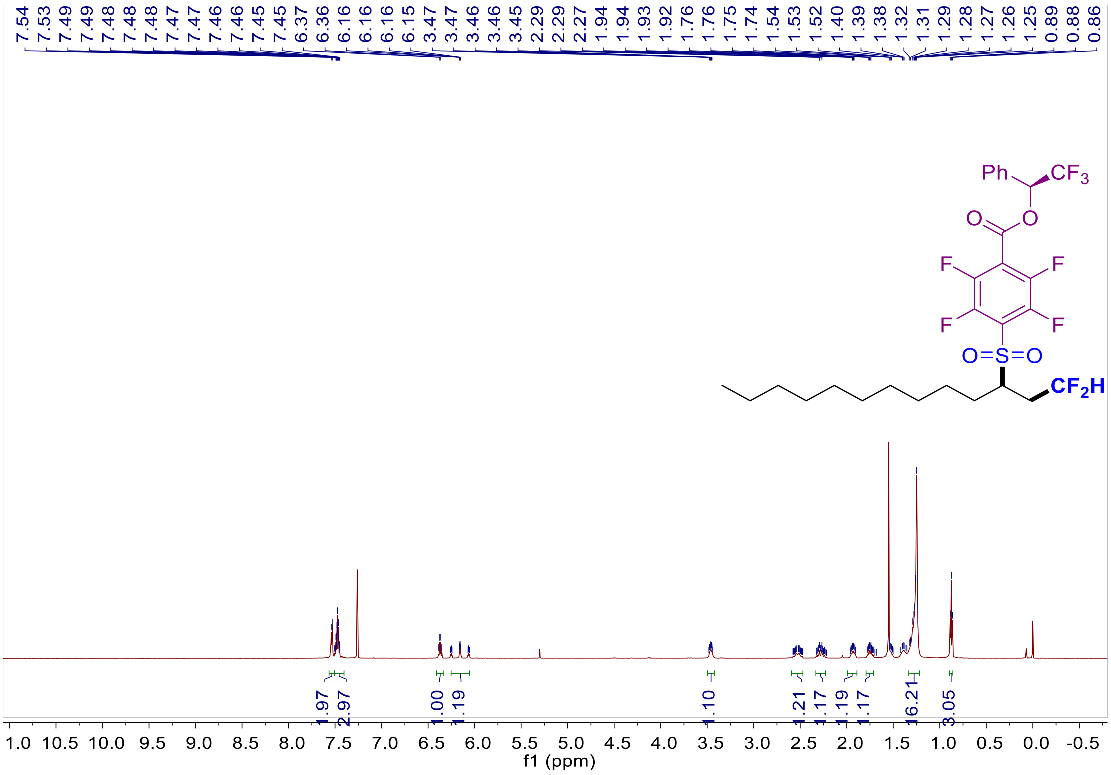

^{13}C NMR (151 MHz, CDCl$_3$) spectrum of 109

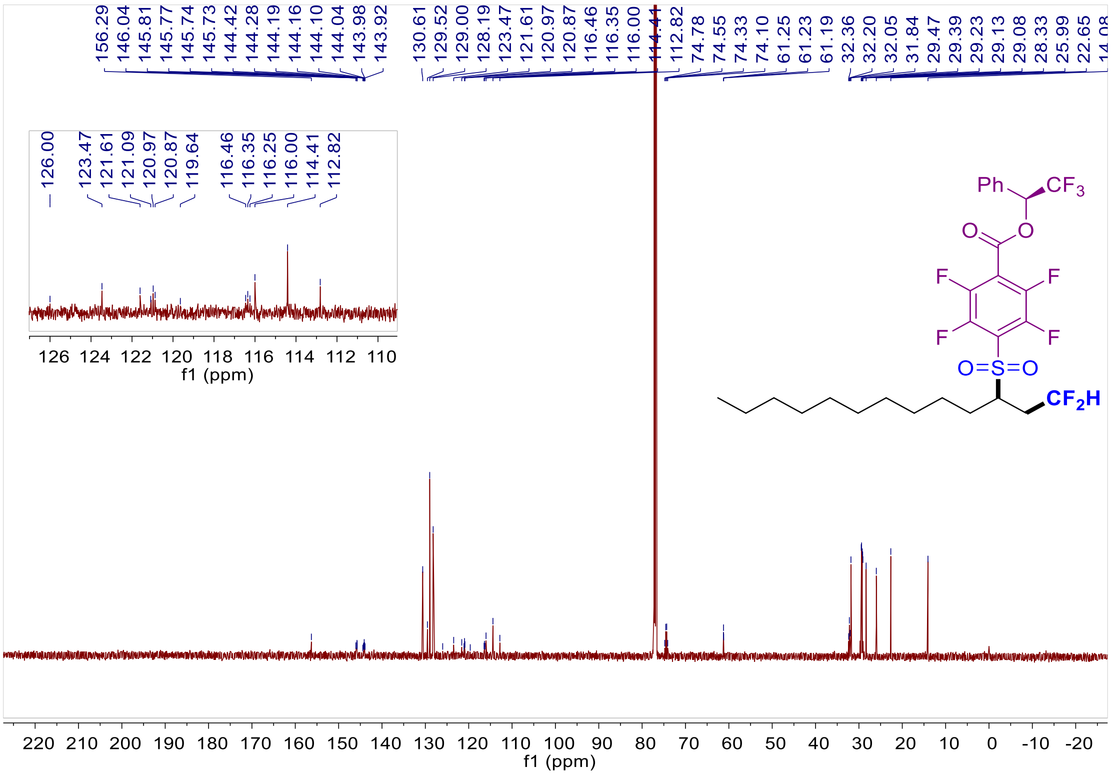

^{19}F NMR (565 MHz, CDCl$_3$) spectrum of 109

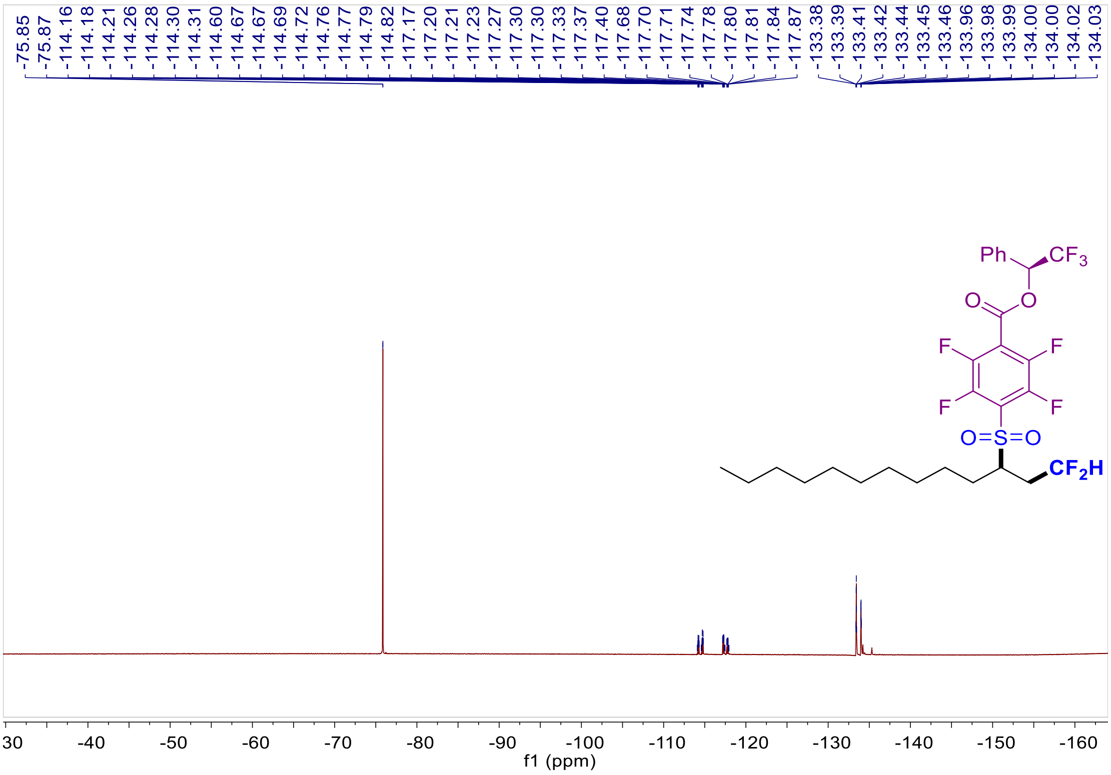

^{1}H NMR (600 MHz, CDCl3) spectrum of 110

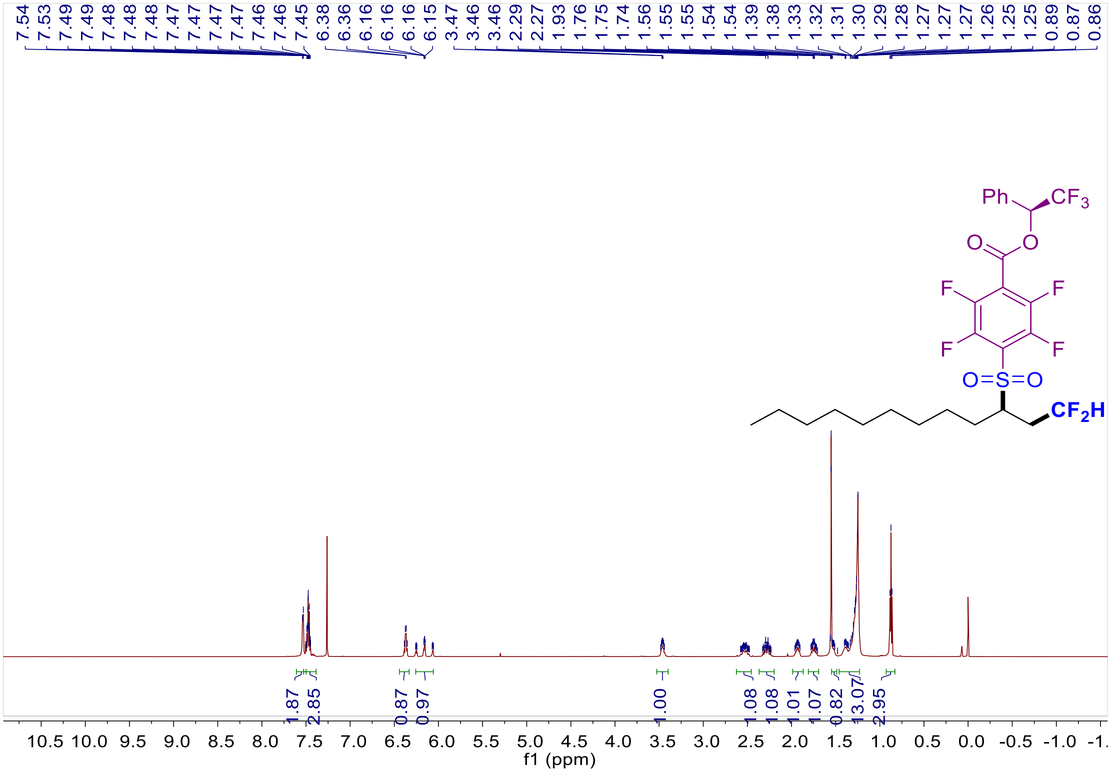

^{13}C NMR (151 MHz, CDCl3) spectrum of 110

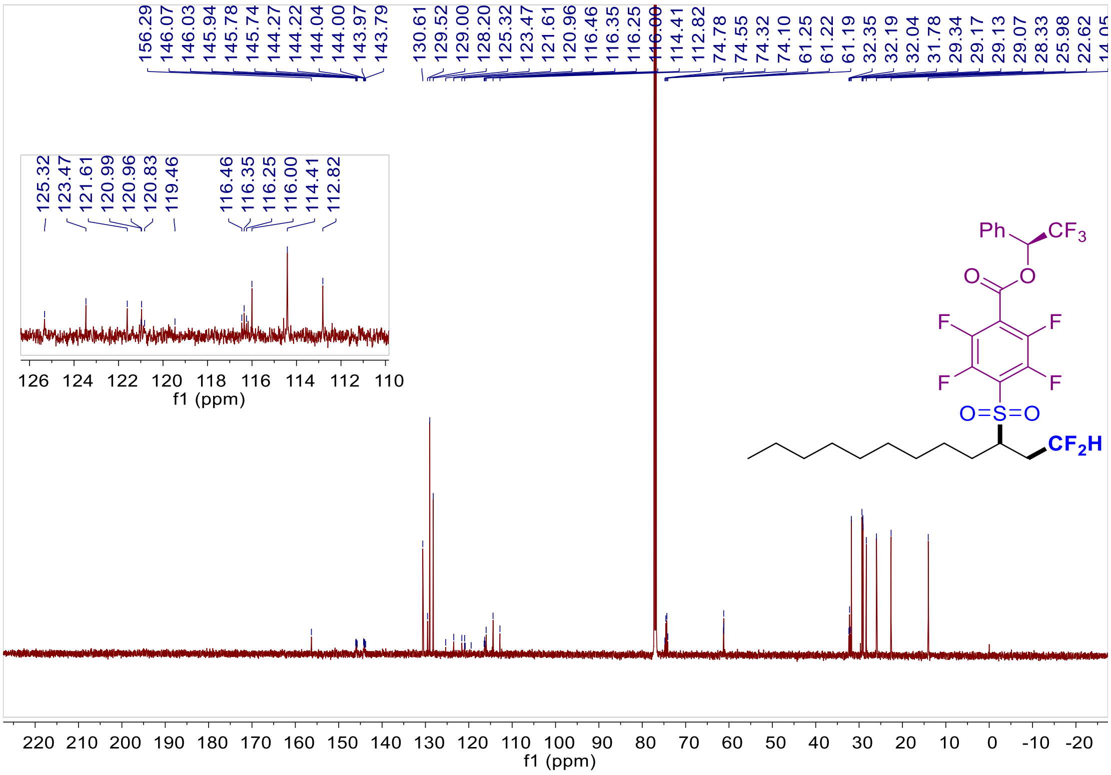

^{19}F NMR (565 MHz, CDCl$_3$) spectrum of 110

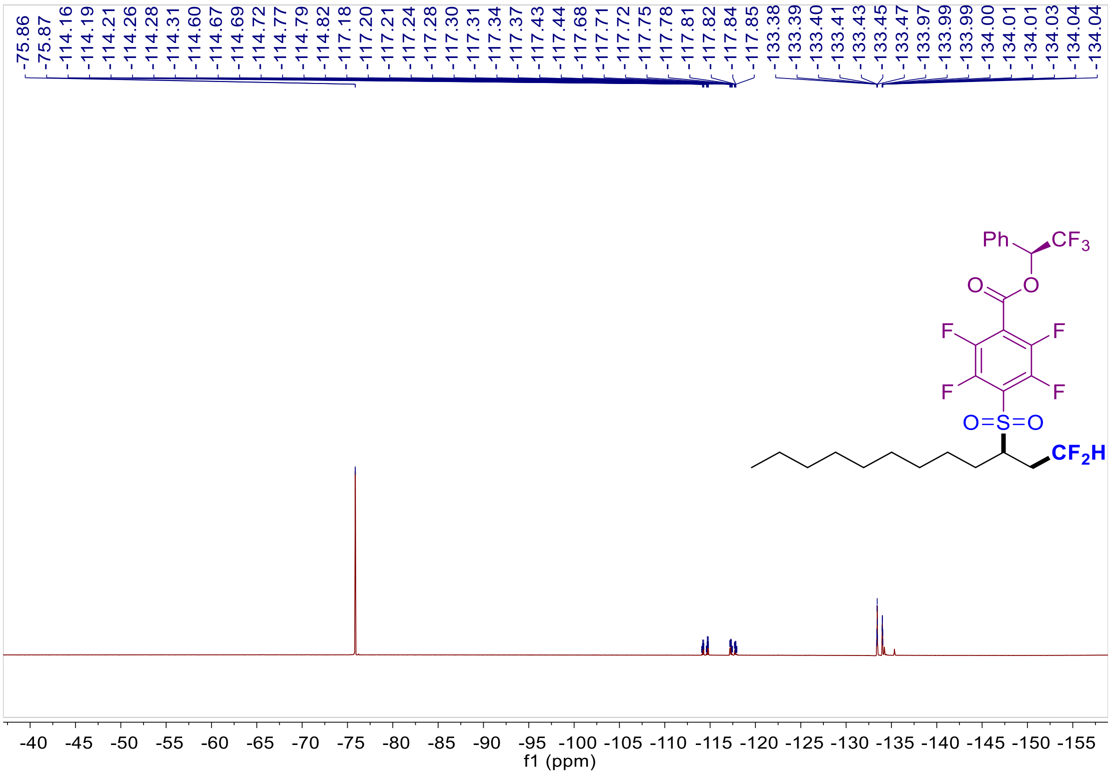

^{1}H NMR (600 MHz, CDCl$_3$) spectrum of 111

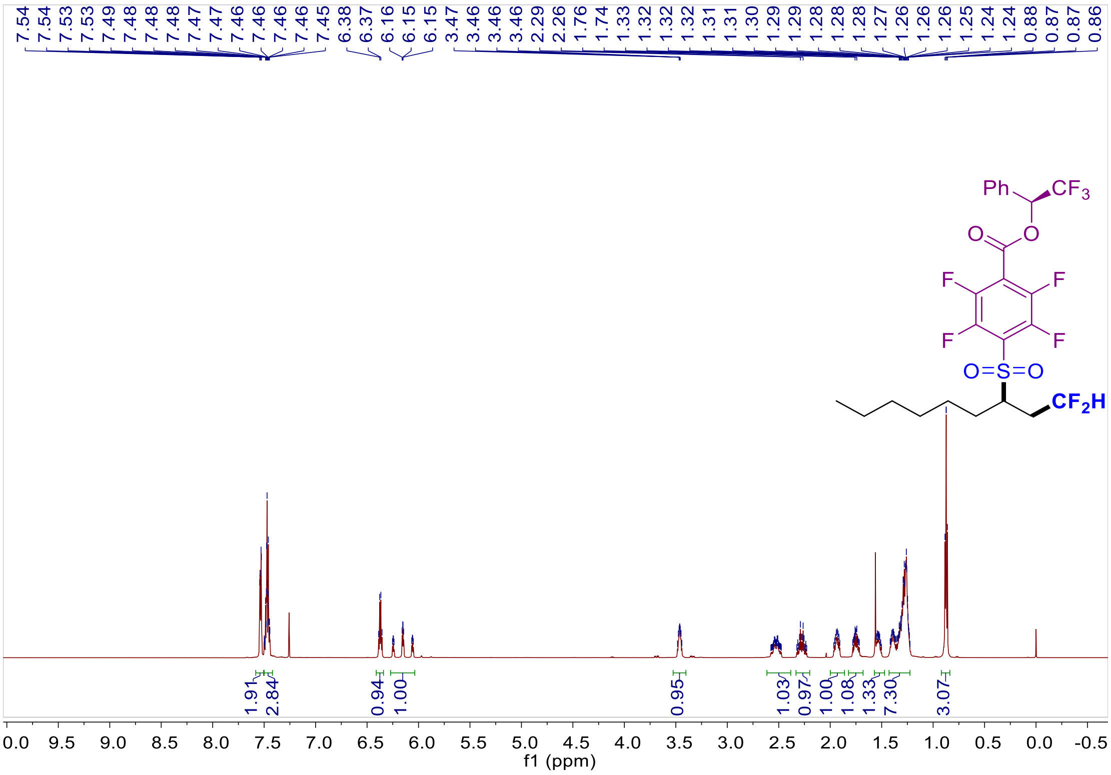

^{13}C NMR (151 MHz, $CDCl_3$) spectrum of 111

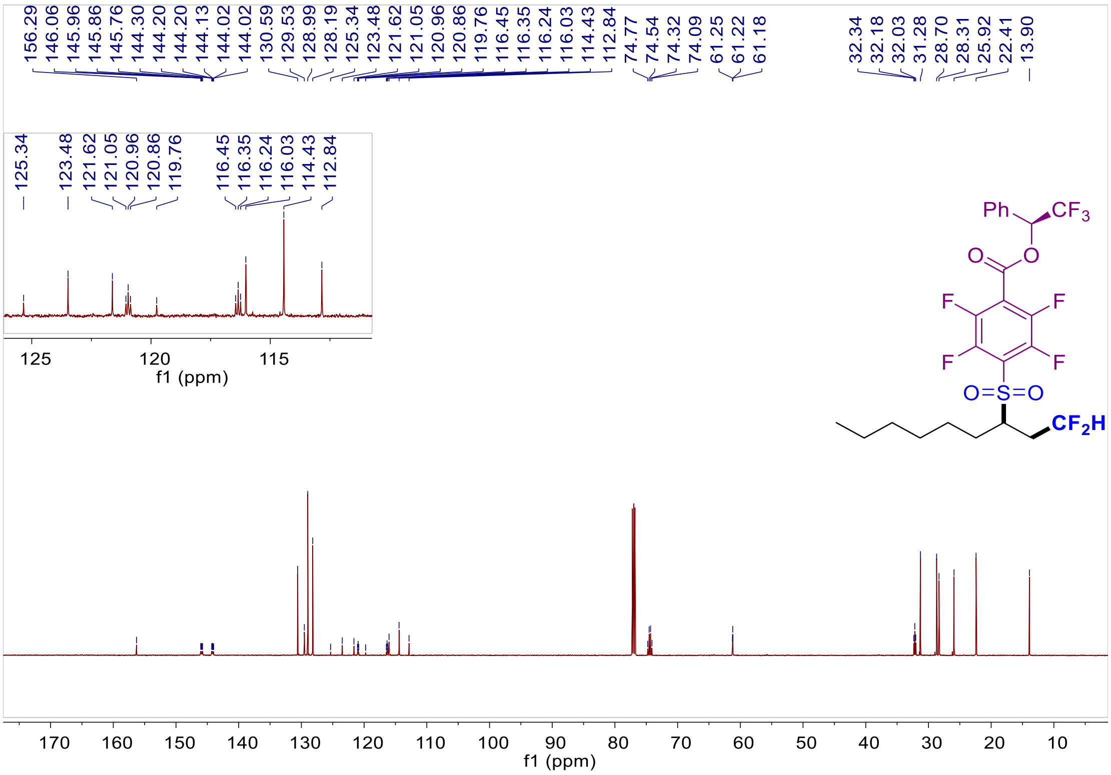

^{19}F NMR (565 MHz, $CDCl_3$) spectrum of 111

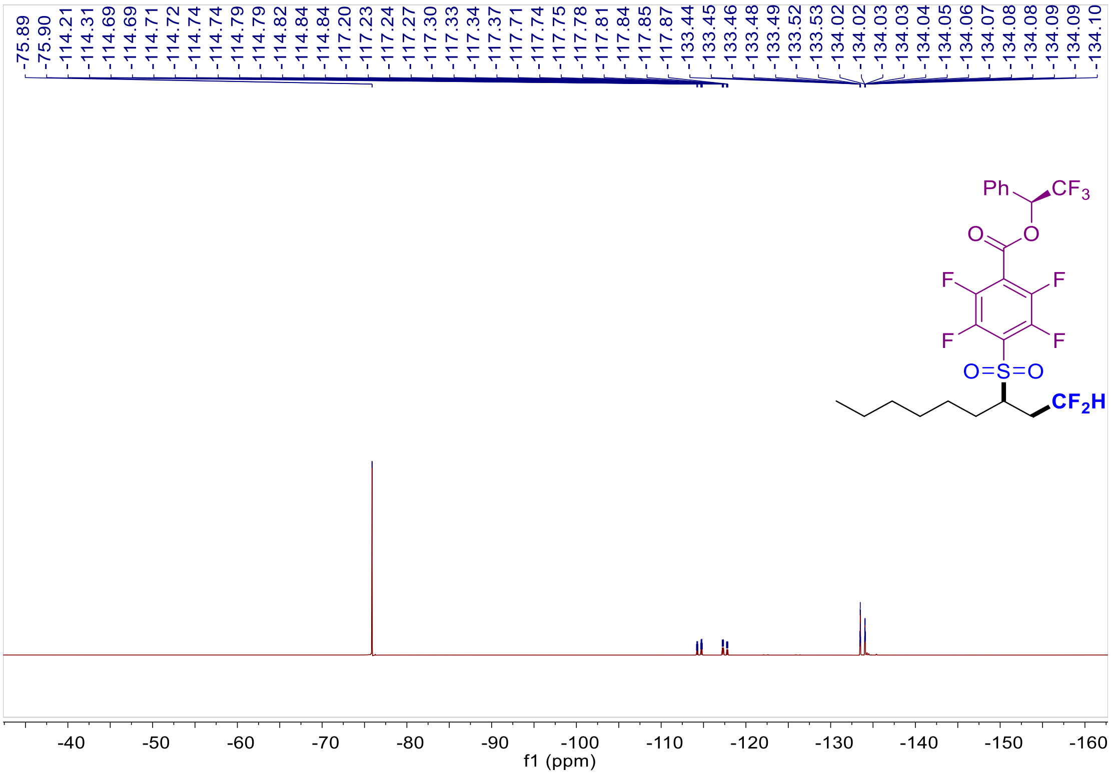

^{1}H NMR (600 MHz, CDCl₃) spectrum of 112

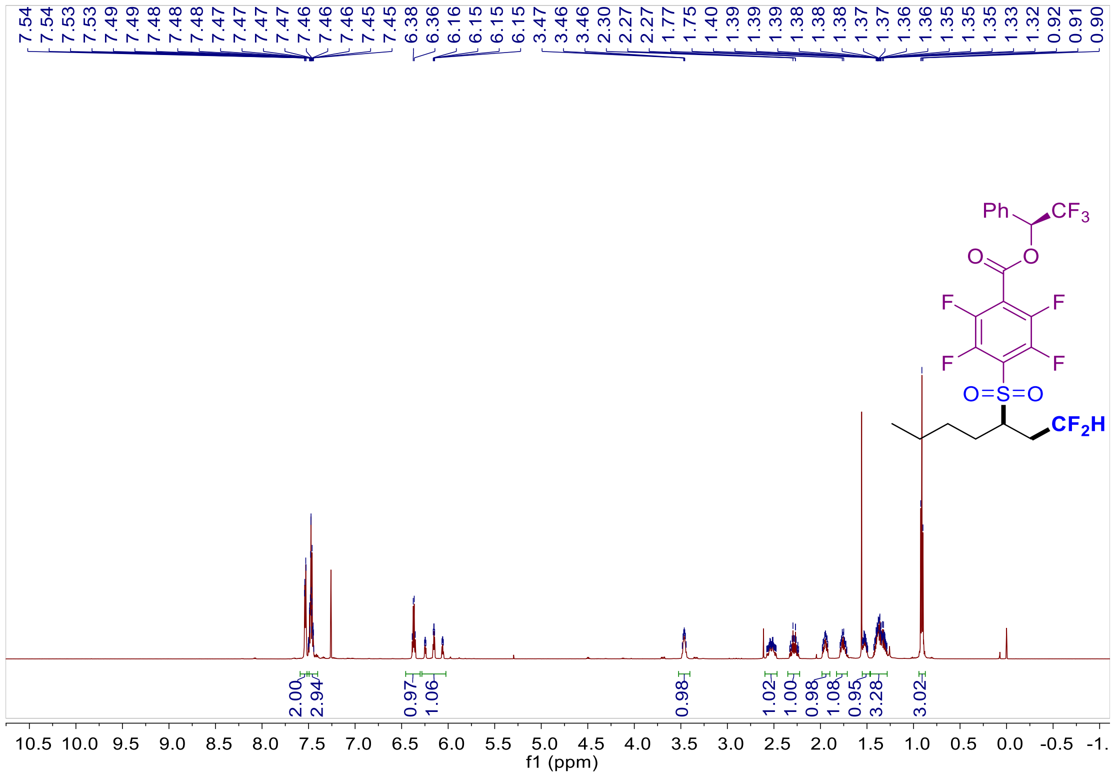

^{13}C NMR (151 MHz, CDCl₃) spectrum of 112

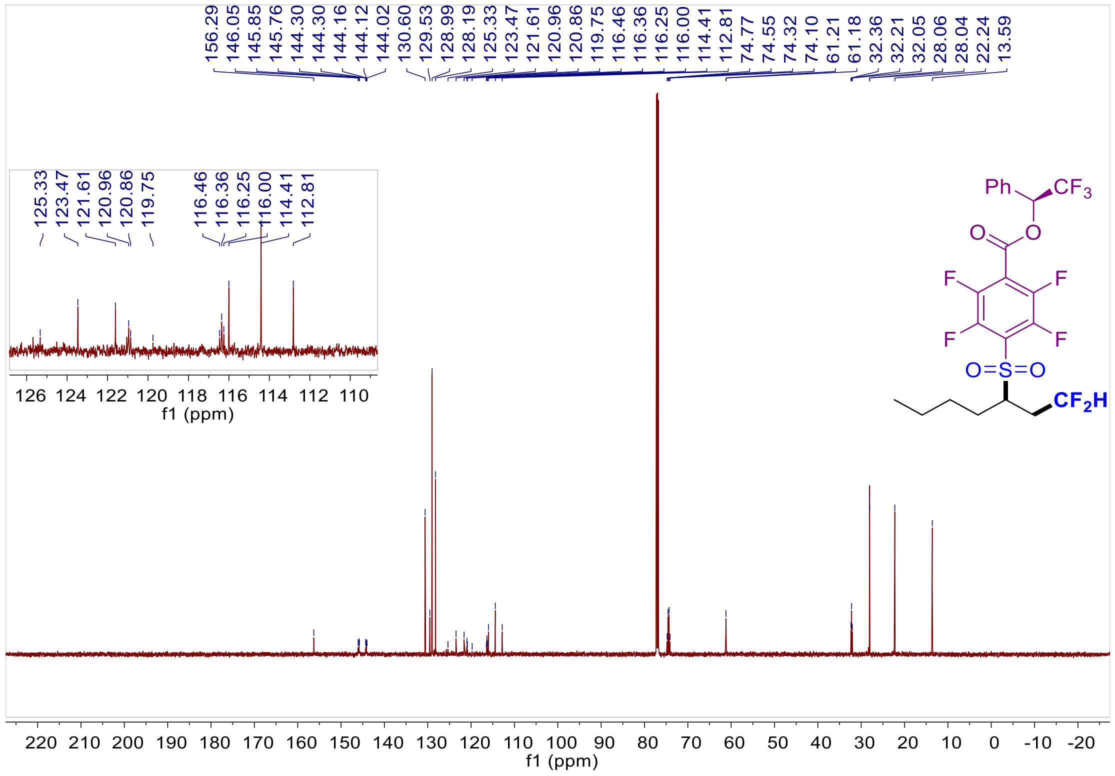

^{19}F NMR (565 MHz, CDCl3) spectrum of 112

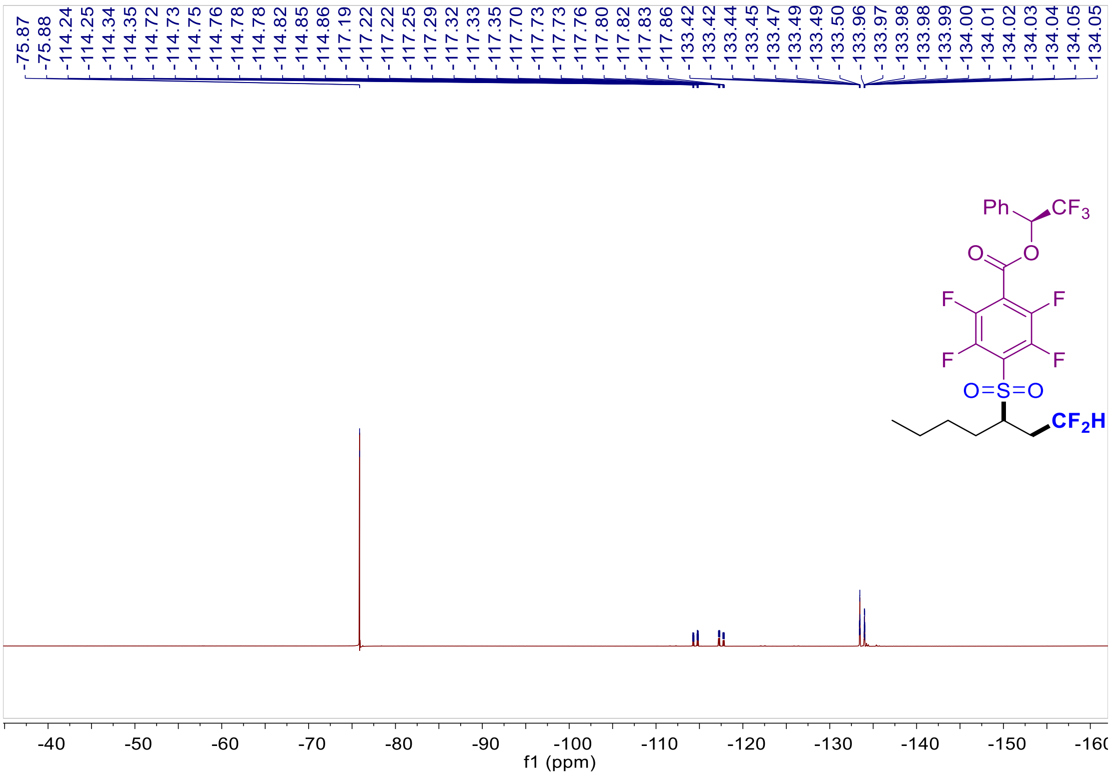

^{1}H NMR (600 MHz, CDCl3) spectrum of 113

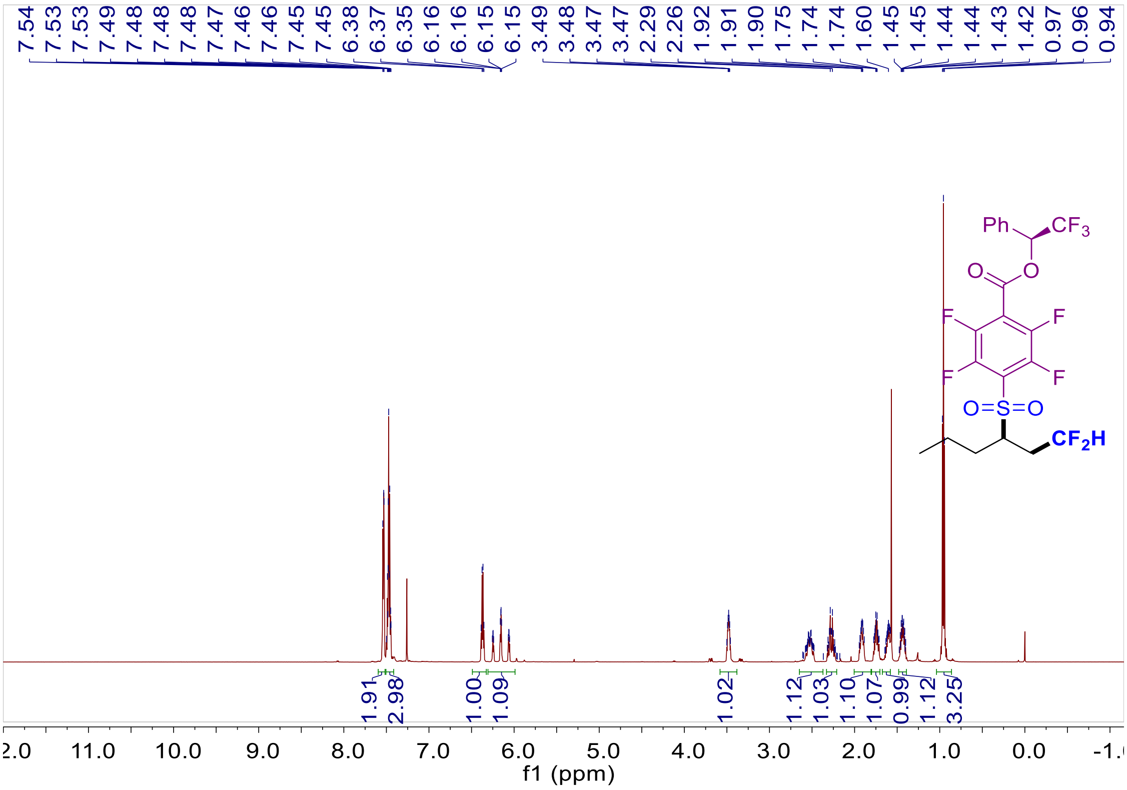

^{13}C NMR (151 MHz, CDCl$_3$) spectrum of 113

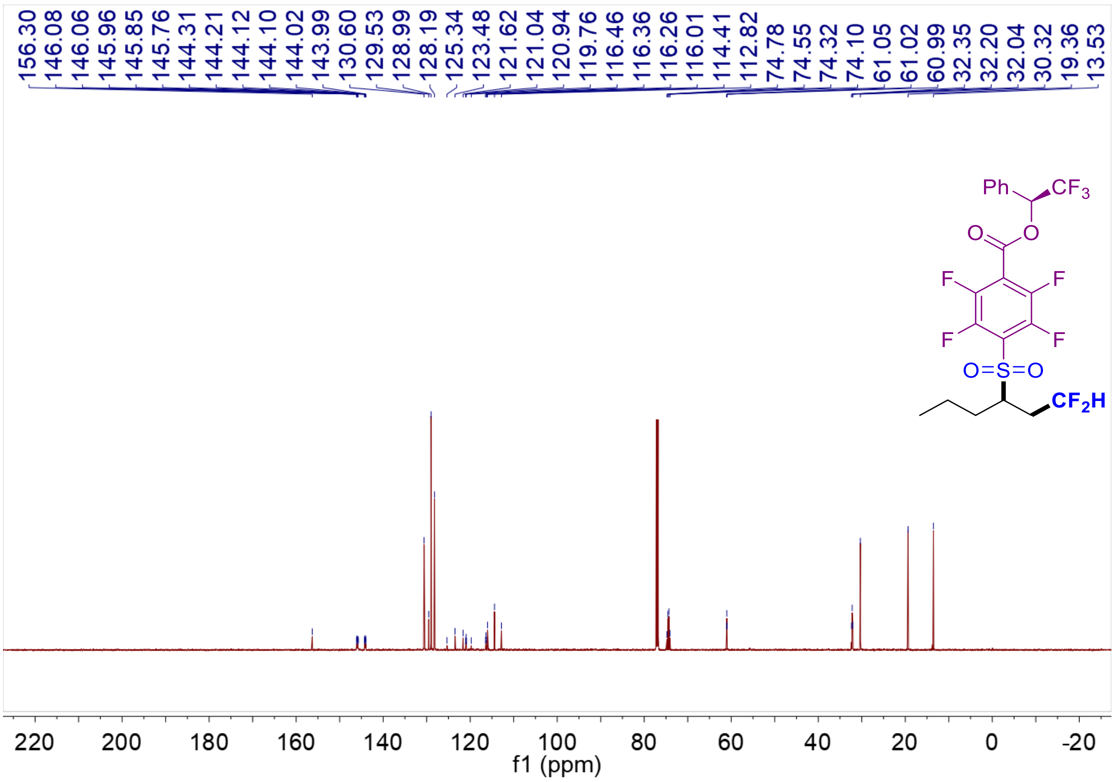

^{19}F NMR (565 MHz, CDCl$_3$) spectrum of 113

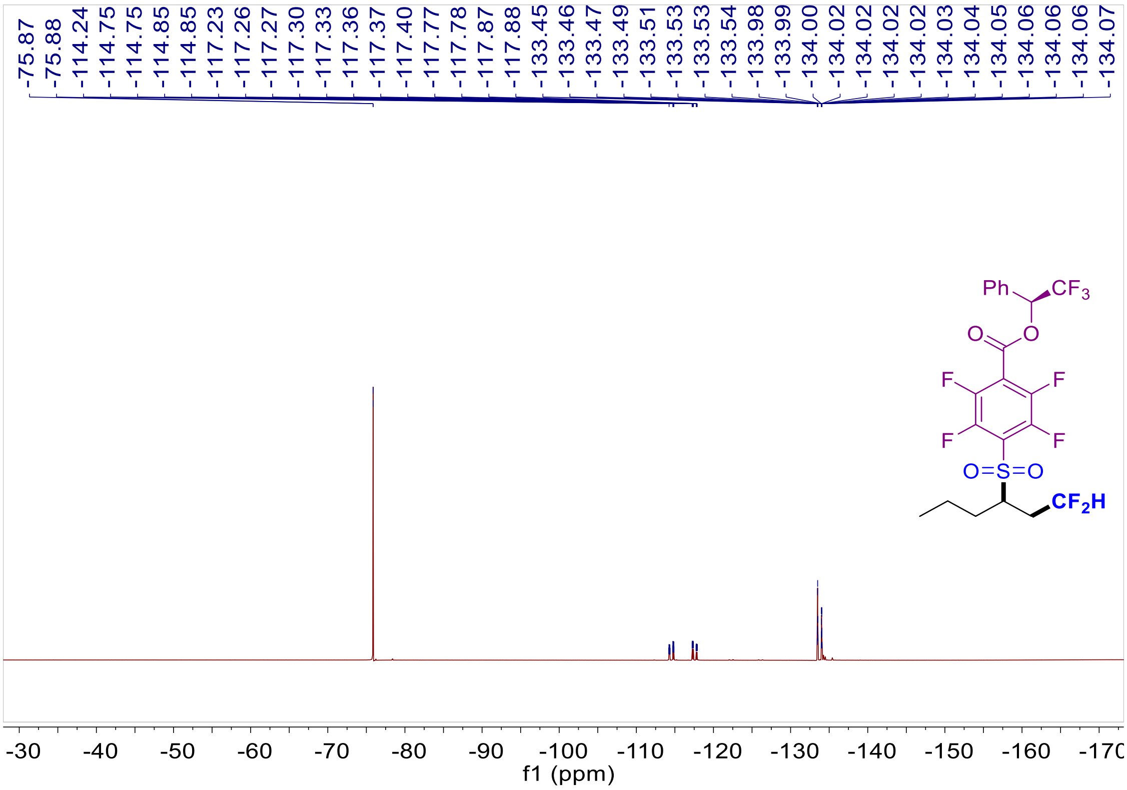

^{1}H NMR (600 MHz, $CDCl_3$) spectrum of 114

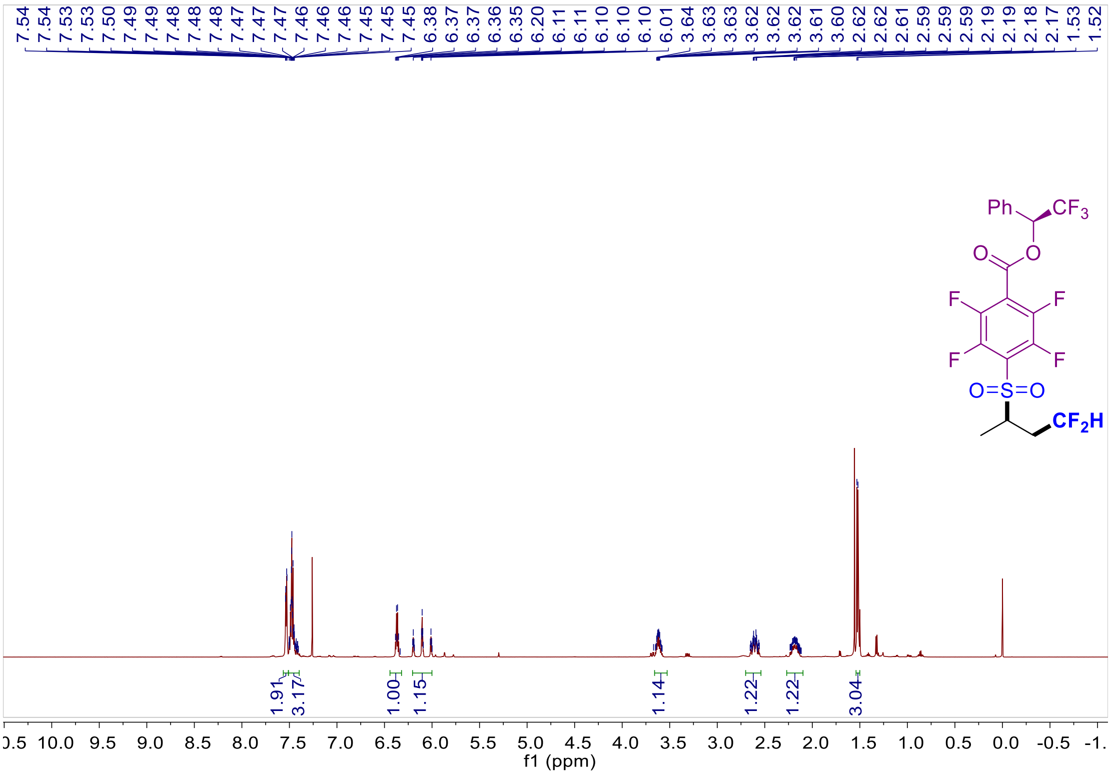

^{13}C NMR (151 MHz, $CDCl_3$) spectrum of 114

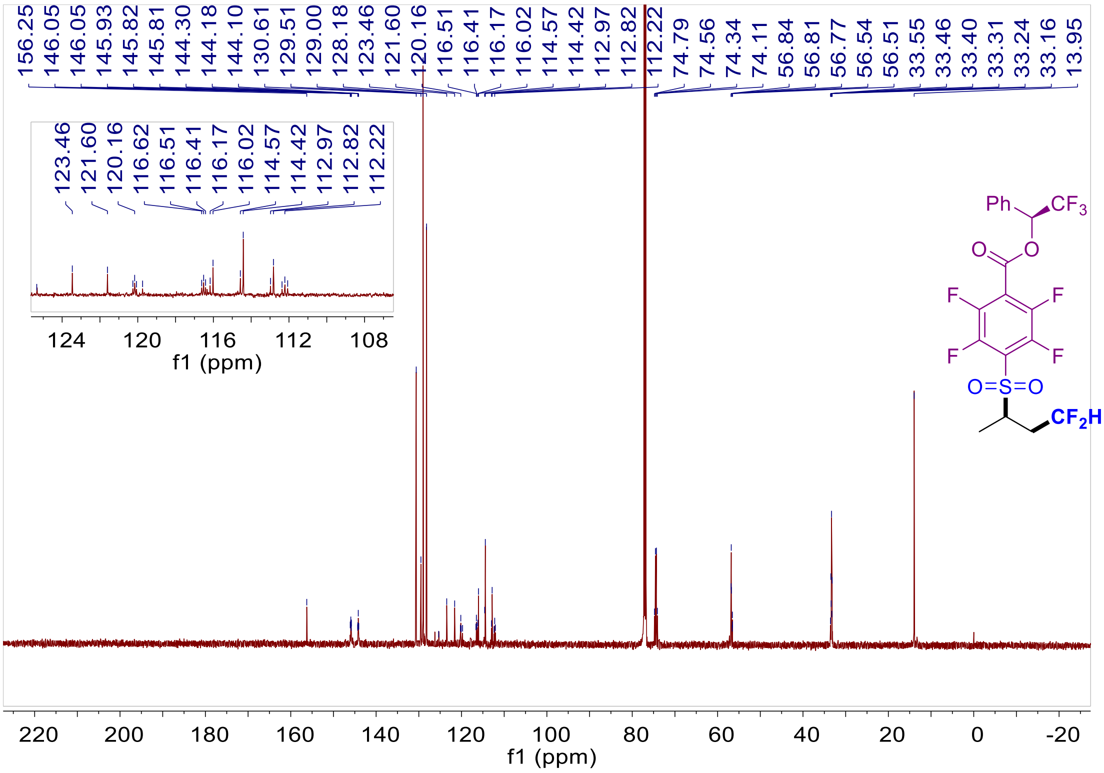

^{19}F NMR (565 MHz, CDCl3) spectrum of 114

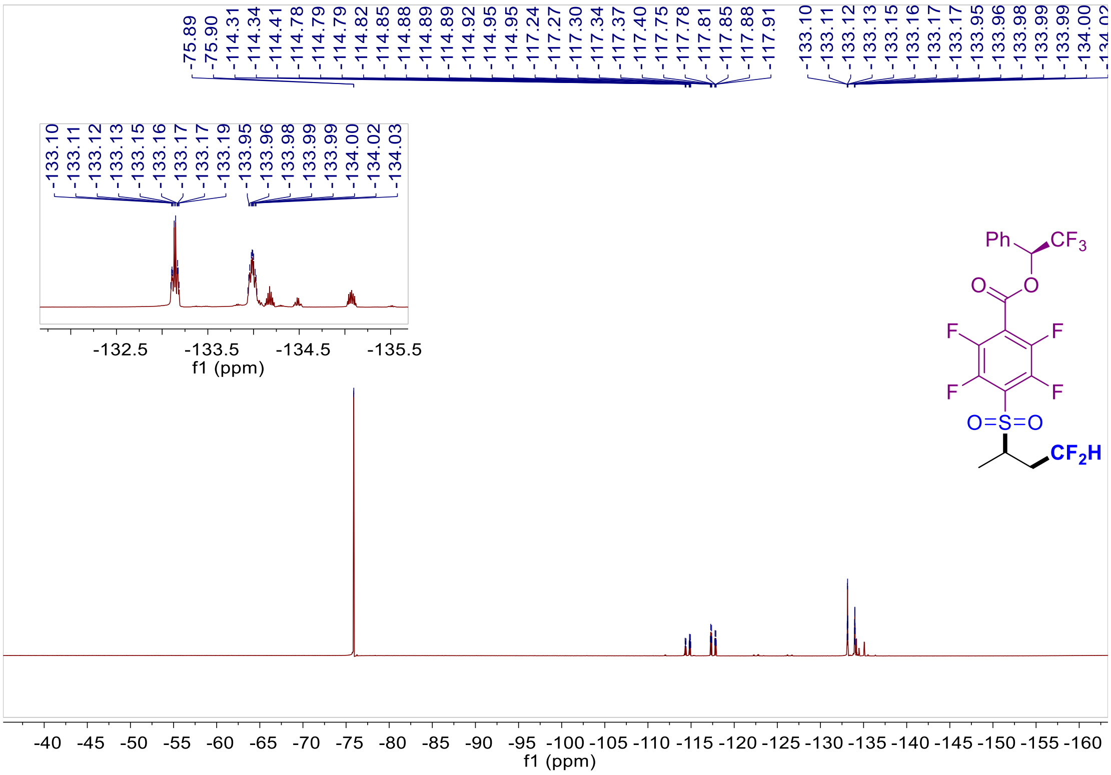

^{1}H NMR (600 MHz, CDCl3) spectrum of 115

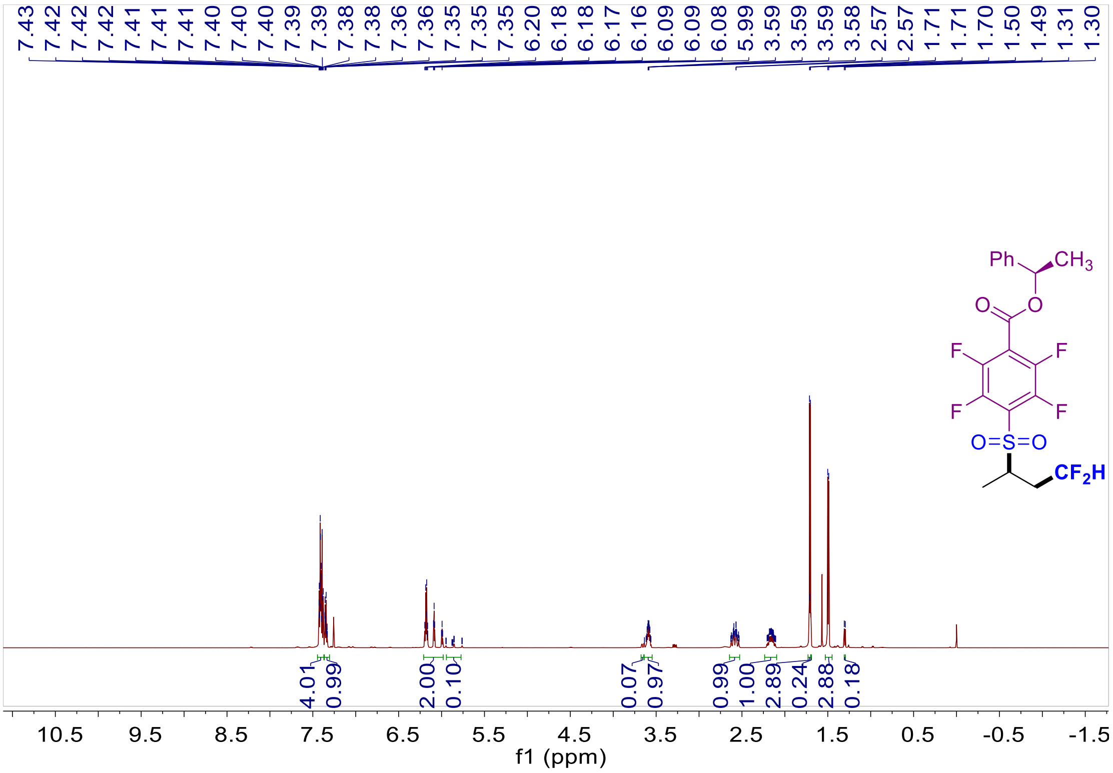

^{13}C NMR (151 MHz, $CDCl_3$) spectrum of 115

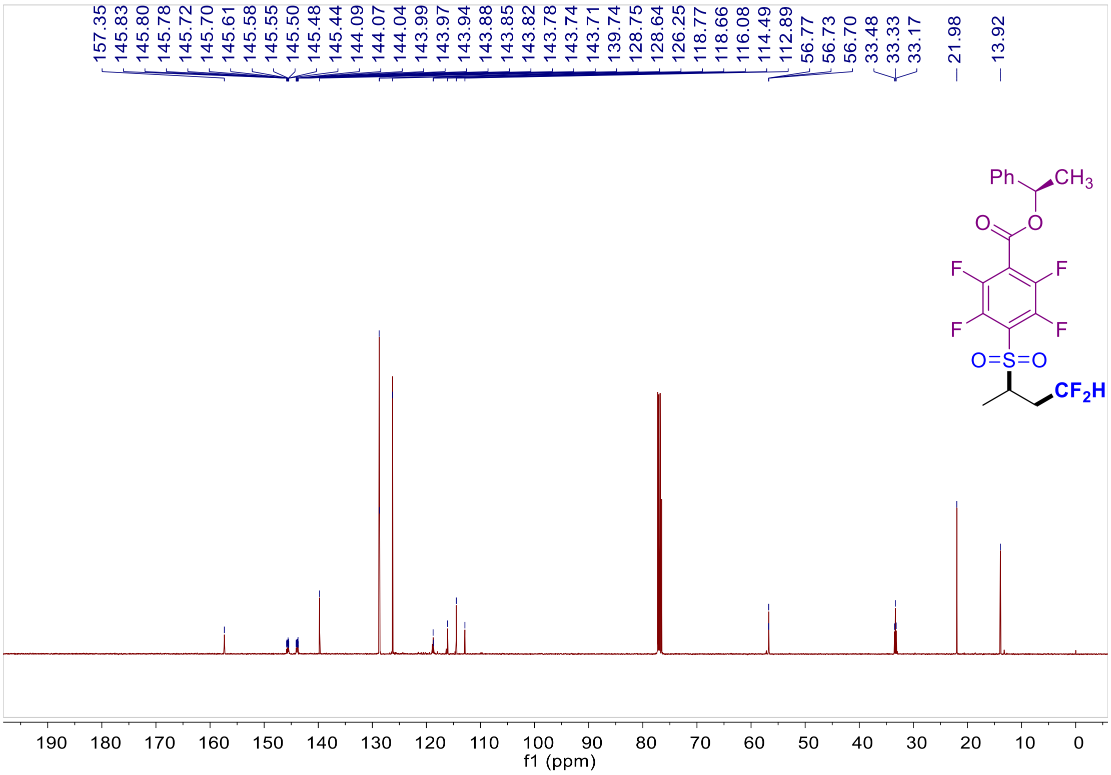

^{19}F NMR (565 MHz, $CDCl_3$) spectrum of 115

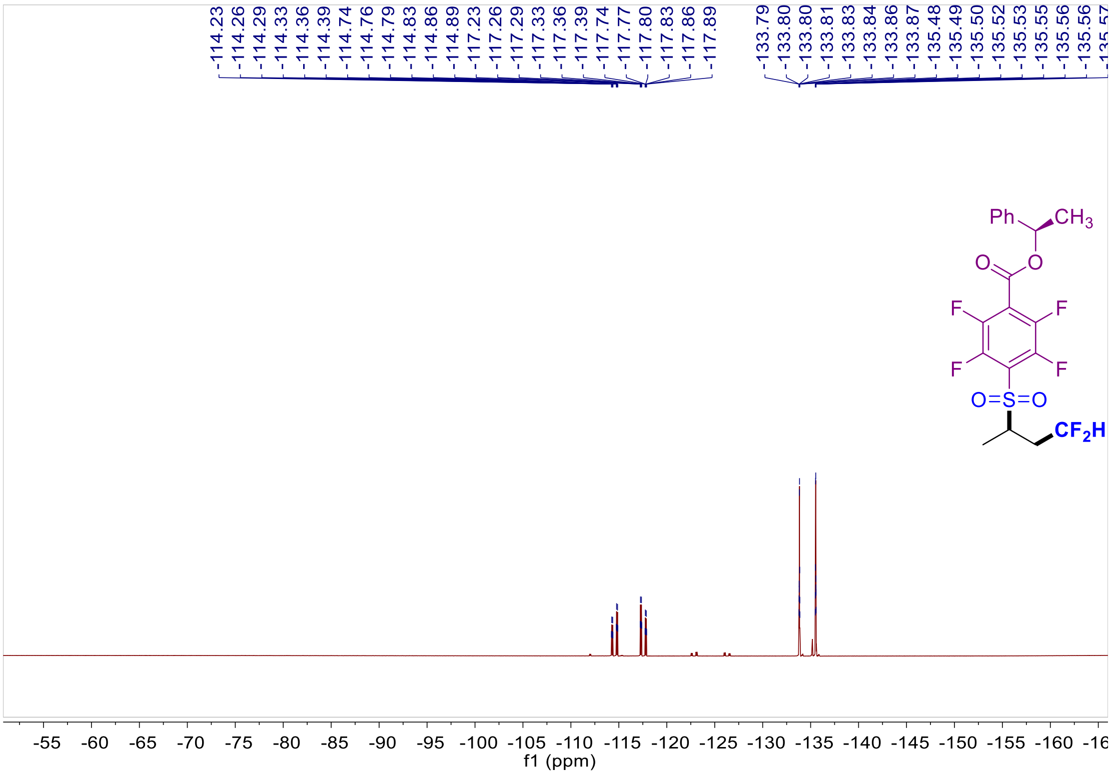

^{1}H NMR (600 MHz, $CDCl_3$) spectrum of 116

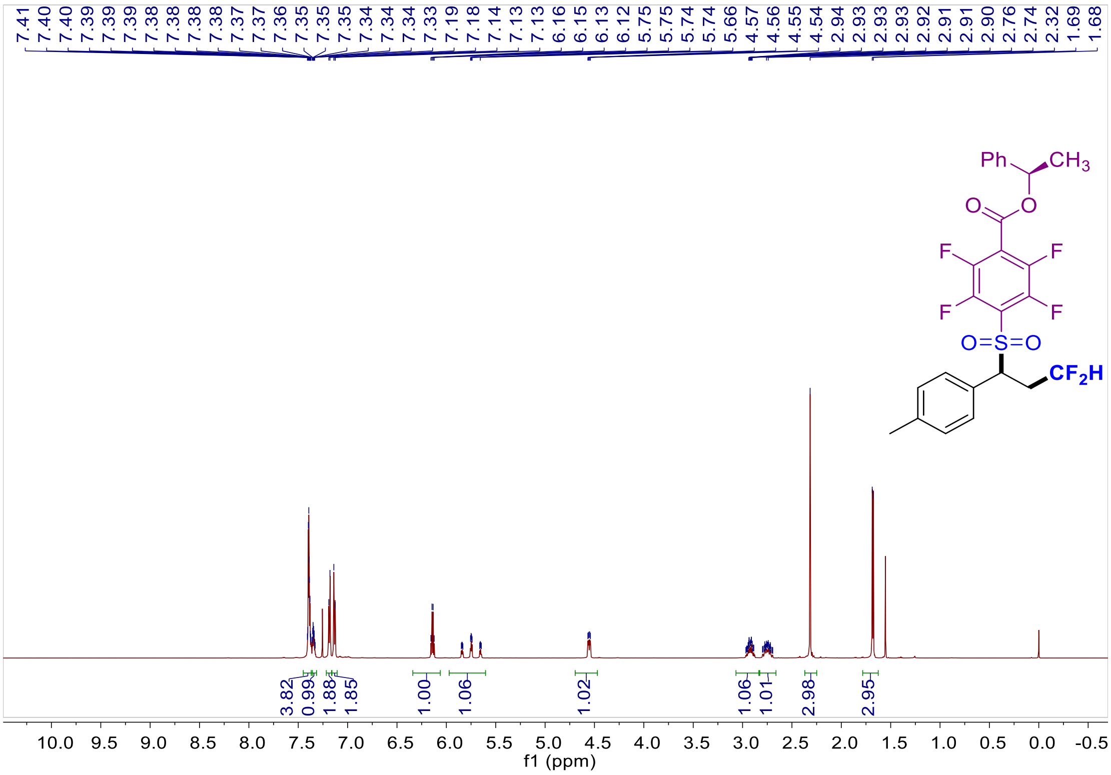

^{13}C NMR (151 MHz, $CDCl_3$) spectrum of 116

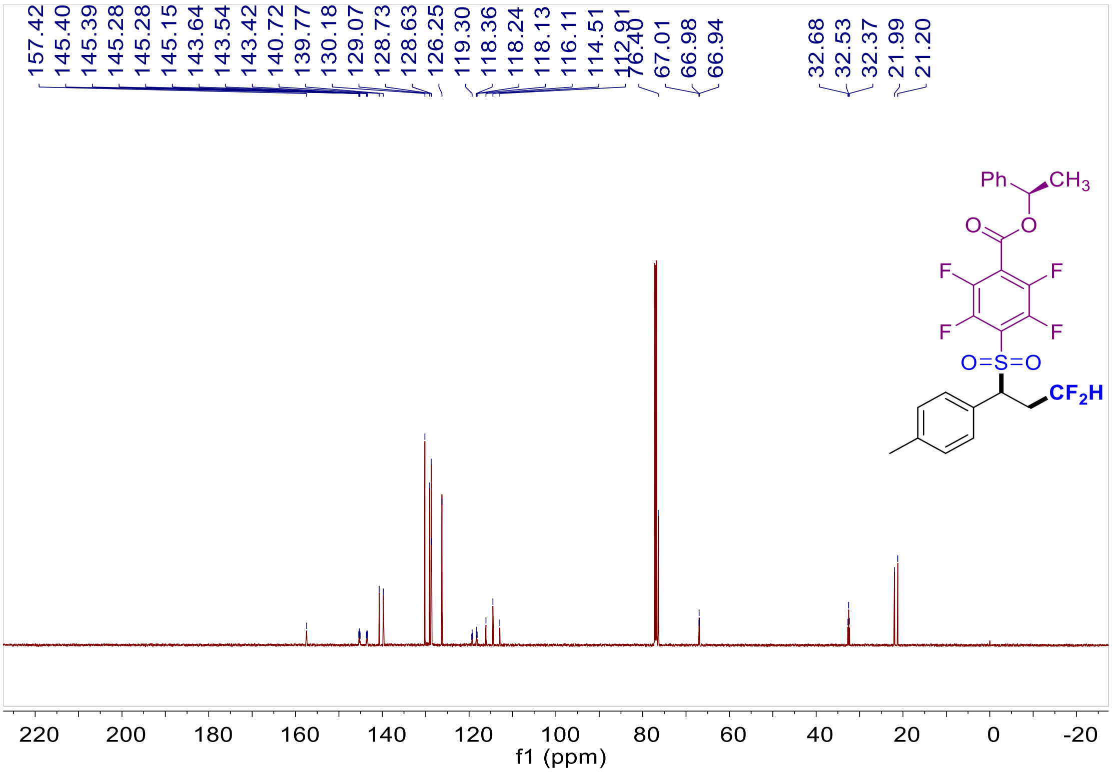

^{19}F NMR (565 MHz, CDCl₃) spectrum of 116

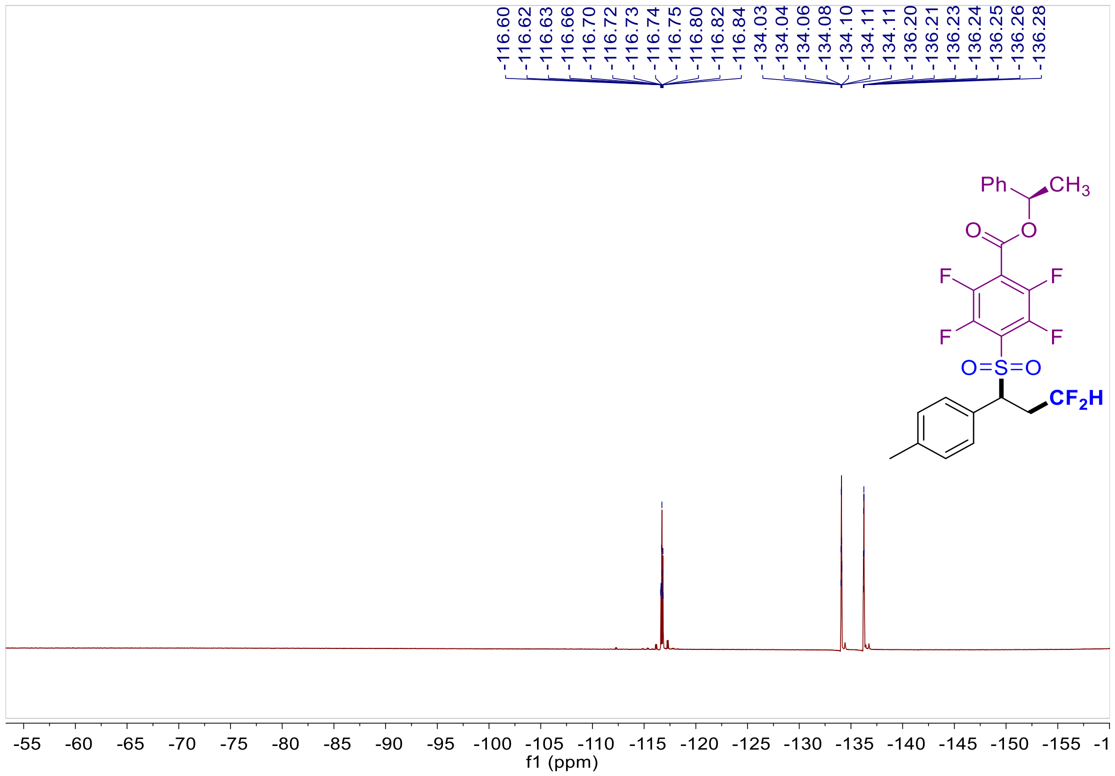

^{1}H NMR (600 MHz, CDCl₃) spectrum of 117

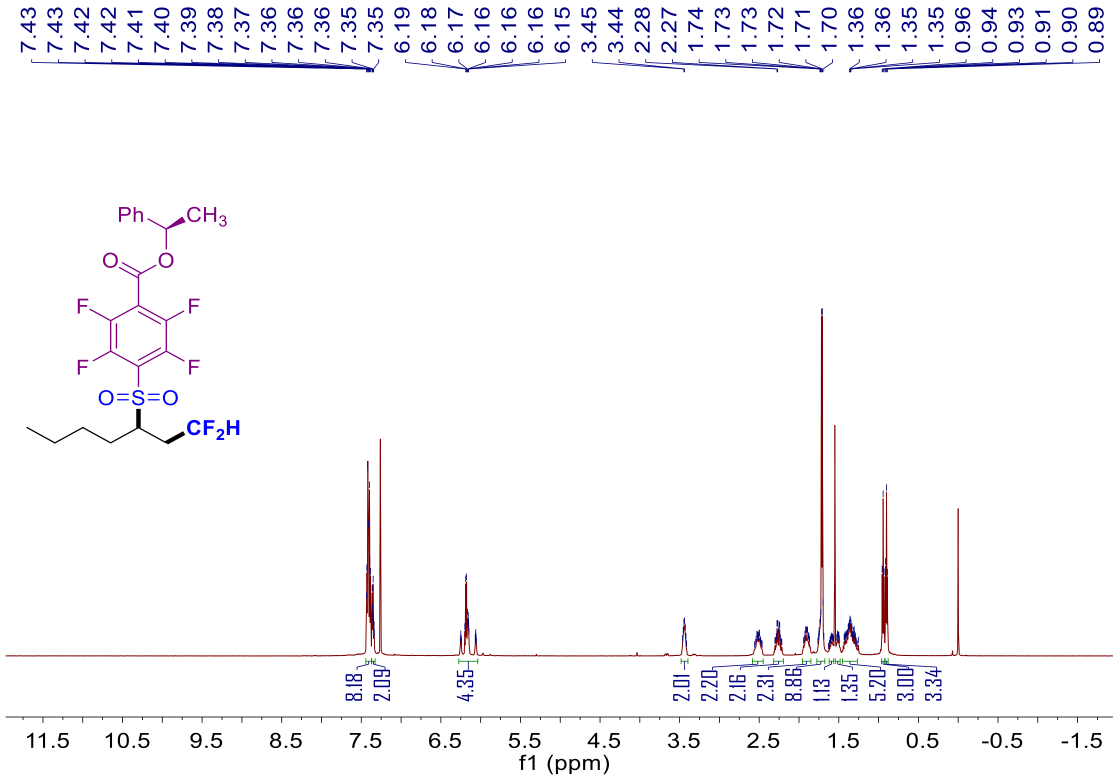

^{13}C NMR (151 MHz, $CDCl_3$) spectrum of 117

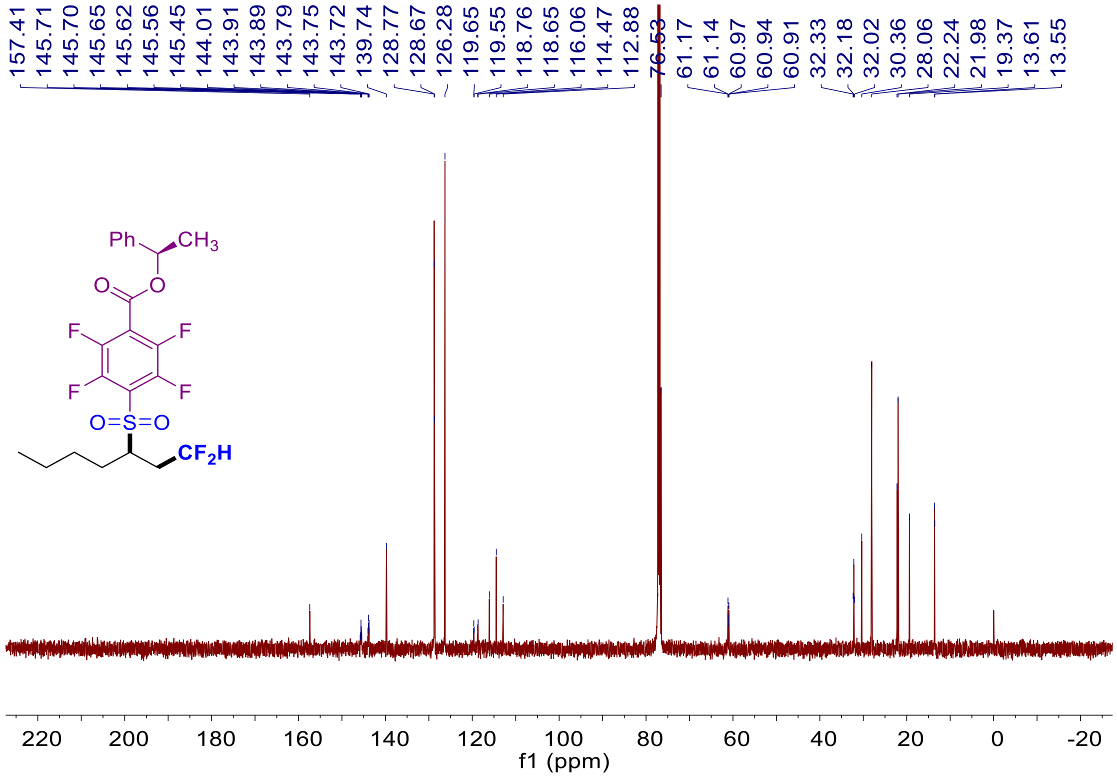

^{19}F NMR (565 MHz, $CDCl_3$) spectrum of 117

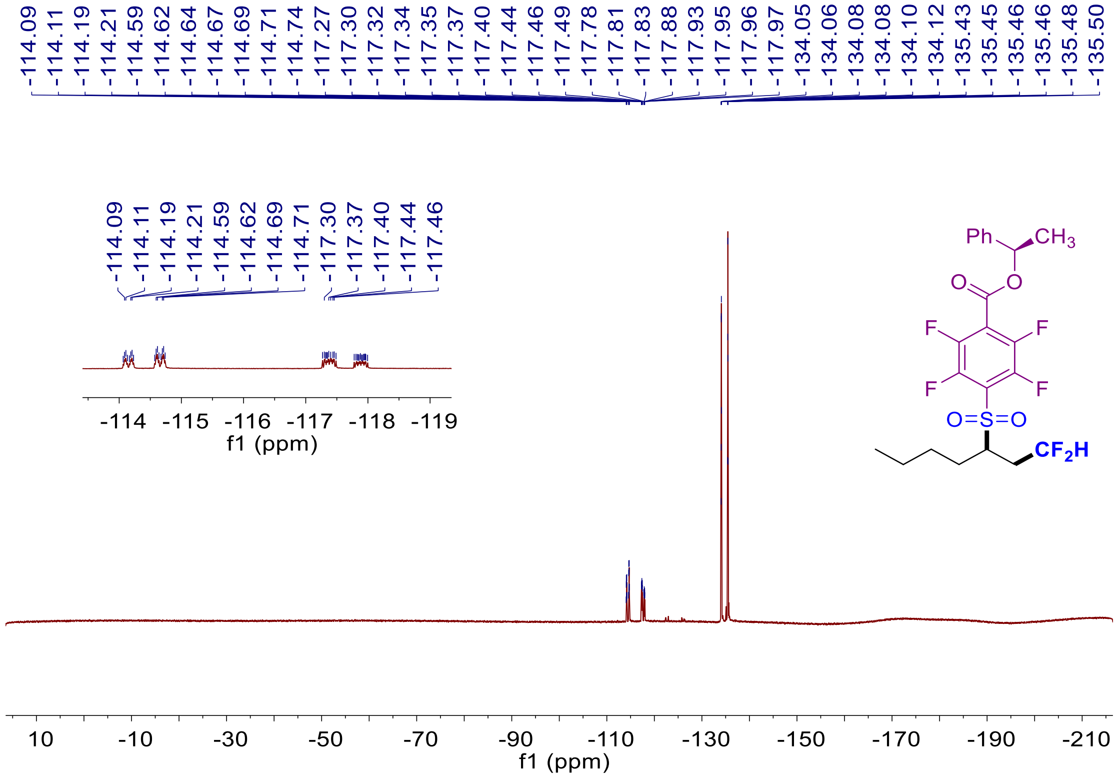

^{1}H NMR (600 MHz, CDCl$_3$) spectrum of 117'

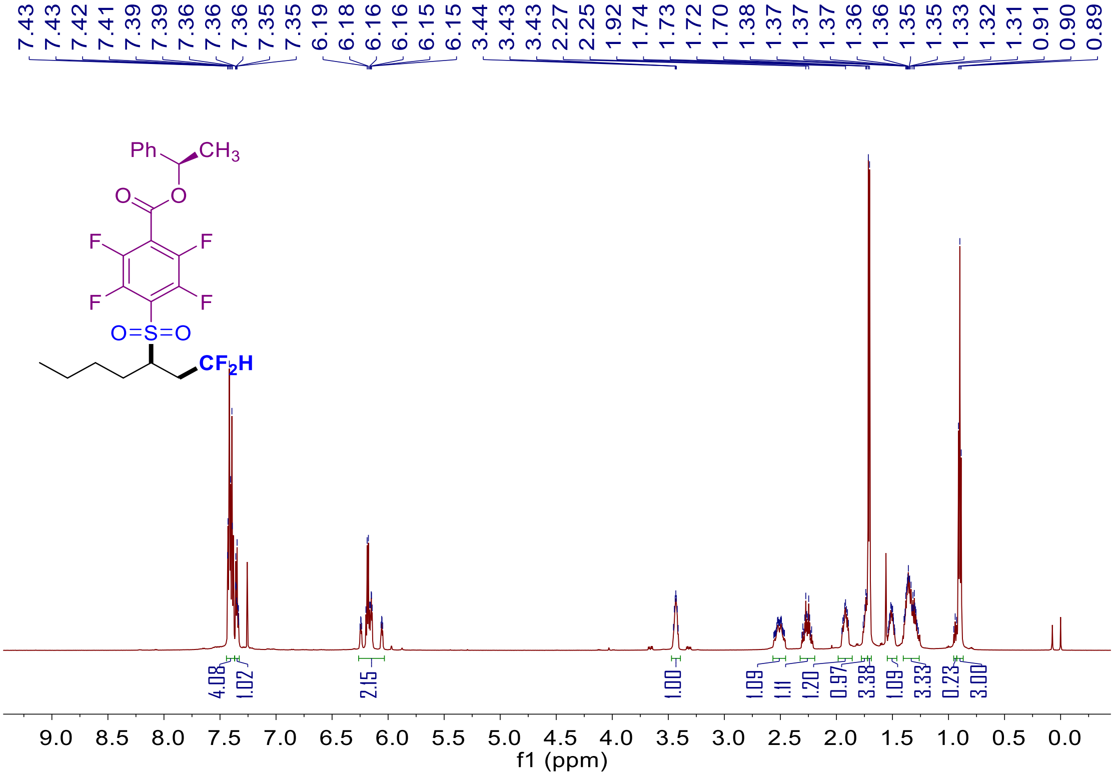

^{13}C NMR (151 MHz, CDCl$_3$) spectrum of 117'

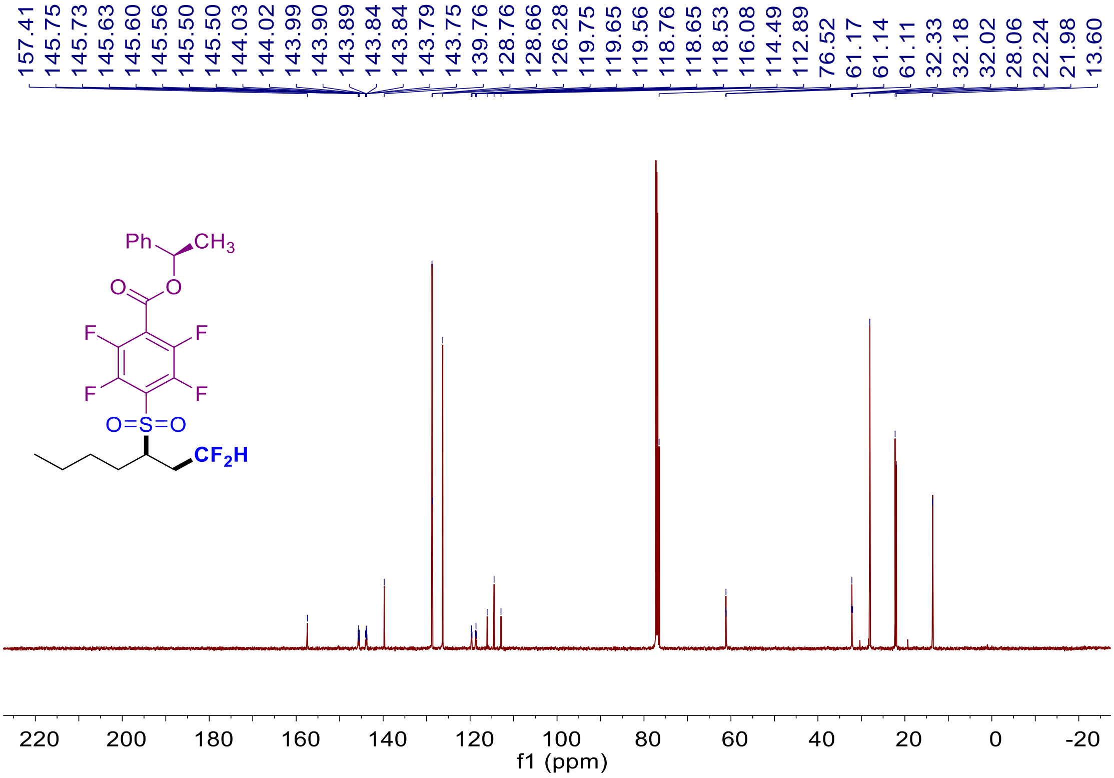

^{19}F NMR (565 MHz, CDCl3) spectrum of 117'

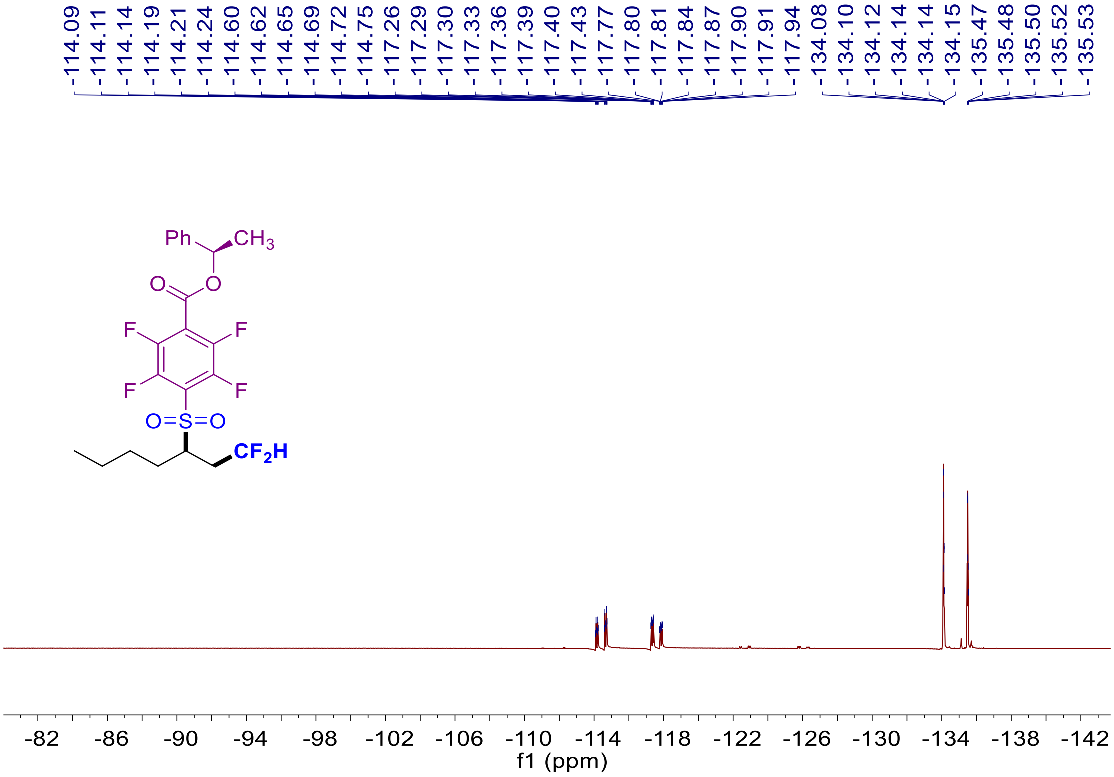

^{1}H NMR (600 MHz, CDCl3) spectrum of 118

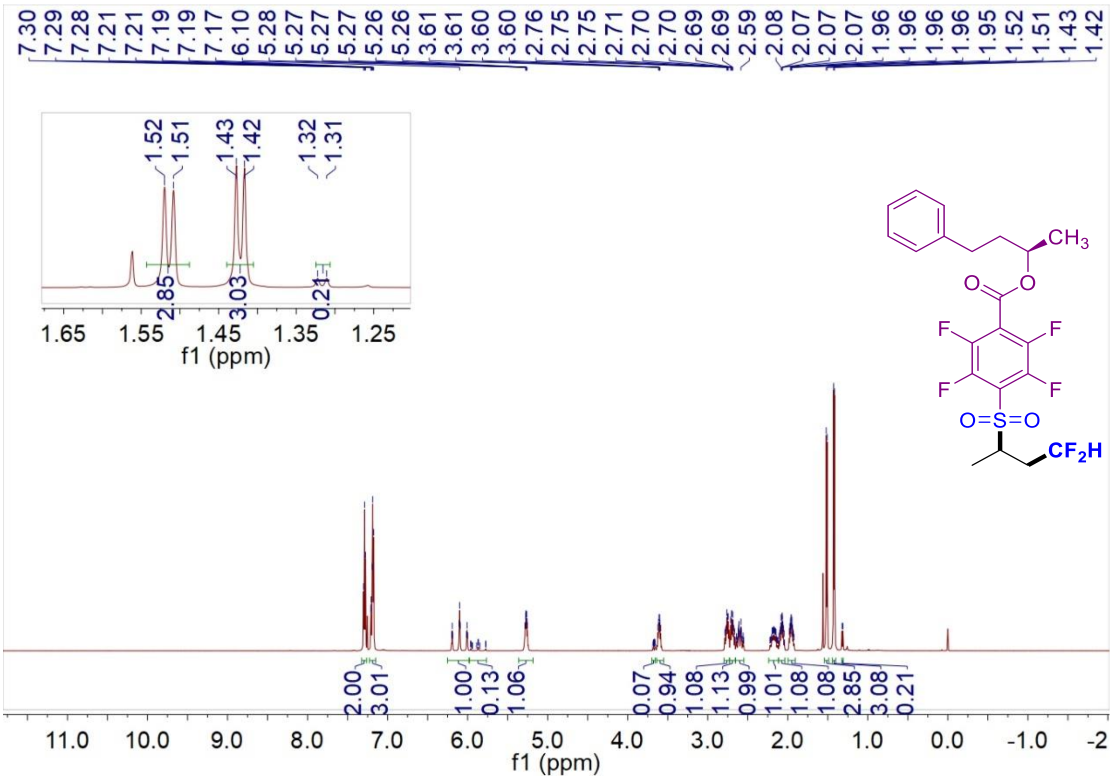

^{13}C NMR (151 MHz, $CDCl_3$) spectrum of 118

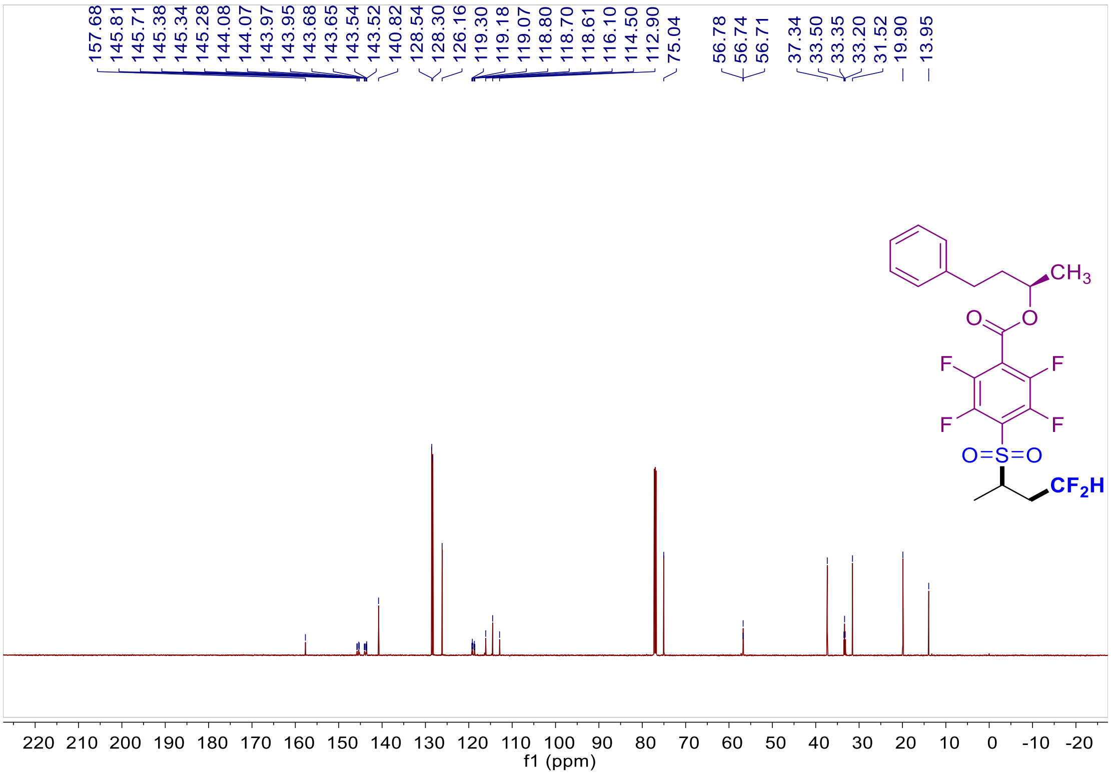

^{19}F NMR (565 MHz, $CDCl_3$) spectrum of 118

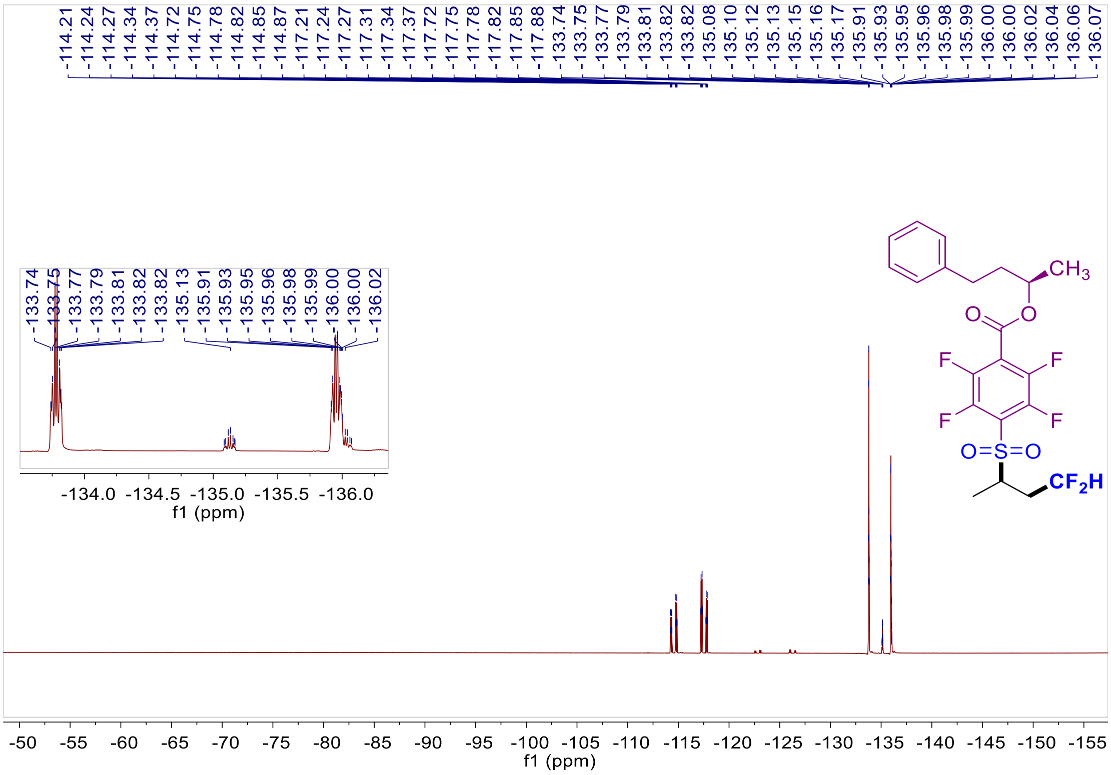

^{1}H NMR (600 MHz, $CDCl_3$) spectrum of 119

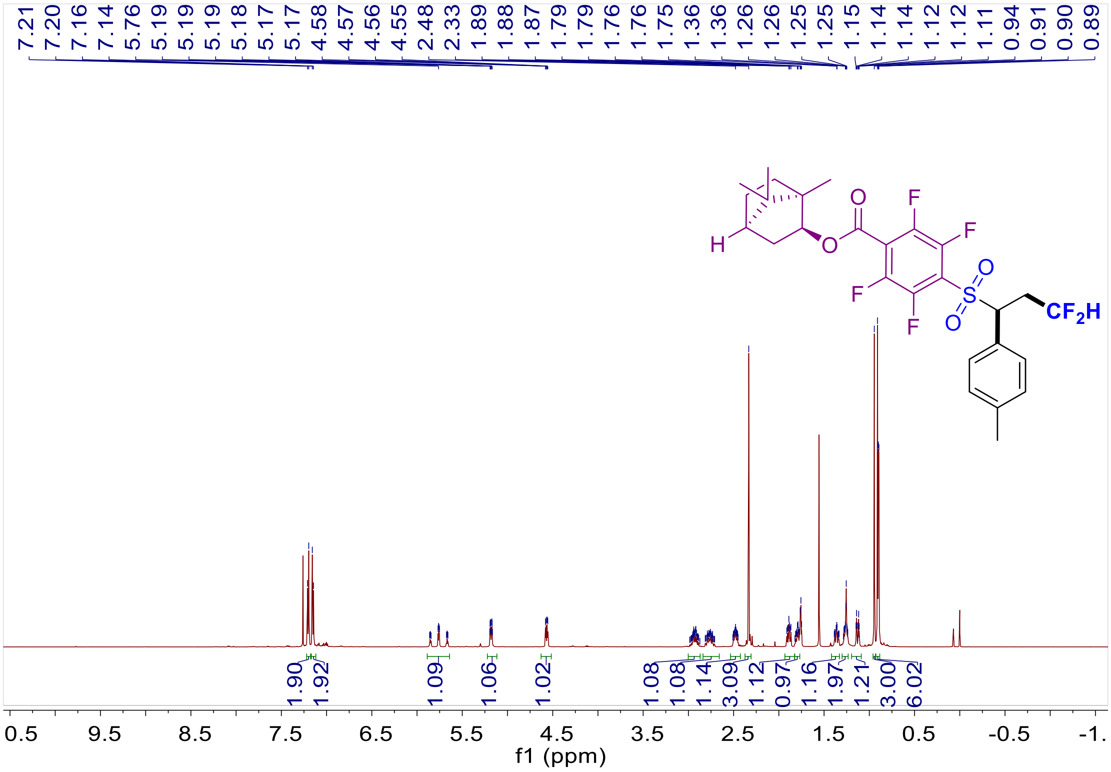

^{13}C NMR (151 MHz, $CDCl_3$) spectrum of 119

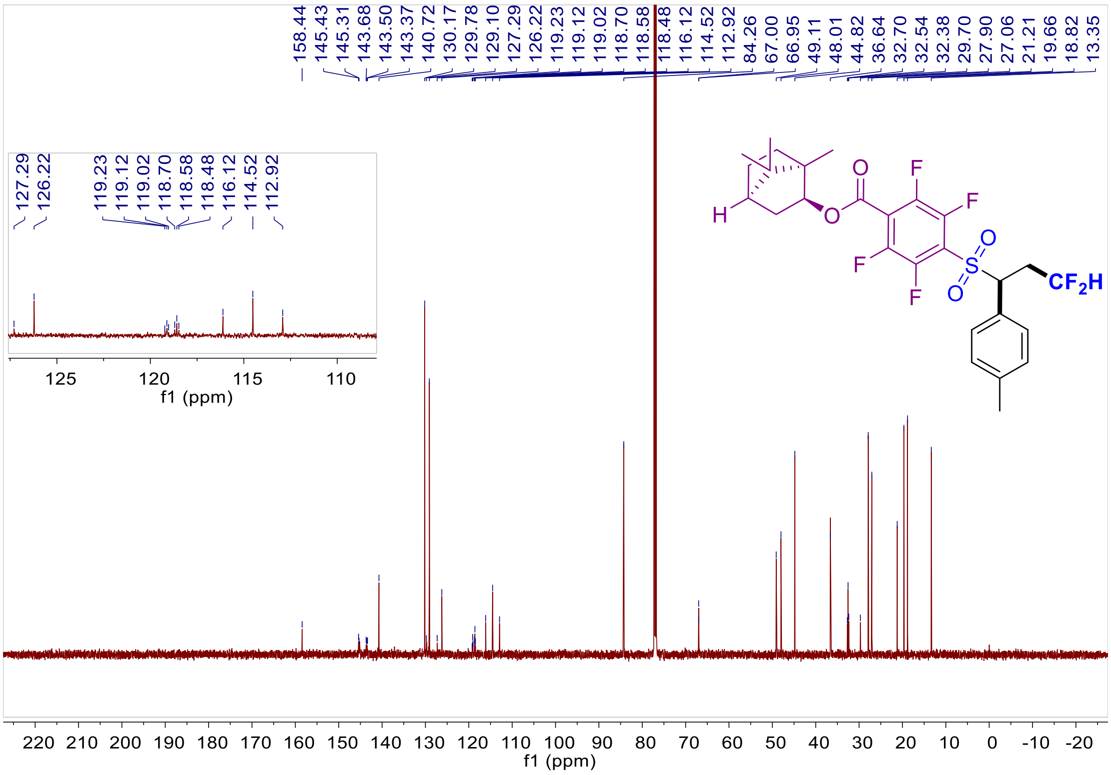

^{19}F NMR (565 MHz, CDCl$_3$) spectrum of 119

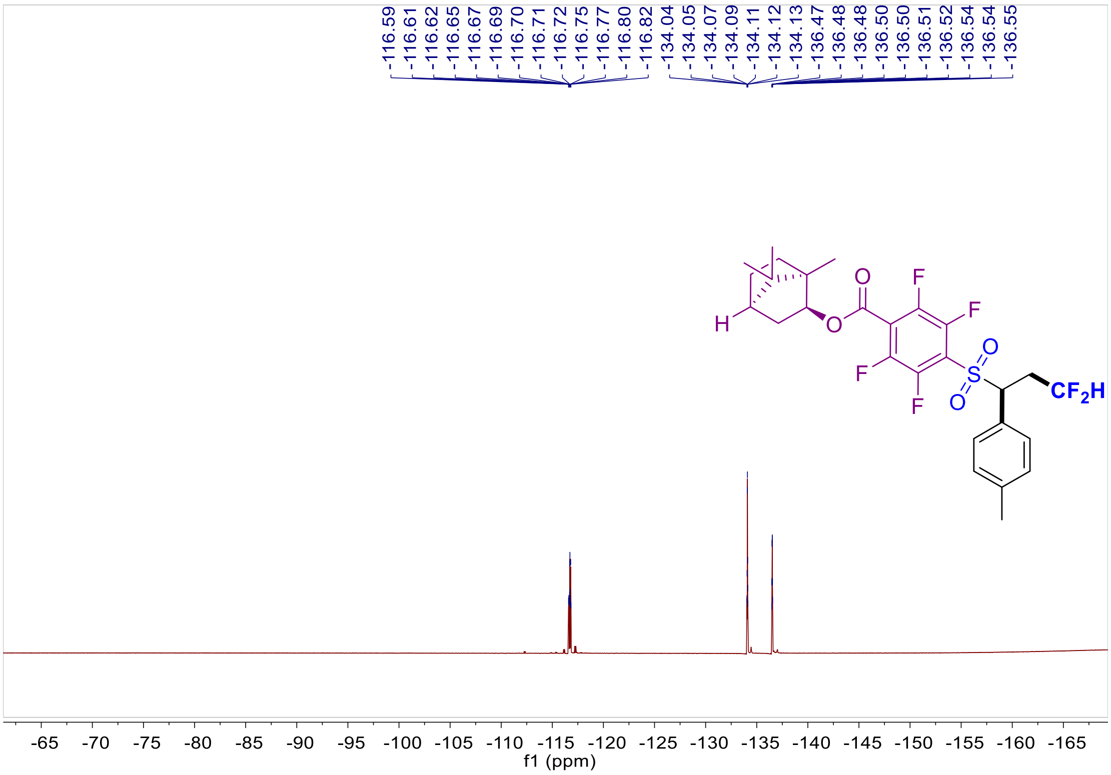

^{1}H NMR (600 MHz, CDCl$_3$) spectrum of 120

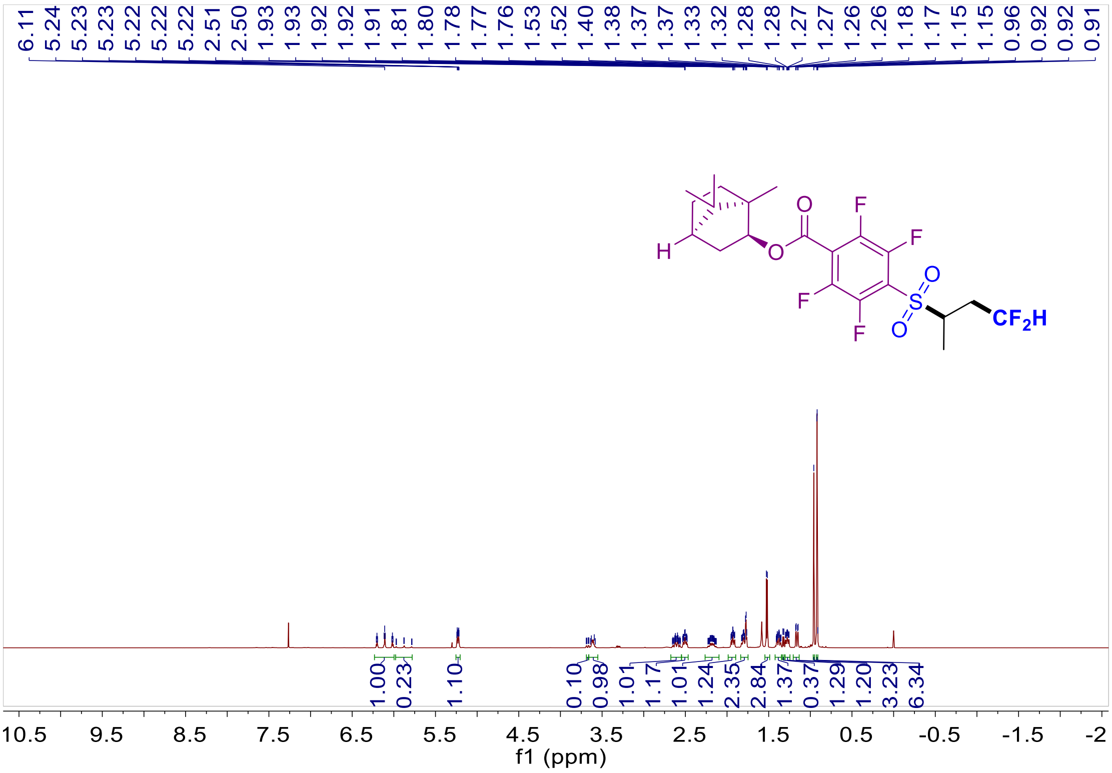

^{13}C NMR (151 MHz, CDCl$_3$) spectrum of 120

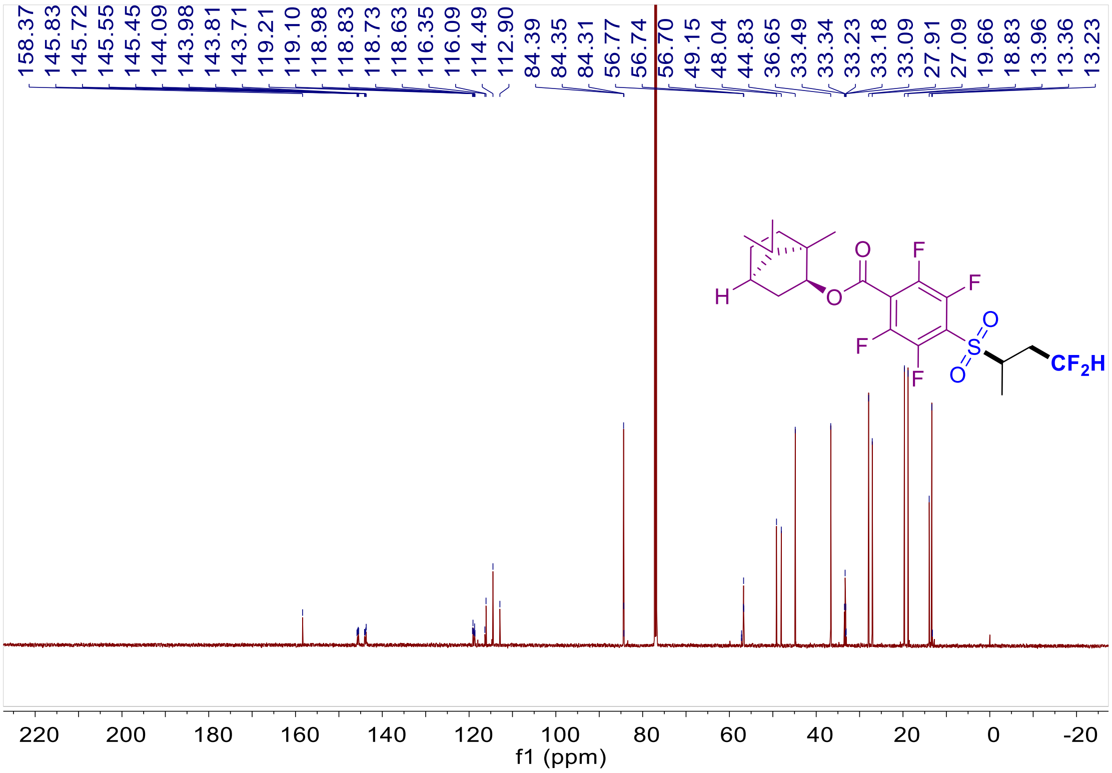

^{19}F NMR (565 MHz, CDCl$_3$) spectrum of 120

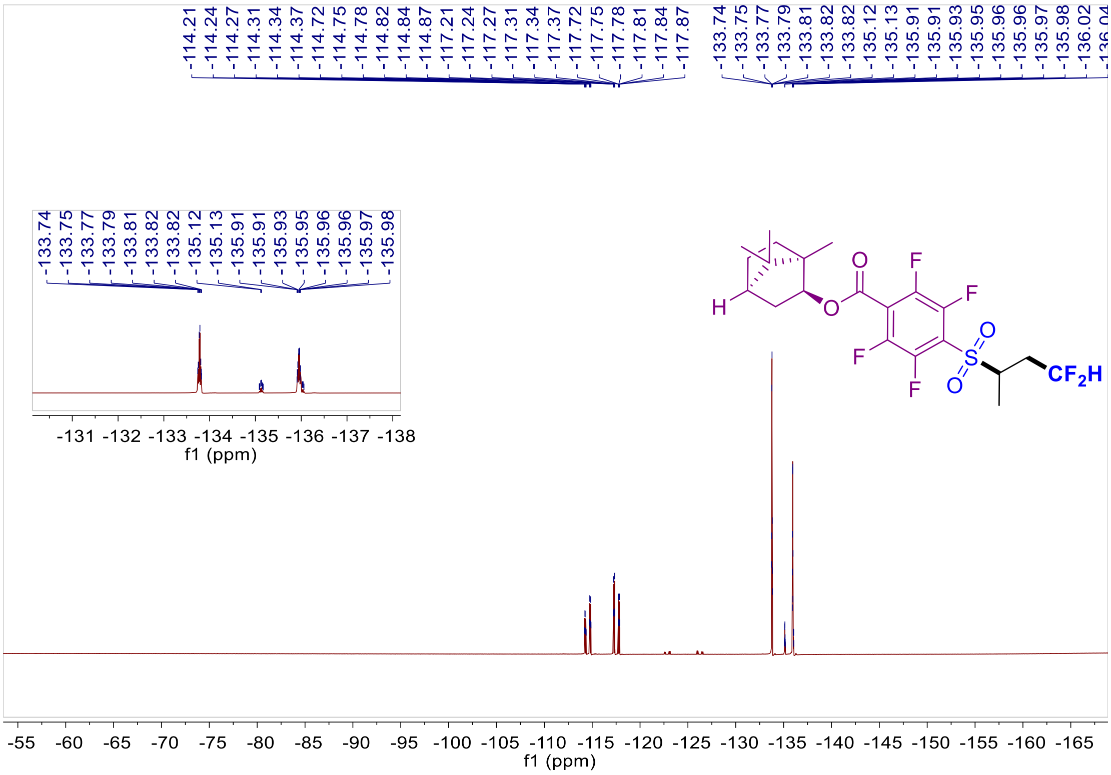

^{1}H NMR (600 MHz, CDCl3) spectrum of 121

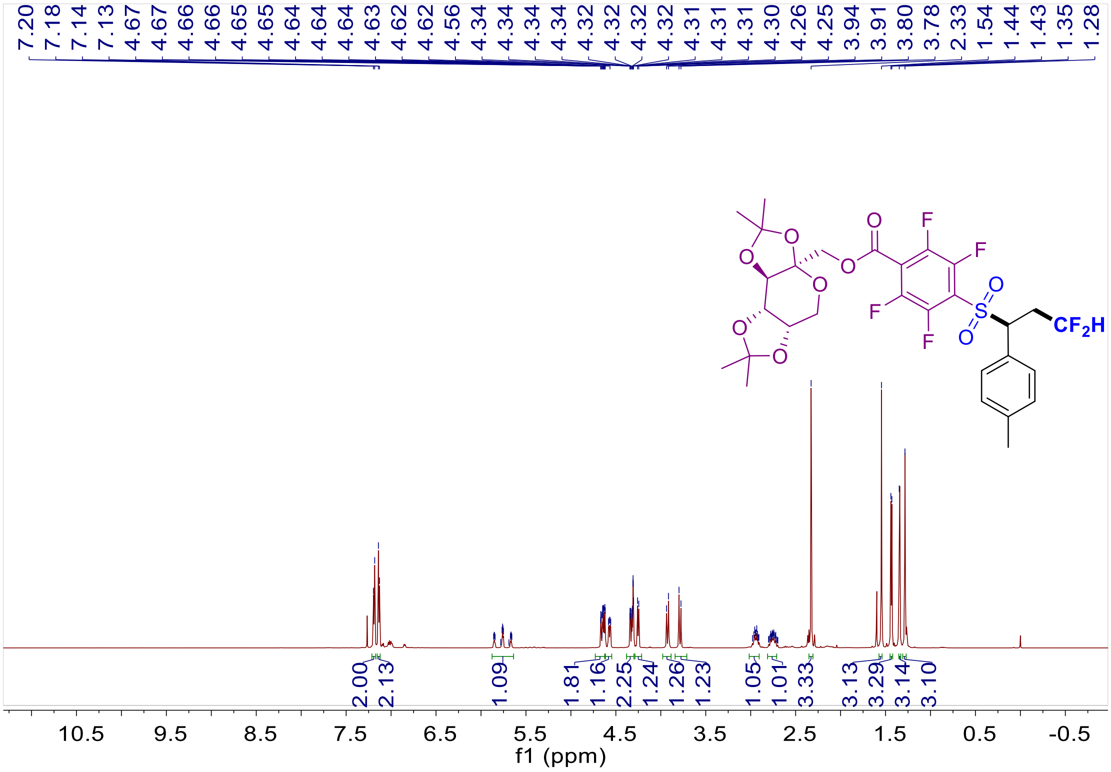

^{13}C NMR (151 MHz, CDCl3) spectrum of 121

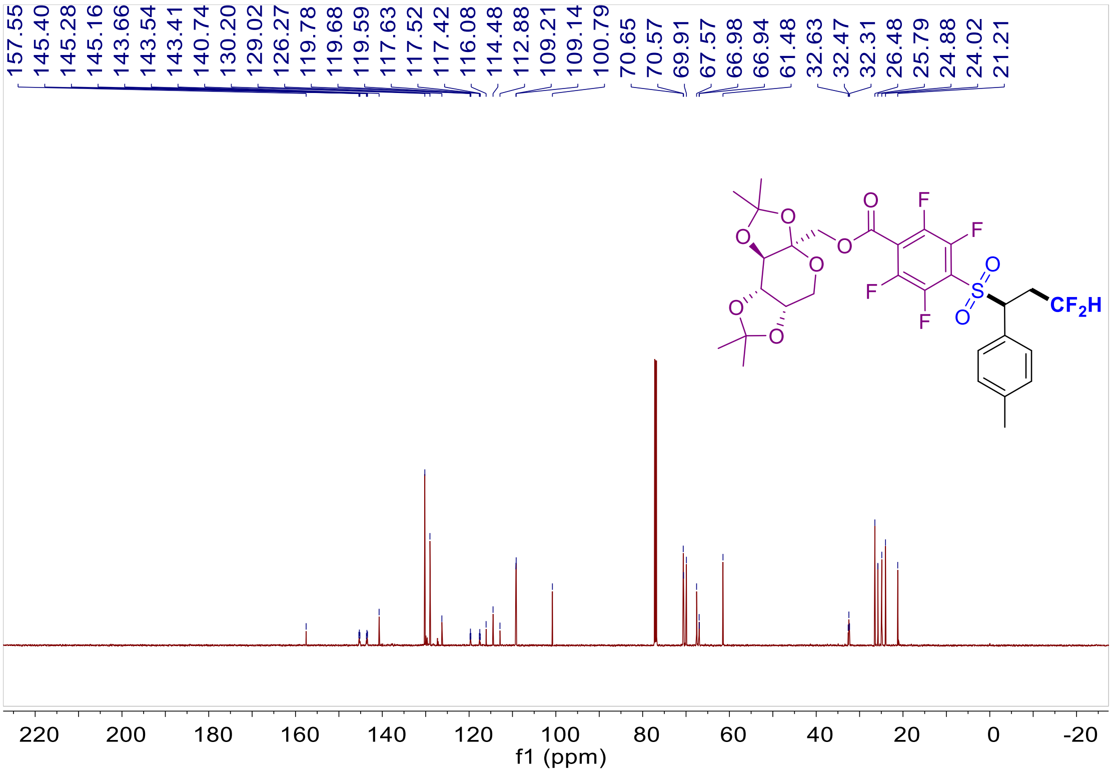

^{19}F NMR (565 MHz, CDCl$_3$) spectrum of 121

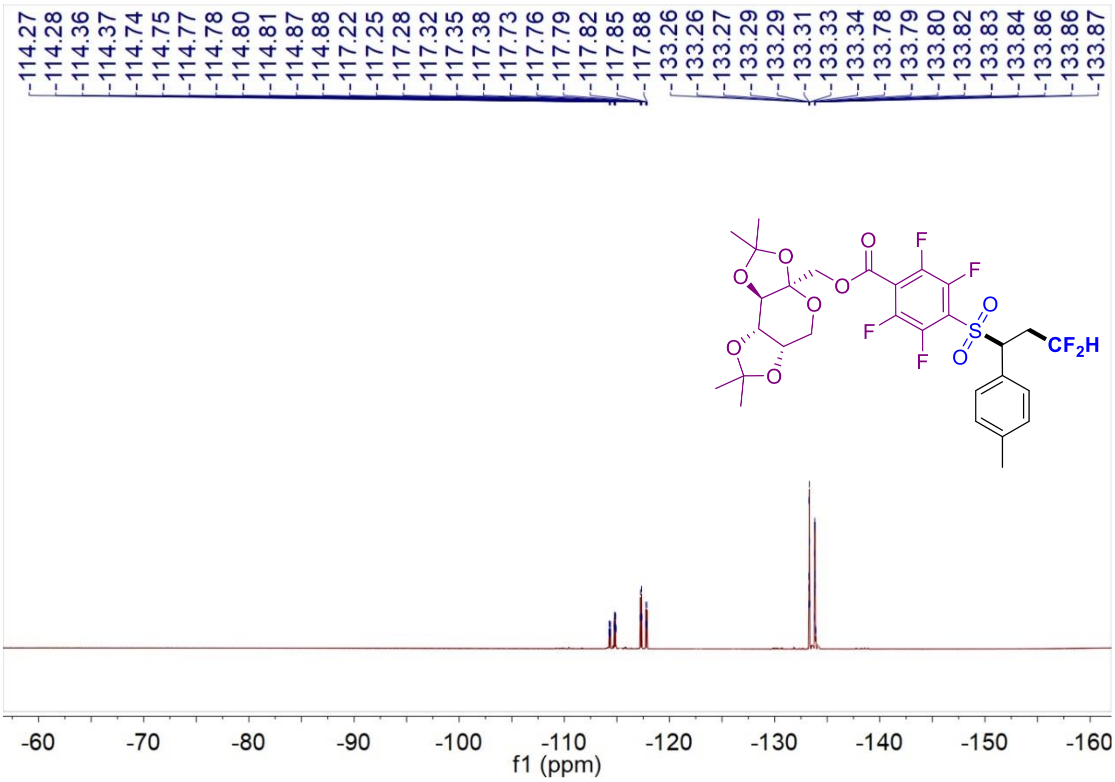

^{1}H NMR (600 MHz, CDCl$_3$) spectrum of 122

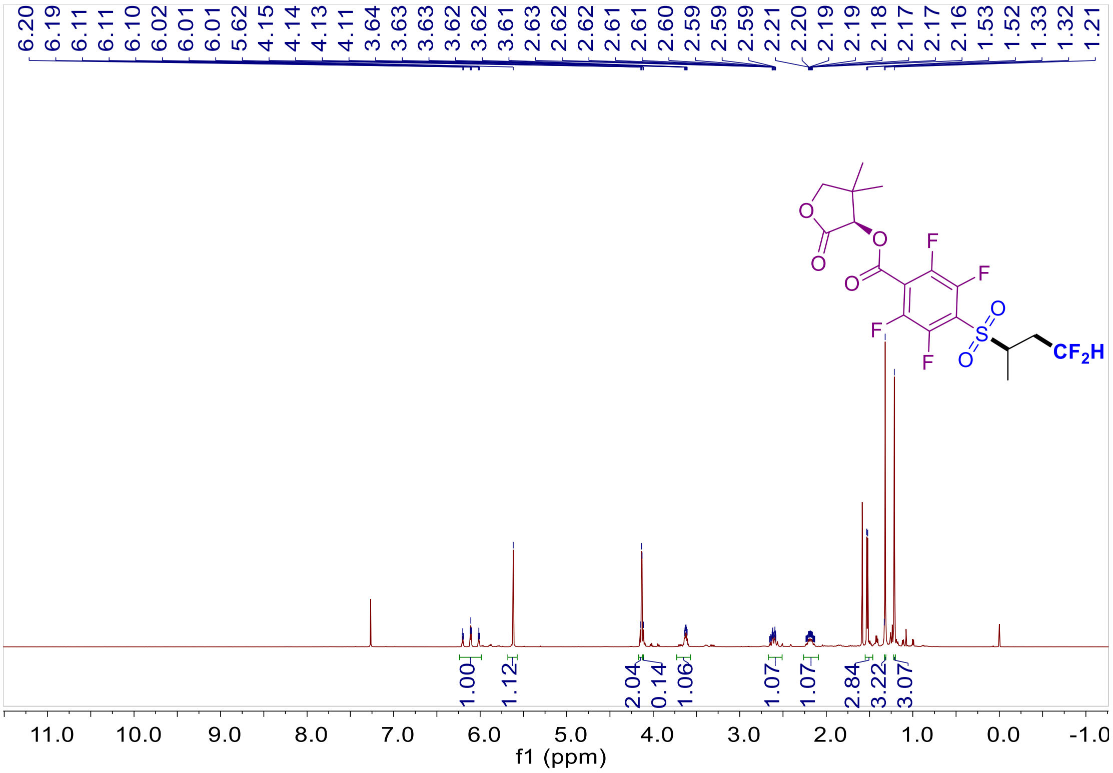

^{13}C NMR (151 MHz, $CDCl_3$) spectrum of 122

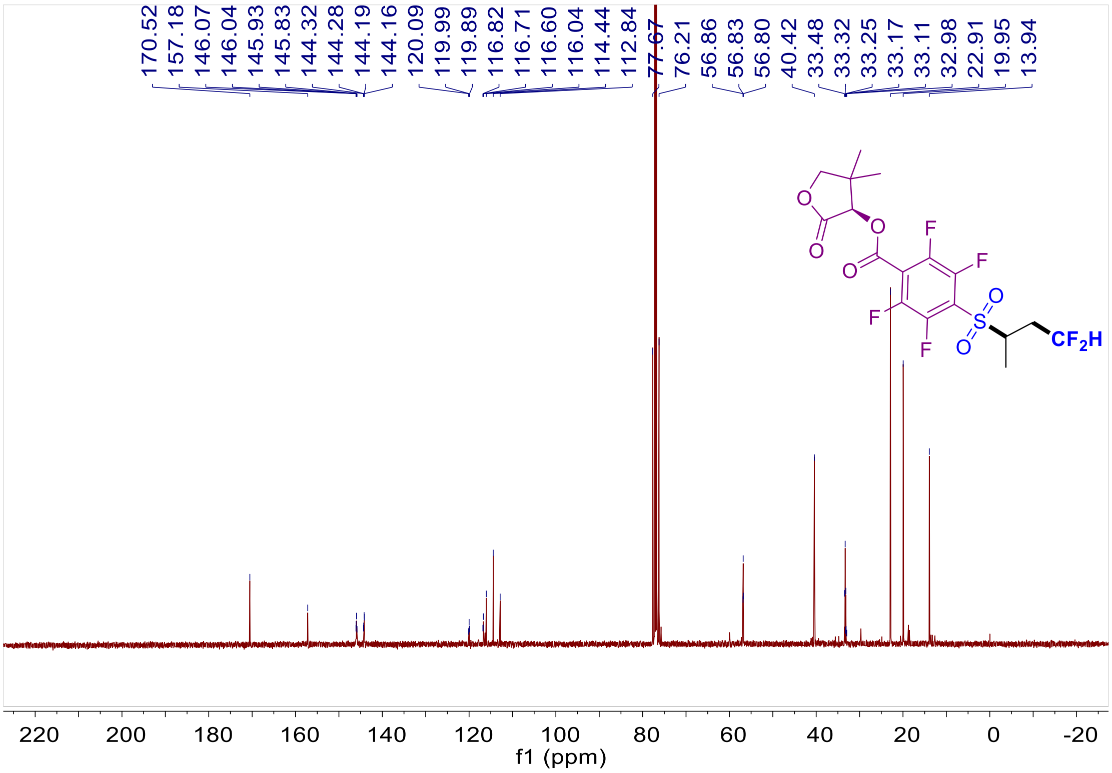

^{19}F NMR (565 MHz, $CDCl_3$) spectrum of 122

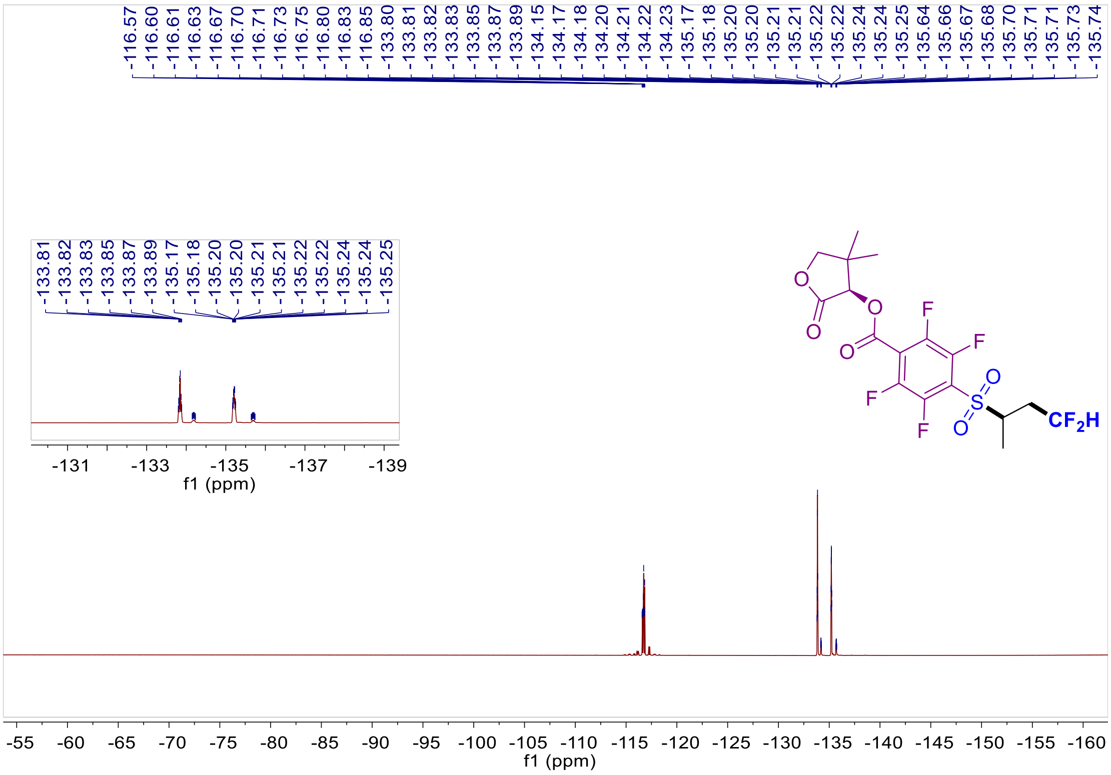

^{1}H NMR (600 MHz, $CDCl_3$) spectrum of 123

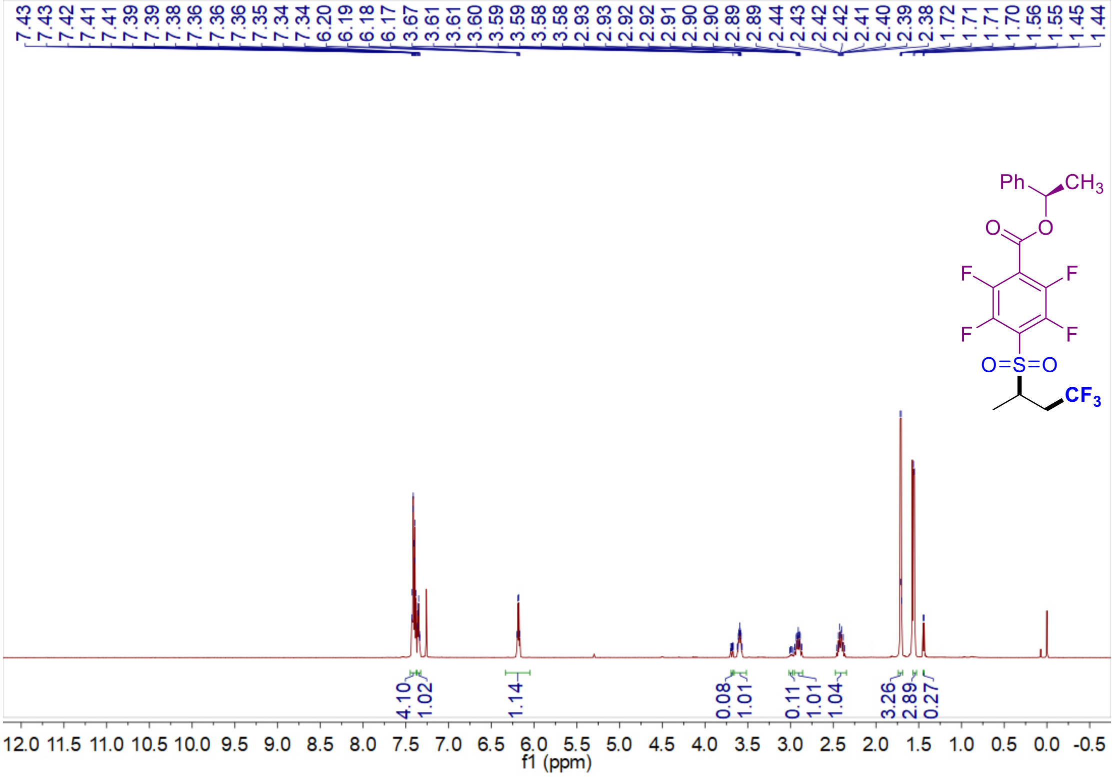

^{13}C NMR (151 MHz, $CDCl_3$) spectrum of 123

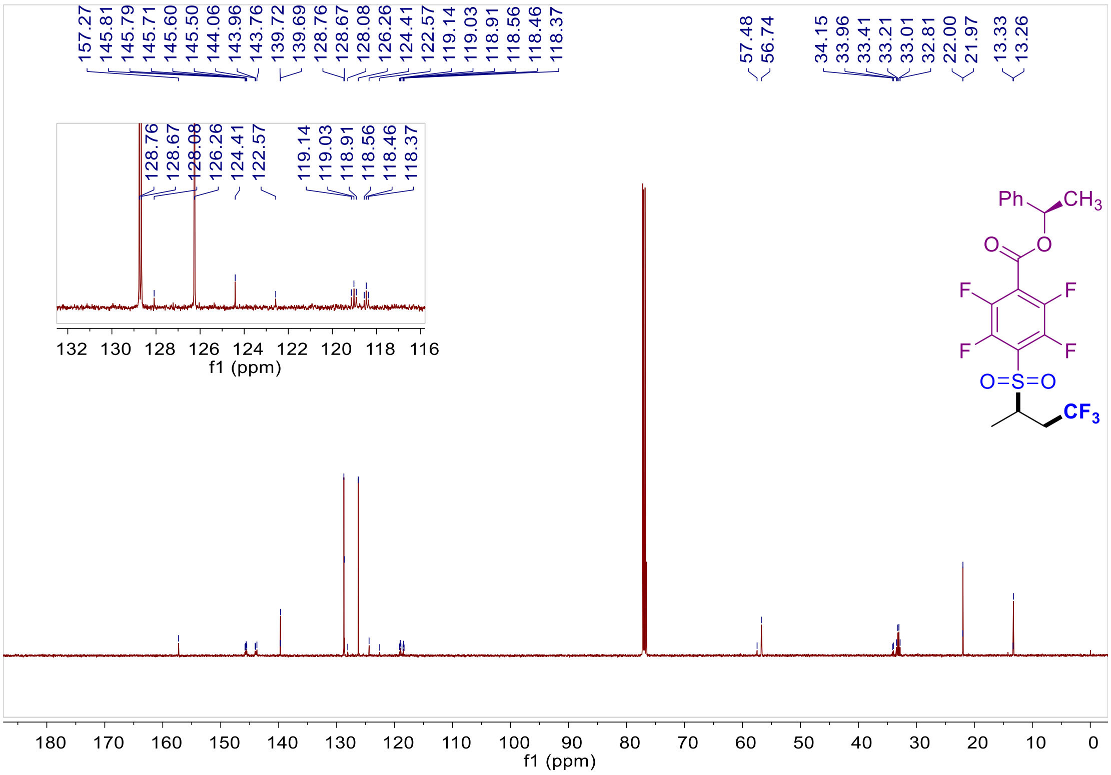

^{19}F NMR (565 MHz, $CDCl_3$) spectrum of 123

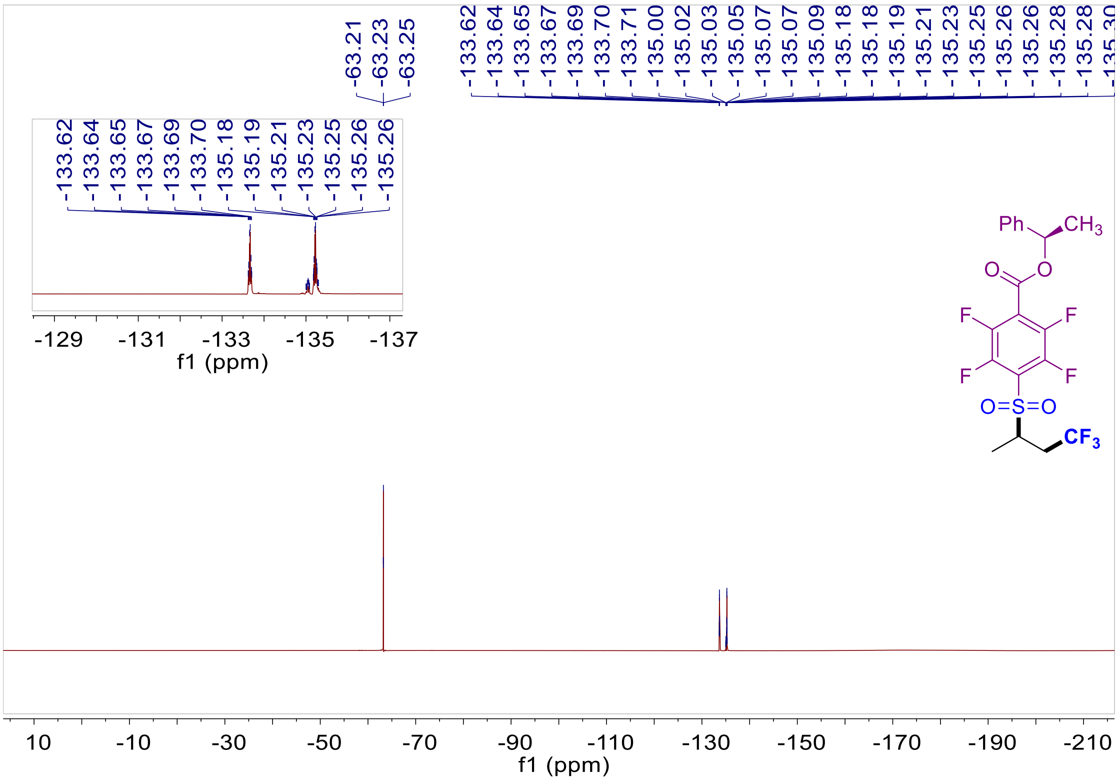

^{1}H NMR (600 MHz, $CDCl_3$) spectrum of 124

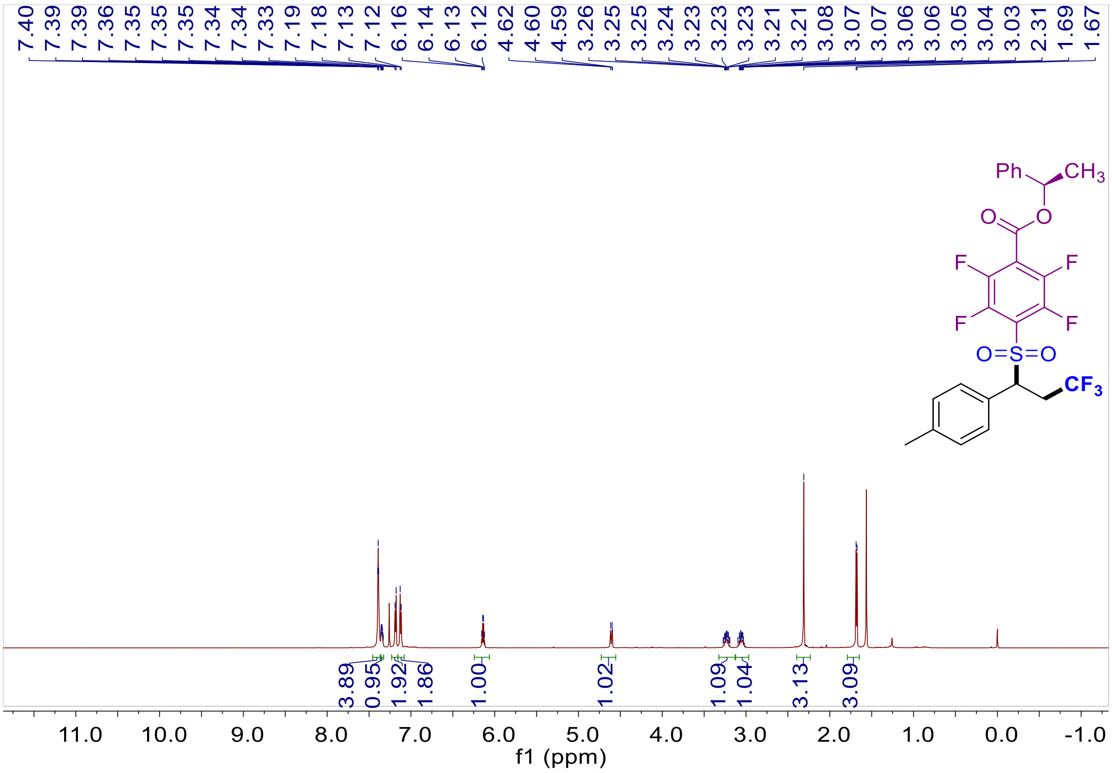

^{13}C NMR (151 MHz, $CDCl_3$) spectrum of 124

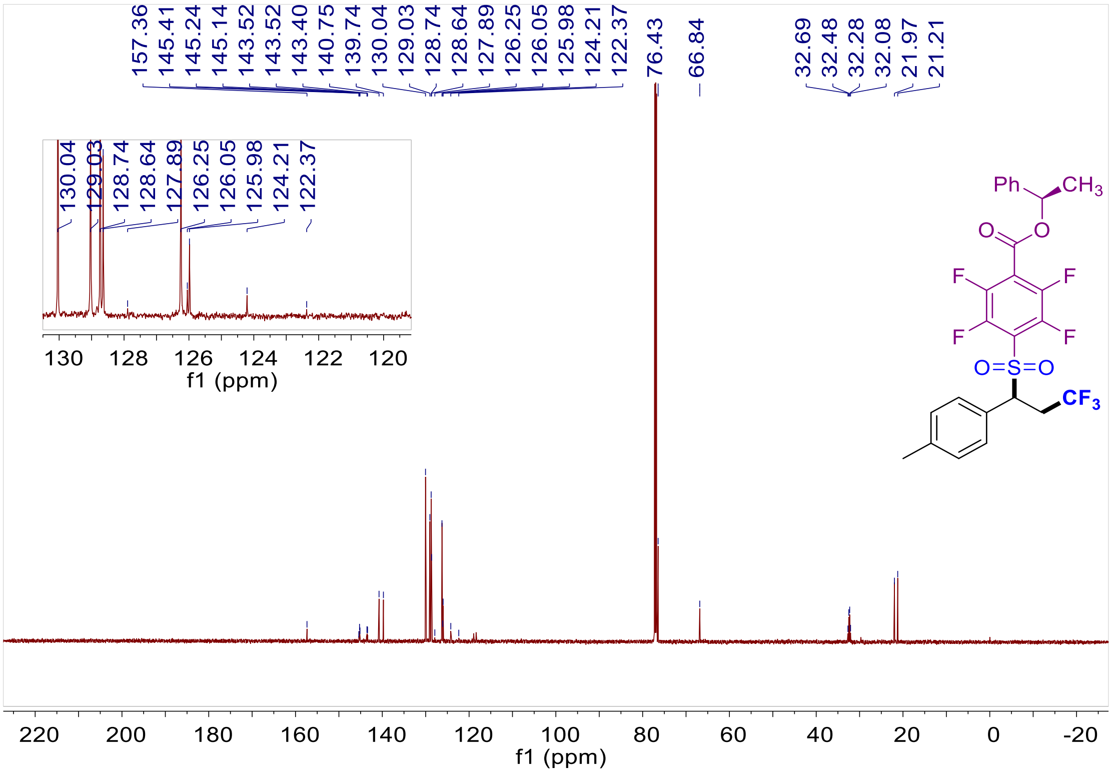

^{19}F NMR (565 MHz, $CDCl_3$) spectrum of 124

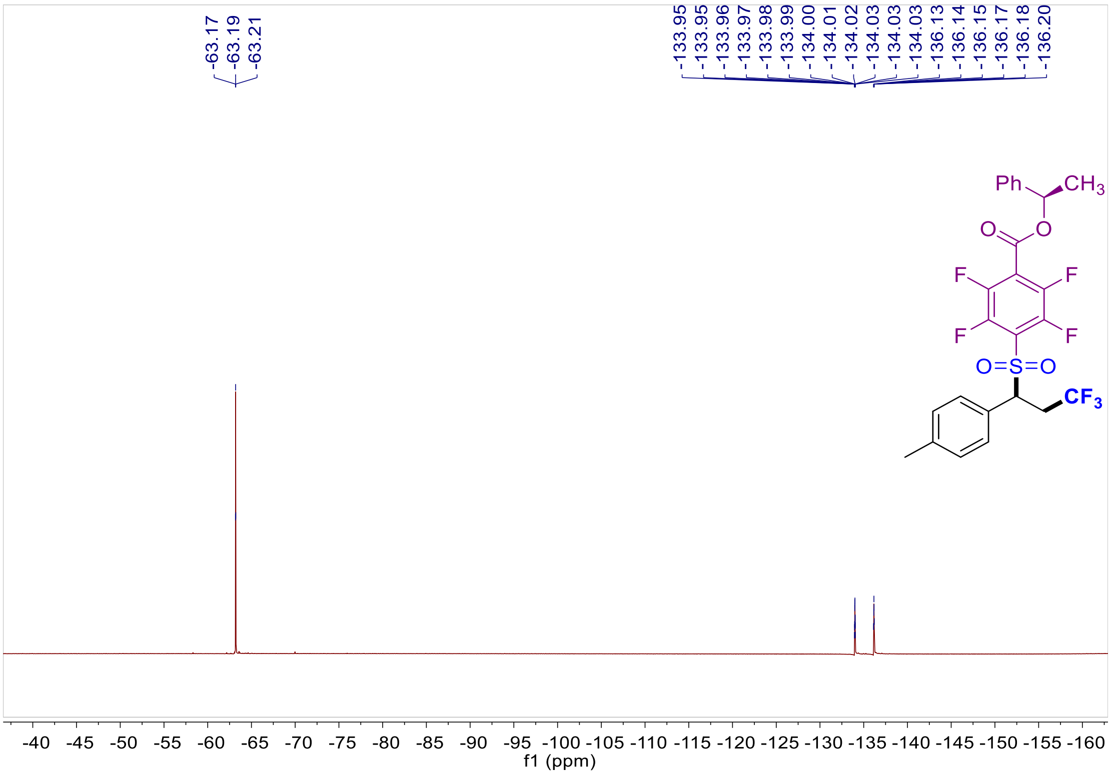

^{1}H NMR (500 MHz, CDCl$_3$) spectrum of 127

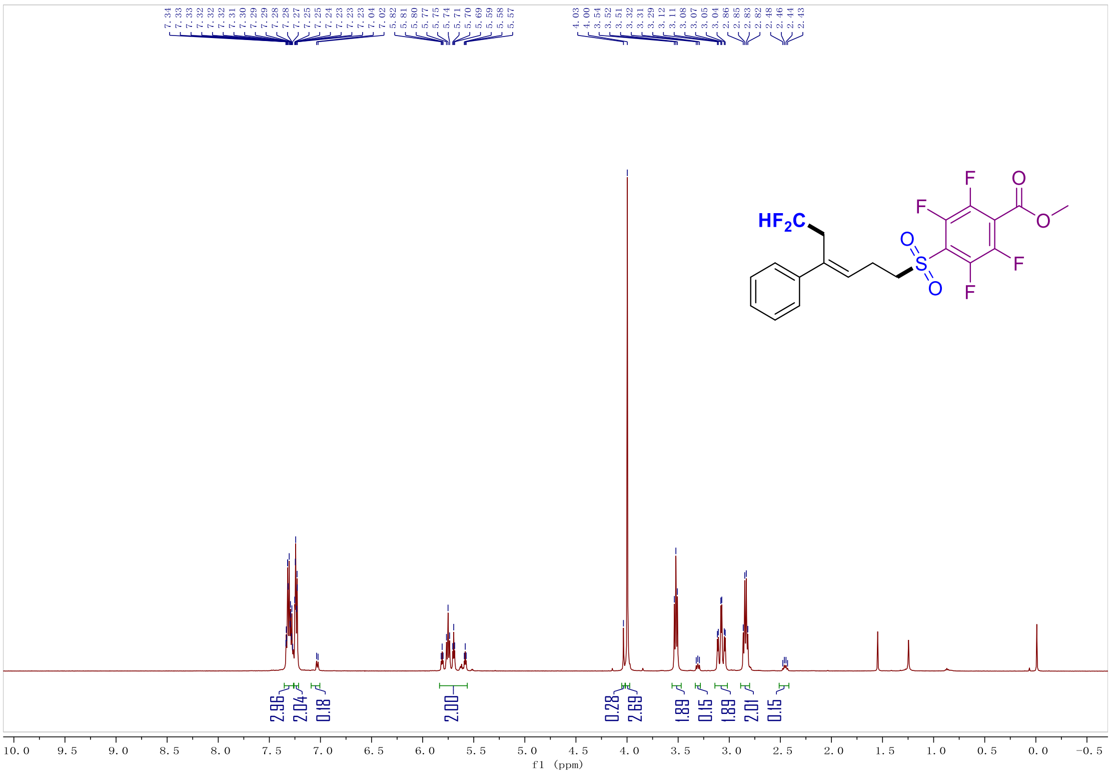

^{13}C NMR (151 MHz, CDCl$_3$) spectrum of 127

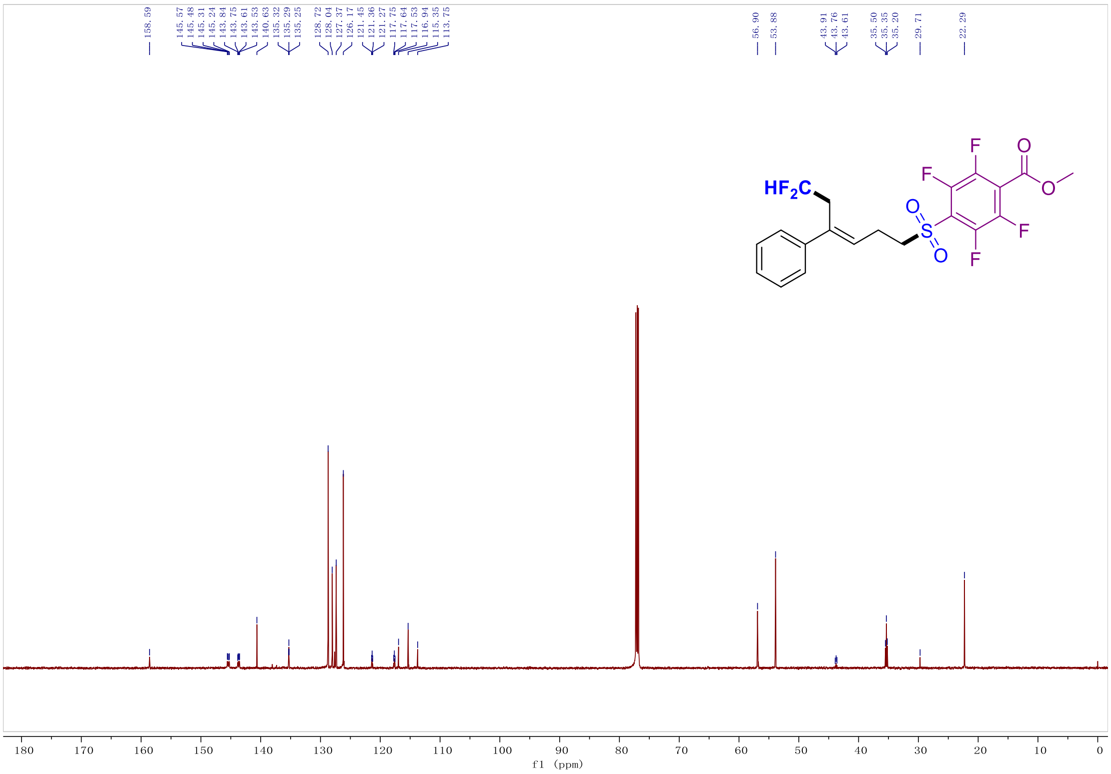

^{19}F NMR (565 MHz, CDCl$_3$) spectrum of 127

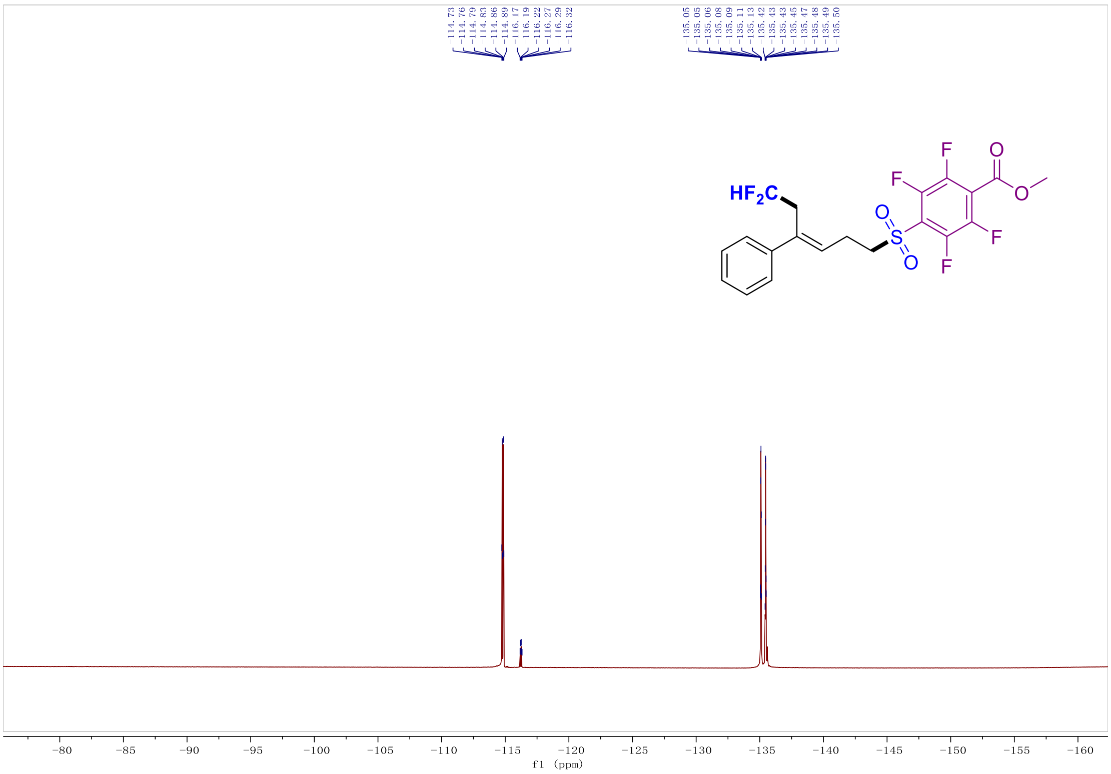

^{1}H NMR (500 MHz, CDCl$_3$) spectrum of 128

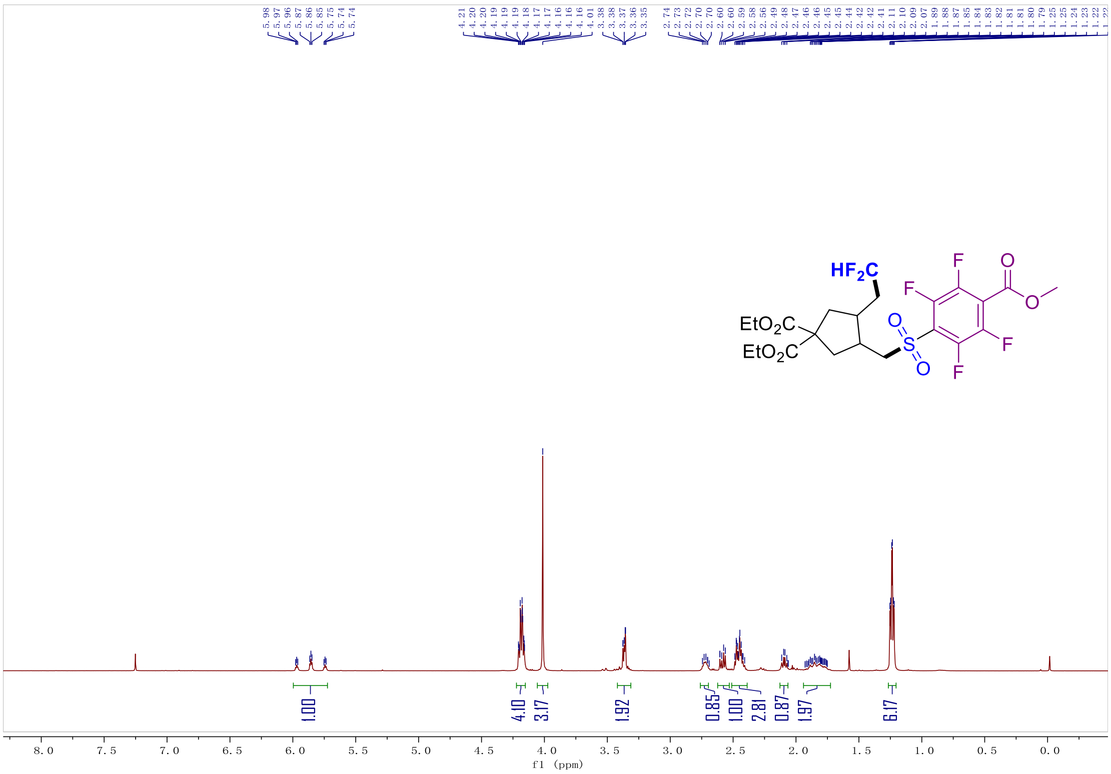

^{13}C NMR (151 MHz, $CDCl_3$) spectrum of 128

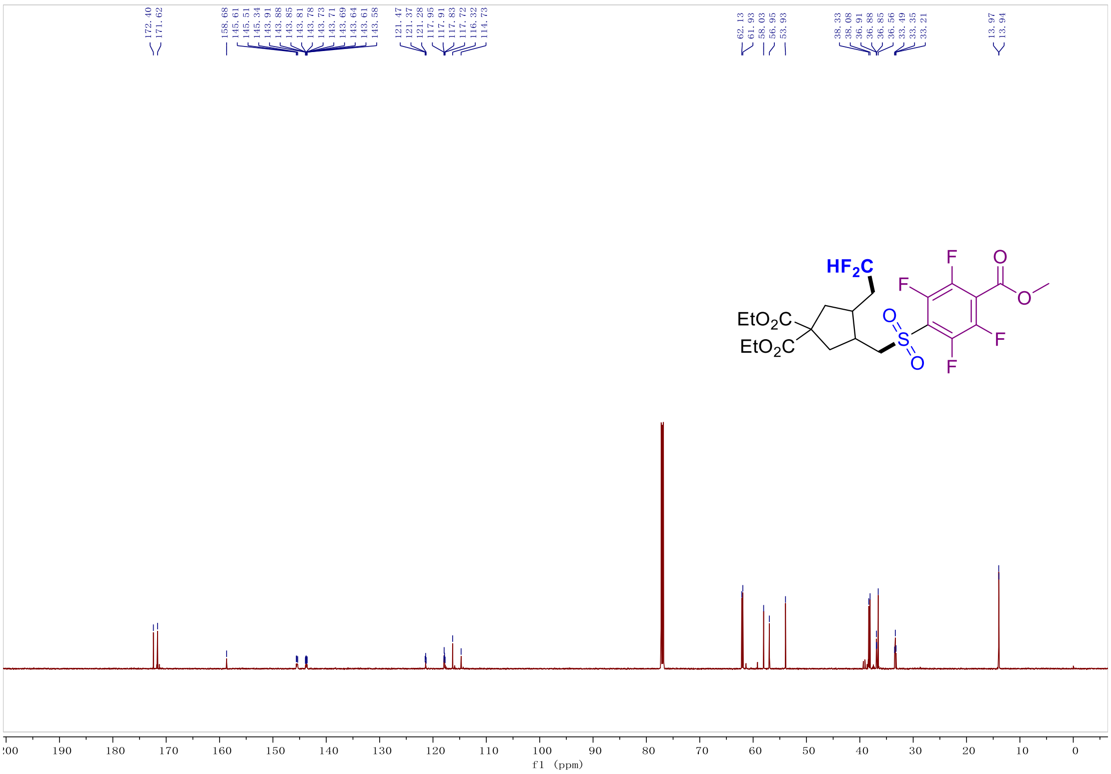

^{19}F NMR (565 MHz, $CDCl_3$) spectrum of 128

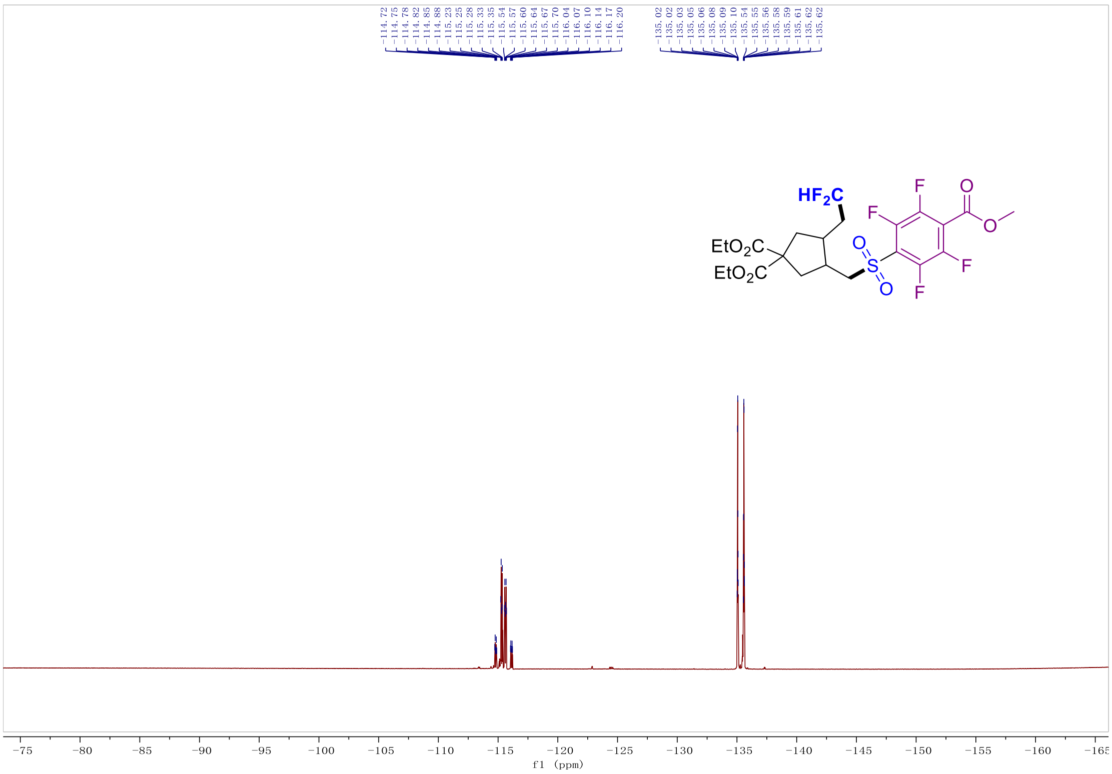

^{1}H NMR (500 MHz, CDCl3) spectrum of 129

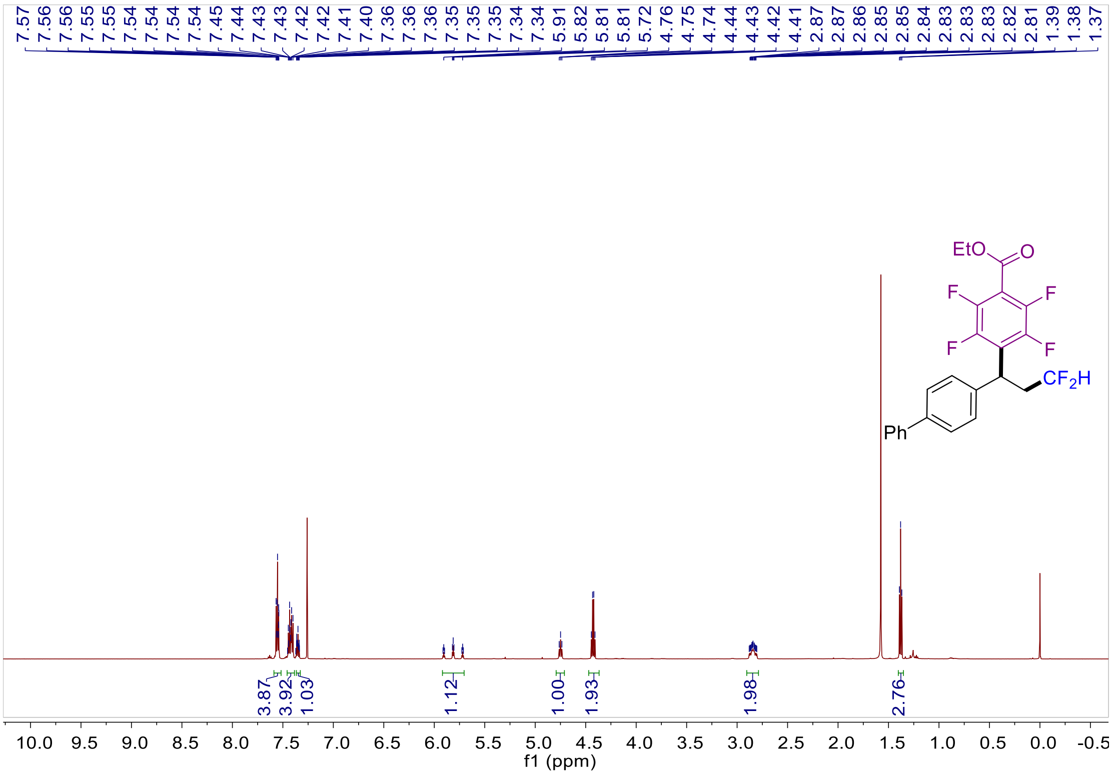

^{13}C NMR (151 MHz, CDCl3) spectrum of 129

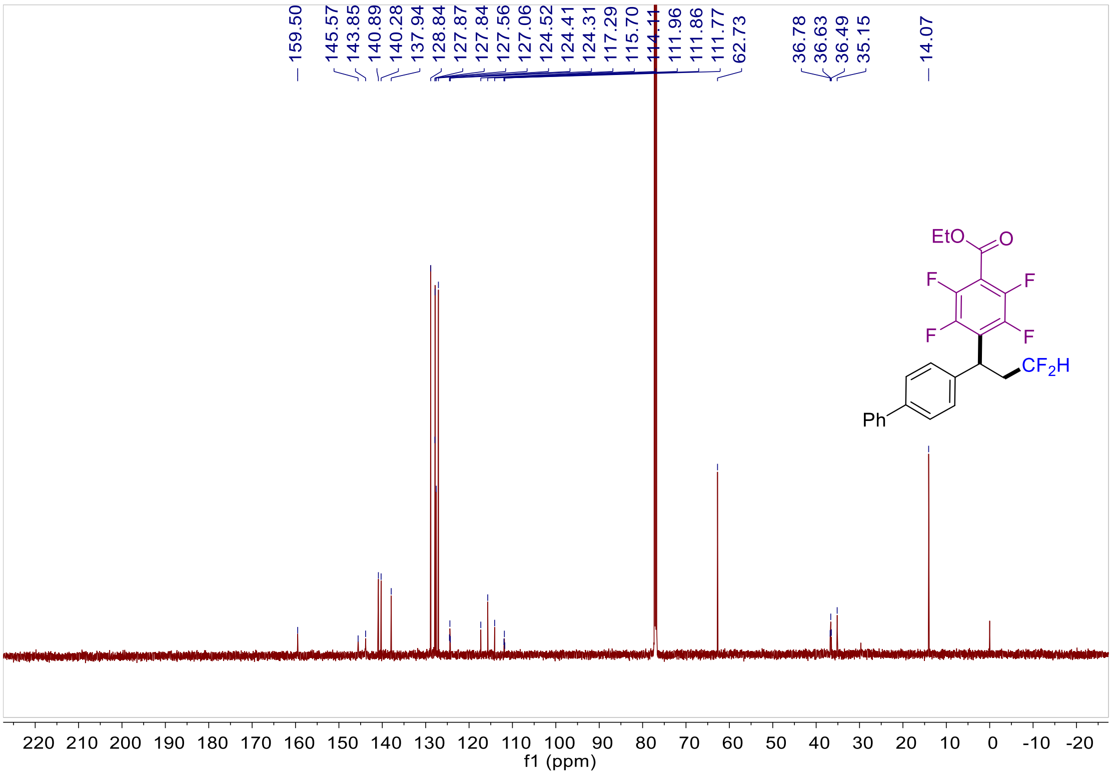

^{19}F NMR (565 MHz, $CDCl_3$) spectrum of 129

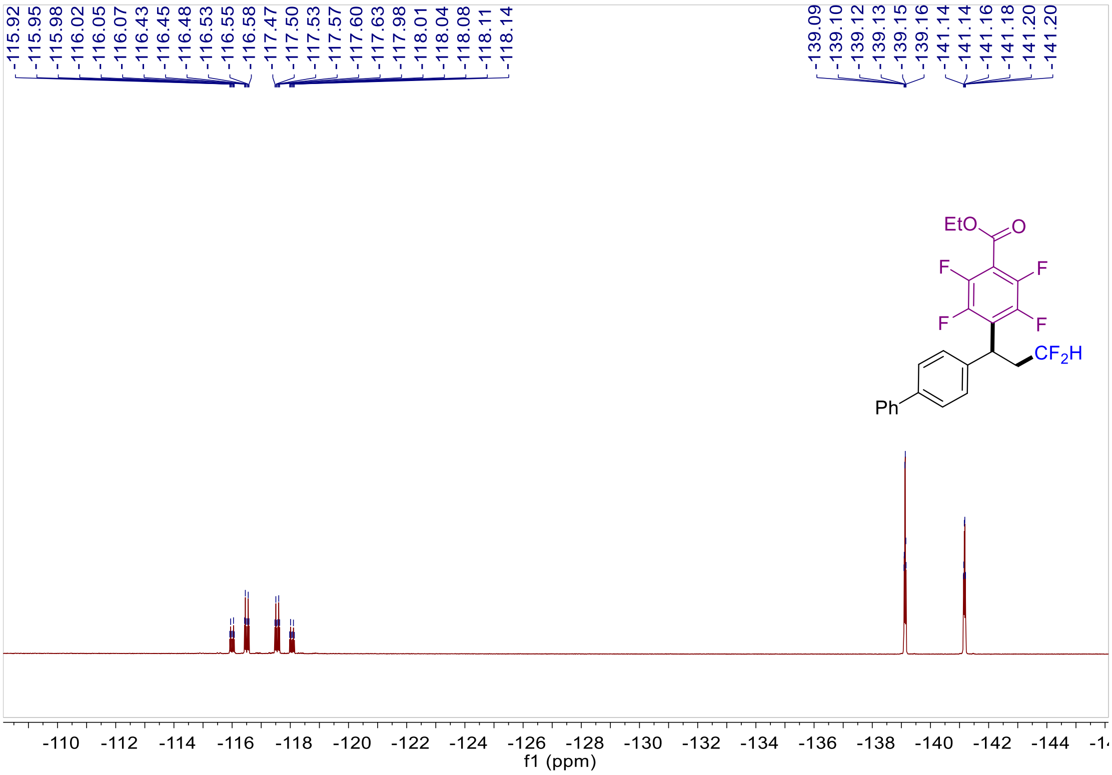

^{1}H NMR (600 MHz, $CDCl_3$) spectrum of 129a

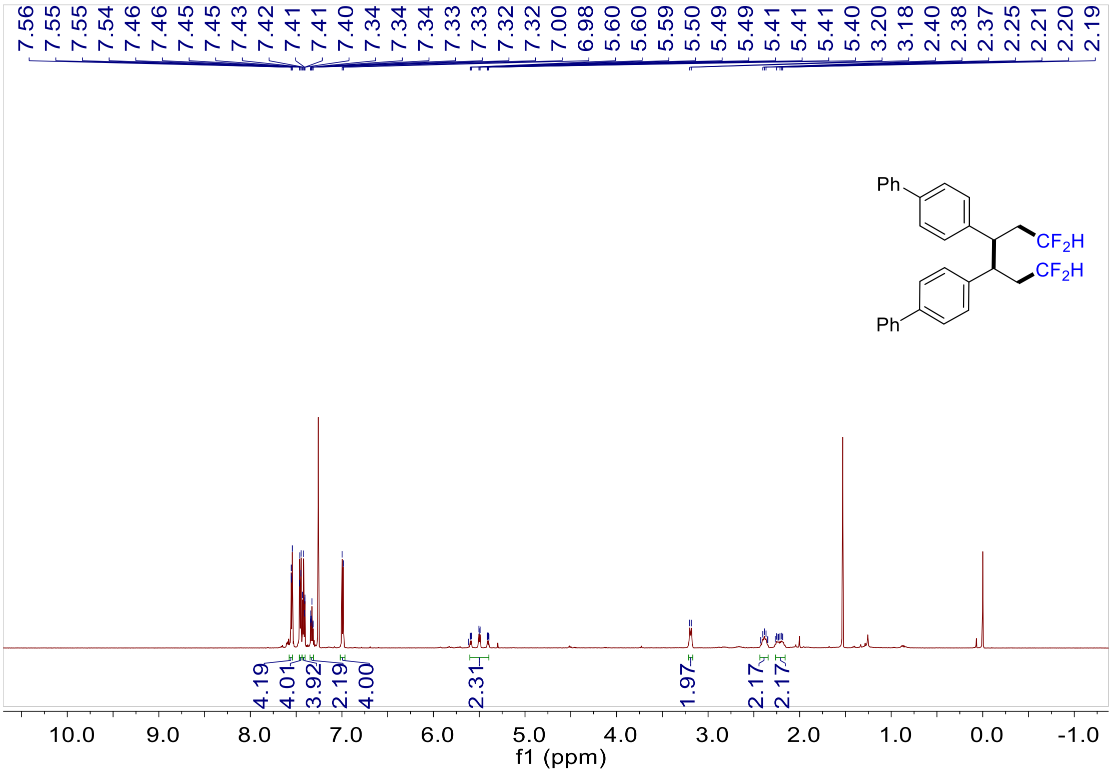

^{13}C NMR (151 MHz, $CDCl_3$) spectrum of 129a

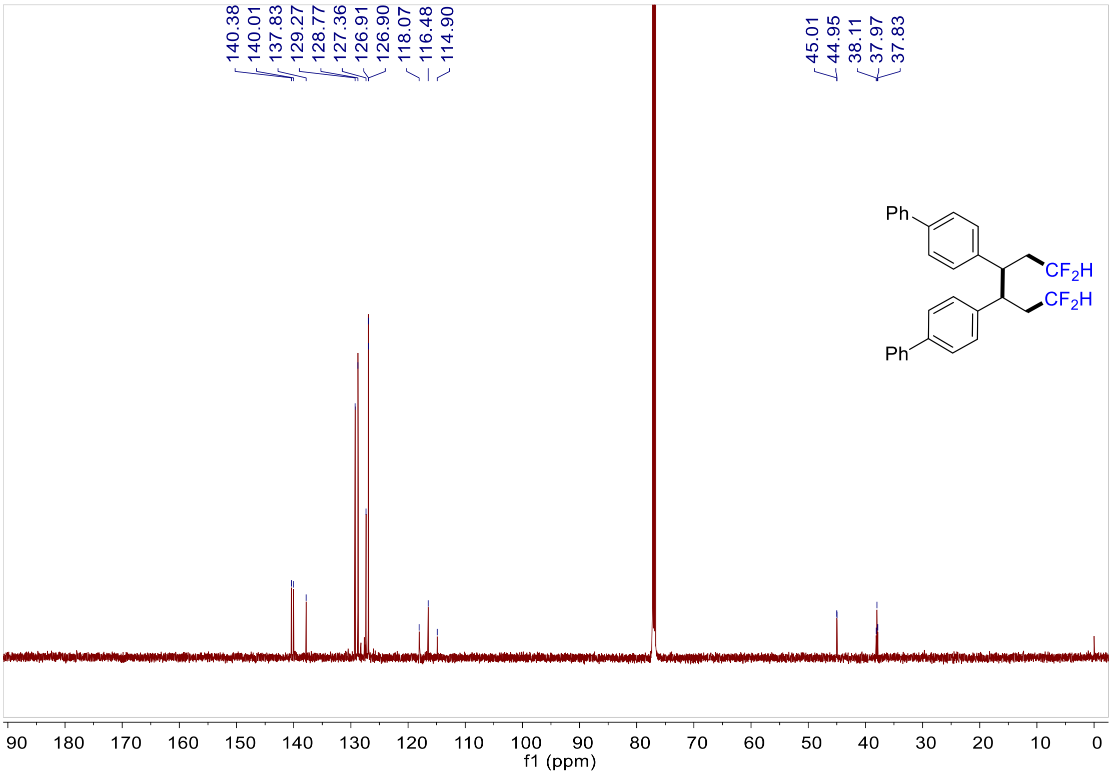

^{19}F NMR (565 MHz, $CDCl_3$) spectrum of 129a

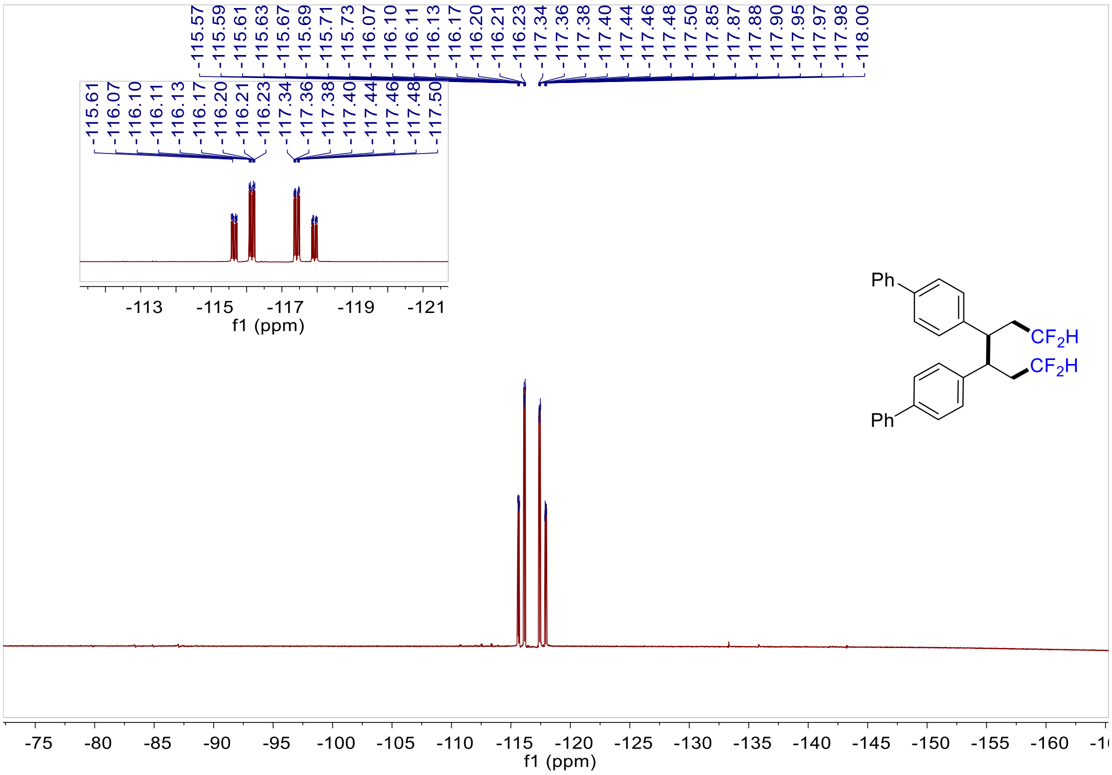

^{1}H NMR (600 MHz, CDCl$_3$) spectrum of 130

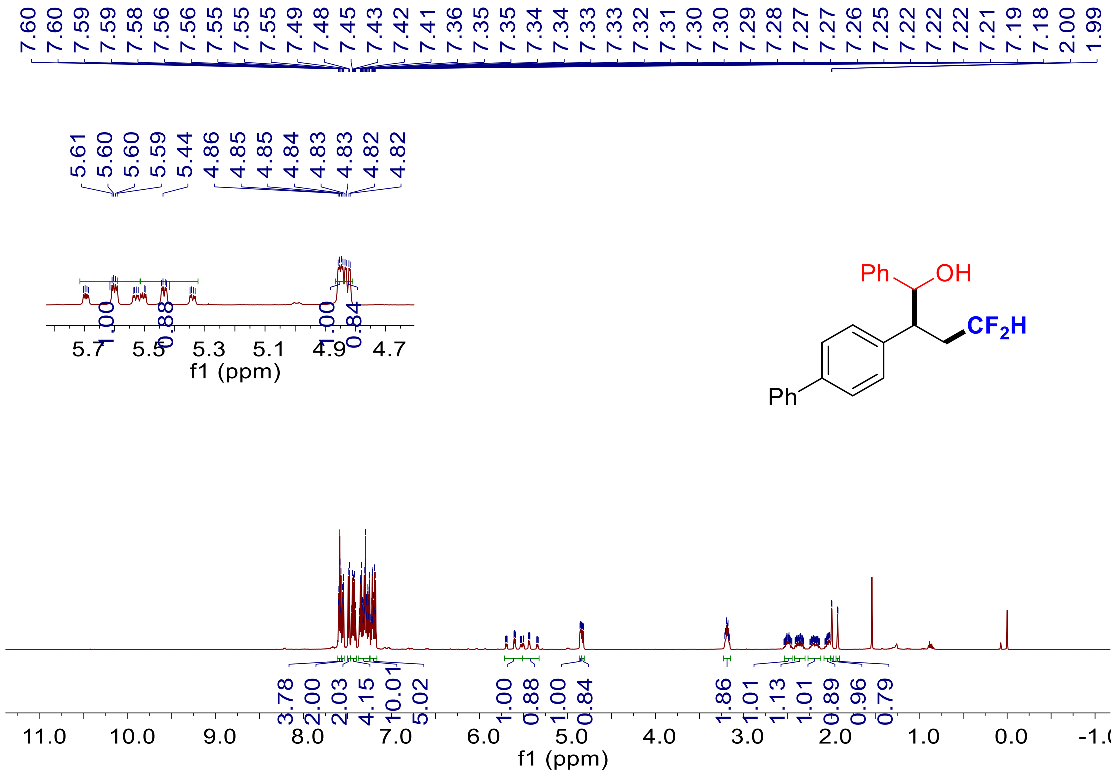

^{13}C NMR (151 MHz, CDCl$_3$) spectrum of 130

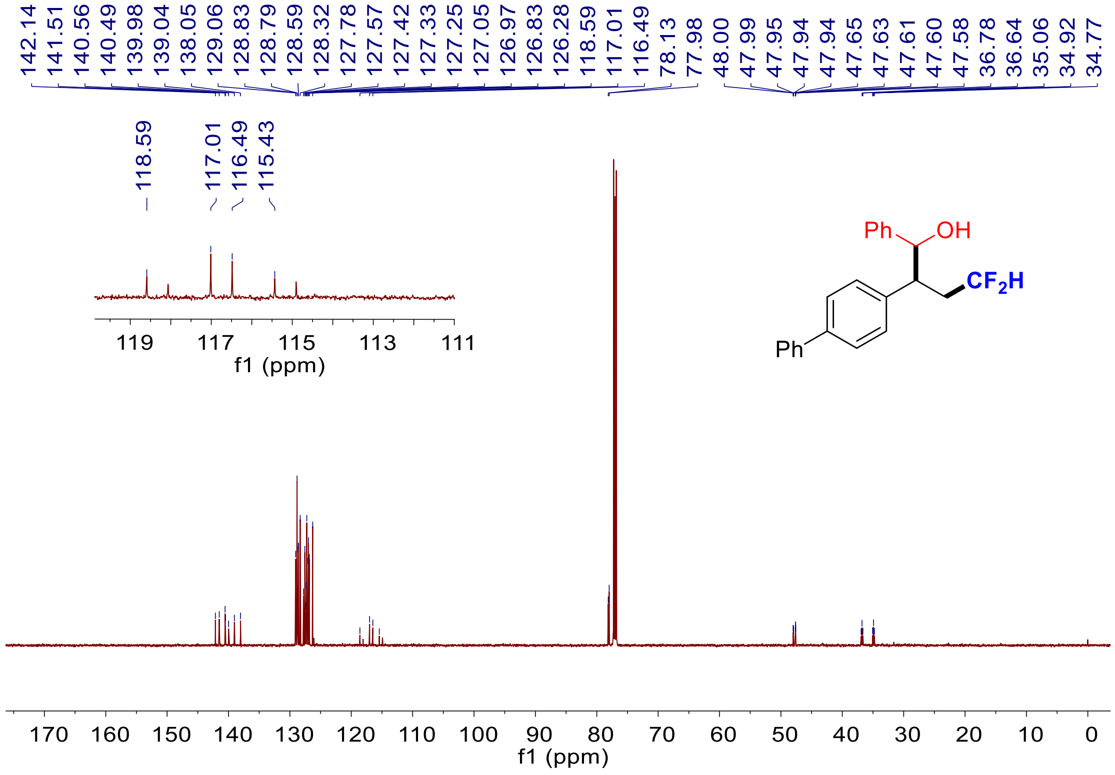

^{19}F NMR (565 MHz, CDCl$_3$) spectrum of 130

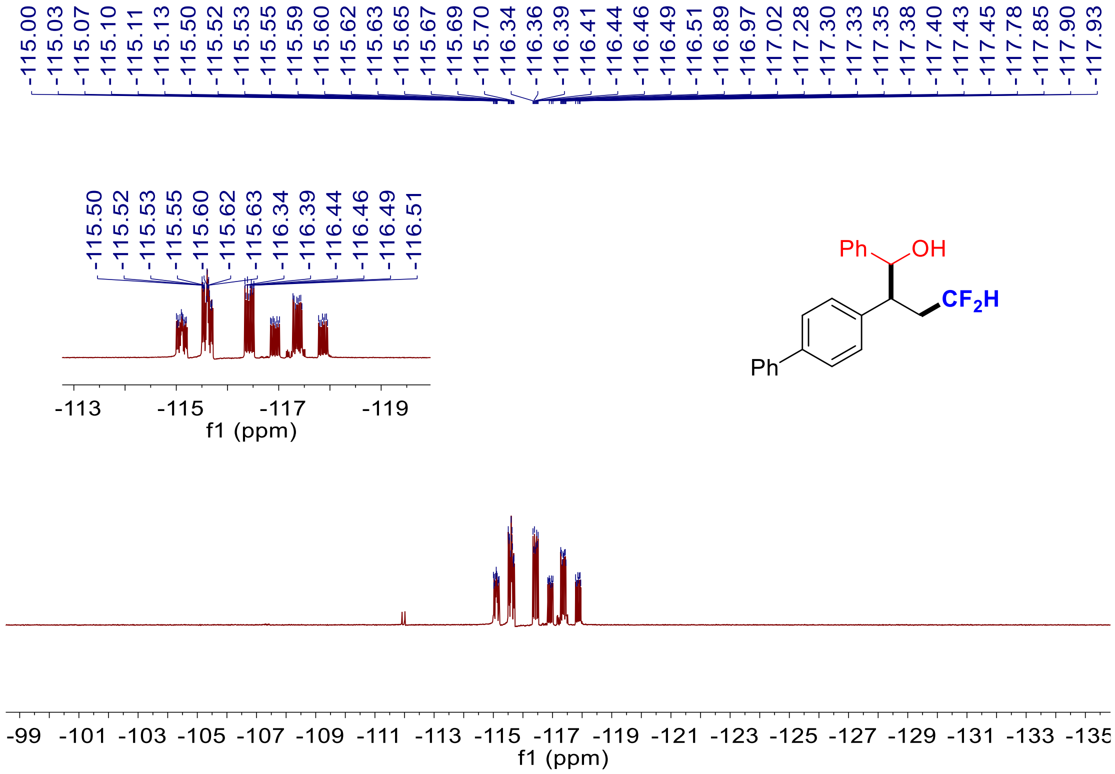

^{1}H NMR (600 MHz, CDCl$_3$) spectrum of 132

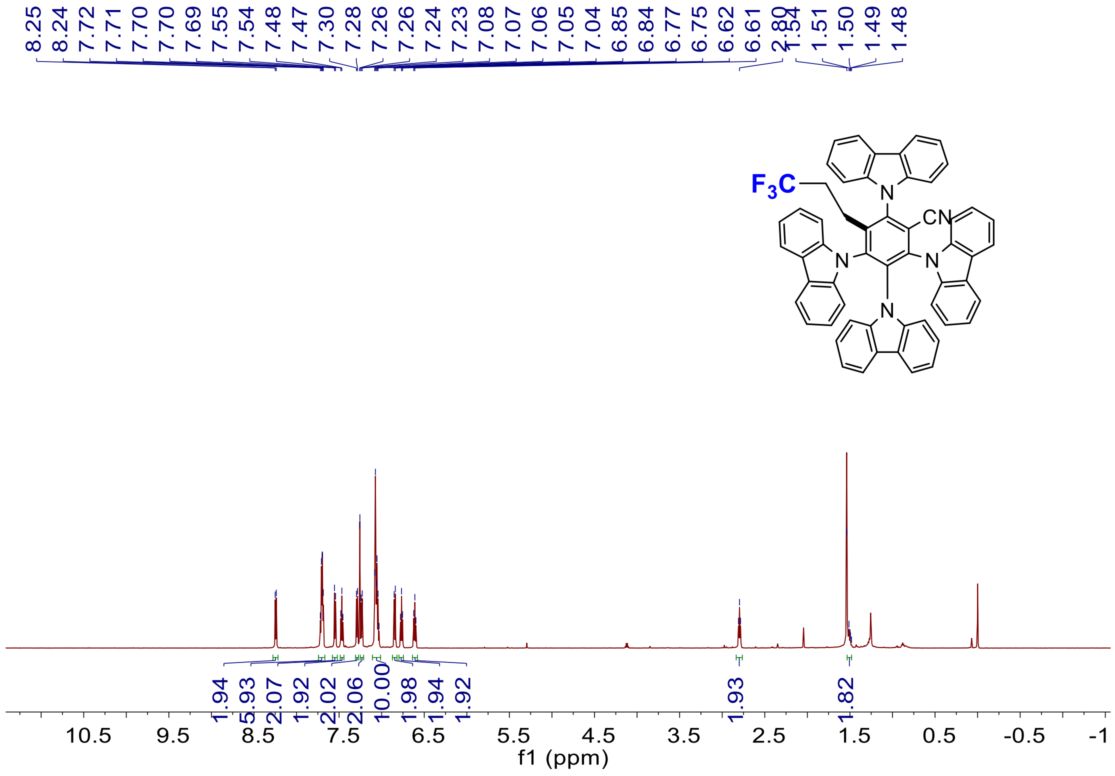

^{13}C NMR (151 MHz, $CDCl_3$) spectrum of 132

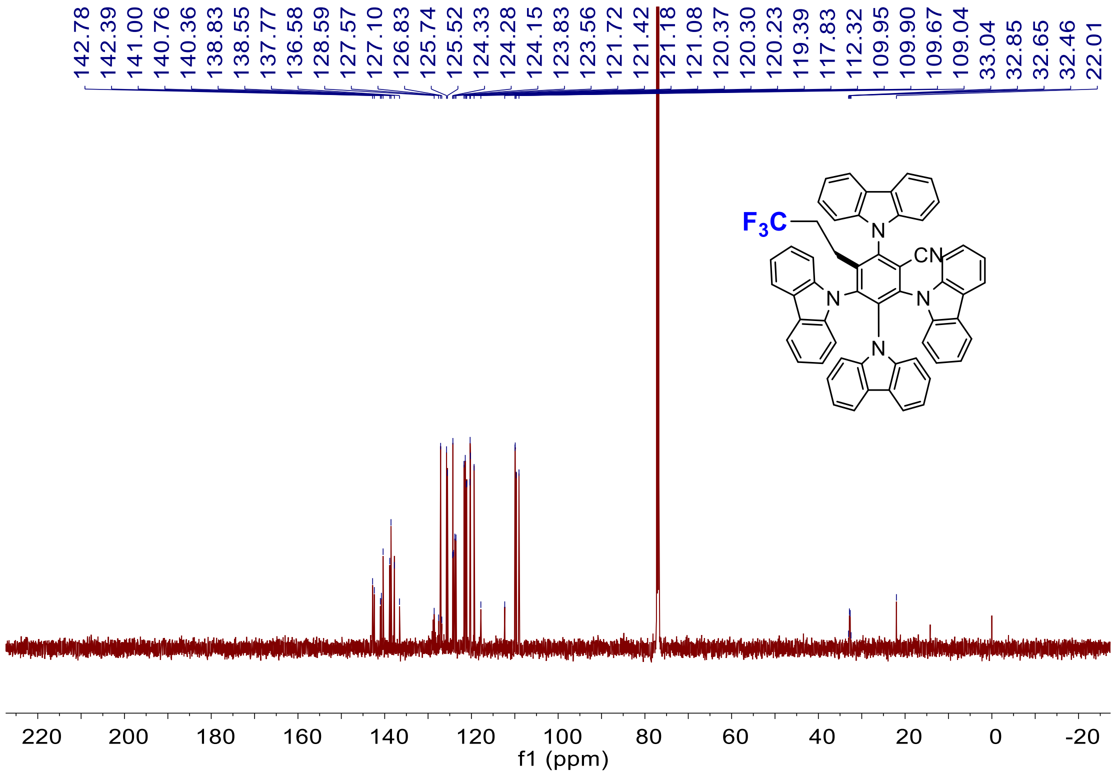

^{19}F NMR (565 MHz, $CDCl_3$) spectrum of 132

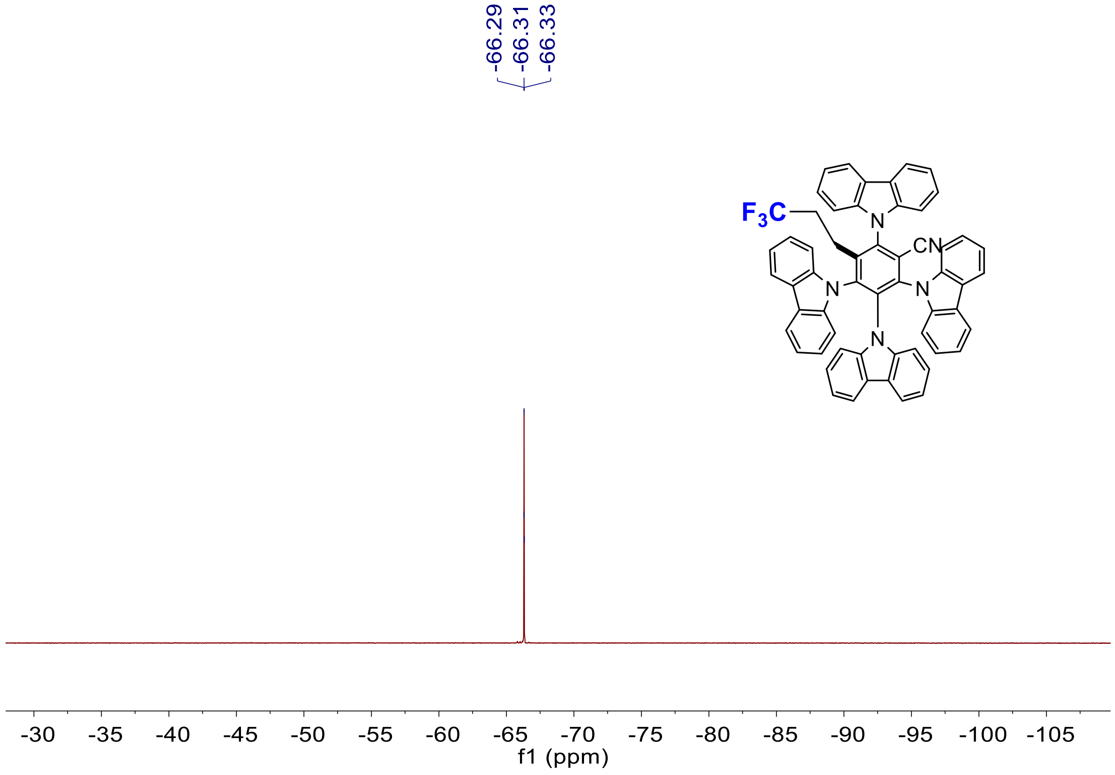

^{1}H NMR (600 MHz, CDCl$_3$) spectrum of 134

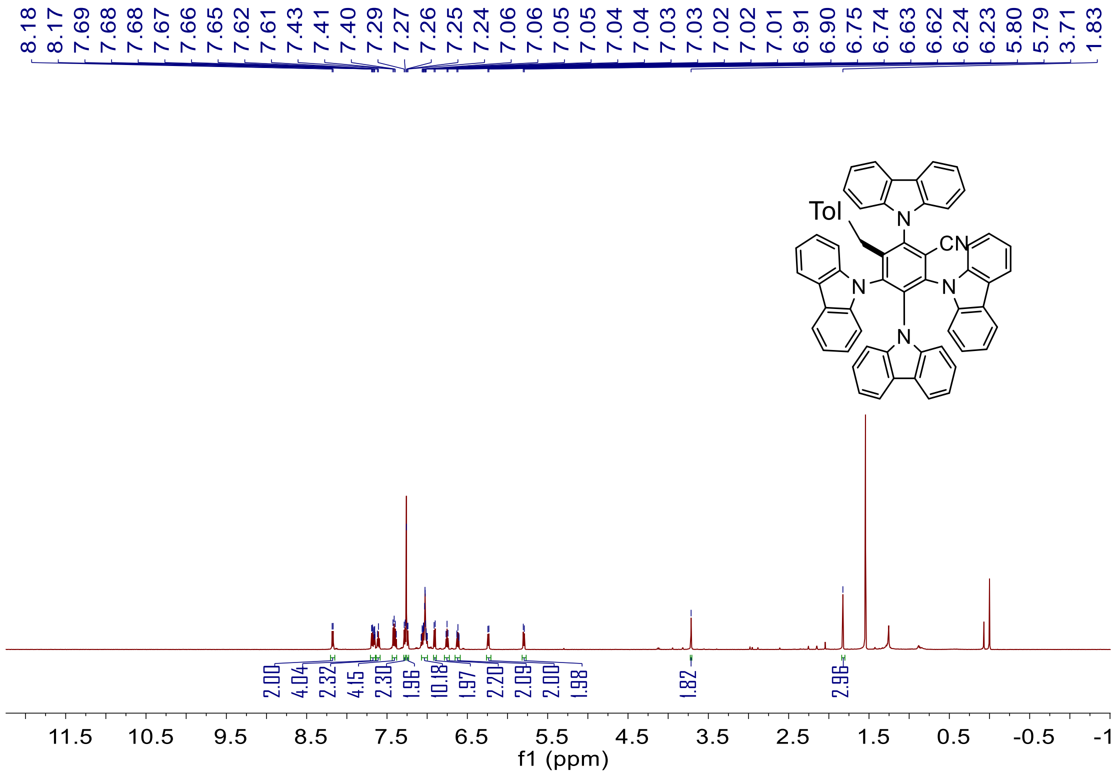

^{13}C NMR (151 MHz, CDCl$_3$) spectrum of 134

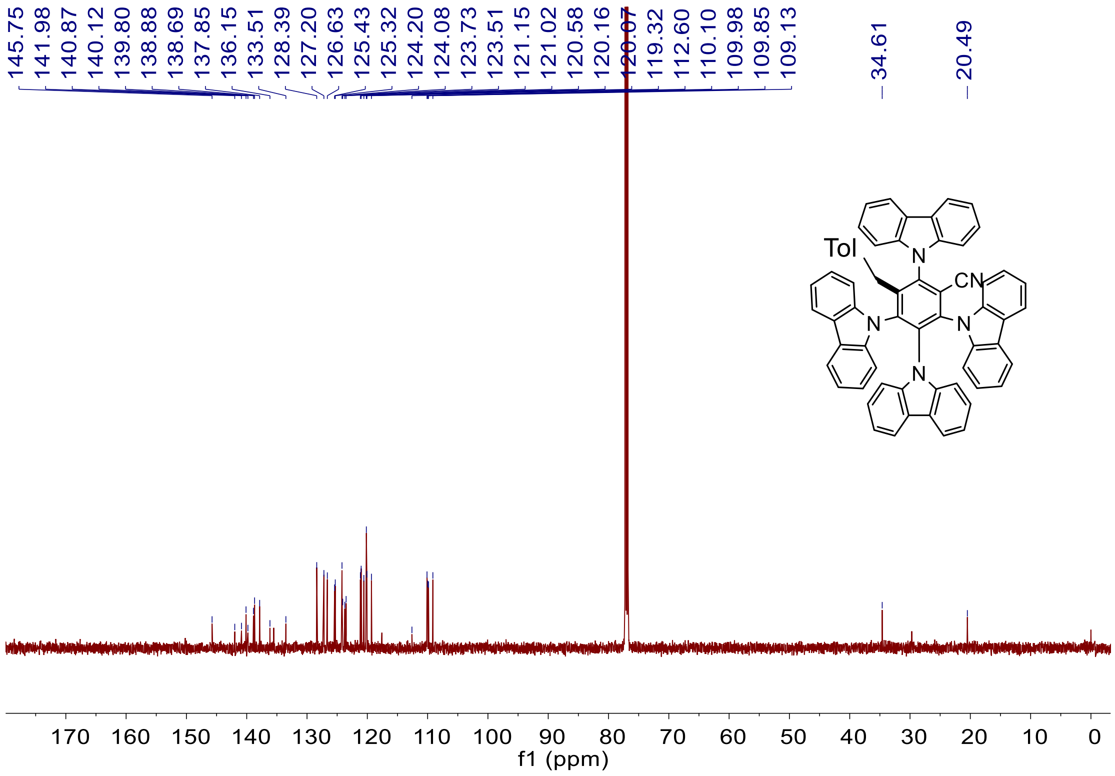

^{1}H NMR (600 MHz, $CDCl_3$) spectrum of 135

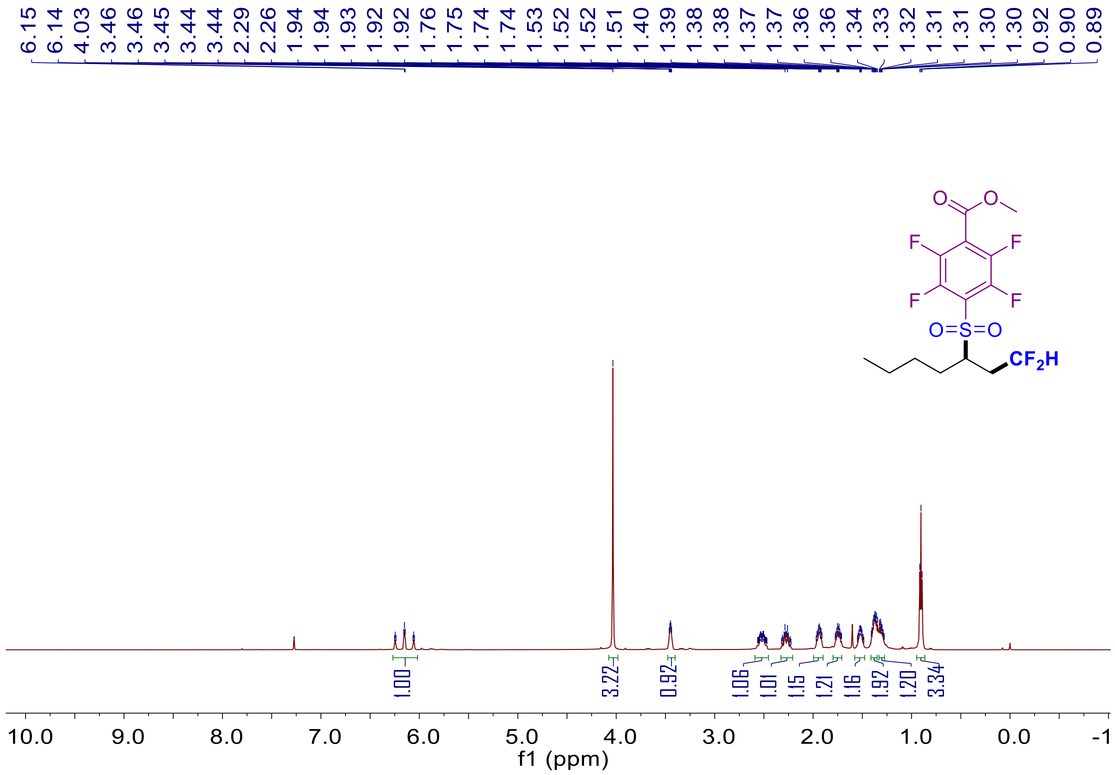

^{13}C NMR (151 MHz, $CDCl_3$) spectrum of 135

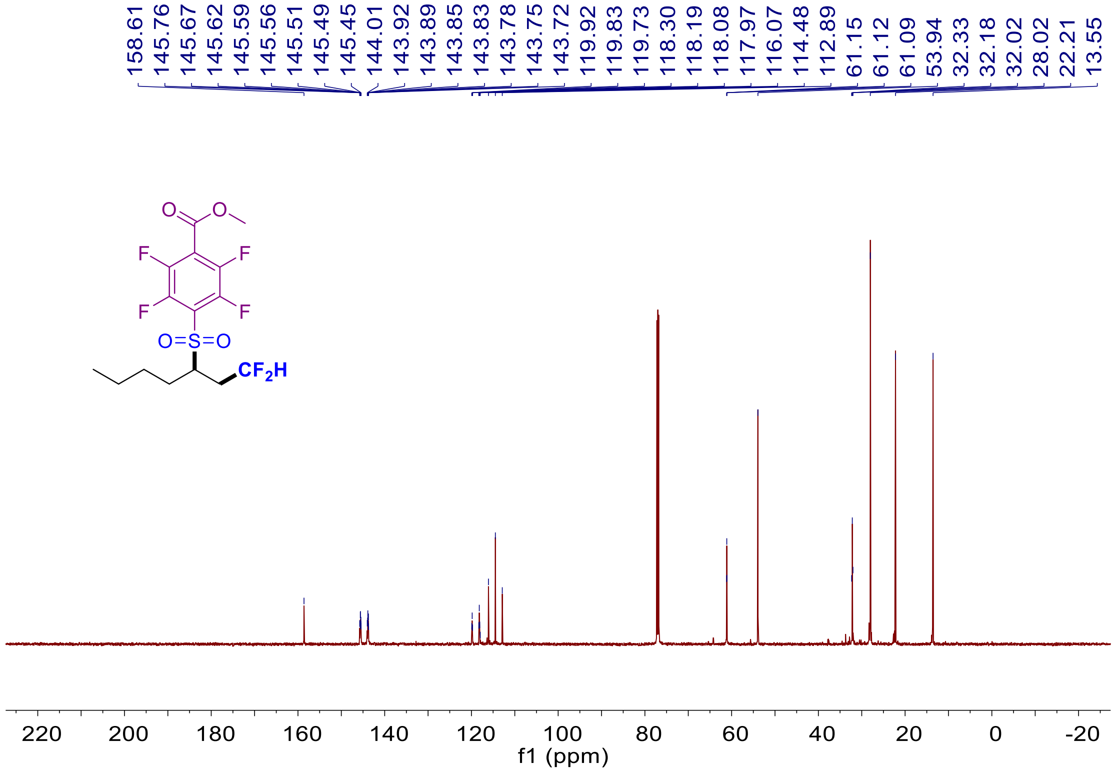

^{19}F NMR (565 MHz, $CDCl_3$) spectrum of 135

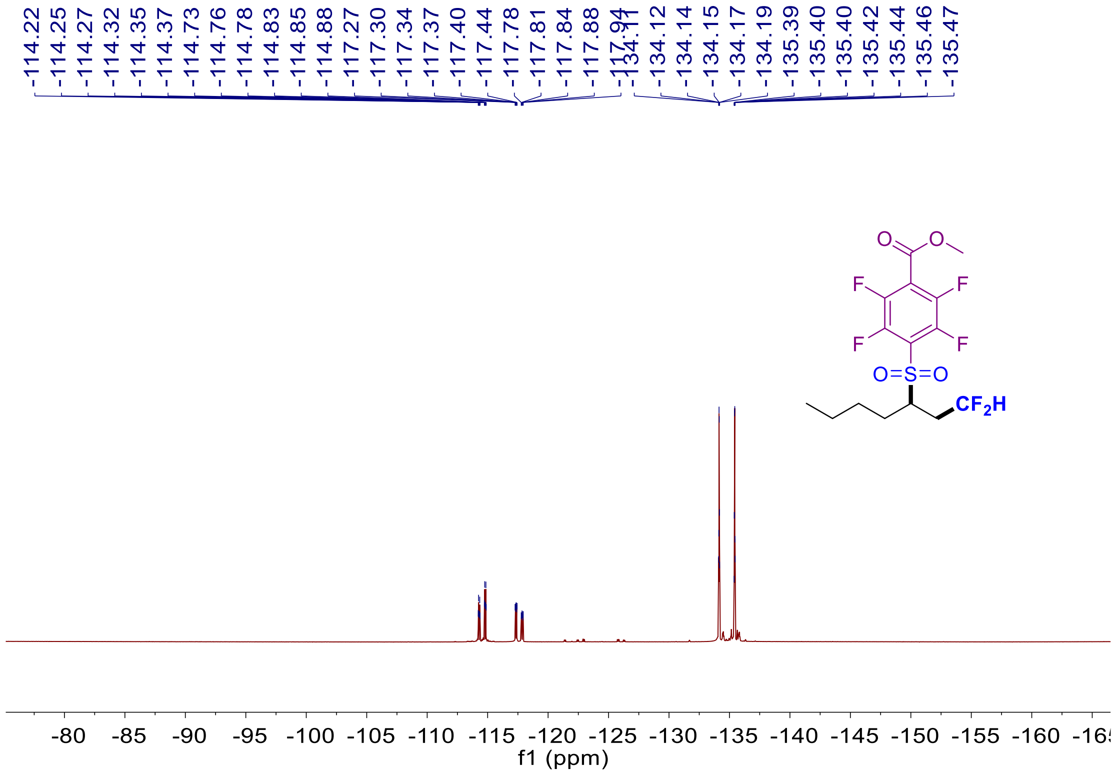

^{1}H NMR (600 MHz, $CDCl_3$) spectrum of 136

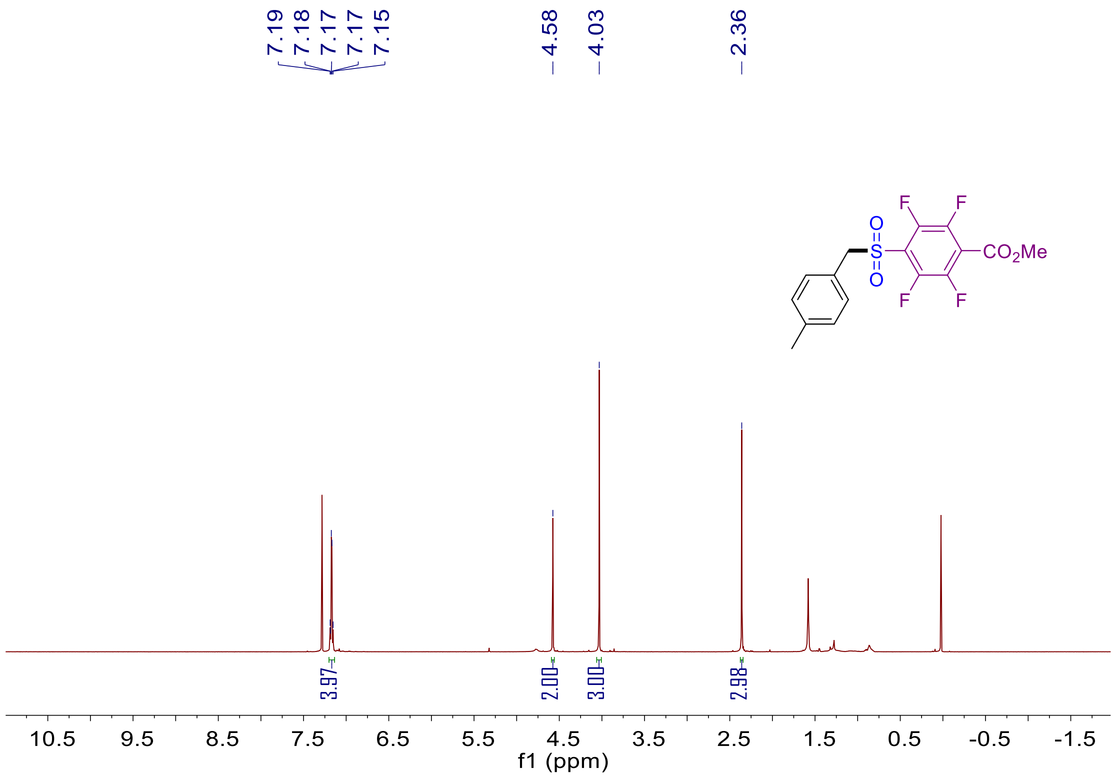

^{13}C NMR (151 MHz, CDCl$_3$) spectrum of 136

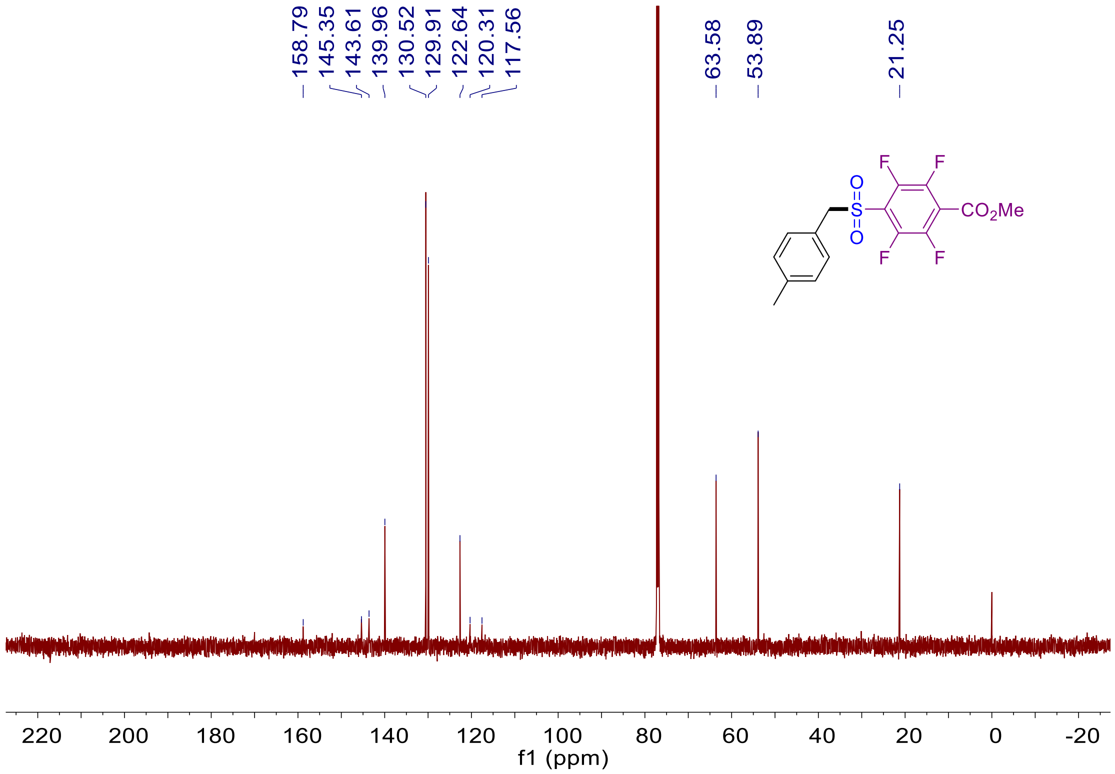

^{19}F NMR (565 MHz, CDCl$_3$) spectrum of 136

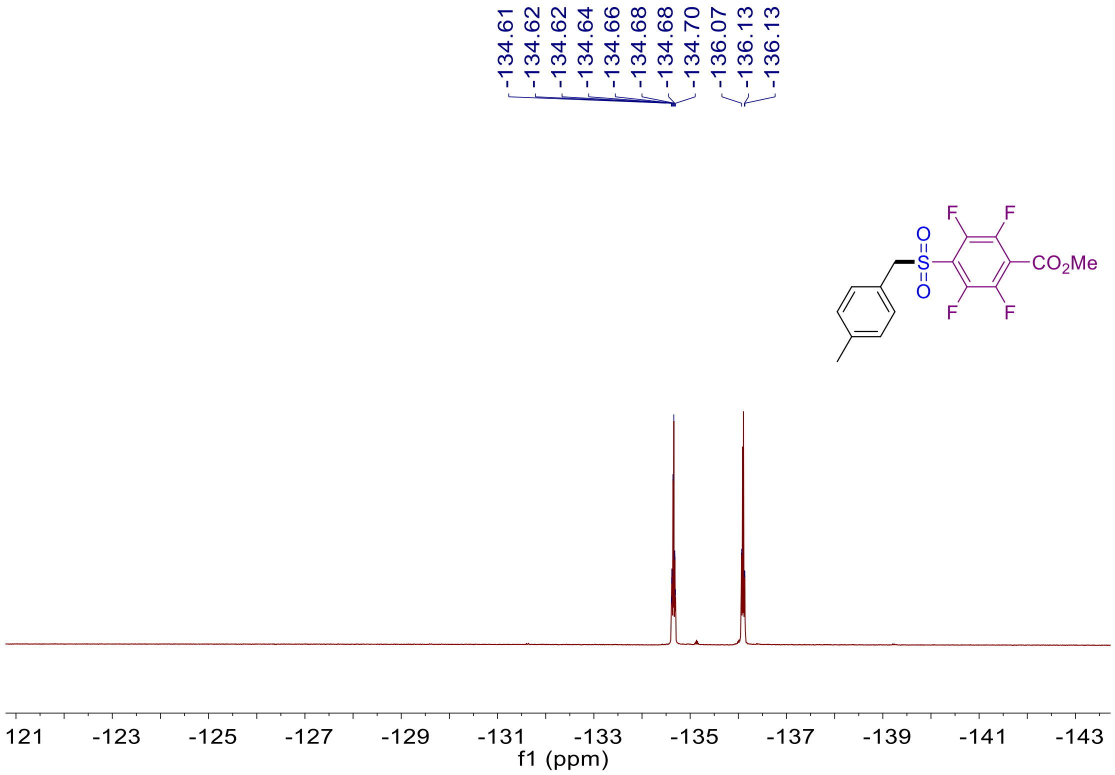

^{1}H NMR (600 MHz, $CDCl_3$) spectrum of 137

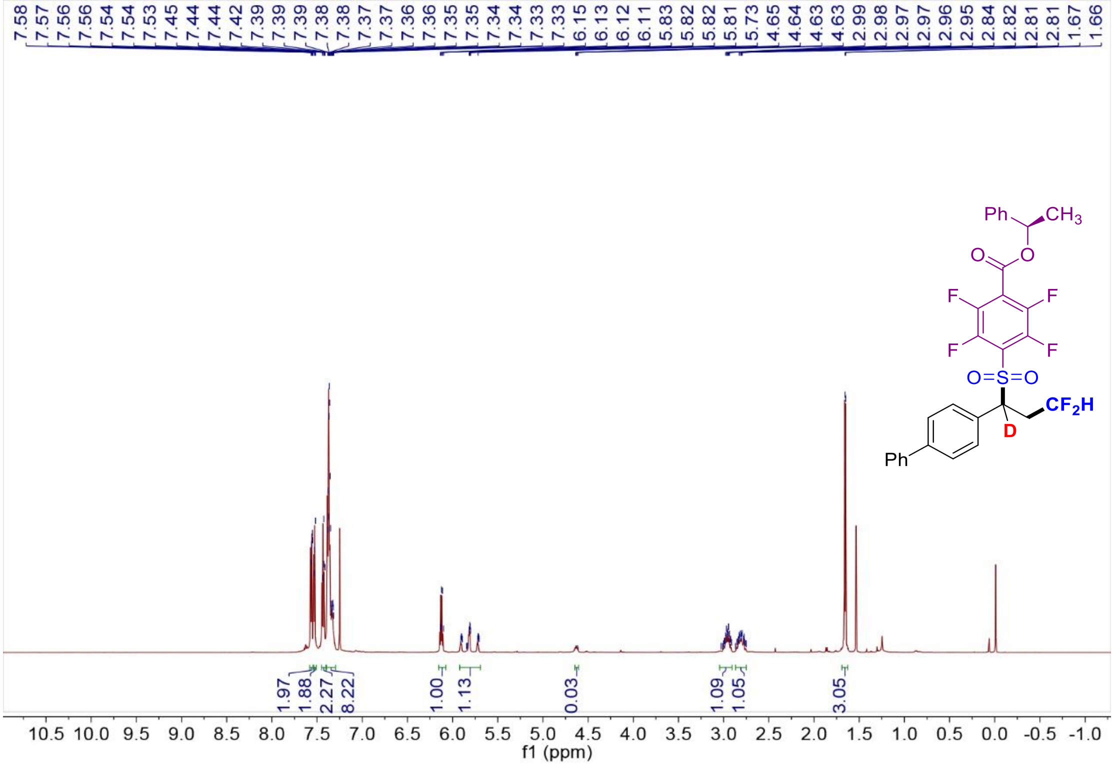

^{13}C NMR (151 MHz, $CDCl_3$) spectrum of 137

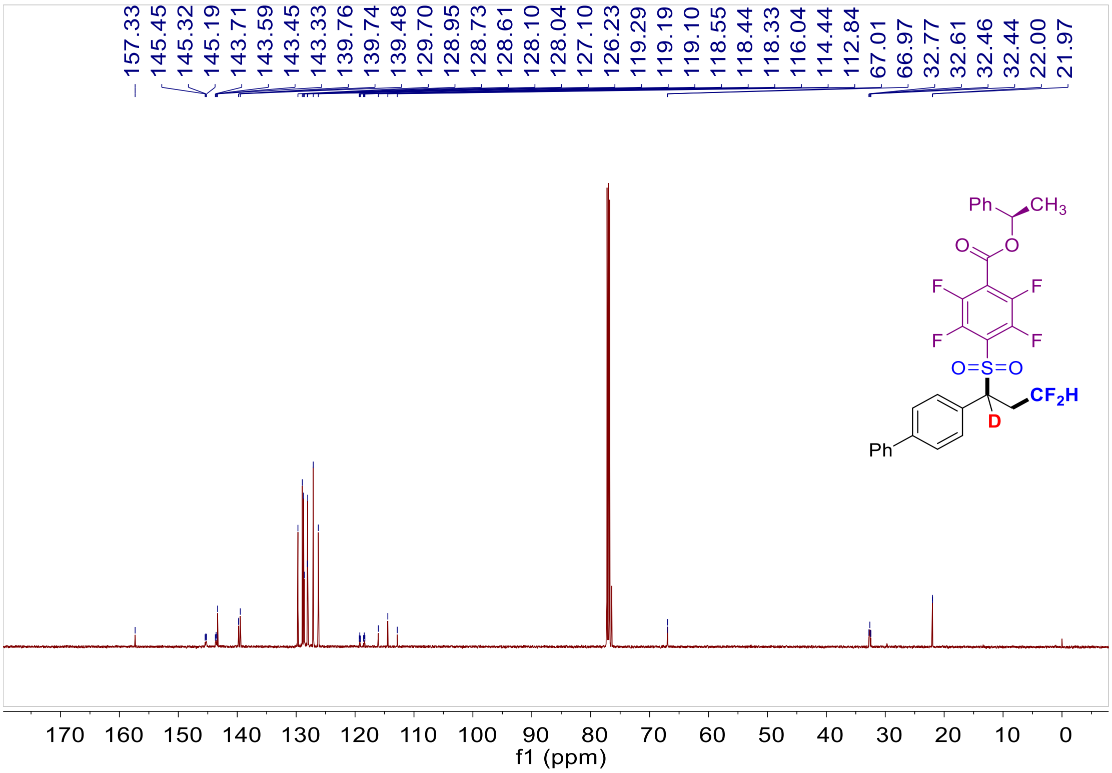

^{19}F NMR (565 MHz, $CDCl_3$) spectrum of 137

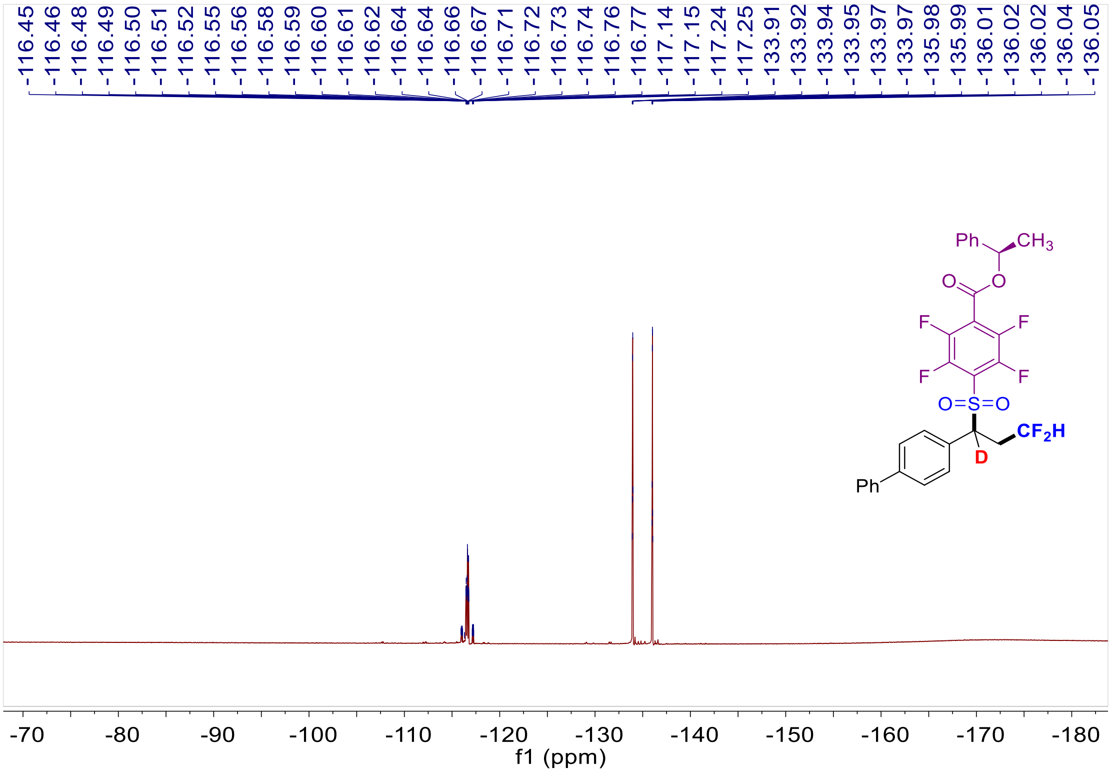

^{1}H NMR (600 MHz, $CDCl_3$) spectrum of 138

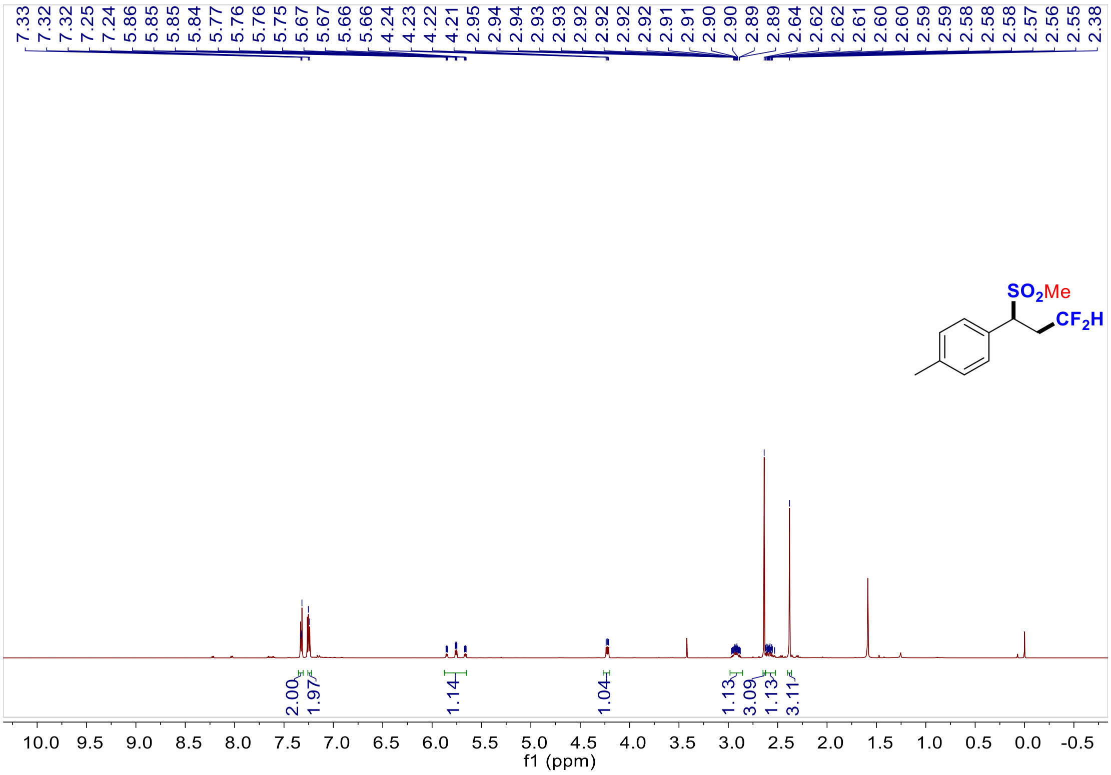

^{13}C NMR (151 MHz, $CDCl_3$) spectrum of 138

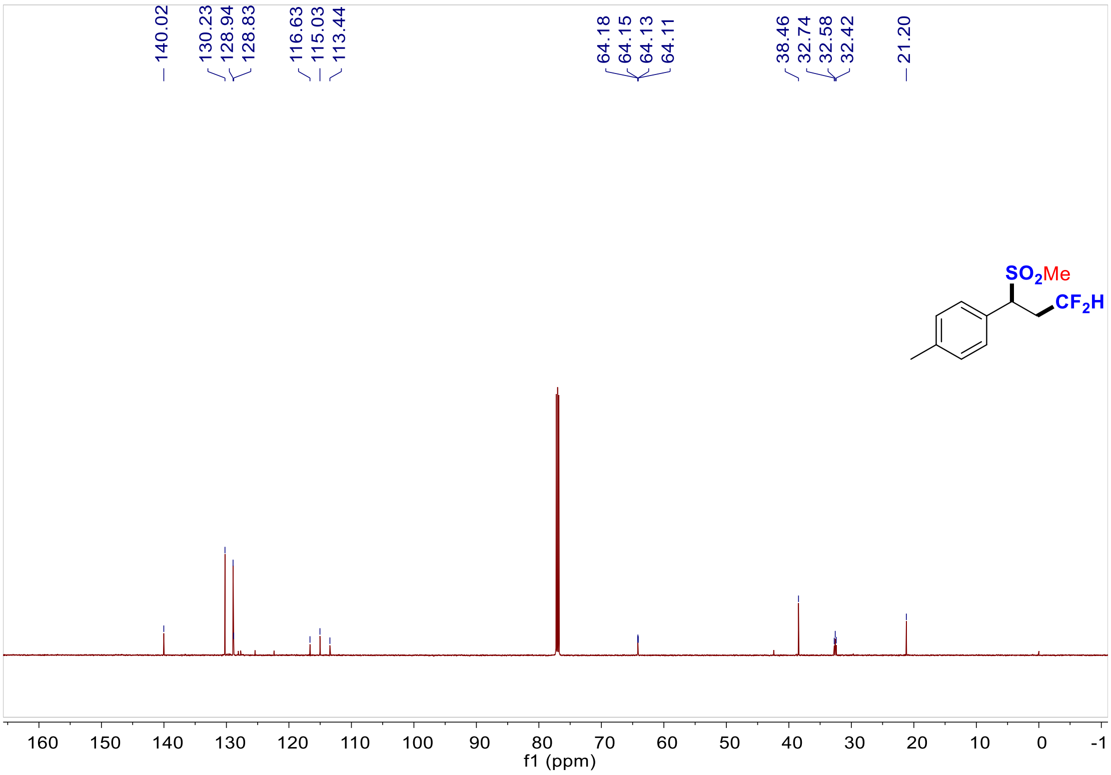

^{19}F NMR (565 MHz, $CDCl_3$) spectrum of 138

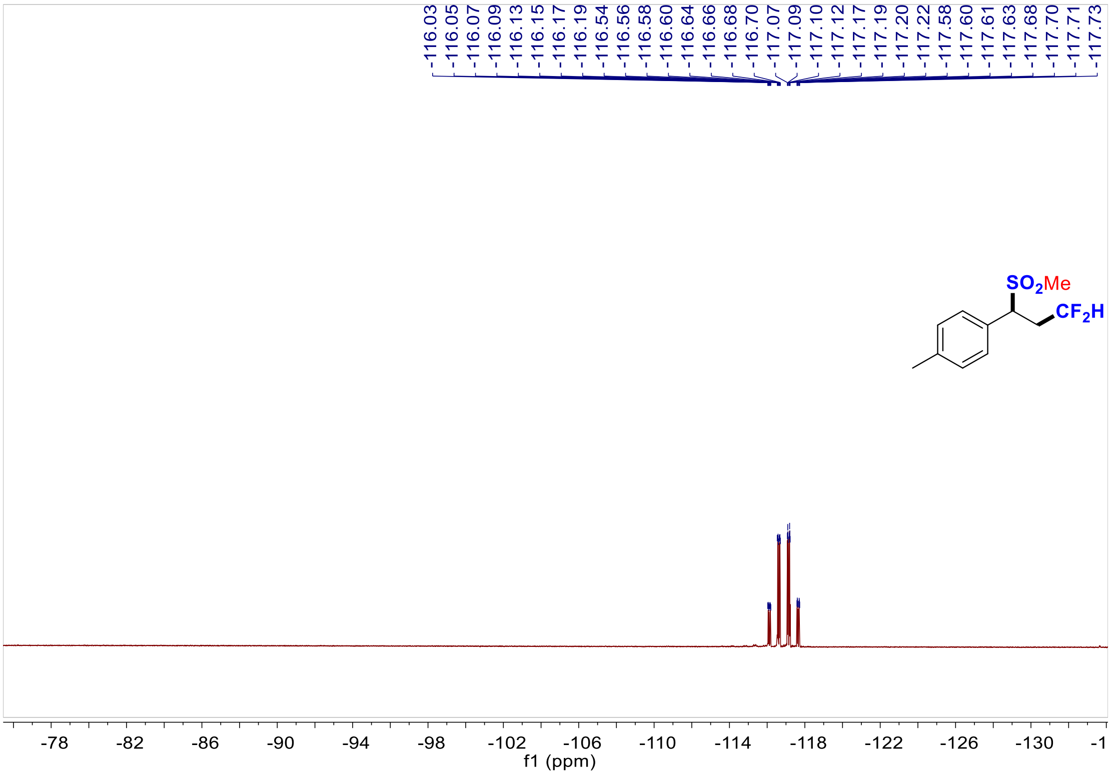

REFERENCES

1. "Ethylene industry installed capacity and capital expenditure (CapEx) forecast by region and countries including details of all active plants, planned and announced projects, 2023-2027" (GlobalData, 2023); www.globaldata.com/search/index/?SearchText=ethylene.

2. A. C. C. Campos, R. A. Reis, A. Ortiz, D. Gorri, A. Ortiz, Perspective of solutions for membrane instabilities in olefin/paraffin separations: A review. *Ind. Eng. Chem. Res.* **57**, 10071–10085 (2018).

3. M. Yan, J. C. Lo, J. T. Edwards, P. S. Baran, Radicals: Reactive intermediates with translational potential. *J. Am. Chem. Soc.* **138**, 12692–12714 (2016).

4. M.-J. Luo, Q. Xiao, J.-H. Li, Electro-/photocatalytic alkene-derived radical cation chemistry: Recent advances in synthetic applications. *Chem. Soc. Rev.* **51**, 7206–7237 (2022).

5. Z.-L. Li, G.-C. Fang, Q.-S. Gu, X.-Y. Liu, Recent advances in copper-catalysed radical-involved asymmetric 1,2-difunctionalization of alkenes. *Chem. Soc. Rev.* **49**, 32–48 (2020).

6. S. Engl, O. Reiser, Copper-photocatalyzed ATRA reactions: Concepts, applications, and opportunities. *Chem. Soc. Rev.* **51**, 5287–5299 (2022).

7. H. Jiang, A. Studer, Intermolecular radical carboamination of alkenes. *Chem. Soc. Rev.* **49**, 1790–1811 (2020).

8. F. Hanns, R. Leo, Factors controlling the addition of carbon-centered radicals to alkenes—An experimental and theoretical perspective. *Angew. Chem. Int. Ed. Engl.* **40**, 1340–1371 (2001).

9. P. Ehrlich, G. A. Mortimer, Fundamentals of the free-radical polymerization of ethylene. *Adv. Polymer. Sci.* **7**, 386–448 (1970).

10. Y. Nakamura, B. Ebeling, A. Wolpers, V. Monteil, F. D. Agosto, S. Yamago, Controlled radical polymerization of ethylene using organotellurium compounds. *Angew. Chem. Int. Ed. Engl.* **57**, 305–309 (2018).

11. H. Takano, H. Katsuyama, H. Hayashi, W. Kanna, Y. Harabuchi, S. Maeda, T. Mita, A theory-driven synthesis of symmetric and unsymmetric 1,2-bis(diphenylphosphino)ethane analogues via radical difunctionalization of ethylene. *Nat. Commun.* **13**, 7034 (2022).

12. J. Yu, X. Zhang, X. Wu, T. Liu, Z.-Q. Zhang, J. Wu, C. Zhu, Metal-free radical difunctionalization of ethylene. *Chem* **9**, 472–482 (2023).

13. X. Zhang, X. Wu, Y. Chen, C. Zhu, Metal-free radical-mediated alkylfunctionalization of ethylene and low-boiling-point alkenes. *Green Chem.* **25**, 4234–4238 (2023).

14. L.-C. Wang, B. Chen, Y. Zhang, X.-F. Wu, Nickel-catalyzed four-component carbonylation of ethers and olefins: Direct access to -γ-oxy esters and amides. *Angew. Chem. Int. Ed. Engl.* **61**, e202207970 (2022).

15. Y. Zhang, B.-H. Teng, X.-F. Wu, Copper-catalyzed trichloromethylative carbonylation of ethylene. *Chem. Sci.* **15**, 1418–1423 (2024).

16. T. Liu, T. Li, Z. Y. Tea, C. Wang, T. Shen, Z. Lei, X. Chen, W. Zhang, J. Wu, Modular assembly of arenes, ethylene and heteroarenes for the synthesis of 1,2-arylheteroaryl ethanes. *Nat. Chem.* **16**, 1705–1714 (2024).

17. T. O. Paulisch, L. A. Mai, F. Strieth-Kalthoff, M. J. James, C. Henkel, D. M. Guldi, F. Glorius, Dynamic kinetic sensitization of β-dicarbonyl compounds—Access to medium-sized rings by De Mayo-type ring expansion. *Angew. Chem. Int. Ed. Engl.* **61**, e202112695 (2022).

18. F. C. James, J. A. Kerr, J. P. Simons, Direct measurement of the rate of reaction of the methyl radical with sulphur dioxide. *J. Chem. Soc. Faraday Trans.* **69**, 2124–2129 (1973).

19. J. S. Hwang, C. P. Tsonis, Radicals in the synthesis of sulphur dioxide-based copolymers. *J. Polym. Sci. Part A: Polym. Chem.* **31**, 1417–1421 (1993).

20. S. Ye, M. Yang, J. Wu, Recent advances in sulfonylation reactions using potassium/sodium metabisulfite. *Chem. Commun.* **56**, 4145–4155 (2020).

21. W. Xiao, J.-Q. Chen, J. Wu, Radical sulfonylation with sulfur dioxide surrogates. *Chem. Soc. Rev.* **54**, 6832–6926 (2025).

22. J. Zhang, X. Wang, P. Wang, J. Fang, S. Li, J. Wu, SO_2-Insertion induced enantioselective oxysulfonylation to access β-chiral sulfones with quaternary carbon stereocenters. *Sci. China Chem.* **67**, 908–913 (2024).

23. J. Huang, F. Liu, L.-H. Zeng, S. Li, Z. Chen, J. Wu, Accessing chiral sulfones bearing quaternary carbon stereocenters via photoinduced radical sulfur dioxide insertion and Truce–Smiles rearrangement. *Nat. Commun.* **13**, 7081 (2022).

24. L. Chen, X. Zhang, M. Zhou, L. Shen, S. Kramer, Z. Lian, Enantioselective four-component arylsulfonylcyanation of vinylarenes via the insertion of SO_2 enabled by SOgen as SO_2 surrogate. *ACS Catal.* **12**, 10764–10770 (2022).

25. X. Hou, H. Liu, H. Huang, Iron-catalyzed fluoroalkylative alkylsulfonylation of alkenes via radical-anion relay. *Nat. Commun.* **15**, 1480 (2024).

26. V. T. Nguyen, G. C. Haug, V. D. Nguyen, N. T. H. Vuong, G. B. Karki, H. D. Arman, O. V. Larionov, Functional group divergence and the structural basis of acridine photocatalysis revealed by direct decarboxysulfonylation. *Chem. Sci.* **13**, 4170–4179 (2022).

27. W. P. Carson II, P. J. Sarver, N. S. Goudy, D. W. C. MacMillan, Photoredox catalysis-enabled sulfination of alcohols and bromides. *J. Am. Chem. Soc.* **145**, 20767–20774 (2023).

28. J. Zhu, W. C. Yang, X. D. Wang, L. Wu, Photoredox catalysis in C–S bond construction: Recent progress in photo-catalyzed formation of sulfones and sulfoxides. *Adv. Synth. Catal.* **360**, 386–400 (2018).

29. P.-Z. Wang, B. Zhang, W.-J. Xiao, J.-R. Chen, Photocatalysis meets copper catalysis: A new opportunity for asymmetric multicomponent radical cross-coupling reactions. *Acc. Chem. Res.* **57**, 3433–3448 (2024).

30. F.-D. Lu, J. Chen, J. X. Jiang, J.-R. Chen, L.-Q. Lu, W.-J. Xiao, Recent advances in transition-metal-catalysed asymmetric coupling reactions with light intervention. *Chem. Soc. Rev.* **50**, 12808–12827 (2021).

31. M. Li, Y. Wu, X. Song, J. Sun, Z. Zhang, G. Zheng, Q. Zhang, Visible light-mediated organocatalyzed 1,3-aminoacylation of cyclopropane employing *N*-benzoyl saccharin as bifunctional reagent. *Nat. Commun.* **15**, 8930 (2024).

32. M. Li, X. Song, X. Lu, J. Xia, G. Zheng, Q. Zhang, Visible light-mediated 1,3-acylative chlorination of cyclopropanes employing benzoyl chloride as bifunctional reagents in NHC catalysis. *Sci. China Chem.* **68**, 3628–3635 (2025).

33. L. Wang, J. Sun, J. Xia, M. Li, L. Zhang, R. Ma, G. Zheng, Q. Zhang, Visible light-mediated NHCs and photoredox co-catalyzed radical 1,2-dicarbonylation of alkenes for 1,4-diketones. *Sci. China Chem.* **65**, 1938–1944 (2022).

34. L. Wang, R. Ma, J. Sun, G. Zheng, Q. Zhang, NHC and visible light-mediated photoredox co-catalyzed 1,4-sulfonylacylation of 1,3-enynes for tetrasubstituted allenyl ketones. *Chem. Sci.* **13**, 3169–3175 (2022).

35. X. Li, B. Fu, Q. Zhang, X. Yuan, Q. Zhang, T. Xiong, Q. Zhang, Copper-catalyzed defluorinative hydroarylation of alkenes with polyfluoroarenes. *Angew. Chem. Int. Ed. Engl.* **59**, 23056–23060 (2020).

36. J. Wang, M. Sánchez-Roselló, J. L. Aceña, C. del Pozo, A. E. Sorochinsky, S. Fustero, V. A. Soloshonok, H. Liu, Fluorine in pharmaceutical industry: Fluorine-containing drugs introduced to the market in the last decade (2001–2011). *Chem. Rev.* **114**, 2432–2506 (2014).

37. C. Zhang, K. Yan, C. Fu, H. Peng, C. J. Hawker, A. K. Whittaker, Biological utility of fluorinated compounds: From materials design to molecular imaging, therapeutics and environmental remediation. *Chem. Rev.* **122**, 167–208 (2022).

38. J. B. I. Sap, C. F. Meyer, N. J. W. Straathof, N. Iwumene, C. W. A. Ende, A. A. Trabanco, V. Gouverneur, Late-stage difluoromethylation: Concepts, developments and perspective. *Chem. Soc. Rev.* **50**, 8214–8247 (2021).

39. M. O. Zubkov, A. D. Dilman, Radical reactions enabled by polyfluoroaryl fragments: Photocatalysis and beyond. *Chem. Soc. Rev.* **53**, 4741–4785 (2024).

40. H. Amii, K. Uneyama, C−F bond activation in organic synthesis. *Chem. Rev.* **109**, 2119–2183 (2009).

41. Y. Fujiwara, J. A. Dixon, F. O'Hara, E. D. Funder, D. D. Dixon, R. A. Rodriguez, R. D. Baxter, B. Herlé, N. Sach, M. R. Collins, Y. Ishihara, P. S. Baran, Practical and innate carbon–hydrogen functionalization of heterocycles. *Nature* **492**, 95–99 (2012).

42. Y. Fujiwara, J. A. Dixon, R. A. Rodriguez, R. D. Baxter, D. D. Dixon, M. R. Collins, D. G. Blackmond, P. S. Baran, A new reagent for direct difluoromethylation. *J. Am. Chem. Soc.* **134**, 1494–1497 (2012).

43. Z. He, P. Tan, C. Ni, J. Hu, Fluoroalkylative aryl migration of conjugated *N*-arylsulfonylated amides using easily accessible sodium di- and monofluoroalkanesulfinates. *Org. Lett.* **17**, 1838–1841 (2015).

44. R. I. Rodríguez, M. Sicignano, J. Alemán, Fluorinated sulfinates as source of alkyl radicals in the photo-enantiocontrolled β-functionalization of enals. *Angew. Chem. Int. Ed. Engl.* **61**, e202112632 (2022).

45. S. Zhang, L. Li, J. Zhang, J. Zhang, M. Xue, K. Xu, Electrochemical fluoromethylation triggered lactonizations of alkenes under semi-aqueous conditions. *Chem. Sci.* **10**, 3181–3185 (2019).

46. Y. Zhang, C. Ma, J. Struwe, J. Feng, G. Zhu, L. Ackermann, Elec-trooxidative dearomatization of biaryls: Synthesis of tri- and difluoromethylated spiro[5.5]trienones. *Chem. Sci.* **12**, 10092–10096 (2021).

47. W. Zhang, X.-X. Xiang, J. Chen, C. Yang, Y.-L. Pan, J.-P. Cheng, Q. Meng, X. Li, Direct C–H difluoromethylation of heterocycles via organic photoredox catalysis. *Nat. Commun.* **11**, 638 (2020).

48. Q. Y. Lin, X. H. Xu, K. Zhang, F. L. Qing, Visible-light-induced hydrodifluoromethylation of alkenes with a bromodifluoromethylphosphonium bromide. *Angew. Chem. Int. Ed. Engl.* **55**, 1479–1483 (2016).

49. C. F. Meyer, S. M. Hell, A. Misale, A. A. Trabanco, V. Gouverneur, Hydrodifluoromethylation of alkenes with difluoroacetic acid. *Angew. Chem. Int. Ed. Engl.* **58**, 8829–8833 (2019).

50. Y. Nakayama, G. Ando, M. Abe, T. Koike, M. Akita, Keto-difluoromethylation of aromatic alkenes by photoredox catalysis: Step-economical synthesis of α-CF_2H-substituted ketones in flow. *ACS Catal.* **9**, 6555–6563 (2019).

51. M. Zhang, J.-H. Lin, J.-C. Xiao, Photocatalyzed cyanodifluoromethylation of alkenes. *Angew. Chem. Int. Ed. Engl.* **58**, 6079–6083 (2019).

52. J. Yang, S. Zhu, F. Wang, F.-L. Qing, L. Chu, Silver-enabled general radical difluoromethylation reaction with $TMSCF_2H$. *Angew. Chem. Int. Ed. Engl.* **60**, 4300–4306 (2021).

53. J. Rong, L. Deng, P. Tan, C. Ni, Y. Gu, J. Hu, Radical fluoroalkylation of isocyanides with fluorinated sulfones by visible-light photoredox catalysis. *Angew. Chem. Int. Ed. Engl.* **55**, 2743–2747 (2016).

54. D. B. Bagal, G. Kachkovskyi, M. Knorn, T. Rawner, B. M. Bhanage, O. Reiser, Trifluoromethylchlorosulfonylation of alkenes: Evidence for an inner-sphere mechanism by a copper phenanthroline photoredox catalyst. *Angew. Chem. Int. Ed. Engl.* **54**, 6999–7002 (2015).

55. Y. Liu, H. Wu, Y. Guo, J.-C. Xiao, Q.-Y. Chen, C. Liu, Trifluoromethylfluorosulfonylation of unactivated alkenes using readily available $Ag(O_2CCF_2SO_2F)$ and *N*-fluorobenzenesulfonimide. *Angew. Chem. Int. Ed. Engl.* **56**, 15432–15435 (2017).

56. Z. Li, L. Jiao, Y. Sun, Z. He, Z. Wei, W. W. Liao, CF_3SO_2Na as a bifunctional reagent: Electrochemical trifluoromethylation of alkenes accompanied by so_2 insertion to access trifluoromethylated cyclic *N*-sulfonylimines. *Angew. Chem. Int. Ed. Engl.* **59**, 7266–7270 (2020).

57. H. Wang, P. Bellotti, X. Zhang, T. O. Paulisch, F. Glorius, A base-controlled switch of SO_2 reincorporation in photocatalyzed radical difunctionalization of alkenes. *Chem* **7**, 3412–3424 (2021).

58. M. Kim, E. You, J. Kim, S. Hong, Site-selective pyridylic C−H functionalization by photocatalytic radical cascades. *Angew. Chem. Int. Ed. Engl.* **61**, e202204217 (2022).

59. H. Li, Y. Zhang, X. Yang, Z. Deng, Z. Zhu, P. Zhou, X. Ouyang, Y. Yuan, X. Chen, L. Yang, M. Liu, C. Shu, Synthesis of multifluoromethylated γ-sultines by a photoinduced radical addition–polar cyclization. *Angew. Chem. Int. Ed. Engl.* **62**, e202300159 (2023).

60. Z. Deng, Z. Zhu, Z. Ru, X. Zou, X. Ouyang, H. Li, X. Yang, P. Zhou, S. Tian, X. Ma, R. Song, Q. Sun, C. X. Lin, C. Shu, Energy-transfer-powered sultine synthesis. *ACS Catal.* **13**, 13232–13244 (2023).

61. B. Shan, J. C. Medina, E. Santha, W. P. Frankmoelle, T.-C. Chou, R. M. Learned, M. R. Narbut, D. Stott, P. Wu, J. C. Jaen, T. Rosen, P. B. M. W. M. Timmermans, H. Beckmann, Selective, covalent modification of β-tubulin residue Cys-239 byT138067, an antitumor agent with in vivo efficacy againstmultidrug-resistant tumors. *Proc. Natl. Acad. Sci. U.S.A.* **96**, 5686–5691 (1999).

62. J. D. Smith, A. M. Jamhawi, J. B. Jasinski, F. Gallou, J. Ge, R. Advincula, J. Liu, S. Handa, Organopolymer with dual chromophores and fast charge-transfer properties for sustainable photocatalysis. *Nat. Commun.* **10**, 1837 (2019).

63. Y. Li, H. Wei, D. Wu, Z. Li, W. Wang, G. Yin, Nickel-catalyzed chemodivergent 1,1-difunctionalization of unactivated α-olefins with alkynyl electrophiles and B_2pin_2. *ACS Catal.* **10**, 4888–4894 (2020).

64. M. Lepori, C. Pratley, I. Dey, V. Butera, V. Roider, J. P. Barham, Photocatalysis enables chemodivergent radical polar crossover: Ritter-type amidation vs heck-type olefin carbofunctionalizations. *Chem. A Eur. J.* **31**, e202500666 (2025).

65. K. Chen, Q. Zeng, L. Xie, Z. Xue, Z. Wang, Y. Xu, Functional-group translocation of cyano groups by reversible C–H sampling. *Nature* **620**, 1007–1012 (2023).

66. M. Wang, Y. Huang, P. Hu, Terminal C(sp^3)–H borylation through intermolecular radical sampling. *Science* **383**, 537–544 (2024).

67. H. A. Sakai, D. W. C. MacMillan, Nontraditional fragment couplings of alcohols and carboxylic acids: C(sp^3)–C(sp^3) cross-coupling via radical sorting. *J. Am. Chem. Soc.* **144**, 6185–6192 (2022).

68. J. Z. Wang, W. L. Lyon, D. W. C. MacMillan, Alkene dialkylation by triple radical sorting. *Nature* **628**, 104–109 (2024).

69. Z. Xing, F. Liu, J. Feng, L. Yu, Z. Wu, B. Zhao, B. Chen, H. Ping, Y. Xu, A. Liu, Y. Zhao, C. Wang, B. Wang, X. Huang, Synergistic photobiocatalysis for enantioselective triple-radical sorting. *Nature* **637**, 1118–1123 (2025).

70. R. Chen, N. E. Intermaggio, J. Xie, J. A. Rossi-Ashton, C. A. Gould, R. T. Martin, J. Alcázar, D. W. C. MacMillan, Alcohol-alcohol cross-coupling enabled by S_H2 radical sorting. *Science* **383**, 1350–1357 (2024).

71. M. Liu, X. Ouyang, C. Xuan, C. Shu, Advances in photoinduced radical–polar crossover cyclization (RPCC) of bifunctional alkenes. *Org. Chem. Front.* **11**, 895–915 (2024).

72. S. Sharma, J. Singh, A. Sharma, Visible light assisted radical-polar/polar-radical crossover reactions in organic synthesis. *Adv. Synth. Catal.* **363**, 3146–3169 (2021).

73. X.-T. Li, L. Lv, T. Wang, Q.-S. Gu, G.-X. Xu, Z.-L. Li, L. Ye, X. Zhang, G. J. Cheng, X.-Y. Liu, Diastereo- and enantioselective catalytic radical oxysulfonylation of alkenes in β,γ-unsaturated ketoximes. *Chem* **6**, 1692–1706 (2020).

74. L.-W. Fan, J.-B. Tang, L.-L. Wang, Z. Gao, J.-R. Liu, Y.-S. Zhang, D.-L. Yuan, L. Qin, Y. Tian, Z.-C. Chen, F. Liu, J.-M. Xiang, P.-J. Huang, W.-L. Liu, C.-Y. Xiao, C. Luan, Z.-L. Li, X. Hong, Z. Dong, Q.-S. Gu, X.-Y. Liu, Copper-catalysed asymmetric cross-coupling reactions tolerant of highly reactive radicals. *Nat. Chem.*, 10.1038/s41557-025-01970-1 (2025).

75. Y. Wu, D. Kim, T. S. Teets, Photophysical properties and redox potentials of photosensitizers for organic photoredox transformations. *Synlett* **33**, 1154–1179 (2022).

76. Z. Zou, W. Zhang, Y. Wang, L. Kong, G. Karotsis, Y. Wang, Y. Pan, Electrochemically promoted fluoroalkylation–distal functionalization of unactivated alkenes. *Org. Lett.* **21**, 1857–1862 (2019).

77. K. Donabauer, M. Maity, A. L. Berger, G. S. Huff, S. Crespi, B. König, Photocatalytic carbanion generation – Benzylation of aliphatic aldehydes to secondary alcohols. *Chem. Sci.* **10**, 5162–5166 (2019).

78. S. Grotjahn, B. König, Photosubstitution in dicyanobenzene-based photocatalysts. *Org. Lett.* **23**, 3146–3150 (2021).

79. W. L. Jorgensen, S. Boudon, T. B. Nguyen, Structure and binding for Rebek's diacid in chloroform. A demure host for pyrazine. *J. Am. Chem. Soc.* **111**, 755–757 (1989).

80. T. Qin, G. Lv, Q. Meng, G. Zhang, T. Xiong, Q. Zhang, Cobalt-catalyzed radical hydroamination of alkenes with *N*-fluorobenzenesulfonimides. *Angew. Chem. Int. Ed. Engl.* **60**, 25949–25957 (2021).

81. Q. Zhang, S. Wang, Q. Zhang, T. Xiong, Q. Zhang, Radical addition-triggered remote migratory isomerization of unactivated alkenes to difluoromethylene-containing alkenes enabled by bimetallic catalysis. *ACS Catal.* **12**, 527–535 (2022).

82. H. R. Miao, M. H. Guan, T. Xiong, G. Zhang, Q. Zhang, Cobalt-catalyzed enantioselective hydroamination of arylalkenes with secondary amines. *Angew. Chem. Int. Ed. Engl.* **62**, e202213913 (2022).

83. W. Xu, Q. Shao, C. Xia, Q. Zhang, Y. Xu, Y. Liu, M. Wu, Visible-light-induced selective defluoroalkylations of polyfluoroarenes with alcohols. *Chem. Sci.* **14**, 916–922 (2023).

84. N. A. Romero, D. A. Nicewicz, Organic photoredox catalysis. *Chem. Rev.* **116**, 10075–10166 (2016).

85. M. Frisch, G. Trucks, H. Schlegel, G. Scuseria, M. Robb, J. Cheeseman, et al. Gaussian 16, Revision C.01, Gaussian Inc., Wallingford CT (2019).

86. Y. Zhao, D. Truhlar, Density functionals with broad applicability in chemistry. *Acc. Chem. Res.* **41**, 157–167 (2008).

87. S. Grimme, J. Antony, S. Ehrlich, H. A. Krieg, A consistent and accurate ab initio parametrization of density functional dispersion correction (DFT-D) for the 94 elements H-Pu. *J. Chem. Phys.* **132**, 154104 (2010).

88. S. Grimme, S. Ehrlich, L. Goerigk, Effect of the damping function in dispersion corrected density functional theory. *J. Comput. Chem.* **32**, 1456–1465 (2011).

89. P. C. Hariharan, J. A. Pople, The influence of polarization functions on molecular orbital hydrogenation energies. *Theor. Chim. Acta.* **28**, 213–222 (1973).

90. W. Hehre, R. Ditchfield, J. Pople, Self—consistent molecular orbital methods. XII. Further extensions of Gaussian—Type Basis Sets for Use in Molecular Orbital Studies of Organic molecules. *J. Chem. Phys.* **56**, 2257–2261 (1972).

91. P. Stephens, F. Devlin, C. Chabalowski, M. Frisch, Ab initio calculation of vibrational absorption and circular dichroism spectra using density functional force fields. *J. Phys. Chem.* **98**, 11623–11627 (1994).

92. C. Gonzalez, H. Schlegel, Reaction path following in mass-weighted internal coordinates. *J. Phys. Chem.* **94**, 5523–5527 (1990).

93. K. Fukui, The path of chemical reactions - The IRC approach. *Acc. Chem. Res.* **14**, 363–368 (1981).

94. J. Lovie-Toon, C. Tram, B. Flynn, E. Krenske, Mechanisms of carbonyl activation by BINOL *N*-triflylphosphoramides: Enantioselective Nazarov cyclizations. *ACS Catal.* **7**, 3466–3476 (2017).

95. A. Moran, A. Hamilton, C. Bo, P. Melchiorre, A mechanistic rationale for the 9-amino(9-deoxy)epi cinchona alkaloids catalyzed asymmetric reactions via iminium ion activation of enones. *J. Am. Chem. Soc.* **135**, 9091–9098 (2013).

96. A. Marenich, C. Cramer, D. Truhlar, Universal solvation model based on solute electron density and on a continuum model of the solvent defined by the bulk dielectric constant and atomic surface tensions. *J. Phys. Chem. B* **113**, 6378–6396 (2009).

97. W. Humphrey, A. Dalke, K. Schulten, VMD: Visual molecular dynamics. *J. Mol. Graph.* **14**, 33–38 (1996).
